# Rhodium‐Catalyzed Synthesis of Enantioenriched α‐Substituted Primary Amines Through Asymmetric Transfer Hydrogenation

**DOI:** 10.1002/chem.71466

**Published:** 2026-07-24

**Authors:** Chonghuo Liu, Guillaume Lefèvre, Qin Yin, Phannarath Phansavath, Virginie Ratovelomanana‐Vidal

**Affiliations:** ^1^ Institute of Chemistry for Life and Health Sciences CNRS, CSB2D Team Paris France; ^2^ Faculty of Pharmaceutical Sciences Shenzhen University of Advanced Technology Shenzhen Guangdong China

**Keywords:** amines, asymmetric transfer hydrogenation, enantioselectivity, imines, rhodium

## Abstract

The preparation of a series of enantioenriched α‐substituted primary amines using rhodium‐catalyzed asymmetric transfer hydrogenation of *ortho‐*hydroxyaryl N─H imines is reported. The reaction proceeds under mild conditions using a low catalyst loading and ammonium formate as the hydrogen source, delivering the reduced compounds in high yields (up to 99%) and enantioselectivities (up to 99% ee).

## Graphical abstract

Enantioenriched α‐substituted primary amines are efficiently prepared by rhodium‐catalyzed asymmetric transfer hydrogenation of *ortho‐*hydroxyaryl N─H imines under mild conditions using a low catalyst loading and ammonium formate as the hydrogen source in high yields (up to 99%) and enantioselectivities (up to 99% ee).

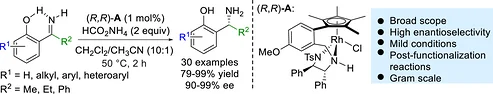

## 1 Introduction

Enantioenriched *α*‐substituted primary amines play crucial roles in drug design and synthesis, and can serve as efficient chiral ligands for asymmetric catalysis or as chiral auxiliaries in asymmetric catalysis [1, 2, 3]. Owing to their high reactivity and limited steric differentiation, however, the selective synthesis of chiral primary amines remains a significant challenge. Over the past decades, a variety of strategies have been developed, including asymmetric hydrogenation, reductive amination, and biocatalytic approaches, which have enabled increasingly efficient and sustainable routes to enantiomerically enriched amines. In addition, chiral primary amines have found widespread applications in organocatalysis and as building blocks for the construction of more complex architectures. In this context, the chiral 2‐(1‐aminoethyl) phenol unit is a privileged pharmacophore in bioactive compounds such as Safracin B [4] and D‐Tetrandrine [5] (Figure 1). The development of general, practical, and highly selective methods for the preparation of these enantioenriched *ortho*‐hydroxy‐benzylamine moieties continues to be an important objective in synthetic chemistry. General approaches to these structures include biocatalytic methods [6] or chemical resolution [7], diastereoselective reduction of imines [8], and transition metal‐catalyzed [9, 10, 11, 12] or organocatalyzed [13, 14] enantioselective reduction of *N*‐protected enamides, enamines, and imines.

**FIGURE 1:**
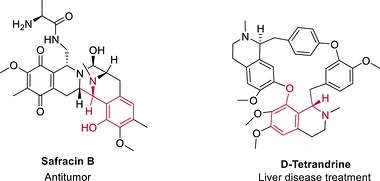
Biologically active compounds containing α‐substituted primary amine scaffolds.

In this area, Wang et al. developed a chiral phosphoric acid‐catalyzed enantioselective transfer hydrogenation of unprotected *ortho*‐hydroxyaryl alkyl or aryl N─H ketimines to access the corresponding primary amines with enantioselectivities ranging from 81% to 99% (Scheme 1a) [15, 16]. Mangion, Chen et al. described an enantioselective synthesis of an HCV NS5a inhibitor MK‐8742 that relies on the asymmetric transfer hydrogenation (ATH) [17, 18, 19, 20, 21, 22, 23, 24, 25] of an N─H imine using a ruthenium catalyst and ammonium formate as the hydrogen source (Scheme 1b) [26]. During their work on the ATH of *ortho*‐hydroxyphenyl ketones, Wills et al. also tested the Ru‐catalyzed asymmetric reduction of the parent imines on two examples using ruthenium tethered complexes that allowed the formation of the amines with enantioselectivities ranging from 32% to 97% ee [27]. The efficient ATH of stable N─H imines using a rhodium catalyst in the presence of HCO_2_NH_4_ was reported by Yang, Yin et al. for the preparation of 2‐(1‐aminoalkyl)anilines, obtained with enantioselectivities ranging from 79% to 99% ee (Scheme 1c) [28]. This study also included the preparation of (*R*)‐2‐(1‐aminoethyl)phenol and (*R*)‐2‐(1‐aminoethyl)‐4‐fluorophenol in 99% ee using the same reaction. On the other hand, Yin et al. developed a highly enantioselective ruthenium‐catalyzed asymmetric reductive amination of *ortho*‐OH‐substituted diaryl and sterically hindered ketones using ammonium salts under 50 atm of hydrogen pressure at 80°C, affording within 24 h enantioenriched chiral primary diarylmethylamines and sterically demanding benzylamines [29]. In this context and as part of our ongoing efforts toward the development of efficient methods for the asymmetric reduction of functionalized ketones and imines [30, 31, 32], we report herein the synthesis of enantioenriched *ortho*‐hydroxy‐benzylamine derivatives via rhodium‐catalyzed asymmetric transfer hydrogenation using a complex developed in our group (Scheme 1d).

**SCHEME 1:**
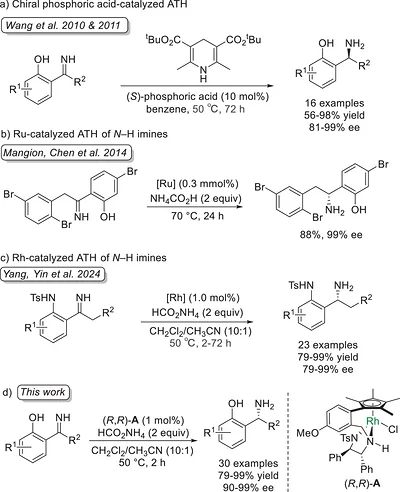
(a) ATH on *ortho*‐hydroxyaryl alkyl or aryl N─H ketimines using (*S*)‐phosphoric acid catalyst; (b) Ru‐catalyzed ATH for the synthesis of an HCV NS5a inhibitor MK‐8742 intermediate; (c) ATH of N─H imines using a Rh catalyst in the presence of HCO_2_NH_4_; (d) synthesis of enantioenriched *ortho*‐hydroxy‐benzylamines via Rh‐catalyzed ATH using a complex developed in our group.

## 2 Results and Discussion

The asymmetric transfer hydrogenation (ATH) was initially investigated using 2‐(1‐iminoethyl)phenol **1a** as a model substrate, in the presence of 1 mol% of the rhodium complex (*R*,*R*)‐**A** developed in our group [33, 34] and ammonium formate (2 equiv) as the hydrogen source. Given its classification as a green solvent, 2‐MeTHF was first evaluated. Under these conditions at 50°C, the desired amine **2a** was obtained in 91% yield within 2 h, albeit with moderate enantioselectivity (60% ee) (Table 1, entry 1). Changing the solvent to dichloromethane significantly improved enantioinduction (97% ee), although the yield decreased to 71% (Table 1, entry 2). In contrast, acetonitrile afforded **2a** in nearly quantitative yield (99%), but with diminished enantioselectivity (75% ee) (Table 1, entry 3). Notably, a 1:1 mixture of CH_2_Cl_2_/CH_3_CN provided an improved balance, delivering **2a** in 98% yield and 90% ee (Table 1, entry 4). Gratifyingly, both excellent yield (97%) and enantioselectivity (99% ee) were achieved using a 10:1 CH_2_Cl_2_/CH_3_CN mixture (Table 1, entry 5). Additionally, tethered Rh(III) complexes (*R*,*R*)‐**B**, (*R*,*R*)‐**C** and (*R*,*R*)‐**D**, also developed in our group, were evaluated but did not surpass the performance of (*R*,*R*)‐**A** (Table 1, entries 6–8). On the basis of these results, the optimal conditions were established as 1 mol% of (*R*,*R*)‐**A** and 2 equiv of ammonium formate in CH_2_Cl_2_/CH_3_CN (10:1) at 50°C.

**TABLE 1:** Optimization of the reaction conditions for the ATH of *ortho‐*hydroxyaryl NH imines.^a^

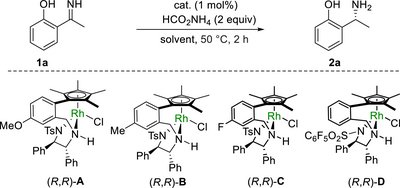				
Entry	Cat.	Solvent	Yield (%)^b^	ee (%)^c^
1	(*R,R*)‐**A**	2‐MeTHF	91	60
2	(*R,R*)‐**A**	DCM	71	97
3	(*R,R*)‐**A**	ACN	99	75
4	(*R,R*)‐**A**	DCM/ACN (1:1)	98	90
5	(*R,R*)‐**A**	DCM/ACN (10:1)	97	99
6^d^	(*R,R*)‐**B**	DCM/ACN (10:1)	94	99
7^e^	(*R,R*)‐**C**	DCM/ACN (10:1)	92	53
8^e^	(*R,R*)‐**D**	DCM/ACN (10:1)	90	63

^a^ Reaction conditions: **1a** (41 mg, 0.3 mmol, 1.0 eq.), HCO_2_NH_4_ (37.8 mg, 0.6 mmol, 2.0 eq.), cat. (1 mol%), solvent (3 mL), 50°C.

^b^ Isolated yield.

^c^ Enantiomeric excesses were determined by HPLC analysis of the diacetylated derivatives with a chiral stationary phase.

^d^ Reaction time of 12 h.

^e^ Reaction time of 15 h.

With the optimized conditions in hand, the substrate scope was investigated using a series of *ortho*‐hydroxybenzylamine derivatives **1a–1ad** bearing electronically and sterically diverse substituents, which were readily prepared from the corresponding ketones by reaction with ammonia in methanol at room temperature [35] (Scheme 2). All N─H ketimines **1a**–**1ad** were stable due to the presence of the *ortho*‐hydroxy group that ensures a strong intramolecular hydrogen bonding with the imine functional group. Across this series, the asymmetric transfer hydrogenation exhibited consistently high levels of stereocontrol. Electron‐donating substituents (Me, OMe) at the *ortho*‐, *meta*‐, and *para*‐positions of the phenol ring had minimal influence on enantioselectivity, with the corresponding amines obtained in 96%–99% ee (**2b**–**2f**). Likewise, *para*‐electron‐withdrawing groups (Cl, Br, CF_3_, CN, NO_2_, CO_2_Me, Ph) furnished products with similarly high enantioselectivities (95%–99% ee; **2h**–**2n**). These results suggest that electronic modulation of the arene has a limited effect on the enantiodetermining step consistent with a transition state weakly affected by the electronic polarization of the substrate aryl backbones. Notably, the fluoro‐substituted substrate **1g** afforded a lower enantioselectivity (90% ee), which may reflect subtle perturbations in arene electronics and/or conformational preferences that weaken key stabilizing interactions.

**SCHEME 2:**
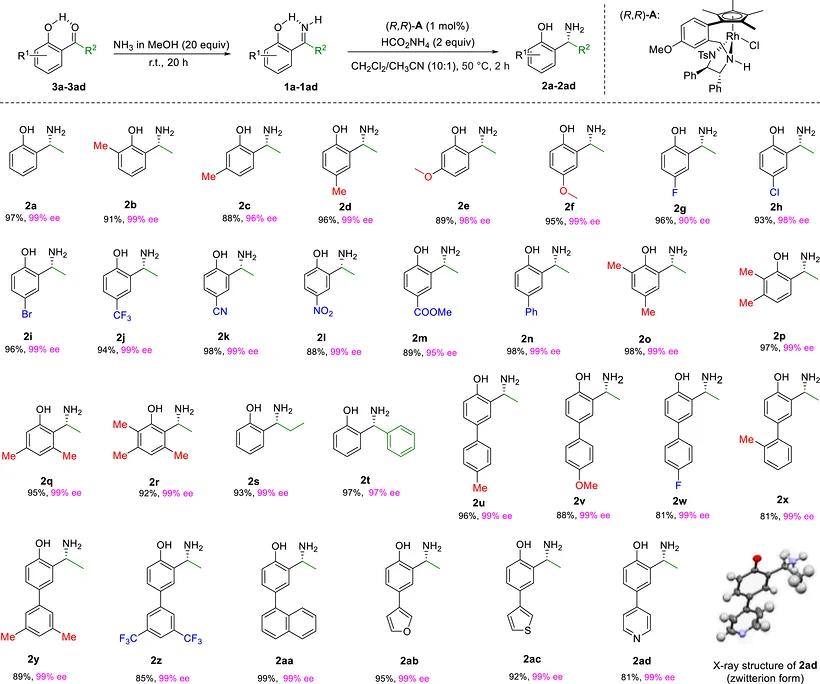
Scope of the ATH. Reaction conditions: imine (0.3 mmol, 1 equiv), (*R*,*R*)‐**A** (0.003 mmol, 2.3 mg, 1 mol%), HCO_2_NH_4_ (0.6 mmol, 37.8 mg, 2 equiv), CH_2_Cl_2_/CH_3_CN (10:1) (2.7 mL/0.27 mL), 50°C, 2 h. Isolated yield. Enantiomeric excesses were determined by HPLC or SFC analysis of the diacetylated derivatives with a chiral stationary phase.

Substrates bearing multiple methyl substituents (**2o**–**2r**) were converted in high yields (92%–98%) and enantioselectivities (up to 99% ee), indicating that increased steric demand on the aryl ring is well accommodated. Furthermore, variation of the imine substituent (from Me to Et, Ph) had little effect on stereoselectivity (**2s**–**2t**, 97%–99% ee), suggesting that differentiation between the imine substituents is not the primary determinant of enantioinduction. A range of *para*‐aryl, naphthyl, and heteroaryl substituents was also tolerated, with substrates bearing substituted phenyl groups (e.g., 4‐Me, 4‐OMe, 4‐F, 2‐Me, 3,5‐Me_2_, 3,5‐(CF_3_)_2_) affording products **2u**–**2z** with uniformly excellent enantioselectivities (up to 99% ee). Similarly, naphthyl, furyl, thienyl, and pyridinyl derivatives (**2aa**–**2ad**) displayed comparable levels of enantioinduction. The broad tolerance toward π‐extended and heteroaromatic systems further supports the involvement of stabilizing π‐interactions in the enantiodetermining transition state. The absolute configurations of **2a–2g**, **2i**, **2l**, and **2q** were assigned by comparison of optical rotation values with literature data [15, 28, 29], while the configuration of **2ad** was unambiguously determined to be *(R)* by X‐ray crystallographic analysis [36] of its zwitterionic form. By analogy, the same absolute configuration was assigned to the remaining products.

Moreover, comparative kinetic studies by ^19^F NMR monitoring of the ATH of **1g** using catalysts (*R*,*R*)‐**A**, (*R*,*R*)‐**B**, (*R*,*R*)‐**C**, and (*R*,*R*)‐**D** were also performed (Figure 2). A 50% conversion of **2g** was reached after ca. 50, 120, 100, and 140 min for those catalysts, respectively. Catalyst (*R*,*R*)‐**A** proved to outcompete the other screened catalysts, and achieved full conversion of **1g** after 120 min, whereas (*R*,*R*)‐**B**, (*R*,*R*)‐**C**, and (*R*,*R*)‐**D** led to similar results after ca. 210 min. A sigmoidal shape was observed for the formation of **2g**, suggesting the existence of thermodynamic equilibria before the generation of catalytically active species. However, this induction period seems significantly shortened for catalyst (*R*,*R*)‐**A**, in line with its more marked efficiency.

**FIGURE 2:**
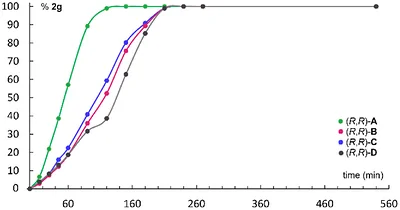
^19^F NMR kinetic monitoring of the ATH of **1g** catalyzed by (*R*,*R*)‐**A**, (*R*,*R*)‐**B**, (*R*,*R*)‐**C**, and (*R*,*R*)‐**D** in the optimized conditions of Table 1.

Origin of the enantioselectivity of this ATH process was investigated by DFT analysis (Gaussian 2016, revision C.01 was used at a M06‐D3 level using PCM solvation model; see Supporting Information for the corresponding references). ATH of imine **1a** using Rh^III^‐H hydride **I** formed by decarboxylation of the formate ion HCOO^–^ at (*R*,*R*)‐**A** was taken as a benchmark reaction (Figure 3). The most stable isomer of **1a** optimized in silico was its hydrogen‐bonded NH_2_^+^‐O^−^ zwitterionic form **II**, located ca. 9.7 kcal.mol^−1^ lower than non‐charged **II’**, which mirrors the structure of **2ad** obtained in the solid state (Scheme 2). The phenoxide moiety of **II** can then be involved in the formation of a H‐bonded adduct with the Rh‐ligated N─H group of **I**, leading to **III**, from which ATH can take place. Major enantiomer **2a** can be formed within Rh‐to‐carbon hydride transfer along with proton transfer from the Rh‐bound N─H group onto the phenoxide moiety (**TS1**), for an overall barrier of 6.5 kcal mol^−1^ from adduct **III**. This transition state allows a good facial selection due to the development of CH–π interactions between the CH_3_ groups of the Cp*Rh^III^ with arene moiety of **II**. Conversely, a similar transition state leading to the Rh‐to‐C hydride transfer and N‐to‐O proton transfer proceeding on the other face of **II**, precluding the development of stabilizing CH–π interactions, was located 3.9 kcal mol^−1^ higher in energy than **TS1** (**TS2**), leading to the minor amine enantiomer ***ent‐*2a**. This key role of the development of CH–π interaction in facial stereoselection echoes what was reported in the ATH of aryl ketones using (*R,R*)‐**A** [37], or in DKR of β‑substituted α‑diketones using Rh or Ru catalysts [38]. Alternatively, a transition state maintaining the CH–π interactions but proceeding on the other face of **II**, which requires breaking of the intramolecular hydrogen bond of **II**, was located higher than **TS1**, leading to ***ent‐*2a** (**TS3**). The transition state more classically observed in the ATH of aryl ketones mediated by **I** features (i) the transfer of the hydride onto the electrophilic carbon of the ketone, and (ii) the transfer of the proton from the Rh‐bound N─H group onto the oxygen atom of the C═O substrate. In the case of **1a**, a similar transition state proceeding from **II’** with direct ATH onto its C═N bond was located on the surface, leading to the unfavored enantiomer of **2a** (**TS4** leading to ***ent*‐2a**, located at 14.8 kcal.mol^−1^, much higher than **TS1‐3**). No similar transition state featuring a direct ATH on the C═N bond of **II** could be located. Overall, **TS1** accounts for the observed facial selectivity, leading to the final adduct **IV**. A 3.7 kcal mol^−1^ free energy difference between the lowest transition states leading to the major and minor enantiomers (**2a** and ***ent*‐2a**) explains the observed selectivity. The lower selectivity of catalyst (*R*,*R*)‐**C** has also been reproduced in silico, with a more modest energy difference of 2.6 kcal mol^−1^ between the transition states leading to **2a** and ***ent‐*2a** being computed, mirroring the lower facial selection (Table 1, entry 7 – see Supporting Information for the computed data).

**FIGURE 3:**
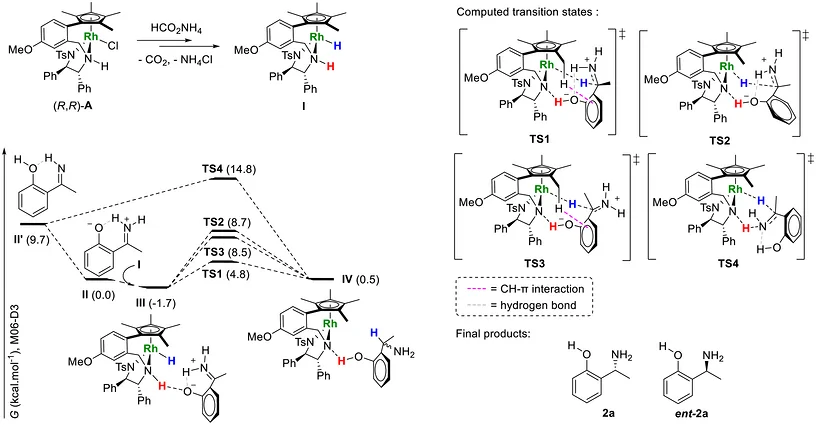
DFT‐computed ATH leading from **1a** (**II** or **II’**) to **2a**; both enantiomers **2a** and ***ent‐*2a** led to near‐energetic final adducts **IV** after ATH reaction.

Based on previous studies on similar systems alongside this DFT computational analysis, a full plausible catalytic cycle for the asymmetric transfer hydrogenation of 2‐(1‐iminoethyl)phenol **1a** is presented in Scheme 3. The addition of ammonium formate to the precatalyst (*R*,*R*)‐**A** followed by decarboxylation leads to the rhodium species **Int1** after elimination of ammonium chloride and CO_2_.

**SCHEME 3:**
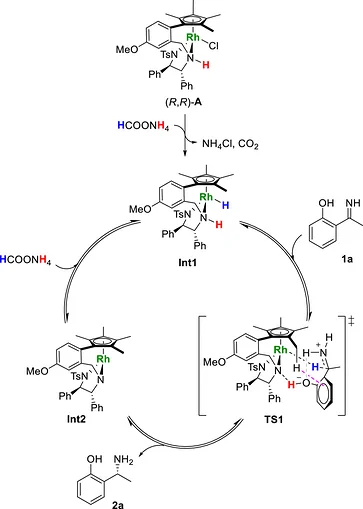
Proposed catalytic cycle.

**Int1** would then react with the imine via directing O─H⋯N and Rh─H⋯C interactions, and thus give the asymmetric reduction of **1a** into the amine **2a**. This step would reform the 16‐electron amino rhodium complex **Int2** that would react with a new molecule of ammonium formate and give **Int1** to complete the catalytic cycle.

The high enantioselectivity observed in the asymmetric transfer hydrogenation of **1** is likely attributable to a well‐established edge‐to‐face CH/π interaction between the Me groups of the *η* ^5^‐Cp* ligand and the phenyl ring substituent of the imine. In the proposed transition state, this stabilizing CH/π interaction governs the stereochemical outcome, leading to high enantiomeric excess (Scheme 3).

The practicality of this Rh‐catalyzed asymmetric transfer hydrogenation (ATH) was demonstrated by performing the reaction on a gram scale without erosion of either yield or enantioselectivity. Under the standard conditions, amine **2a** was obtained with yield and ee values identical to those observed on a smaller scale (Scheme 4), highlighting the robustness and scalability of the protocol. In addition, the resulting ATH products constitute versatile intermediates for further derivatization, enabling functionalization either on the aryl ring or at the amine moiety. For instance, biaryl derivative **2v** was synthesized in 89% yield from **2i** via a Suzuki–Miyaura coupling using 1 mol% Pd(PPh_3_)_4_, K_2_CO_3_ as the base, and 4‐methoxyphenylboronic acid as the coupling partner. Alternatively, conjugate addition of **2i** to *tert*‐butyl acrylate in MeOH at 50°C furnished the corresponding adduct **5i** in 77% yield. These transformations illustrate the synthetic utility of the enantioenriched amine products as modular building blocks.

**SCHEME 4:**
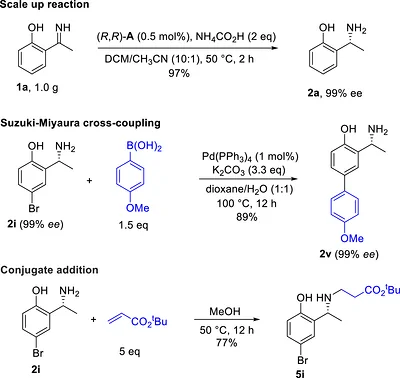
Gram‐scale and post‐functionalization reactions.

## 3 Conclusion

In summary, we have reported an efficient method for the preparation of enantioenriched 2‐(1‐aminoethyl)phenols via rhodium‐catalyzed asymmetric transfer hydrogenation of *ortho*‐hydroxybenzylimine derivatives, employing the tethered Rh(III) complex (*R*,*R*)‐**A** developed in the group in combination with ammonium formate as the hydrogen donor. The transformation proceeds under mild conditions and exhibits broad substrate scope, tolerating both electron‐donating and electron‐withdrawing substituents, as well as sterically demanding and heteroaromatic groups. High levels of enantioselectivity (90%–99% ee) are consistently achieved across the substrate range. Comparative kinetic studies highlight the performance of catalyst (*R*,*R*)‐**A**, and DFT calculations demonstrate the key role of CH–π interactions alongside hydrogen bonds in the enantioinduction of the reaction. Moreover, the method is readily scalable and provides access to enantioenriched amines that can be further diversified, underscoring its potential utility in the synthesis of valuable chiral building blocks.

## Conflicts of Interest

The authors have no conflicts of interest.

## Supporting information



The authors have cited additional references within the Supporting Information [39, 40, 41, 42, 43, 44, 45, 46, 47, 48, 49].
**Supporting File 1**: chem71466‐sup‐0001‐SuppMat.pdf

## Data Availability

The data that support the findings of this study are available in the Supporting Information of this article.
