# Supplementary material for: Rhodium‐Catalyzed Synthesis of Enantioenriched α‐Substituted Primary Amines Through Asymmetric Transfer Hydrogenation
Source: Chemistry. 2026 Jul 24;32(39):e71466. doi: 10.1002/chem.71466 (PMC13715052; doi:10.1002/chem.71466)
Supplement: Supplementary file 1 [file CHEM-32-e71466-s001.pdf]

Supporting information

Rhodium-Catalyzed Synthesis of Enantioenriched *α*- Substituted Primary Amines through Asymmetric Transfer Hydrogenation

Chonghuo Liu,[a] Guillaume Lefèvre,[a] Qin Yin,[b] Phannarath Phansavath,*[a]and Virginie Ratovelomanana-Vidal*[a]

[a] PSL University, Chimie ParisTech, Institute of Chemistry for Life & Health Sciences, CNRS UMR8060, CSB2D Team, 11 rue Pierre et Marie Curie, 75005 Paris France
[b] Faculty of Pharmaceutical Sciences, Shenzhen University of Advanced Technology, Shenzhen, Guangdong 518107, China

Table of Contents

I. Experimental Section 3

I.1 General Information 3

I.2 Details of Optimization Studies 3

I.3 Preparation of Substrates 4

II. Experimental Procedures and Characterization Data 5

III. Postfunctionalization reactions of ortho-hydroxyaryl amine **2i**..44

IV. Kinetic studies……………………………………………………………………….47

V. DFT calculations………………………………………………………………….....50

VI. Spectral Data for Compounds...81

VI.1 ^{1}H, ^{13}C and ^{19}F NMR spectra for compounds **3a-3ad**……………………………………81

VI.2 HPLC and SFC chromatograms for compounds **2a-2ad** after diacetylation……………167

VII. Crystallographic data...197

VIII. References 199

I. Experimental Section

I. 1. General Information

All reactions were carried out under argon atmosphere. All reagents were obtained from commercial sources and used as supplied unless stated otherwise.

All reactions were performed using dry and degassed solvents unless stated otherwise. DCM was purified by Solvent Purification System (SPS) and all the solvents (ACN, 2-MeTHF, DCM) were stored over activated 4Å molecular sieves prior to use.

^{1}H NMR (400 MHz, 500 MHz), ^{13}C NMR (101 MHz) and ^{19}F NMR (376 MHz) spectra were recorded on a Brucker Avance III HD equipment and processed using MestReNova software. NMR chemical shifts (δ) are reported in parts per million (ppm) and referenced to the solvent signals. Coupling constants (*J*) are given in hertz (Hz). The abbreviations s, d, t, q and m stand for singlet, doublet, triplet, quadruplet and multiplet accordingly.

High resolution mass spectrometric (HRMS) analyses were measured on Agilent 6546 LC/QTOF at Chimie ParisTech. Column purifications were performed using VWR Chemicals Silica Gel 60 (40-60 µm) or using the CombiFlash NEXTGEN 300+. Optical rotations were measured on a Jasco P-1010 polarimeter. The enantiomeric excess were determined by HPLC and SFC analysis by using Daicel CHIRALPAK® (OD-3, AD-H and IE) columns.

I. 2. Details of Optimization Studies

Table S1: Screening of precatalysts for ATH reaction[a]

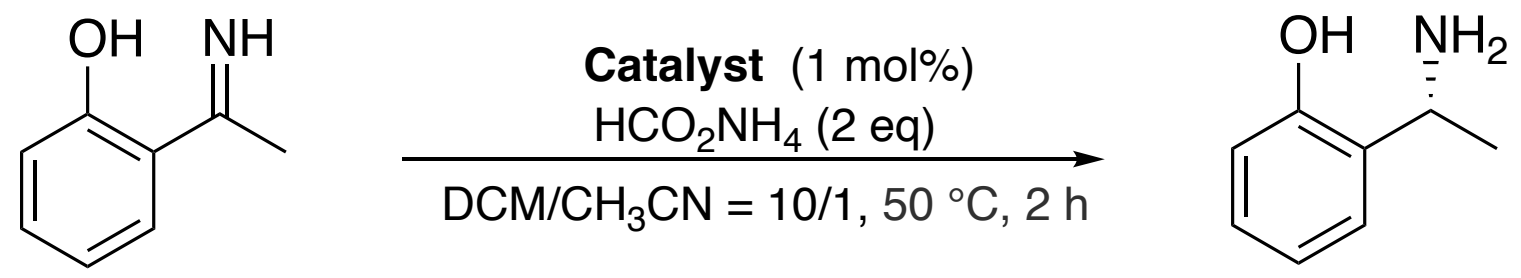

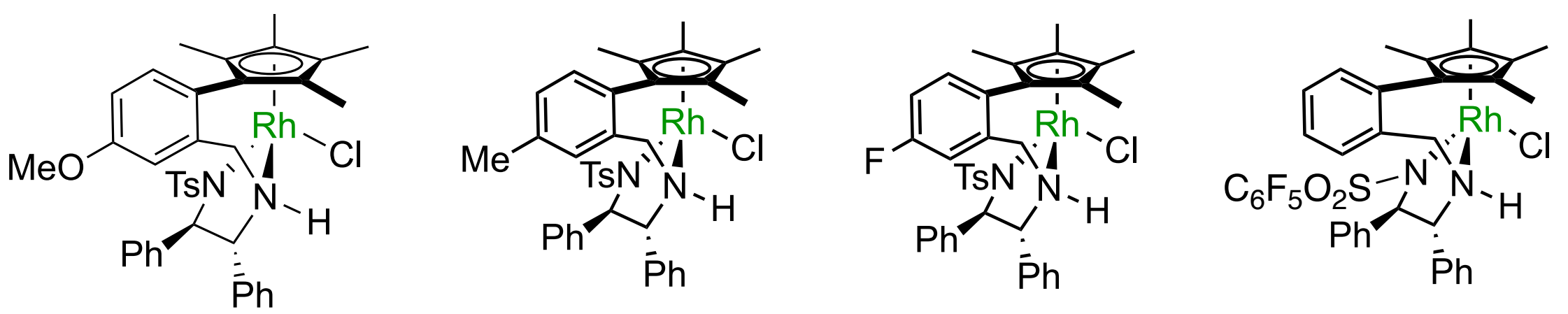

(*R*,*R*)-**A** (*R*,*R*)-**B** (*R*,*R*)-**C** (*R*,*R*)-**D**

Entry	Catalyst.	Solvent	Yield(%)[b]	ee(%)[c]
1	(*R*,*R*)-**A**	2-MeTHF	91	60
2	(*R*,*R*)-**A**	DCM	71	97
3	(*R*,*R*)-**A**	ACN	99	75
4	(*R*,*R*)-**A**	DCM/ACN(1:1)	98	90

5	(*R*,*R*)-**A**	DCM/ACN(10:1)	97	99
6	(*R*,*R*)-**B**	DCM/ACN(10:1)	94	99
7	(*R*,*R*)-**C**	DCM/ACN(10:1)	92	53
8	(*R*,*R*)-**D**	DCM/ACN(10:1)	90	63

[a] Reaction conditions: imines (41 mg, 0.3 mmol), cat. (1 mol%), $HCOONH_4$ (2 eq.), Solvent (3 mL).
[b] Isolated yield. [c] Enantiomeric excesses were determined by HPLC analysis of the diacetylated derivatives with a chiral stationary phase. [d] Reaction time of 12 h. [e] Reaction time of 15 h.

I. 3. Preparation of Substrates

Procedure A : General procedure for the preparation of ketones:

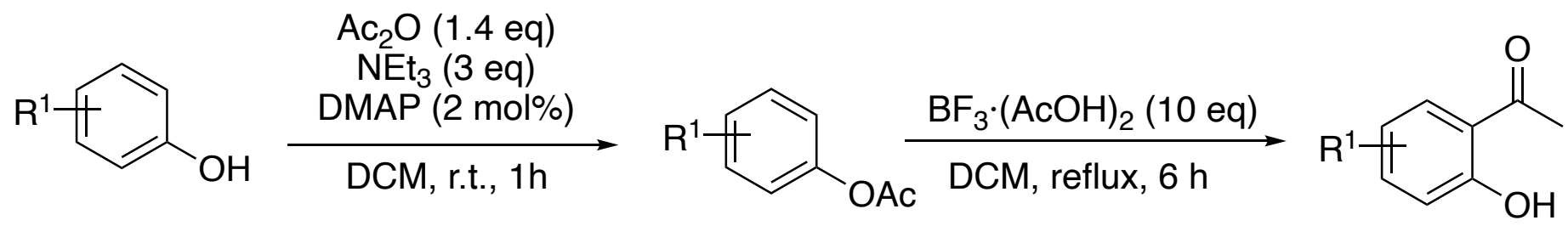

A 100 mL round-bottomed flask was charged with phenol (10 mmol), Ac_2O (14 mmol), NEt_3 (4.2 mL, 30 mmol), DMAP (25 mg, 0.2 mmol) and dichloromethane (30 mL) The resulting mixture was stirred for 1 hour at room temperature. After completion of the reaction (monitored by TLC), the reaction mixture was diluted with DCM and washed with sat. $NaHCO_3$ and brine. The organic phase was dried over anhydrous Na_2SO_4 and concentrated under vacuum.
The residue was purified by flash column chromatography on silica gel (10% ethyl acetate in petroleum ether) to afford the acetate. To a solution of acetate (10 mmol) in DCM (30 mL) was added $BF_3{\cdot}(AcOH)_2$ (13.8 mL, 10 eq) and the reaction was mixture was heated under reflux for 6 h. The residue was purified by flash column chromatography on silica gel (10% ethyl acetate in petroleum ether) to afford the ketone.

Procedure B : General procedure for the preparation of ketones:

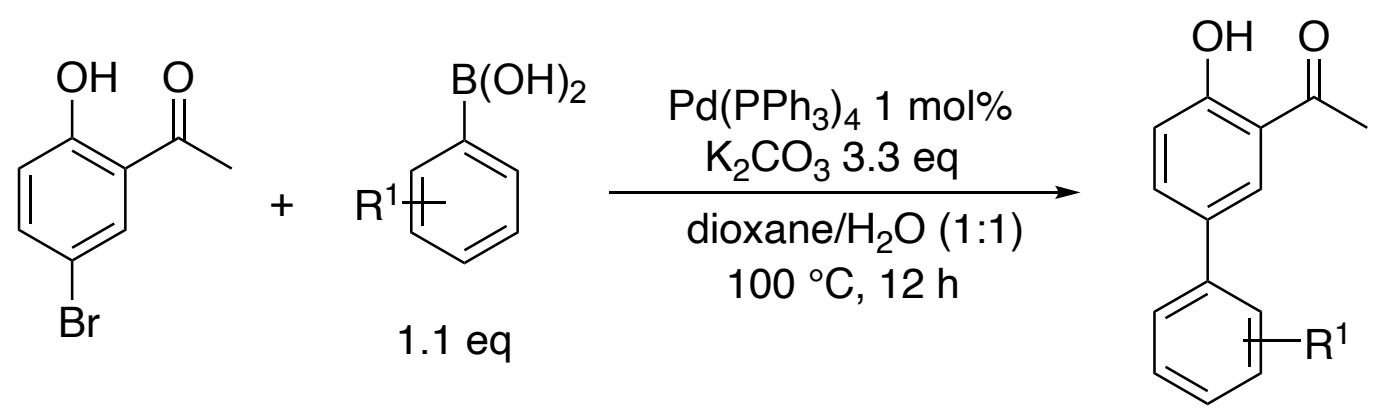

A 100 mL two-necked round-bottomed flask was charged with 1-(5-bromo-2-hydroxyphenyl)ethan-1-one (1.1 g， 5.0 mmol), aryl boronic acid (5.5 mmol, 1.1 equiv), K_2CO_3 (2.3 g, 16.5 mmol, 3.3 equiv) and $Pd(PPh_3)_4$ (58 mg, 0.05 mmol, 1 mol%). The tube was charged under an argon atmosphere, and the solvent of dioxane/H_2O (1/1, v/v, 30 mL) was injected by syringes. The reaction mixture was allowed to stir for 12 h under argon atmosphere at 100 °C. After completion of the reaction (monitored by TLC), the reaction mixture was diluted with EA and washed with sat. $NaHCO_3$ and brine. The organic phase was dried over anhydrous Na_2SO_4

and concentrated under vacuum. The residue was purified by flash column chromatography on silica gel (5% ethyl acetate in petroleum ether) to afford the target ketone.

Procedure C : General procedure for the preparation of imines:

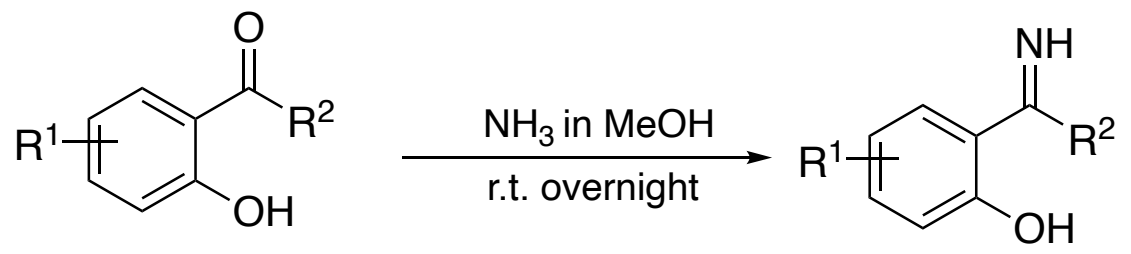

A 250 mL round bottom flask was charged under an argon atmosphere with ortho-hydroxyaryl ketone (10 mmol, 1 equiv.), and then 7N ammonia in methanol solution (100 mL, 20 equiv.). was injected into the flask. Upon addition of the ammonia, the colorless solution turned yellow and the suspension remained yellow throughout the reaction. The mixture was stirred vigorously under argon at room temperature for 20 hours and monitored by TLC for complete conversion. The suspension of imine was then filtered and the collected solids were rinsed with a minimal amount of methanol. The solids were dried under vacuum and purified by silica gel flash column chromatography (DCM/MeOH: 95/5) to give the imine.

II. Experimental Procedures and Characterization Data

1-(4-hydroxy-[1,1'-biphenyl]-3-yl)ethan-1-one[1] (3n):

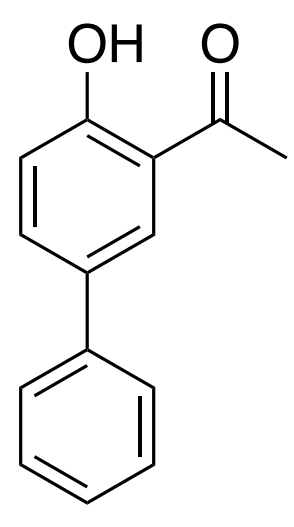

Chemical Formula: $C_{14}H_{12}O_2$
Exact Mass: 212.0837

This compound was prepared according to the general procedure B. After purification by column chromatography (silica gel: PE/EA: 95/5), the title compound was isolated in 91% yield as a white solid.

^{1}H NMR (400 MHz, $CDCl_3$) δ 12.27 (s, 1H), 7.92 (d, J = 2.3 Hz, 1H), 7.72 (dd, J = 8.6, 2.3 Hz, 1H), 7.58 – 7.50 (m, 2H), 7.50 – 7.41 (m, 2H), 7.41 – 7.31 (m, 1H), 7.07 (d, J = 8.6 Hz, 1H), 2.70 (s, 3H).

^{13}C NMR (101 MHz, $CDCl_3$) δ 204.7, 161.9, 140.1, 135.5, 132.5, 129.2, 129.1, 127.36, 126.9, 119.9, 119.0, 26.9.

2-hydroxy-3,4-dimethylacetophenone[2] **(3p):**

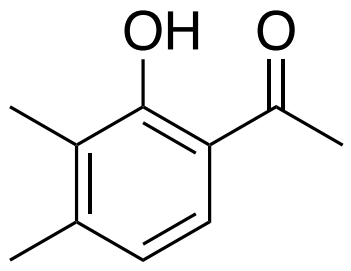

Chemical Formula: $C_{10}H_{12}O_2$
Exact Mass: 164.0837

This compound was prepared from 2,3-dimethylphenol (1.22 g, 10.0 mmol) according to the general procedure A. After purification by column chromatography (silica gel: PE/EA: 90/10), the title compound was isolated in 99% yield as a yellow oil.

^{1}H NMR (400 MHz, $CDCl_3$) δ = 12.67 (s, 1H), δ 7.48 (d, J = 8.2 Hz, 1H), 6.70 (d, J = 8.2 Hz, 1H), 2.60 (s, 3H), 2.31 (s, 3H), 2.17 (s, 3H).

^{13}C NMR (101 MHz, $CDCl_3$) δ = 204.4, 160.8, 146.2, 127.7 125.5, 120.5, 117.3, 26.63, 20.9, 11.1.

1-(2-hydroxy-4,6-dimethylphenyl)ethan-1-one[2] **(3q):**

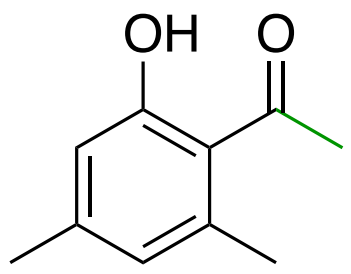

Chemical Formula: $C_{10}H_{12}O_2$
Exact Mass: 164.0837

This compound was prepared according to the general procedure A. After purification by column chromatography (silica gel: PE/EA: 90/10), the title compound was isolated in 91% yield as a yellow oil.

^{1}H NMR (400 MHz, $CDCl_3$) δ 12.68 (s, 1H), 6.68 (s, 1H), 6.57 (s, 1H), 2.67 (s, 3H), 2.59 (s, 3H), 2.30 (s, 3H).

^{13}C NMR (101 MHz, $CDCl_3$) δ = 205.6, 163.7, 146.3, 139.6, 124.6, 119.2, 116.9, 33.4, 24.8, 21.7.

2-hydroxy-3,4,6-trimethylacetophenone[2] **(3r):**

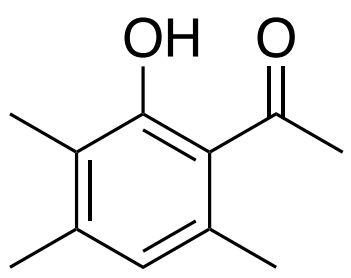

Chemical Formula: $C_{11}H_{14}O_2$
Exact Mass: 178.0994

This compound was prepared from 2,3,5-trimethylphenol (1.36 g, 10.0 mmol) according to the general procedure A. After purification by column chromatography (silica gel: PE/EA: 90/10), the title compound was isolated in 89% yield as a yellow oil.

^1H NMR (400 MHz, $CDCl_3$) δ 13.10 (s, 1H), 6.54 (s, 1H), 2.64 (s, 3H), 2.54 (s, 3H), 2.25 (s, 3H), 2.14 (s, 3H).

^{13}C NMR (101 MHz, $CDCl_3$) δ 205.9, 161.7, 144.4, 136.2, 124.7, 123.3, 118.8, 33.4, 24.7, 20.5, 11.3.

1-(4-hydroxy-4'-methyl-[1,1'-biphenyl]-3-yl)ethan-1-one[1] **(3u):**

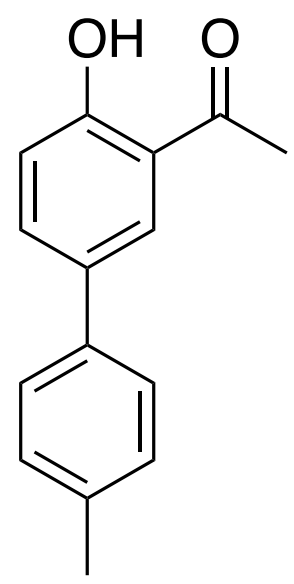

Chemical Formula: $C_{15}H_{14}O_2$
Exact Mass: 226.0994

This compound was prepared according to the general procedure B. After purification by column chromatography (silica gel: PE/EA: 95/5), the title compound was isolated in 82% yield as a yellow oil.

^1H NMR (400 MHz, $CDCl_3$) δ 12.24 (s, 1H), 7.89 (d, J = 2.3 Hz, 1H), 7.70 (dd, J = 8.6, 2.3 Hz, 1H), 7.53 – 7.39 (m, 2H), 7.27 – 7.25 (m, 2H), 7.05 (d, J = 8.6 Hz, 1H), 2.69 (s, 3H), 2.41 (s, 3H).

^{13}C NMR (101 MHz, $CDCl_3$) *δ* =204.8, 161.8, 137.3, 137.2, 135.4, 132.5, 129.8, 128.9, 126.7, 119.9, 119.0, 26.9, 21.2.

1-(4-hydroxy-4'-methoxy-[1,1'-biphenyl]-3-yl)ethan-1-one[1]**(3v):**

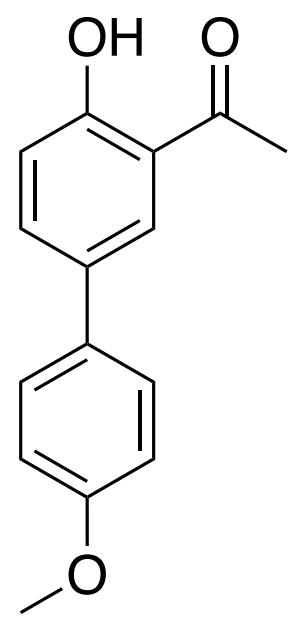

Chemical Formula: $C_{15}H_{14}O_3$
Exact Mass: 242.0943

This compound was prepared according to the general procedure B. After purification by column chromatography (silica gel: PE/EA: 95/5), the title compound was isolated in 88% yield as a yellow oil.

^{1}H NMR (400 MHz, $CDCl_3$) δ = 12.24 (s, 1H), 7.85 (d, J = 2.3 Hz, 1H), 7.65 (dd, J = 8.6, 2.3 Hz, 1H), 7.50 – 7.41 (m, 2H), 7.04 – 7.02 (m, 1H), 7.00 - 6.97 (m, 2H), 3.85 (s, 3H), 2.68 (s, 3H).

^{13}C NMR (101 MHz, $CDCl_3$) δ = 204.7, 161.4, 159.2 135.1, 132.6, 132.1, 128.6, 127.8, 119.8, 118.9, 114.5, 55.5, 26.8.

1-(4'-fluoro-4-hydroxy-[1,1'-biphenyl]-3-yl)ethan-1-one[4] **(3w):**

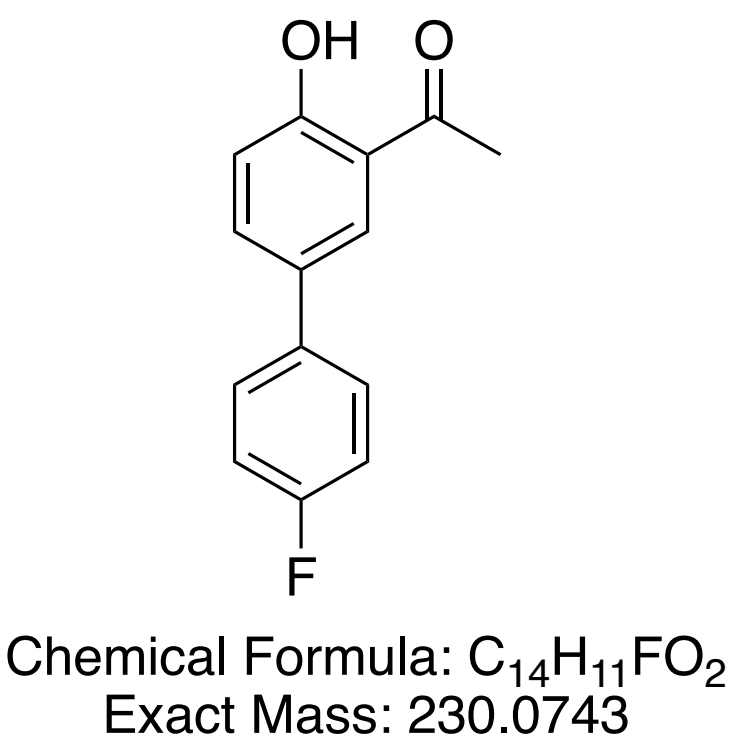

This compound was prepared according to the general procedure B. After purification by column chromatography (silica gel: PE/EA: 95/5), the title compound was isolated in 79% yield as a yellow oil.

^{1}H NMR (400 MHz, $CDCl_3$) δ 12.26 (s, 1H), 7.85 (d, J = 2.3 Hz, 1H), 7.65 (dd, J = 8.6, 2.3 Hz, 1H), 7.53 – 7.43 (m, 2H), 7.19 – 7.02 (m, 3H), 2.69 (s, 3H).

^{13}C NMR (101 MHz, $CDCl_3$) δ = 204.6, 162.5 (d, $^{1}J_{C\text{-}F}$ = 247.4 Hz), 161.9, 136.3 (d, $^{4}J_{C\text{-}F}$ = 3.3 Hz), 135.3, 131.6, 129.0, 128.4 (d, $^{3}J_{C\text{-}F}$ = 8.0 Hz), 119.9, 119.1, 115.9 (d, $^{2}J_{C\text{-}F}$ = 21.21 Hz), 26.9.

^{19}F NMR (376 MHz, $CDCl_3$) δ = -115.60.

1-(4-hydroxy-2'-methyl-[1,1'-biphenyl]-3-yl)ethan-1-one **(3x):**

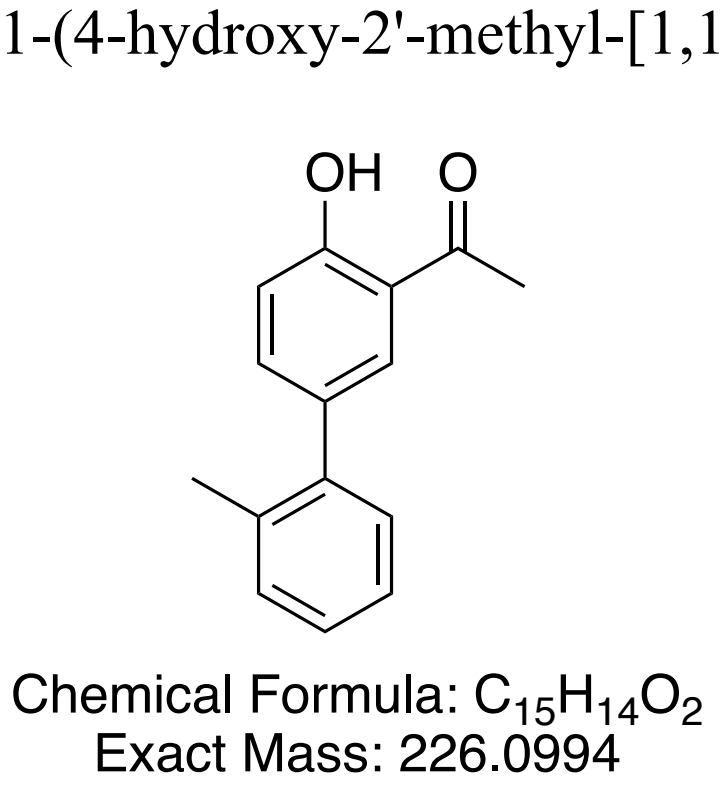

This compound was prepared according to the general procedure B. After purification by column chromatography (silica gel: PE/EA: 95/5), the title compound was isolated in 89% yield as a yellow oil.

^{1}H NMR (400 MHz, $CDCl_3$) δ = 12.23 (s, 1H), 7.65 (d, J = 2.2 Hz, 1H), 7.43 (dd, J = 8.5, 2.2 Hz, 1H), 7.28 – 7.24 (m, 2H), 7.24 – 7.22 (m, 1H), 7.21 – 7.17 (m, 1H), 7.01 (d, J = 8.5 Hz, 1H), 2.61 (s, 3H), 2.26 (s, 3H).

^{13}C NMR (101 MHz, $CDCl_3$) δ = 204.7, 161.5, 140.6, 137.5, 135.6, 132.9, 131.2, 130.6, 129.9, 127.7, 126.2, 119.5, 118.3, 26.9, 20.6.

HRMS(ESI) exact mass calculated for [M-H]+($C_{15}H_{13}O_2$+): m/z 225.0916; found: 225.0921.

1-(4-hydroxy-3',5'-dimethyl-[1,1'-biphenyl]-3-yl)ethan-1-one **(3y):**

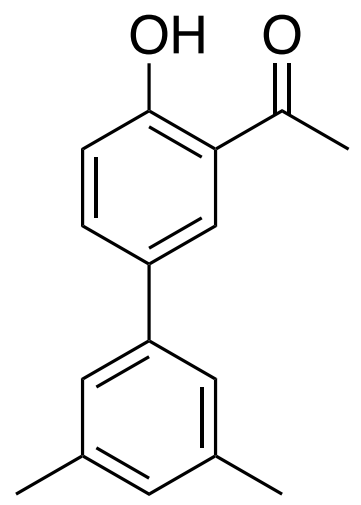

Chemical Formula: $C_{16}H_{16}O_2$
Exact Mass: 240.1150

This compound was prepared according to the general procedure B. After purification by column chromatography (silica gel: PE/EA: 95/5), the title compound was isolated in 80% yield as a white solid.

^{1}H NMR (400 MHz, $CDCl_3$) δ = 12.26 (s, 1H), 7.89 (d, J = 2.3 Hz, 1H), 7.70 (dd, J = 8.6, 2.3 Hz, 1H), 7.17 – 7.12 (m, 2H), 7.08 – 6.99 (m, 2H), 2.71 (s, 3H), 2.57 (s, 6H).

^{13}C NMR (101 MHz, $CDCl_3$) δ = 204.8, 161.8, 138.7, 135.6, 132.8, 129.1, 129.0, 124.8 (2C), 119.8, 118.9, 26.9, 21.5.

HRMS(ESI) exact mass calculated for [M-H]+($C_{16}H_{15}O_2$+): m/z 239.1072; found: 239.1076.

1-(4-hydroxy-3',5'-bis(trifluoromethyl)-[1,1'-biphenyl]-3-yl)ethan-1-one **(3z):**

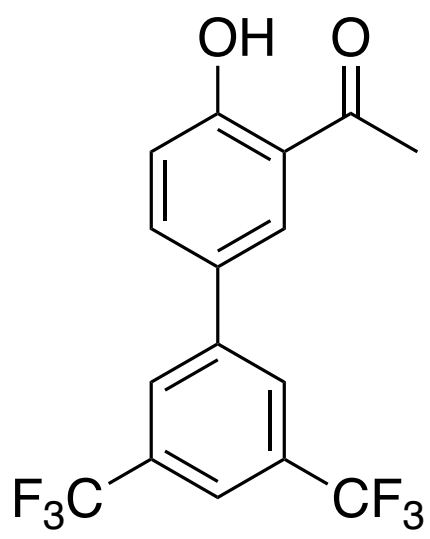

Chemical Formula: $C_{16}H_{10}F_6O_2$
Exact Mass: 348.0585

This compound was prepared according to the general procedure B. After purification by column chromatography (silica gel: PE/EA: 95/5), the title compound was isolated in 80% yield as a white solid.

^{1}H NMR (400 MHz, $CDCl_3$) δ = 12.40 (s, 1H), 7.93 (dd, J = 11.5, 2.0 Hz, 3H), 7.86 (s, 1H), 7.73 (dd, J = 8.7, 2.4 Hz, 1H), 7.14 (d, J = 8.7 Hz, 1H), 2.74 (s, 3H).

^{13}C NMR (101 MHz, $CDCl_3$) δ = 204.5, 163.1, 142.3, 135.3, 132.5 (q, $^{2}J_{C-F}$ = 33.3 Hz), 132.33, 129.43, 126.9, 123.4 (q, $^{1}J_{C-F}$ = 272.7 Hz), 121.0 (p, $^{3}J_{C-F}$ = 3.9 Hz), 120.1, 119.9, 27.0.

^{19}F NMR (376 MHz, $CDCl_3$) δ = −62.82.

HRMS(ESI) exact mass calculated for [M-H]+($C_{16}H_9F_6O_2+$): m/z 347.0507; found: 347.0505.

1-(2-hydroxy-5-(naphthalen-1-yl)phenyl)ethan-1-one **(3aa):**

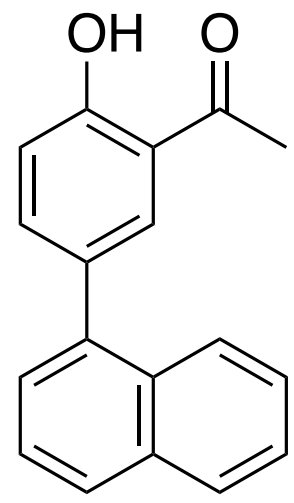

Chemical Formula: $C_{18}H_{14}O_2$
Exact Mass: 262.0994

This compound was prepared according to the general procedure B. After purification by column chromatography (silica gel: PE/EA: 95/5), the title compound was isolated in 72% yield as a yellow oil.

^{1}H NMR (400 MHz, $CDCl_3$) δ = 12.39 (s, 1H), 7.98 – 7.94 (m, 1H), 7.94 – 7.83 (m, 3H), 7.63 (dd, J = 8.5, 2.2 Hz, 1H), 7.59 – 7.40 (m, 4H), 7.14 (d, J = 8.6 Hz, 1H), 2.64 (s, 3H).

^{13}C NMR (101 MHz, $CDCl_3$) δ = 204.7, 161.8, 138.8, 138.2, 134.0, 132.0, 131.8, 131.6, 128.57, 128.0, 127.1, 126.4, 126.1, 125.6, 125.5, 119.7, 118.4, 26.8.

HRMS(ESI) exact mass calculated for [M+H]+($C_{18}H_{15}O_2+$): m/z 263.1072; found: 263.1081.

1-(5-(furan-3-yl)-2-hydroxyphenyl)ethan-1-one **(3ab):**

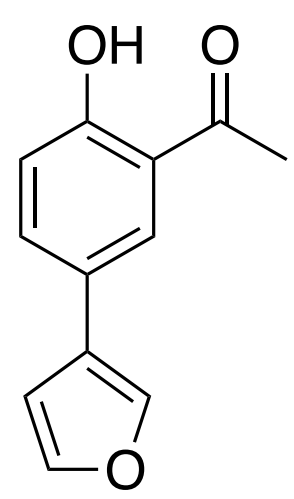

Chemical Formula: $C_{12}H_{10}O_3$
Exact Mass: 202.0630

This compound was prepared according to the general procedure B. After purification by column chromatography (silica gel: PE/EA: 95/5), the title compound was isolated in 89% yield as a yellow oil.

^{1}H NMR (400 MHz, $CDCl_3$) δ 12.24 (s, 1H), 7.78 (d, J = 2.3 Hz, 1H), 7.67 (dd, J = 1.6, 1.0 Hz, 1H), 7.58 (dd, J = 8.7, 2.2 Hz, 1H), 7.49 (t, J = 1.7 Hz, 1H), 7.00 (d, J = 8.6 Hz, 1H), 6.65 (dd, J = 1.9, 1.0 Hz, 1H), 2.67 (s, 3H).

^{13}C NMR (101 MHz, $CDCl_3$) δ 204.6, 161.6, 144.0, 138.1, 134.4, 127.7, 125.5, 123.8, 119.9, 119.1, 108.8, 26.8.

HRMS(ESI) exact mass calculated for [M+H]+($C_{12}H_{13}O_3^+$): m/z 203.0708; found: 203.0711.

1-(2-hydroxy-5-(thiophen-3-yl)phenyl)ethan-1-one **(3ac):**

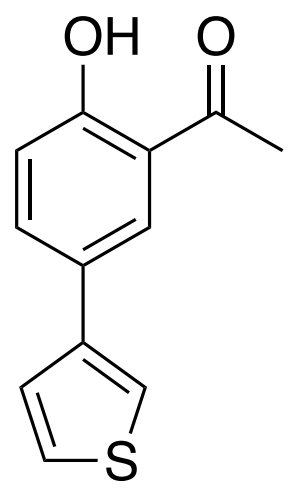

Chemical Formula: $C_{12}H_{10}O_2S$
Exact Mass: 218.0402

This compound was prepared according to the general procedure B. After purification by column chromatography (silica gel: PE/EA: 95/5), the title compound was isolated in 93% yield as a yellow oil.

^{1}H NMR (400 MHz, $CDCl_3$) δ 12.26 (s, 1H), 7.90 (d, J = 2.3 Hz, 1H), 7.70 (dd, J = 8.6, 2.3 Hz, 1H), 7.44 – 7.29 (m, 3H), 7.02 (d, J = 8.7 Hz, 1H), 2.68 (s, 3H).

^{13}C NMR (101 MHz, $CDCl_3$) δ 204.6, 161.7, 141.2, 134.9, 128.3, 127.4, 126.7, 126.13, 119.8, 119.0, 26.8.

HRMS(ESI) exact mass calculated for [M+H]+($C_{12}H_{13}SO_2^+$): m/z 219.0480; found: 219.0485.

1-(2-hydroxy-5-(pyridin-4-yl)phenyl)ethan-1-one[3] **(3ad):**

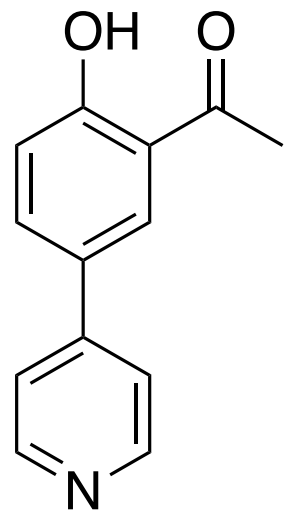

Chemical Formula: $C_{13}H_{11}NO_2$
Exact Mass: 213.0790

This compound was prepared according to the general procedure B. After purification by column chromatography (silica gel: PE/EA: 95/5), the title compound was isolated in 91% yield as a white solid.

^{1}H NMR (400 MHz, $CDCl_3$) δ = 12.38 (s, 1H), 8.67 – 8.61 (m, 2H), 7.97 (d, J = 2.3 Hz, 1H), 7.74 (dd, J = 8.7, 2.3 Hz, 1H), 7.48 – 7.40 (m, 2H), 7.09 (d, J = 8.7 Hz, 1H), 2.70 (s, 3H).

^{13}C NMR (101 MHz, $CDCl_3$) δ = 204.5 163.2, 150.4, 147.1, 134.9, 129.2, 129.1, 121.1, 120.0, 119.6, 26.8.

2-(1-iminoethyl)phenol**(1a)**[5]**:**

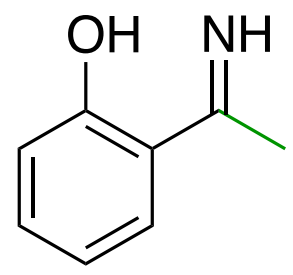

Chemical Formula: C_8H_9NO
Exact Mass: 135.0684

This compound was prepared from 1-(2-hydroxyphenyl)ethan-1-one (1.36 g, 10.0 mmol) according to the general procedure A. After purification by column chromatography (silica gel: DCM/MeOH: 95/5), the title compound was isolated in 91% yield as a yellow solid.

^{1}H NMR (400 MHz, DMSO-d_6). δ = δ 15.82 (s, 1H), 10.47 (s, 1H), 7.50 (dd, J = 8.1, 1.8 Hz, 1H), 7.25 (ddd, J = 8.7, 6.9, 1.8 Hz, 1H), 6.67 (dd, J = 8.6, 1.2 Hz, 1H), 6.56 (ddd, J = 8.2, 6.9, 1.2 Hz, 1H), 2.46 (s, 3H).

^{13}C NMR (101 MHz, DMSO-d_6) δ = 177.4, 169.7, 134.1, 129.9, 120.6, 116.5, 114.4, 21.8.

2-(1-iminoethyl)-6-methylphenol **(1b)**[6]**:**

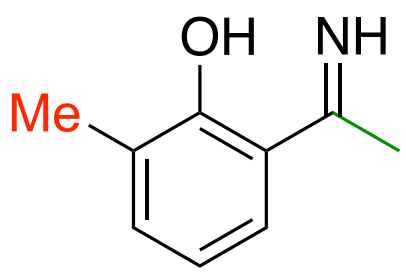

Chemical Formula: $C_9H_{11}NO$
Exact Mass: 149.0841

This compound was prepared from 1-(2-hydroxy-3-methylphenyl)ethan-1-one (1.5 g, 10.0 mmol) according to the general procedure C. After purification by column chromatography (silica gel: DCM/MeOH: 95/5), the title compound was isolated in 74% yield as a yellow solid.

^{1}H NMR (400 MHz, $CDCl_3$) δ 15.48 (s, 1H), 9.15 (s, 1H), 7.39 (dd, J = 8.1, 1.7 Hz, 1H), 7.25 (ddt, J = 7.3, 1.7, 0.8 Hz, 1H), 6.74 (dd, J = 8.0, 7.3 Hz, 1H), 2.51 (d, J = 1.4 Hz, 3H), 2.31 (s, 3H).

^{13}C NMR (101 MHz, $CDCl_3$) δ 178.5, 162.4, 134.23 127.5, 126.8, 117.8, 116.9, 26.3, 16.1.

2-(1-iminoethyl)-5-methylphenol **(1c)**[6]**:**

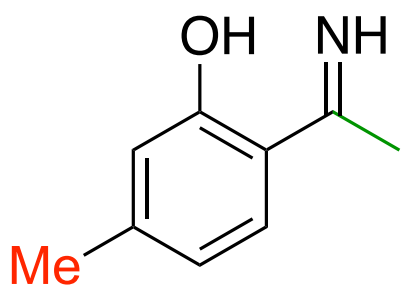

Chemical Formula: $C_9H_{11}NO$
Exact Mass: 149.0841

This compound was prepared from 1-(2-hydroxy-4-methylphenyl)ethan-1-one (1.5 g, 10.0 mmol) according to the general procedure C. After purification by column chromatography (silica gel: 95/5), the title compound was isolated in 67% yield as a yellow solid.

^{1}H NMR (400 MHz, $CDCl_3$) δ 15.07 (s, 1H), 8.91 (s, 1H), 7.29 (d, J = 8.1 Hz, 1H), 6.71 (s, 1H), 6.54 (d, J = 8.1 Hz, 1H), 2.38 (s, 3H), 2.25 (s, 3H).

^{13}C NMR (101 MHz, $CDCl_3$) δ 177.9, 164.2, 144.6, 129.0, 119.0, 118.9, 77.4, 26.0, 21.8.

2-(1-iminoethyl)-4-methylphenol **(1d)**[6]**:**

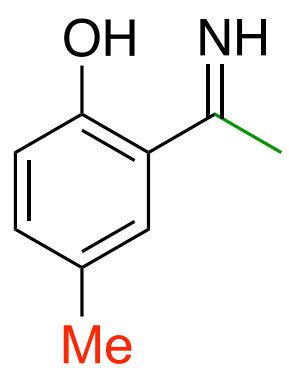

Chemical Formula: $C_9H_{11}NO$
Exact Mass: 149.0841

This compound was prepared from 1-(2-hydroxy-5-methylphenyl)ethan-1-one (1.5 g, 10.0 mmol) according to the general procedure C. After purification by column chromatography (silica gel: DCM/MeOH: 95/5), the title compound was isolated in 51% yield as a yellow solid.

^{1}H NMR (400 MHz, $CDCl_3$) δ 14.86 (s, 1H), 9.17 (s, 1H), 7.28 (d, J = 1.9 Hz, 1H), 7.19 – 7.12 (m, 1H), 6.88 (d, J = 8.4 Hz, 1H), 2.48 (d, J = 1.3 Hz, 3H), 2.29 (s, 3H).

^{13}C NMR (101 MHz, $CDCl_3$) δ 178.3, 161.2, 134.4, 129.1, 126.6, 118.5, 118.3, 26.4, 20.7.

2-(1-iminoethyl)-5-methoxyphenol **(1e)[6]:**

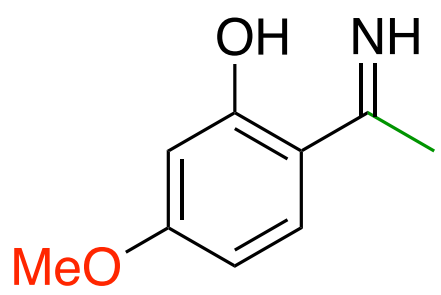

Chemical Formula: $C_9H_{11}NO_2$
Exact Mass: 165.0790

This compound was prepared from 1-(2-hydroxy-4-methoxyphenyl)ethan-1-one (1.66 g, 10.0 mmol) according to the general procedure C. After purification by column chromatography (silica gel: DCM/MeOH: 95/5), the title compound was isolated in 63% yield as a yellow solid.

^{1}H NMR (400 MHz, $CDCl_3$) δ 15.43 (s, 1H), 8.10 (s, 1H), 7.32 (d, J = 9.0 Hz, 1H), 6.34 (d, J = 2.5 Hz, 1H), 6.26 (dd, J = 9.0, 2.6 Hz, 1H), 3.80 (s, 3H), 2.44 (d, J = 1.1 Hz, 3H).

^{13}C NMR (101 MHz, $CDCl_3$) δ 175.3, 172.1, 165.3, 130.5, 111.3, 106.5, 102.4, 55.4, 24.0.

2-(1-iminoethyl)-4-methoxyphenol **(1f)[6]:**

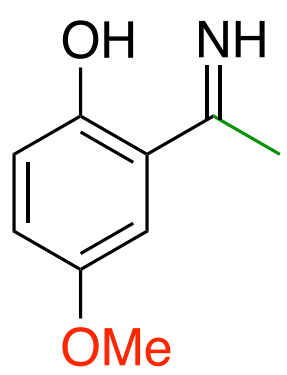

Chemical Formula: $C_9H_{11}NO_2$
Exact Mass: 165.0790

This compound was prepared from 1-(2-hydroxy-5-methoxyphenyl)ethan-1-one (1.66 g, 10.0 mmol) according to the general procedure C. After purification by column chromatography (silica gel: DCM/MeOH: 95/5), the title compound was isolated in 71% yield as a yellow solid.

^{1}H NMR (400 MHz, $CDCl_3$) δ 14.52 (s, 1H), 9.26 (s, 1H), 6.99 – 6.93 (m, 2H), 6.91 (dd, *J* = 8.6 Hz, 1H), 3.81 (s, 3H), 2.47 (d, *J* = 1.4 Hz, 3H).

^{13}C NMR (101 MHz, $CDCl_3$) δ 178.1, 157.5, 151.2, 120.3, 119.0, 118.6, 113.4, 56.2, 26.6.

4-fluoro-2-(1-iminoethyl)phenol **(1g)[5]:**

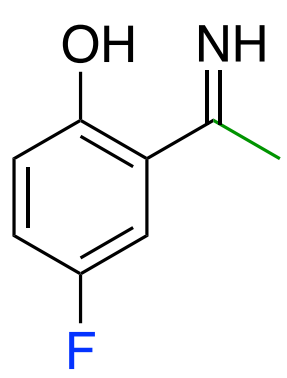

Chemical Formula: C_8H_8FNO
Exact Mass: 153.0590

This compound was prepared from 1-(5-fluoro-2-hydroxyphenyl)ethan-1-one according to the general procedure C. After purification by column chromatography (silica gel: DCM/MeOH: 95/5), the title compound was isolated in 85% yield as a yellow solid.

^{1}H NMR (400 MHz, $CDCl_3$) δ 14.70 (s, 1H), 9.26 (s, 1H), 7.10 (dd, J = 9.5, 3.1 Hz, 1H), 7.00 (ddd, J = 9.1, 7.9, 3.1 Hz, 1H), 6.85 (dd, J = 9.1, 4.8 Hz, 1H), 2.39 (d, J = 1.4 Hz, 3H).

^{13}C NMR (101 MHz, $CDCl_3$) δ 177.6, 159.4, 154.6 (d, $^{1}J_{C-F}$ = 236.3 Hz), 120.6 (d, $^{2}J_{C-F}$ = 23.2 Hz), 120.50, 119.43 (d, $^{3}J_{C-F}$ = 7.5 Hz), 118.5, 114.3 (d, $^{2}J_{C-F}$ = 24.2 Hz), 26.4.

^{19}F NMR (376 MHz, $CDCl_3$) δ -126.28.

4-chloro-2-(1-iminoethyl)phenol **(1h):**

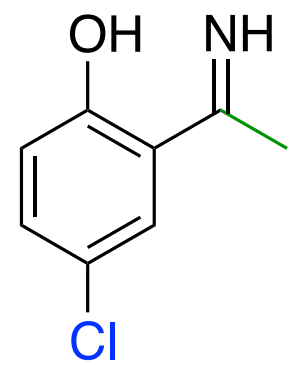

Chemical Formula: C_8H_8ClNO
Exact Mass: 169.0294

This compound was prepared from 1-(5-chloro-2-hydroxyphenyl)ethan-1-one according to the general procedure C. After purification by column chromatography (silica gel: DCM/MeOH: 95/5), the title compound was isolated in 83% yield as a yellow solid.

^{1}H NMR (400 MHz, $CDCl_3$) δ 15.12 (s, 1H), 9.26 (s, 1H), 7.45 (d, *J* = 2.6 Hz, 1H), 7.28 (dd, *J* = 8.8 Hz, 1H), 6.92 (d, *J* = 8.9 Hz, 1H), 2.47 (d, *J* = 1.4 Hz, 3H).

^{13}C NMR (101 MHz, $CDCl_3$) δ 177.6, 162.4, 133.4, 128.5, 120.3, 26.1.

HRMS(ESI) exact mass calculated for [M-H]+(C_8H_7ClNO+): m/z 168.0216; found: 168.0222.

4-bromo-2-(1-iminoethyl)phenol **(1i)**[6]**:**

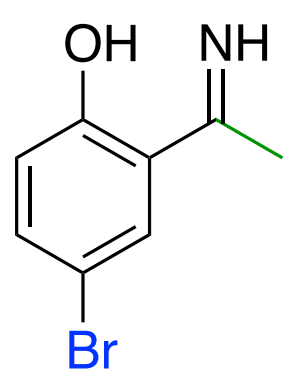

Chemical Formula: C_8H_8BrNO
Exact Mass: 212.9789

This compound was prepared from 1-(5-bromo-2-hydroxyphenyl)ethan-1-one according to the general procedure C. After purification by column chromatography (silica gel: DCM/MeOH: 95/5), the title compound was isolated in 73% yield as a yellow solid.

^{1}H NMR (400 MHz, $CDCl_3$) δ 15.17 (s, 1H), 9.24 (s, 1H), 7.59 (d, *J* = 2.5 Hz, 1H), 7.40 (dd, *J* = 8.9, 2.5 Hz, 1H), 6.87 (d, *J* = 8.9 Hz, 1H), 2.47 (d, *J* = 1.4 Hz, 3H).

^{13}C NMR (101 MHz, $CDCl_3$) δ 177.5, 162.9, 136.2, 131.5, 120.8, 120.1, 109.1, 26.1.

2-(1-iminoethyl)-4-(trifluoromethyl)phenol **(1j):**

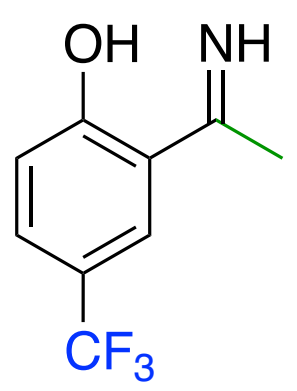

Chemical Formula: $C_9H_8F_3NO$
Exact Mass: 203.0558

This compound was prepared from 1-(2-hydroxy-5-(trifluoromethyl)phenyl)ethan-1-one according to the general procedure C. After purification by column chromatography (silica gel: DCM/MeOH: 95/5), the title compound was isolated in 74% yield as a yellow solid.

^{1}H NMR (400 MHz, $CDCl_3$) δ 15.78 (s, 1H), 9.17 (s, 1H), 7.74 (dd, J = 2.3, 1.1 Hz, 1H), 7.59 – 7.51 (m, 1H), 7.02 (dd, J = 8.9, 0.9 Hz, 1H), 2.54 (d, J = 1.3 Hz, 3H).

^{13}C NMR (101 MHz, $CDCl_3$) δ 177.9, 167.6, 130.3, 126.8, 120.0, 117.6, 25.6.

^{19}F NMR (376 MHz, $CDCl_3$) δ -61.45.

HRMS(ESI) exact mass calculated for [M-H]+($C_9H_7F_3NO^+$): m/z 202.0480; found: 202.0483.

4-hydroxy-3-(1-iminoethyl)benzonitrile **(1k):**

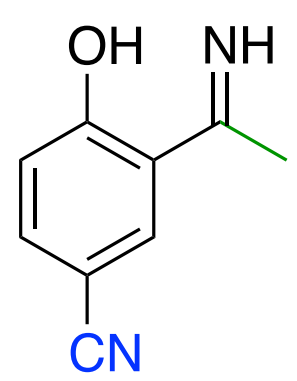

Chemical Formula: $C_9H_8N_2O$
Exact Mass: 160.0637

This compound was prepared from 3-acetyl-4-hydroxybenzonitrile according to the general procedure C. After purification by column chromatography (silica gel: DCM/MeOH: 95/5), the title compound was isolated in 91% yield as a yellow solid.

^{1}H NMR (400 MHz, DMSO-d_6). δ 14.84 (s, 1H), 10.83 (s, 1H), 8.02 (q, J = 2.1 Hz, 1H), 7.41 (dt, J = 9.2, 1.8 Hz, 1H), 6.55 (dd, J = 9.1, 1.2 Hz, 1H), 2.56 (s, 3H).

^{13}C NMR (101 MHz, DMSO-d_6) δ 177.9, 138.2, 136.0, 125.2, 120.3, 114.5 (2C), 92.2, 19.7.

HRMS(ESI) exact mass calculated for [M+H]+($C_9H_9N_2O^+$): m/z 161.0715; found: 161.0710.

2-(1-iminoethyl)-4-nitrophenol **(1l)**[6]**:**

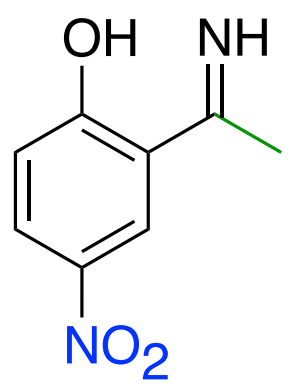

Chemical Formula: $C_8H_8N_2O_3$
Exact Mass: 180.0535

This compound was prepared from 1-(2-hydroxy-5-nitrophenyl)ethan-1-one according to the general procedure C. After purification by column chromatography (silica gel: DCM/MeOH: 95/5), the title compound was isolated in 82% yield as a yellow solid.

^{1}H NMR (400 MHz, $CDCl_3$). δ = 14.39 (s, 1H), 8.49 (d, J = 3.1 Hz, 1H), 7.98 (dd, J = 9.7, 3.1 Hz, 1H), 6.52 (d, J = 9.8 Hz, 1H), 2.64 (s, 3H).

^{13}C NMR (101 MHz, $CDCl_3$) δ = 180.0, 179.0, 132.0, 130.3, 129.2, 124.6, 112.5, 19.7.

methyl 4-hydroxy-3-(1-iminoethyl)benzoate **(1m):**

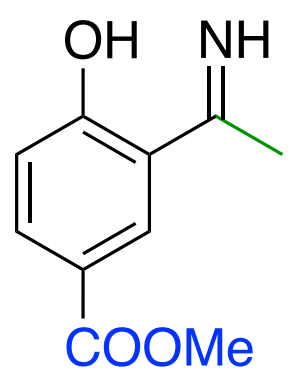

Chemical Formula: $C_{10}H_{11}NO_3$
Exact Mass: 193.0739

This compound was prepared from methyl 3-acetyl-4-hydroxybenzoate according to the general procedure C. After purification by column chromatography (silica gel: DCM/MeOH: 95/5), the title compound was isolated in 62% yield as a yellow solid.

^{1}H NMR (400 MHz, $CDCl_3$) δ 9.01 (s, 1H), 8.26 (d, *J* = 2.2 Hz, 1H), 8.00 (dd, *J* = 8.8, 2.2 Hz, 1H), 6.95 (d, *J* = 8.8 Hz, 1H), 3.91 (s, 3H), 2.59 (d, *J* = 1.3 Hz, 3H).

^{13}C NMR (101 MHz, $CDCl_3$) δ 178.4, 170.4, 166.8, 135.0, 132.4, 120.0, 118.6, 117.2, 52.0, 25.2.

HRMS(ESI) exact mass calculated for [M-H]+($C_{10}H_{10}NO_3$+): m/z 192.0661; found: 192.0666.

3-(1-iminoethyl)-[1,1'-biphenyl]-4-ol **(1n):**

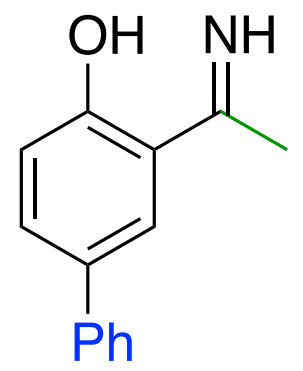

Chemical Formula: $C_{14}H_{13}NO$
Exact Mass: 211.0997

This compound was prepared from 1-(2-hydroxyphenyl)ethan-1-one according to the general procedure C. After purification by column chromatography (silica gel: DCM/MeOH: 95/5), the title compound was isolated in 76% yield as a yellow solid.

^{1}H NMR (400 MHz, DMSO-d_6). δ = 15.77 (s, 1H), 10.52 (s, 1H), 7.74 (d, J = 2.5 Hz, 1H), 7.65 – 7.56 (m, 3H), 7.45 – 7.35 (m, 2H), 7.30 – 7.21 (m, 1H), 6.73 (d, J = 8.9 Hz, 1H), 2.58 (s, 3H).

^{13}C NMR (101 MHz, DMSO-d_6) δ = 177.6, 171.3, 140.2, 133.2, 128.7, 127.8, 126.0, 125.7, 125.4, 122.1, 115.7, 21.3.

HRMS(ESI) exact mass calculated for [M+H]+($C_{14}H_{14}NO^+$): m/z 212.1075; found: 212.1063.

2-(1-iminoethyl)-4,6-dimethylphenol **(1o):**

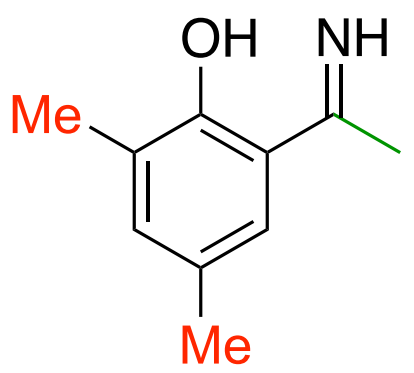

Chemical Formula: $C_{10}H_{13}NO$
Exact Mass: 163.0997

This compound was prepared from 1-(2-hydroxy-3,5-dimethylphenyl)ethan-1-one according to the general procedure C. After purification by column chromatography (silica gel: DCM/MeOH: 95/5), the title compound was isolated in 77% yield as a yellow solid.

^{1}H NMR (400 MHz, DMSO-d_6).δ = 15.81 (s, 1H), 10.32 (s, 1H), 7.14 (d, J = 2.3 Hz, 1H), 7.01 (d, J = 2.2 Hz, 1H), 2.43 (d, J = 1.0 Hz, 3H), 2.16 (s, 3H), 2.07 (s, 3H).

^{13}C NMR (101 MHz, DMSO-d_6) δ= 177.0, 165.4, 135.3, 127.6, 126.4, 122.2, 115.3, 22.4, 20.2, 16.0.

HRMS(ESI) exact mass calculated for $C_{10}H_{14}NO$ $[M+H]^+$: 164.1070, found: 164.1074.

6-(1-iminoethyl)-2,3-dimethylphenol **(1p)**:

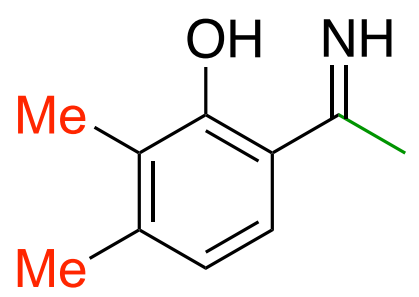

Chemical Formula: $C_{10}H_{13}NO$
Exact Mass: 163.0997

This compound was prepared from 1-(2-hydroxy-3,4-dimethylphenyl)ethan-1-one according to the general procedure C. After purification by column chromatography (silica gel: DCM/MeOH: 95/5), the title compound was isolated in 88% yield as a yellow solid.

^{1}H NMR (400 MHz, $CDCl_3$) δ = 9.00 (s, 1H), 7.25 (d, J = 8.2 Hz, 1H), 6.62 (d, J = 8.1 Hz, 1H), 2.47 (s, 3H), 2.29 (s, 3H), 2.20 (s, 3H).

^{13}C NMR (101 MHz, $CDCl_3$) δ = 178.2, 162.1, 142.5, 125.9, 125.5, 119.1, 115.9, 26.3, 20.7, 11.5.

HRMS(ESI) exact mass calculated for [M+H]+($C_{10}H_{14}NO^+$): m/z 164.1070; found: 164.1074.

2-(1-iminoethyl)-3,5-dimethylphenol **(1q)**:

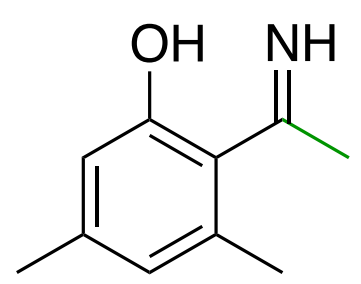

Chemical Formula: $C_{10}H_{13}NO$
Exact Mass: 163.0997

This compound was prepared from 1-(4-hydroxy-4'-methoxy-[1,1'-biphenyl]-3-yl)ethan-1-one according to the general procedure C. After purification by column chromatography (silica gel: DCM/MeOH: 95/5), the title compound was isolated in 89% yield as a yellow solid.

^{1}H NMR (400 MHz, $CDCl_3$) δ 9.17 (s, 1H), 7.14 (s, 1H), 7.07 (s, 1H), 2.48 (s, 3H), 2.26 (s, 3H), 2.26 (s, 3H).

^{13}C NMR (101 MHz, $CDCl_3$) δ 178.5, 159.9, 135.5, 127.2, 126.6, 125.8, 117.5, 26.4, 20.7, 16.1.

HRMS(ESI) exact mass calculated for [M+H]+($C_{10}H_{14}NO^+$): m/z 164.1075; found: 164.1069.

2-(1-iminoethyl)-3,5,6-trimethylphenol **(1r)**:

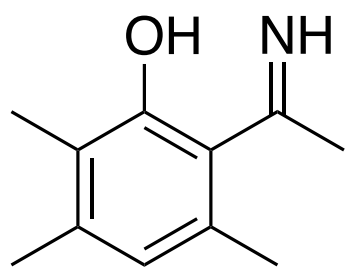

Chemical Formula: $C_{11}H_{15}NO$
Exact Mass: 177.1154

This compound was prepared from 1-(2-hydroxy-3,4-dimethylphenyl)ethan-1-one according to the general procedure C. After purification by column chromatography (silica gel: DCM/MeOH: 95/5), the title compound was isolated in 67% yield as a yellow solid.

^{1}H NMR (400 MHz, $CDCl_3$) δ 6.46 (s, 1H), 2.54 (s, 3H), 2.44 (s, 3H), 2.23 (s, 3H), 2.16 (s, 3H).

^{13}C NMR (101 MHz, $CDCl_3$) δ 180.1, 161.9, 140.7, 134.7, 123.4, 123.3, 117.1, 31.8, 24.4, 20.3, 11.8.

HRMS(ESI) exact mass calculated for [M+H]+($C_{11}H_{16}NO^+$): m/z 178.1232; found: 178.1225.

2-(1-iminopropyl)phenol **(1s)**[6]**:**

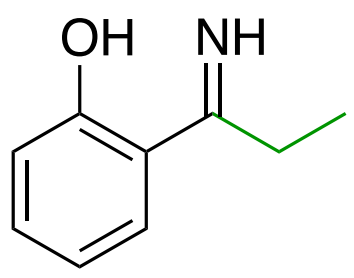

Chemical Formula: $C_9H_{11}NO$
Exact Mass: 149.0841

This compound was prepared from 1-(2-hydroxyphenyl)propan-1-one according to the general procedure C. After purification by column chromatography (silica gel: DCM/MeOH: 95/5), the title compound was isolated in 83% yield as a yellow solid.

^{1}H NMR (400 MHz, $CDCl_3$) δ 15.41 (s, 1H), 9.36 (s, 1H), 7.55 (dd, J = 8.0, 1.7 Hz, 1H), 7.33 (ddd, J = 8.6, 7.1, 1.7 Hz, 1H), 6.98 (dd, J = 8.4, 1.2 Hz, 1H), 6.81 (ddd, J = 8.2, 7.1, 1.2 Hz, 1H), 2.82 (qd, J = 7.4, 1.4 Hz, 2H), 1.26 (t, J = 7.4 Hz, 3H).

^{13}C NMR (101 MHz, $CDCl_3$) δ 182.1, 163.7, 133.2, 128.2, 118.8, 118.4, 117.6, 29.1, 9.4.

2-(imino(phenyl)methyl)phenol **(1t)**[7]**:**

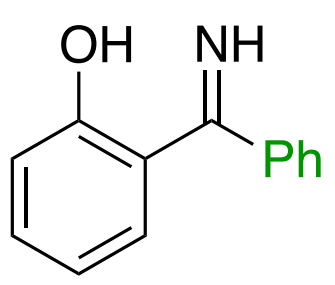

Chemical Formula: $C_{13}H_{11}NO$
Exact Mass: 197.0841

This compound was prepared from (2-hydroxyphenyl)(phenyl)methanone (1.36 g, 10.0 mmol) according to the general procedure C. After purification by column chromatography

(silica gel: DCM/MeOH: 95/5), the title compound was isolated in 92% yield as a yellow solid.

^{1}H NMR (400 MHz, DMSO-d$_6$). δ = 15.57 (s, 1H), 10.75 (s, 1H), 7.63 – 7.43 (m, 5H), 7.32 (ddd, J = 8.6, 7.0, 1.8 Hz, 1H), 7.05 (dd, J = 8.1, 1.7 Hz, 1H), 6.83 (dd, J = 8.5, 1.2 Hz, 1H), 6.64 – 6.57 (m, 1H).

^{13}C NMR (101 MHz, DMSO-d$_6$) δ = 178.4, 168.2, 136.3, 134.0, 131.8, 130.1, 128.5, 127.8, 119.9, 116.4, 115.7.

3-(1-iminoethyl)-4'-methyl-[1,1'-biphenyl]-4-ol **(1u):**

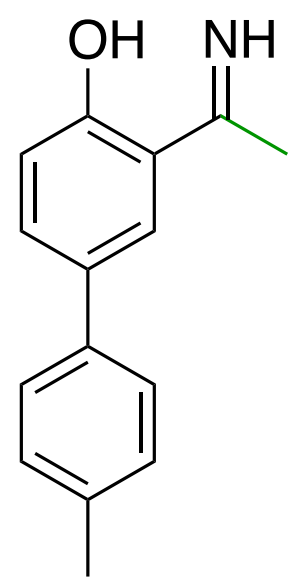

Chemical Formula: $C_{15}H_{15}NO$
Exact Mass: 225.1154

This compound was prepared from 1-(4-hydroxy-4'-methyl-[1,1'-biphenyl]-3-yl)ethan-1-one (1.36 g, 10.0 mmol) according to the general procedure C. After purification by column chromatography (silica gel: DCM/MeOH: 95/5), the title compound was isolated in 81% yield as a yellow solid.

^{1}H NMR (400 MHz, DMSO-d$_6$). δ = 15.78 (s, 1H), 10.49 (s, 1H), 7.71 (d, J = 2.5 Hz, 1H), 7.59 – 7.56 (m, 1H), 7.53 – 7.49 (m, 2H), 7.22 – 7.20 (m, 2H), 6.73 (d, J = 8.9 Hz, 1H), 2.58 (s, 3H), 2.32 (s, 3H).

^{13}C NMR (101 MHz, DMSO-d$_6$) δ = 178.0, 171.2, 137.8, 135.6, 133.4, 129.8, 127.8, 126.1, 126.0, 122.3, 116.3, 21.9, 21.1.

HRMS(ESI) exact mass calculated for [M+H]+($C_{15}H_{16}NO$+): m/z 226.1232; found: 226.1219.

3-(1-iminoethyl)-4'-methoxy-[1,1'-biphenyl]-4-ol **(1v)**:

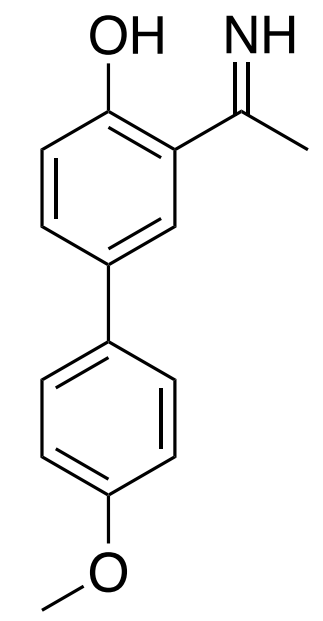

Chemical Formula: $C_{15}H_{15}NO_2$
Exact Mass: 241.1103

This compound was prepared from 1-(4-hydroxy-4'-methoxy-[1,1'-biphenyl]-3-yl)ethan-1-one according to the general procedure C. After purification by column chromatography (silica gel: DCM/MeOH: 95/5), the title compound was isolated in 75% yield as a yellow solid.

^{1}H NMR (400 MHz, DMSO-d_6) δ 15.76 (s, 1H), 7.66 (d, J = 2.4 Hz,1H), 7.58 – 7.49 (m, 3H), 7.06 – 6.93 (m, 2H), 6.72 (d, J = 8.9 Hz, 1H), 3.78 (s, 3H), 2.56 (s, 3H).

^{13}C NMR (101 MHz, DMSO-d_6) δ 177.5, 170.1, 157.9, 132.8, 132.7, 127.0, 126.8, 125.7, 121.6, 115.9, 114.2, 55.1, 21.6.

HRMS(ESI) exact mass calculated for [M+H]+($C_{15}H_{16}NO_2$+): m/z 242.1176; found: 242.1184.

4'-fluoro-3-(1-iminoethyl)-[1,1'-biphenyl]-4-ol **(1w)**:

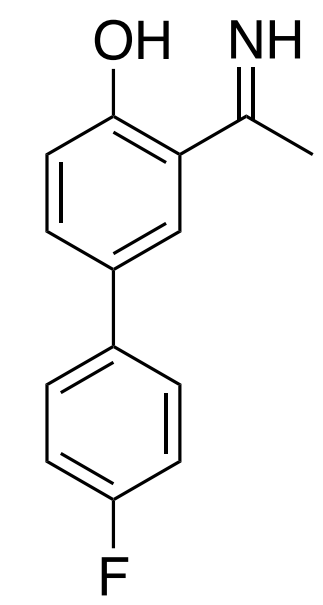

Chemical Formula: $C_{14}H_{12}FNO$
Exact Mass: 229.0903

This compound was prepared from 1-(4'-fluoro-4-hydroxy-[1,1'-biphenyl]-3-yl)ethan-1-oneaccording to the general procedure C. After purification by column chromatography (silica gel: DCM/MeOH: 95/5), the title compound was isolated in 82% yield as a yellow solid.

^{1}H NMR (400 MHz, DMSO-d_6) δ 15.76 (s, 1H), 10.53 (s, 1H), 7.71 (d, J = 2.5 Hz, 1H), 7.69 – 7.59 (m, 2H), 7.56 (dd, J = 8.9, 2.5 Hz, 1H), 7.27 – 7.17 (m, 2H), 6.72 (d, J = 8.9 Hz, 1H), 2.58 (s, 3H).

^{13}C NMR (101 MHz, DMSO-d_6) δ = 177.6, 171.3, 162.19 (d, $^1J_{C\text{-}F}$ = 240.38 Hz), 136.7 (d, $^4J_{C\text{-}F}$ = 3.1 Hz), 133.1, 127.8, 127.6 (d, $^3J_{C\text{-}F}$ = 8.08 Hz), 124.4, 122.1, 115.7, 115.4 (d, $^2J_{C\text{-}F}$ = 21.21 Hz), 21.3.

^{19}F NMR (376 MHz, DMSO-d_6) δ = -117.43.

HRMS(ESI) exact mass calculated for $[M+NH_4]^+$($C_{14}H_{16}FN_2O^+$): m/z 247.1247; found: 247.1300.

3-(1-iminoethyl)-2'-methyl-[1,1'-biphenyl]-4-ol **(1x)**:

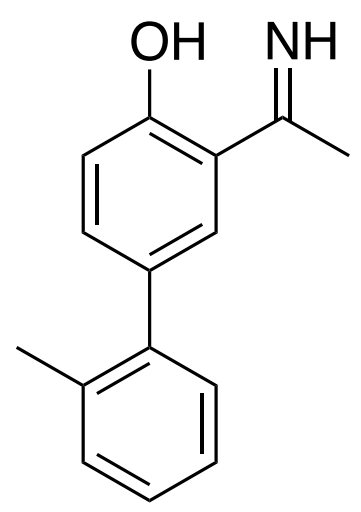

Chemical Formula: $C_{15}H_{15}NO$
Exact Mass: 225.1154

This compound was prepared from 1-(4-hydroxy-2'-methyl-[1,1'-biphenyl]-3-yl)ethan-1-one according to the general procedure C. After purification by column chromatography (silica gel: DCM/MeOH: 95/5), the title compound was isolated in 77% yield as a yellow solid.

^1H NMR (400 MHz, $CDCl_3$) δ = 9.26 (s, 1H), 7.44 (d, J = 2.2 Hz, 1H), 7.36 – 7.18 (m, 5H), 7.02 (d, J = 8.5 Hz, 1H), 2.52 (s, 3H), 2.31 (s, 3H).

^{13}C NMR (101 MHz, $CDCl_3$) δ =178.5, 162.7, 141.5, 135.7, 134.5 (2C), 131.4, 130.5, 130.0, 129.7, 127.3, 126.0, 118.3, 26.3, 20.7.

HRMS(ESI) exact mass calculated for $[M+H]^+$($C_{15}H_{16}NO^+$): m/z 226.1226; found: 226.1235.

3-(1-iminoethyl)-3',5'-dimethyl-[1,1'-biphenyl]-4-ol **(1y)**:

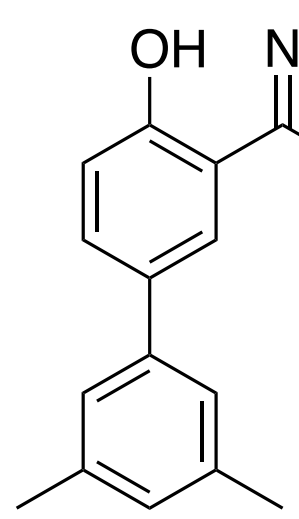

Chemical Formula: $C_{16}H_{17}NO$
Exact Mass: 239.1310

This compound was prepared from 1-(4-hydroxy-3',5'-dimethyl-[1,1'-biphenyl]-3-yl)ethan-1-one according to the general procedure C. After purification by column chromatography (silica gel: DCM/MeOH: 95/5), the title compound was isolated in 82% yield as a yellow solid.

^{1}H NMR (400 MHz, $CDCl_3$) δ = 12.26 (s, 1H), 7.89 (d, J = 2.3 Hz, 1H), 7.70 (dd, J = 8.6, 2.3 Hz, 1H), 7.17 – 7.12 (m, 2H), 7.08 – 6.99 (m, 2H), 2.71 (s, 3H), 2.57 (s, 6H).

^{13}C NMR (101 MHz, $CDCl_3$) δ = 161.8, 138.7, 135.6, 132.8, 129.1, 129.0, 124.8, 119.8, 118.9, 26.9, 21.5.

HRMS(ESI) exact mass calculated for [M+H]+($C_{16}H_{18}NO^+$): m/z 240.1383; found: 240.1390.

3-(1-iminoethyl)-3',5'-bis(trifluoromethyl)-[1,1'-biphenyl]-4-ol **(1z)**:

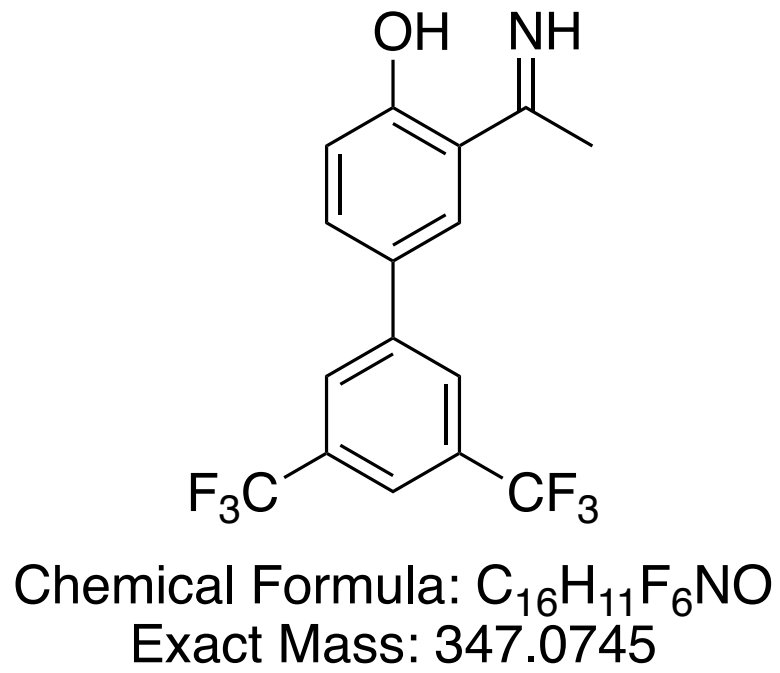

This compound was prepared from 1-(4-hydroxy-3',5'-bis(trifluoromethyl)-[1,1'-biphenyl]-3-yl)ethan-1-one according to the general procedure C. After purification by column chromatography (silica gel: DCM/MeOH: 95/5), the title compound was isolated in 81% yield as a yellow solid.

^{1}H NMR (400 MHz, $CDCl_3$) δ = 9.23 (s, 1H), 7.94 (d, J = 1.6 Hz, 2H), 7.81 (tt, J = 1.6, 0.8 Hz, 1H), 7.68 (d, J = 2.4 Hz, 1H), 7.60 (dd, J = 8.7, 2.4 Hz, 1H), 7.10 (d, J = 8.7 Hz, 1H), 2.60 (d, J = 1.1 Hz, 3H).

^{13}C NMR (101 MHz, $CDCl_3$) δ = 178.1, 165.4, 143.0, 132.6, 132.50 (q, J = 33.3 Hz), 127.9, 127.6, 126.7, 123.6 (q, $^{1}J_{C-F}$ = 274.7 Hz), 120.3, 119.5 (q, J = 5,0 Hz), 118.8, 26.0.

^{19}F NMR (376 MHz, $CDCl_3$) δ = -63.2.

HRMS(ESI) exact mass calculated for [M+H]+($C_{16}H_{12}F_6NO^+$): m/z 348.0818; found: 348.0828.

2-(1-iminoethyl)-4-(naphthalen-1-yl)phenol **(1aa)**:

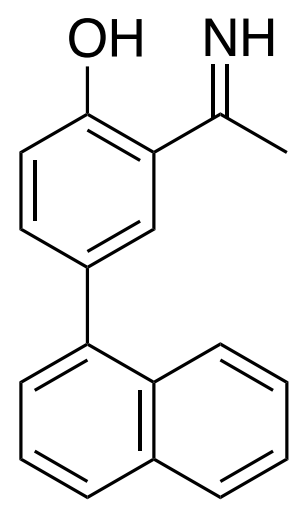

Chemical Formula: $C_{18}H_{15}NO$
Exact Mass: 261.1154

This compound was prepared from 1-(2-hydroxy-5-(naphthalen-1-yl)phenyl)ethan-1-one according to the general procedure C. After purification by column chromatography (silica gel: DCM/MeOH: 95/5), the title compound was isolated in 86% yield as a yellow solid.

^{1}H NMR (400 MHz, DMSO-d_6) δ 9.53 (s, 1H), 7.99 – 7.82 (m, 3H), 7.61 (d, J = 2.2 Hz, 1H), 7.58 – 7.38 (m, 5H), 7.14 (d, J = 8.5 Hz, 1H), 2.53 (s, 3H).

^{13}C NMR (101 MHz, DMSO-d_6) δ 177.4, 170.9, 139.5, 136.2, 133.6, 131.2, 130.8, 128.3, 127.0, 126.7, 126.2, 125.5, 121.3, 115.7, 21.4.

HRMS(ESI) exact mass calculated for [M+H]+($C_{18}H_{16}NO$+): m/z 262.1226; found: 262.1234.

2-(imino(phenyl)methyl)-5-methoxyphenol **(1ab):**

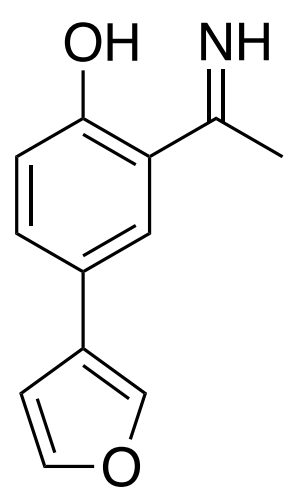

Chemical Formula: $C_{12}H_{11}NO_2$
Exact Mass: 201.0790

This compound was prepared from 4-(furan-3-yl)-2-(1-iminoethyl)phenol according to the general procedure C. After purification by column chromatography (silica gel: DCM/MeOH: 95/5), the title compound was isolated in 81% yield as a yellow solid.

^{1}H NMR (400 MHz, DMSO-d_6) δ = δ 15.70 (s, 1H), 10.46 (s, 1H), 8.02 (dd, J = 1.6, 0.9 Hz, 1H), 7.70 – 7.62 (m, 2H), 7.51 (dd, J = 8.9, 2.4 Hz, 1H), 6.92 (dd, J = 1.9, 0.9 Hz, 1H), 6.67 (d, J = 8.8 Hz, 1H), 2.54 (s, 3H).

^{13}C NMR (101 MHz, DMSO-d_6) δ = 177.2, 170.7, 143.8, 137.6, 132.5, 126.3, 125.8, 121.88, 117.6, 115.6, 108.6, 21.4.

HRMS(ESI) exact mass calculated for [M+H]+($C_{12}H_{12}NO_2$+): m/z 202.00863; found: 202.0869.

2-(1-iminoethyl)-4-(thiophen-3-yl)phenol **(1ac)**:

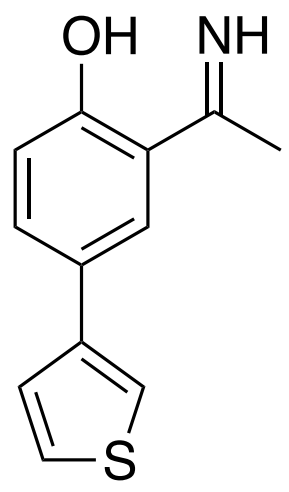

Chemical Formula: $C_{12}H_{11}NOS$
Exact Mass: 217.0561

This compound was prepared from 1-(2-hydroxy-5-(thiophen-3-yl)phenyl)ethan-1-one according to the general procedure C. After purification by column chromatography (silica gel: DCM/MeOH: 95/5), the title compound was isolated in 83% yield as a yellow solid.

^{1}H NMR (400 MHz, $CDCl_3$) δ = 9.30 (s, 1H), 7.69 (s, 1H), 7.60 (s, 1H), 7.59-7.57(m, 1H), 7.39-7.32 (m, 2H),7.01 (m, 1H), 2.56 (s, 3H).

^{13}C NMR (101 MHz, $CDCl_3$) δ 178.3, 163.5, 142.1, 132.2, 127.0, 126.4, 126.3, 125.9, 119.3, 119.0, 118.5, 26.1.

HRMS(ESI) exact mass calculated for [M+H]+($C_{12}H_{12}NOS$+): m/z 218.0641; found: 218.0634.

2-(1-iminoethyl)-4-(pyridin-4-yl)phenol**(1ad)**:

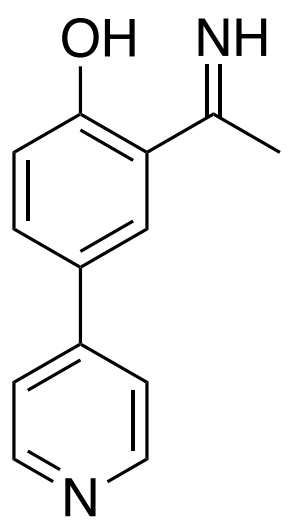

Chemical Formula: $C_{13}H_{12}N_2O$
Exact Mass: 212.0950

This compound was prepared from 1-(4-hydroxy-4'-methoxy-[1,1'-biphenyl]-3-yl)ethan-1-one according to the general procedure C. After purification by column chromatography (silica gel: DCM/MeOH: 95/5), the title compound was isolated in 79% yield as a yellow solid.

^{1}H NMR (400 MHz, DMSO-d_6) δ 15.58 (s, 1H), 8.55 – 8.49 (m, 2H), 7.93 (d, J = 2.5 Hz, 1H), 7.72 (dd, J = 9.1, 2.6 Hz, 1H), 7.69 – 7.63 (m, 2H), 6.70 (d, J = 9.1 Hz, 1H), 2.62 (s, 3H).

^{13}C NMR (101 MHz, DMSO-d_6) δ 177.7, 177.6, 149.9, 133.0, 129.0, 123.5, 120.7, 119.7, 38.9, 20.6.

HRMS(ESI) exact mass calculated for [M+Na]+($C_{13}H_{12}N_2NaO$+): m/z 235.0847; found: 235.0851.

II. 1. Synthesis of α- Substituted Primary Amines through Asymmetric Transfer Hydrogenation

Standard Method: A dried Schlenk tube equipped with a magnetic stirring bar was charged with imine (0.30 mmol, 1 equiv.), ammonium formate (0.6 mmol, 37.8 mg, 2 equiv.) and (*R,R*)-A (0.003 mmol, 2.3 mg, 1 mol%). The tube was purged 3 times with argon atmosphere. The solvent of DCM/ACN (2.7 mL/0.27 mL) was added by syringe and the reaction mixture was allowed to stir for 2 h under argon atmosphere at 50 °C. The mixture was directly loaded onto a silica column (1% MeOH in DCM) for the purification by column chromatography to afford the amine.

(*R*)-2-(1-aminoethyl)phenol **(2a)**[5]**:**

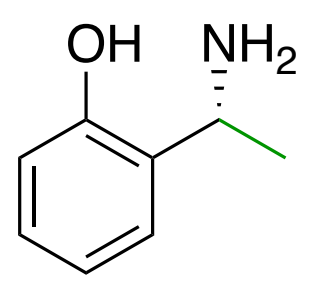

Chemical Formula: $C_8H_{11}NO$
Exact Mass: 137.0841

This compound was prepared according to the standard method. After purification by column chromatography (silica gel: DCM/MeOH: 99/1), the title compound was isolated in 91% yield as a yellow oil, ee = 99%, $[\alpha]^{25}_{D}$ = -39.1 (c 1.0, EtOH).{lit. $[\alpha]^{25}_{D}$ = -23.6 (c 1.0, EtOH)}[5].

^{1}H NMR (400 MHz, $CDCl_3$) δ 7.15 (td, *J* = 7.5, 1.7 Hz, 1H), 6.97 (d, *J* = 7.6 Hz, 1H), 6.85 (d, *J* = 8.1 Hz, 1H), 6.78 (t, *J* = 7.4 Hz, 1H), 4.33 (q, *J* = 6.7 Hz, 1H), 1.48 (d, *J* = 6.7 Hz, 3H).

^{13}C NMR (101 MHz, $CDCl_3$) δ 157.7, 128.7, 128.0, 127.4, 119.2, 117.3, 51.8, 23.8.

HPLC: Chiralpak OD-3, Hexane/i-PrOH 85/15, 1 mL/min, λ=215 nm, $t_R[1]$ = 6.201, $t_R[2]$ = 8.680.

1 gram scale procedure for 2a

A 100 mL dried Schlenk tube equipped with a magnetic stir bar was charged with imine (1 g, 7.4 mmol, 100 mol%), ammonium formate (14.8 mmol, 930 mg, 2 equiv.) and (*R,R*)-A (0.037 mmol, 28 mg, 0.5 mol%). The tube was purged 3 times with argon atmosphere. The solvent of DCM/ACN (10/1, v/v, 30 mL) was added by syringe. The reaction mixture was allowed to stir for 2 h under argon atmosphere at 50 °C. Then the reaction mixture was washed with $NaHCO_3$ (aqueous saturated solution) and DCM, the organic layer was separated and the aqueous layer was extracted twice with DCM. The combined organic layers were dried over $MgSO_4$, filtered, and concentrated under vacuum. The residue was purified by flash column chromatography (1% MeOH in DCM) to provide the target product **2a** as a yellow oil in 97% yield (974 mg, 2.07 mmol, 99% ee).

(R)-2-(1-aminoethyl)-6-methylphenol **(2b)**[6]**:**

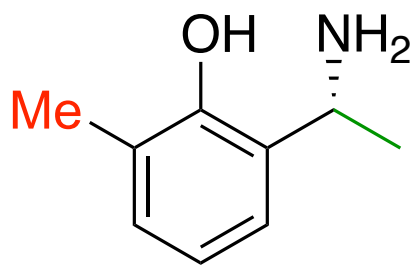

Chemical Formula: $C_9H_{13}NO$
Exact Mass: 151.0997

This compound was prepared according to the standard method. After purification by column chromatography (silica gel: DCM/MeOH: 99/1), the title compound was isolated in 91% yield as a yellow oil, ee = 99%, $[\alpha]^{25}_{D}$ = -4.211 (c 0.2, EtOH). {lit. $[\alpha]^{25}_{D}$ = +20.3 (c 0.68, $CHCl_3$) for the S enantiomer}[6].

^{1}H NMR (400 MHz, $CDCl_3$) δ 7.03 (ddd, J = 7.4, 1.8, 0.9 Hz, 1H), 6.91 – 6.57 (m, 2H), 4.31 (q, J = 6.7 Hz, 1H), 2.24 (s, 3H), 1.47 (d, J = 6.7 Hz, 3H).

^{13}C NMR (101 MHz, $CDCl_3$) δ 155.8, 129.7, 127.5, 126.0, 124.9, 118.6, 51.9, 23.9, 15.9.

HPLC: Chiralpak OD-3, Hexane/i-PrOH 85/15, 1.0 mL/min, λ=215 nm, t_R(major) = 5.594, t_R[minor] = 6.465.

(R)-2-(1-aminoethyl)-5-methylphenol **(2c)**[6]**:**

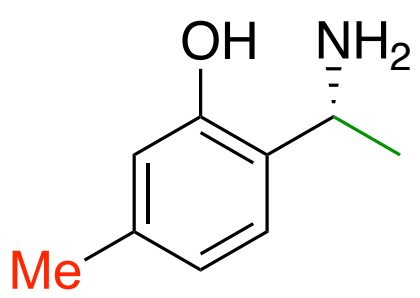

Chemical Formula: $C_9H_{13}NO$
Exact Mass: 151.0997

This compound was prepared according to the standard method. After purification by column chromatography (silica gel: DCM/MeOH: 99/1), the title compound was isolated in 91% yield as a yellow solid, ee= 96%, $[\alpha]^{25}_{D}$ = -0.562 (c 0.3, EtOH). {lit. $[\alpha]^{26}_{D}$ = +18.2 (c 1.30, MeOH) for the S enantiomer}[6].

^{1}H NMR (400 MHz, $CDCl_3$) δ 6.94 (ddd, J = 8.2, 2.3, 0.8 Hz, 1H), 6.80 – 6.71 (m, 1H), 4.28 (q, J = 6.6 Hz, 1H), 2.24 (s, 3H), 1.46 (d, J = 6.7 Hz, 3H).

^{1}H NMR (400 MHz, DMSO-d_6) δ 6.85 – 6.78 (m, 2H), 6.56 – 6.51 (m, 1H), 5.62 (v. br s, 3H, NH_3^+), 4.12 (q, J = 6.6 Hz, 1H), 2.16 (s, 3H), 1.29 (d, J = 6.7 Hz, 3H).

^{13}C NMR (101 MHz, $CDCl_3$) δ 155.3 129.0, 128.2, 127.9, 127.9, 117.1, 51.9, 24.0, 20.6.

HPLC: Chiralpak OD-3, Hexane/i-PrOH 85/15, 1.0 mL/min, λ=215 nm, t_R(major) =5.481, t_R[minor] =6.896

(*R*)-2-(1-aminoethyl)-4-methylphenol **(2d)**[6]**:**

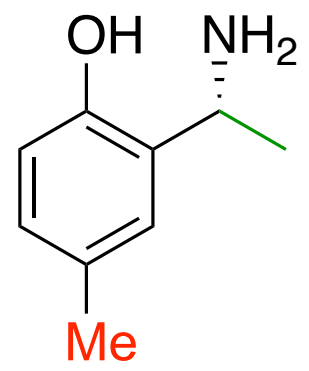

Chemical Formula: $C_9H_{13}NO$
Exact Mass: 151.0997

This compound was prepared according to the standard method. After purification by column chromatography (silica gel: DCM/MeOH: 99/1), the title compound was isolated in 91% yield as a yellow solid, ee =99%, $[\alpha]^{25}_{D}$ = -0.802 (c 0.2, EtOH). {lit. $[\alpha]^{25}_{D}$ = +2.3 (c 0.48, MeOH) for the *S* enantiomer}[6].

^{1}H NMR (400 MHz, $CDCl_3$) δ 6.95 (ddd, *J* = 8.2, 2.3, 0.7 Hz, 1H), 6.80 – 6.69 (m, 2H), 4.27 (q, *J* = 6.7 Hz, 1H), 2.24 (d, *J* = 0.8 Hz, 3H), 1.46 (d, *J* = 6.7 Hz, 3H).

^{13}C NMR (101 MHz, $CDCl_3$) δ 155.3, 128.9, 128.1, 128.0, 127.9, 117.1, 51.9, 24.0, 20.6.

HPLC: Chiralpak OD-3, Hexane/i-PrOH 85/15, 1.0 mL/min, λ=215 nm, t_R(major) =5.563, t_R[minor] =8.421.

(*R*)-2-(1-aminoethyl)-5-methoxyphenol **(2e)**[6]**:**

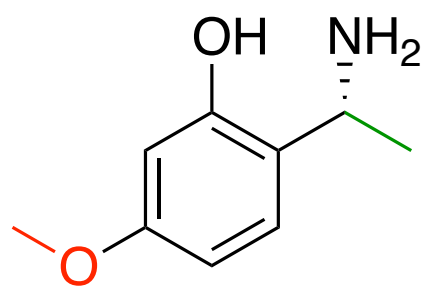

Chemical Formula: $C_9H_{13}NO_2$
Exact Mass: 167.0946

This compound was prepared according to the standard method. After purification by column chromatography (silica gel: DCM/MeOH: 99/1), the title compound was isolated in 89% yield as a yellow solid, ee =98%, $[\alpha]^{25}_{D}$ = -0.278 (c 0.3, EtOH). {lit. $[\alpha]^{25}_{D}$ = -173.9 (c 0.40, MeOH) for the *S* enantiomer}[6].

^{1}H NMR (400 MHz, $CDCl_3$) δ 6.85 (d, *J* = 8.3 Hz, 1H), 6.42 – 6.30 (m, 2H), 4.27 (q, *J* = 6.6 Hz, 1H), 3.76 (s, 3H), 1.45 (d, *J* = 6.6 Hz, 3H).

^{13}C NMR (101 MHz, $CDCl_3$) δ 160.2, 158.9, 127.8, 120.6, 105.1, 102.6, 55.3, 51.1, 24.2.

HPLC: Chiralpak OD-3, Hexane/i-PrOH 85/15, 1.0 mL/min, λ=215 nm, t_R(major) =6.9985, t_R[minor] =9.109.

(*R*)-2-(1-aminoethyl)-4-methoxyphenol **(2f)**[6]**:**

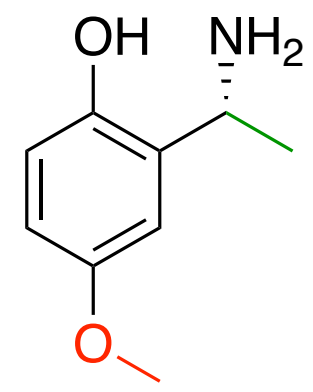

Chemical Formula: $C_9H_{13}NO_2$
Exact Mass: 167.0946

This compound was prepared according to the standard method. After purification by column chromatography (silica gel: DCM/MeOH: 99/1), the title compound was isolated in 95% yield as a yellow solid, ee =99%, $[\alpha]^{25}_D$ = -1.599 (c 0.3, EtOH). {lit. $[\alpha]^{25}_D$ = +4.57 (c 1.36, MeOH) for the *S* enantiomer}[6].

^{1}H NMR (400 MHz, $CDCl_3$) δ 6.76 (d, J = 8.7 Hz, 1H), 6.70 (dd, J = 8.8, 3.0 Hz, 1H), 6.54 (d, J = 3.0 Hz, 1H), 4.26 (q, J = 6.7 Hz, 1H), 3.74 (s, 3H), 1.46 (d, J = 6.7 Hz, 3H).

^{13}C NMR (101 MHz, $CDCl_3$) δ 152.5, 151.5, 128.9, 117.6, 113.4, 113.2, 55.9, 51.9, 23.8.

HPLC: Chiralpak OD-3, Hexane/i-PrOH 85/15, 1.0 mL/min, λ=215 nm, t_R(major) =6.998, t_R[minor] =13.228.

(*R*)-2-(1-aminoethyl)-4-fluorophenol **(2g)**[5]**:**

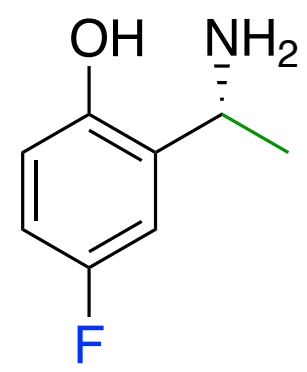

Chemical Formula: $C_8H_{10}FNO$
Exact Mass: 155.0746

This compound was prepared f according to the standard method. After purification by column chromatography (silica gel: DCM/MeOH: 99/1), the title compound was isolated in 96% yield as a yellow solid, ee =90%, $[\alpha]^{25}_D$ = -1.477 (c 0.2, EtOH), {lit. $[\alpha]^{25}_D$ = -13.8(c 1.0, EtOH)}[5].

^{1}H NMR (400 MHz, $CDCl_3$) δ 6.83 (ddd, J = 8.9, 8.1, 3.0 Hz, 1H), 6.75 (dd, J = 8.9, 4.9 Hz, 1H), 6.68 (dd, J = 9.1, 3.0 Hz, 1H), 4.27 (q, J = 6.7 Hz, 1H), 1.47 (d, J = 6.7 Hz, 3H).

^{1}H NMR (400 MHz, DMSO-d_6) δ 6.92 (dd, J = 9.7, 3.2 Hz, 1H), 6.83 (td, J = 8.6, 3.2 Hz, 1H), 6.64 (dd, J = 8.8, 4.9 Hz, 1H), 5.71 (v. br s, 3H, NH_3^+), 4.16 (q, J = 6.6 Hz, 1H), 1.28 (d, J = 6.7 Hz, 3H).

^{13}C NMR (101 MHz, $CDCl_3$) δ 156.1 (d, $^1J_{C-F}$ = 236.3 Hz), 153.6, 129.1, 117.9 (d, $^3J_{C-F}$ = 7.7 Hz), 114.8 (d, $^2J_{C-F}$ = 23.1 Hz), 113.8 (d, $^2J_{C-F}$ = 23.1 Hz), 51.5, 23.8.

^{19}F NMR (376 MHz, $CDCl_3$) δ -125.98.

HPLC: Chiralpak OD-3, Hexane/i-PrOH 85/15, 1.0 mL/min, λ=215 nm, t_R(major) =5.717, t_R[minor] =6.775.

(*R*)-2-(1-aminoethyl)-4-chlorophenol **(2h):**

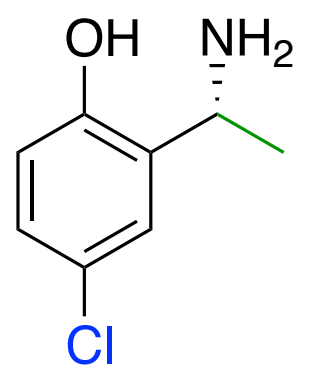

Chemical Formula: $C_8H_{10}ClNO$
Exact Mass: 171.0451

This compound was prepared according to the standard method. After purification by column chromatography (silica gel: DCM/MeOH: 99/1), the title compound was isolated in 93% yield as a yellow solid, ee = 98%, $[\alpha]^{25}_D$ = -0.927 (c 0.4, EtOH).

^1H NMR (400 MHz, $CDCl_3$) δ 7.08 (dd, *J* = 8.6, 2.6 Hz, 1H), 6.93 (dd, *J* = 2.6, 0.6 Hz, 1H), 6.75 (d, *J* = 8.6 Hz, 1H), 4.27 (q, *J* = 6.7 Hz, 1H), 1.47 (d, *J* = 6.7 Hz, 3H).

^{13}C NMR (101 MHz, $CDCl_3$) δ 156.4, 129.6, 128.3, 127.1, 123.6, 118.6, 51.6, 23.8.

HRMS(ESI) exact mass calculated for [M-H]+($C_8H_9ClNO^+$): m/z 170.0373; found: 170.0378.

HPLC: Chiralpak OD-3, Hexane/i-PrOH 85/15, 1.0 mL/min, λ=215 nm, t_R(major) =5.613, t_R[minor] =7.541.

(*R*)-2-(1-aminoethyl)-4-bromophenol **(2i)**[6]**:**

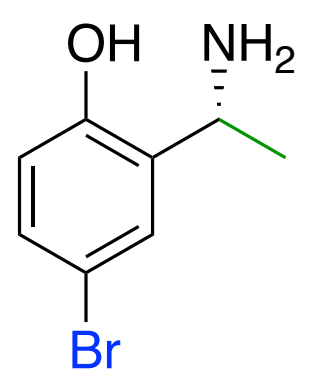

Chemical Formula: $C_8H_{10}BrNO$
Exact Mass: 214.9946

This compound was prepared according to the standard method. After purification by column chromatography (silica gel: DCM/MeOH: 99/1), the title compound was isolated in 96% yield as a yellow solid, ee = 99%, $[\alpha]^{25}_D$ = -2.875 (c 0.2, EtOH). {lit. $[\alpha]^{24}_D$ = -0.55 (c 1.66, MeOH) for the *S* enantiomer}[6].

^1H NMR (400 MHz, $CDCl_3$) δ 7.21 (dd, *J* = 8.6, 2.5 Hz, 1H), 7.06 (d, *J* = 2.5 Hz, 1H), 6.70 (d, *J* = 8.6 Hz, 1H), 4.26 (q, *J* = 6.7 Hz, 1H), 1.46 (d, *J* = 6.7 Hz, 3H).

^{13}C NMR (101 MHz, $CDCl_3$) δ 156.9, 131.2, 130.2, 130.0, 119.1, 110.8, 51.5, 23.8.

HPLC: Chiralpak OD-3, Hexane/i-PrOH 85/15, 1.0 mL/min, λ=215 nm, t_R(major) =5.701, t_R[minor] = 8.844.

(*R*)-2-(1-aminoethyl)-4-(trifluoromethyl)phenol **(2j):**

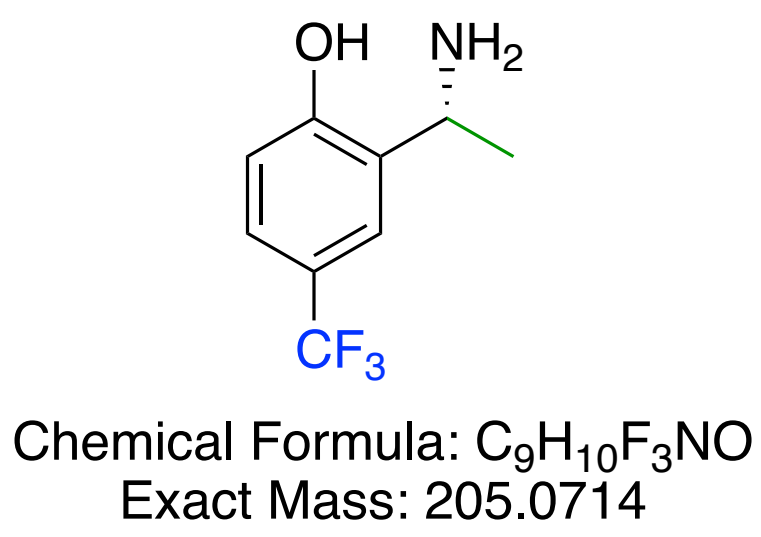

This compound was prepared according to the standard method. After purification by column chromatography (silica gel: DCM/MeOH: 99/1), the title compound was isolated in 94% yield as a yellow solid, ee =99%, $[\alpha]^{25}_{D}$ = -3.54 (c 0.2,EtOH)

^{1}H NMR (400 MHz, $CDCl_3$) δ 7.39 (dd, *J* = 8.5, 2.3 Hz, 1H), 7.21 (d, *J* = 2.3 Hz, 1H), 6.88 (dd, *J* = 8.6, 0.9 Hz, 1H), 4.36 (q, *J* = 6.7 Hz, 1H), 1.49 (d, *J* = 6.7 Hz, 3H).

^{13}C NMR (101 MHz, $CDCl_3$) δ 160.9, 128.2, 124.8 (q, $^{1}J_{C-F}$= 272.7 Hz),125.9 (q, $^{3}J_{C-F}$= 3.8 Hz), 124.6 (q, $^{3}J_{C-F}$ = 3.8 Hz), 121.2 (q, $^{2}J_{C-F}$ = 32.3 Hz), 117.5, 51.7, 23.9.

^{19}F NMR (376 MHz, $CDCl_3$) δ -61.20.

HRMS(ESI) exact mass calculated for [M-H]+($C_9H_9F_3NO^+$): m/z 204.0636; found: 204.0637.

HPLC: Chiralpak OD-3, Hexane/i-PrOH 85/15, 1.0 mL/min, λ=215 nm, t_R(major) =4.995, t_R[minor] =5.631.

(*R*)-3-(1-aminoethyl)-4-hydroxybenzonitrile **(2k):**

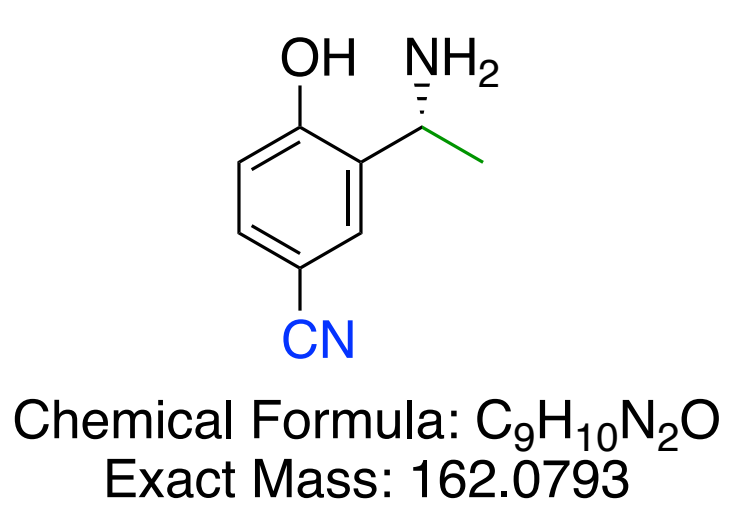

This compound was prepared according to the standard method. After purification by column chromatography (silica gel: DCM/MeOH: 99/1), the title compound was isolated in 98% yield as a yellow solid, ee =93%, $[\alpha]^{25}_{D}$ = -2.835 (c 0.3, EtOH).

^{1}H NMR (400 MHz, DMSO-d_6) δ = 7.41 – 7.31 (m, 2H), 6.55 (d, J = 8.5 Hz, 1H), 5.92 (v. br s, 3H, NH_3^+), 4.22 (q, J = 6.7 Hz, 1H), 1.36 (d, J = 6.7 Hz, 3H).

^{13}C NMR (101 MHz, DMSO-d_6) δ 167.0, 132.5, 130.8, 129.5, 120.8, 118.1, 95.1, 48.7, 21.1.

HRMS(ESI) exact mass calculated for [M-H]+($C_9H_9N_2O$+): m/z 161.0715; found: 161.0722.

SFC: Chiralpak IE, $scCO_2$/MeOH 90/10, 2.0 mL/min, λ=215 nm, t_R(major) =5.586, t_R[minor] =6.128.

(*R*)-2-(1-aminoethyl)-4-nitrophenol **(2l)**[6]**:**

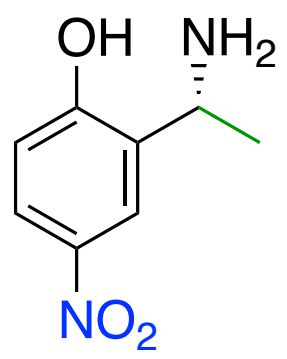

Chemical Formula: $C_8H_{10}N_2O_3$
Exact Mass: 182.0691

This compound was prepared f according to the standard method. After purification by column chromatography (silica gel: DCM/MeOH: 99/1), the title compound was isolated in 88% yield as a yellow solid, ee = $[\alpha]^{25}_D$ = -1.243 (c 0.2, EtOH). {lit. $[\alpha]^{25}_D$ = +28.0 (c 0.28, MeOH) for the *S* enantiomer}[6].

^{1}H NMR (400 MHz, MeOD). δ = 8.08 – 7.96 (m, 2H), 6.54 (d, J = 9.1 Hz, 1H), 4.50 – 4.40 (m, 1H), 1.62 (d, J = 6.8 Hz, 3H).

^{13}C NMR (101 MHz, MeOD) δ = 175.6, 135.4, 127.8, 126.5, 125.3, 120.2, 50.1, 18.5.

SFC: Chiralpak IE, $scCO_2$/MeOH 90/10, 2.0 mL/min, λ=215 nm, t_R(major) =5.825, t_R[minor] =6.619.

methyl (*R*)-3-(1-aminoethyl)-4-hydroxybenzoate **(2m):**

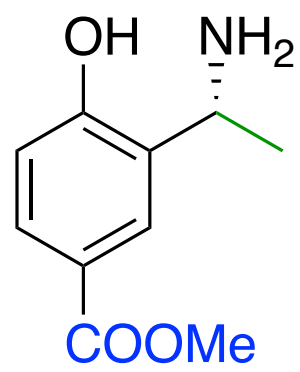

Chemical Formula: $C_{10}H_{13}NO_3$
Exact Mass: 195.0895

This compound was prepared according to the general procedure B. After purification by column chromatography (silica gel: DCM/MeOH: 99/1), the title compound was isolated in 89% yield (1.22 g, 9.1 mmol) as a yellow solid, ee =95%, $[\alpha]^{25}_D$ = -2.878 (c 0.3, EtOH).

^{1}H NMR (400 MHz, $CDCl_3$) δ 7.81 (dd, J = 8.5, 2.2 Hz, 1H), 7.67 (d, J = 2.2 Hz, 1H), 6.81 (d, J = 8.5 Hz, 1H), 4.36 (q, J = 6.7 Hz, 1H), 3.84 (s, 3H), 1.48 (d, J = 6.6 Hz, 3H).

^{13}C NMR (101 MHz, $CDCl_3$) δ 167.2, 162.5, 130.6, 129.4, 127.7, 120.9, 117.2, 51.9, 51.6, 23.8.

HRMS(ESI) exact mass calculated for [M-H]+($C_{10}H_{12}NO_3$+): m/z 194.0817; found: 194.0822.

SFC: Chiralpak IE, $scCO_2$/MeOH 90/10, 2.0 mL/min, λ=215 nm, t_R(major) =7.623, t_R[minor] =9.160.

(*R*)-3-(1-aminoethyl)-[1,1'-biphenyl]-4-ol **(2n):**

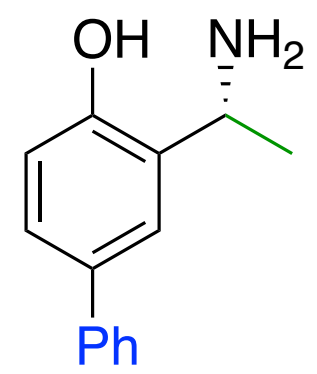

Chemical Formula: $C_{14}H_{15}NO$
Exact Mass: 213.1154

This compound was prepared according to the standard method. After purification by column chromatography (silica gel: DCM/MeOH: 99/1), the title compound was isolated in 86% yield as a yellow solid, ee =99%, $[\alpha]^{25}_D$ = -1.822 (c 0.2, MeOH).

^{1}H NMR (400 MHz, $CDCl_3$) δ 7.44 – 7.36 (m, 2H), 7.36 – 7.23 (m, 3H), 7.24 – 7.09 (m,1H), 7.08 (d, J = 2.3 Hz, 1H), 6.80 (d, J = 8.3 Hz, 1H), 4.27 (q, J = 6.7 Hz, 1H), 1.40 (d, J = 6.7 Hz, 3H).

^{13}C NMR (101 MHz, $CDCl_3$) δ 157.3, 141.2, 132.4, 128.8, 128.3, 127.3, 126.7, 126.6, 126.1, 117.7, 52.0, 23.9.

HRMS(ESI) exact mass calculated for [M+H]+($C_{14}H_{16}NO$+): m/z 214.1232; found: 214.1219.

HPLC: Chiralpak ADH, Hexane/i-PrOH 90/10, 0.8 mL/min, λ=215 nm, t_R(major) =9.101, t_R[minor] = 18.514.

(*R*)-2-(1-aminoethyl)-4,6-dimethylphenol **(2o):**

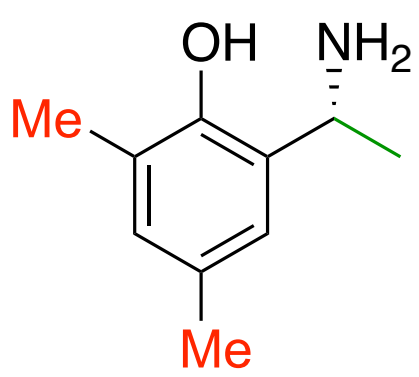

Chemical Formula: $C_{10}H_{15}NO$
Exact Mass: 165.1154

This compound was prepared according to the standard method. After purification by column chromatography (silica gel: DCM/MeOH: 99/1), the title compound was isolated in 98% yield as a yellow solid, ee =99%, $[\alpha]^{25}_D$ = -0.869 (c 0.3, EtOH).

^{1}H NMR (400 MHz, $CDCl_3$) δ 6.85 (s, 1H), 6.63 (s, 1H), 4.27 (q, J = 6.7 Hz, 1H), 2.22 (s, 3H), 2.21 (s, 3H), 1.46 (d, J = 6.7 Hz, 3H).

^{13}C NMR (101 MHz, $CDCl_3$) δ 153.3, 130.3, 127.6, 127.3, 125.8, 125.4, 51.9, 23.9, 20.6, 15.8.

HRMS(ESI) exact mass calculated for [M-H]+($C_{10}H_4NO$+): m/z 164.1075; found: 164.1082.

HPLC: Chiralpak OD-3, Hexane/i-PrOH 88/12, 0.9 mL/min, λ=215 nm, t_R(major) =6.372, t_R[minor] =7.238.

(*R*)-6-(1-aminoethyl)-2,3-dimethylphenol **(2p)**:

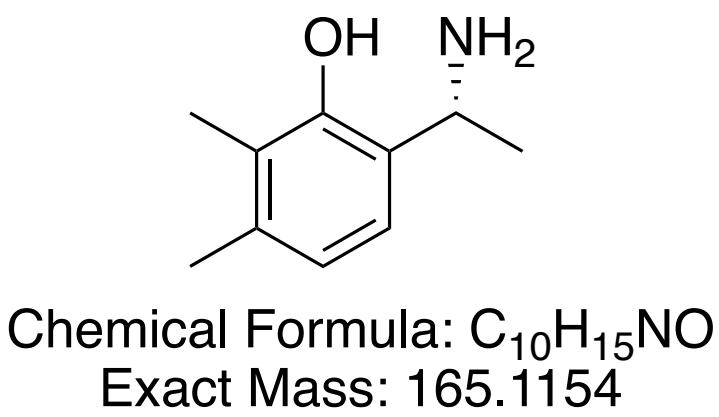

This compound was prepared according to the standard method. After purification by column chromatography (silica gel: DCM/MeOH: 99/1), the title compound was isolated in 97% yield as a yellow solid, ee =99%, $[\alpha]^{25}_D$ = -0.987 (c 0.2, EtOH).

^{1}H NMR (400 MHz, $CDCl_3$) δ = 6.74 (d, J = 7.7 Hz, 1H), 6.62 (d, J = 7.7 Hz, 1H), 4.29 (q, J = 6.7 Hz, 1H), 2.26 (s, 3H), 2.18 (s, 3H), 1.47 (d, J = 6.7 Hz, 3H).

^{13}C NMR (101 MHz, $CDCl_3$) δ = 155.5, 137.0, 125.2, 124.5, 124.0, 120.2, 51.8, 24.0, 20.1, 11.5.

HRMS(ESI) exact mass calculated for [M+H]+($C_{10}H_{16}NO^+$): m/z 166.1226; found: 166.1231.

HPLC: Chiralpak ADH, Hexane/i-PrOH 90/10, 0.8 mL/min, λ=215 nm, t_R(major) =8.507, t_R[minor] = 11.921

(*R*)-2-(1-aminoethyl)-3,5-dimethylphenol **(2q)**:

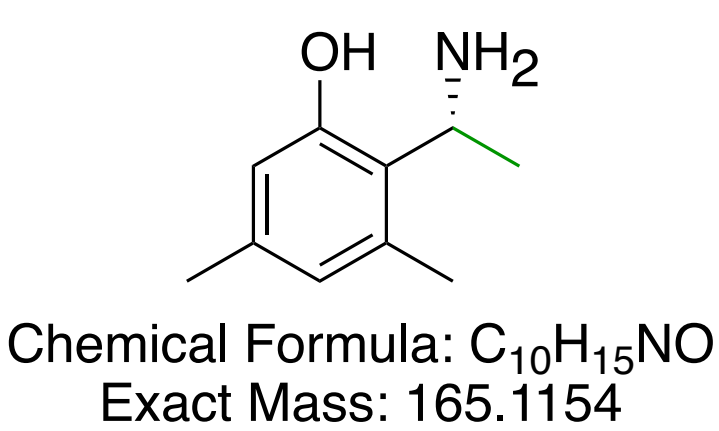

This compound was prepared according to the standard method. After purification by column chromatography (silica gel: DCM/MeOH: 99/1), the title compound was isolated in 81% yield as a yellow solid, ee =99%, $[\alpha]^{25}_D$ = -2.847 (c 0.2, EtOH).

^{1}H NMR (400 MHz, $CDCl_3$) δ 6.57 (s, 1H), 6.44 (s, 1H), 4.61 (q, J = 6.7 Hz, 1H), 3.63 – 3.54 (m, 1H), 2.20 (s, 3H), 2.19 (s, 3H), 1.43 (d, J = 6.7 Hz, 3H).

^{13}C NMR (101 MHz, $CDCl_3$) δ 157.5, 138.3, 135.3, 122.5, 122.2, 116.1, 47.5, 21.2, 21.1, 19.5.

HRMS(ESI) exact mass calculated for [M+H]+($C_{10}H_{16}NO^+$): m/z 166.1232; found: 166.1221.

HPLC: Chiralpak OD-3, Hexane/i-PrOH 90/10, 0.8 mL/min, λ=215 nm, t_R(major) =4.995, t_R[minor] = 5.631.

(*R*)-2-(1-aminoethyl)-3,5,6-trimethylphenol **(2r)**:

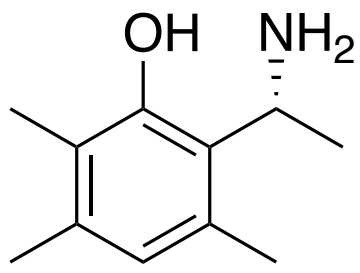

Chemical Formula: $C_{11}H_{17}NO$
Exact Mass: 179.1310

This compound was prepared according to the standard method. After purification by column chromatography (silica gel: DCM/MeOH: 99/1), the title compound was isolated in 97% yield as a yellow solid, ee =99%, $[\alpha]^{25}_D$ = -1.411 (c 0.2, EtOH).

^{1}H NMR (400 MHz, $CDCl_3$) δ 6.46 (s, 1H), 4.63 (q, J = 6.6 Hz, 1H), 2.20 (d, J = 2.0 Hz, 6H), 2.12 (s, 3H), 1.42 (d, J = 6.6 Hz, 3H).
^{13}C NMR (101 MHz, $CDCl_3$) δ 156.0, 136.4, 131.7, 123.0, 122.5, 47.6, 21.7, 19.9, 19.2, 11.3.
HRMS(ESI) exact mass calculated for [M+H]+($C_{11}H_{18}NO^+$): m/z 180.1388; found: 180.1376.

HPLC: Chiralpak ADH, Hexane/i-PrOH 90/10, 0.8 mL/min, λ=215 nm, t_R(major) =6.372, t_R[minor] = 7.238.

(*R*)-2-(1-aminopropyl)phenol **(2s)[6]:**

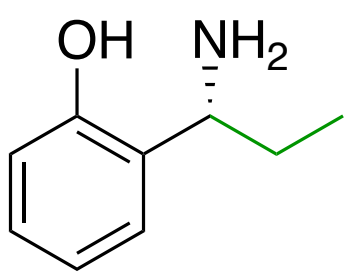

Chemical Formula: $C_9H_{13}NO$
Exact Mass: 151.0997

This compound was prepared according to the standard method. After purification by column chromatography (silica gel: DCM/MeOH: 99/1), the title compound was isolated in 93% yield as a yellow solid, ee =95%, $[\alpha]^{25}_D$ = -2.491 (c 0.2, EtOH). {lit. $[\alpha]^{24}_D$ = +21.2 (c 1.34, $CHCl_3$) for the *S* enantiomer}[6].

^{1}H NMR (400 MHz, $CDCl_3$) δ 7.14 (ddd, J = 8.1, 7.3, 1.7 Hz, 1H), 6.93 (dd, J = 7.5, 1.7 Hz, 1H), 6.84 (dd, J = 8.1, 1.2 Hz, 1H), 6.77 (td, J = 7.4, 1.2 Hz, 1H), 4.01 (t, J = 7.0 Hz, 1H), 1.92 – 1.66 (m, 2H), 0.92 (t, J = 7.4 Hz, 3H).

^{13}C NMR (101 MHz, $CDCl_3$) δ 157.8, 128.6, 128.4, 126.9, 118.9, 117.3, 58.4, 29.8, 10.9.

HPLC: Chiralpak OD-3, Hexane/i-PrOH 85/15, 1 mL/min, λ=215 nm, t_R[1] =6.985, t_R[2] = 9.747.

(*R*)-2-(amino(phenyl)methyl)phenol **(2t)**[7]**:**

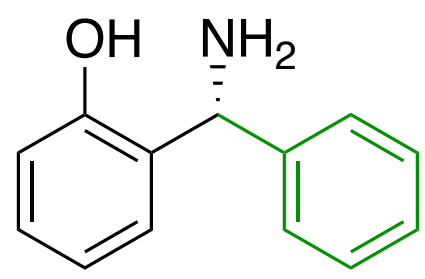

Chemical Formula: $C_{13}H_{13}NO$
Exact Mass: 199.0997

This compound was prepared according to the standard method. After purification by column chromatography (silica gel: DCM/MeOH: 99/1), the title compound was isolated in 91% yield as a yellow solid, ee =97%, $[\alpha]^{25}_{D}$ = -0.630 (c 0.2, EtOH). {lit. $[\alpha]^{25}_{D}$ = +101.3 (c 1.0, $CHCl_3$) for the *S* enantiomer}[7].

^{1}H NMR (400 MHz, $CDCl_3$) δ 7.30 – 7.09 (m, 5H), 7.04 (ddd, J = 8.2, 7.1, 1.9 Hz, 1H), 6.78 (dd, J = 8.2, 1.2 Hz, 1H), 6.67 (dd, J = 7.8, 1.9 Hz, 1H), 6.61 (td, J = 7.3, 1.2 Hz, 1H), 5.18 (s, 1H).

^{13}C NMR (101 MHz, $CDCl_3$) δ 158.0, 143.3, 129.0, 129.0, , 128.7, 127.9, 127.0, 126.4, 119.2, 117.5, 60.1.

HPLC: Chiralpak OD-3, Hexane/i-PrOH 85/15, 1.0 mL/min, λ=215 nm, t_R(major) =8.350, t_R[minor] =9.491.

(*R*)-3-(1-aminoethyl)-4'-methyl-[1,1'-biphenyl]-4-ol **(2u):**

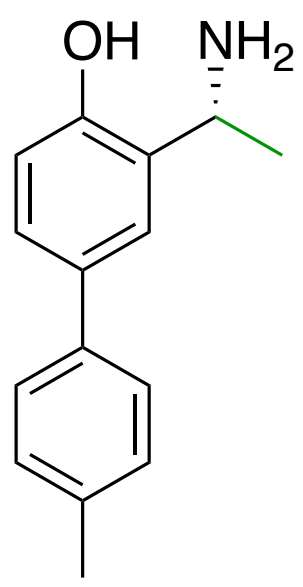

Chemical Formula: $C_{15}H_{17}NO$
Exact Mass: 227.1310

This compound was prepared according to the standard method. After purification by column chromatography (silica gel: DCM/MeOH: 99/1), the title compound was isolated in 89% yield as a yellow solid, ee =99%, $[\alpha]^{25}_{D}$ = -1.432 (c 0.3, EtOH).

^{1}H NMR (400 MHz, Acetone-d6) δ 7.26 – 7.23 (m, 1H), 7.23 – 7.15 (m, 4H), 7.11 – 7.10 (m, 1H), 7.06 – 7.03 (m, 1H), 6.72 (d, J = 8.3 Hz, 1H), 4.20 (q, J = 6.7 Hz, 1H), 2.26 (s, 3H), 1.46 (d, J = 6.7 Hz, 3H).

^{13}C NMR (101 MHz, Acetone-d6) δ 153.1, 142.8, 135.9, 133.8, 131.1, 129.1, 127.6, 126.5, 126.4, 117.5, 45.2, 20.9, 20.7.

HRMS(ESI) exact mass calculated for [M+H]+($C_{15}H_{18}NO^+$): m/z 228.1388; found: 228.1374.

HPLC: Chiralpak ADH, Hexane/i-PrOH 90/10, 0.8 mL/min, λ=215 nm, t_R(major) =7.122, t_R[minor] =14.256.

(*R*)-3-(1-aminoethyl)-4'-methoxy-[1,1'-biphenyl]-4-ol **(2v)**:

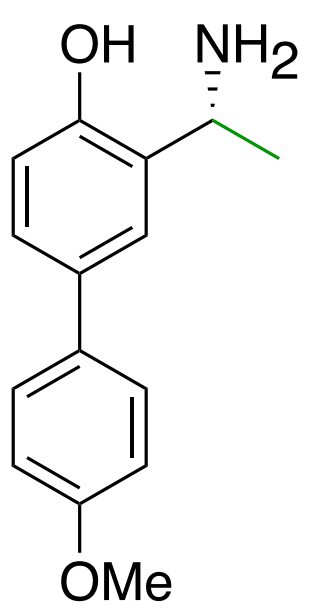

Chemical Formula: $C_{15}H_{17}NO_2$
Exact Mass: 243.1259

This compound was prepared according to the standard method. After purification by column chromatography (silica gel: DCM/MeOH: 99/1), the title compound was isolated in 81% yield as a yellow solid, ee =99%, $[\alpha]^{25}_D$ = -3.341 (c 0.2, EtOH).

^{1}H NMR (400 MHz, $CDCl_3$) δ 7.50 – 7.41 (m, 2H), 7.34 (dd, J = 8.3, 2.3 Hz, 1H), 7.15 (d, J = 2.4 Hz, 1H), 7.03 – 6.82 (m, 3H), 4.38 (q, J = 6.7 Hz, 1H), 3.84 (s, 3H), 1.52 (d, J = 6.6 Hz, 3H).

^{13}C NMR (101 MHz, $CDCl_3$) δ 158.5, 156.8, 133.8, 131.9, 128.3, 127.6, 126.8, 125.6, 117.6, 114.1, 55.4, 52.0, 24.0.

HRMS(ESI) exact mass calculated for [M+H]+($C_{15}H_{18}NO_2^+$): m/z 244.1338; found: 244.1342.

HPLC: Chiralpak ADH, Hexane/i-PrOH 90/10, 0.8 mL/min, λ=215 nm, t_R(major) =12.820, t_R[minor] =28.319.

(*R*)-3-(1-aminoethyl)-4'-fluoro-[1,1'-biphenyl]-4-ol **(2w)**:

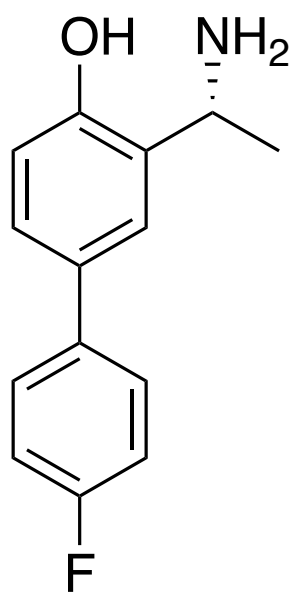

Chemical Formula: $C_{14}H_{14}FNO$
Exact Mass: 231.1059

This compound was prepared according to the standard method. After purification by column chromatography (silica gel: DCM/MeOH: 99/1), the title compound was isolated in 81% yield as a yellow solid, ee =99%, $[\alpha]^{25}_{D}$ = -2.897 (c 0.2, EtOH).

^{1}H NMR (400 MHz, $CDCl_3$) δ 7.52 – 7.40 (m, 2H), 7.33 (dd, J = 8.4, 2.4 Hz, 1H), 7.18 – 7.03 (m, 3H), 6.90 (d, J = 8.4 Hz, 1H), 4.39 (q, J = 6.7 Hz, 1H), 1.52 (d, J = 6.7 Hz, 3H).

^{13}C NMR (101 MHz, $CDCl_3$) δ 162.1 (d, $^{1}J_{C-F}$ = 245.43 Hz), 157.4, 137.4, 131.4, 128.5, 128.2 (d, $^{3}J_{C-F}$ = 8.08 Hz), 127.1, 126.0, 117.8, 115.6 (d, $^{2}J_{C-F}$ = 21.21 Hz), 52.1, 24.1.

^{19}F NMR (376 MHz, $CDCl_3$) δ -117.09.

HRMS(ESI) exact mass calculated for [M+H]+($C_{14}H_{15}FNO^+$): m/z 232.1138; found: 232.1124.

HPLC: Chiralpak ADH, Hexane/i-PrOH 90/10, 0.8 mL/min, λ=215 nm, t_R(major) =11.471, t_R[minor] =28.940.

(*R*)-3-(1-aminoethyl)-2'-methyl-[1,1'-biphenyl]-4-ol **(2x)**:

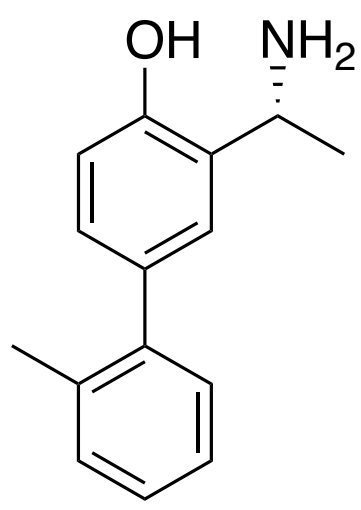

Chemical Formula: $C_{15}H_{17}NO$
Exact Mass: 227.1310

This compound was prepared according to the standard method. After purification by column chromatography (silica gel: DCM/MeOH: 99/1), the title compound was isolated in 81% yield as a yellow solid, ee =99%, $[\alpha]^{25}_{D}$ = -3.258 (c 0.2, EtOH).

^{1}H NMR (400 MHz, $CDCl_3$) δ = 7.30 – 7.18 (m, 4H), 7.11 (dd, J = 8.3, 2.3 Hz, 1H), 6.94 (d, J = 2.2 Hz, 1H), 6.89 (d, J = 8.2 Hz, 1H), 4.36 (q, J = 6.7 Hz, 1H), 2.29 (s, 3H), 1.52 (d, J = 6.7 Hz, 3H).

^{13}C NMR (101 MHz, $CDCl_3$) δ = 156.5, 141.8, 135.5, 132.8, 130.3, 129.3, 128.1 (2C), 127.6, 126.8, 125.8, 116.8, 51.8, 24.0, 20.6.

HRMS(ESI) exact mass calculated for [M+H]+($C_{15}H_{18}NO^+$): m/z 228.1383; found: 228.1390.

HPLC: Chiralpak ADH, Hexane/i-PrOH 90/10, 0.8 mL/min, λ=215 nm, t_R(major) =7.761, t_R[minor] = 14.391.

(*R*)-3-(1-aminoethyl)-3',5'-dimethyl-[1,1'-biphenyl]-4-ol **(2y)**:

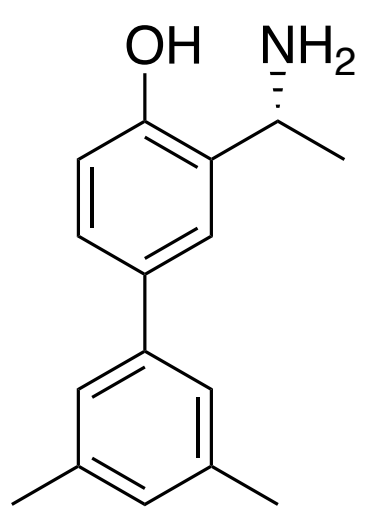

Chemical Formula: $C_{16}H_{19}NO$
Exact Mass: 241.1467

This compound was prepared according to the standard method. After purification by column chromatography (silica gel: DCM/MeOH: 99/1), the title compound was isolated in 89% yield as a yellow solid, ee =98%, $[\alpha]^{25}_D$ = -0.443(c 0.2, EtOH).

^{1}H NMR (400 MHz, $CDCl_3$) δ = 7.39 (dd, J = 8.4, 2.3 Hz, 1H), 7.18 (dd, J = 15.2, 2.0 Hz, 3H), 6.98 – 6.87 (m, 2H), 4.40 (q, J = 6.7 Hz, 1H), 2.39 (s, 6H), 1.53 (d, J = 6.7 Hz, 3H).

^{13}C NMR (101 MHz, $CDCl_3$) δ = 157.2, 141.2, 138.3, 132.5, 128.3, 128.3, 127.2, 126.1, 124.7, 117.6, 52.1, 24.0, 21.5.

HRMS(ESI) exact mass calculated for [M+H]+($C_{16}H_{20}NO^+$): m/z 242.1539; found: 242.1546.

HPLC: Chiralpak ADH, Hexane/i-PrOH 90/10, 0.8 mL/min, λ=215 nm, t_R(major) =7.962, t_R[minor] = 8.999.

(R)-3-(1-aminoethyl)-3',5'-bis(trifluoromethyl)-[1,1'-biphenyl]-4-ol **(2z)**:

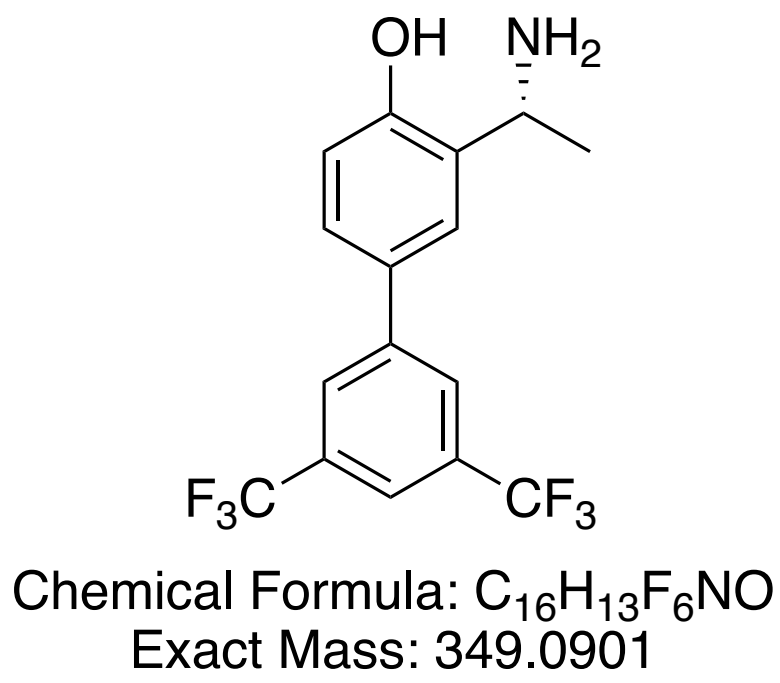

This compound was prepared according to the standard method. After purification by column chromatography (silica gel: DCM/MeOH: 99/1), the title compound was isolated in 85% yield as a yellow solid, ee =99%, $[\alpha]^{25}_{D}$ = -0.481 (c 0.2, EtOH).

^{1}H NMR (400 MHz, $CDCl_3$) δ 7.93 (d, J = 1.6 Hz, 2H), 7.85 – 7.71 (m, 1H), 7.41 (dd, J = 8.4, 2.4 Hz, 1H), 7.22 (d, J = 2.4 Hz, 1H), 6.96 (d, J = 8.4 Hz, 1H), 4.45 (q, J = 6.7 Hz, 1H), 1.55 (d, J = 6.7 Hz, 3H).

^{13}C NMR (101 MHz, $CDCl_3$) δ 158.9, 143.3, 132.6, 131.9 ($^{3}J_{C-F}$ = 3.9 Hz), 129.1 (q, $^{2}J_{C-F}$ = 33.2 Hz), 127.5, 126.9 (q, $^{1}J_{C-F}$ = 272.7 Hz), 125.0, 122.3, 121.5 ($^{3}J_{C-F}$ = 3.9 Hz), 119.6, 118.3, 52.1, 24.0.

^{19}F NMR (376 MHz, $CDCl_3$) δ = -62.85

HRMS(ESI) exact mass calculated for [M+H]+($C_{16}H_{14}F_6NO^+$): m/z 350.0974; found: 350.0987.

HPLC: Chiralpak ADH, Hexane/i-PrOH 90/10, 0.8 mL/min, λ=215 nm, t_R(major) =5.604, t_R[minor] = 6.313.

(R)-2-(1-aminoethyl)-4-(naphthalen-1-yl)phenol **(2aa)**:

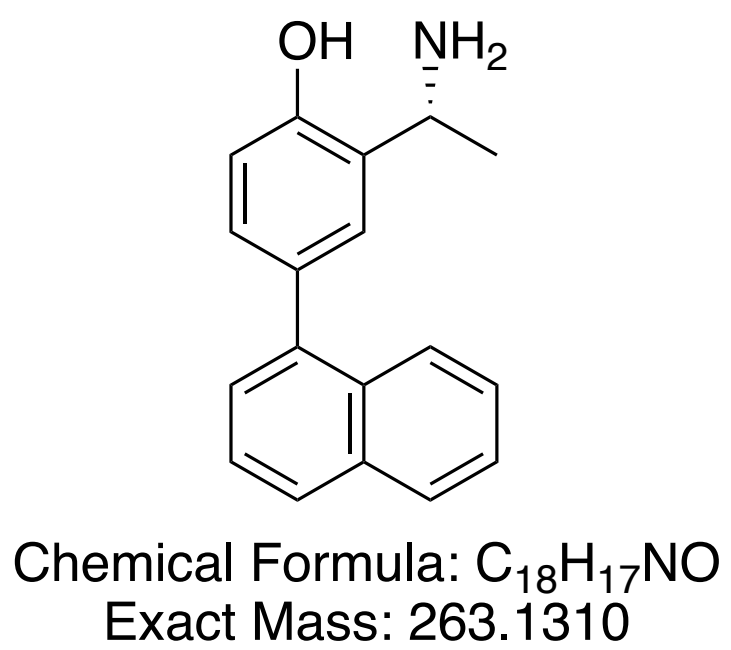

This compound was prepared according to the standard method. After purification by column chromatography (silica gel: DCM/MeOH: 99/1), the title compound was isolated in 99% yield as a yellow solid, ee =99%, $[\alpha]^{25}_{D}$ = -0.443 (c 0.3, EtOH).

^1H NMR (400 MHz, $CDCl_3$) δ = 7.92 (ddt, J = 8.2, 1.5, 0.8 Hz, 1H), 7.89 – 7.83 (m, 1H), 7.78 (dt, J = 8.2, 1.2 Hz, 1H), 7.51 – 7.32 (m, 4H), 7.29 – 7.19 (m, 1H), 7.07 (d, J = 2.2 Hz, 1H), 6.94 (d, J = 8.2 Hz, 1H), 4.38 – 4.22 (m, 1H), 1.60 – 1.39 (m, 4H).

^{13}C NMR (101 MHz, $CDCl_3$) δ = 157.1, 140.3, 134.0, 132.0, 131.6, 130.2, 129.0, 128.4, 127.9, 127.3, 127.0, 126.2, 125.8, 125.5, 117.2, 117.1, 51.8, 24.0.

HRMS(ESI) exact mass calculated for [M+H]+($C_{18}H_{18}NO+$): m/z 264.1388; found: 264.1395.

HPLC: Chiralpak ADH, Hexane/i-PrOH 90/10, 0.8 mL/min, λ=215 nm, t_R(major) =8.629, t_R[minor] = 12.368.

(*R*)-2-(1-aminoethyl)-4-(furan-3-yl)phenol **(2ab)**:

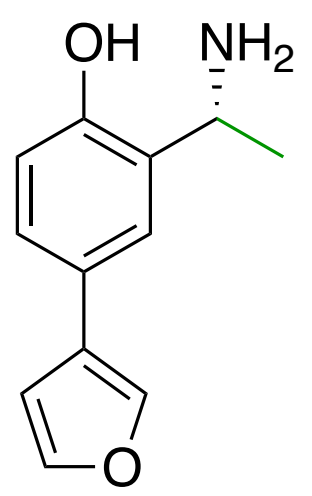

Chemical Formula: $C_{12}H_{13}NO_2$
Exact Mass: 203.0946

This compound was prepared according to the standard method. After purification by column chromatography (silica gel: DCM/MeOH: 99/1), the title compound was isolated in 95% yield as a yellow solid, ee =99%, $[\alpha]^{25}_D$ = 1-.001 (c 0.1, EtOH).

^1H NMR (400 MHz, $CDCl_3$) δ =7.44 (dd, J = 1.6, 0.9 Hz, 1H), 7.27 (d, J = 1.7 Hz, 1H), 7.13 – 7.05 (m, 1H), 6.90 (d, J = 2.3 Hz, 1H), 6.68 (d, J = 8.3 Hz, 1H), 6.45 (dd, J = 1.9, 0.9 Hz, 1H), 4.16 (q, J = 6.7 Hz, 1H), 1.32 (d, J = 6.7 Hz, 3H).

^{13}C NMR (101 MHz, $CDCl_3$) δ = 156.9, 143.5, 137.5, 128.5, 126.4, 126.1, 125.0, 123.5, 117.7, 10.0, 51.92, 23.9.

HRMS(ESI) exact mass calculated for [M+H]+($C_{12}H_{13}NO_2+$): m/z 204.1019; found: 204.1024.

HPLC: Chiralpak ADH, Hexane/i-PrOH 90/10, 0.8 mL/min, λ=215 nm, t_R(major) =11.141, t_R[minor] = 29.768.

(R)-2-(1-aminoethyl)-4-(thiophen-3-yl)phenol **(2ac)**:

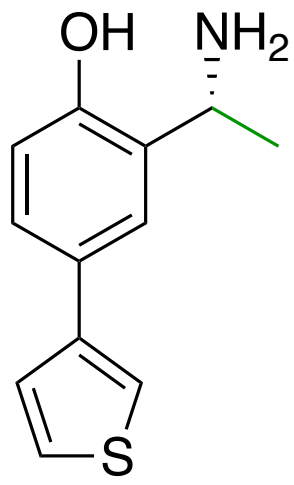

Chemical Formula: $C_{12}H_{13}NOS$
Exact Mass: 219.0718

This compound was prepared according to the standard method. After purification by column chromatography (silica gel: DCM/MeOH: 99/1), the title compound was isolated in 92% yield as a yellow solid, ee =99%, $[\alpha]^{25}_{D}$ = 2-.471 (c 0.3 EtOH).

^{1}H NMR (400 MHz, $CDCl_3$) δ = 7.36 (dd, J = 1.6, 0.9 Hz, 1H), 7.27 (d, J = 1.7 Hz, 1H), 7.25 (m, 1H), 7.24 (d, J = 2.3 Hz, 1H), 7.15(d, J = 8.3 Hz, 1H), 6.83 (dd, J = 1.9, 0.9 Hz, 1H), 4.31 (q, J = 6.7 Hz, 1H), 1.46 (d, J = 6.7 Hz, 3H).

^{13}C NMR (101 MHz, $CDCl_3$) δ 157.1, 142.4, 128.4, 127.3, 126.7, 126.3, 126.1, 125.5, 118.6, 117.7, 52.0, 24.0.

HRMS(ESI) exact mass calculated for [M+H]+($C_{12}H_{14}NOS$+): m/z 220.0791; found: 220.0796.

HPLC: Chiralpak ADH, Hexane/i-PrOH 90/10, 0.8 mL/min, λ=215 nm, t_R(major) =12.045, t_R[minor] = 39.163.

(R)-2-(1-aminoethyl)-4-(pyridin-4-yl)phenol **(2ad)**:

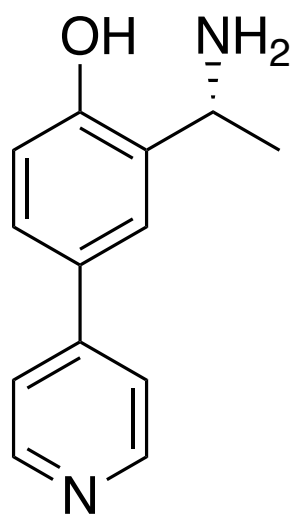

Chemical Formula: $C_{13}H_{14}N_2O$
Exact Mass: 214.1106

This compound was prepared according to the standard method. After purification by column chromatography (silica gel: DCM/MeOH: 99/1), the title compound was isolated in 81% yield as a yellow solid, ee =99%, $[\alpha]^{25}_{D}$ = -3.582 (c 0.1, EtOH).

^{1}H NMR (400 MHz, $CDCl_3$) 8.59 (s, 2H), 7.50 – 7.41 (m, 3H), 7.27 (d, J = 2.4 Hz, 1H), 6.94 (d, J = 8.4 Hz, 1H), 4.42 (q, J = 6.7 Hz, 1H), 1.53 (d, J = 6.7 Hz, 3H).

^{13}C NMR (101 MHz, MeOD) δ = 161.9, 150.8, 150.2, 130.6, 128.3, 127.8, 126.7, 122.0, 118.8, 50.2, 21.7.

HRMS(ESI) exact mass calculated for [M+H]+($C_{13}H_{15}N_2O+$): m/z 215.1184; found: 215.1172.

HPLC: Chiralpak ADH, Hexane/i-PrOH 80/20, 0.8 mL/min, λ=215 nm, t_R(major) =8.629, t_R[minor] = 12.369.

III. Postfunctionalization reactions of ortho-hydroxyaryl amine 2i

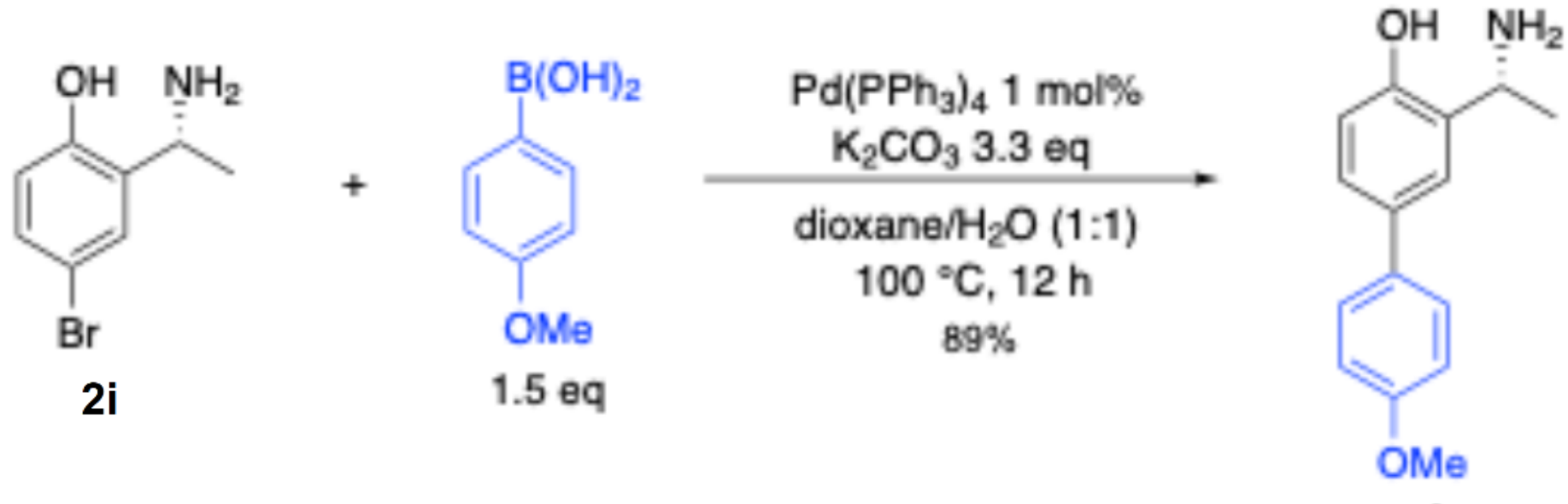

A 10 mL two-necked round-bottomed flask was charged with (*R*)-2-(1-aminoethyl)-4-bromophenol (**2i**) (64.5 mg, 0.3 mmol), (4- methoxyphenyl) boronic acid (68.4 mg, 0.45 mmol mmol, 1.5 equiv), K_2CO_3 (137.0 mg, 1 mmol, 3.3 equiv) and $Pd(PPh_3)_4$ (3.5 mg, 0.003 mmol, 1 mol%). The tube was charged under an argon atmosphere, and the solvent (dioxane/H_2O 1/1, v/v, 2 mL) was injected by syringes. The reaction mixture was allowed to stir for 12 h under argon atmosphere at 100 °C. After completion of the reaction (monitored by TLC), the reaction mixture was diluted with EA and washed with saturated $NaHCO_3$ and brine. The organic phase was dried over anhydrous Na_2SO_4 and concentrated under vacuum. The residue was purified by flash column chromatography on silica gel (5% ethyl acetate in petroleum ether) to afford **2v** as a yellow solid (65 mg, 89%).

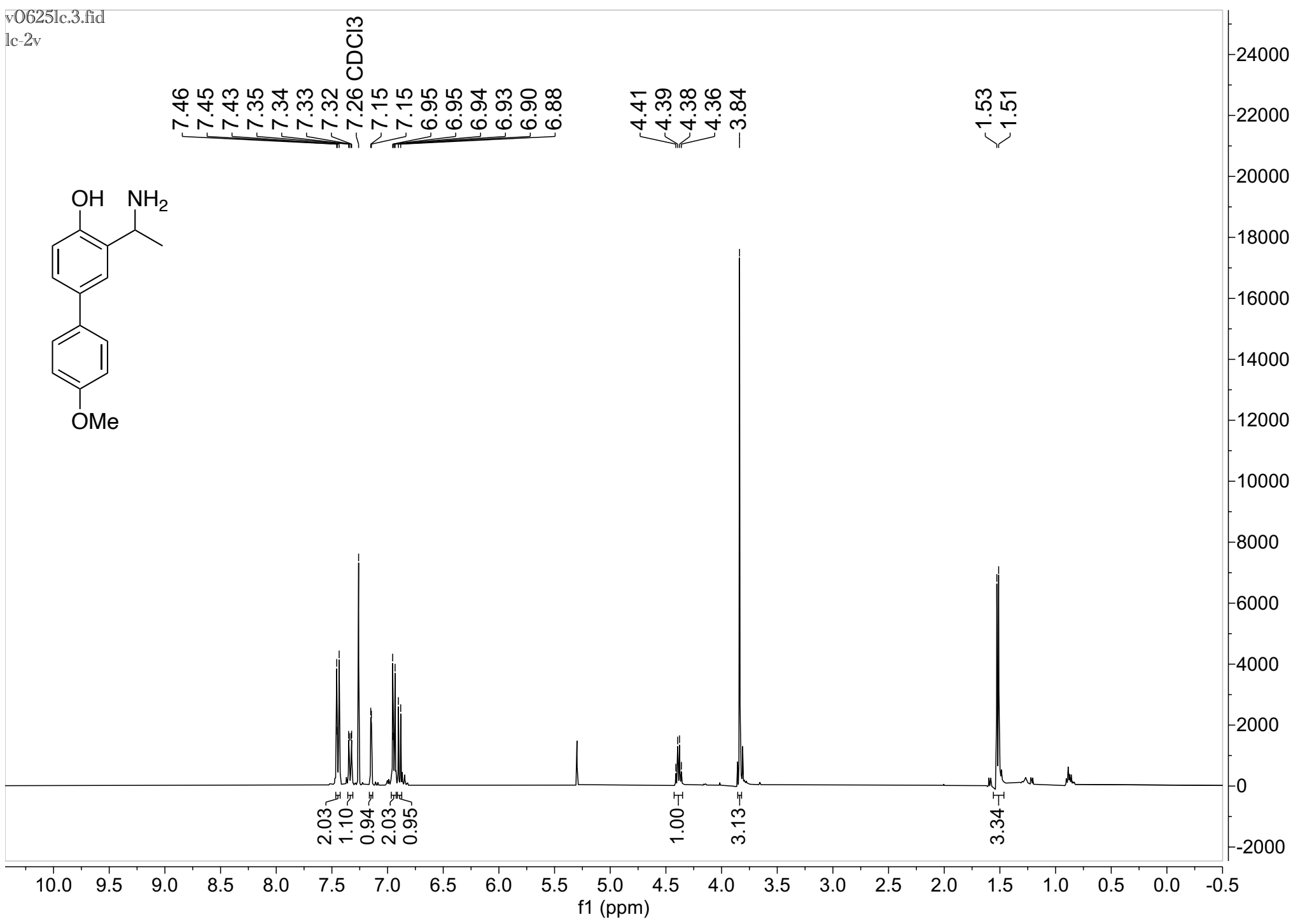

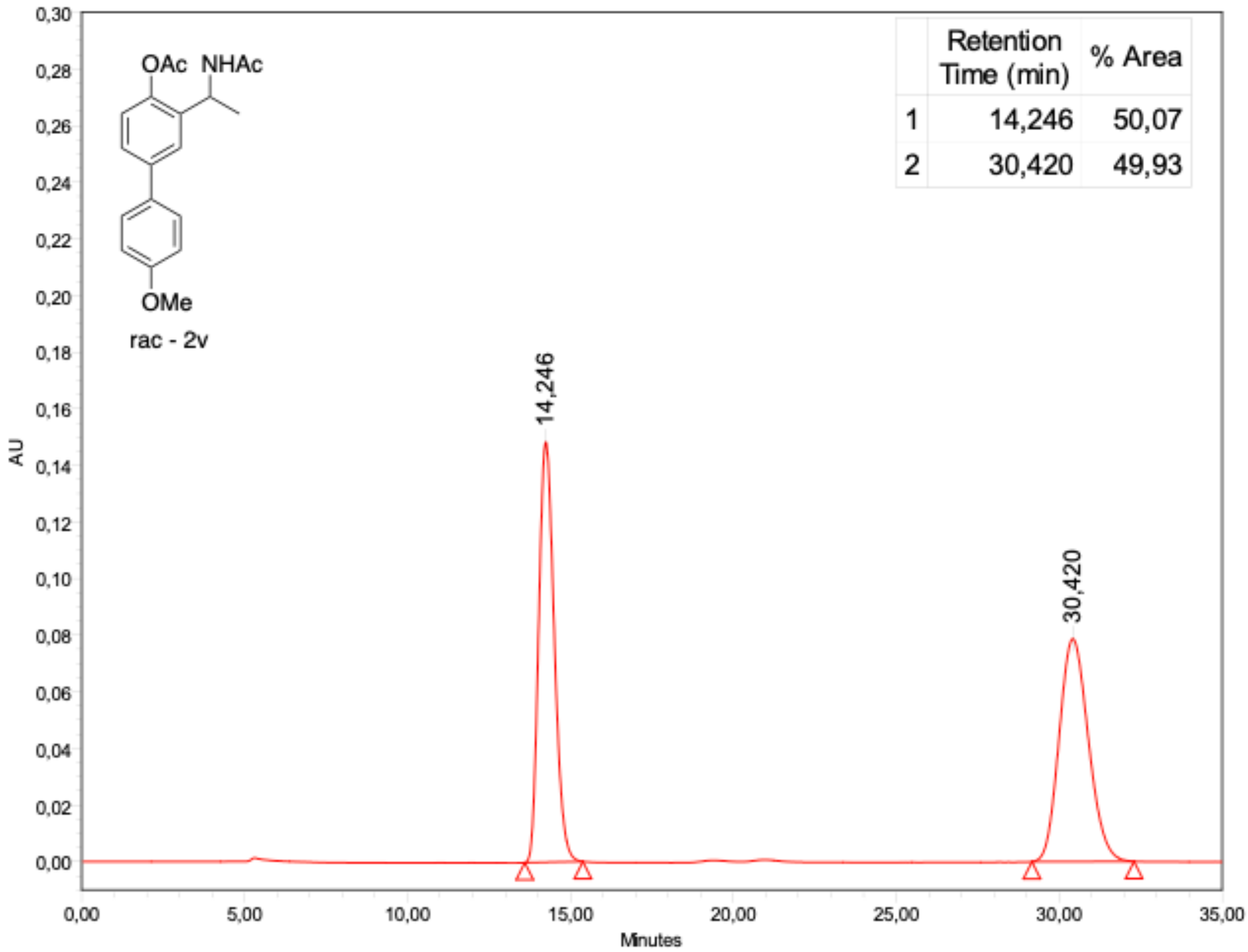
OAc NHAc
OMe
rac - 2v
Retention Time (min)
% Area
1 14,246 50,07
2 30,420 49,93
14,246
30,420
AU
Minutes
0,00
5,00
10,00
15,00
20,00
25,00
30,00
35,00
0,30
0,28
0,26
0,24
0,22
0,20
0,18
0,16
0,14
0,12
0,10
0,08
0,06
0,04
0,02
0,00

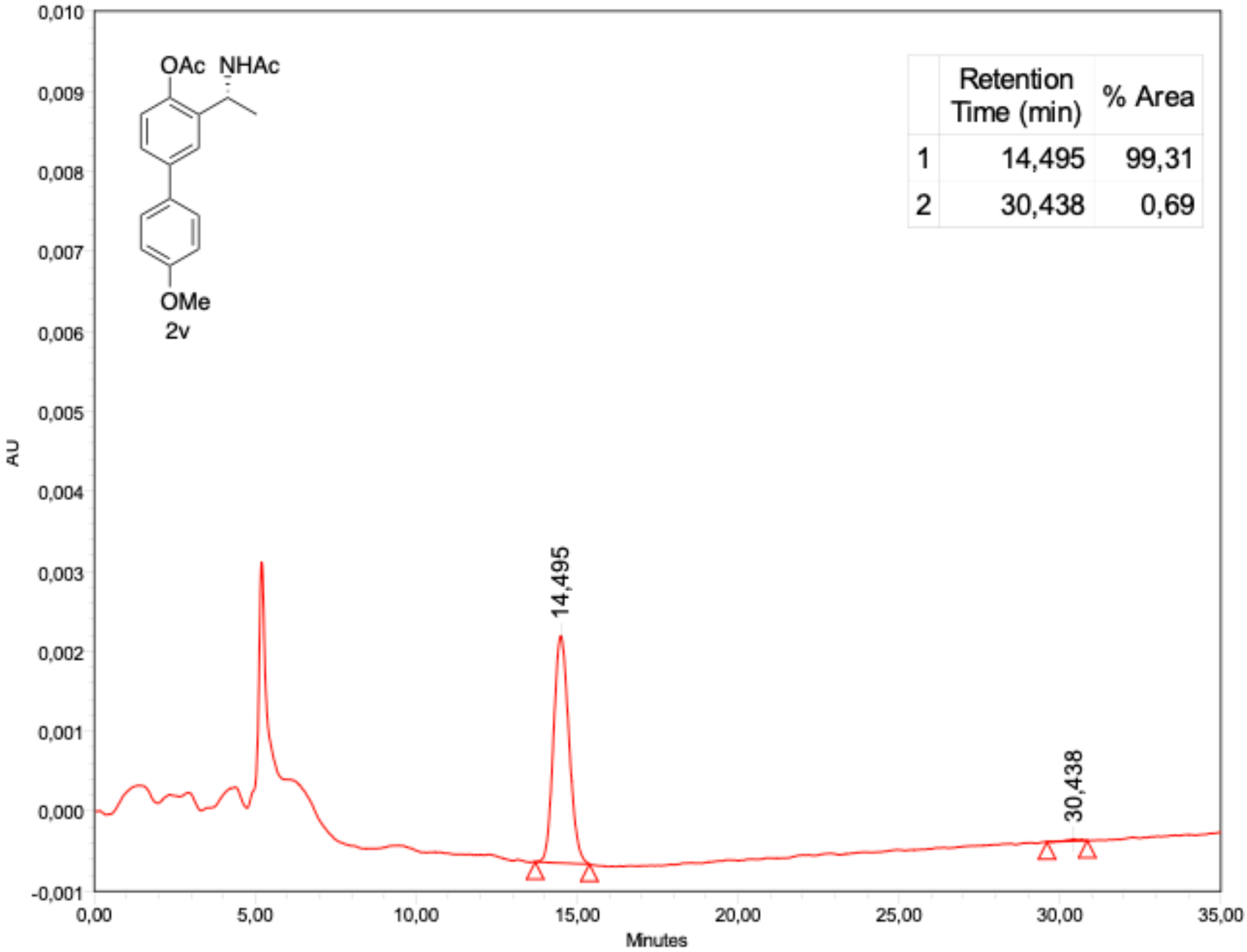
OAc NHAc
OMe
2v
Retention Time (min)
% Area
1 14,495 99,31
2 30,438 0,69
14,495
30,438
AU
Minutes
0,00
5,00
10,00
15,00
20,00
25,00
30,00
35,00
0,010
0,009
0,008
0,007
0,006
0,005
0,004
0,003
0,002
0,001
0,000
-0,001

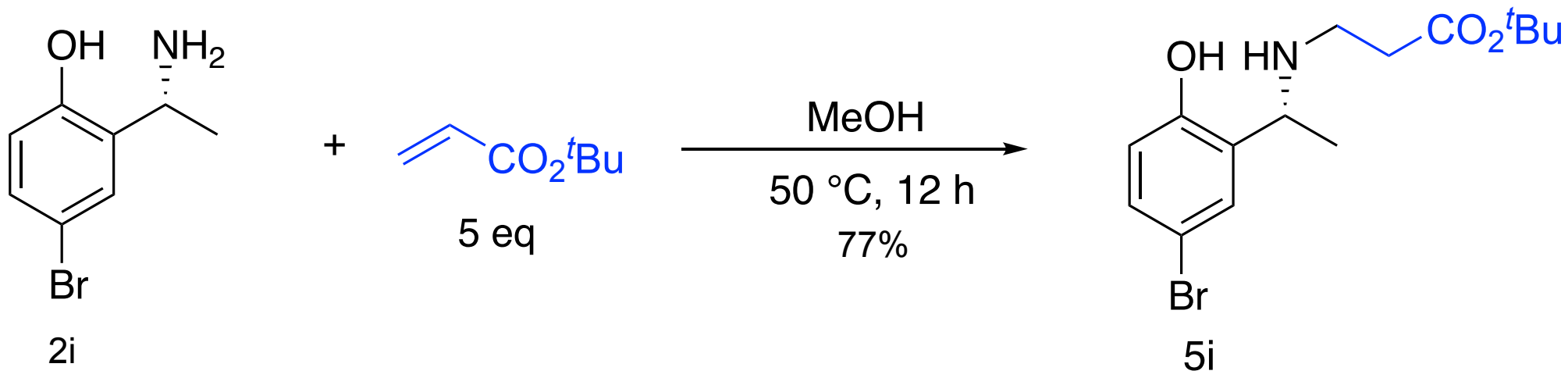

A 10 mL two-necked round-bottomed flask was charged with (*R*)-2-(1-aminoethyl)-4-bromophenol (**2i**) (43.0 mg，0.2 mmol). The tube was charged under an argon atmosphere, and the *tert*-butyl acrylate (128 mg, 1 mmol) and MeOH (1 mL) were injected by syringes at room temperature. The reaction mixture was allowed to stir for 12 h under argon atmosphere at 50 °C. After completion of the reaction (monitored by TLC), the mixture was concentrated under vacuum. The residue was purified by flash column chromatography on silica gel (ethyl acetate/cyclohexane 1:1) to afford **5i** as a colorless oil (53 mg, 77%).

tert-butyl (*R*)-3-((1-(5-bromo-2-hydroxyphenyl)ethyl)amino)propanoate **(5i)**:

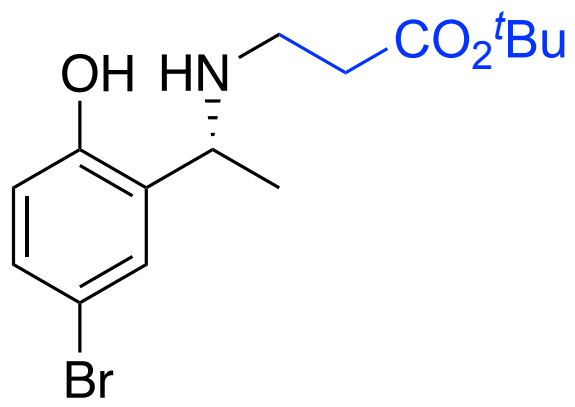

Chemical Formula: $C_{15}H_{22}BrNO_3$
Exact Mass: 343.0783

This compound was prepared according to the standard method. After purification by column chromatography (silica gel: EtOAc/cyclohexane: 1/1), the title compound was isolated in 77% yield as a colorless oil.

^1H NMR (400 MHz, $CDCl_3$) δ 7.22 (dd, J = 8.6, 2.5 Hz, 1H), 7.08 (d, J = 2.5 Hz, 1H), 6.72 (d, J = 8.6 Hz, 1H), 3.93 (q, J = 6.7 Hz, 1H), 3.64 (s, 1H), 2.89 (ddd, J = 12.3, 8.1, 5.0 Hz, 1H), 2.81 – 2.69 (m, 1H), 2.56 – 2.43 (m, 2H), 1.46 (d, J = 3.0 Hz, 3H), 1.45 (s, 9H).

^{13}C NMR (101 MHz, $CDCl_3$) δ 171.9, 156.2, 131.6, 130.9 127.7, 118.8, 111.1, 81.7, 58.2, 42.7, 34.2, 28.2, 21.6.

HRMS(ESI) exact mass calculated for $[M+H]^+$($C_{15}H_{23}BrNO_3{}^+$): m/z 344.0861; found: 344.0846.

IV. Kinetic studies

The respective kinetic performances of catalysts (*R*,*R*)-**A**, (*R*,*R*)-**B**, (*R*,*R*)-**C**, and (*R*,*R*)-**D** towards the ATH of imine **1g** into amine **2g** under the optimized conditions were evaluated by ^{19}F NMR monitoring. To do so, imine **1g** (91.8 mg, 0.6 mmol), NH_4COOH (75.6 mg, 1.2 mmol) and 1 mol% of the screened catalyst were introduced in a DCM/ACN mixture (5.4 mL/0.54 mL); standard procedure described in this experimental section was followed and reaction mixture was heated at 50 °C under Ar atmosphere. 300 µL aliquots of the reaction mixture were taken repeteadly at reaction times t = 0, 15, 30, 45, 60, 90, 120, 150, 180, 210, 240, 270 and 540 minutes. Fluorobenzene PhF was used as an internal standard for ^{19}F NMR. 10 µL PhF were dissolved into 8 mL $CDCl_3$, and 0.4 mL of this internal standard solution were syringed and mixed with each aliquot prior to analysis. Integration of ^{19}F NMR signals of **1g** and **2g** was performed and the percentage of **2g** for each aliquot and each of the screened catalyst is given in the following table.

Table S2: Yield for the ATH of **1g** into **2g** using catalysts (*R*,*R*)-**A**, (*R*,*R*)-**B**, (*R*,*R*)-**C**, and (*R*,*R*)-**D** in optimized conditions.

t (minutes)	%(**2g**) with catalyst (*R*,*R*)-**A**	%(**2g**) with catalyst (*R*,*R*)-**B**	%(**2g**) with catalyst (*R*,*R*)-**C**	%(**2g**) with catalyst (*R*,*R*)-**D**
0	0	0	0	0
15	7	3	4	4
30	22	7	8	8
45	39	12	16	13
60	57	19	22	19
90	89	36	41	32
120	99	52	59	39
150	100	76	80	63
180	100	89	91	85
210	100	99	99	99
240	100	100	100	100
270	100	100	100	100
540	100	100	100	100

^{19}F NMR kinetic monitoring of the ATH of **1g** into **2g** using catalyst (*R*,*R*)-**A**:

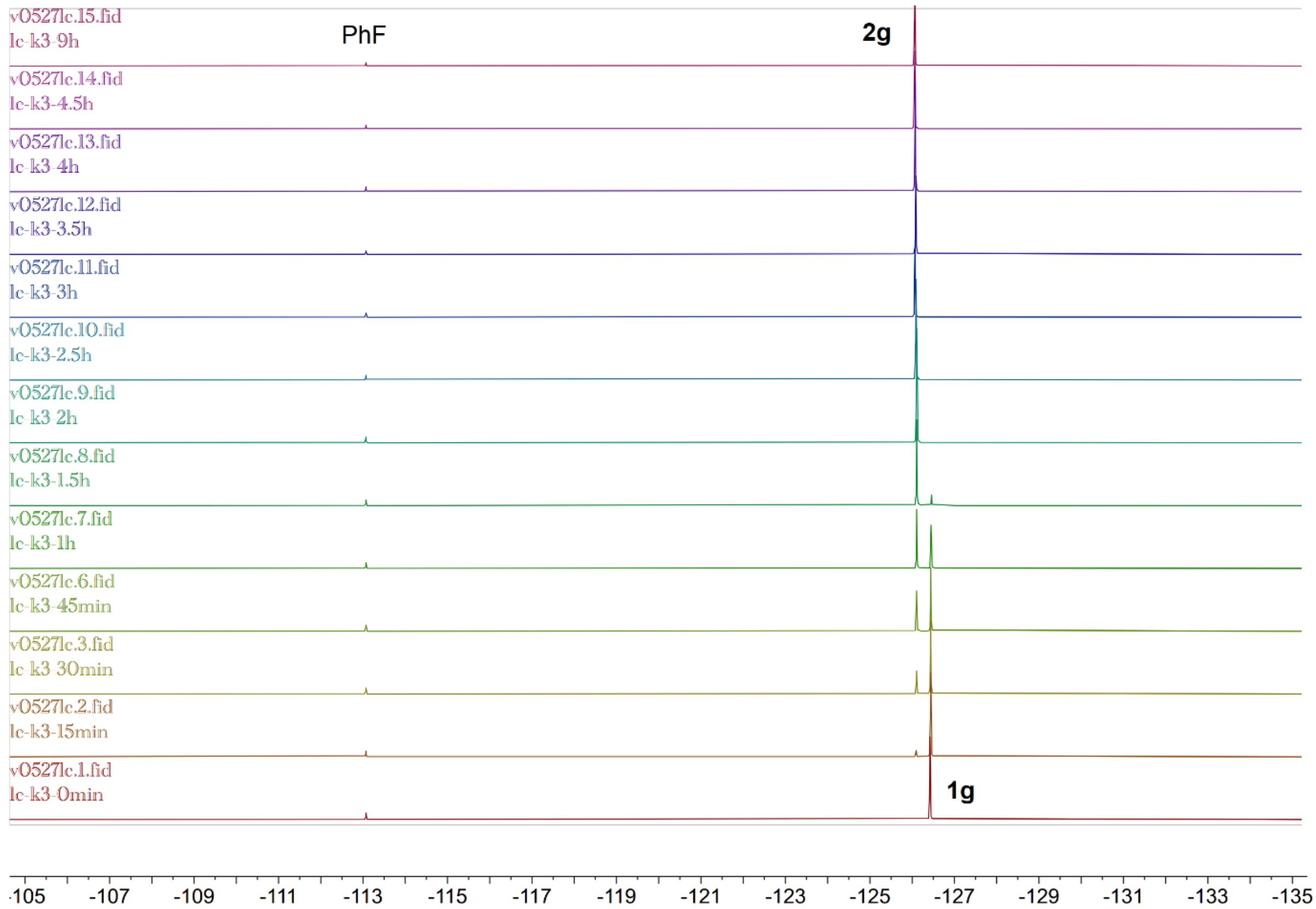

^{19}F NMR kinetic monitoring of the ATH of **1g** into **2g** using catalyst (*R*,*R*)-**B**:

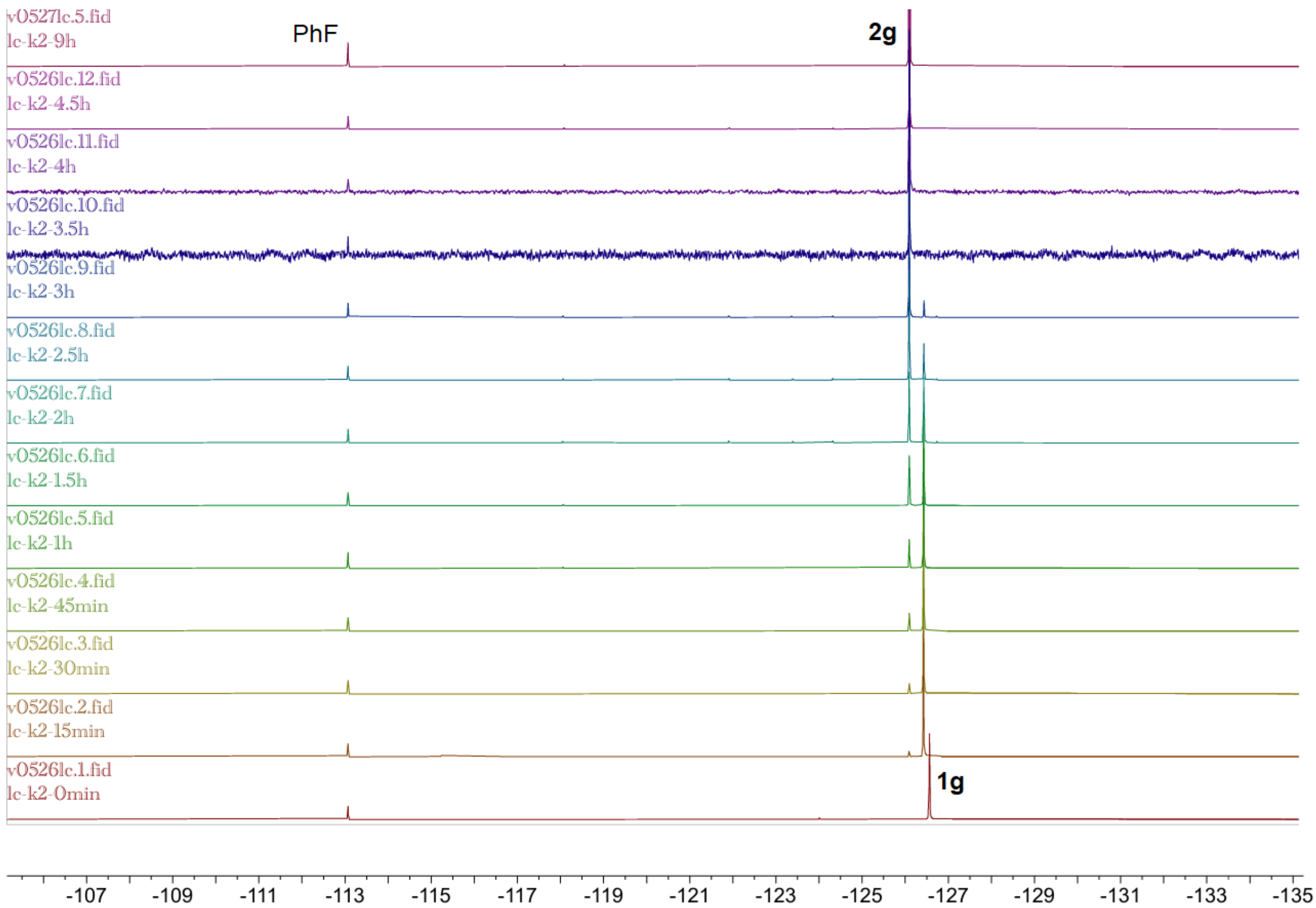

^{19}F NMR kinetic monitoring of the ATH of **1g** into **2g** using catalyst (*R*,*R*)-**C**:

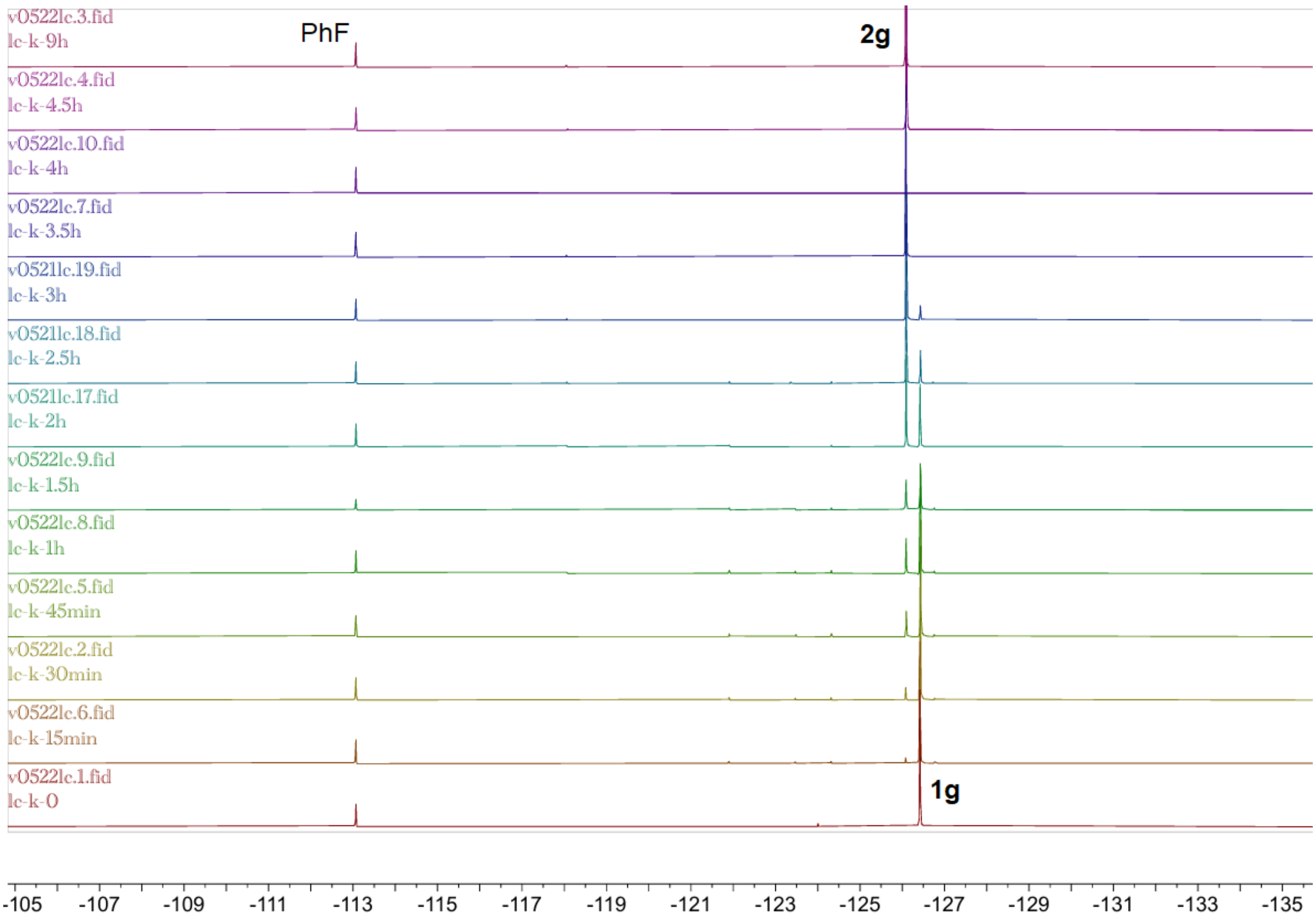

^{19}F NMR kinetic monitoring of the ATH of **1g** into **2g** using catalyst (*R*,*R*)-**D**:

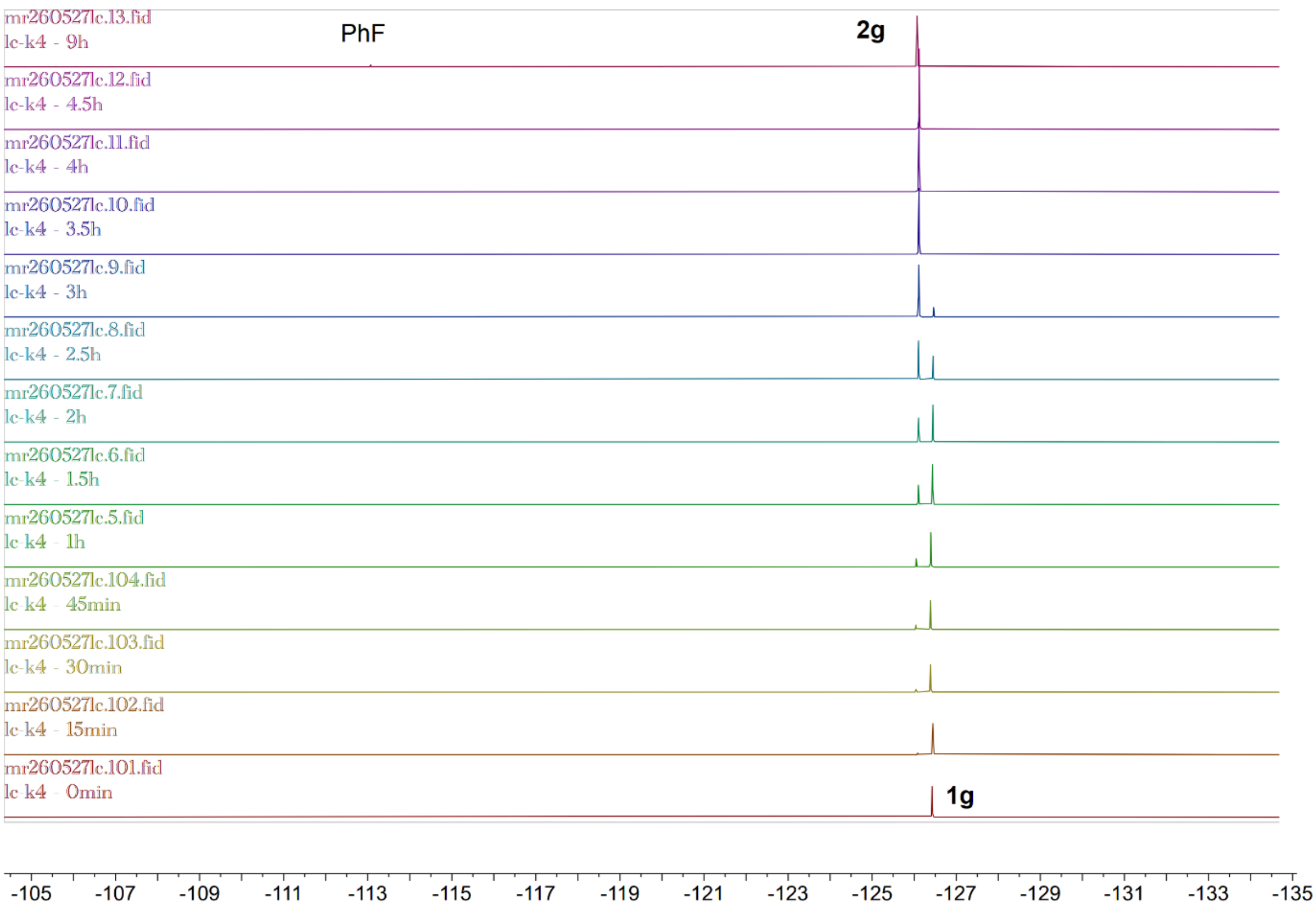

V. DFT calculations

All DFT computations were performed using Gaussian16 version C.01 [8] at the M06 level,[9] using the Grimme's dispersion GD3 damping function.[10] Solvation was taken into account using the PCM implicit continuum model, implemented in the Gaussian code.[11,12] Unless specified otherwise, all structures were characterized as minima by frequency analysis (no imaginary frequency, except for transition states which were characterized by a sole imaginary frequency corresponding to the expected vibration mode), and were optimized without geometrical constraint. Unrestricted DFT methods were applied. LANL2DZ basis set and pseudopotential were chosen for Rh,[13] and def2svp was chosen for C, H, O, N, F and S.[14]

Computed structures:

I

C	2.243212000	-1.698637000	1.427335000
C	2.726588000	-2.652548000	0.527780000
C	2.618618000	-1.783067000	2.769665000
C	3.565193000	-3.674465000	0.964453000
H	2.442880000	-2.579856000	-0.528225000
C	3.452554000	-2.808090000	3.212257000
H	2.247029000	-1.033080000	3.477758000
C	3.928069000	-3.757595000	2.309565000
H	3.941545000	-4.412784000	0.249310000
H	3.736198000	-2.862636000	4.267522000
H	4.585660000	-4.562203000	2.652297000
C	-0.575363000	-2.032367000	2.019654000
C	-0.595530000	-3.429441000	2.029258000
C	-0.913549000	-1.342907000	3.188666000
C	-0.927773000	-4.127386000	3.187907000
H	-0.333361000	-3.973818000	1.114705000
C	-1.253922000	-2.037581000	4.346883000
H	-0.897158000	-0.246476000	3.209452000
C	-1.255642000	-3.432030000	4.350520000
H	-0.931033000	-5.221055000	3.182364000
H	-1.512542000	-1.485936000	5.255177000
H	-1.516052000	-3.977651000	5.261997000
C	1.257020000	-0.643152000	0.972630000
C	-0.119561000	-1.295812000	0.785764000
N	1.612521000	0.031228000	-0.277085000
N	-1.054167000	-0.248780000	0.330895000
S	2.772029000	1.169149000	-0.096094000
O	2.848044000	1.973444000	-1.322292000
O	2.658114000	1.895610000	1.184386000
C	4.999567000	0.127210000	1.169960000

C	6.138925000	-0.671264000	1.215216000
C	6.588674000	-1.355439000	0.080781000
C	5.883688000	-1.191780000	-1.120202000
C	4.744138000	-0.399604000	-1.178303000
C	4.299613000	0.245043000	-0.025089000
H	4.636590000	0.649780000	2.059179000
H	6.687271000	-0.777890000	2.157714000
H	6.234233000	-1.704943000	-2.022171000
H	4.185714000	-0.287508000	-2.113491000
C	7.766525000	-2.274550000	0.160893000
H	7.450550000	-3.280188000	0.491440000
H	8.260073000	-2.394653000	-0.815314000
H	8.512509000	-1.919053000	0.888082000
C	-2.426629000	-0.714942000	0.057268000
H	-2.888877000	-1.064499000	0.997337000
H	-2.996718000	0.173906000	-0.260317000
H	-0.035616000	-2.021243000	-0.045655000
H	1.147920000	0.092000000	1.801591000
H	-1.108868000	0.486672000	1.039620000
C	-2.227275000	-2.869110000	-3.115271000
C	-2.902446000	-4.015732000	-2.703485000
C	-3.401126000	-4.073704000	-1.400911000
C	-3.209356000	-2.978726000	-0.549110000
C	-2.541821000	-1.827091000	-0.957666000
C	-2.031850000	-1.764616000	-2.281346000
H	-1.835332000	-2.824346000	-4.136942000
H	-3.027002000	-4.845226000	-3.402423000
H	-3.596440000	-3.060683000	0.471908000
O	-4.061999000	-5.123407000	-0.878046000
C	-4.273803000	-6.256391000	-1.680860000
H	-3.321100000	-6.706274000	-2.012647000
H	-4.815202000	-6.988546000	-1.068644000
H	-4.882983000	-6.018887000	-2.571091000
H	-0.084279000	2.000685000	-0.539419000
Rh	-0.123019000	0.644329000	-1.381126000
C	-1.252246000	-0.627153000	-2.808747000
C	-1.698712000	0.764807000	-2.879708000
C	0.099591000	-0.689647000	-3.222659000
C	-0.593471000	1.543128000	-3.355473000
C	-3.104565000	1.239941000	-2.742204000
C	0.528278000	0.668672000	-3.512265000
C	1.000587000	-1.872469000	-3.238846000
C	-0.624421000	2.993952000	-3.683825000
H	-3.177391000	2.155787000	-2.134058000

H	-3.536509000	1.469142000	-3.732421000
H	-3.749654000	0.473372000	-2.286352000
C	1.857151000	1.032545000	-4.065168000
H	1.242129000	-2.190117000	-4.268421000
H	1.954343000	-1.636933000	-2.734931000
H	0.549510000	-2.732981000	-2.720151000
H	-0.871847000	3.150582000	-4.748745000
H	-1.377925000	3.528092000	-3.085916000
H	0.351749000	3.467145000	-3.496733000
H	1.875362000	0.869079000	-5.158002000
H	2.108676000	2.083127000	-3.867002000
H	2.657238000	0.419490000	-3.624396000

Sum of electronic and thermal Energies= -2312.119120
Sum of electronic and thermal Enthalpies= -2312.118176
Sum of electronic and thermal Free Energies= -2312.236322

II

C	-1.098092000	3.151281000	1.142023000
N	-1.274010000	2.341988000	2.166210000
C	0.042167000	4.001693000	1.100693000
C	1.007897000	3.973570000	2.197565000
C	0.244697000	4.878390000	-0.001843000
C	2.126327000	4.874979000	2.065916000
C	1.330183000	5.713608000	-0.073910000
H	-0.485122000	4.888350000	-0.817283000
C	2.276473000	5.701097000	0.983893000
H	2.854931000	4.863092000	2.883123000
H	1.466520000	6.378827000	-0.930148000
H	3.145291000	6.366526000	0.932298000
C	-2.119111000	3.136589000	0.055456000
H	-1.661249000	2.858782000	-0.906097000
H	-2.924723000	2.422641000	0.273859000
H	-2.565749000	4.133917000	-0.074348000
O	0.894347000	3.223954000	3.202666000
H	-0.534635000	2.390104000	2.898530000
H	-2.081880000	1.730624000	2.213352000

Sum of electronic and thermal Energies= -439.463682
Sum of electronic and thermal Enthalpies= -439.462738
Sum of electronic and thermal Free Energies= -439.505849

II'

C	-1.113895000	3.085893000	1.210592000
N	-1.380172000	2.244268000	2.136463000
C	0.075138000	3.990353000	1.154247000

C	1.058328000	4.055660000	2.171292000
C	0.258155000	4.836282000	0.046277000
C	2.146381000	4.925395000	2.055732000
C	1.339264000	5.701249000	-0.072503000
H	-0.477685000	4.816425000	-0.760351000
C	2.290793000	5.744563000	0.943626000
H	2.886944000	4.947933000	2.862996000
H	1.436549000	6.338163000	-0.955184000
H	3.150608000	6.416710000	0.874803000
C	-2.089079000	3.174840000	0.068605000
H	-1.618415000	2.917224000	-0.894462000
H	-2.910290000	2.471085000	0.256161000
H	-2.508367000	4.188825000	-0.037092000
O	0.940176000	3.269636000	3.263290000
H	1.679110000	3.424499000	3.867462000
H	-0.658069000	2.265123000	2.859153000

Sum of electronic and thermal Energies= -439.446744
Sum of electronic and thermal Enthalpies= -439.445799
Sum of electronic and thermal Free Energies= -439.490432

III

Rh	-1.064251000	0.940058000	-0.712010000
S	2.040747000	0.308925000	-1.313460000
O	2.235455000	1.736697000	-0.968890000
O	2.084500000	0.020297000	-2.765506000
O	-5.869580000	-4.072678000	0.124644000
N	0.689909000	-0.278729000	-0.656244000
N	-1.426185000	-0.334201000	0.959629000
C	0.670934000	-1.373655000	0.297870000
C	-0.807724000	-1.633012000	0.637770000
C	1.282837000	-2.668449000	-0.188625000
C	1.076873000	-3.090324000	-1.504524000
C	1.572443000	-4.318356000	-1.937053000
C	2.277738000	-5.139113000	-1.056216000
C	2.480148000	-4.726613000	0.260767000
C	1.983855000	-3.497783000	0.689993000
C	-0.978154000	-2.666636000	1.721084000
C	-0.712614000	-2.361405000	3.060040000
C	-0.825818000	-3.348719000	4.037601000
C	-1.199897000	-4.646216000	3.689280000
C	-1.471917000	-4.954448000	2.356570000
C	-1.367867000	-3.965501000	1.381261000
C	3.418541000	-0.577324000	-0.593985000
C	4.153161000	-1.461271000	-1.378166000

C	5.209764000	-2.162595000	-0.805880000
C	5.540593000	-2.000880000	0.546281000
C	4.802685000	-1.085022000	1.308416000
C	3.749522000	-0.372002000	0.744805000
C	6.632528000	-2.815348000	1.166820000
C	-2.857963000	-0.395709000	1.293121000
C	-3.697199000	-1.084501000	0.238459000
C	-4.414625000	-2.210085000	0.662315000
C	-5.185732000	-2.965683000	-0.224198000
C	-5.254307000	-2.577819000	-1.566187000
C	-4.549510000	-1.464675000	-1.993492000
C	-3.759328000	-0.697400000	-1.122234000
C	-5.762809000	-4.543827000	1.444103000
C	-2.992129000	0.432054000	-1.684072000
C	-3.014301000	1.800457000	-1.166152000
C	-2.034122000	2.552927000	-1.886114000
C	-1.373462000	1.662102000	-2.792743000
C	-2.012934000	0.355621000	-2.705939000
C	-4.031838000	2.367099000	-0.235273000
C	-1.817250000	4.020494000	-1.767735000
C	-0.322546000	2.028417000	-3.780030000
C	-1.570369000	-0.830609000	-3.486360000
H	1.158847000	-1.104533000	1.265385000
H	-1.291400000	-2.005408000	-0.284477000
H	0.536248000	-2.430148000	-2.191173000
H	1.410060000	-4.636447000	-2.971510000
H	2.669464000	-6.102253000	-1.396879000
H	3.032872000	-5.364222000	0.957924000
H	2.143660000	-3.172087000	1.724842000
H	-0.414646000	-1.345237000	3.348125000
H	-0.620227000	-3.101614000	5.083215000
H	-1.279435000	-5.419097000	4.459415000
H	-1.770414000	-5.968798000	2.075556000
H	-1.585023000	-4.200778000	0.332181000
H	3.879660000	-1.597815000	-2.428140000
H	5.789226000	-2.862229000	-1.418720000
H	5.057838000	-0.932368000	2.362842000
H	3.169816000	0.331397000	1.347065000
H	6.245285000	-3.797302000	1.492329000
H	7.057116000	-2.322770000	2.055207000
H	7.447291000	-3.014314000	0.453664000
H	-3.178610000	0.642595000	1.479514000
H	-2.996682000	-0.938079000	2.245229000
H	-4.332728000	-2.508366000	1.710953000

H	-5.861360000	-3.171328000	-2.255264000
H	-4.604684000	-1.165748000	-3.045519000
H	-4.715953000	-4.781421000	1.708499000
H	-6.362032000	-5.460850000	1.508563000
H	-6.154285000	-3.812926000	2.174029000
H	-4.714227000	3.047956000	-0.773868000
H	-3.572593000	2.940515000	0.586369000
H	-4.659247000	1.579487000	0.208478000
H	-2.153371000	4.399242000	-0.787879000
H	-2.385791000	4.564734000	-2.542886000
H	-0.757545000	4.290124000	-1.897798000
H	0.516625000	1.314756000	-3.755818000
H	0.086224000	3.029495000	-3.574825000
H	-0.734346000	2.037513000	-4.805221000
H	-1.962376000	-0.808192000	-4.518964000
H	-1.906317000	-1.773121000	-3.024620000
H	-0.468626000	-0.850163000	-3.553422000
H	-0.884271000	0.091329000	1.719505000
C	0.772902000	3.385573000	1.796972000
N	1.730540000	2.490906000	1.801632000
H	-0.319857000	1.886793000	0.287505000
C	-0.402575000	3.180909000	2.588429000
C	-1.442753000	4.145300000	2.565708000
C	-0.604698000	1.923750000	3.291237000
C	-2.655343000	3.925462000	3.178614000
H	-1.286672000	5.089770000	2.033450000
C	-1.891050000	1.735478000	3.895248000
C	-2.872876000	2.696895000	3.843377000
H	-3.439845000	4.686118000	3.147938000
H	-2.050029000	0.775559000	4.398663000
H	-3.839146000	2.509264000	4.324615000
C	0.905302000	4.536685000	0.860195000
H	0.232623000	4.367683000	-0.000754000
H	1.933334000	4.618533000	0.483544000
H	0.623029000	5.495052000	1.320849000
O	0.265728000	0.994347000	3.332042000
H	1.547983000	1.663252000	2.406764000
H	2.424874000	2.492629000	1.050590000

Sum of electronic and thermal Energies= -2751.610744
Sum of electronic and thermal Enthalpies= -2751.609800
Sum of electronic and thermal Free Energies= -2751.744926

TS1

Rh	-0.819801000	1.030894000	-1.147127000

S	2.235675000	0.047274000	-1.716915000
O	2.567247000	1.493243000	-1.680270000
O	2.145687000	-0.512285000	-3.082593000
O	-5.871479000	-3.639234000	0.250203000
N	0.906779000	-0.218611000	-0.853217000
N	-1.256244000	-0.027336000	0.667311000
C	0.793785000	-1.240172000	0.180814000
C	-0.705137000	-1.382188000	0.495995000
C	1.327765000	-2.615994000	-0.152739000
C	1.027724000	-3.214307000	-1.379030000
C	1.426314000	-4.522116000	-1.643724000
C	2.129310000	-5.248361000	-0.681992000
C	2.433466000	-4.656881000	0.543435000
C	2.033874000	-3.348124000	0.804074000
C	-0.966034000	-2.302993000	1.661230000
C	-0.709268000	-1.896442000	2.975683000
C	-0.910597000	-2.782944000	4.032452000
C	-1.363509000	-4.079169000	3.788958000
C	-1.625071000	-4.487213000	2.481249000
C	-1.432582000	-3.599533000	1.425540000
C	3.581296000	-0.805129000	-0.904938000
C	4.215080000	-1.855927000	-1.562507000
C	5.240125000	-2.541184000	-0.921145000
C	5.641949000	-2.196834000	0.375294000
C	5.009809000	-1.115758000	1.003020000
C	3.986584000	-0.418033000	0.371263000
C	6.694685000	-2.989103000	1.082991000
C	-2.689738000	0.011107000	0.999931000
C	-3.554843000	-0.772858000	0.044088000
C	-4.334531000	-1.797821000	0.590502000
C	-5.120799000	-2.625340000	-0.215559000
C	-5.133591000	-2.416708000	-1.599670000
C	-4.361346000	-1.406796000	-2.147177000
C	-3.562170000	-0.568194000	-1.353312000
C	-5.833998000	-3.936241000	1.624087000
C	-2.732588000	0.446639000	-2.027523000
C	-2.734471000	1.873353000	-1.744554000
C	-1.746379000	2.478376000	-2.589567000
C	-1.081695000	1.448477000	-3.323344000
C	-1.728493000	0.186314000	-3.010938000
C	-3.710889000	2.618775000	-0.902230000
C	-1.550333000	3.941233000	-2.736564000
C	-0.022189000	1.638481000	-4.346859000
C	-1.350044000	-1.118374000	-3.609913000

H	1.280165000	-0.908943000	1.127885000
H	-1.183989000	-1.818976000	-0.400230000
H	0.487077000	-2.635554000	-2.133881000
H	1.186435000	-4.978679000	-2.608646000
H	2.442889000	-6.275727000	-0.889576000
H	2.987669000	-5.217729000	1.302377000
H	2.268829000	-2.884300000	1.769240000
H	-0.365224000	-0.872274000	3.173408000
H	-0.711225000	-2.456897000	5.057570000
H	-1.516202000	-4.772728000	4.621093000
H	-1.983165000	-5.501708000	2.282147000
H	-1.640719000	-3.914984000	0.395787000
H	3.885884000	-2.136198000	-2.566152000
H	5.735218000	-3.373699000	-1.432523000
H	5.323356000	-0.818921000	2.009157000
H	3.500316000	0.414977000	0.885930000
H	6.250319000	-3.876962000	1.566419000
H	7.187609000	-2.402163000	1.872173000
H	7.463966000	-3.356124000	0.386613000
H	-2.976300000	1.075104000	1.050639000
H	-2.841781000	-0.391658000	2.017340000
H	-4.289497000	-1.959726000	1.670815000
H	-5.750972000	-3.067827000	-2.224012000
H	-4.371943000	-1.250336000	-3.230893000
H	-4.810879000	-4.186109000	1.958643000
H	-6.481808000	-4.807508000	1.782083000
H	-6.212407000	-3.097620000	2.234838000
H	-4.307973000	3.308574000	-1.523039000
H	-3.211365000	3.227024000	-0.126970000
H	-4.417340000	1.938935000	-0.403573000
H	-1.794844000	4.481330000	-1.808449000
H	-2.229336000	4.325134000	-3.518829000
H	-0.525139000	4.199607000	-3.038872000
H	0.754905000	0.863175000	-4.263960000
H	0.469481000	2.616785000	-4.238087000
H	-0.451029000	1.588795000	-5.363497000
H	-1.773375000	-1.222644000	-4.624780000
H	-1.717857000	-1.965093000	-3.009614000
H	-0.253629000	-1.194291000	-3.697502000
H	-0.730180000	0.443607000	1.430706000
C	0.701619000	3.124907000	0.664377000
N	1.779039000	2.406379000	1.063615000
H	-0.066528000	2.194371000	-0.197764000
C	-0.372137000	3.371429000	1.643179000

C	-1.204038000	4.499818000	1.526251000
C	-0.627788000	2.394301000	2.674842000
C	-2.269587000	4.719066000	2.386523000
H	-1.000628000	5.237987000	0.743371000
C	-1.737170000	2.652703000	3.533452000
C	-2.530461000	3.775650000	3.393501000
H	-2.890517000	5.612833000	2.282574000
H	-1.939854000	1.907527000	4.310998000
H	-3.372519000	3.930275000	4.077048000
C	1.016852000	4.155810000	-0.381689000
H	0.118496000	4.545790000	-0.870695000
H	1.673332000	3.716830000	-1.147669000
H	1.542549000	4.998928000	0.098473000
O	0.062271000	1.315533000	2.784699000
H	1.551377000	1.716919000	1.792582000
H	2.397217000	2.130708000	0.296437000

Sum of electronic and thermal Energies= -2751.599328
Sum of electronic and thermal Enthalpies= -2751.598384
Sum of electronic and thermal Free Energies= -2751.734510
Lowest vibration frequency (/cm^-1): -357.9067

TS2

Rh	-0.778896000	1.158688000	-0.711369000
S	2.012021000	0.081926000	-1.906650000
O	2.331637000	1.522550000	-1.861730000
O	1.723657000	-0.435636000	-3.264632000
O	-5.943241000	-3.443916000	0.518332000
N	0.806205000	-0.242730000	-0.881269000
N	-1.116981000	-0.094845000	0.996367000
C	0.833585000	-1.272950000	0.144768000
C	-0.621549000	-1.431388000	0.628793000
C	1.337714000	-2.633267000	-0.278832000
C	0.893475000	-3.220137000	-1.466697000
C	1.270824000	-4.520352000	-1.793668000
C	2.094614000	-5.248557000	-0.934830000
C	2.544079000	-4.666589000	0.250156000
C	2.167079000	-3.365468000	0.573388000
C	-0.768845000	-2.473607000	1.708596000
C	-0.365790000	-2.224079000	3.024983000
C	-0.421144000	-3.239712000	3.978518000
C	-0.880044000	-4.509483000	3.630889000
C	-1.303260000	-4.757832000	2.325409000
C	-1.252688000	-3.742310000	1.373962000
C	3.449931000	-0.801178000	-1.332251000

C	3.990017000	-1.841130000	-2.084057000
C	5.096272000	-2.524405000	-1.591999000
C	5.668583000	-2.187463000	-0.358004000
C	5.119493000	-1.118811000	0.361750000
C	4.015616000	-0.424400000	-0.119198000
C	6.815085000	-2.977608000	0.188978000
C	-2.514680000	-0.066513000	1.453613000
C	-3.498129000	-0.677957000	0.488953000
C	-4.274397000	-1.743024000	0.959721000
C	-5.183451000	-2.404208000	0.129553000
C	-5.323220000	-1.985795000	-1.198993000
C	-4.551668000	-0.938498000	-1.671881000
C	-3.629956000	-0.264875000	-0.853815000
C	-5.791103000	-3.946583000	1.822133000
C	-2.805558000	0.795709000	-1.460814000
C	-2.701805000	2.173853000	-1.006497000
C	-1.762182000	2.833887000	-1.861926000
C	-1.232819000	1.878220000	-2.784338000
C	-1.917388000	0.618494000	-2.563696000
C	-3.584961000	2.841882000	-0.010658000
C	-1.492652000	4.292813000	-1.866550000
C	-0.293586000	2.158286000	-3.900750000
C	-1.666058000	-0.624496000	-3.336429000
H	1.428703000	-0.951474000	1.033097000
H	-1.208600000	-1.763188000	-0.248445000
H	0.255079000	-2.641201000	-2.142506000
H	0.918496000	-4.970430000	-2.726715000
H	2.390301000	-6.270059000	-1.192087000
H	3.195024000	-5.229062000	0.926639000
H	2.516208000	-2.903762000	1.505331000
H	-0.026219000	-1.219373000	3.309803000
H	-0.102067000	-3.034987000	5.005029000
H	-0.915825000	-5.305428000	4.380604000
H	-1.675170000	-5.748100000	2.046131000
H	-1.579755000	-3.937029000	0.345638000
H	3.529168000	-2.113894000	-3.036890000
H	5.523808000	-3.349088000	-2.172744000
H	5.563013000	-0.826992000	1.320664000
H	3.589473000	0.406750000	0.449671000
H	7.396813000	-2.400180000	0.922986000
H	7.494710000	-3.314252000	-0.608989000
H	6.450883000	-3.884818000	0.702618000
H	-2.750077000	0.988973000	1.672389000
H	-2.591311000	-0.601717000	2.417306000

H	-4.134902000	-2.065744000	1.994915000
H	-6.037019000	-2.507256000	-1.842079000
H	-4.658503000	-0.620348000	-2.714183000
H	-4.758165000	-4.292100000	2.007490000
H	-6.472787000	-4.800849000	1.918802000
H	-6.055794000	-3.193772000	2.585551000
H	-4.223731000	3.596309000	-0.501124000
H	-3.015221000	3.364744000	0.775234000
H	-4.254928000	2.121581000	0.482012000
H	-1.663127000	4.749567000	-0.879317000
H	-2.181708000	4.793287000	-2.570254000
H	-0.468997000	4.527575000	-2.194228000
H	0.281567000	3.078687000	-3.722195000
H	-0.851210000	2.287173000	-4.845816000
H	0.425098000	1.336090000	-4.038400000
H	-2.164737000	-0.581762000	-4.321088000
H	-2.047759000	-1.512728000	-2.809087000
H	-0.584919000	-0.754479000	-3.509228000
H	-0.553864000	0.297066000	1.779149000
C	0.789108000	3.060617000	1.353649000
N	-0.297655000	3.379799000	2.092374000
H	0.267670000	2.032612000	0.286083000
C	1.801438000	2.219606000	2.007471000
C	3.169050000	2.448989000	1.769741000
C	1.392469000	1.223677000	2.968555000
C	4.155020000	1.745813000	2.443487000
H	3.469193000	3.211642000	1.045517000
C	2.438197000	0.476190000	3.590283000
C	3.772284000	0.742954000	3.348834000
H	5.211405000	1.957211000	2.255738000
H	2.139564000	-0.296553000	4.307372000
H	4.540650000	0.163882000	3.873482000
C	1.234021000	4.086384000	0.356019000
H	0.367073000	4.594621000	-0.085616000
H	1.821110000	3.621309000	-0.448839000
H	1.848420000	4.849666000	0.863108000
O	0.157639000	0.998130000	3.236534000
H	-1.008047000	3.975373000	1.679840000
H	-0.576289000	2.660928000	2.771386000

Sum of electronic and thermal Energies= -2751.596582

Sum of electronic and thermal Enthalpies= -2751.595638

Sum of electronic and thermal Free Energies= -2751.728284

Lowest vibration frequency (/cm^-1): -535.1411

TS3

Rh	-0.927618000	0.891227000	-0.973547000
S	2.095981000	-0.035041000	-1.721156000
O	2.396540000	1.417884000	-1.716581000
O	1.921551000	-0.600997000	-3.076349000
O	-5.833108000	-4.013346000	-0.160466000
N	0.833710000	-0.320673000	-0.766810000
N	-1.375496000	-0.248507000	0.761461000
C	0.771716000	-1.349294000	0.261250000
C	-0.714778000	-1.551799000	0.620158000
C	1.332485000	-2.708026000	-0.097790000
C	0.972240000	-3.324022000	-1.300024000
C	1.406203000	-4.615226000	-1.588231000
C	2.203675000	-5.306811000	-0.675508000
C	2.565953000	-4.698285000	0.525193000
C	2.130135000	-3.406158000	0.810526000
C	-0.887751000	-2.440513000	1.825321000
C	-0.604923000	-1.963604000	3.109761000
C	-0.720536000	-2.803610000	4.215123000
C	-1.120306000	-4.129269000	4.048290000
C	-1.411584000	-4.609397000	2.772300000
C	-1.299202000	-3.766542000	1.668589000
C	3.526132000	-0.853502000	-1.024582000
C	4.051080000	-1.966759000	-1.677662000
C	5.139387000	-2.630530000	-1.126310000
C	5.717452000	-2.201146000	0.075024000
C	5.197957000	-1.055594000	0.690493000
C	4.112876000	-0.375784000	0.146035000
C	6.842954000	-2.968024000	0.693191000
C	-2.823060000	-0.332057000	1.022391000
C	-3.624250000	-1.055980000	-0.029454000
C	-4.360189000	-2.170178000	0.391525000
C	-5.125530000	-2.921141000	-0.504114000
C	-5.167250000	-2.540671000	-1.850284000
C	-4.436180000	-1.444043000	-2.274029000
C	-3.651937000	-0.683327000	-1.391363000
C	-5.764639000	-4.474443000	1.165277000
C	-2.837210000	0.412730000	-1.949176000
C	-2.823332000	1.800317000	-1.500947000
C	-1.813374000	2.483786000	-2.257619000
C	-1.163439000	1.534331000	-3.104249000
C	-1.832240000	0.252938000	-2.945217000
C	-3.800796000	2.466153000	-0.595139000
C	-1.545840000	3.940801000	-2.168690000

C	-0.101793000	1.818588000	-4.103838000
C	-1.445254000	-0.992154000	-3.658498000
H	1.282043000	-1.011535000	1.194362000
H	-1.186777000	-2.045869000	-0.251066000
H	0.356782000	-2.774472000	-2.020623000
H	1.120006000	-5.086602000	-2.533173000
H	2.544979000	-6.321135000	-0.902559000
H	3.194335000	-5.232186000	1.244755000
H	2.412320000	-2.926561000	1.755294000
H	-0.301621000	-0.918882000	3.253009000
H	-0.498235000	-2.419246000	5.214690000
H	-1.208946000	-4.788511000	4.916612000
H	-1.728972000	-5.647223000	2.634502000
H	-1.523247000	-4.142797000	0.663262000
H	3.588839000	-2.312262000	-2.605484000
H	5.546428000	-3.512716000	-1.632285000
H	5.656328000	-0.686970000	1.614090000
H	3.732009000	0.525963000	0.632803000
H	6.457978000	-3.845837000	1.241371000
H	7.410050000	-2.357064000	1.410923000
H	7.539986000	-3.349929000	-0.068634000
H	-3.164346000	0.705538000	1.162322000
H	-2.984251000	-0.834136000	1.993315000
H	-4.308331000	-2.454032000	1.446070000
H	-5.772137000	-3.129091000	-2.545261000
H	-4.465272000	-1.155649000	-3.329999000
H	-4.728133000	-4.723177000	1.457366000
H	-6.376708000	-5.383317000	1.222116000
H	-6.164635000	-3.732500000	1.878910000
H	-4.367982000	3.239269000	-1.141771000
H	-3.306451000	2.969687000	0.255934000
H	-4.533905000	1.751394000	-0.192271000
H	-1.564270000	4.293788000	-1.122591000
H	-2.324147000	4.509428000	-2.707509000
H	-0.578847000	4.210469000	-2.620261000
H	0.662993000	1.027659000	-4.106474000
H	0.406030000	2.771933000	-3.896168000
H	-0.534368000	1.880957000	-5.118239000
H	-1.860568000	-1.015289000	-4.682001000
H	-1.812363000	-1.887580000	-3.131979000
H	-0.347859000	-1.058707000	-3.739000000
H	-0.995769000	0.335373000	1.532025000
C	0.699483000	3.025777000	0.861578000
N	1.373681000	3.629371000	-0.141214000

H	-0.229340000	1.918859000	0.091537000
C	-0.417405000	3.697871000	1.499077000
C	-0.666211000	5.062619000	1.219938000
C	-1.311893000	2.963686000	2.384078000
C	-1.729700000	5.748590000	1.770258000
H	0.036524000	5.610837000	0.582297000
C	-2.404654000	3.727455000	2.926203000
C	-2.606446000	5.055788000	2.631506000
H	-1.875530000	6.811166000	1.559613000
H	-3.084471000	3.185162000	3.592387000
H	-3.457704000	5.583840000	3.075321000
C	1.513038000	2.000345000	1.596929000
H	2.407087000	2.509301000	1.996752000
H	1.838999000	1.218127000	0.898423000
H	0.941604000	1.551011000	2.415356000
O	-1.200020000	1.731220000	2.645793000
H	0.860265000	4.279281000	-0.729153000
H	2.005090000	3.010428000	-0.663355000

Sum of electronic and thermal Energies= -2751.594393
Sum of electronic and thermal Enthalpies= -2751.593449
Sum of electronic and thermal Free Energies= -2751.728652
Lowest vibration frequency (/cm^-1): -469.8748

TS4

Rh	-0.851508000	1.157962000	-0.757144000
S	1.939853000	0.035937000	-1.966978000
O	2.185148000	1.473233000	-2.175415000
O	1.609427000	-0.706945000	-3.206741000
O	-6.029500000	-3.380884000	0.412898000
N	0.827768000	-0.138690000	-0.810131000
N	-1.171371000	-0.039498000	0.947242000
C	0.842571000	-1.191709000	0.196159000
C	-0.604183000	-1.371835000	0.688119000
C	1.351909000	-2.554090000	-0.221312000
C	0.860843000	-3.178788000	-1.371723000
C	1.260823000	-4.472699000	-1.695843000
C	2.150012000	-5.160693000	-0.869776000
C	2.637549000	-4.546450000	0.282761000
C	2.239424000	-3.250573000	0.601977000
C	-0.698057000	-2.299266000	1.871365000
C	-0.153389000	-1.936863000	3.108595000
C	-0.206737000	-2.818922000	4.185427000
C	-0.803185000	-4.071005000	4.037410000
C	-1.350603000	-4.437084000	2.808802000

C	-1.300090000	-3.552497000	1.733251000
C	3.440131000	-0.697746000	-1.338510000
C	4.061406000	-1.720508000	-2.049783000
C	5.228361000	-2.283788000	-1.545769000
C	5.781110000	-1.844284000	-0.335900000
C	5.144675000	-0.799688000	0.347604000
C	3.980246000	-0.222674000	-0.146618000
C	7.002097000	-2.499939000	0.228196000
C	-2.575079000	-0.041653000	1.383782000
C	-3.552002000	-0.646297000	0.408221000
C	-4.336202000	-1.710056000	0.867294000
C	-5.268418000	-2.338231000	0.036833000
C	-5.430087000	-1.879723000	-1.275771000
C	-4.649002000	-0.833731000	-1.736953000
C	-3.698539000	-0.199398000	-0.921261000
C	-5.846192000	-3.926774000	1.695591000
C	-2.847700000	0.842784000	-1.529950000
C	-2.725427000	2.225836000	-1.120624000
C	-1.728236000	2.834381000	-1.964797000
C	-1.227203000	1.846387000	-2.863574000
C	-1.930833000	0.606237000	-2.607729000
C	-3.619280000	2.933918000	-0.164777000
C	-1.333387000	4.266317000	-1.946645000
C	-0.278090000	2.073257000	-3.981959000
C	-1.722492000	-0.664373000	-3.346402000
H	1.442870000	-0.867298000	1.079783000
H	-1.184255000	-1.808157000	-0.148018000
H	0.173308000	-2.635218000	-2.027426000
H	0.874323000	-4.949393000	-2.601663000
H	2.463464000	-6.177128000	-1.125965000
H	3.336352000	-5.077713000	0.936125000
H	2.623944000	-2.764908000	1.506264000
H	0.324475000	-0.957095000	3.230088000
H	0.224740000	-2.527165000	5.147199000
H	-0.840945000	-4.763504000	4.883282000
H	-1.818751000	-5.418143000	2.685713000
H	-1.725768000	-3.838563000	0.764005000
H	3.614101000	-2.076531000	-2.981369000
H	5.720131000	-3.092633000	-2.097072000
H	5.568455000	-0.430867000	1.288669000
H	3.483539000	0.586307000	0.396933000
H	7.560656000	-1.821291000	0.890205000
H	7.679469000	-2.849740000	-0.565823000
H	6.727238000	-3.385081000	0.828329000

H	-2.829184000	1.010081000	1.603943000
H	-2.656536000	-0.584349000	2.343228000
H	-4.189475000	-2.054553000	1.894251000
H	-6.164188000	-2.372515000	-1.918509000
H	-4.767997000	-0.489900000	-2.769823000
H	-4.808438000	-4.276189000	1.844756000
H	-6.524930000	-4.784601000	1.779791000
H	-6.093443000	-3.200112000	2.489531000
H	-4.306015000	3.605892000	-0.707758000
H	-3.063568000	3.556371000	0.554177000
H	-4.243099000	2.226646000	0.402230000
H	-1.596451000	4.751704000	-0.995610000
H	-1.850400000	4.816686000	-2.752394000
H	-0.251042000	4.389885000	-2.110184000
H	0.359051000	2.949982000	-3.800818000
H	-0.840290000	2.245579000	-4.917386000
H	0.383838000	1.208438000	-4.133081000
H	-2.247693000	-0.642235000	-4.318092000
H	-2.111398000	-1.526066000	-2.781130000
H	-0.650104000	-0.826137000	-3.542330000
H	-0.631126000	0.456170000	1.709313000
C	0.415875000	2.575824000	1.686091000
N	-0.075346000	1.788004000	2.682907000
H	0.061600000	2.082661000	0.458463000
C	1.913634000	2.542699000	1.546707000
C	2.546419000	3.204738000	0.488177000
C	2.719935000	1.942643000	2.551096000
C	3.927398000	3.332841000	0.417566000
H	1.924463000	3.608519000	-0.318198000
C	4.116546000	2.097515000	2.484650000
C	4.711505000	2.784810000	1.436458000
H	4.390592000	3.846628000	-0.428847000
H	4.711865000	1.647057000	3.285246000
H	5.801643000	2.879477000	1.402899000
C	-0.190248000	3.960033000	1.541486000
H	-1.290538000	3.893771000	1.574258000
H	0.101247000	4.463609000	0.608267000
H	0.141007000	4.585774000	2.387946000
O	2.205590000	1.253469000	3.565970000
H	1.183929000	1.336287000	3.447006000
H	-0.976448000	2.143833000	3.011680000

Sum of electronic and thermal Energies= -2751.582988

Sum of electronic and thermal Enthalpies= -2751.582044

Sum of electronic and thermal Free Energies= -2751.718560

Lowest vibration frequency (/cm^-1): -623.5379

IV

Rh	-0.844400000	0.814102000	-1.009478000
S	2.060707000	-0.092583000	-1.809815000
O	2.198694000	1.352679000	-2.050590000
O	1.880403000	-0.915874000	-3.023372000
O	-6.153476000	-3.504115000	-0.170569000
N	0.832970000	-0.295319000	-0.745621000
N	-1.302782000	-0.200756000	0.788825000
C	0.825851000	-1.277356000	0.341599000
C	-0.649447000	-1.515068000	0.715531000
C	1.467341000	-2.613054000	0.053337000
C	1.144194000	-3.325113000	-1.105737000
C	1.669962000	-4.596895000	-1.318987000
C	2.516018000	-5.173971000	-0.371564000
C	2.834303000	-4.472505000	0.790615000
C	2.312363000	-3.198424000	0.998603000
C	-0.784358000	-2.346654000	1.965188000
C	-0.505503000	-1.804862000	3.225754000
C	-0.548395000	-2.615118000	4.358299000
C	-0.867665000	-3.968207000	4.245981000
C	-1.161092000	-4.508883000	2.995300000
C	-1.123671000	-3.698831000	1.862510000
C	3.529664000	-0.648309000	-0.979399000
C	4.295398000	-1.662195000	-1.549064000
C	5.458696000	-2.067561000	-0.906365000
C	5.864991000	-1.473239000	0.295877000
C	5.070311000	-0.455866000	0.841389000
C	3.904033000	-0.029449000	0.213229000
C	7.128741000	-1.909896000	0.967405000
C	-2.738409000	-0.224224000	1.100155000
C	-3.635768000	-0.816607000	0.044926000
C	-4.460574000	-1.879308000	0.427443000
C	-5.348598000	-2.473868000	-0.474409000
C	-5.415800000	-1.990905000	-1.787615000
C	-4.588286000	-0.953662000	-2.177624000
C	-3.690061000	-0.345938000	-1.284710000
C	-6.093946000	-4.060442000	1.120551000
C	-2.784250000	0.686109000	-1.819168000
C	-2.621143000	2.044304000	-1.398803000
C	-1.525254000	2.600787000	-2.160587000
C	-1.037304000	1.623038000	-3.086492000
C	-1.791365000	0.419384000	-2.864548000

C	-3.418053000	2.786349000	-0.387292000
C	-1.008831000	3.972756000	-1.973559000
C	-0.018586000	1.813320000	-4.147939000
C	-1.577653000	-0.858474000	-3.581481000
H	1.321394000	-0.836476000	1.235599000
H	-1.117356000	-2.064862000	-0.125559000
H	0.488648000	-2.868552000	-1.854875000
H	1.416299000	-5.143559000	-2.232092000
H	2.927676000	-6.173455000	-0.540224000
H	3.495358000	-4.919334000	1.539047000
H	2.557610000	-2.645024000	1.912509000
H	-0.281736000	-0.735694000	3.328773000
H	-0.331014000	-2.183941000	5.339858000
H	-0.894859000	-4.601330000	5.137664000
H	-1.419030000	-5.567608000	2.899910000
H	-1.343951000	-4.123138000	0.875794000
H	3.963274000	-2.134303000	-2.477516000
H	6.064724000	-2.870310000	-1.340069000
H	5.372356000	0.009246000	1.785949000
H	3.268644000	0.747960000	0.666267000
H	7.257128000	-3.002173000	0.912088000
H	7.153663000	-1.609611000	2.025285000
H	8.010076000	-1.460877000	0.477908000
H	-3.016646000	0.820511000	1.319231000
H	-2.890822000	-0.771514000	2.047282000
H	-4.388369000	-2.241997000	1.455986000
H	-6.115410000	-2.458561000	-2.484954000
H	-4.632767000	-0.593002000	-3.210785000
H	-5.086347000	-4.451627000	1.348696000
H	-6.810203000	-4.890919000	1.144676000
H	-6.374770000	-3.325667000	1.895133000
H	-3.763289000	3.748231000	-0.800451000
H	-2.826860000	3.019521000	0.517840000
H	-4.305810000	2.212208000	-0.082809000
H	-1.043392000	4.270413000	-0.911812000
H	-1.643057000	4.686393000	-2.529418000
H	0.019809000	4.077342000	-2.347854000
H	0.636212000	2.668869000	-3.936399000
H	-0.518947000	1.996367000	-5.114863000
H	0.619390000	0.923899000	-4.256699000
H	-2.051709000	-0.820789000	-4.578581000
H	-2.012212000	-1.707067000	-3.032412000
H	-0.499753000	-1.038060000	-3.723638000
H	-0.885328000	0.406425000	1.550876000

C	0.963035000	2.813117000	1.084364000
N	1.789665000	1.947497000	1.922352000
H	0.403253000	2.120109000	0.391624000
C	-0.154617000	3.528659000	1.817221000
C	-0.436998000	4.882810000	1.634428000
C	-1.013338000	2.728171000	2.649790000
C	-1.532875000	5.507273000	2.239717000
H	0.220176000	5.483555000	0.994883000
C	-2.119553000	3.389640000	3.249372000
C	-2.372772000	4.742360000	3.050080000
H	-1.722441000	6.572795000	2.078719000
H	-2.775667000	2.779813000	3.882452000
H	-3.240766000	5.206398000	3.532765000
C	1.863807000	3.686622000	0.242385000
H	1.312683000	4.261427000	-0.516174000
H	2.593979000	3.054977000	-0.284807000
H	2.408071000	4.409126000	0.877661000
O	-0.805211000	1.464927000	2.830731000
H	1.154097000	1.494985000	2.581471000
H	2.410116000	2.536970000	2.482336000

Sum of electronic and thermal Energies= -2751.602035
Sum of electronic and thermal Enthalpies= -2751.601091
Sum of electronic and thermal Free Energies= -2751.741389

Computed structures using catalyst *(R,R)*-**C**:

III-F

Rh	-0.946055000	0.869356000	-0.497339000
S	2.270373000	0.585325000	-0.048080000
O	2.764188000	0.837566000	1.334453000
O	2.180635000	1.754158000	-0.931812000
F	-5.665139000	-4.255054000	-0.551062000
N	0.863049000	-0.222649000	-0.080502000
N	-1.597261000	-0.369989000	1.115452000
C	0.682403000	-1.187815000	1.005418000
C	-0.798117000	-1.602330000	1.027452000
C	1.551699000	-2.421446000	0.908895000
C	1.610143000	-3.156710000	-0.278622000
C	2.342322000	-4.338535000	-0.342060000
C	3.026261000	-4.800203000	0.783885000
C	2.977980000	-4.069272000	1.969376000
C	2.246601000	-2.884007000	2.027522000
C	-1.079028000	-2.603307000	2.120651000
C	-1.146958000	-2.207181000	3.461150000

C	-1.295736000	-3.158865000	4.468088000
C	-1.377457000	-4.513515000	4.148916000
C	-1.331386000	-4.913145000	2.814064000
C	-1.189772000	-3.961074000	1.807492000
C	3.476374000	-0.506595000	-0.784270000
C	3.353928000	-0.808011000	-2.139535000
C	4.234978000	-1.711051000	-2.719960000
C	5.242473000	-2.324787000	-1.962097000
C	5.363006000	-1.979514000	-0.611589000
C	4.486019000	-1.076829000	-0.016940000
C	6.139411000	-3.357230000	-2.568407000
C	-3.055241000	-0.571326000	1.124399000
C	-3.626553000	-1.295942000	-0.070370000
C	-4.376012000	-2.446745000	0.192049000
C	-4.958818000	-3.164305000	-0.841826000
C	-4.817892000	-2.775794000	-2.163919000
C	-4.066264000	-1.637382000	-2.432822000
C	-3.462991000	-0.882962000	-1.417025000
C	-2.635555000	0.272588000	-1.818167000
C	-2.786226000	1.646620000	-1.333694000
C	-1.708362000	2.413145000	-1.884694000
C	-0.869649000	1.525380000	-2.629278000
C	-1.481881000	0.204405000	-2.632829000
C	-3.969780000	2.211352000	-0.623373000
C	-1.527526000	3.881145000	-1.718821000
C	0.318630000	1.906338000	-3.434497000
C	-0.883856000	-0.996836000	-3.275220000
H	0.881795000	-0.717721000	1.995439000
H	-1.029741000	-2.070729000	0.050873000
H	1.077381000	-2.788418000	-1.162861000
H	2.383972000	-4.904074000	-1.278391000
H	3.600548000	-5.730192000	0.734328000
H	3.514968000	-4.422520000	2.854675000
H	2.205828000	-2.306641000	2.958703000
H	-1.103132000	-1.144076000	3.726126000
H	-1.347751000	-2.837739000	5.512489000
H	-1.487045000	-5.258917000	4.942004000
H	-1.406617000	-5.973003000	2.554296000
H	-1.147820000	-4.273829000	0.757767000
H	2.559401000	-0.343137000	-2.732053000
H	4.138607000	-1.954907000	-3.783286000
H	6.153462000	-2.440192000	-0.009059000
H	4.567974000	-0.819636000	1.042179000
H	5.735712000	-4.370468000	-2.393504000

H	7.146265000	-3.333736000	-2.124259000
H	6.234545000	-3.229538000	-3.657182000
H	-3.501024000	0.431939000	1.234687000
H	-3.338588000	-1.131098000	2.034051000
H	-4.505970000	-2.805892000	1.217675000
H	-5.289365000	-3.359309000	-2.958185000
H	-3.941231000	-1.310080000	-3.469864000
H	-4.533863000	2.888778000	-1.288250000
H	-3.682939000	2.792792000	0.269642000
H	-4.669005000	1.422754000	-0.305859000
H	-1.946518000	4.228882000	-0.760652000
H	-2.035243000	4.438886000	-2.525739000
H	-0.462525000	4.160408000	-1.743095000
H	1.087120000	1.119368000	-3.418615000
H	0.788607000	2.825501000	-3.058303000
H	0.033629000	2.070172000	-4.489392000
H	-1.049153000	-1.005369000	-4.367222000
H	-1.306290000	-1.928185000	-2.866418000
H	0.207362000	-1.024649000	-3.113092000
H	-1.381806000	0.128628000	1.994158000
C	0.252197000	3.036393000	2.551362000
N	0.786903000	1.933660000	3.018155000
H	-0.462717000	1.890247000	0.592163000
C	-1.138715000	3.315252000	2.762801000
C	-1.679849000	4.564324000	2.368336000
C	-2.017500000	2.273644000	3.265140000
C	-3.027668000	4.832910000	2.451087000
H	-1.006041000	5.341301000	1.993905000
C	-3.413712000	2.594956000	3.313042000
C	-3.894361000	3.825139000	2.930475000
H	-3.420985000	5.807734000	2.152506000
H	-4.082349000	1.810348000	3.683081000
H	-4.969100000	4.026397000	2.995694000
C	1.135229000	3.948842000	1.771765000
H	0.654784000	4.244268000	0.827014000
H	2.087730000	3.453618000	1.537072000
H	1.341042000	4.868129000	2.343668000
O	-1.603387000	1.118629000	3.609856000
H	0.122947000	1.291764000	3.482980000
H	1.691954000	1.610854000	2.650489000

Sum of electronic and thermal Energies= -2736.421862
Sum of electronic and thermal Enthalpies= -2736.420917
Sum of electronic and thermal Free Energies= -2736.555348

TS1-F

Rh	-0.820976000	1.020283000	-1.141735000
S	2.233003000	0.042549000	-1.715038000
O	2.559646000	1.489206000	-1.676064000
O	2.144191000	-0.516338000	-3.080942000
F	-5.980277000	-3.468648000	0.269668000
N	0.902673000	-0.228345000	-0.853017000
N	-1.263947000	-0.044948000	0.667425000
C	0.793620000	-1.248053000	0.182839000
C	-0.704465000	-1.397022000	0.500803000
C	1.333824000	-2.622241000	-0.148194000
C	1.036810000	-3.224041000	-1.373491000
C	1.439914000	-4.531116000	-1.635109000
C	2.144420000	-5.253009000	-0.671268000
C	2.446042000	-4.657804000	0.553002000
C	2.042046000	-3.349872000	0.810563000
C	-0.948306000	-2.313826000	1.672853000
C	-0.670519000	-1.902385000	2.981785000
C	-0.830227000	-2.793328000	4.041626000
C	-1.263973000	-4.097763000	3.807011000
C	-1.552575000	-4.508284000	2.506257000
C	-1.399871000	-3.617154000	1.447109000
C	3.579756000	-0.807190000	-0.902857000
C	4.215747000	-1.856451000	-1.560812000
C	5.242475000	-2.539344000	-0.919715000
C	5.643725000	-2.194205000	0.376714000
C	5.009311000	-1.114595000	1.004683000
C	3.984424000	-0.419090000	0.373246000
C	6.698180000	-2.984309000	1.084173000
C	-2.696339000	-0.015179000	0.999855000
C	-3.570310000	-0.776647000	0.033669000
C	-4.396369000	-1.771078000	0.562319000
C	-5.206388000	-2.530872000	-0.269059000
C	-5.219072000	-2.344660000	-1.642248000
C	-4.394476000	-1.361070000	-2.175680000
C	-3.572170000	-0.565575000	-1.366762000
C	-2.725590000	0.443791000	-2.029761000
C	-2.731643000	1.869461000	-1.745082000
C	-1.741398000	2.474705000	-2.587219000
C	-1.073506000	1.445916000	-3.319894000
C	-1.719777000	0.182979000	-3.010794000
C	-3.709050000	2.612698000	-0.902097000
C	-1.547022000	3.937490000	-2.734329000
C	-0.013128000	1.638355000	-4.341780000

C	-1.342840000	-1.121533000	-3.610638000
H	1.278573000	-0.912794000	1.129076000
H	-1.183289000	-1.841617000	-0.392065000
H	0.494819000	-2.648904000	-2.130201000
H	1.202303000	-4.990526000	-2.599235000
H	2.461227000	-6.279878000	-0.876315000
H	3.001384000	-5.215216000	1.313610000
H	2.274465000	-2.883544000	1.775121000
H	-0.339750000	-0.872748000	3.172571000
H	-0.612516000	-2.464508000	5.062120000
H	-1.383335000	-4.794954000	4.641544000
H	-1.899439000	-5.527841000	2.315034000
H	-1.621547000	-3.937826000	0.421988000
H	3.886978000	-2.137495000	-2.564374000
H	5.739324000	-3.370693000	-1.431258000
H	5.322355000	-0.817172000	2.010783000
H	3.496455000	0.412641000	0.888350000
H	6.255574000	-3.873042000	1.567602000
H	7.190011000	-2.396438000	1.873325000
H	7.468028000	-3.349800000	0.387635000
H	-2.985989000	1.047407000	1.068827000
H	-2.849493000	-0.435689000	2.009972000
H	-4.403568000	-1.979243000	1.636669000
H	-5.864337000	-2.962475000	-2.270936000
H	-4.390848000	-1.191720000	-3.257056000
H	-4.308259000	3.301375000	-1.521974000
H	-3.209305000	3.221439000	-0.127379000
H	-4.414029000	1.932373000	-0.401995000
H	-1.795042000	4.478173000	-1.807604000
H	-2.225486000	4.318407000	-3.518512000
H	-0.522015000	4.197534000	-3.035450000
H	0.761572000	0.860310000	-4.263333000
H	0.481161000	2.614693000	-4.227922000
H	-0.442719000	1.595439000	-5.358374000
H	-1.759594000	-1.220174000	-4.628715000
H	-1.718122000	-1.968613000	-3.015497000
H	-0.246205000	-1.202034000	-3.690893000
H	-0.743079000	0.430568000	1.432857000
C	0.689372000	3.112350000	0.661389000
N	1.769380000	2.400991000	1.073378000
H	-0.063373000	2.185423000	-0.188347000
C	-0.387887000	3.366099000	1.636501000
C	-1.212995000	4.498822000	1.517008000
C	-0.652628000	2.390090000	2.666298000

C	-2.281023000	4.722892000	2.373401000
H	-1.002149000	5.237024000	0.736146000
C	-1.764510000	2.652414000	3.520010000
C	-2.551442000	3.779816000	3.377806000
H	-2.896413000	5.620324000	2.268047000
H	-1.974273000	1.907553000	4.295952000
H	-3.395643000	3.938059000	4.057851000
C	1.012544000	4.144029000	-0.382110000
H	0.117661000	4.537182000	-0.874759000
H	1.671913000	3.704658000	-1.145333000
H	1.537788000	4.984978000	0.102149000
O	0.033114000	1.308324000	2.777852000
H	1.538128000	1.711193000	1.800456000
H	2.391326000	2.122834000	0.310435000

Sum of electronic and thermal Energies= -2736.396075

Sum of electronic and thermal Enthalpies= -2736.395131

Sum of electronic and thermal Free Energies= -2736.527972

Lowest vibration frequency (/cm^-1) = -368.6201

TS2-F

Rh	-0.783297000	1.145205000	-0.704669000
S	2.005753000	0.077012000	-1.908580000
O	2.311643000	1.520760000	-1.872868000
O	1.719045000	-0.452298000	-3.262213000
F	-6.049558000	-3.275058000	0.548416000
N	0.804940000	-0.247844000	-0.877045000
N	-1.131665000	-0.123762000	0.989456000
C	0.831959000	-1.283335000	0.143654000
C	-0.624962000	-1.455814000	0.619546000
C	1.353201000	-2.637209000	-0.279563000
C	0.917887000	-3.230301000	-1.467524000
C	1.312468000	-4.525639000	-1.793551000
C	2.144759000	-5.242676000	-0.933587000
C	2.585408000	-4.654476000	0.251617000
C	2.191116000	-3.358286000	0.573897000
C	-0.759416000	-2.501980000	1.697200000
C	-0.351248000	-2.251482000	3.012019000
C	-0.374338000	-3.274448000	3.958664000
C	-0.806913000	-4.552172000	3.606093000
C	-1.239551000	-4.800541000	2.303970000
C	-1.220119000	-3.778178000	1.358931000
C	3.451611000	-0.789789000	-1.330647000
C	4.004065000	-1.824531000	-2.080594000
C	5.117010000	-2.494964000	-1.586110000

C	5.683823000	-2.150114000	-0.351788000
C	5.122571000	-1.086159000	0.365596000
C	4.011616000	-0.404762000	-0.117537000
C	6.836951000	-2.927992000	0.198672000
C	-2.528999000	-0.103663000	1.443885000
C	-3.522609000	-0.682533000	0.469319000
C	-4.348364000	-1.712822000	0.926145000
C	-5.279104000	-2.297232000	0.079749000
C	-5.417711000	-1.894695000	-1.239158000
C	-4.592235000	-0.876486000	-1.701575000
C	-3.647238000	-0.255557000	-0.873972000
C	-2.801926000	0.797030000	-1.467664000
C	-2.698996000	2.172442000	-1.007290000
C	-1.753048000	2.831452000	-1.856460000
C	-1.221368000	1.877105000	-2.778993000
C	-1.911071000	0.619001000	-2.567533000
C	-3.583893000	2.837929000	-0.011414000
C	-1.480501000	4.289540000	-1.858181000
C	-0.276107000	2.160436000	-3.889157000
C	-1.665487000	-0.622393000	-3.344413000
H	1.417595000	-0.959723000	1.037377000
H	-1.206273000	-1.790473000	-0.260883000
H	0.272070000	-2.661190000	-2.144425000
H	0.966828000	-4.980748000	-2.726639000
H	2.453822000	-6.260395000	-1.190045000
H	3.242984000	-5.208076000	0.929011000
H	2.532645000	-2.892030000	1.506357000
H	-0.030390000	-1.241703000	3.300182000
H	-0.050015000	-3.069438000	4.983509000
H	-0.816859000	-5.353955000	4.350339000
H	-1.593462000	-5.796469000	2.022152000
H	-1.551540000	-3.973570000	0.332312000
H	3.547393000	-2.103921000	-3.033488000
H	5.554191000	-3.315883000	-2.164966000
H	5.561676000	-0.788055000	1.324583000
H	3.574904000	0.422190000	0.449399000
H	7.519914000	-3.261682000	-0.597649000
H	6.480134000	-3.836537000	0.715243000
H	7.412929000	-2.342914000	0.931102000
H	-2.762952000	0.947353000	1.686883000
H	-2.611061000	-0.661048000	2.394505000
H	-4.263708000	-2.084204000	1.951948000
H	-6.157680000	-2.376898000	-1.882042000
H	-4.682844000	-0.540911000	-2.739485000

H	-4.222359000	3.593415000	-0.500496000
H	-3.014653000	3.358393000	0.776288000
H	-4.254520000	2.117178000	0.479784000
H	-1.658594000	4.746751000	-0.872611000
H	-2.163499000	4.790183000	-2.567645000
H	-0.454289000	4.522762000	-2.178653000
H	0.325254000	3.058760000	-3.686377000
H	-0.831571000	2.329979000	-4.829046000
H	0.419933000	1.323123000	-4.049098000
H	-2.147986000	-0.565216000	-4.336292000
H	-2.067943000	-1.508722000	-2.829514000
H	-0.583756000	-0.766515000	-3.502026000
H	-0.573277000	0.268335000	1.776651000
C	0.773121000	3.039386000	1.366160000
N	-0.315397000	3.360210000	2.103170000
H	0.259876000	2.017055000	0.304829000
C	1.783506000	2.198201000	2.024475000
C	3.152561000	2.435713000	1.804362000
C	1.368172000	1.194468000	2.973964000
C	4.133798000	1.729525000	2.482119000
H	3.458144000	3.207827000	1.092544000
C	2.409347000	0.444412000	3.599764000
C	3.745091000	0.716921000	3.373750000
H	5.191299000	1.947454000	2.308675000
H	2.106260000	-0.335131000	4.307555000
H	4.509864000	0.135306000	3.900780000
C	1.224581000	4.069259000	0.375409000
H	0.360737000	4.580792000	-0.068232000
H	1.815144000	3.607476000	-0.428807000
H	1.836913000	4.829701000	0.889015000
O	0.130853000	0.965209000	3.228172000
H	-1.022557000	3.959290000	1.690183000
H	-0.599508000	2.639609000	2.777736000

Sum of electronic and thermal Energies= -2736.392723
Sum of electronic and thermal Enthalpies= -2736.391779
Sum of electronic and thermal Free Energies= -2736.523868
Lowest vibration frequency (/cm^-1) = -522.1441

TS3-F

Rh	-0.930885000	0.876750000	-0.988966000
S	2.094314000	-0.035130000	-1.726243000
O	2.398353000	1.416442000	-1.678442000
O	1.934865000	-0.563801000	-3.097783000
F	-5.935029000	-3.874186000	-0.203273000

N	0.813858000	-0.353382000	-0.803557000
N	-1.393953000	-0.270194000	0.735955000
C	0.768732000	-1.358679000	0.246867000
C	-0.713095000	-1.566181000	0.629576000
C	1.330477000	-2.720199000	-0.101540000
C	0.971730000	-3.341692000	-1.301552000
C	1.404973000	-4.634734000	-1.582379000
C	2.200826000	-5.321882000	-0.664714000
C	2.561563000	-4.707526000	0.533492000
C	2.125905000	-3.413786000	0.811880000
C	-0.845925000	-2.407431000	1.873975000
C	-0.576080000	-1.858650000	3.133038000
C	-0.625458000	-2.656212000	4.273688000
C	-0.944557000	-4.010002000	4.168731000
C	-1.227188000	-4.560164000	2.919700000
C	-1.182567000	-3.760030000	1.780043000
C	3.515916000	-0.871025000	-1.033594000
C	4.028864000	-1.988389000	-1.689529000
C	5.111258000	-2.663615000	-1.140739000
C	5.695378000	-2.241838000	0.060522000
C	5.188358000	-1.092277000	0.678555000
C	4.109356000	-0.400851000	0.136606000
C	6.814115000	-3.021209000	0.675159000
C	-2.841763000	-0.372408000	0.982147000
C	-3.642697000	-1.070510000	-0.087736000
C	-4.422253000	-2.161598000	0.306400000
C	-5.205310000	-2.840400000	-0.615722000
C	-5.239705000	-2.472753000	-1.951221000
C	-4.458640000	-1.394783000	-2.351770000
C	-3.659191000	-0.680331000	-1.449420000
C	-2.829458000	0.416455000	-1.983795000
C	-2.821647000	1.797844000	-1.517783000
C	-1.806986000	2.490060000	-2.259073000
C	-1.150125000	1.551733000	-3.113066000
C	-1.818585000	0.267933000	-2.974906000
C	-3.805353000	2.451601000	-0.609938000
C	-1.541207000	3.945891000	-2.150981000
C	-0.085333000	1.852017000	-4.104325000
C	-1.424739000	-0.968557000	-3.698946000
H	1.288255000	-1.004739000	1.168425000
H	-1.188602000	-2.100160000	-0.216084000
H	0.358048000	-2.793537000	-2.025478000
H	1.120161000	-5.111030000	-2.525284000
H	2.541909000	-6.337554000	-0.885972000

H	3.187942000	-5.238505000	1.256934000
H	2.404685000	-2.930380000	1.755854000
H	-0.336718000	-0.791959000	3.229678000
H	-0.413630000	-2.216747000	5.252630000
H	-0.979142000	-4.636122000	5.064983000
H	-1.484370000	-5.619560000	2.831173000
H	-1.395561000	-4.191970000	0.795063000
H	3.562521000	-2.328341000	-2.617365000
H	5.508798000	-3.549001000	-1.648666000
H	5.651804000	-0.729546000	1.601922000
H	3.738924000	0.504222000	0.625131000
H	6.421729000	-3.900601000	1.215510000
H	7.384002000	-2.419853000	1.398722000
H	7.510014000	-3.402182000	-0.088197000
H	-3.193729000	0.659083000	1.142887000
H	-3.007979000	-0.897418000	1.940290000
H	-4.420109000	-2.502341000	1.346371000
H	-5.866255000	-3.026579000	-2.654188000
H	-4.469136000	-1.085419000	-3.401675000
H	-4.371241000	3.229627000	-1.150787000
H	-3.316000000	2.946501000	0.249013000
H	-4.539790000	1.732066000	-0.218116000
H	-1.559012000	4.285442000	-1.100413000
H	-2.321806000	4.519640000	-2.680846000
H	-0.575876000	4.223268000	-2.601114000
H	0.670535000	1.053299000	-4.130425000
H	0.432584000	2.793773000	-3.870390000
H	-0.517914000	1.948057000	-5.116099000
H	-1.814928000	-0.970077000	-4.732467000
H	-1.810139000	-1.870432000	-3.197407000
H	-0.326158000	-1.042095000	-3.753961000
H	-1.035702000	0.325630000	1.508822000
C	0.676625000	2.995635000	0.875683000
N	1.357242000	3.615947000	-0.112637000
H	-0.225201000	1.892058000	0.087961000
C	-0.445766000	3.659305000	1.514719000
C	-0.692134000	5.027755000	1.252348000
C	-1.343625000	2.916300000	2.387509000
C	-1.757594000	5.707565000	1.806639000
H	0.013985000	5.583916000	0.625594000
C	-2.437927000	3.672994000	2.934795000
C	-2.638193000	5.005025000	2.655566000
H	-1.901550000	6.772810000	1.608705000
H	-3.120027000	3.122777000	3.592009000

H	-3.490866000	5.527991000	3.102565000
C	1.491361000	1.969650000	1.609841000
H	2.361127000	2.488311000	2.048754000
H	1.856485000	1.213081000	0.903177000
H	0.907379000	1.489205000	2.401403000
O	-1.233048000	1.680085000	2.635866000
H	0.847723000	4.273734000	-0.695069000
H	1.996455000	3.007224000	-0.636879000

Sum of electronic and thermal Energies= -2736.390838
Sum of electronic and thermal Enthalpies= -2736.389893
Sum of electronic and thermal Free Energies= -2736.523102
Lowest vibration frequency (/cm^-1) = -469.5589

IV-F

Rh	-0.942602000	0.771256000	-1.000766000
S	2.006665000	-0.021654000	-1.750255000
O	2.110775000	1.423052000	-1.997756000
O	1.831834000	-0.863710000	-2.949815000
F	-6.353963000	-3.301363000	0.006100000
N	0.774128000	-0.232523000	-0.677435000
N	-1.381859000	-0.230626000	0.789102000
C	0.758086000	-1.266274000	0.368840000
C	-0.723161000	-1.543767000	0.696281000
C	1.433113000	-2.579074000	0.058172000
C	1.124640000	-3.288109000	-1.106953000
C	1.677587000	-4.546315000	-1.330906000
C	2.535097000	-5.113597000	-0.387798000
C	2.838421000	-4.415832000	0.780382000
C	2.289824000	-3.154916000	0.998912000
C	-0.879045000	-2.408880000	1.920508000
C	-0.621346000	-1.900477000	3.200118000
C	-0.689595000	-2.741126000	4.308966000
C	-1.010367000	-4.089831000	4.154215000
C	-1.279162000	-4.596563000	2.884099000
C	-1.218512000	-3.756570000	1.774192000
C	3.485154000	-0.546665000	-0.916792000
C	4.249009000	-1.569667000	-1.472516000
C	5.414259000	-1.968431000	-0.828041000
C	5.824729000	-1.360174000	0.365190000
C	5.035483000	-0.329589000	0.894252000
C	3.871288000	0.092775000	0.261678000
C	7.087215000	-1.784265000	1.046295000
C	-2.807646000	-0.232982000	1.127090000
C	-3.744860000	-0.793928000	0.085138000

C	-4.615623000	-1.810949000	0.482546000
C	-5.542746000	-2.334300000	-0.407664000
C	-5.638437000	-1.879418000	-1.713418000
C	-4.762857000	-0.880674000	-2.121440000
C	-3.817928000	-0.324533000	-1.249312000
C	-2.889441000	0.678111000	-1.809088000
C	-2.699825000	2.035277000	-1.407642000
C	-1.591830000	2.562671000	-2.178245000
C	-1.129529000	1.567024000	-3.090180000
C	-1.904700000	0.377050000	-2.848837000
C	-3.508669000	2.806115000	-0.430560000
C	-1.035318000	3.922314000	-2.012059000
C	-0.111127000	1.725835000	-4.156748000
C	-1.713169000	-0.919443000	-3.538049000
H	1.216793000	-0.848732000	1.291475000
H	-1.161615000	-2.078059000	-0.170223000
H	0.457688000	-2.840992000	-1.851451000
H	1.435339000	-5.090786000	-2.248350000
H	2.966825000	-6.103164000	-0.564396000
H	3.508621000	-4.854822000	1.525232000
H	2.524571000	-2.603217000	1.916514000
H	-0.381987000	-0.836289000	3.324219000
H	-0.488299000	-2.338143000	5.305936000
H	-1.056825000	-4.746597000	5.027849000
H	-1.538158000	-5.651452000	2.755648000
H	-1.422993000	-4.153607000	0.773027000
H	3.915373000	-2.054428000	-2.393666000
H	6.016106000	-2.778803000	-1.252934000
H	5.337132000	0.157601000	1.828182000
H	3.281099000	0.900749000	0.717868000
H	7.311866000	-2.845376000	0.860035000
H	7.034493000	-1.623279000	2.133581000
H	7.948440000	-1.201951000	0.675528000
H	-3.068040000	0.816523000	1.353683000
H	-2.960076000	-0.788225000	2.069991000
H	-4.577649000	-2.212466000	1.499674000
H	-6.379657000	-2.311196000	-2.389500000
H	-4.812806000	-0.513093000	-3.151540000
H	-3.979078000	3.669694000	-0.930194000
H	-2.898896000	3.203549000	0.399438000
H	-4.315093000	2.189945000	-0.005831000
H	-1.129942000	4.269171000	-0.970262000
H	-1.585082000	4.635834000	-2.651498000
H	0.025418000	3.959861000	-2.302618000

H	0.544305000	2.586251000	-3.968181000
H	-0.611994000	1.881749000	-5.128077000
H	0.527057000	0.834086000	-4.240431000
H	-2.221211000	-0.912580000	-4.518916000
H	-2.126368000	-1.753495000	-2.950616000
H	-0.640507000	-1.101466000	-3.713458000
H	-0.876879000	0.346667000	1.534335000
C	1.507588000	2.952026000	1.150738000
N	2.402689000	2.416774000	2.184881000
H	1.290602000	2.100451000	0.473315000
C	0.178371000	3.420574000	1.708077000
C	-0.313931000	4.714029000	1.536851000
C	-0.593691000	2.463708000	2.452252000
C	-1.548961000	5.121141000	2.055488000
H	0.280243000	5.440346000	0.970026000
C	-1.841379000	2.903205000	2.970345000
C	-2.307765000	4.200045000	2.778020000
H	-1.904768000	6.144329000	1.900670000
H	-2.426558000	2.175147000	3.546439000
H	-3.276651000	4.495001000	3.197350000
C	2.257817000	3.983934000	0.337348000
H	1.658584000	4.366934000	-0.504249000
H	3.171761000	3.532504000	-0.079372000
H	2.556561000	4.847871000	0.959060000
O	-0.174184000	1.248359000	2.630052000
H	1.837564000	1.750777000	2.721858000
H	2.630055000	3.174727000	2.833581000

Sum of electronic and thermal Energies= -2736.400777

Sum of electronic and thermal Enthalpies= -2736.399833

Sum of electronic and thermal Free Energies= -2736.534983

VI. Spectral Data for Compounds.

VI.1 ^{1}H, ^{13}C and ^{19}F NMR spectra for compounds 3a – 3ad

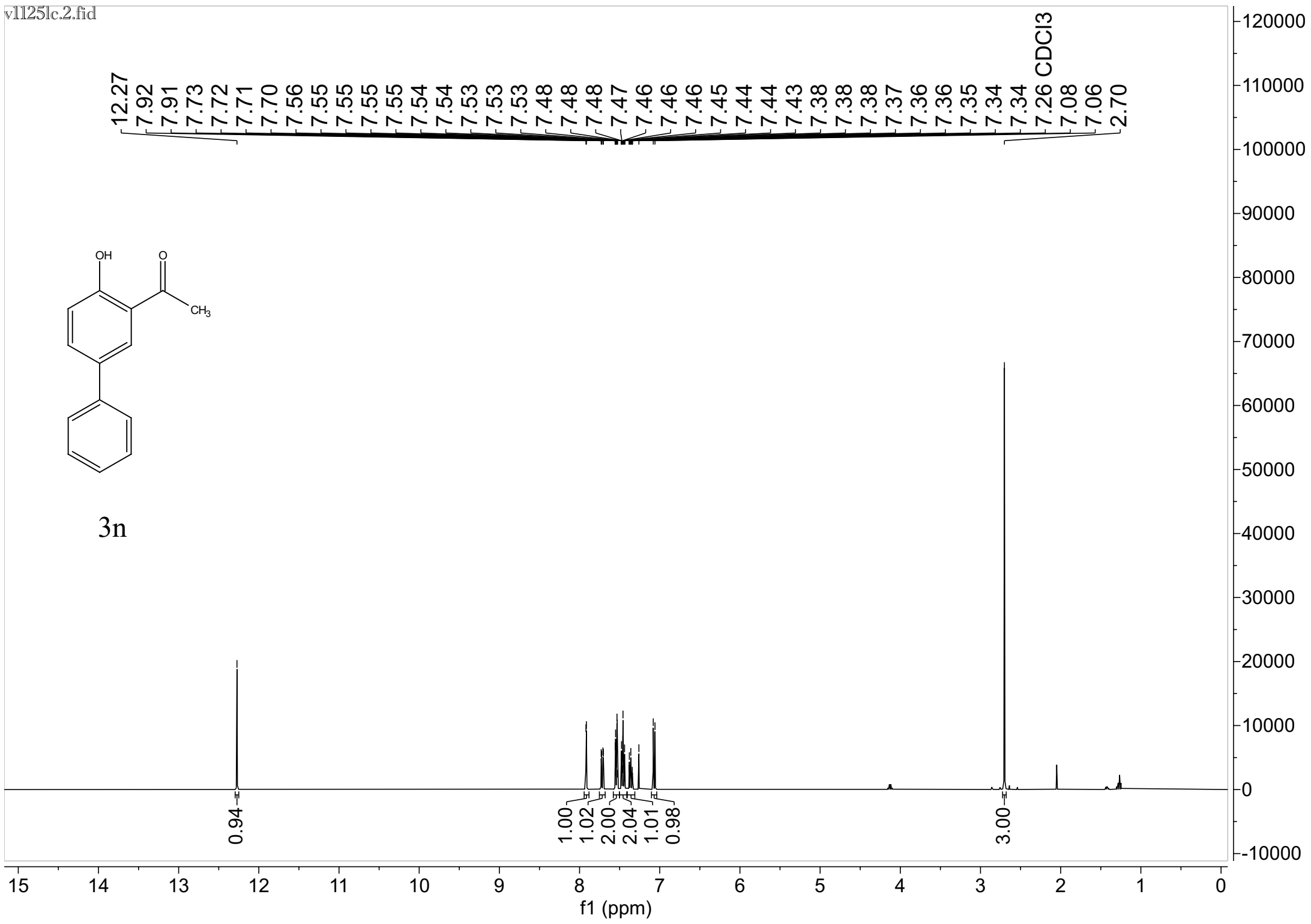

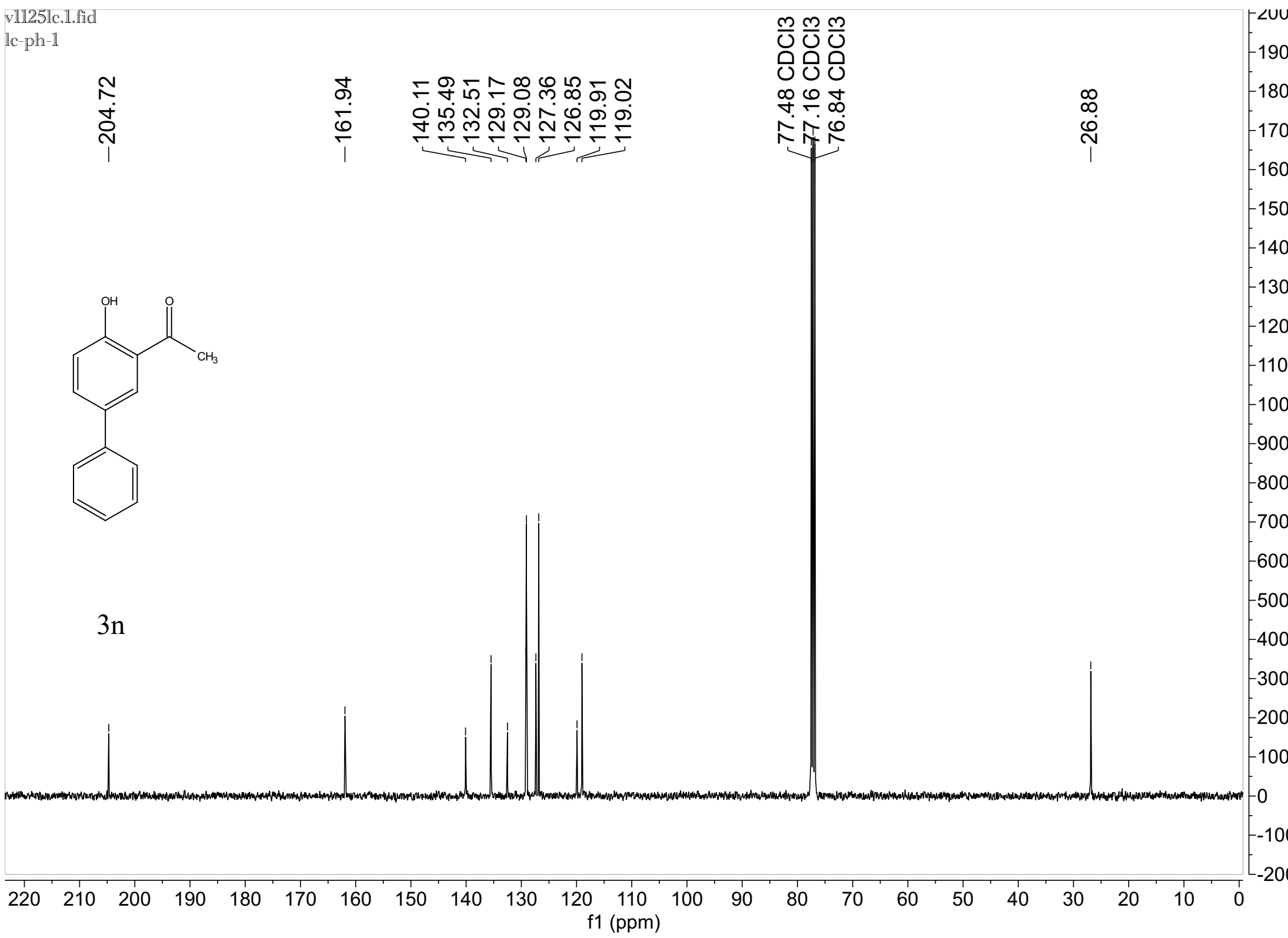

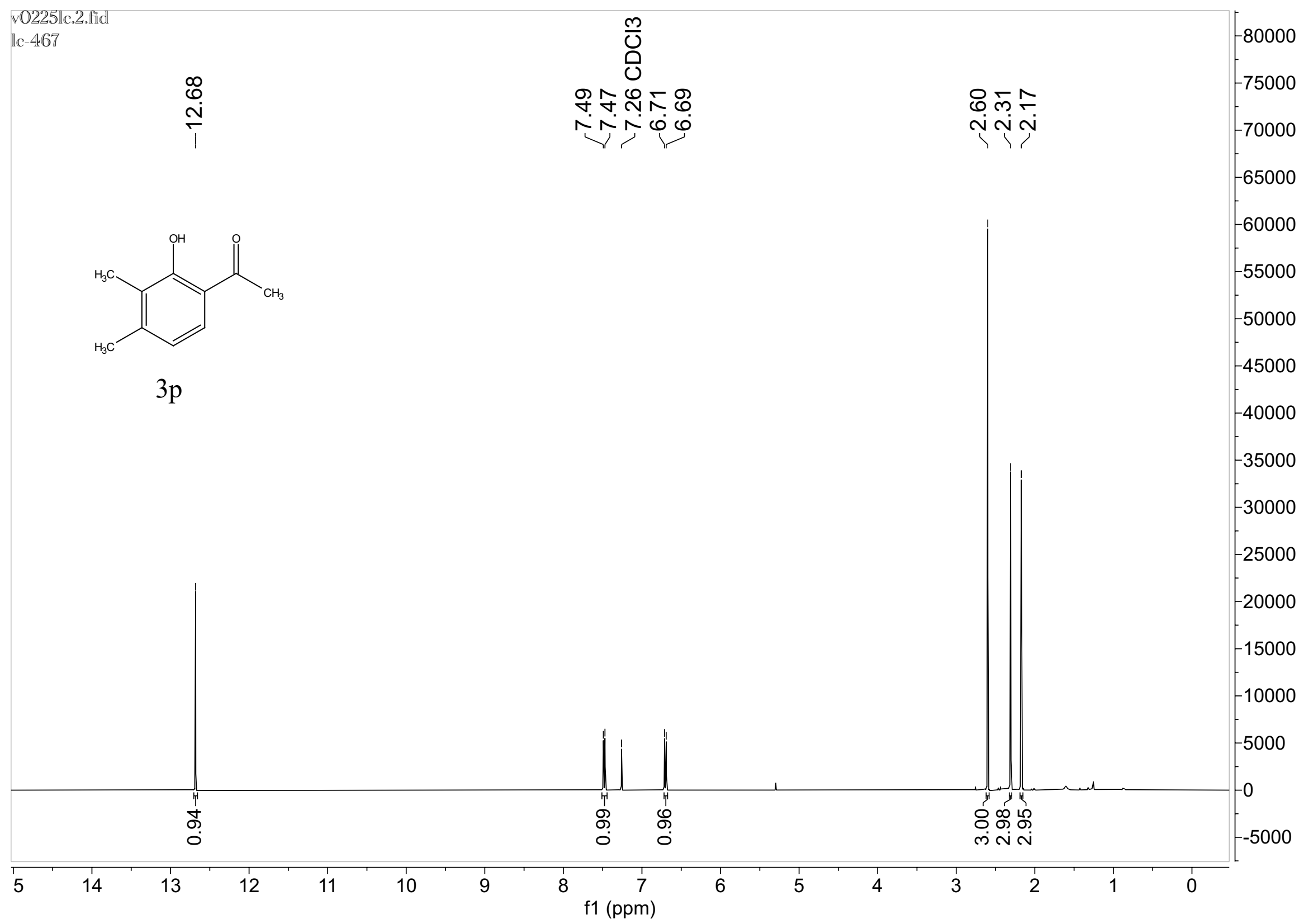
v0225lc.2.fid
lc-467
3p
f1 (ppm)

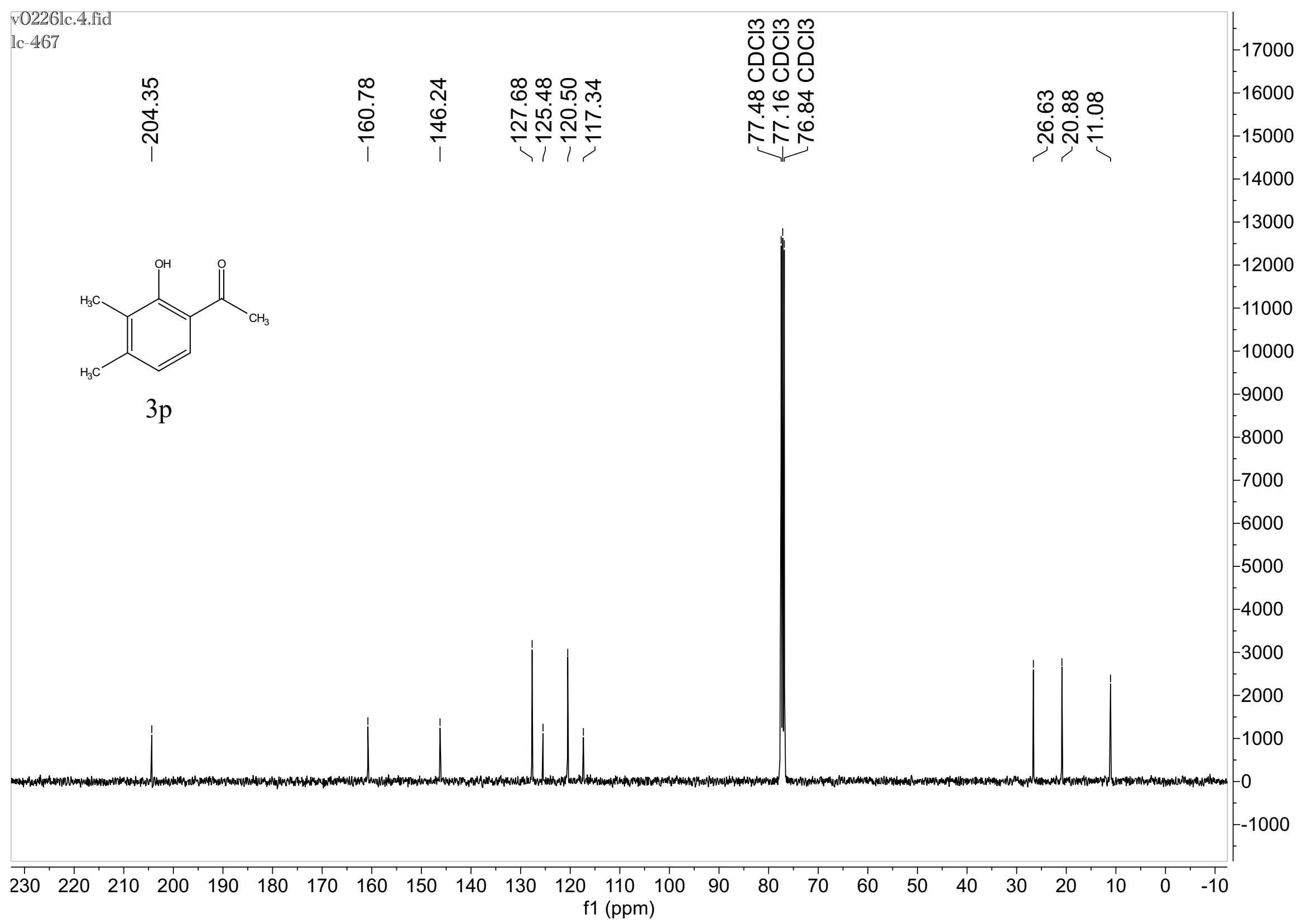
v0226lc.4.fid
lc-467
3p
f1 (ppm)

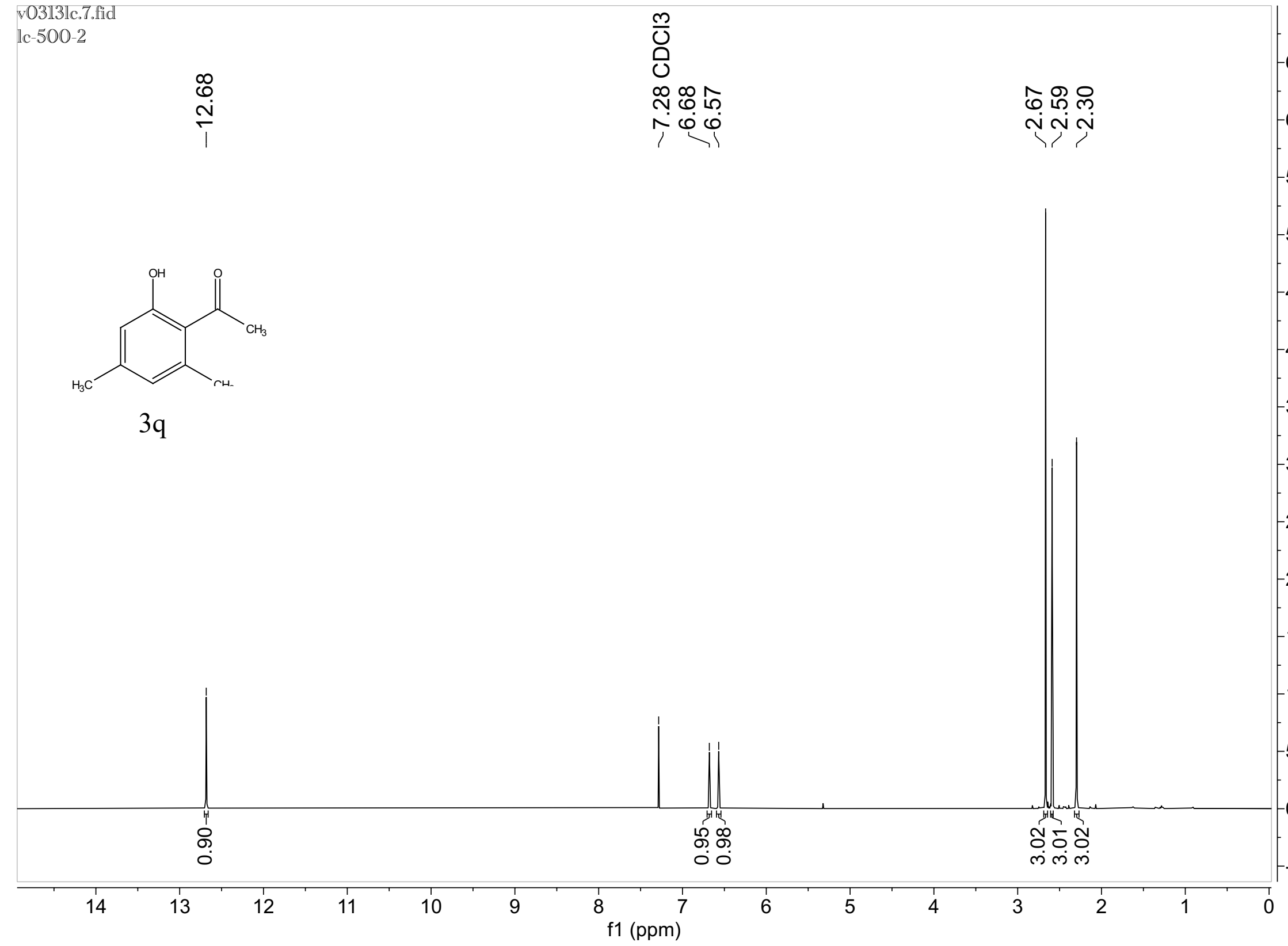

v0313lc.7.fid
lc-500-2
12.68
7.28 CDCl3
6.68
6.57
2.67
2.59
2.30
OH
O
CH3
H3C
3q
0.90
0.95
0.98
3.02
3.01
3.02
f1 (ppm)

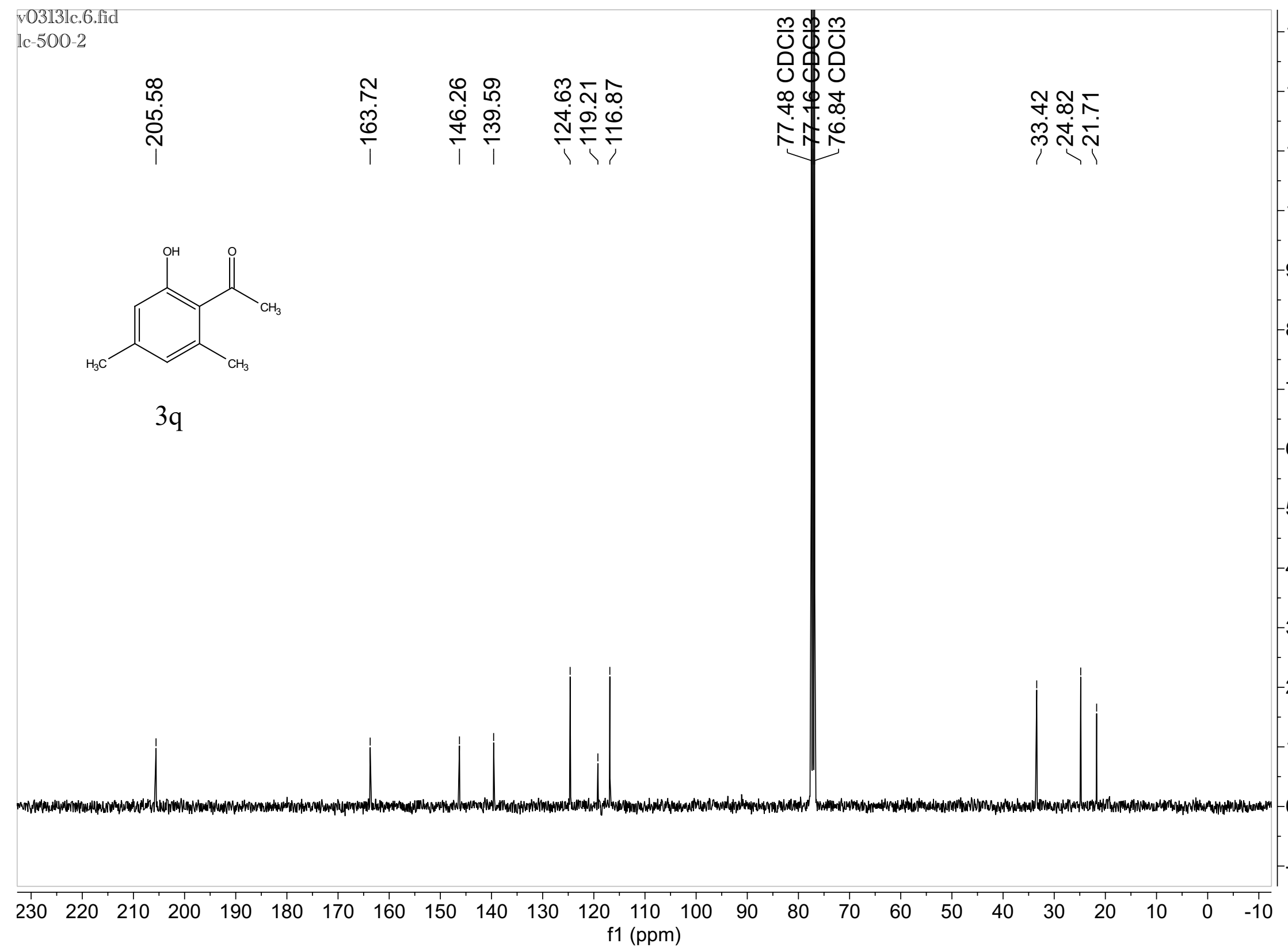

v0313lc.6.fid
lc-500-2
205.58
163.72
146.26
139.59
124.63
119.21
116.87
77.48 CDCl3
77.16 CDCl3
76.84 CDCl3
33.42
24.82
21.71
OH
O
CH3
H3C
CH3
3q
f1 (ppm)

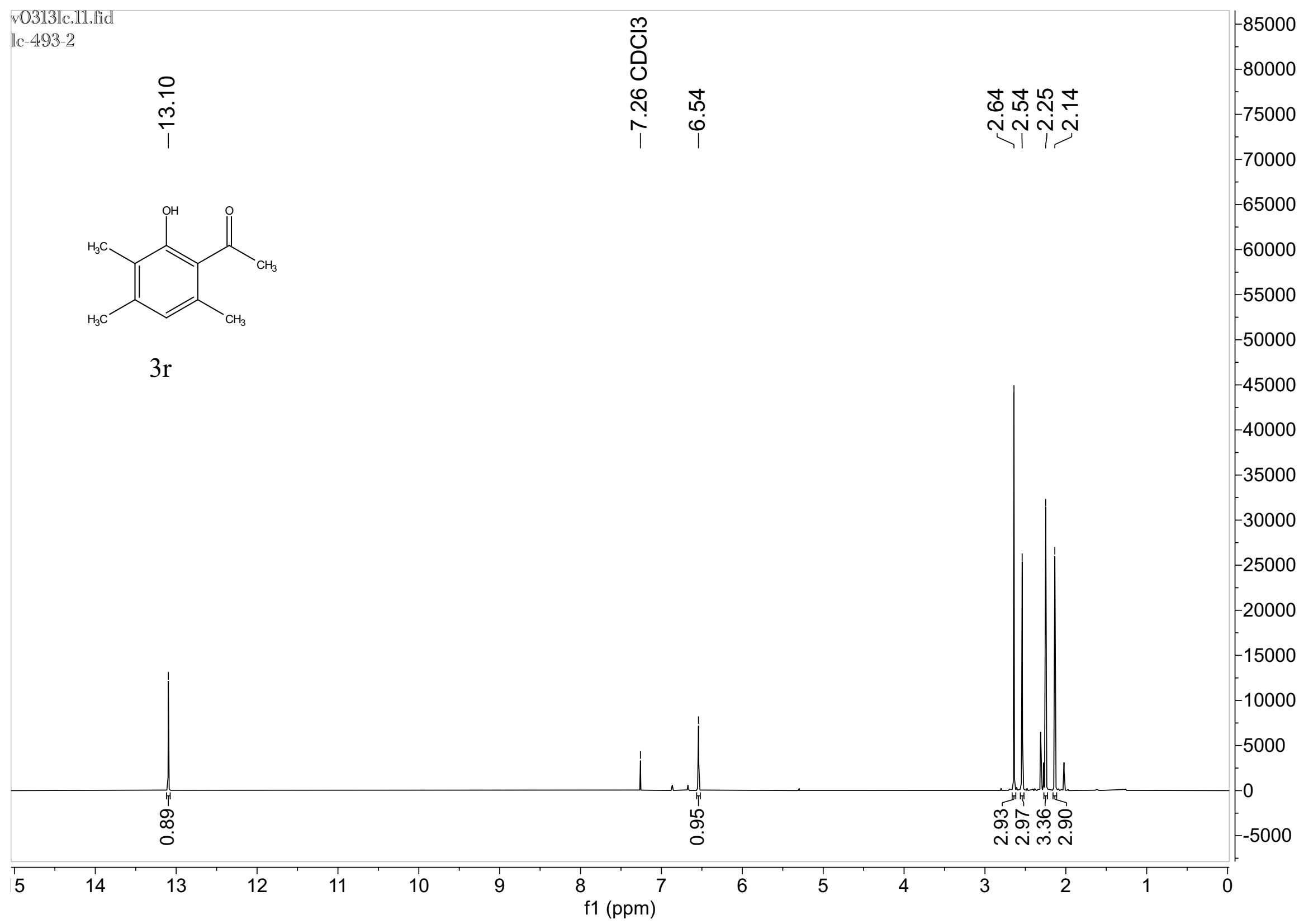
v0313lc.11.fid
lc-493-2
13.10
7.26 CDCl3
6.54
2.64
2.54
2.25
2.14
OH
O
H3C
CH3
H3C
CH3
3r
0.89
0.95
2.93
2.97
3.36
2.90
f1 (ppm)

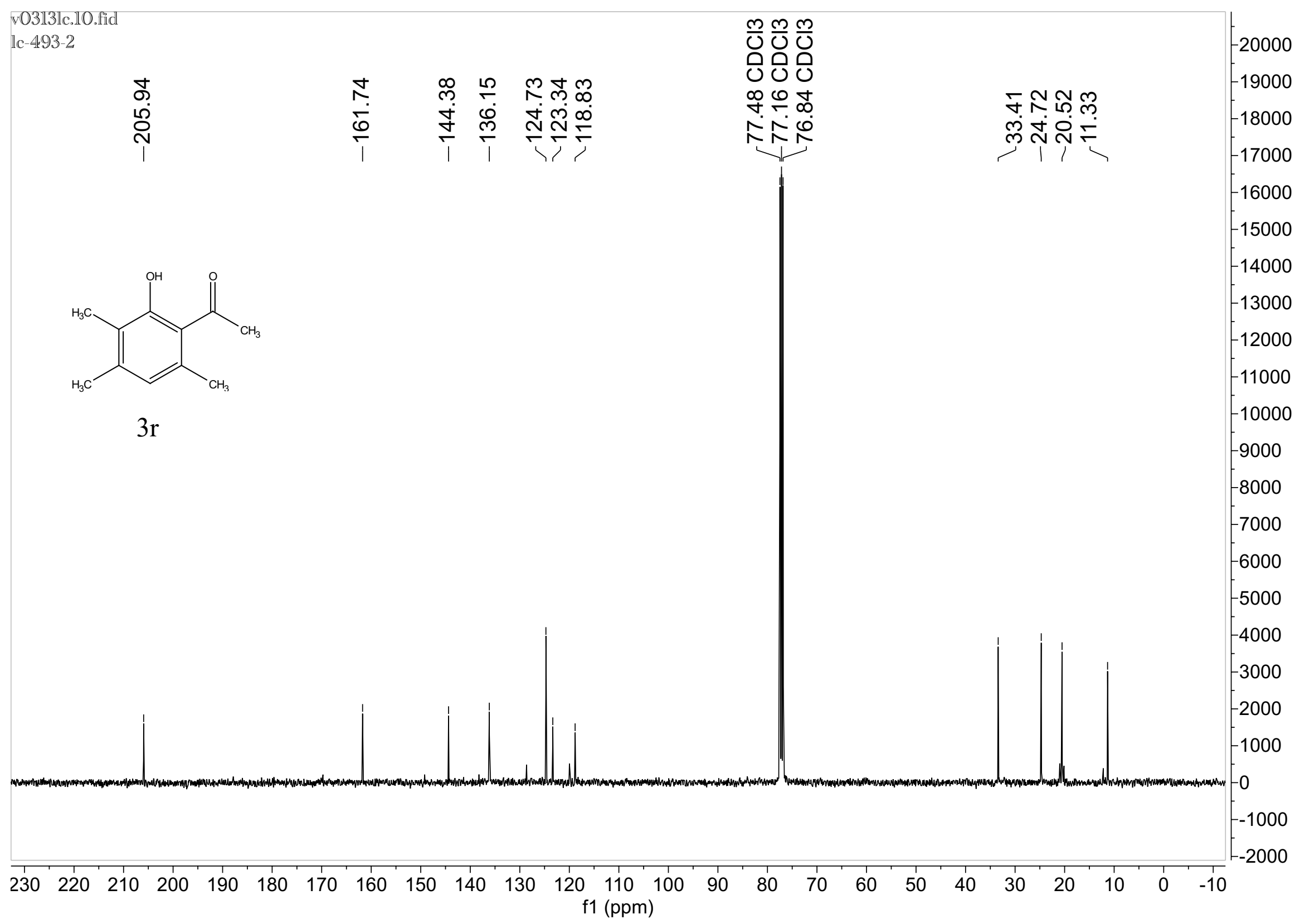
v0313lc.10.fid
lc-493-2
205.94
161.74
144.38
136.15
124.73
123.34
118.83
77.48 CDCl3
77.16 CDCl3
76.84 CDCl3
33.41
24.72
20.52
11.33
OH
O
H3C
CH3
H3C
CH3
3r
f1 (ppm)

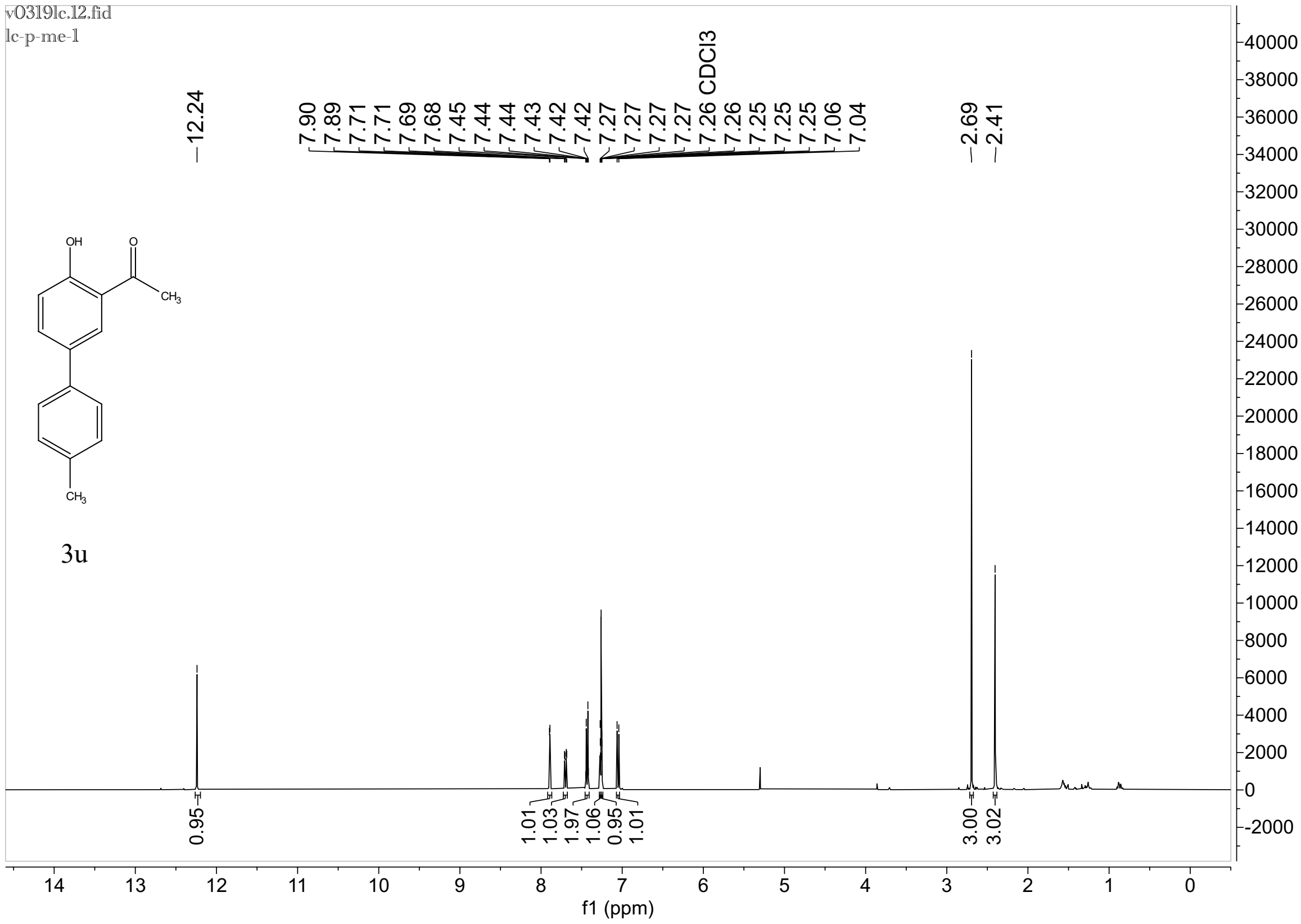
v0319lc.12.fid
lc-p-me-1
12.24
7.90 7.89 7.71 7.71 7.69 7.68 7.45 7.44 7.44 7.43 7.42 7.42 7.27 7.27 7.27 7.27 7.26 CDCl3 7.26 7.25 7.25 7.25 7.06 7.04
2.69
2.41
3u
0.95
1.01 1.03 1.97 1.06 0.95 1.01
3.00 3.02
f1 (ppm)

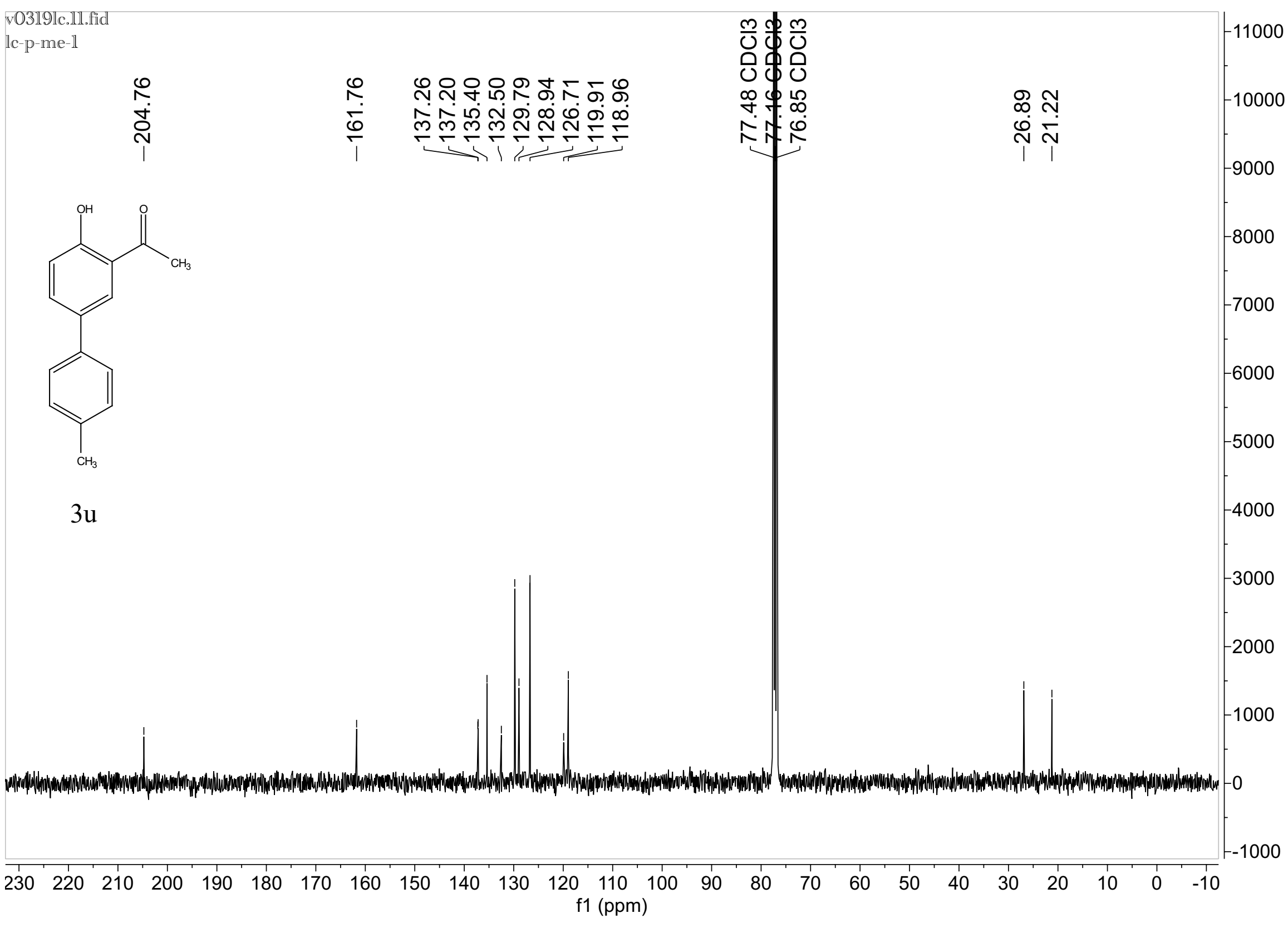
v0319lc.11.fid
lc-p-me-1
204.76
161.76
137.26 137.20 135.40 132.50 129.79 128.94 126.71 119.91 118.96
77.48 CDCl3 77.16 CDCl3 76.85 CDCl3
26.89
21.22
3u
f1 (ppm)

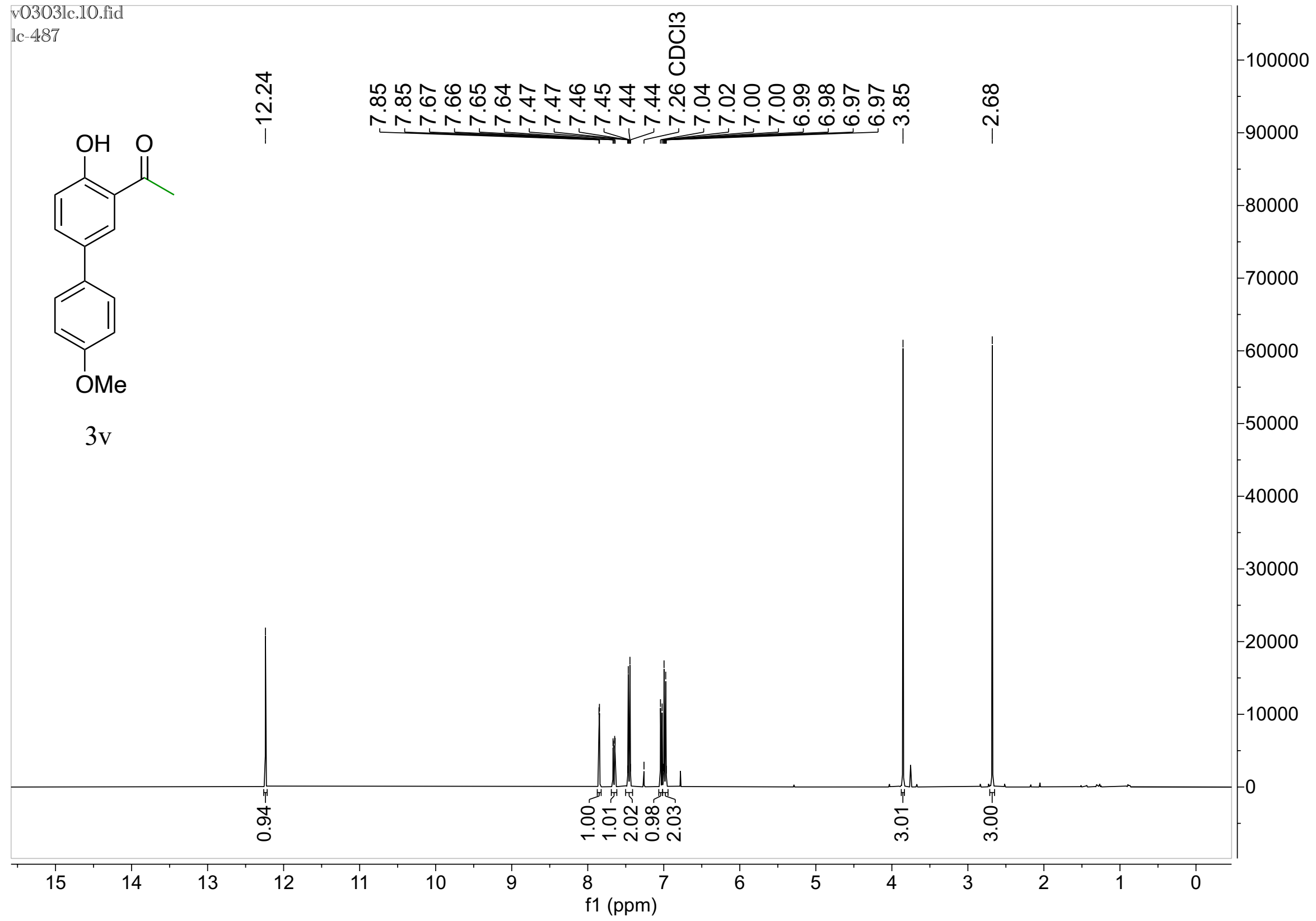
v0303lc.10.fid
lc-487
12.24
7.85
7.85
7.67
7.66
7.65
7.64
7.47
7.47
7.46
7.45
7.44
7.44
7.26 CDCl3
7.04
7.02
7.00
7.00
6.99
6.98
6.97
6.97
3.85
2.68
OH
O
OMe
0.94
1.00
1.01
2.02
0.98
2.03
3.01
3.00
f1 (ppm)

3v

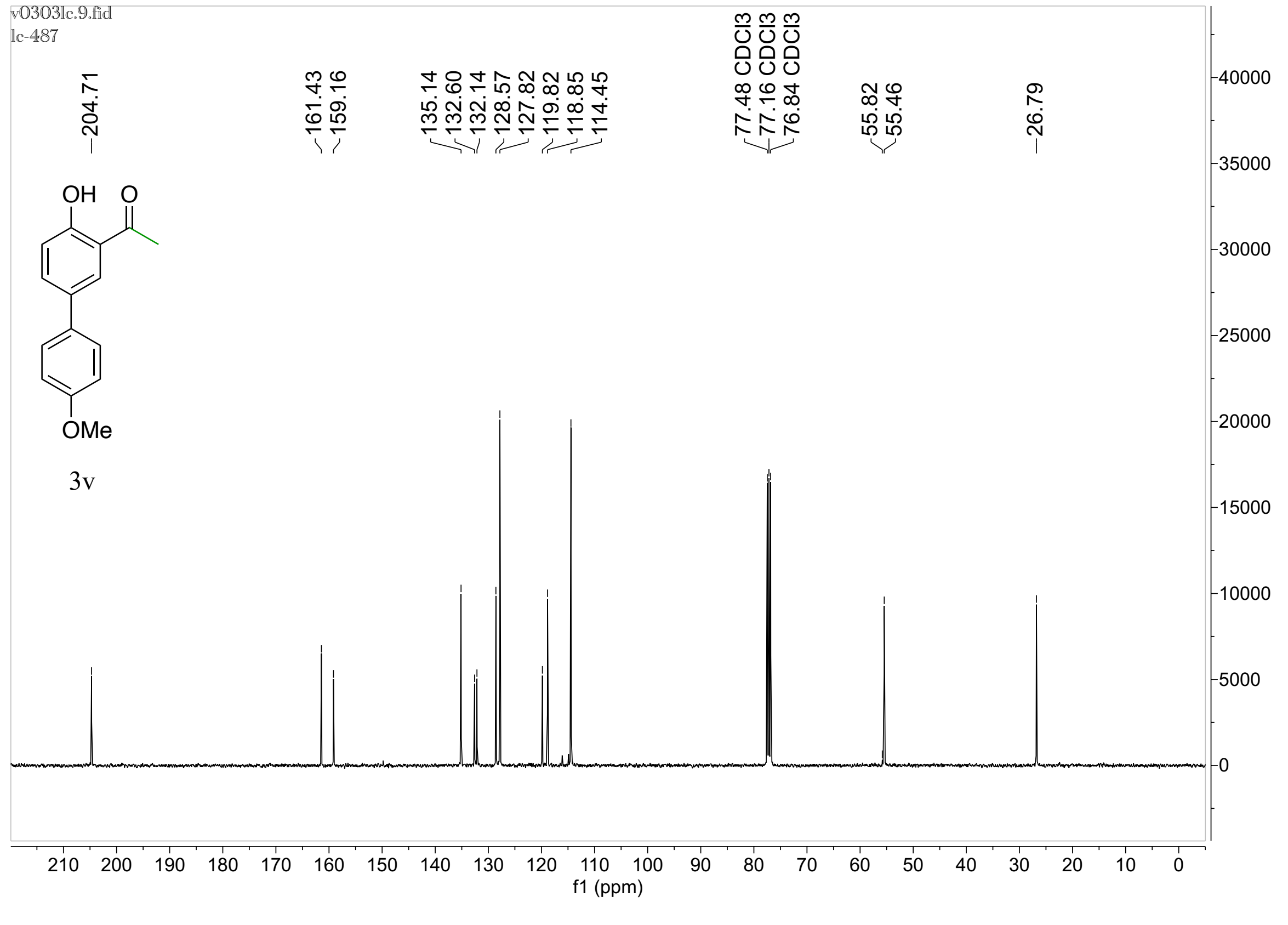
v0303lc.9.fid
lc-487
204.71
161.43
159.16
135.14
132.60
132.14
128.57
127.82
119.82
118.85
114.45
77.48 CDCl3
77.16 CDCl3
76.84 CDCl3
55.82
55.46
26.79
OH
O
OMe
f1 (ppm)

3v

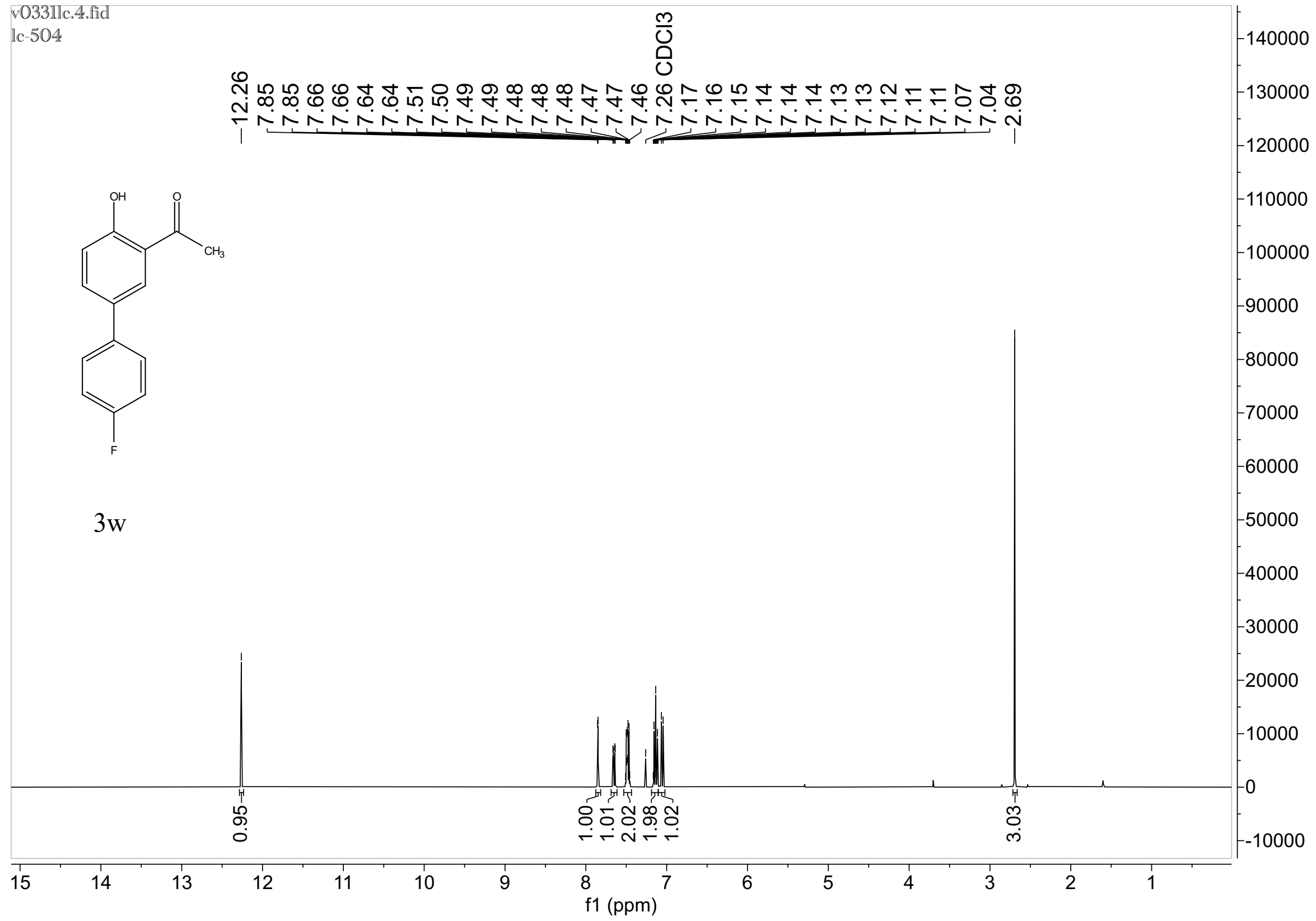
v0331lc.4.fid
lc-504
3w
f1 (ppm)

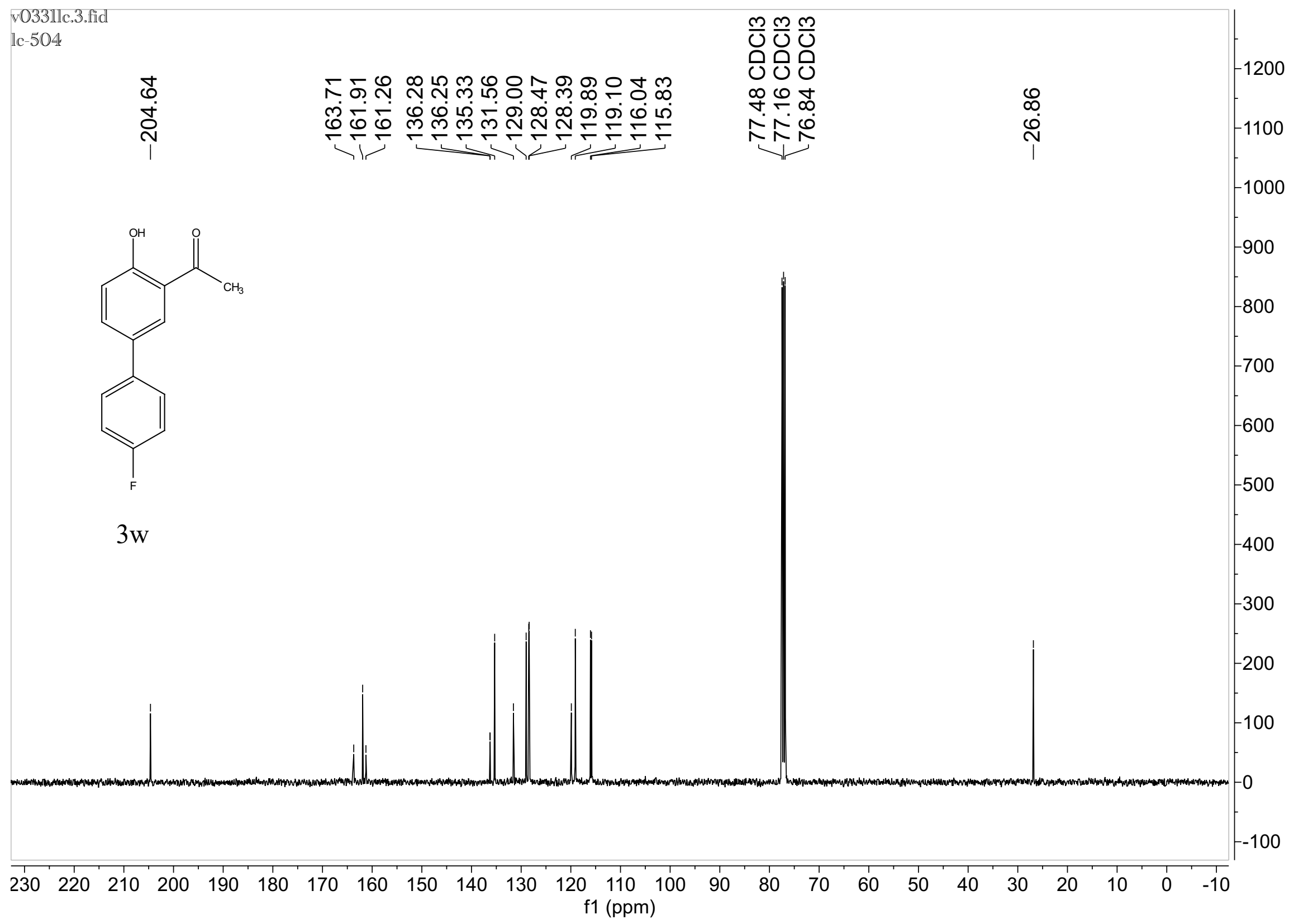
v0331lc.3.fid
lc-504
3w
f1 (ppm)

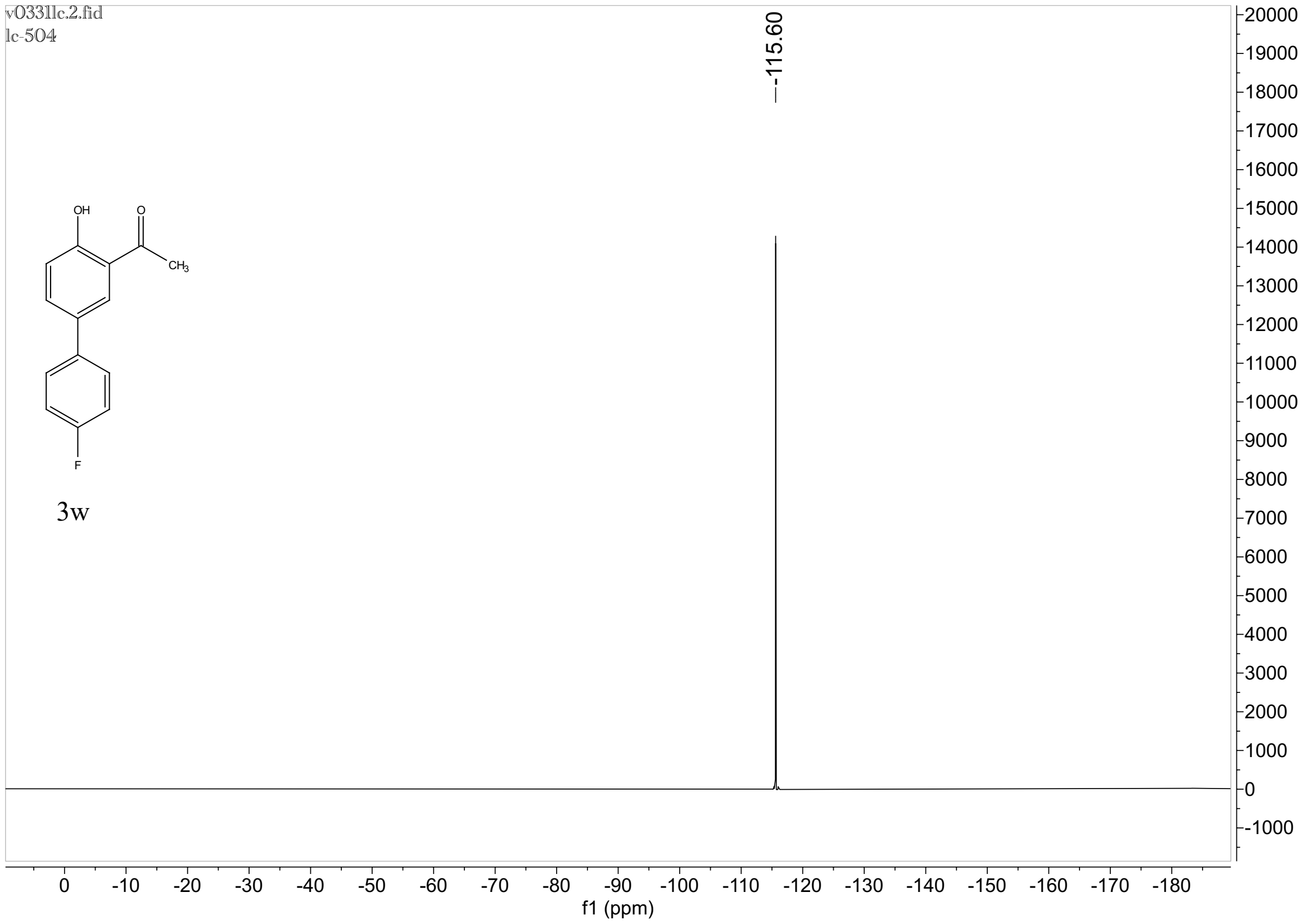
v0331lc.2.fid
lc-504
-115.60
OH
O
CH3
F
3w
20000
19000
18000
17000
16000
15000
14000
13000
12000
11000
10000
9000
8000
7000
6000
5000
4000
3000
2000
1000
0
-1000
0 -10 -20 -30 -40 -50 -60 -70 -80 -90 -100 -110 -120 -130 -140 -150 -160 -170 -180
f1 (ppm)

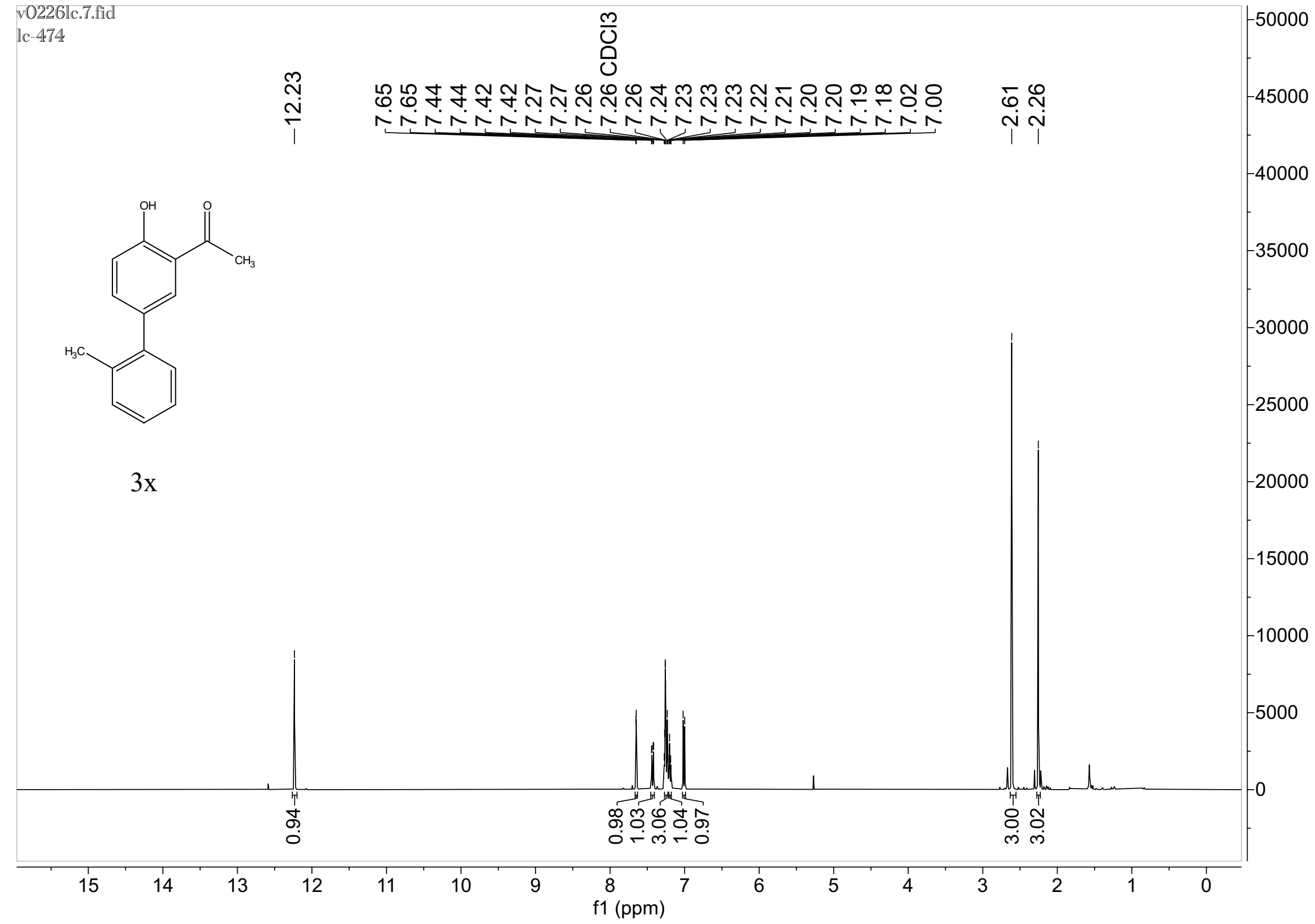
v0226lc.7.fid
lc-474
12.23
7.65 7.65 7.44 7.44 7.42 7.42 7.27 7.27 7.26 7.26 CDCl3 7.26 7.24 7.23 7.23 7.23 7.22 7.21 7.20 7.20 7.19 7.18 7.02 7.00
2.61 2.26
OH
O
CH3
H3C
3x
0.94
0.98 1.03 3.06 1.04 0.97
3.00 3.02
f1 (ppm)

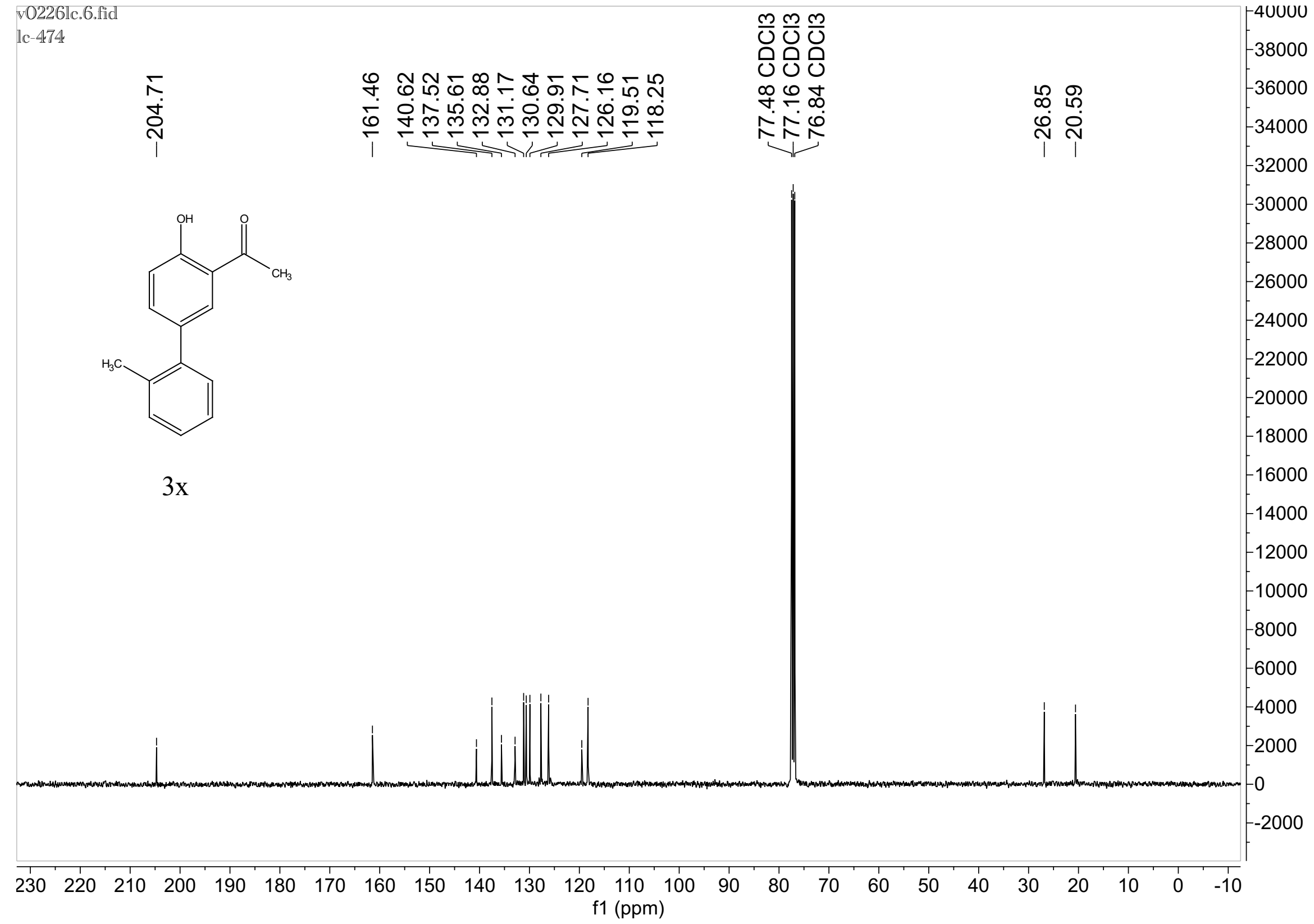
v0226lc.6.fid
lc-474
204.71
161.46
140.62 137.52 135.61 132.88 131.17 130.64 129.91 127.71 126.16 119.51 118.25
77.48 CDCl3 77.16 CDCl3 76.84 CDCl3
26.85 20.59
OH
O
CH3
H3C
3x
f1 (ppm)

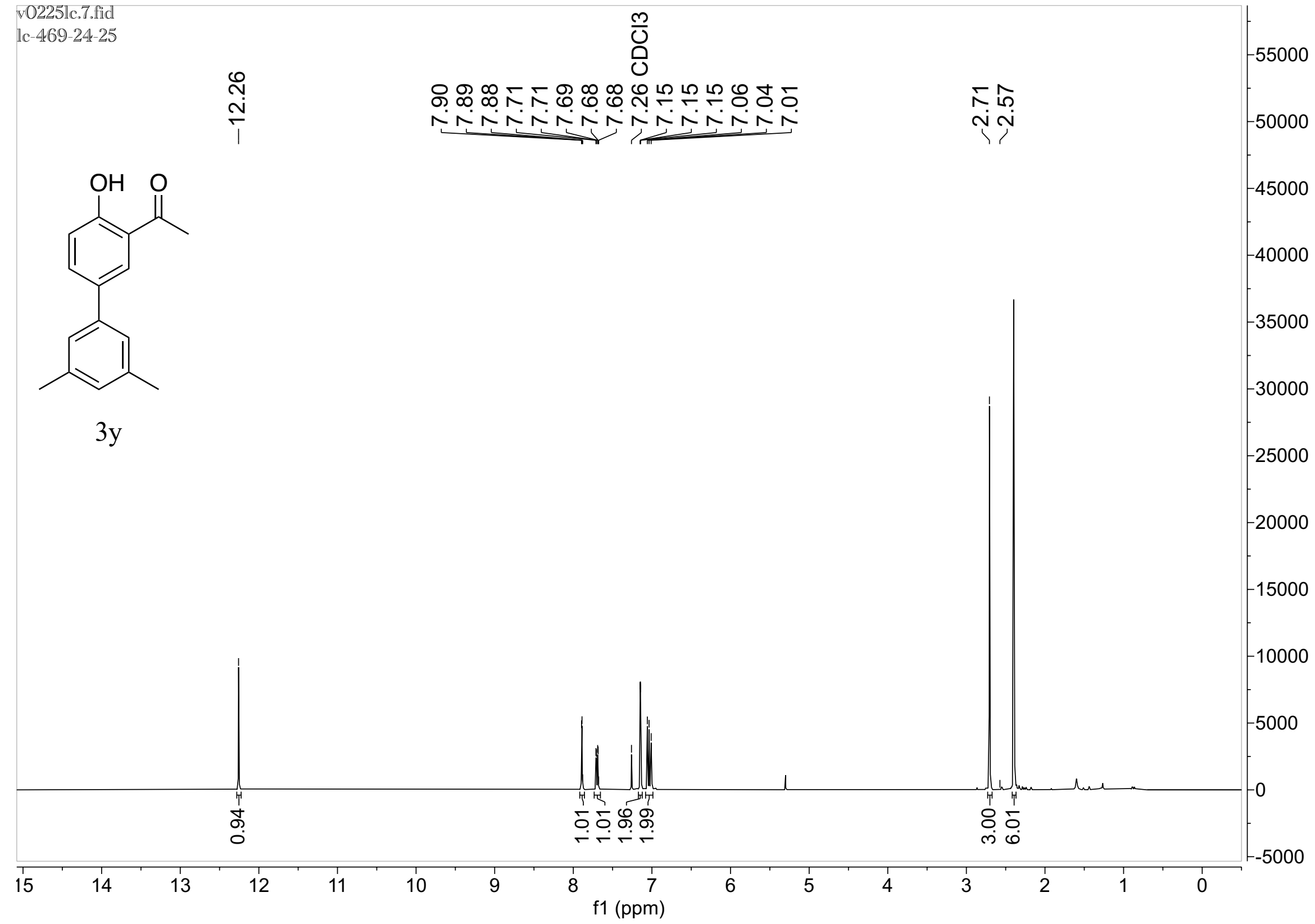
v0225lc.7.fid
lc-469-24-25
12.26
7.90 7.89 7.88 7.71 7.71 7.69 7.68 7.68 7.26 CDCl3 7.15 7.15 7.15 7.06 7.04 7.01
2.71 2.57
OH
O
0.94
1.01 1.01 1.96 1.99
3.00 6.01
f1 (ppm)

3y

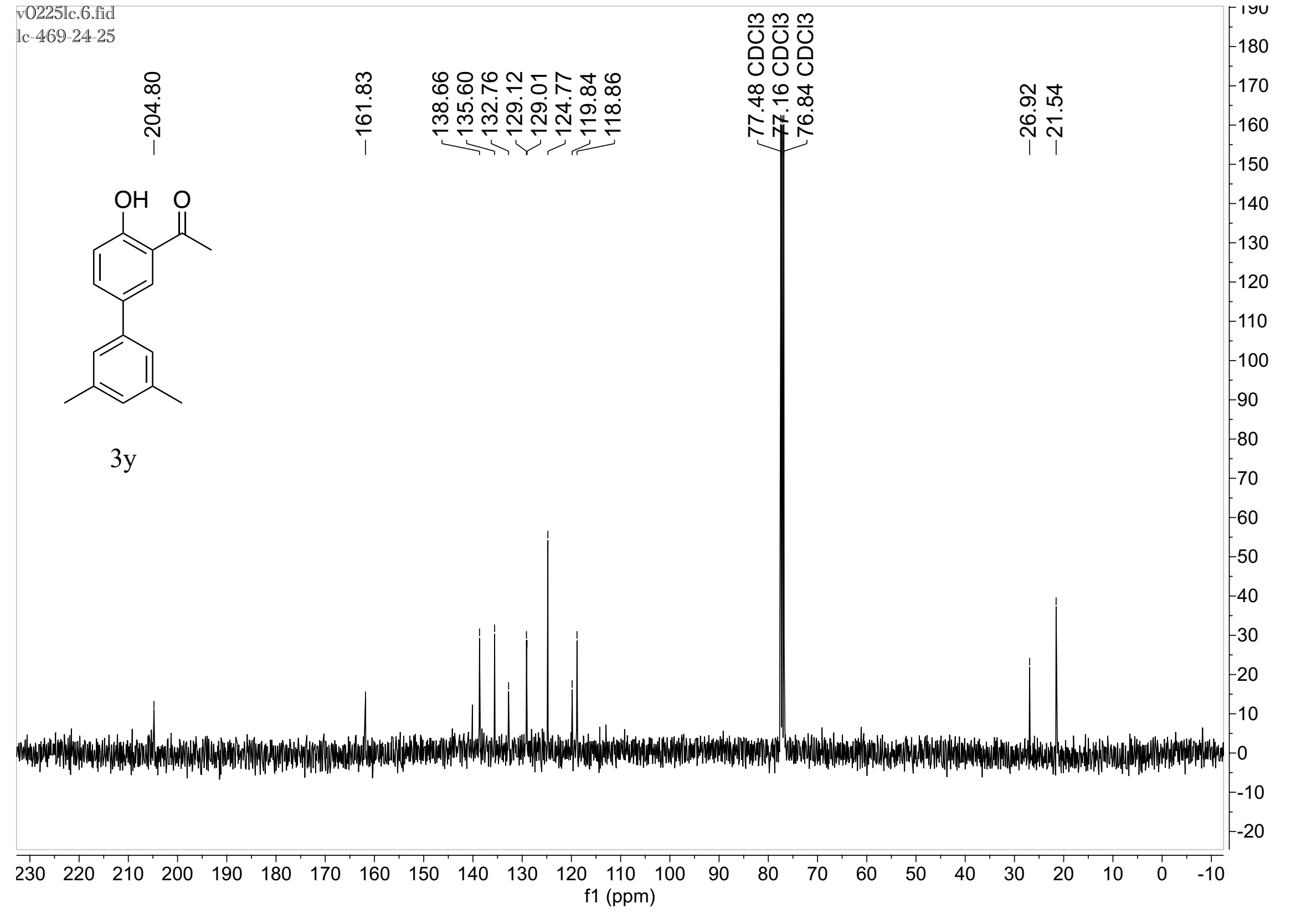
v0225lc.6.fid
lc-469-24-25
204.80
161.83
138.66 135.60 132.76 129.12 129.01 124.77 119.84 118.86
77.48 CDCl3 77.16 CDCl3 76.84 CDCl3
26.92 21.54
OH
O
f1 (ppm)

3y

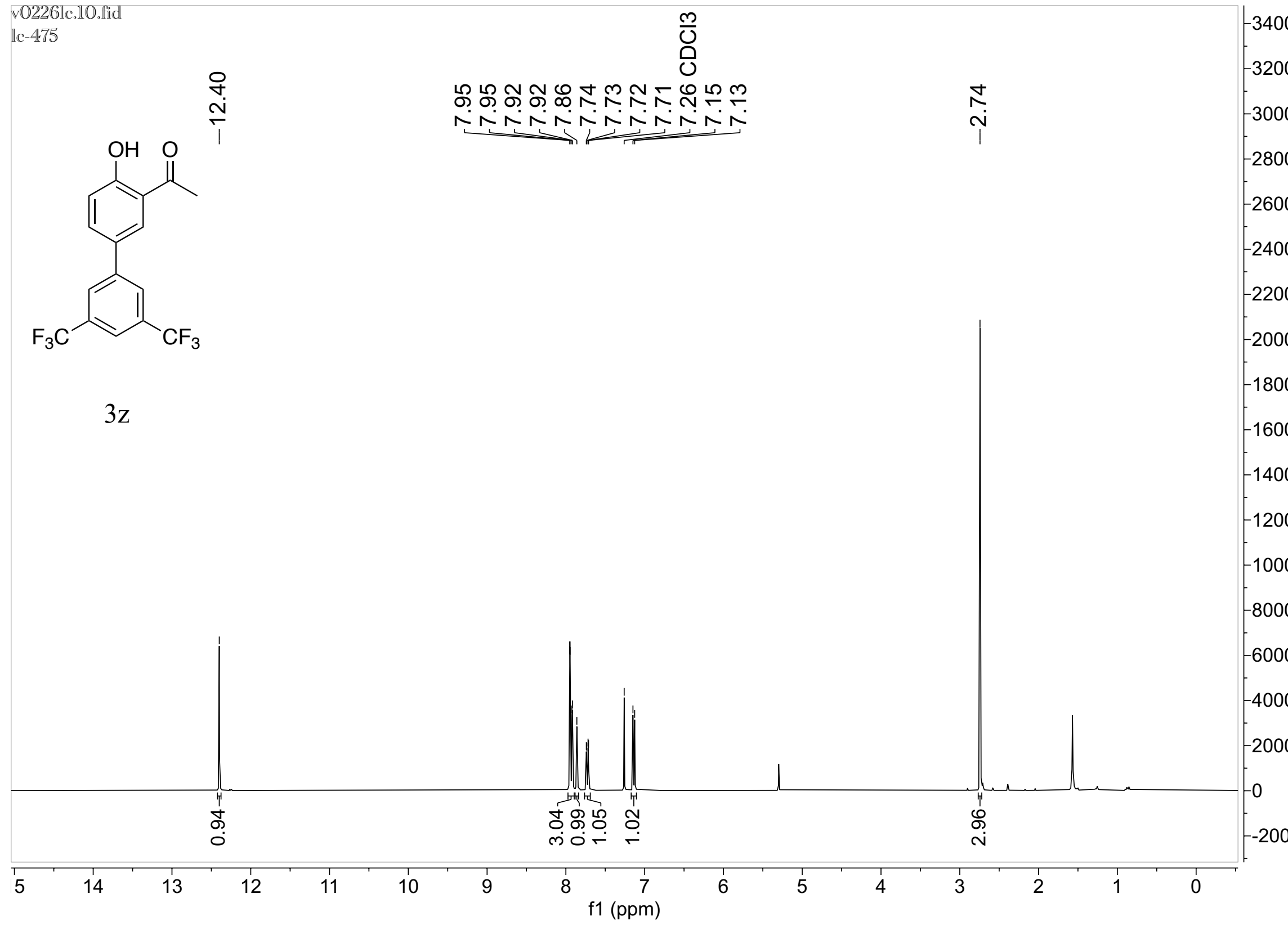
v0226lc.10.fid
lc-475
12.40
7.95
7.95
7.92
7.92
7.86
7.74
7.73
7.72
7.71
7.26 CDCl3
7.15
7.13
2.74
OH
O
F3C
CF3
3z
0.94
3.04
0.99
1.05
1.02
2.96
f1 (ppm)

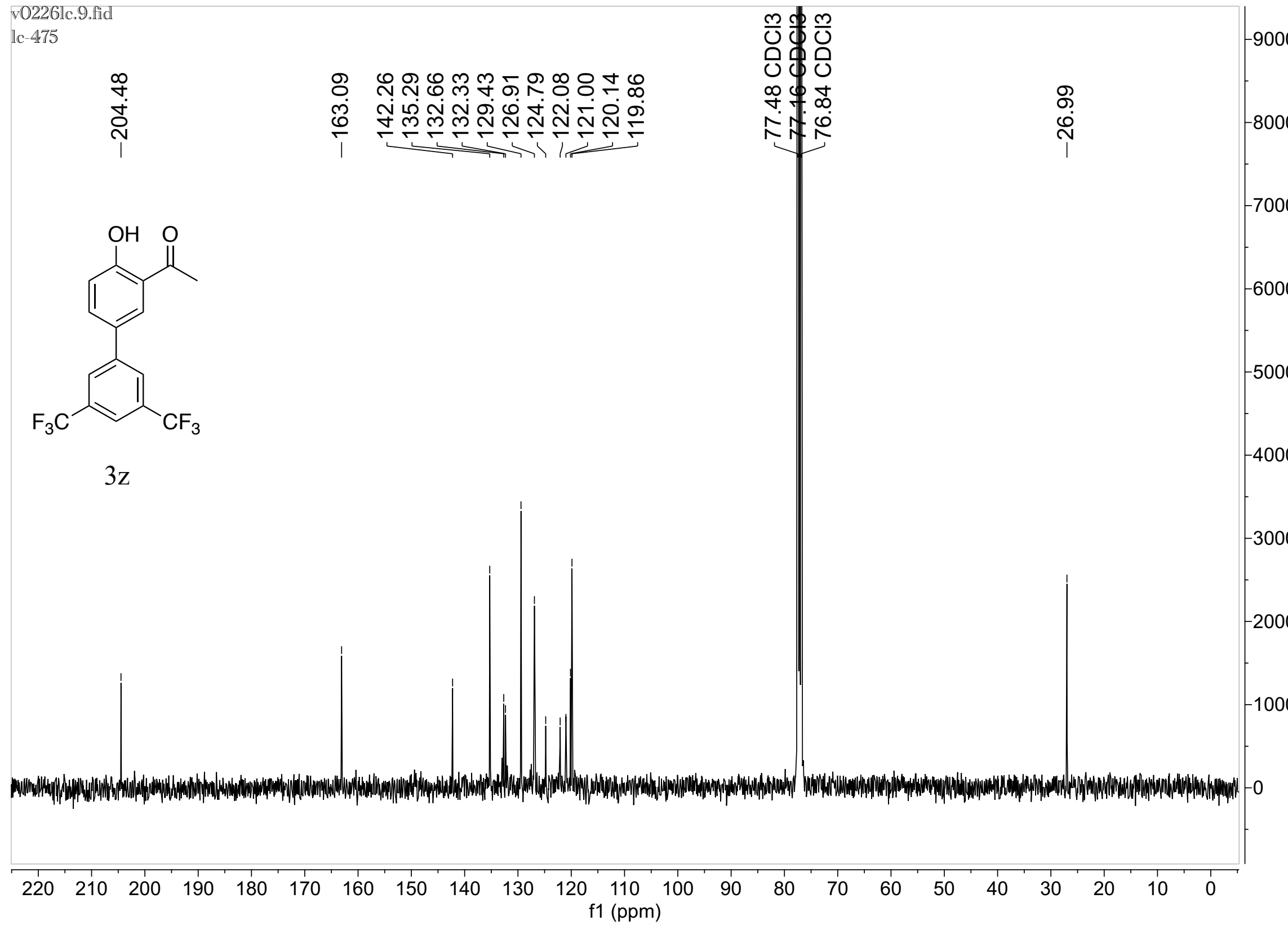
v0226lc.9.fid
lc-475
204.48
163.09
142.26
135.29
132.66
132.33
129.43
126.91
124.79
122.08
121.00
120.14
119.86
77.48 CDCl3
77.16 CDCl3
76.84 CDCl3
26.99
OH
O
F3C
CF3
3z
f1 (ppm)

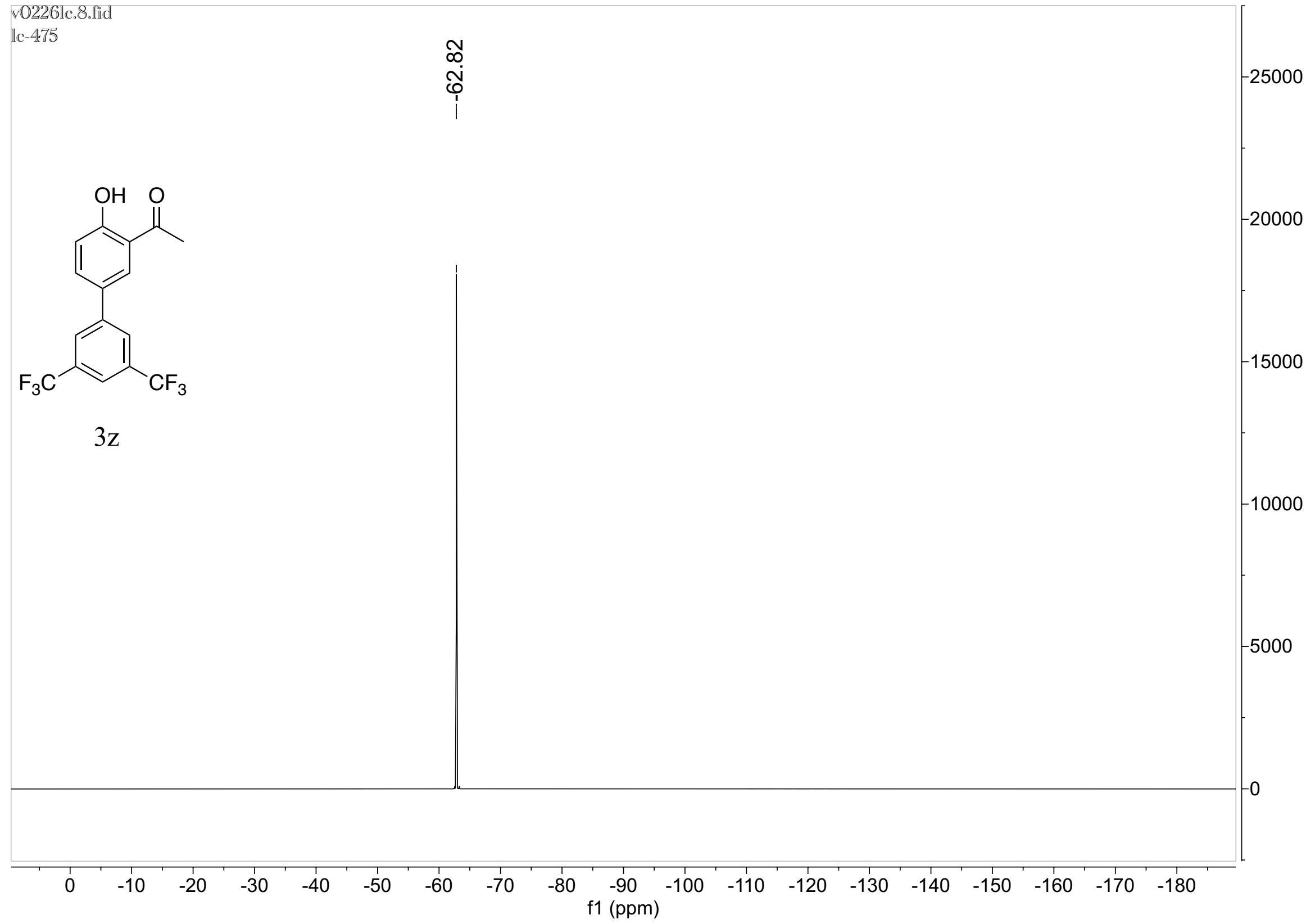

v0226lc.8.fid
lc-475
-62.82
OH
O
F3C
CF3
25000
20000
15000
10000
5000
0
0 -10 -20 -30 -40 -50 -60 -70 -80 -90 -100 -110 -120 -130 -140 -150 -160 -170 -180
f1 (ppm)

3z

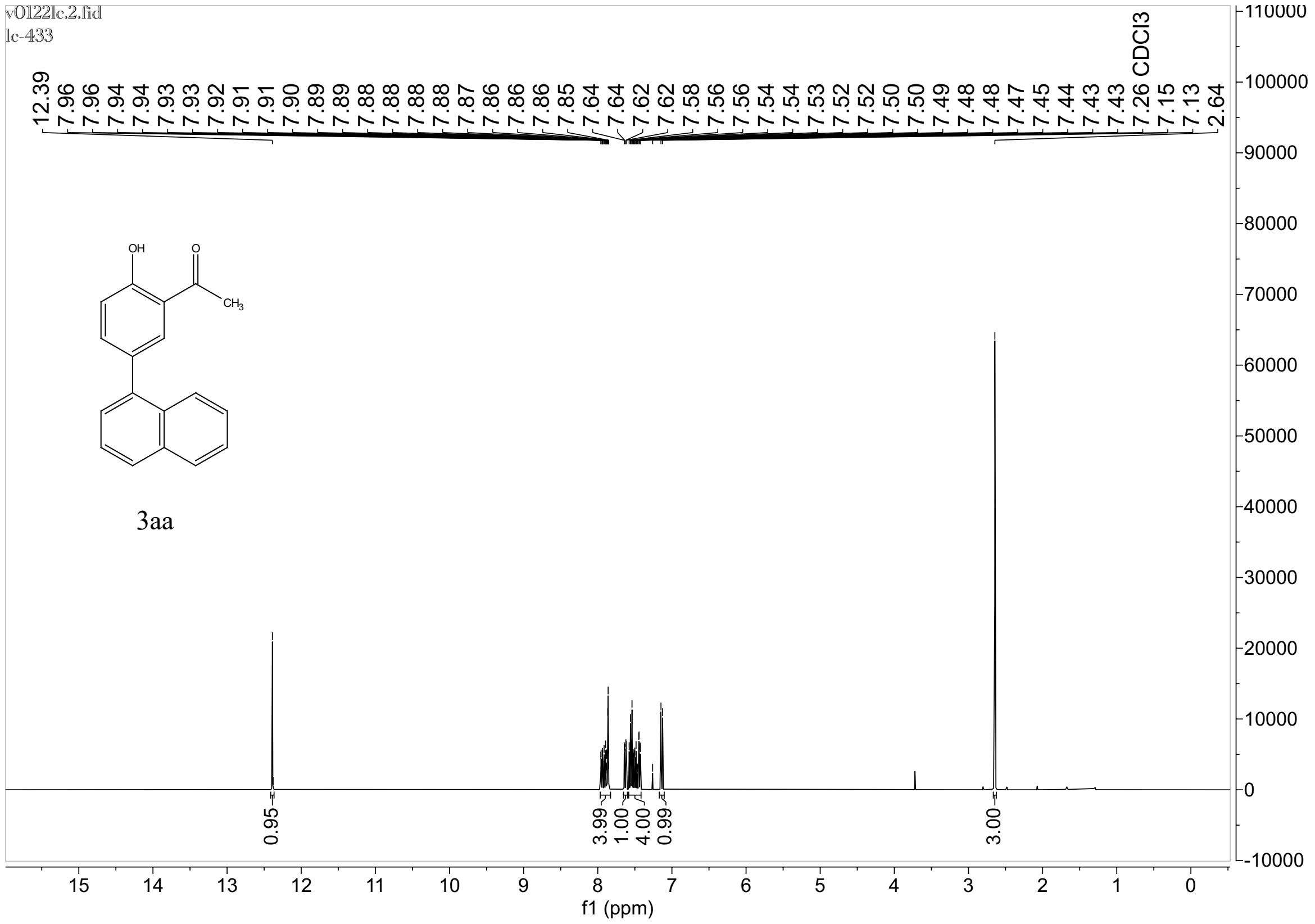
v0122lc.2.fid
lc-433
12.39
7.96
7.96
7.94
7.94
7.93
7.93
7.92
7.91
7.91
7.90
7.89
7.89
7.88
7.88
7.88
7.88
7.87
7.86
7.86
7.86
7.85
7.64
7.62
7.62
7.58
7.56
7.54
7.54
7.53
7.52
7.52
7.50
7.50
7.49
7.48
7.48
7.47
7.45
7.44
7.43
7.43
7.26 CDCl3
7.15
7.13
2.64
OH
O
CH3
3aa
0.95
3.99
1.00
4.00
0.99
3.00
f1 (ppm)

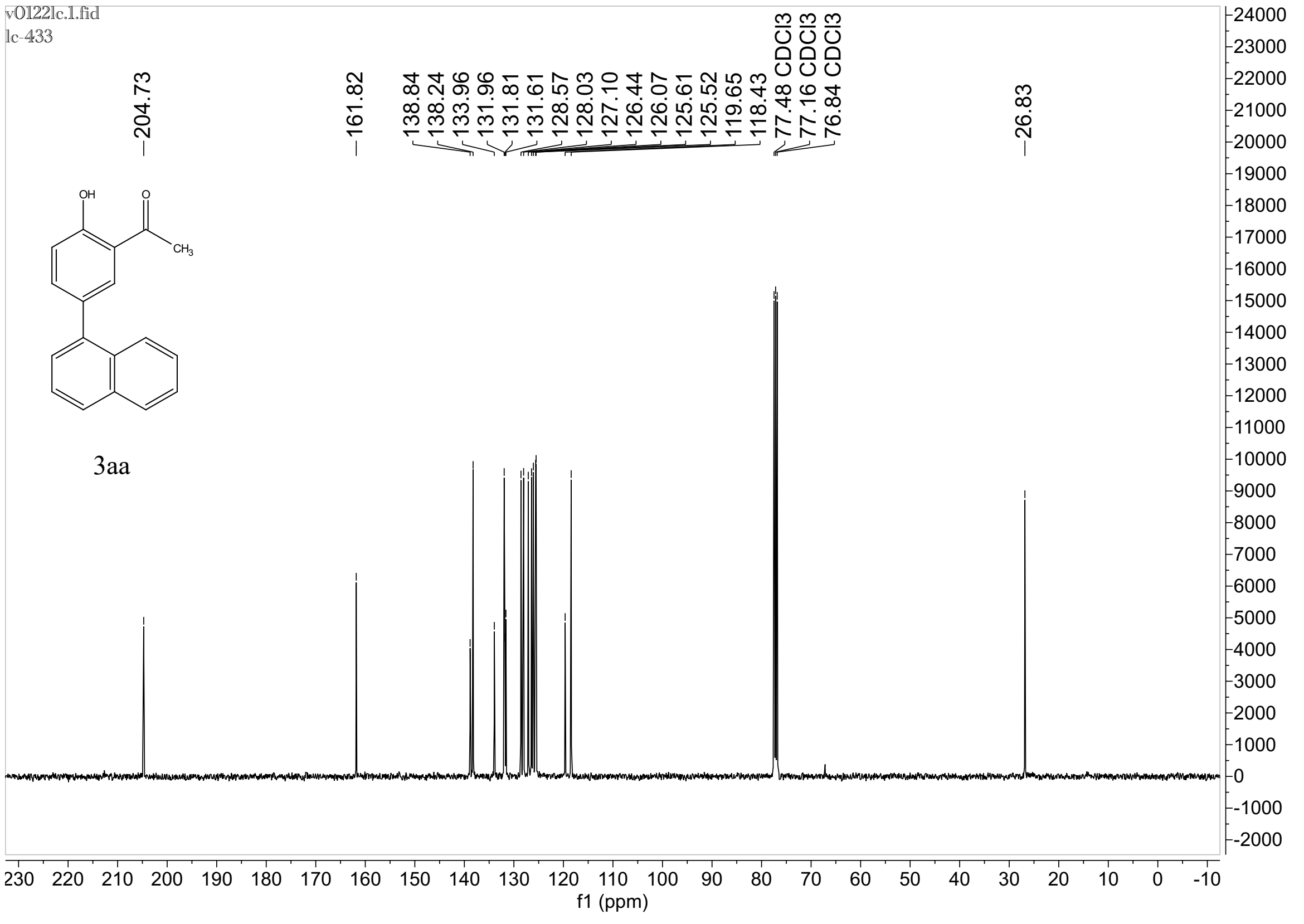
v0122lc.1.fid
lc-433
204.73
161.82
138.84
138.24
133.96
131.96
131.81
131.61
128.57
128.03
127.10
126.44
126.07
125.61
125.52
119.65
118.43
77.48 CDCl3
77.16 CDCl3
76.84 CDCl3
26.83
OH
O
CH3
3aa
f1 (ppm)

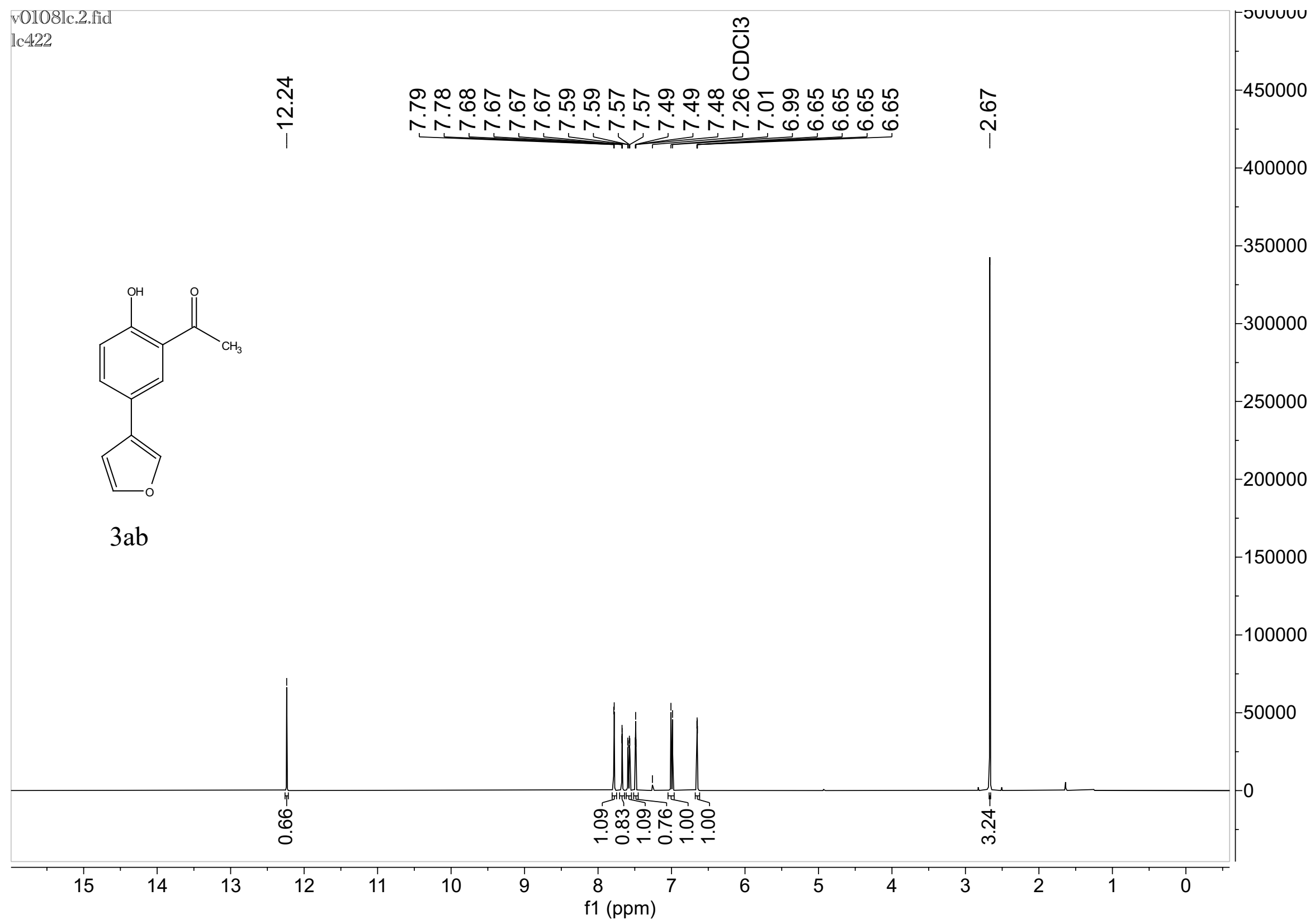
v0108lc.2.fid
lc422
12.24
7.79
7.78
7.68
7.67
7.67
7.67
7.59
7.59
7.57
7.57
7.49
7.49
7.48
7.26 CDCl3
7.01
6.99
6.65
6.65
6.65
6.65
2.67
OH
O
CH3
O
3ab
0.66
1.09
0.83
1.09
0.76
1.00
1.00
3.24
f1 (ppm)

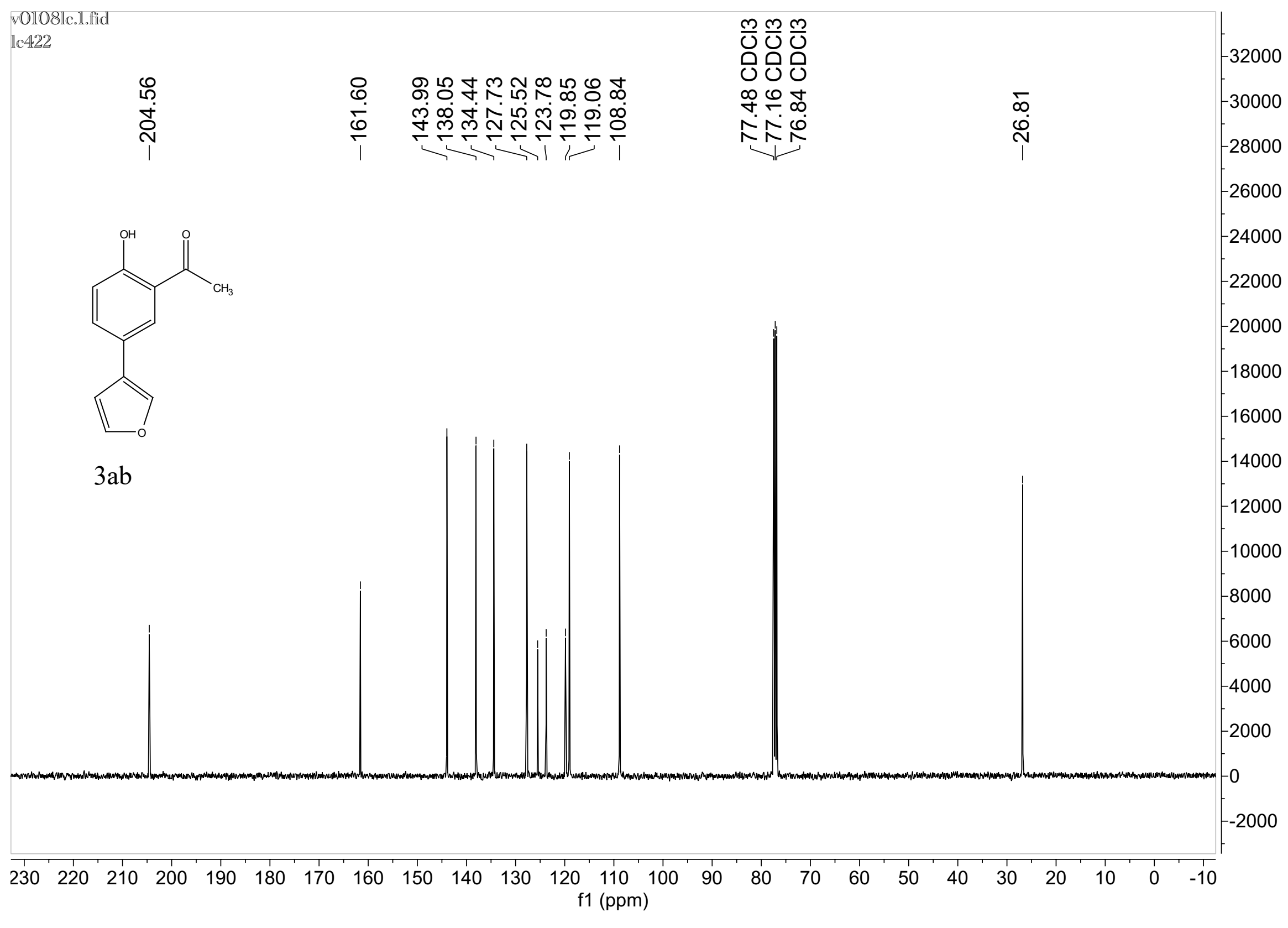
v0108lc.1.fid
lc422
204.56
161.60
143.99
138.05
134.44
127.73
125.52
123.78
119.85
119.06
108.84
77.48 CDCl3
77.16 CDCl3
76.84 CDCl3
26.81
OH
O
CH3
O
3ab
f1 (ppm)

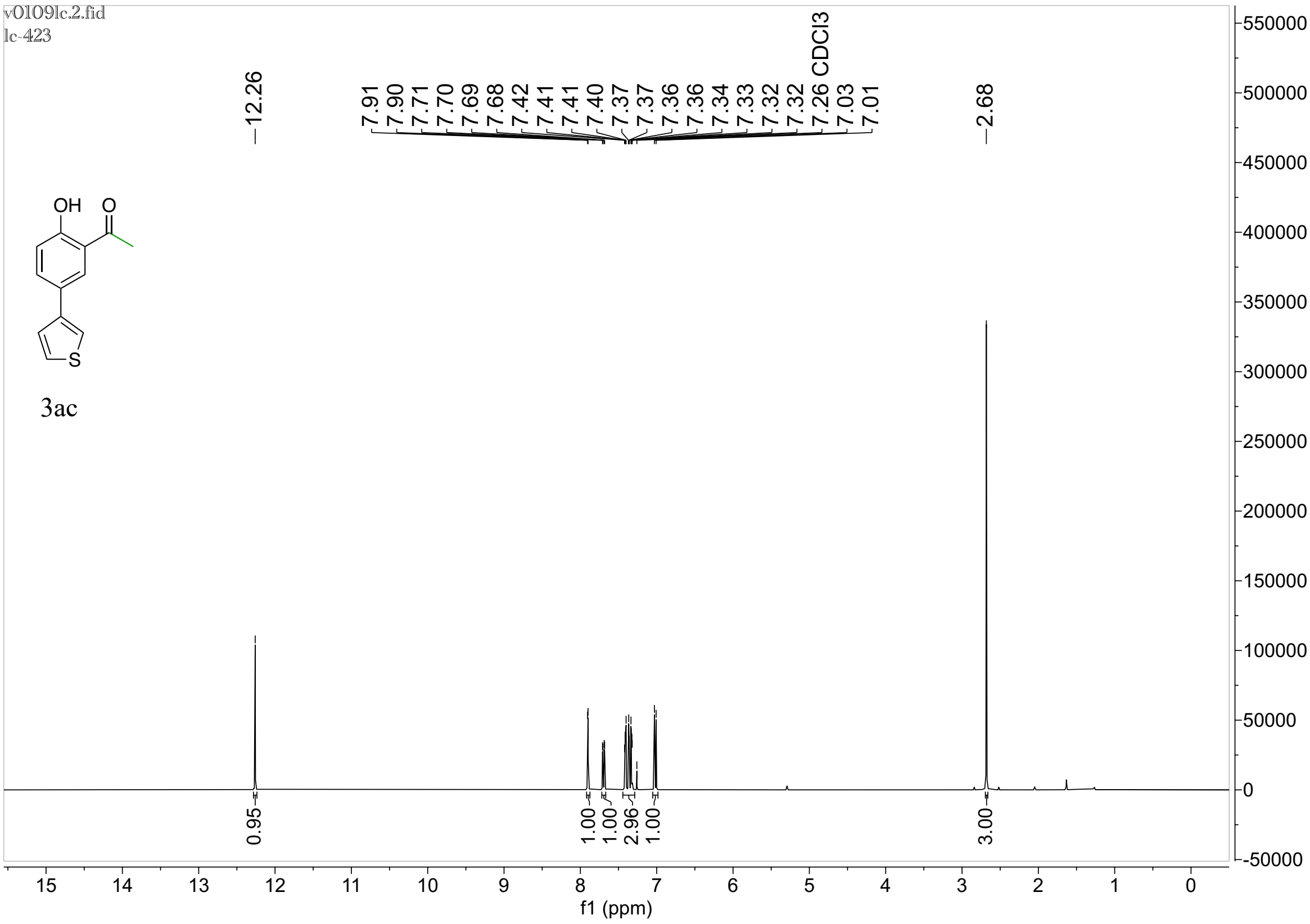
v0109lc.2.fid
lc-423
12.26
7.91 7.90 7.71 7.70 7.69 7.68 7.42 7.41 7.41 7.40 7.37 7.37 7.36 7.36 7.34 7.33 7.32 7.32 7.26 CDCl3 7.03 7.01
2.68
OH
O
S
0.95
1.00 1.00 2.96 1.00
3.00
f1 (ppm)

3ac

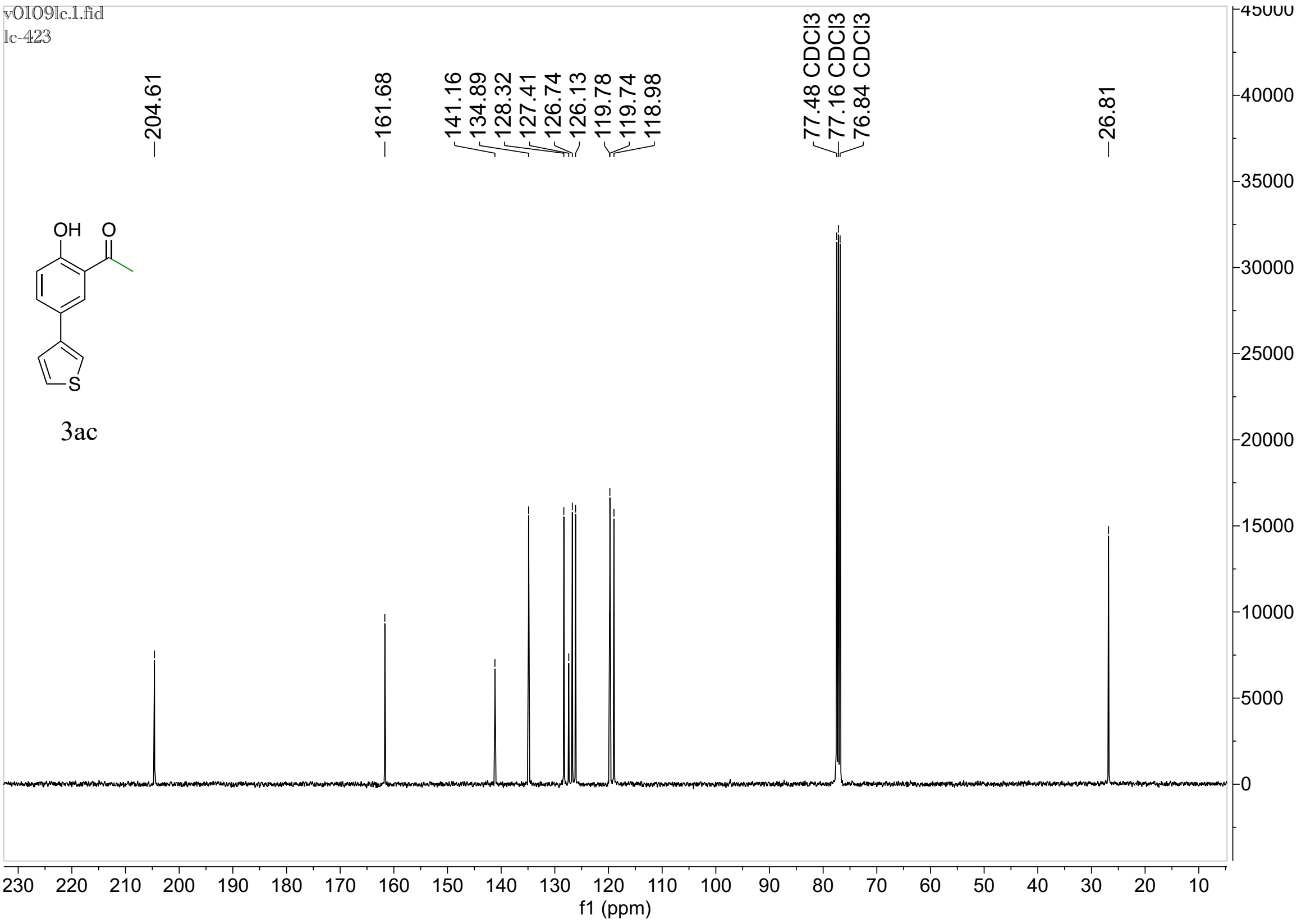
v0109lc.1.fid
lc-423
204.61
161.68
141.16 134.89 128.32 127.41 126.74 126.13 119.78 119.74 118.98
77.48 CDCl3 77.16 CDCl3 76.84 CDCl3
26.81
OH
O
S
f1 (ppm)

3ac

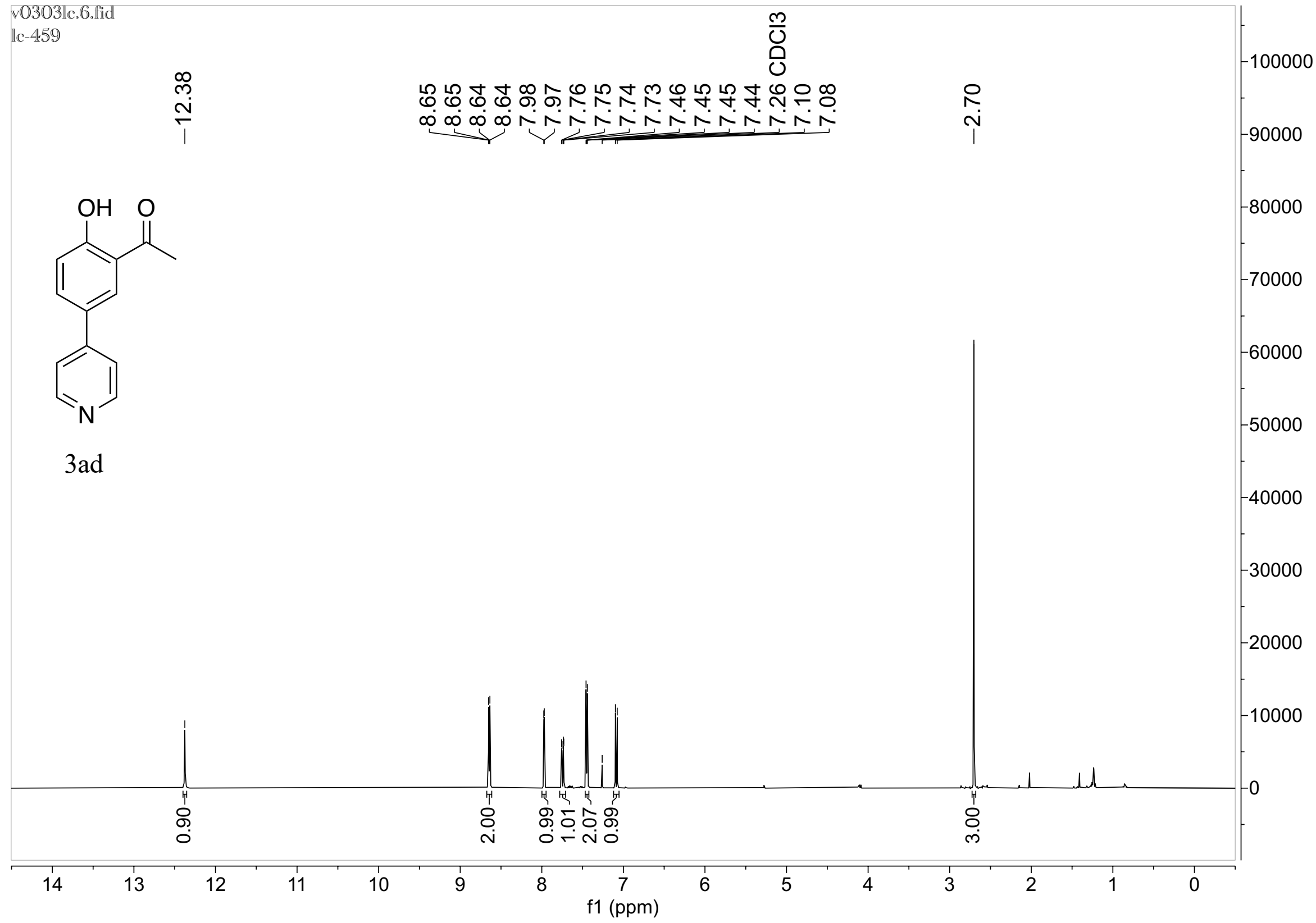
v0303lc.6.fid
lc-459
12.38
8.65
8.65
8.64
8.64
7.98
7.97
7.76
7.75
7.74
7.73
7.46
7.45
7.45
7.44
7.26 CDCl3
7.10
7.08
2.70
OH
O
N
3ad
0.90
2.00
0.99
1.01
2.07
0.99
3.00
f1 (ppm)

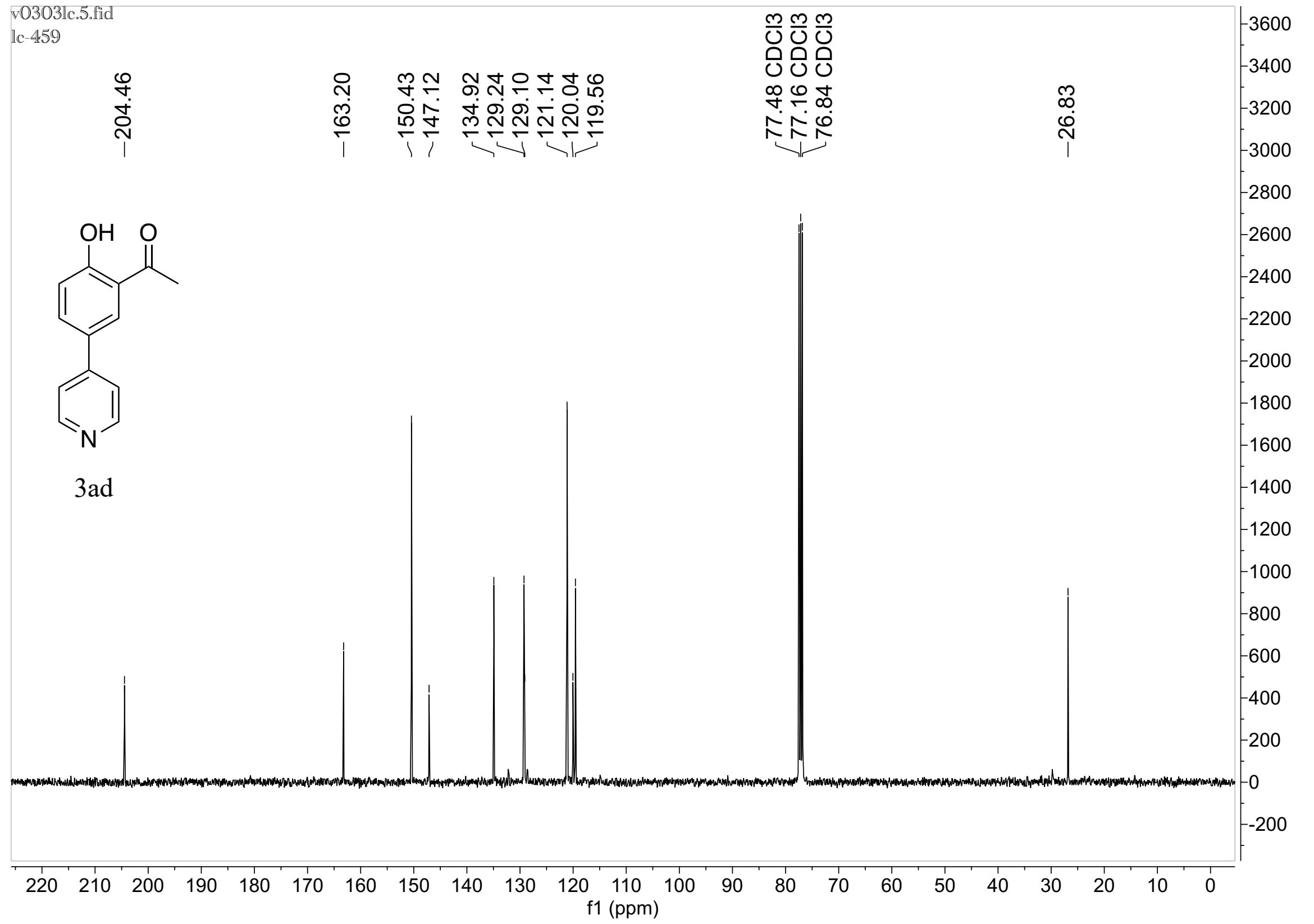
v0303lc.5.fid
lc-459
204.46
163.20
150.43
147.12
134.92
129.24
129.10
121.14
120.04
119.56
77.48 CDCl3
77.16 CDCl3
76.84 CDCl3
26.83
OH
O
N
3ad
f1 (ppm)

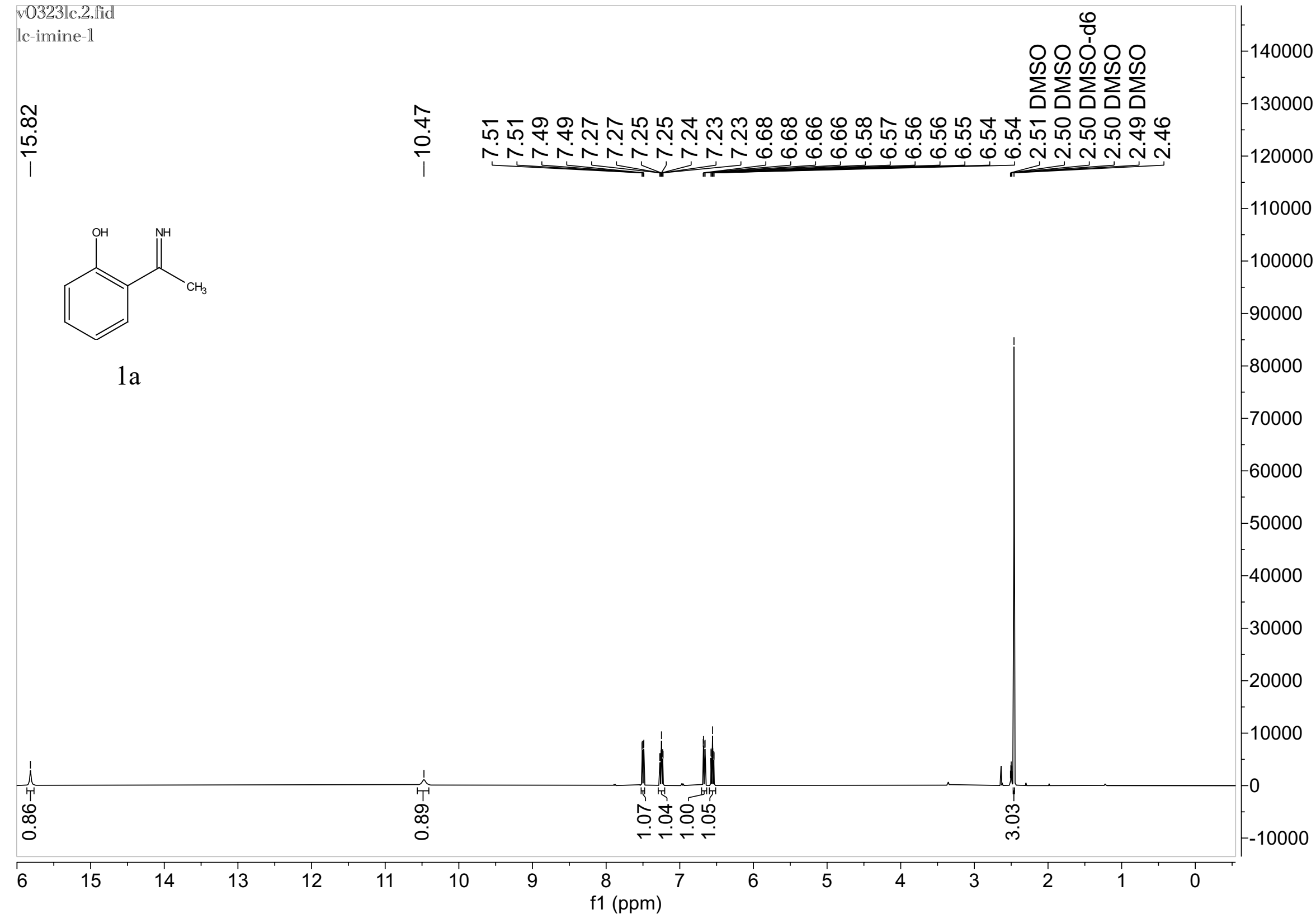
v0323lc.2.fid
lc-imine-1
15.82
10.47
7.51 7.51 7.49 7.49 7.27 7.27 7.25 7.25 7.24 7.23 7.23 6.68 6.68 6.66 6.66 6.58 6.57 6.56 6.56 6.55 6.54 6.54
2.51 DMSO 2.50 DMSO 2.50 DMSO-d6 2.50 DMSO 2.49 DMSO 2.46
OH
NH
CH3
1a
0.86
0.89
1.07 1.04 1.00 1.05
3.03
f1 (ppm)

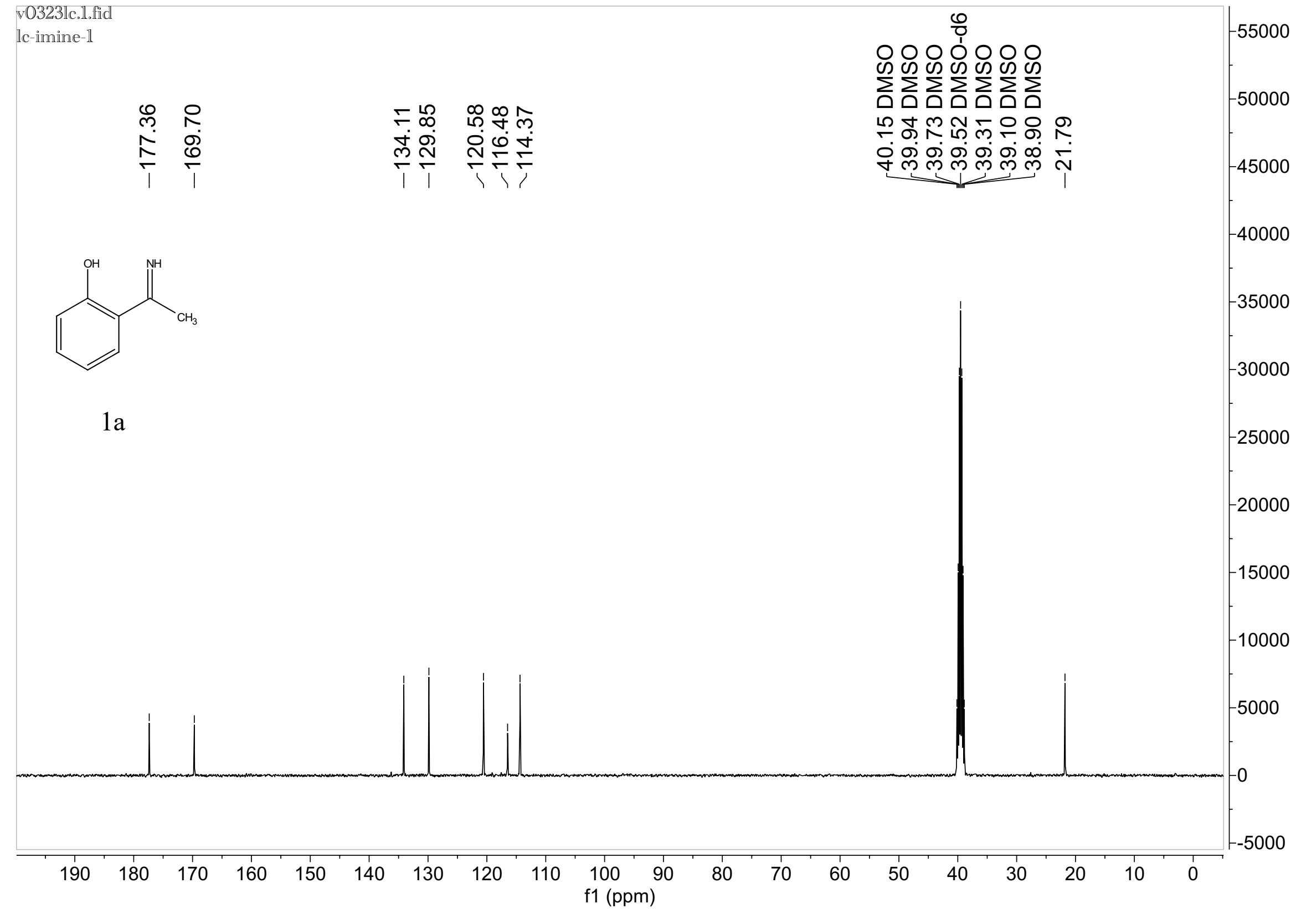
v0323lc.1.fid
lc-imine-1
177.36
169.70
134.11
129.85
120.58
116.48
114.37
40.15 DMSO 39.94 DMSO 39.73 DMSO 39.52 DMSO-d6 39.31 DMSO 39.10 DMSO 38.90 DMSO
21.79
OH
NH
CH3
1a
f1 (ppm)

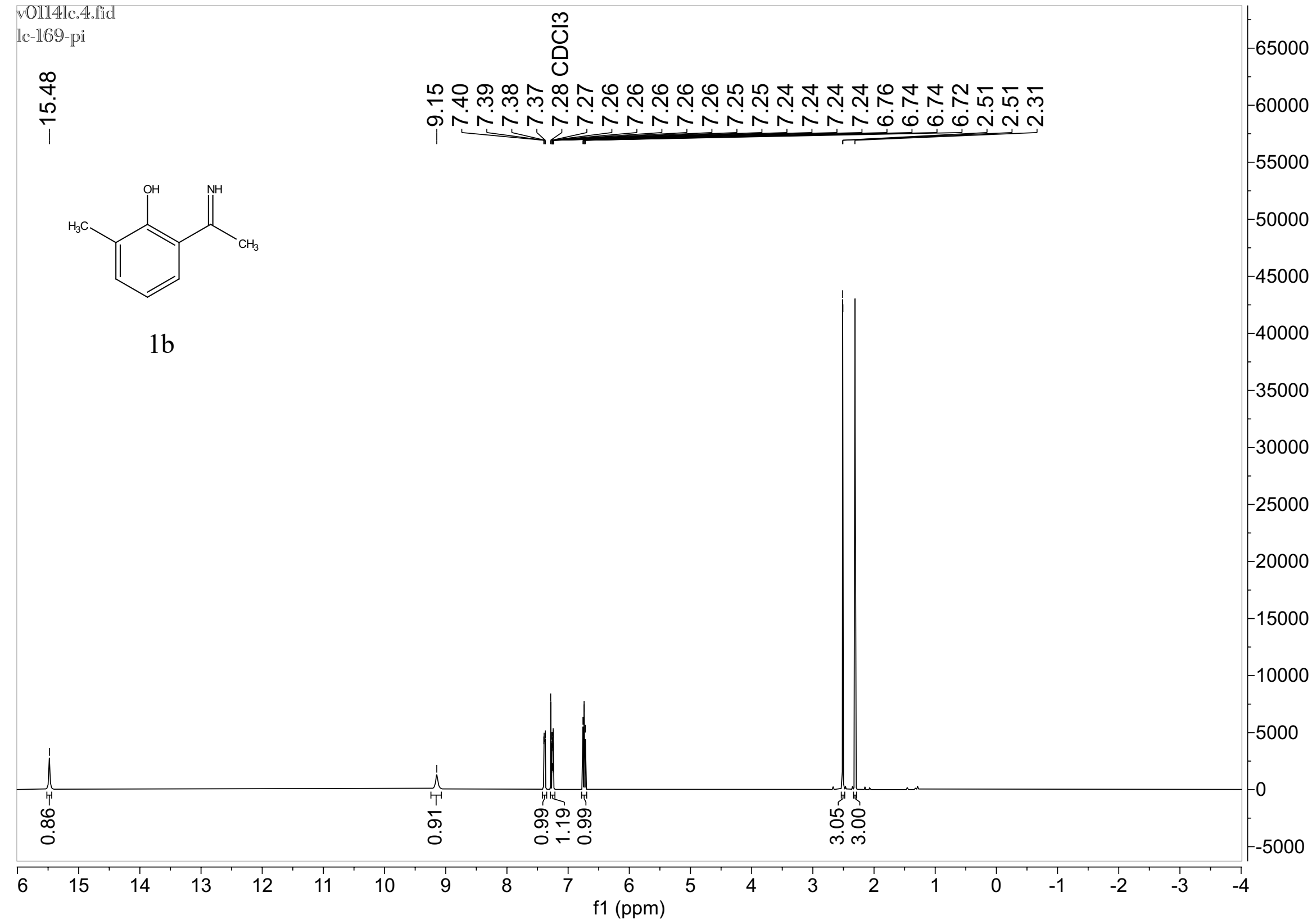
v0114lc.4.fid
lc-169-pi
15.48
9.15
7.40
7.39
7.38
7.37
7.28 CDCl3
7.27
7.26
7.26
7.26
7.26
7.25
7.25
7.24
7.24
7.24
7.24
6.76
6.74
6.74
6.72
2.51
2.51
2.31
OH
NH
H3C
CH3
1b
0.86
0.91
0.99
1.19
0.99
3.05
3.00
f1 (ppm)

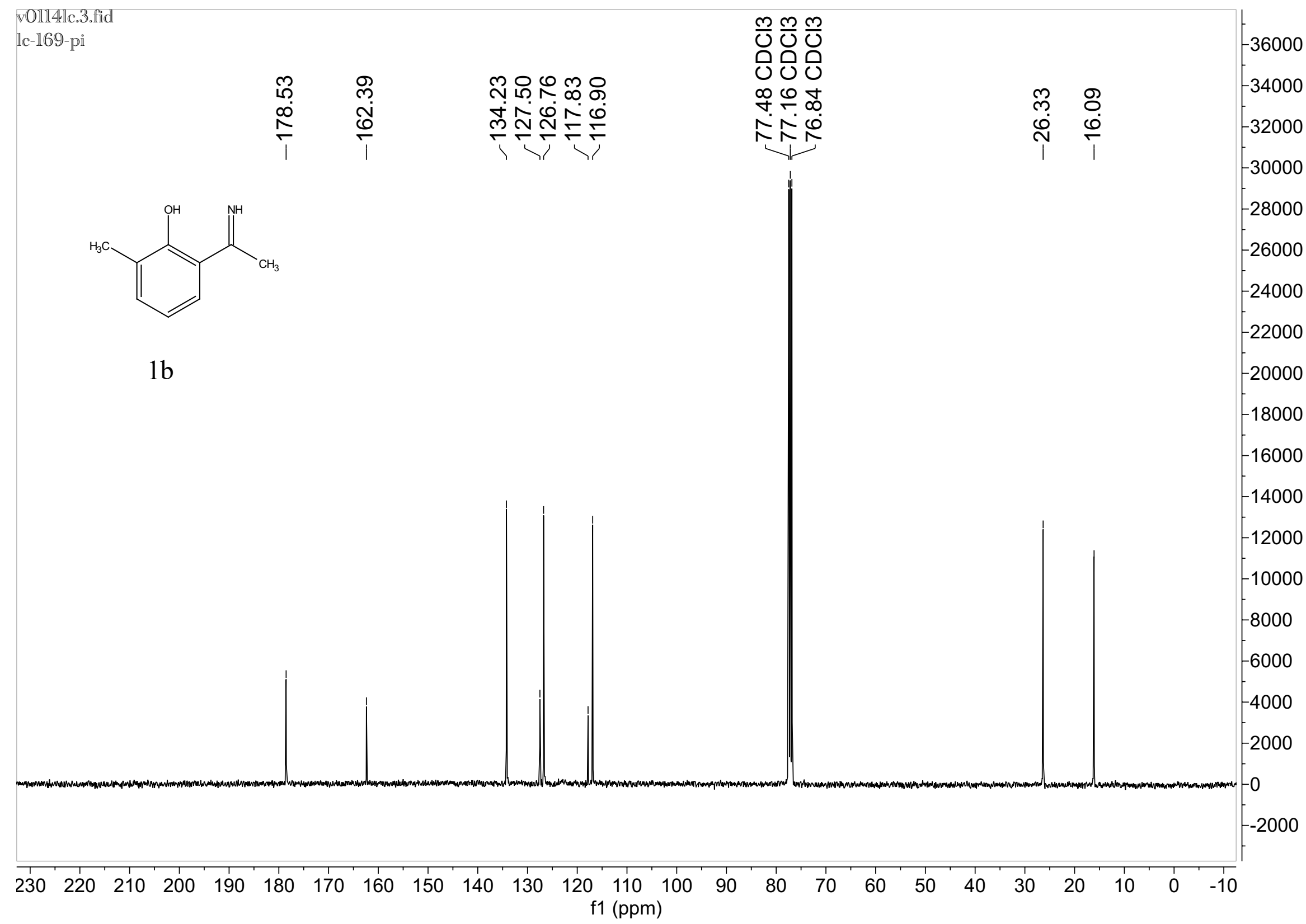
v0114lc.3.fid
lc-169-pi
178.53
162.39
134.23
127.50
126.76
117.83
116.90
77.48 CDCl3
77.16 CDCl3
76.84 CDCl3
26.33
16.09
OH
NH
H3C
CH3
1b
f1 (ppm)

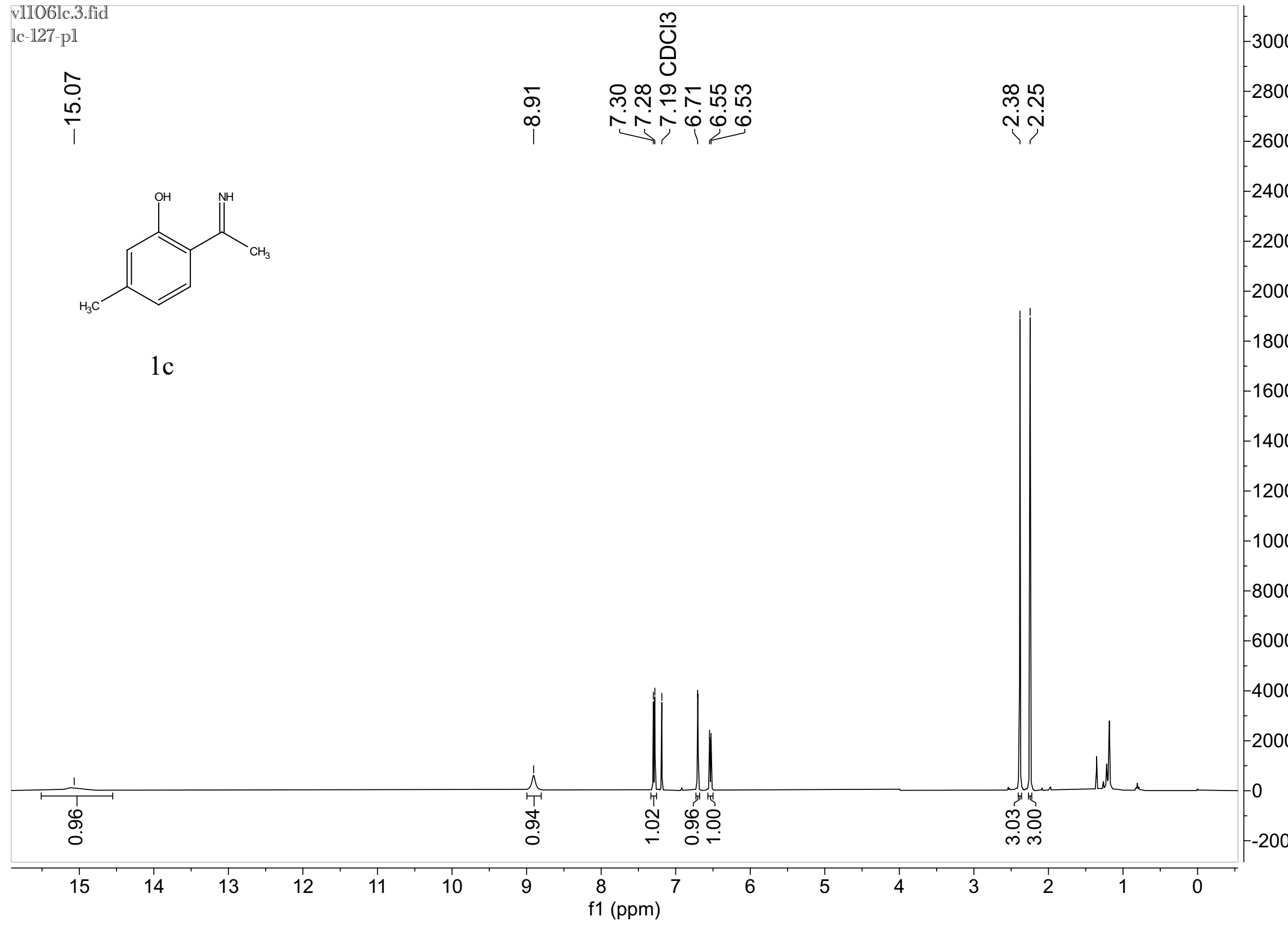
v1106lc.3.fid
lc-127-p1
15.07
8.91
7.30
7.28
7.19 CDCl3
6.71
6.55
6.53
2.38
2.25
OH
NH
CH3
H3C
1c
0.96
0.94
1.02
0.96
1.00
3.03
3.00
f1 (ppm)

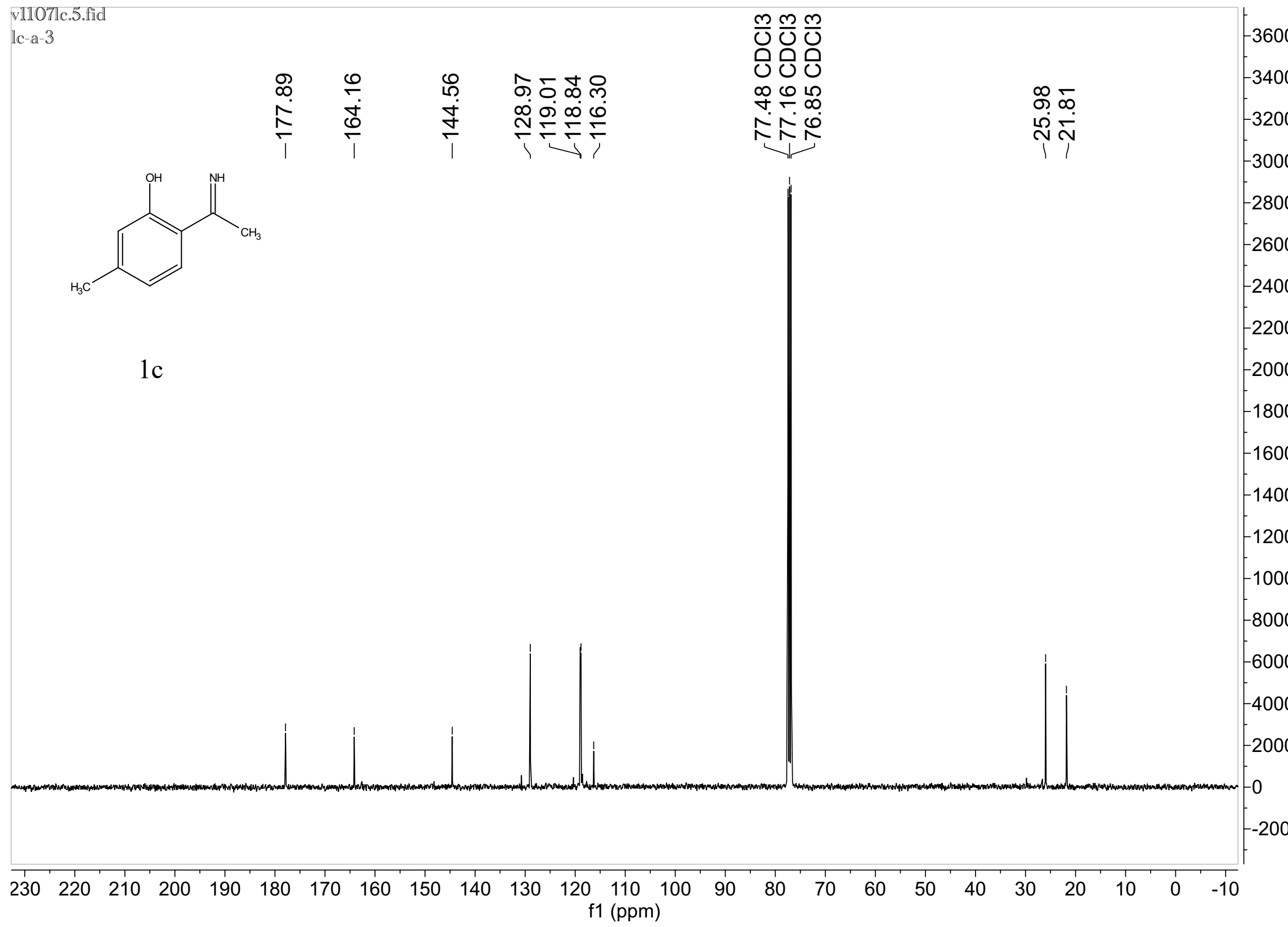
v1107lc.5.fid
lc-a-3
177.89
164.16
144.56
128.97
119.01
118.84
116.30
77.48 CDCl3
77.16 CDCl3
76.85 CDCl3
25.98
21.81
OH
NH
CH3
H3C
1c
f1 (ppm)

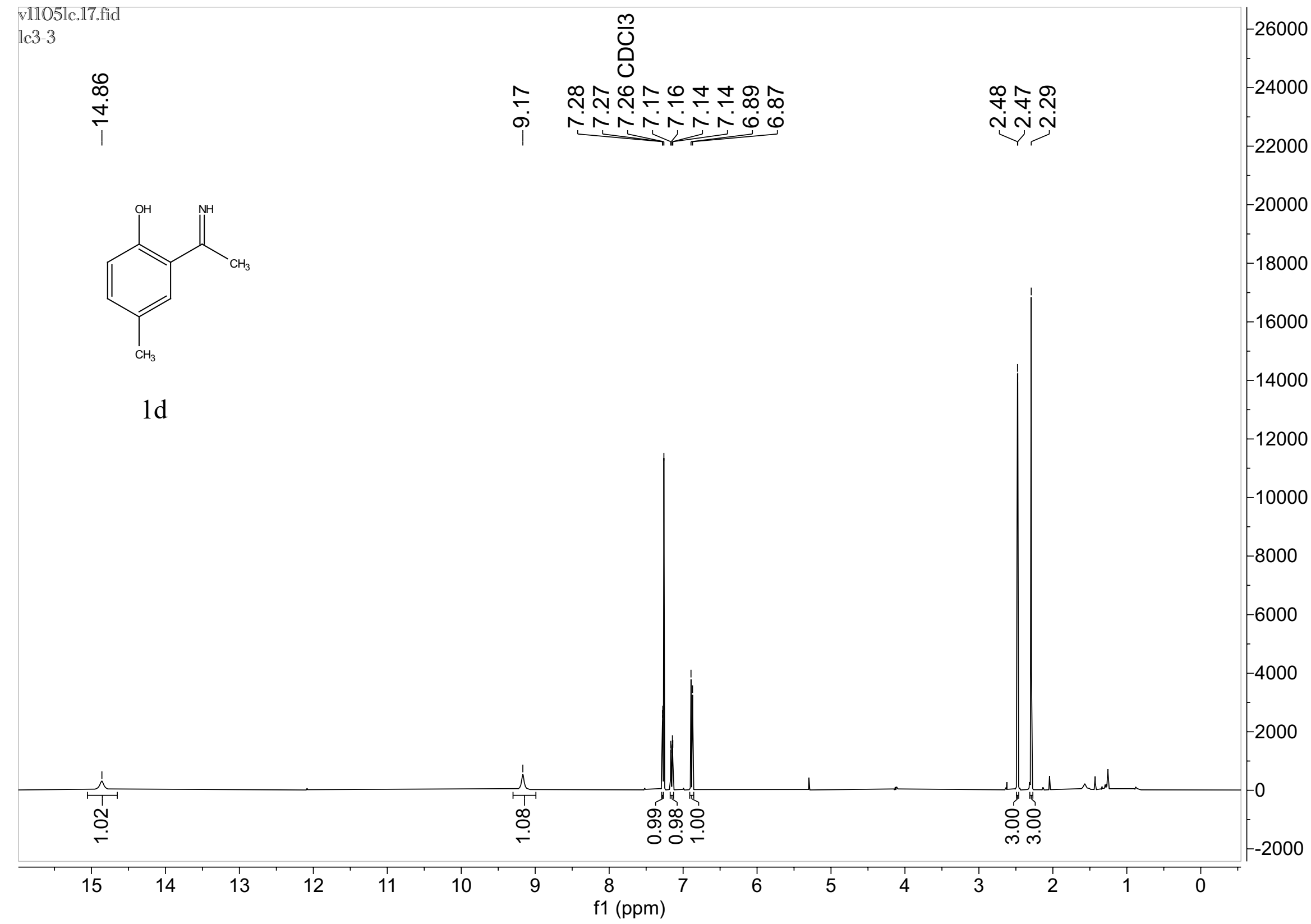
v1105lc.17.fid
lc3-3
14.86
9.17
7.28
7.27
7.26 CDCl3
7.17
7.16
7.14
7.14
6.89
6.87
2.48
2.47
2.29
OH
NH
CH3
CH3
1d
1.02
1.08
0.99
0.98
1.00
3.00
3.00
f1 (ppm)

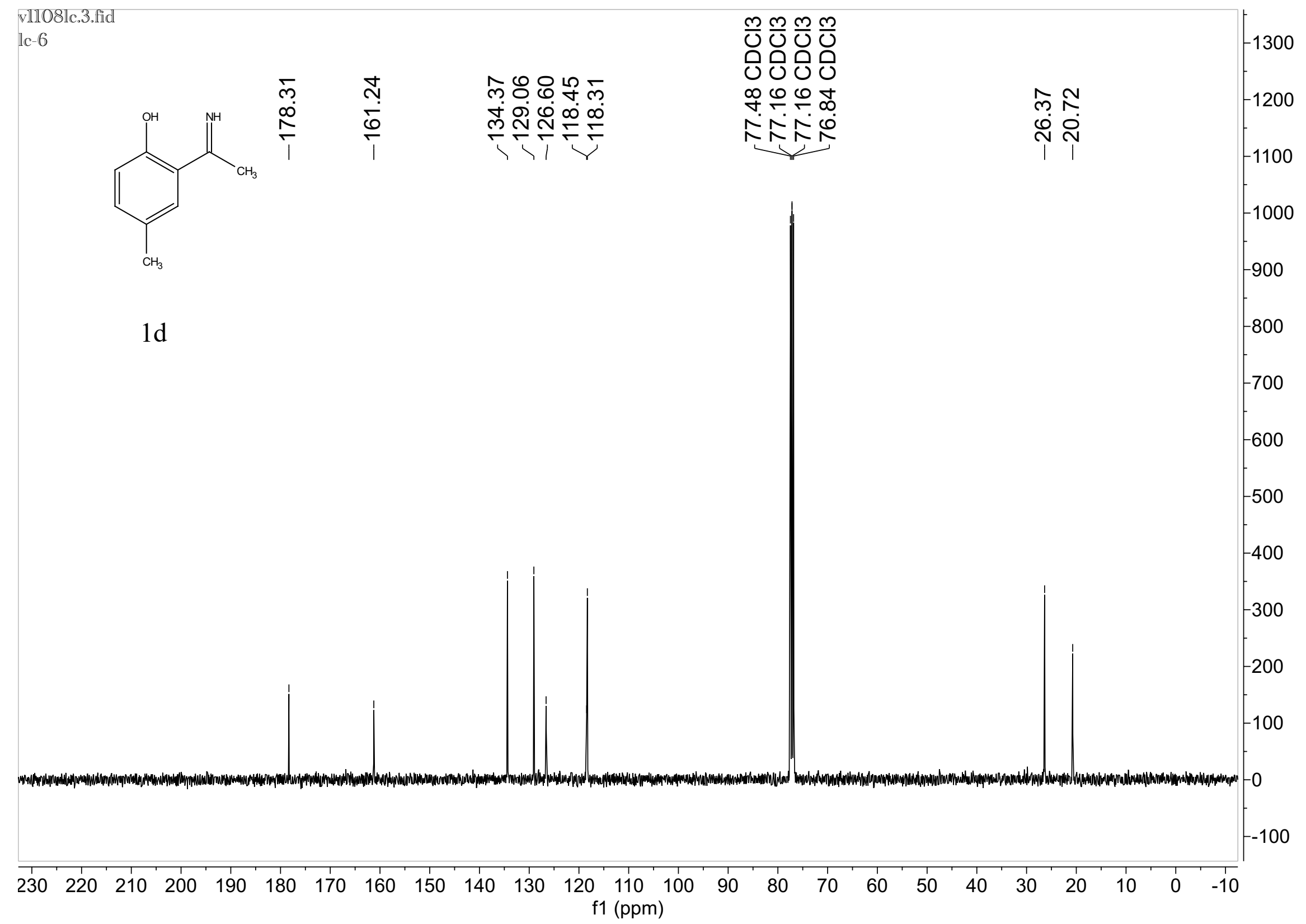
v1108lc.3.fid
lc-6
178.31
161.24
134.37
129.06
126.60
118.45
118.31
77.48 CDCl3
77.16 CDCl3
77.16 CDCl3
76.84 CDCl3
26.37
20.72
OH
NH
CH3
CH3
1d
f1 (ppm)

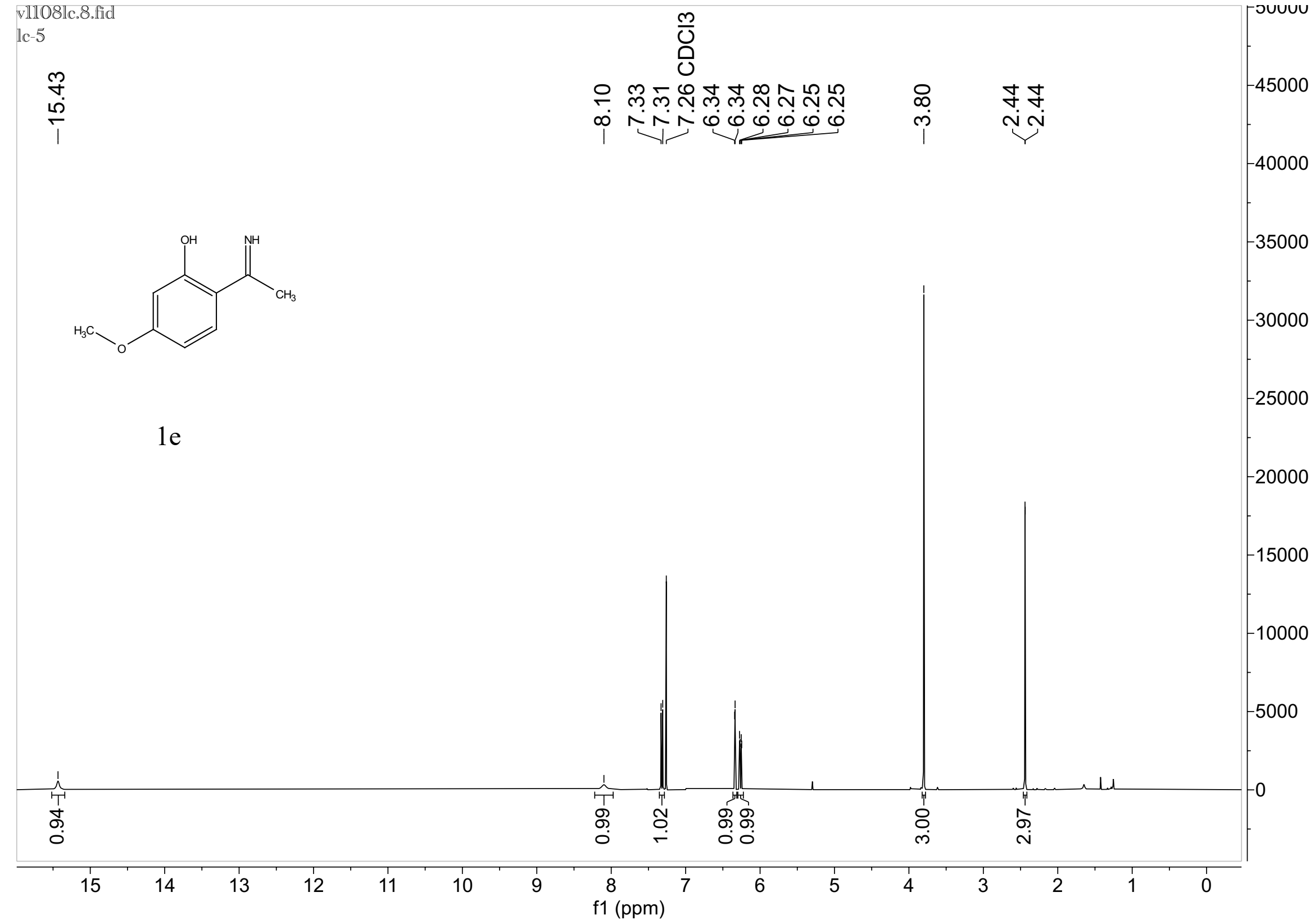
vl108lc.8.fid
lc-5
15.43
8.10
7.33
7.31
7.26 CDCl3
6.34
6.34
6.28
6.27
6.25
6.25
3.80
2.44
2.44
OH
NH
CH3
H3C
O
0.94
0.99
1.02
0.99
0.99
3.00
2.97
f1 (ppm)

1e

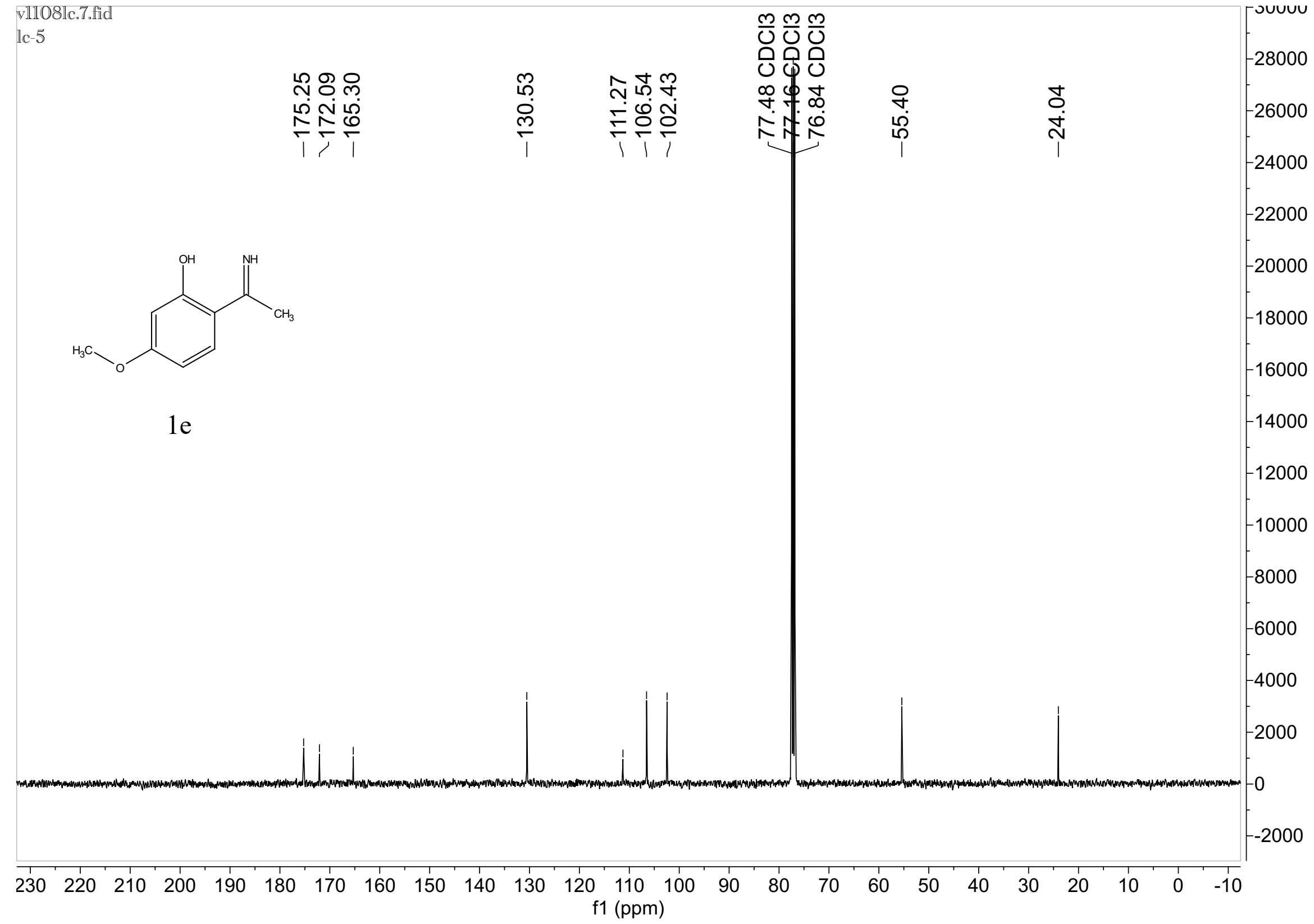
vl108lc.7.fid
lc-5
175.25
172.09
165.30
130.53
111.27
106.54
102.43
77.48 CDCl3
77.16 CDCl3
76.84 CDCl3
55.40
24.04
OH
NH
CH3
H3C
O
f1 (ppm)

1e

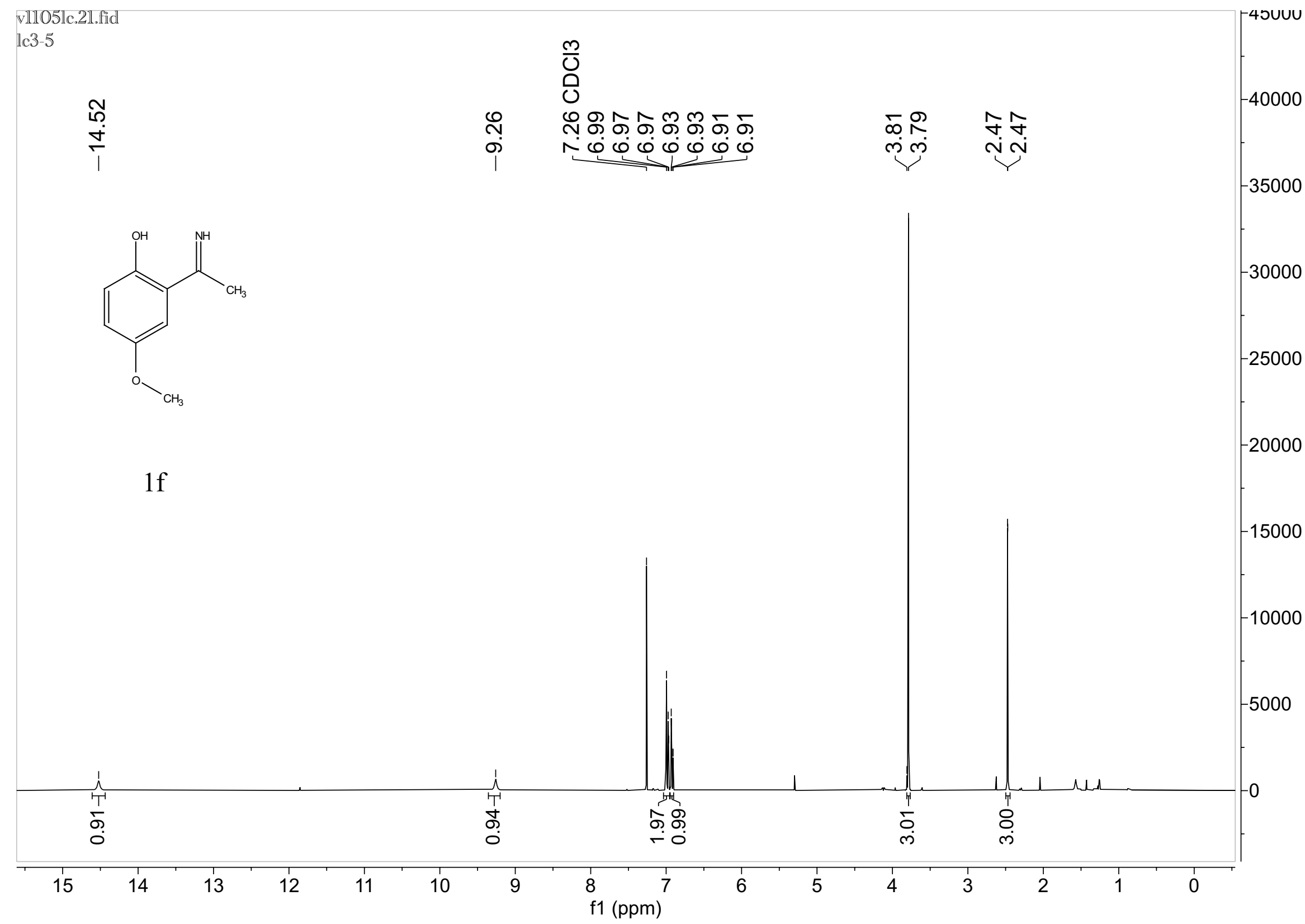
v1105lc.21.fid
lc3-5
14.52
9.26
7.26 CDCl3
6.99
6.97
6.97
6.93
6.93
6.91
6.91
3.81
3.79
2.47
2.47
OH
NH
CH3
O
CH3
1f
0.91
0.94
1.97
0.99
3.01
3.00
f1 (ppm)

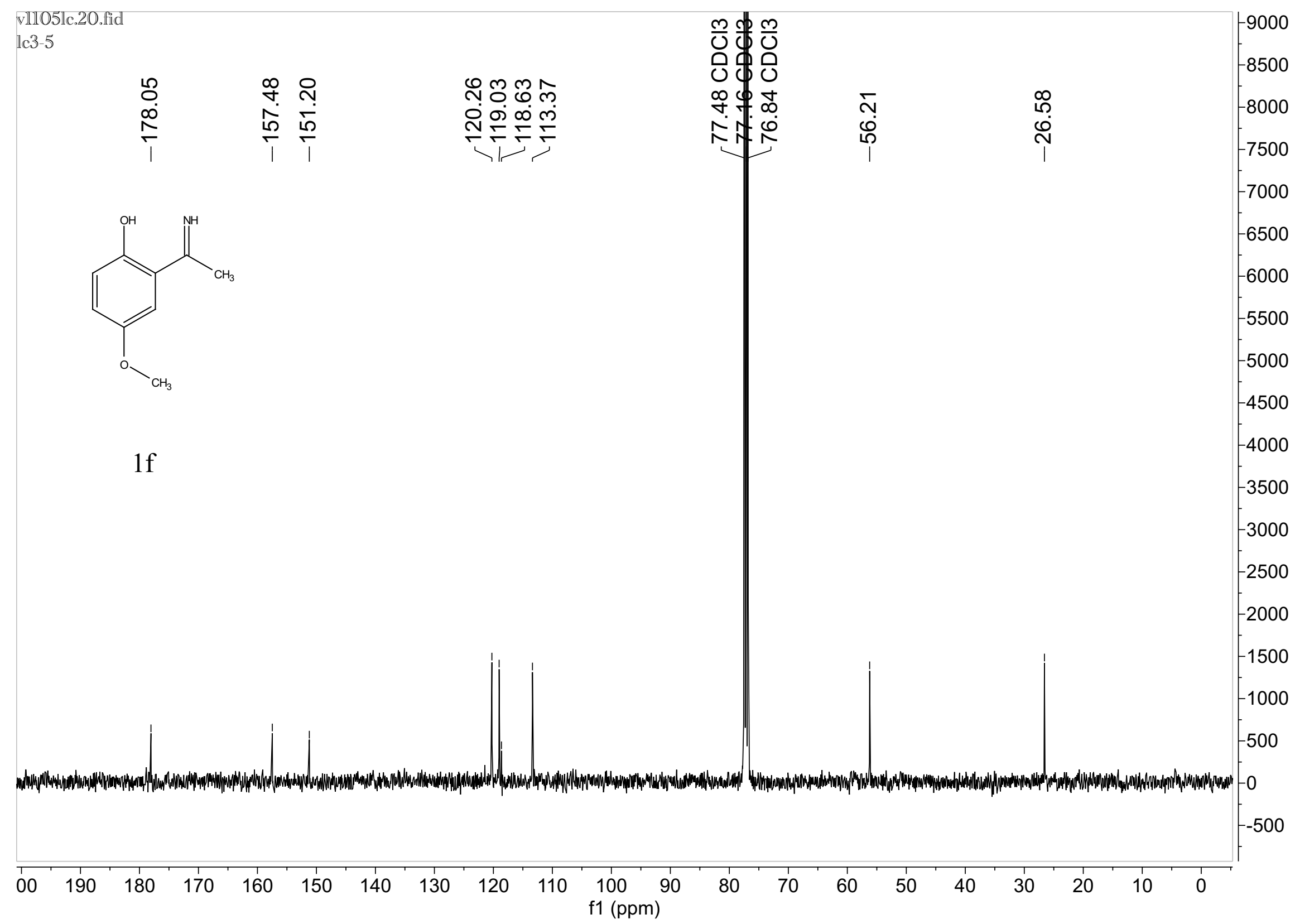
v1105lc.20.fid
lc3-5
178.05
157.48
151.20
120.26
119.03
118.63
113.37
77.48 CDCl3
77.16 CDCl3
76.84 CDCl3
56.21
26.58
OH
NH
CH3
O
CH3
1f
f1 (ppm)

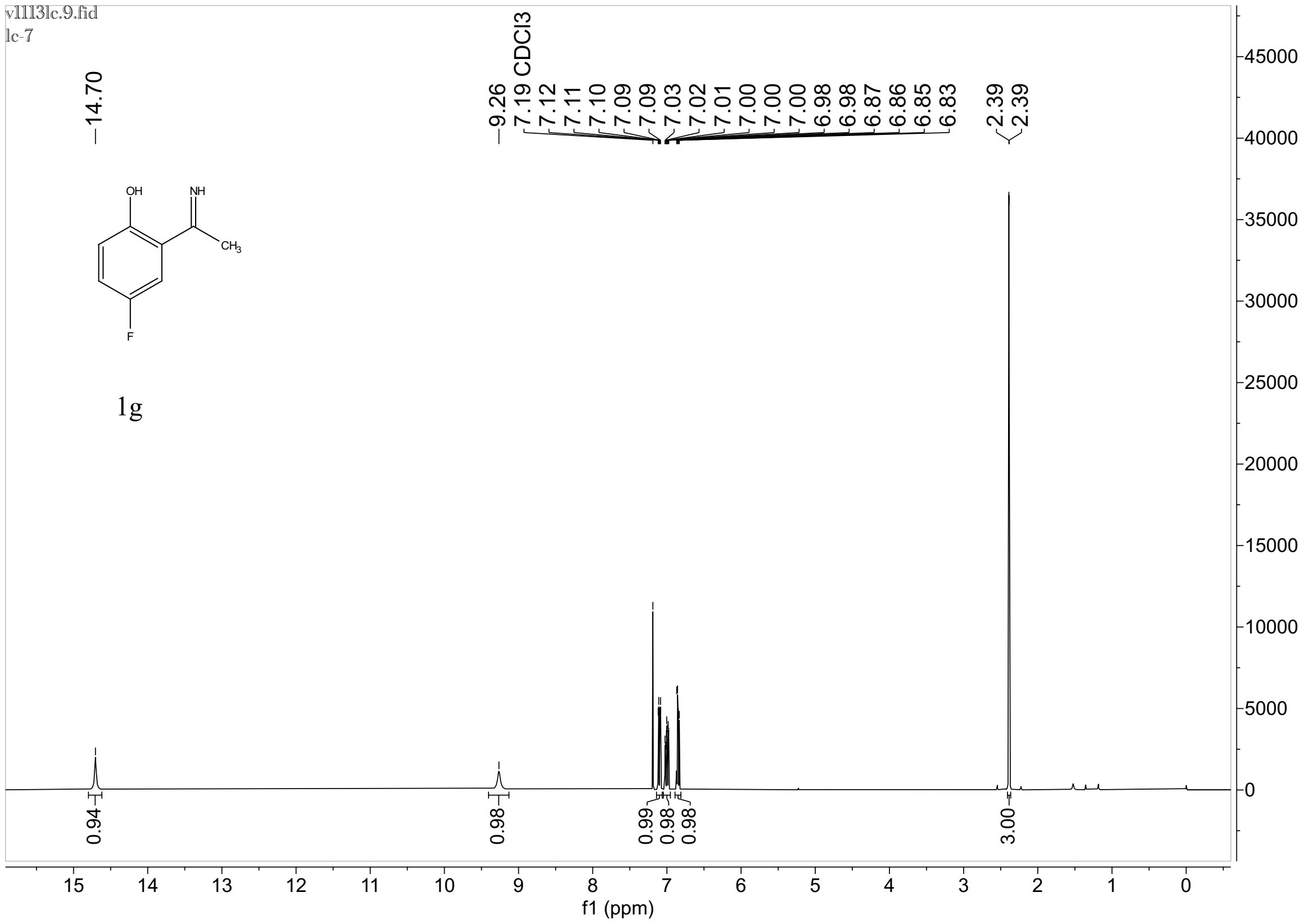
v1113lc.9.fid
lc-7
1g
14.70
9.26
7.19 CDCl3
7.12
7.11
7.10
7.09
7.09
7.03
7.02
7.01
7.00
7.00
6.98
6.98
6.87
6.86
6.85
6.83
2.39
2.39
0.94
0.98
0.99
0.98
0.98
3.00
f1 (ppm)

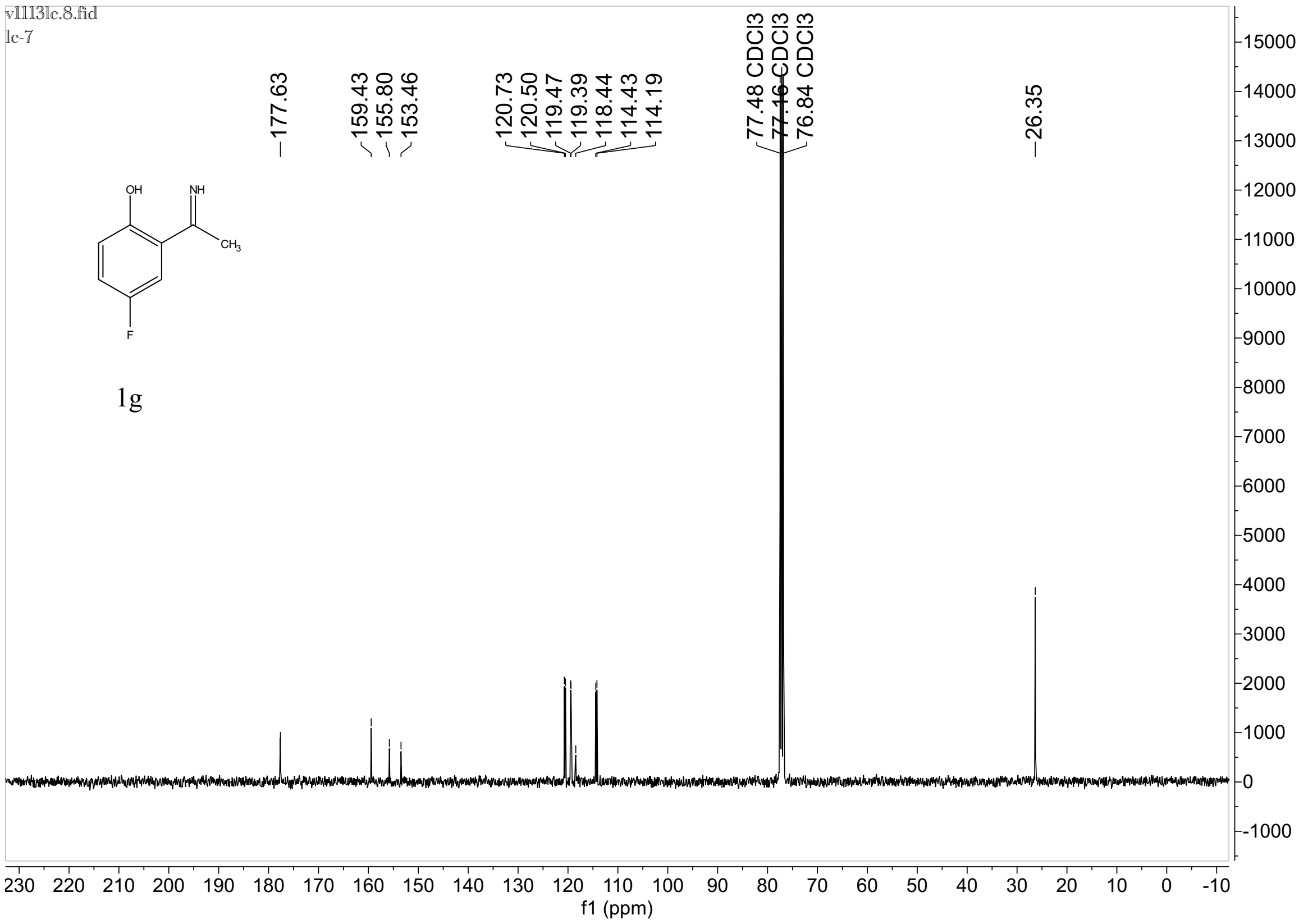
v1113lc.8.fid
lc-7
1g
177.63
159.43
155.80
153.46
120.73
120.50
119.47
119.39
118.44
114.43
114.19
77.48 CDCl3
77.16 CDCl3
76.84 CDCl3
26.35
f1 (ppm)

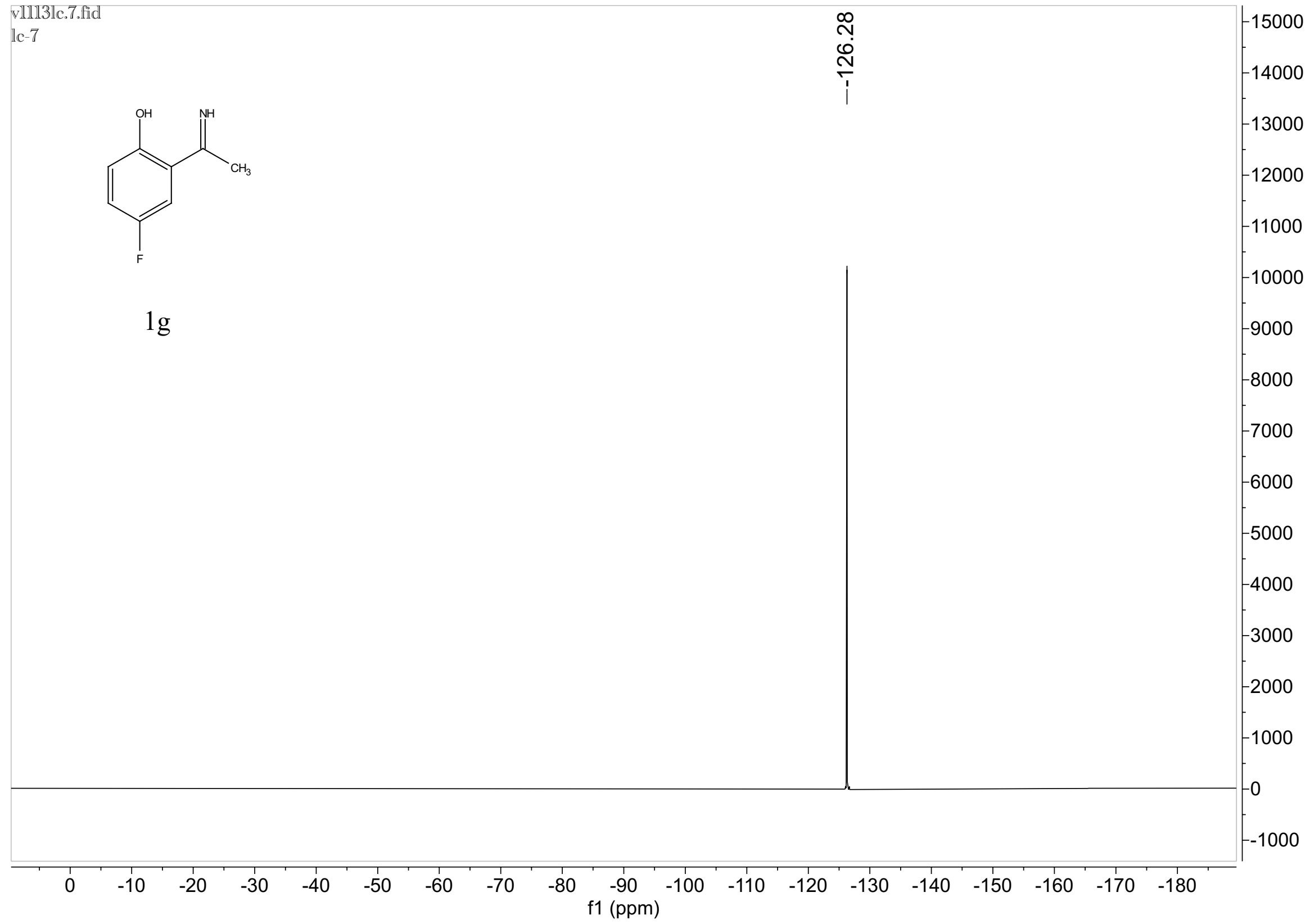

vl113lc.7.fid
lc-7
OH
NH
CH3
F
1g
--126.28
15000
14000
13000
12000
11000
10000
9000
8000
7000
6000
5000
4000
3000
2000
1000
0
-1000
0
-10
-20
-30
-40
-50
-60
-70
-80
-90
-100
-110
-120
-130
-140
-150
-160
-170
-180
f1 (ppm)

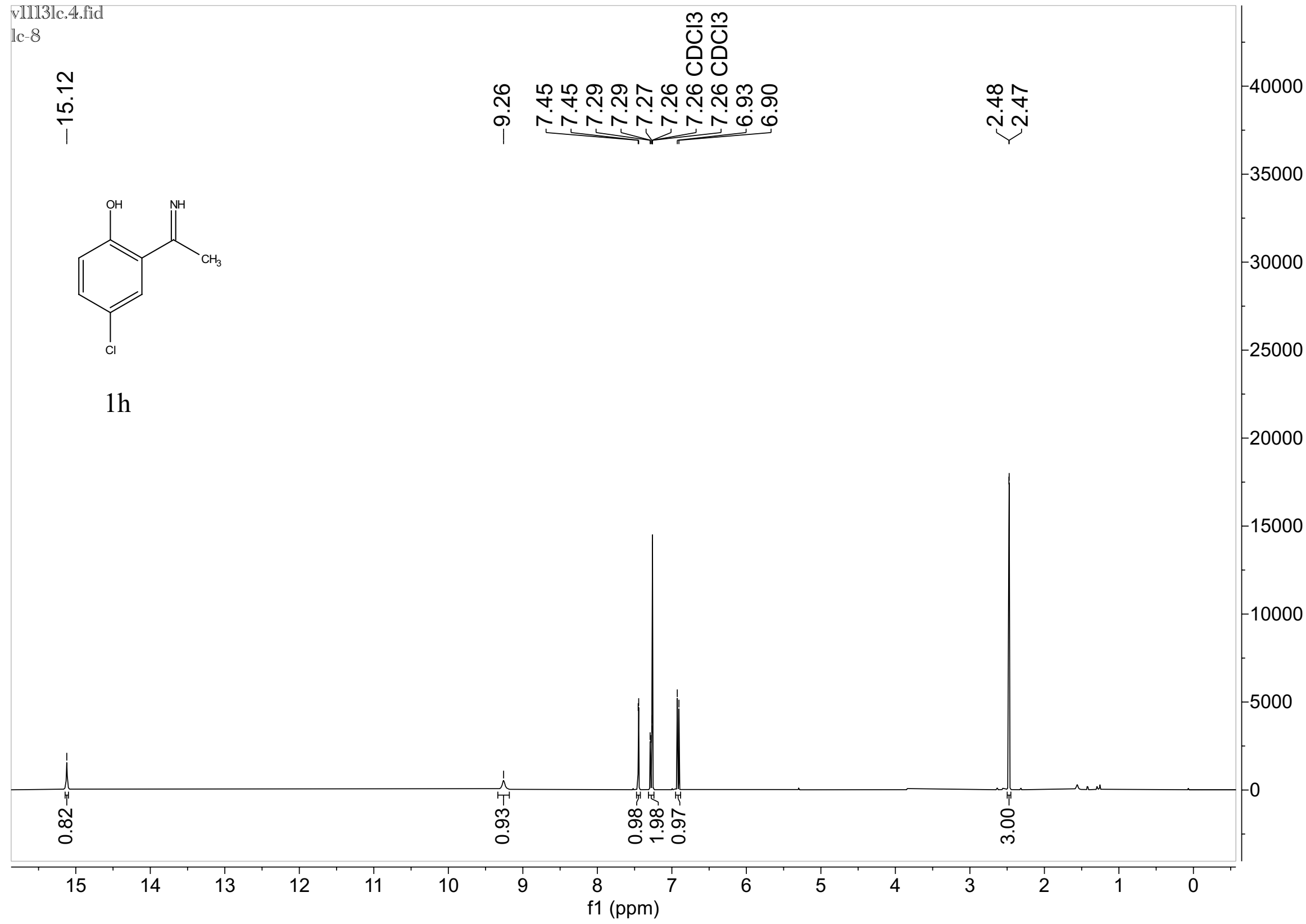
vl113lc.4.fid
lc-8
15.12
9.26
7.45
7.45
7.29
7.29
7.27
7.26 CDCl3
7.26 CDCl3
7.26 CDCl3
6.93
6.90
2.48
2.47
OH
NH
CH3
Cl
1h
0.82
0.93
0.98
1.98
0.97
3.00
f1 (ppm)

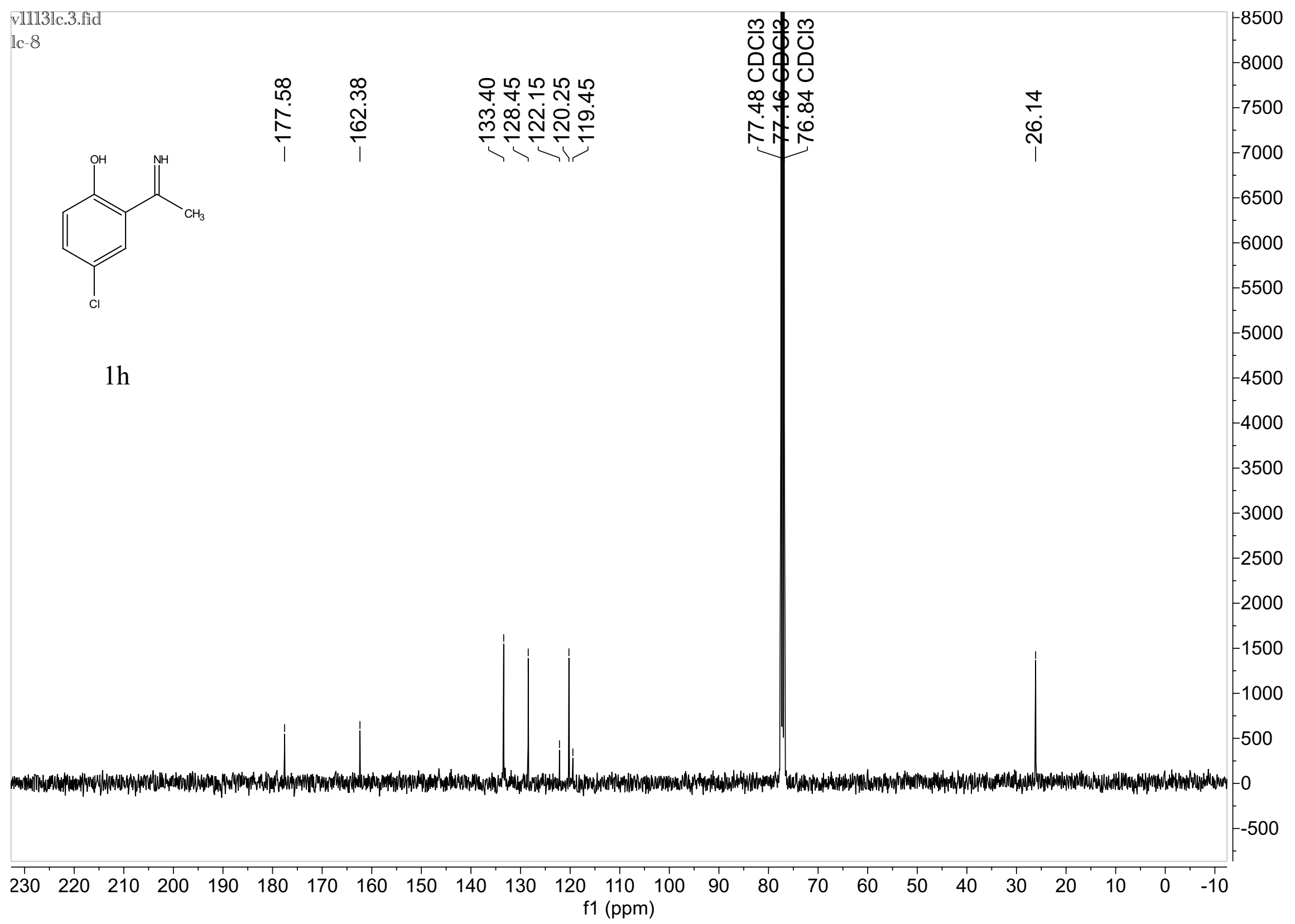
vl113lc.3.fid
lc-8
177.58
162.38
133.40
128.45
122.15
120.25
119.45
77.48 CDCl3
77.16 CDCl3
76.84 CDCl3
26.14
OH
NH
CH3
Cl
1h
f1 (ppm)

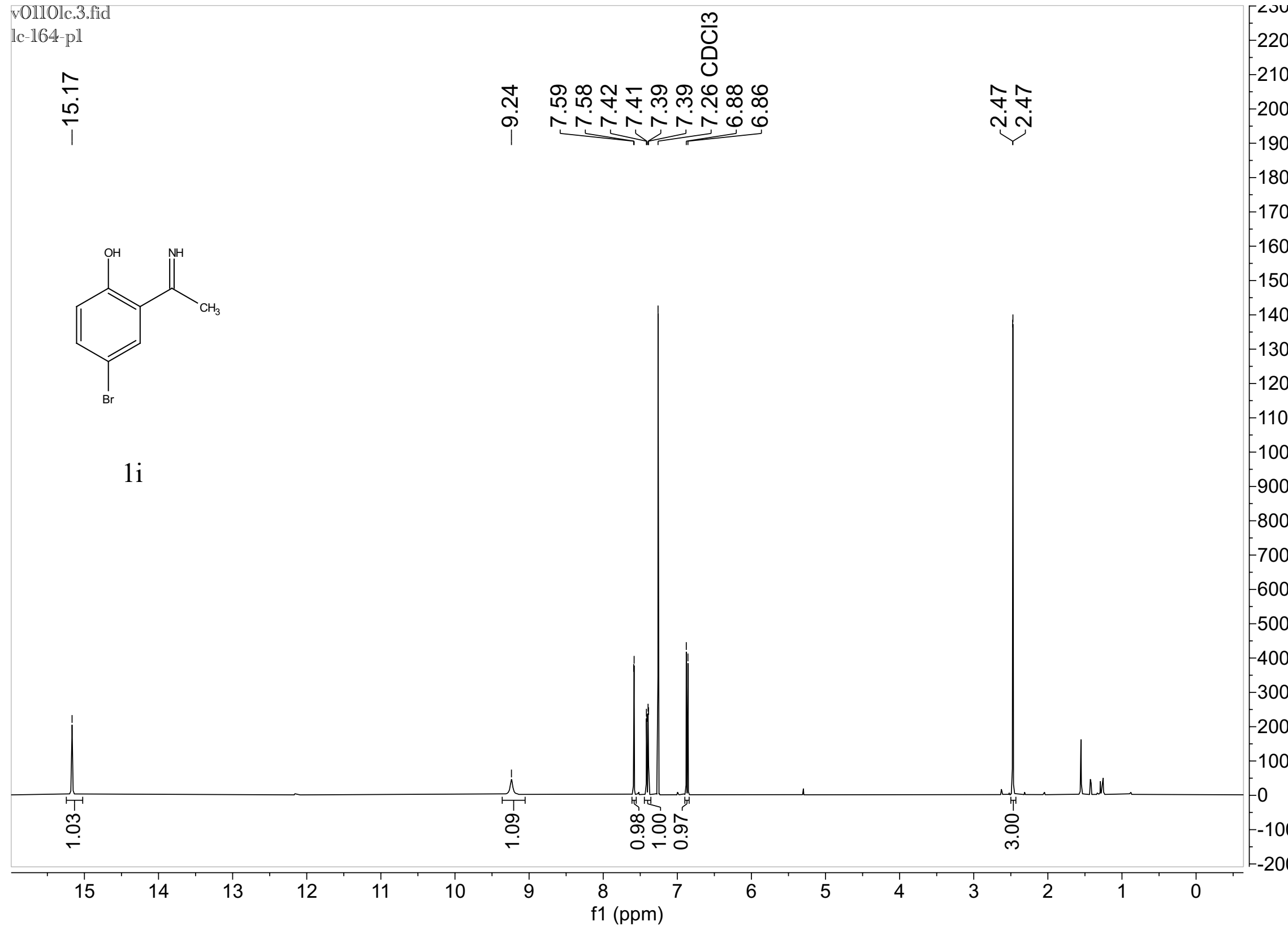
v0110lc.3.fid
lc-164-p1
15.17
9.24
7.59
7.58
7.42
7.41
7.39
7.39
7.26 CDCl3
6.88
6.86
2.47
2.47
OH
NH
CH3
Br
1i
1.03
1.09
0.98
1.00
0.97
3.00
f1 (ppm)

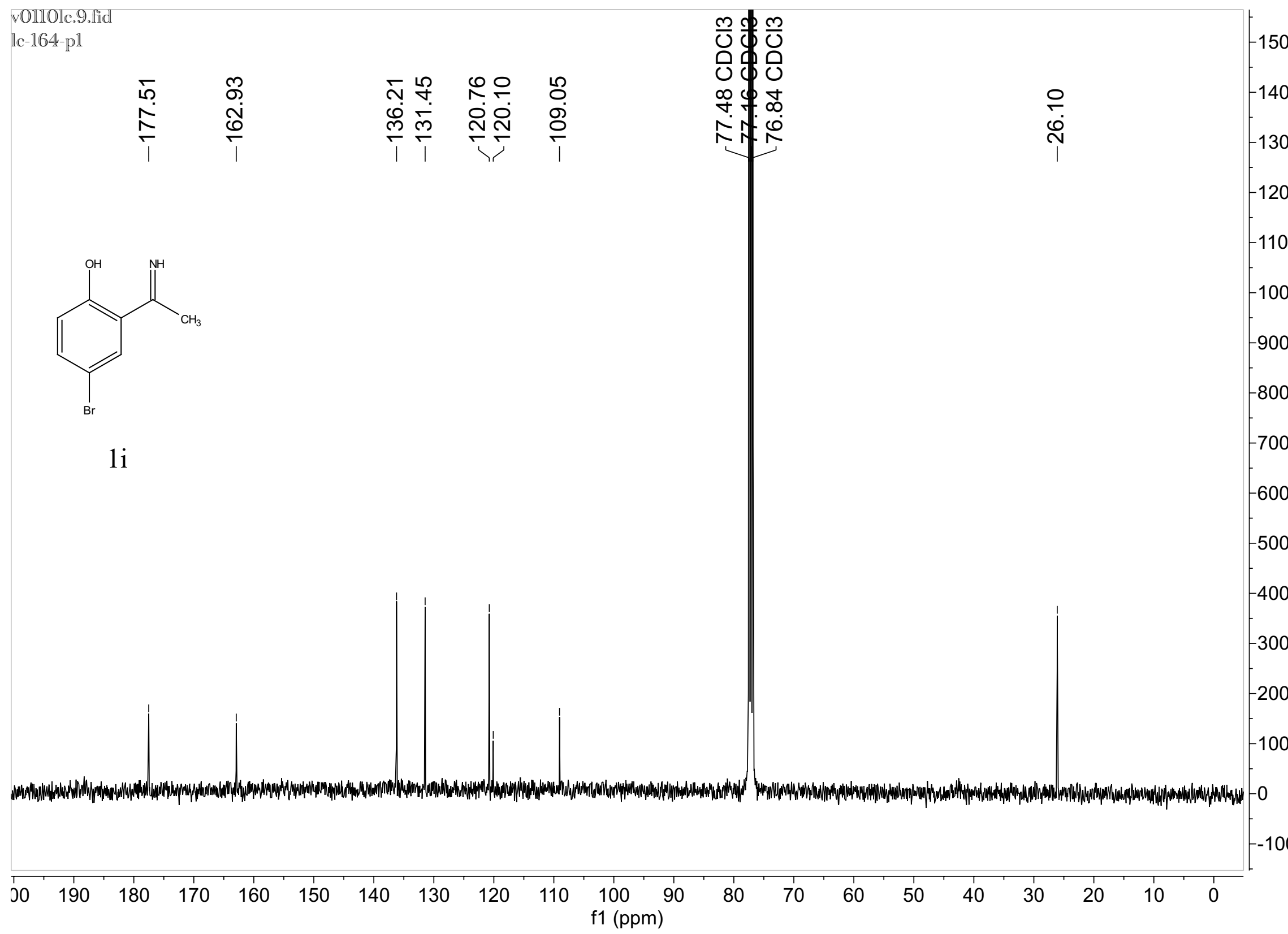
v0110lc.9.fid
lc-164-p1
177.51
162.93
136.21
131.45
120.76
120.10
109.05
77.48 CDCl3
77.16 CDCl3
76.84 CDCl3
26.10
OH
NH
CH3
Br
1i
f1 (ppm)

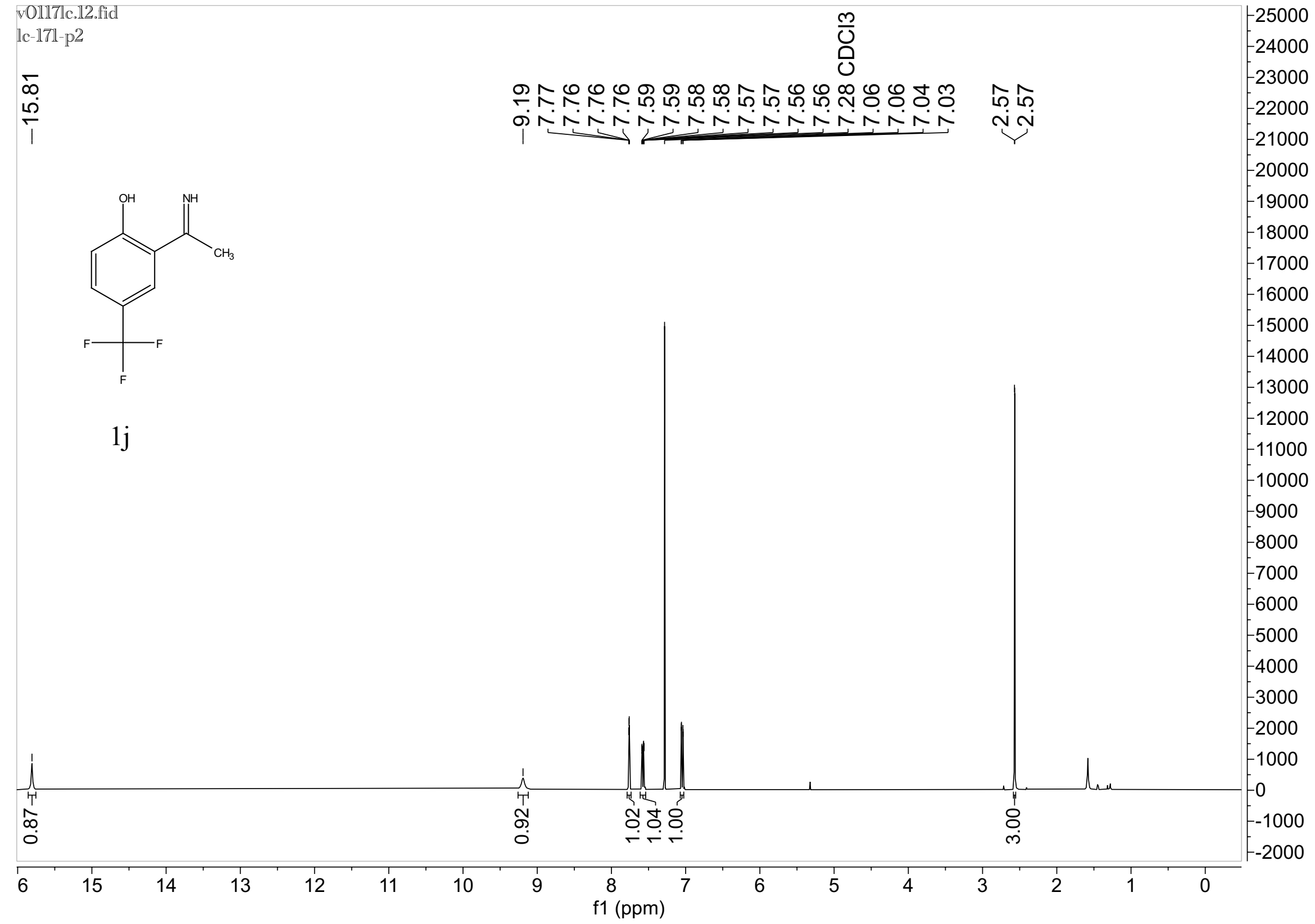

v0117lc.12.fid
lc-171-p2
15.81
9.19
7.77
7.76
7.76
7.76
7.59
7.59
7.58
7.57
7.57
7.56
7.56
7.28 CDCl3
7.06
7.06
7.04
7.03
2.57
2.57
OH
NH
CH3
F
F
F
1j
0.87
0.92
1.02
1.04
1.00
3.00
f1 (ppm)

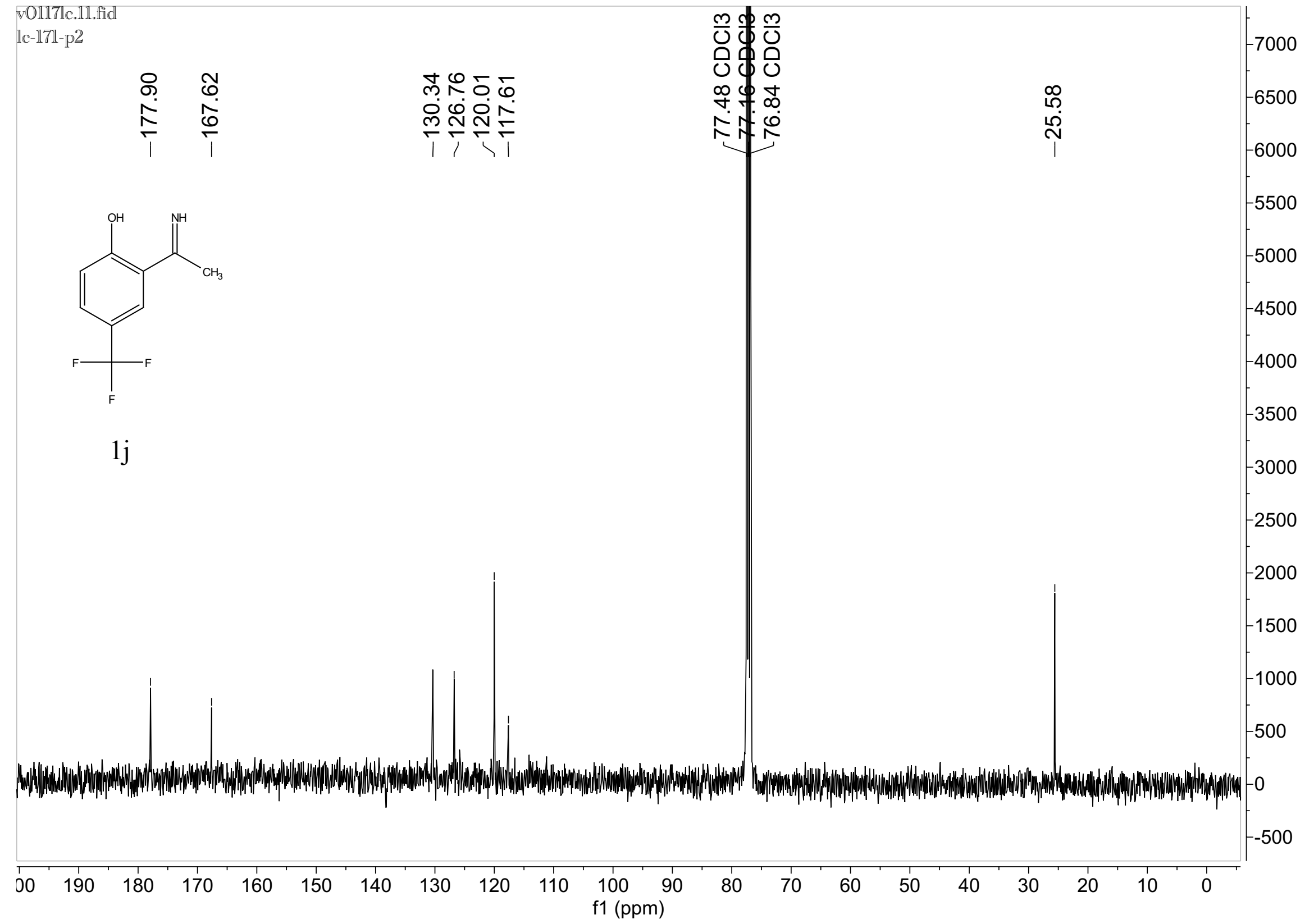

v0117lc.11.fid
lc-171-p2
177.90
167.62
130.34
126.76
120.01
117.61
77.48 CDCl3
77.16 CDCl3
76.84 CDCl3
25.58
OH
NH
CH3
F
F
F
1j
f1 (ppm)

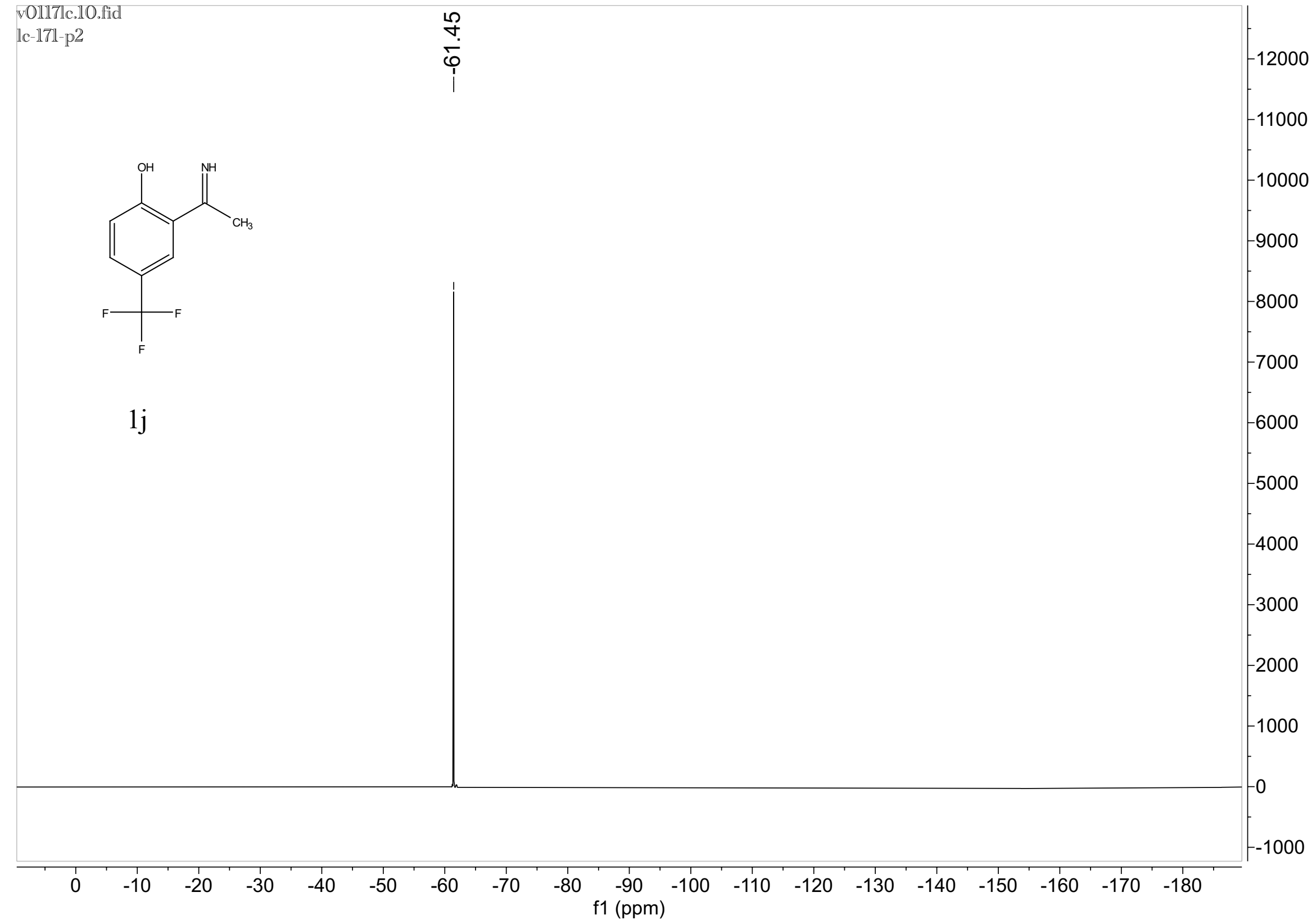
v0117lc.10.fid
lc-171-p2
-61.45
OH
NH
CH3
F
F
F
1j
12000
11000
10000
9000
8000
7000
6000
5000
4000
3000
2000
1000
0
-1000
0 -10 -20 -30 -40 -50 -60 -70 -80 -90 -100 -110 -120 -130 -140 -150 -160 -170 -180
f1 (ppm)

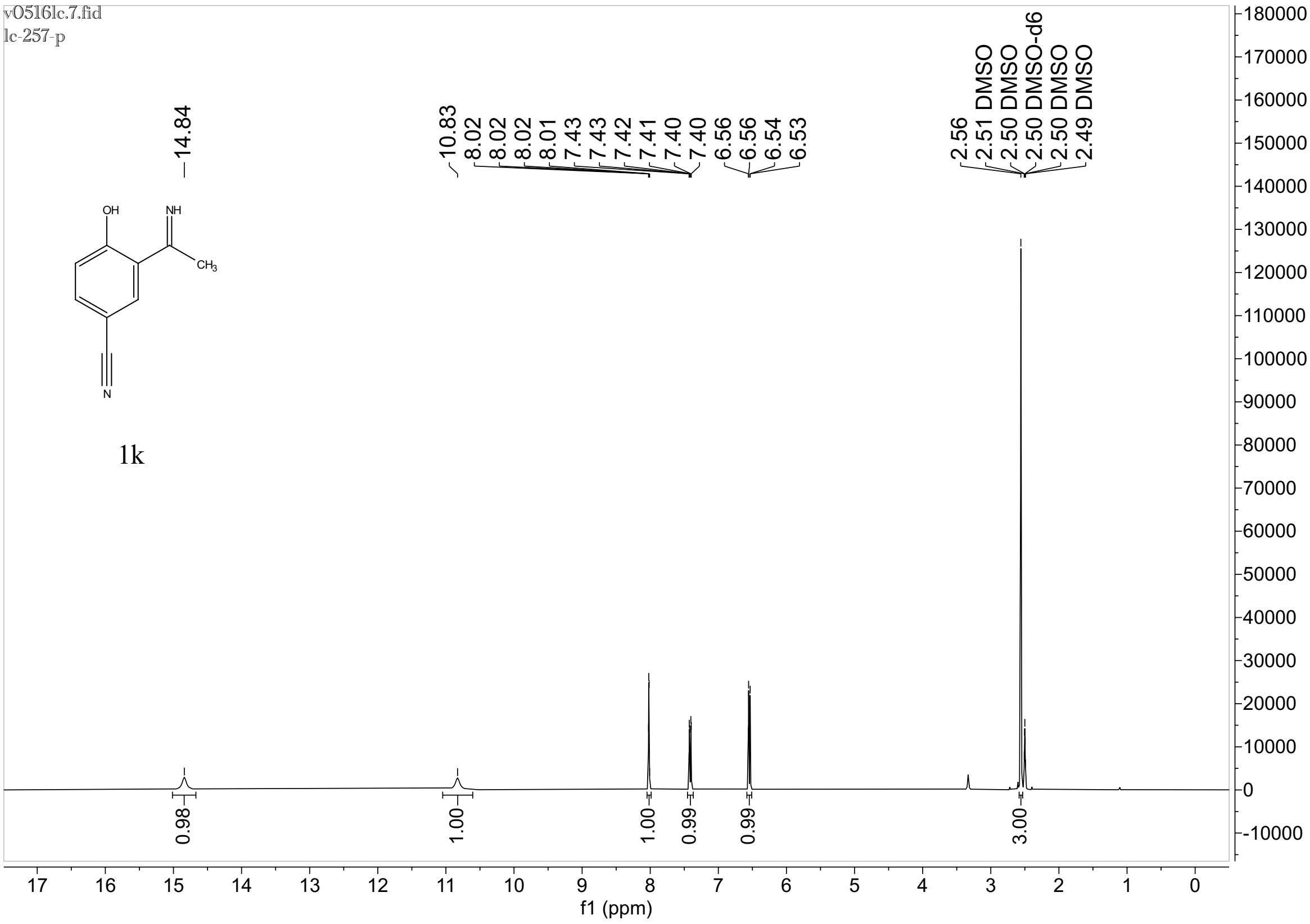
v0516lc.7.fid
lc-257-p
OH
NH
CH3
N
1k
14.84
10.83
8.02
8.02
8.02
8.01
7.43
7.43
7.42
7.41
7.40
7.40
6.56
6.56
6.54
6.53
2.56
2.51 DMSO
2.50 DMSO
2.50 DMSO-d6
2.50 DMSO
2.49 DMSO
0.98
1.00
1.00
0.99
0.99
3.00
f1 (ppm)

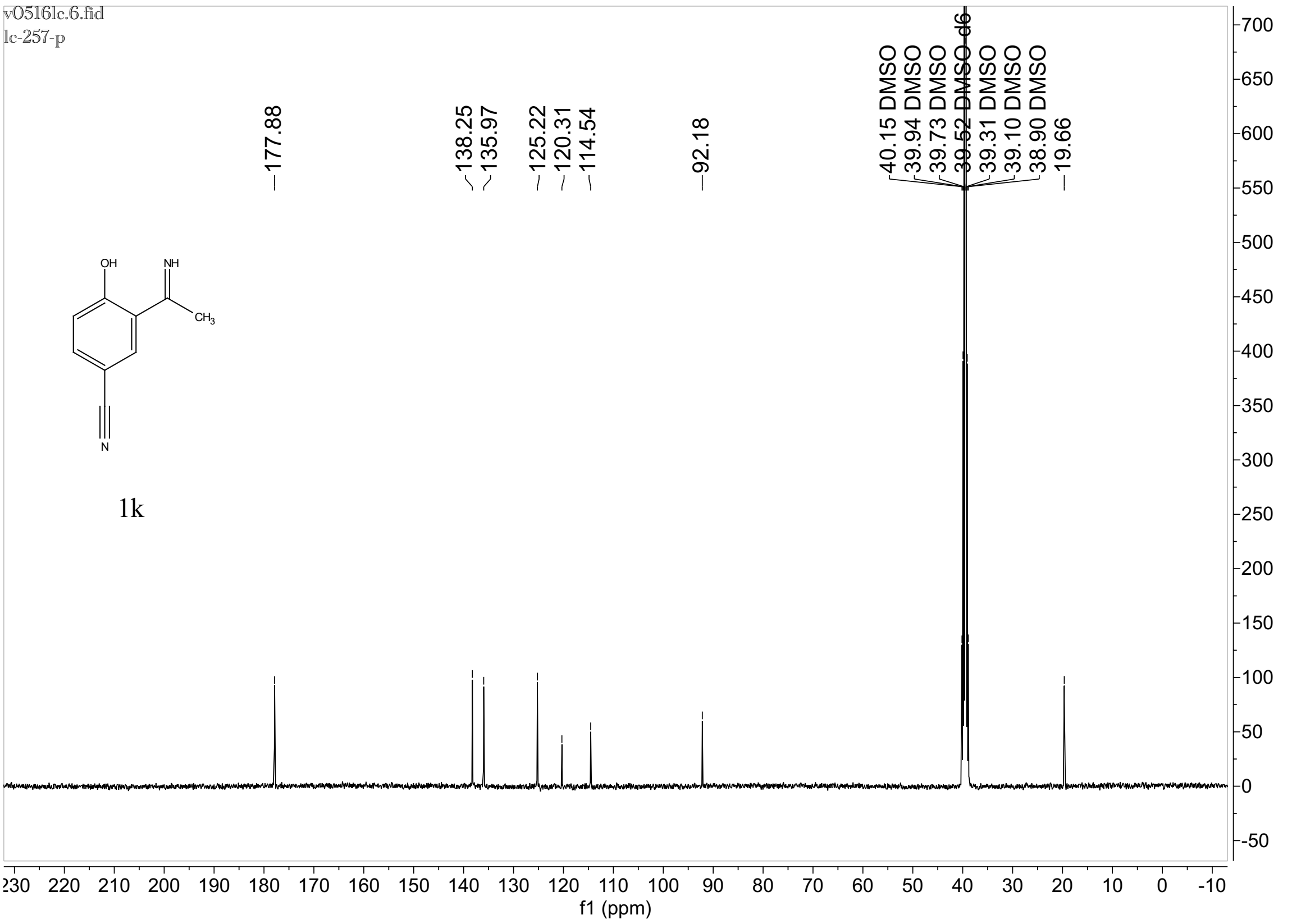
v0516lc.6.fid
lc-257-p
OH
NH
CH3
N
1k
177.88
138.25
135.97
125.22
120.31
114.54
92.18
40.15 DMSO
39.94 DMSO
39.73 DMSO
39.52 DMSO-d6
39.31 DMSO
39.10 DMSO
38.90 DMSO
19.66
f1 (ppm)

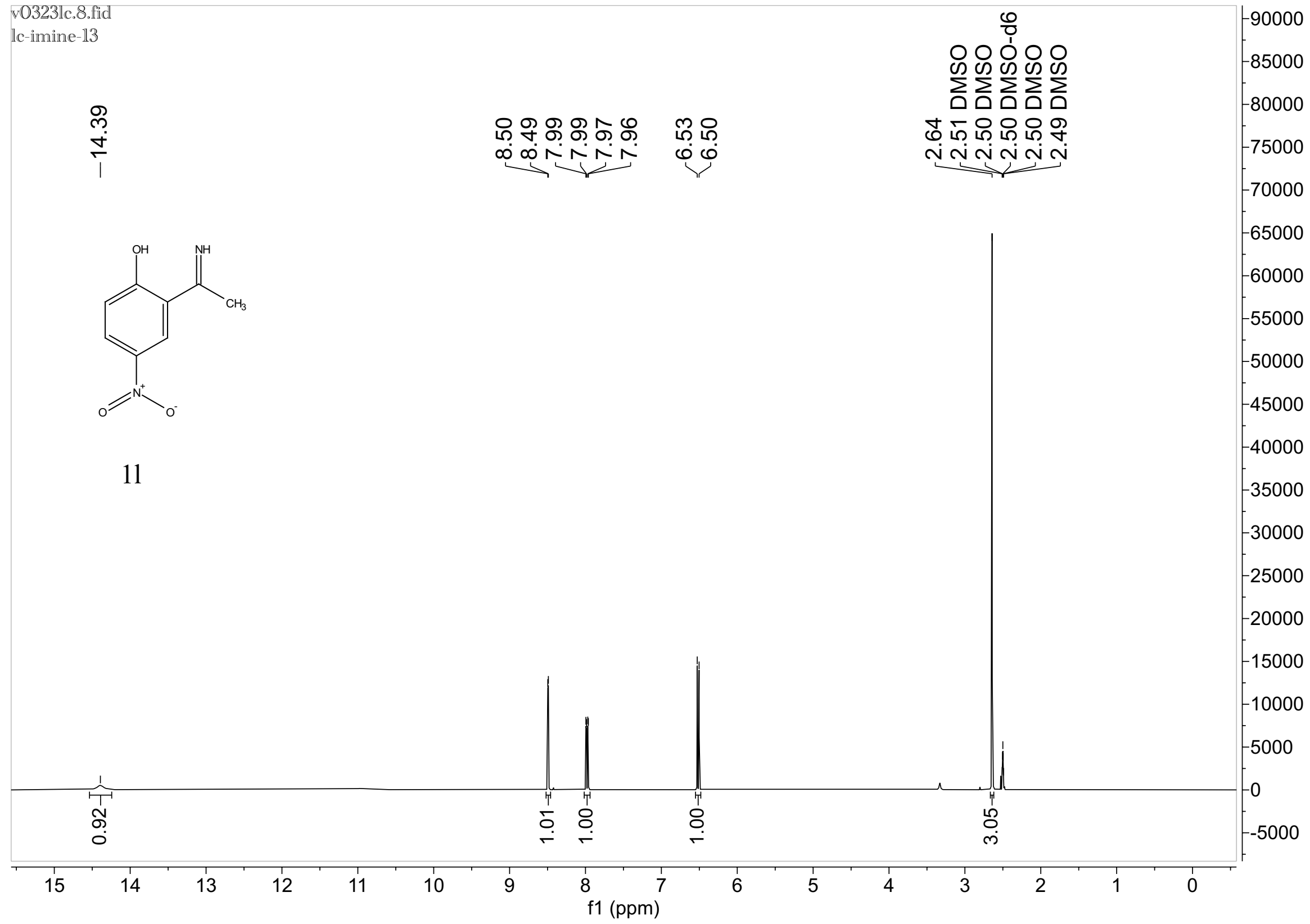
v0323lc.8.fid
lc-imine-13
—14.39
8.50
8.49
7.99
7.99
7.97
7.96
6.53
6.50
2.64
2.51 DMSO
2.50 DMSO
2.50 DMSO-d6
2.50 DMSO
2.49 DMSO
OH
NH
CH3
11
0.92
1.01
1.00
1.00
3.05
f1 (ppm)

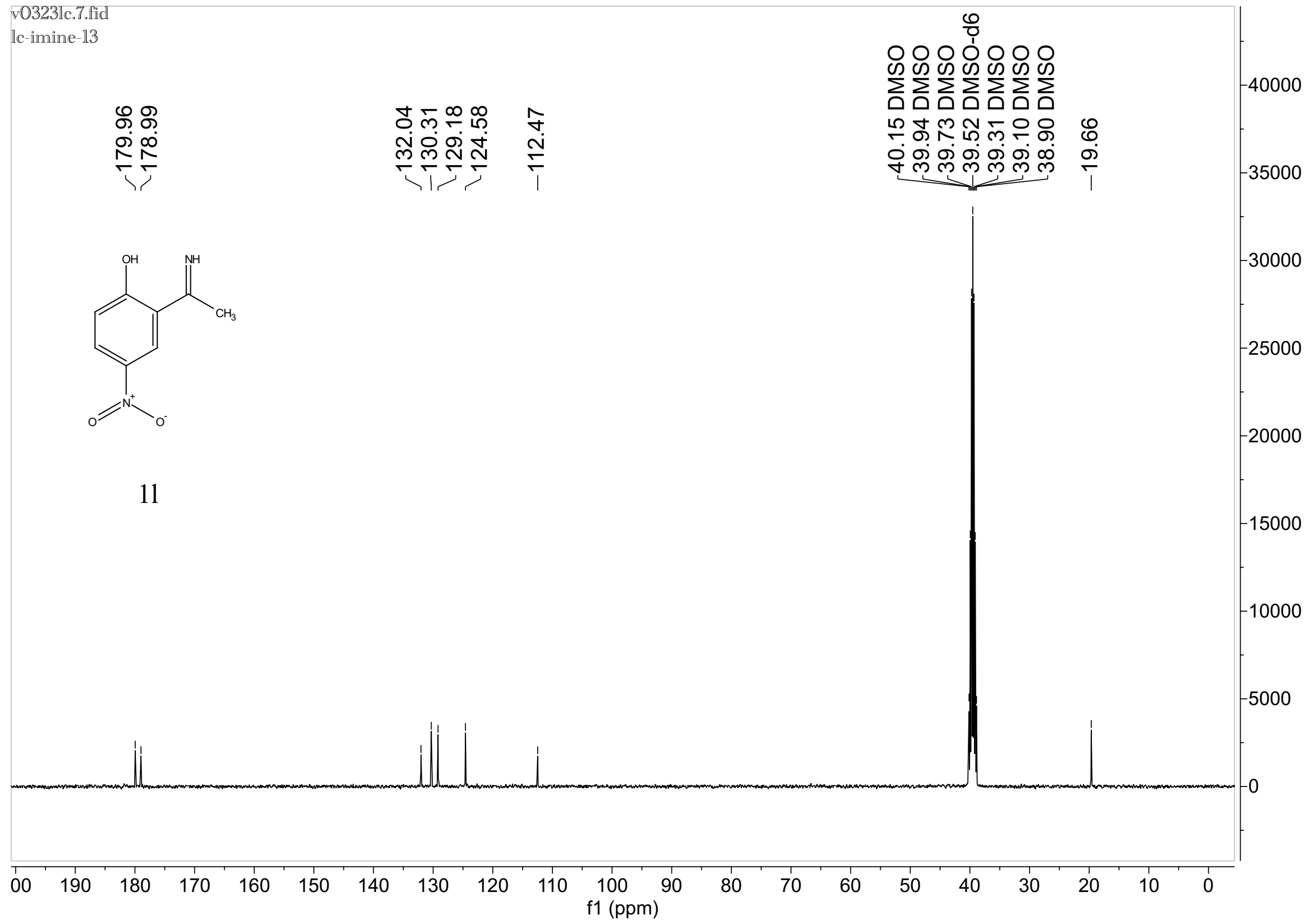
v0323lc.7.fid
lc-imine-13
179.96
178.99
132.04
130.31
129.18
124.58
—112.47
40.15 DMSO
39.94 DMSO
39.73 DMSO
39.52 DMSO-d6
39.31 DMSO
39.10 DMSO
38.90 DMSO
—19.66
OH
NH
CH3
11
f1 (ppm)

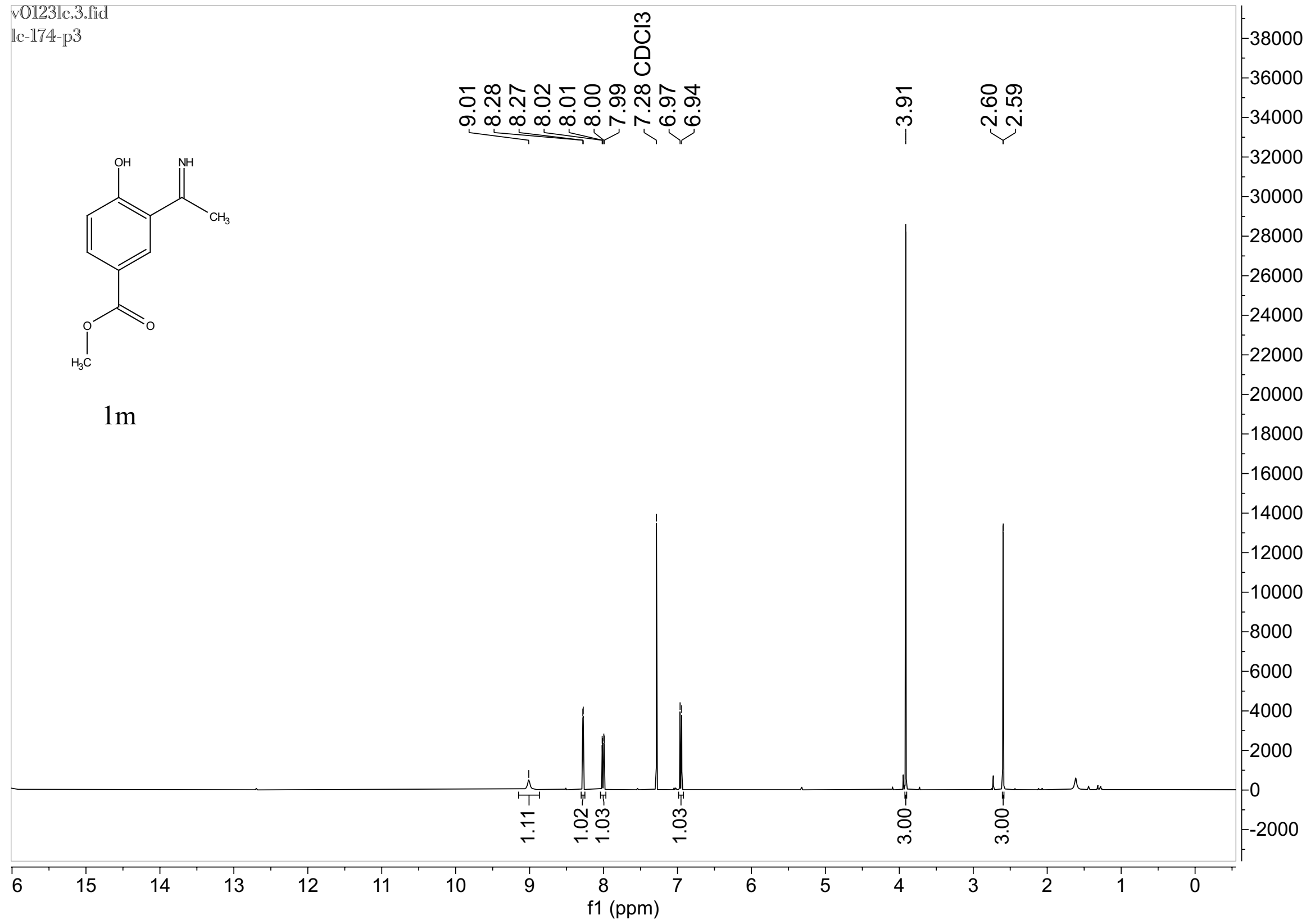
v0123lc.3.fid
lc-174-p3
9.01
8.28
8.27
8.02
8.01
8.00
7.99
7.28 CDCl3
6.97
6.94
3.91
2.60
2.59
OH
NH
CH3
O
O
H3C
1m
1.11
1.02
1.03
1.03
3.00
3.00
f1 (ppm)

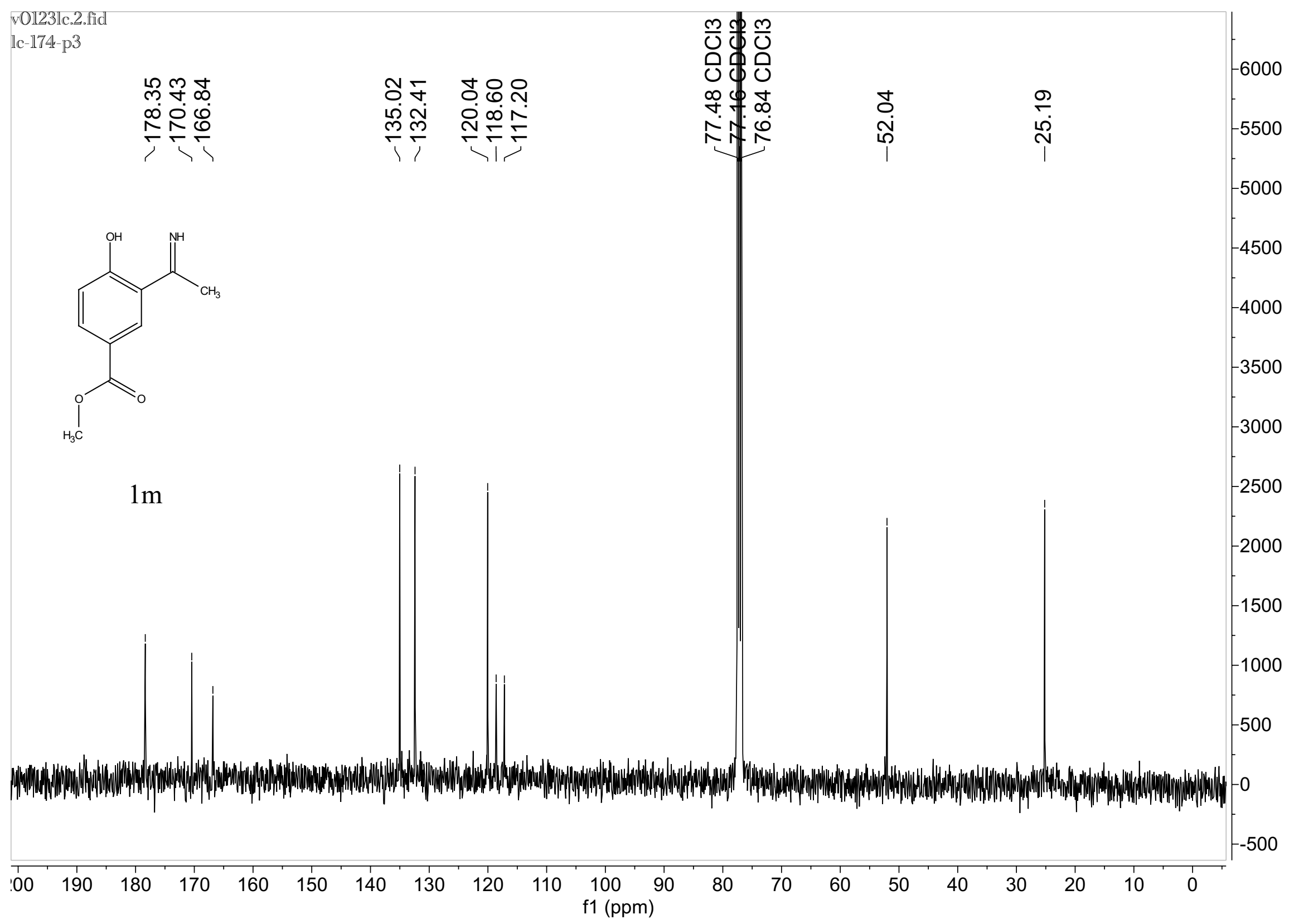
v0123lc.2.fid
lc-174-p3
178.35
170.43
166.84
135.02
132.41
120.04
118.60
117.20
77.48 CDCl3
77.16 CDCl3
76.84 CDCl3
52.04
25.19
OH
NH
CH3
O
O
H3C
1m
f1 (ppm)

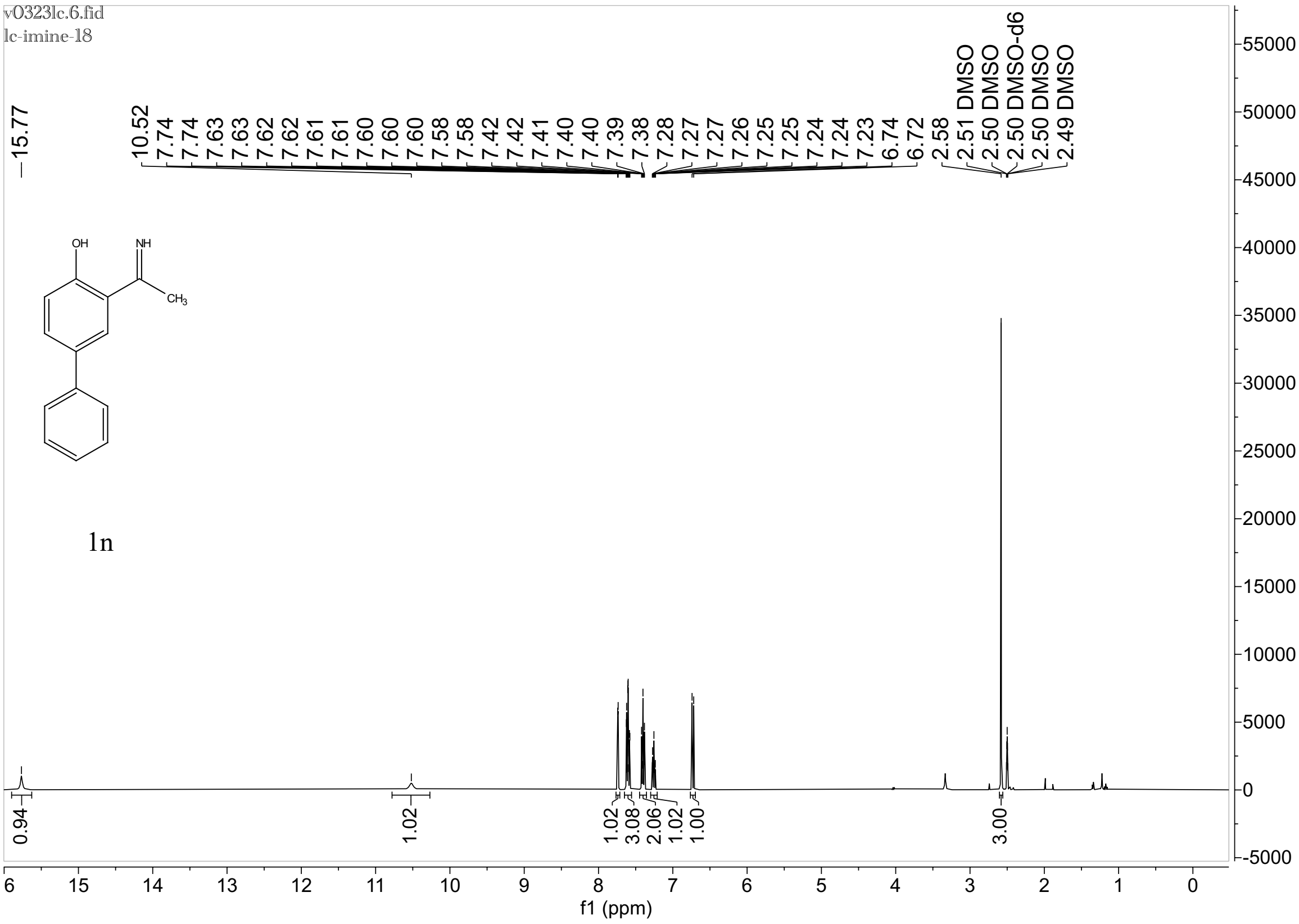
v0323lc.6.fid
lc-imine-18
—15.77
10.52
7.74
7.74
7.63
7.63
7.62
7.62
7.61
7.61
7.60
7.60
7.60
7.58
7.58
7.42
7.42
7.41
7.40
7.40
7.39
7.38
7.28
7.27
7.27
7.26
7.25
7.25
7.24
7.24
7.23
6.74
6.72
2.58
2.51 DMSO
2.50 DMSO
2.50 DMSO-d6
2.50 DMSO
2.49 DMSO
OH
NH
CH3
0.94
1.02
1.02
3.08
2.06
1.02
1.00
3.00
f1 (ppm)

1n

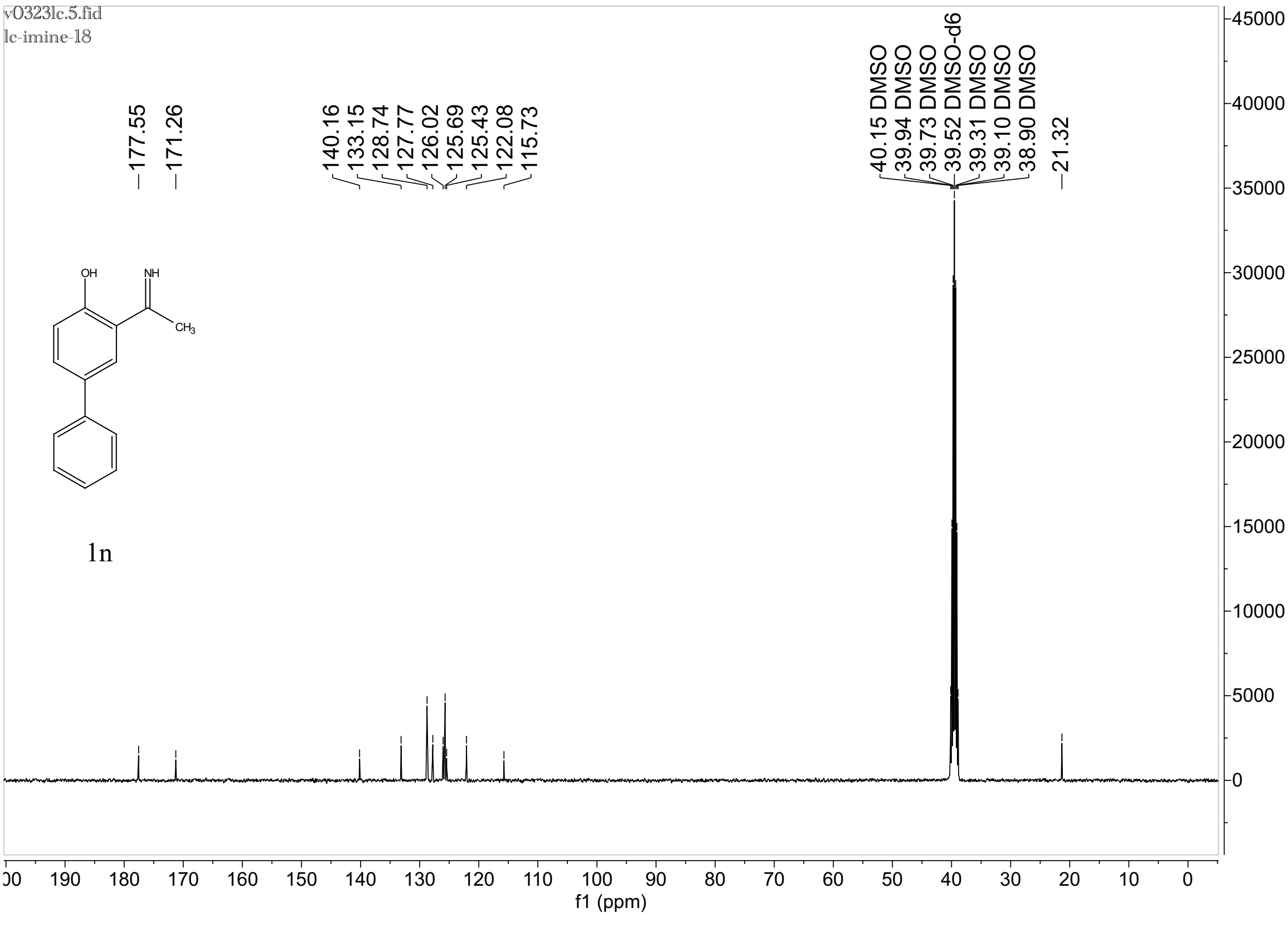
v0323lc.5.fid
lc-imine-18
—177.55
—171.26
140.16
133.15
128.74
127.77
126.02
125.69
125.43
122.08
115.73
40.15 DMSO
39.94 DMSO
39.73 DMSO
39.52 DMSO-d6
39.31 DMSO
39.10 DMSO
38.90 DMSO
—21.32
OH
NH
CH3
f1 (ppm)

1n

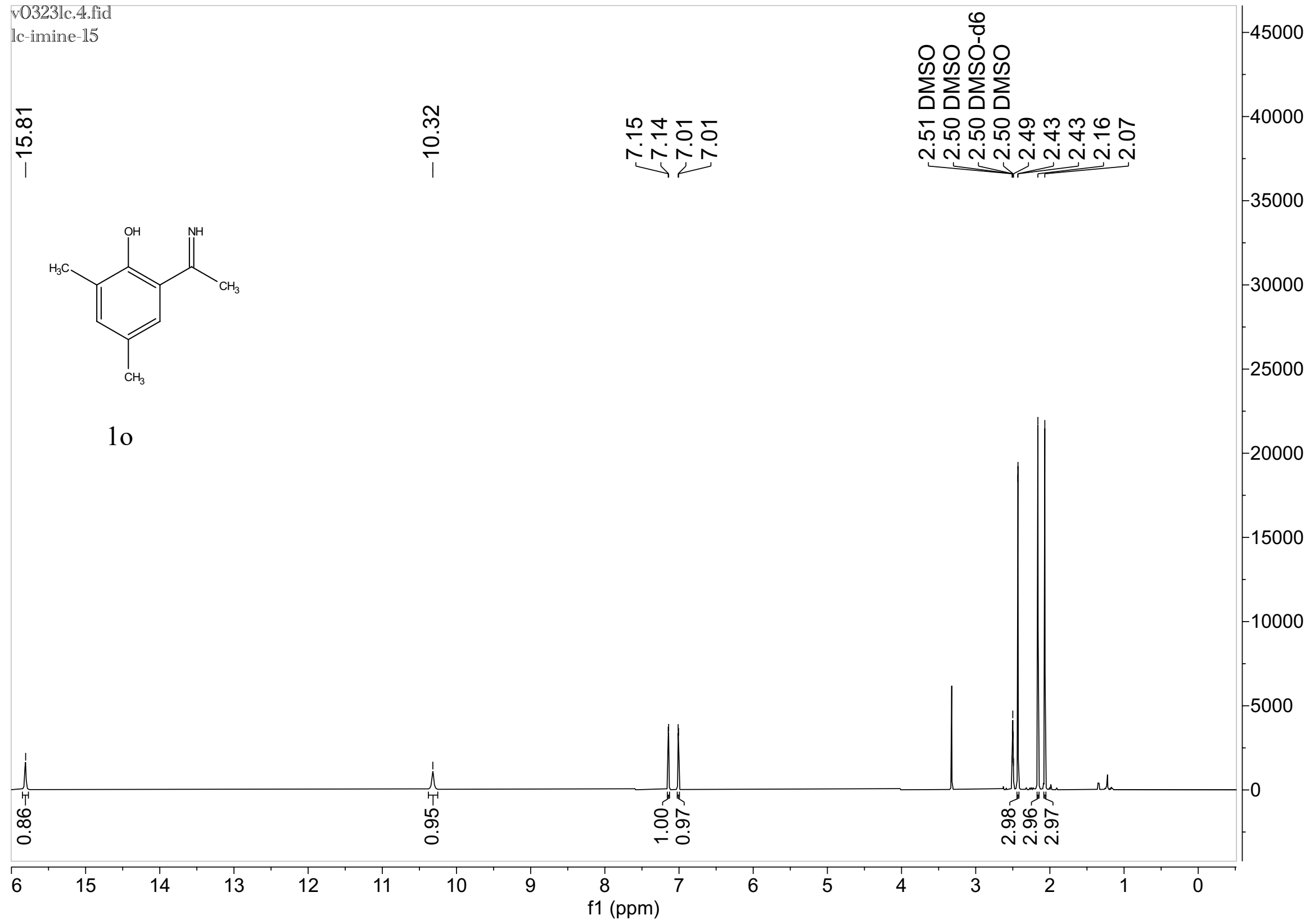
v0323lc.4.fid
lc-imine-15
15.81
10.32
7.15
7.14
7.01
7.01
2.51 DMSO
2.50 DMSO
2.50 DMSO-d6
2.50 DMSO
2.49
2.43
2.43
2.16
2.07
1o
0.86
0.95
1.00
0.97
2.98
2.96
2.97
f1 (ppm)

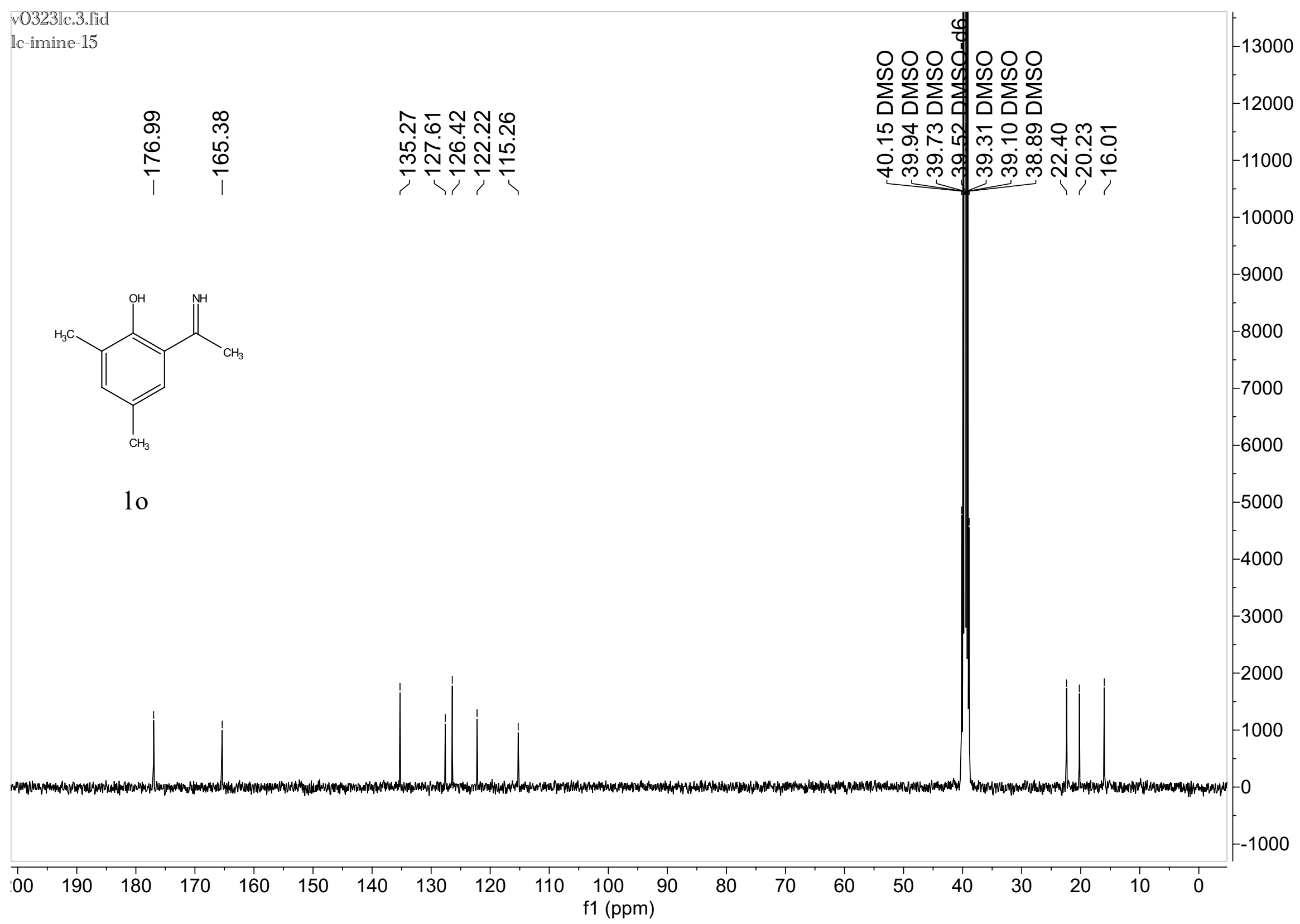
v0323lc.3.fid
lc-imine-15
176.99
165.38
135.27
127.61
126.42
122.22
115.26
40.15 DMSO
39.94 DMSO
39.73 DMSO
39.52 DMSO-d6
39.31 DMSO
39.10 DMSO
38.89 DMSO
22.40
20.23
16.01
1o
f1 (ppm)

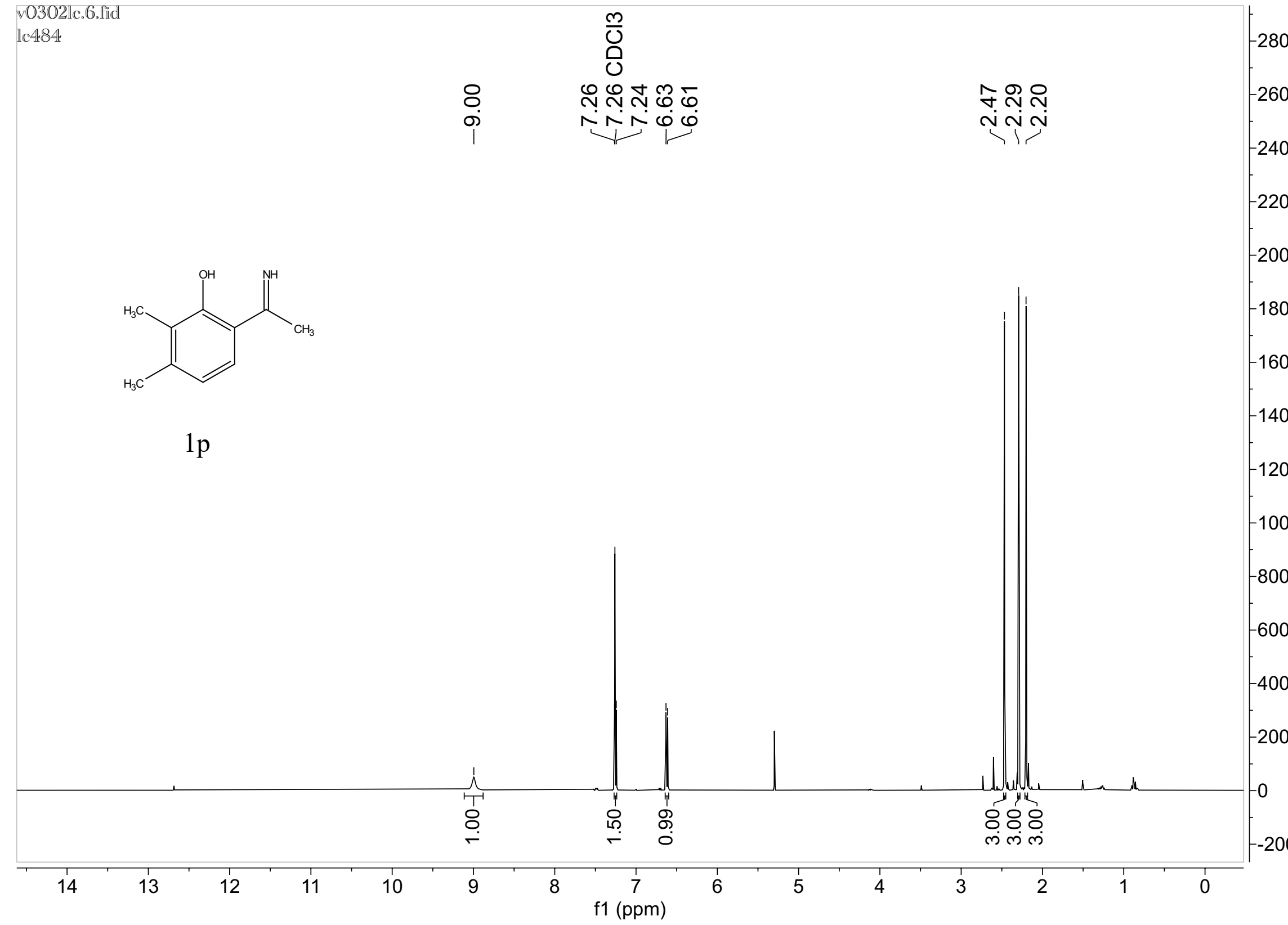
v0302lc.6.fid
lc484
9.00
7.26
7.26 CDCl3
7.24
6.63
6.61
2.47
2.29
2.20
1.00
1.50
0.99
3.00
3.00
3.00
f1 (ppm)

1p

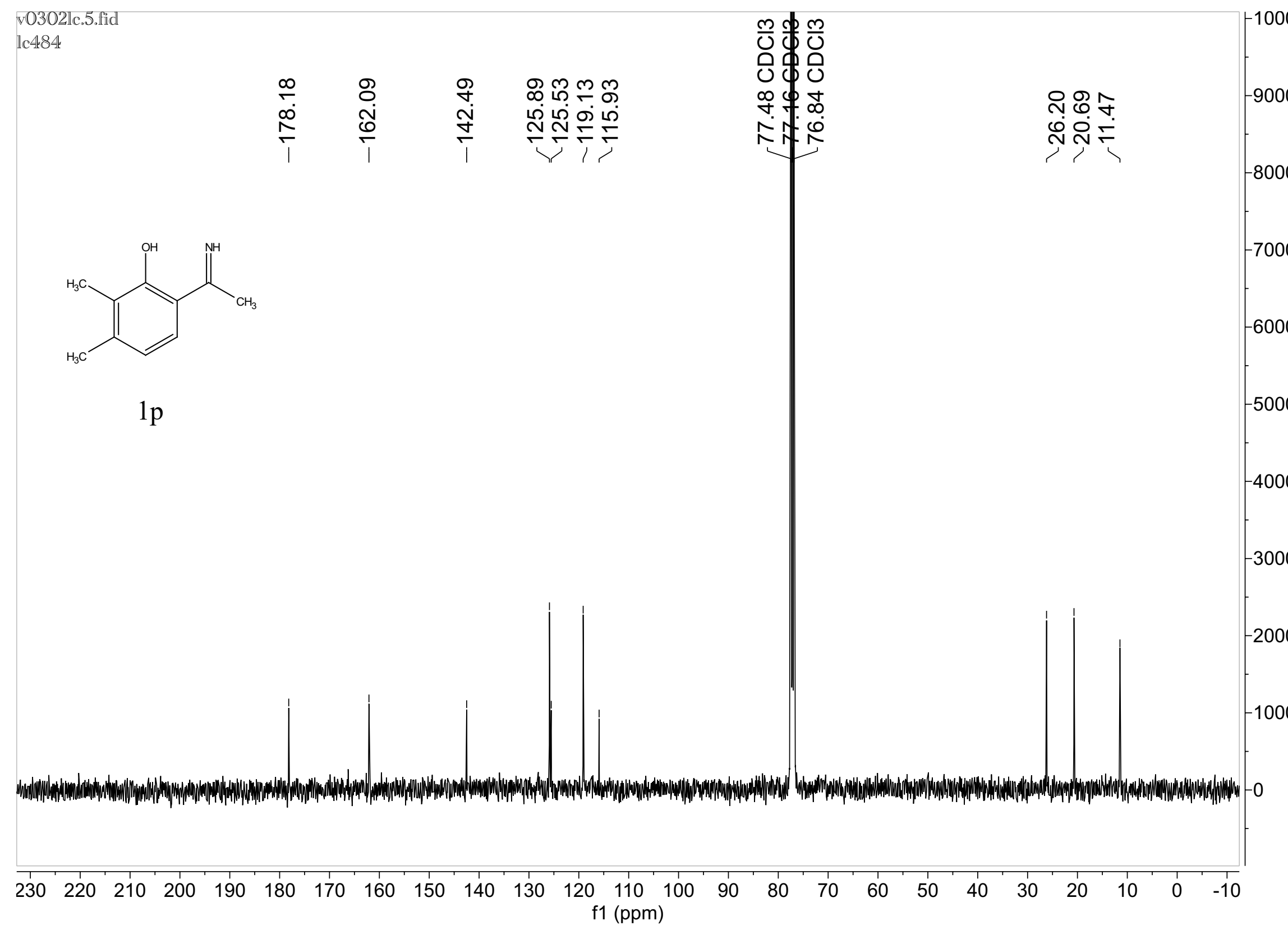
v0302lc.5.fid
lc484
178.18
162.09
142.49
125.89
125.53
119.13
115.93
77.48 CDCl3
77.16 CDCl3
76.84 CDCl3
26.20
20.69
11.47
f1 (ppm)

1p

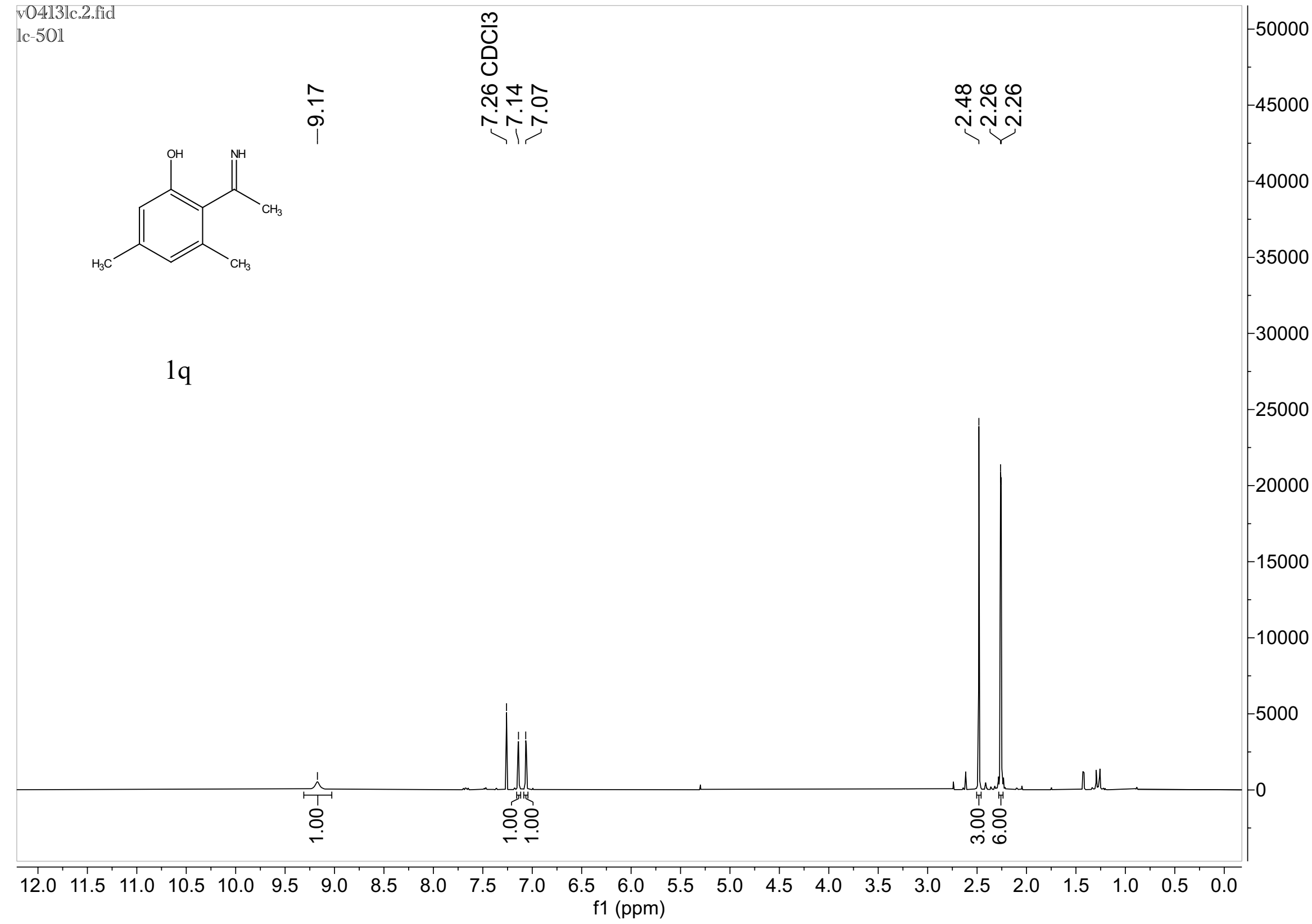
v0413lc.2.fid
lc-501
9.17
7.26 CDCl3
7.14
7.07
2.48
2.26
2.26
OH
NH
CH3
H3C
CH3
1q
1.00
1.00
1.00
3.00
6.00
f1 (ppm)

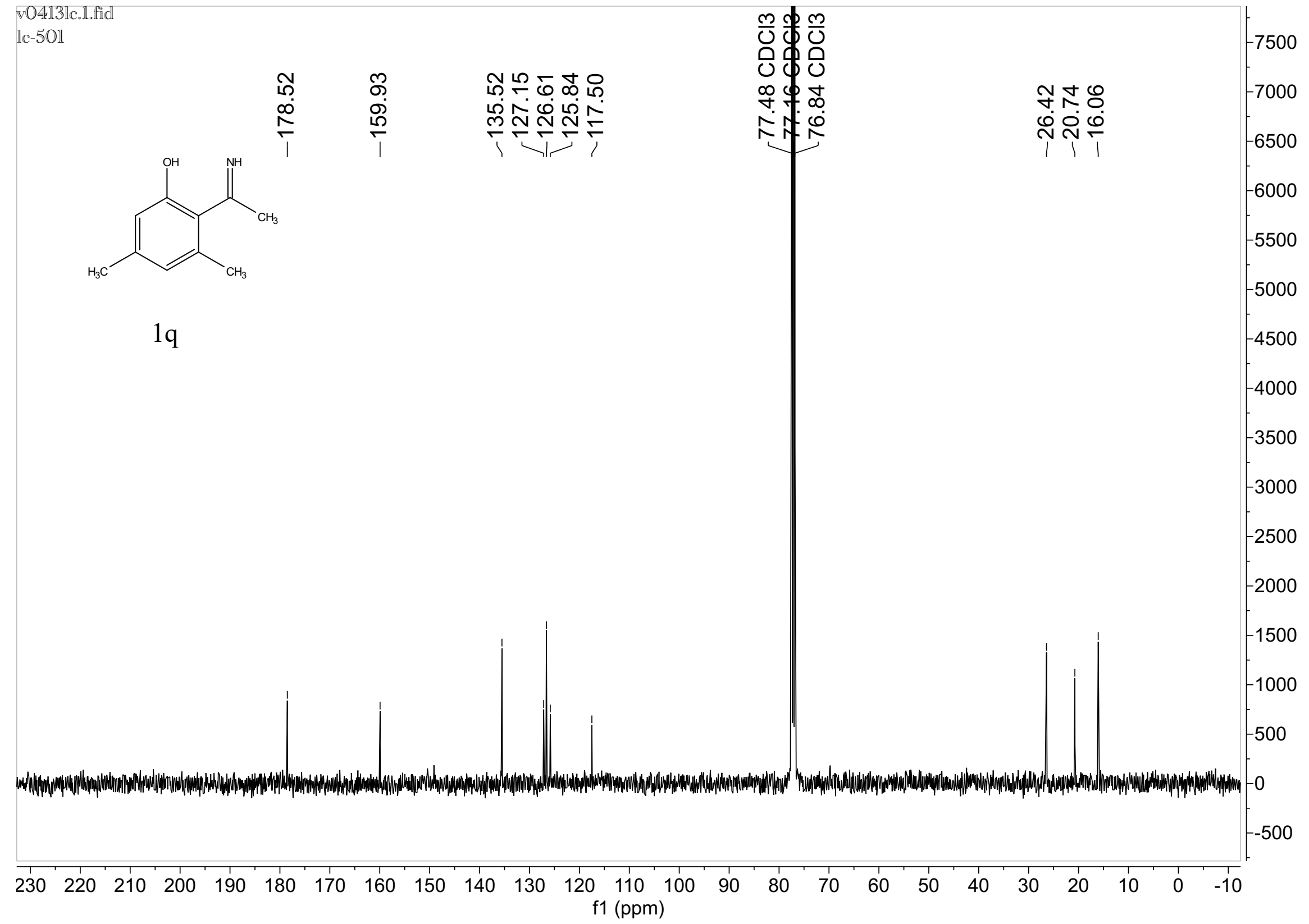
v0413lc.1.fid
lc-501
178.52
159.93
135.52
127.15
126.61
125.84
117.50
77.48 CDCl3
77.16 CDCl3
76.84 CDCl3
26.42
20.74
16.06
OH
NH
CH3
H3C
CH3
1q
f1 (ppm)

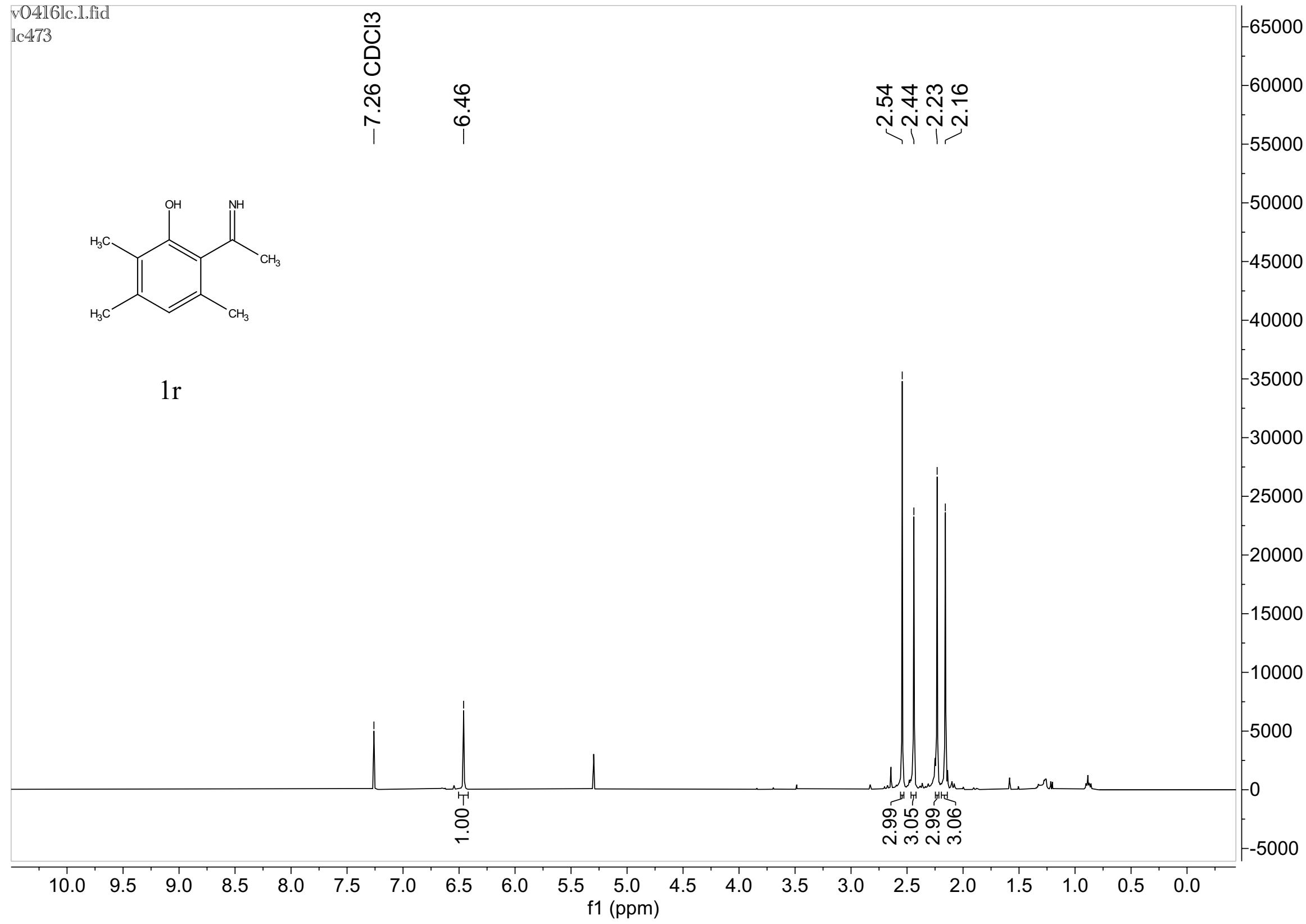
v0416lc.1.fid
lc473
7.26 CDCl3
6.46
2.54
2.44
2.23
2.16
OH
NH
H3C
CH3
H3C
CH3
1r
1.00
2.99
3.05
2.99
3.06
f1 (ppm)

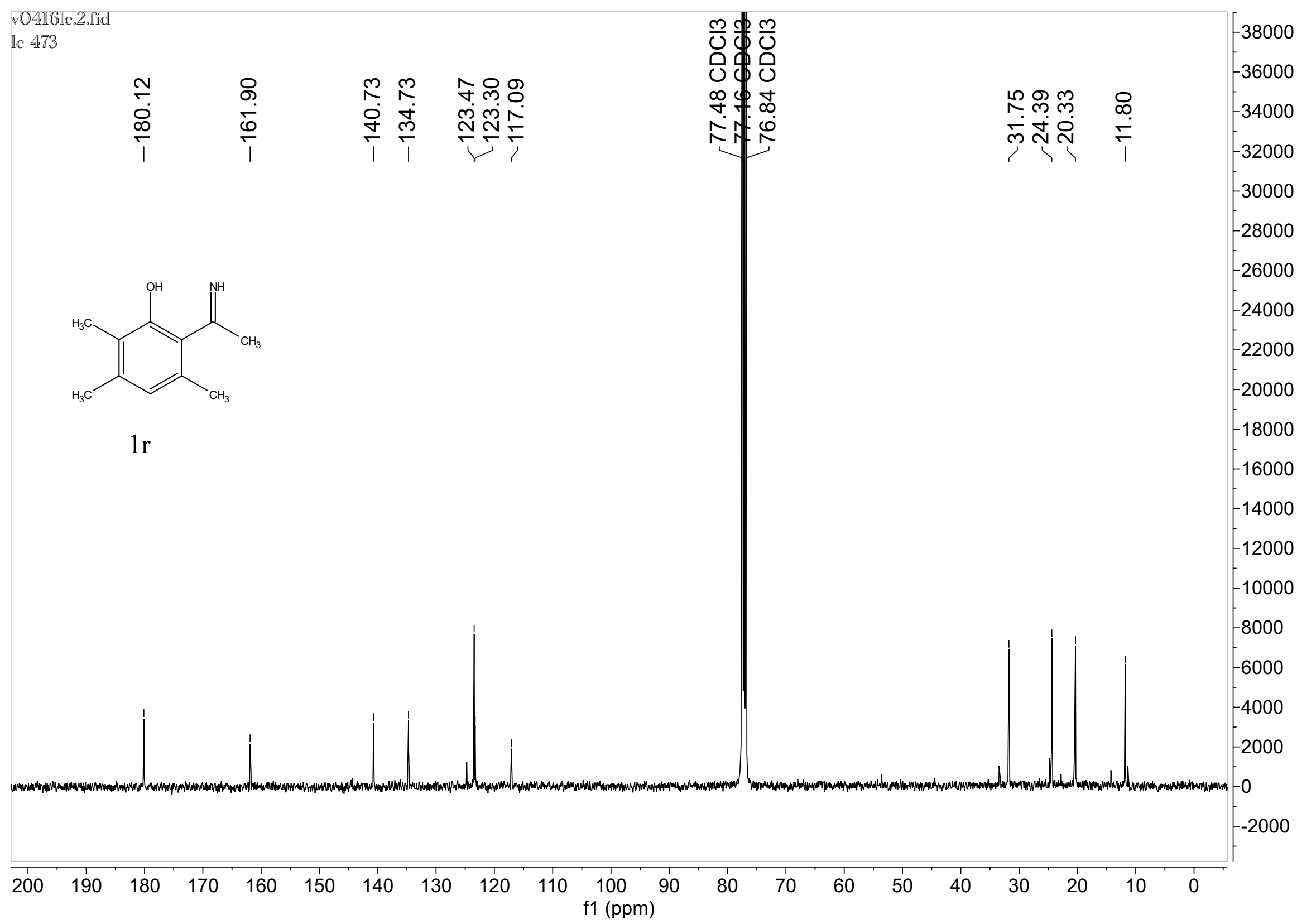
v0416lc.2.fid
lc-473
180.12
161.90
140.73
134.73
123.47
123.30
117.09
77.48 CDCl3
77.16 CDCl3
76.84 CDCl3
31.75
24.39
20.33
11.80
OH
NH
H3C
CH3
H3C
CH3
1r
f1 (ppm)

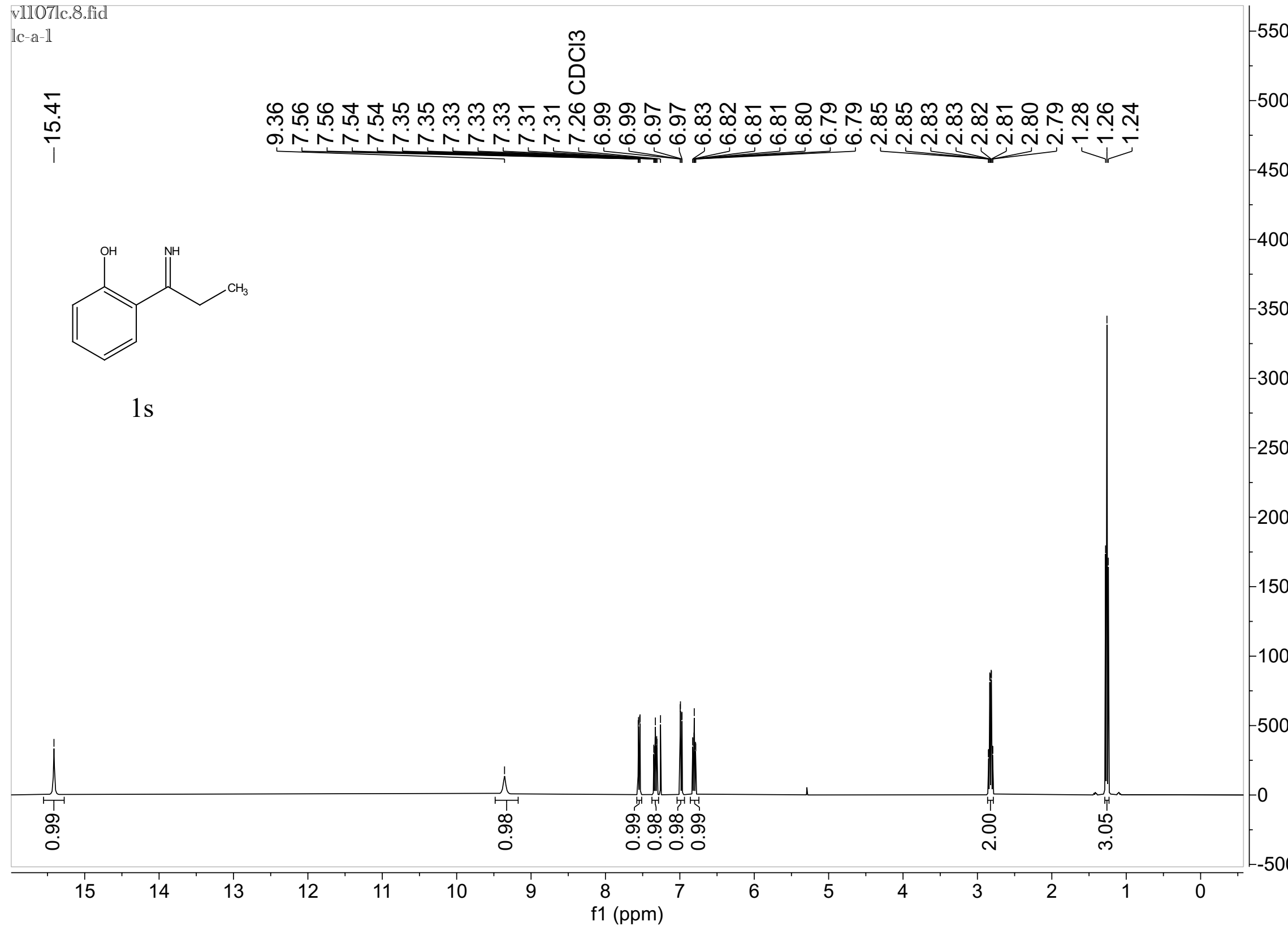
v1107lc.8.fid
lc-a-1
15.41
9.36
7.56
7.56
7.54
7.54
7.35
7.35
7.33
7.33
7.33
7.31
7.26 CDCl3
6.99
6.99
6.97
6.97
6.83
6.82
6.81
6.81
6.80
6.79
6.79
2.85
2.85
2.83
2.83
2.82
2.81
2.80
2.79
1.28
1.26
1.24
OH
NH
CH3
1s
0.99
0.98
0.99
0.98
0.98
0.99
2.00
3.05
f1 (ppm)

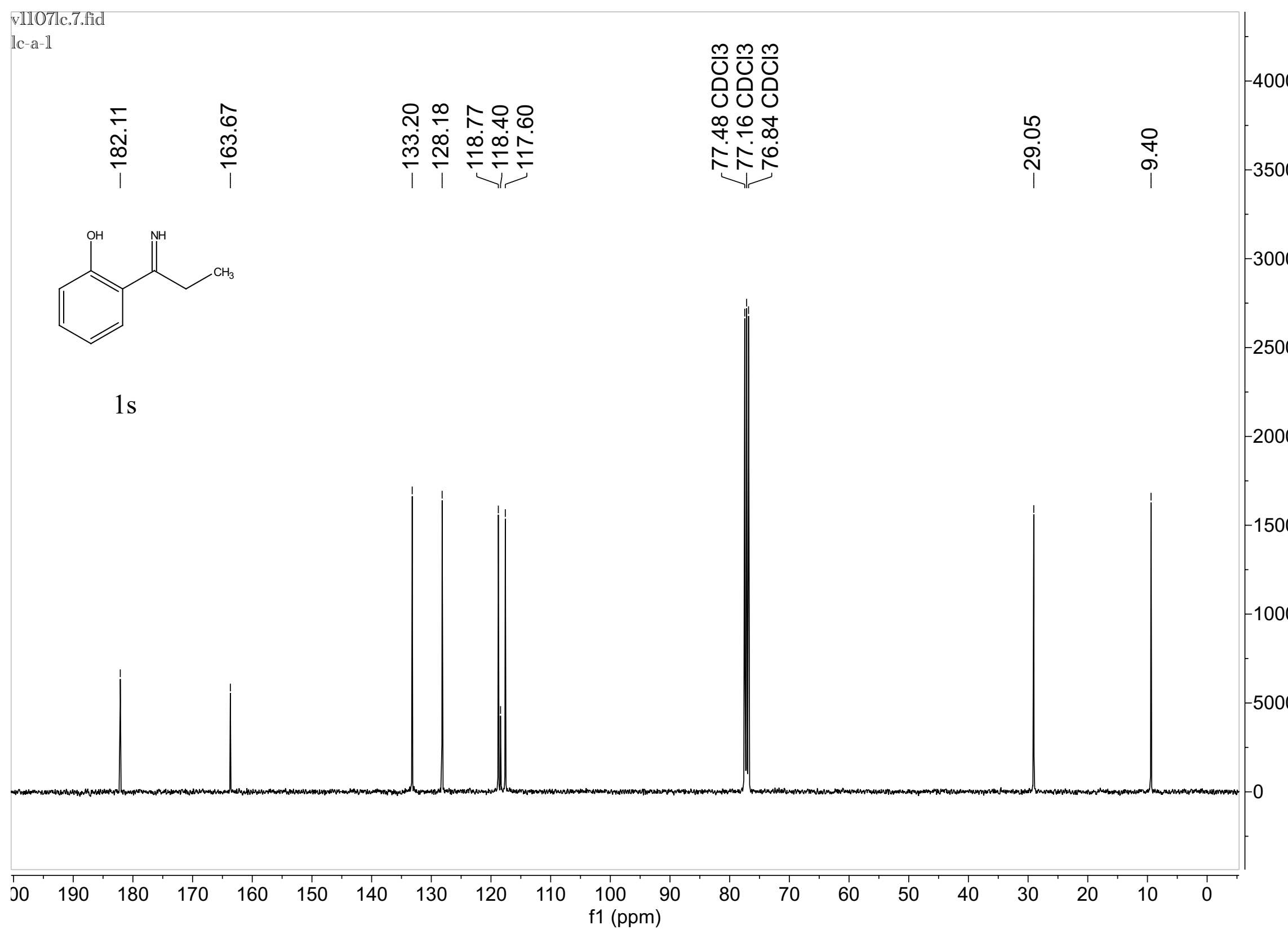
v1107lc.7.fid
lc-a-1
182.11
163.67
133.20
128.18
118.77
118.40
117.60
77.48 CDCl3
77.16 CDCl3
76.84 CDCl3
29.05
9.40
OH
NH
CH3
1s
f1 (ppm)

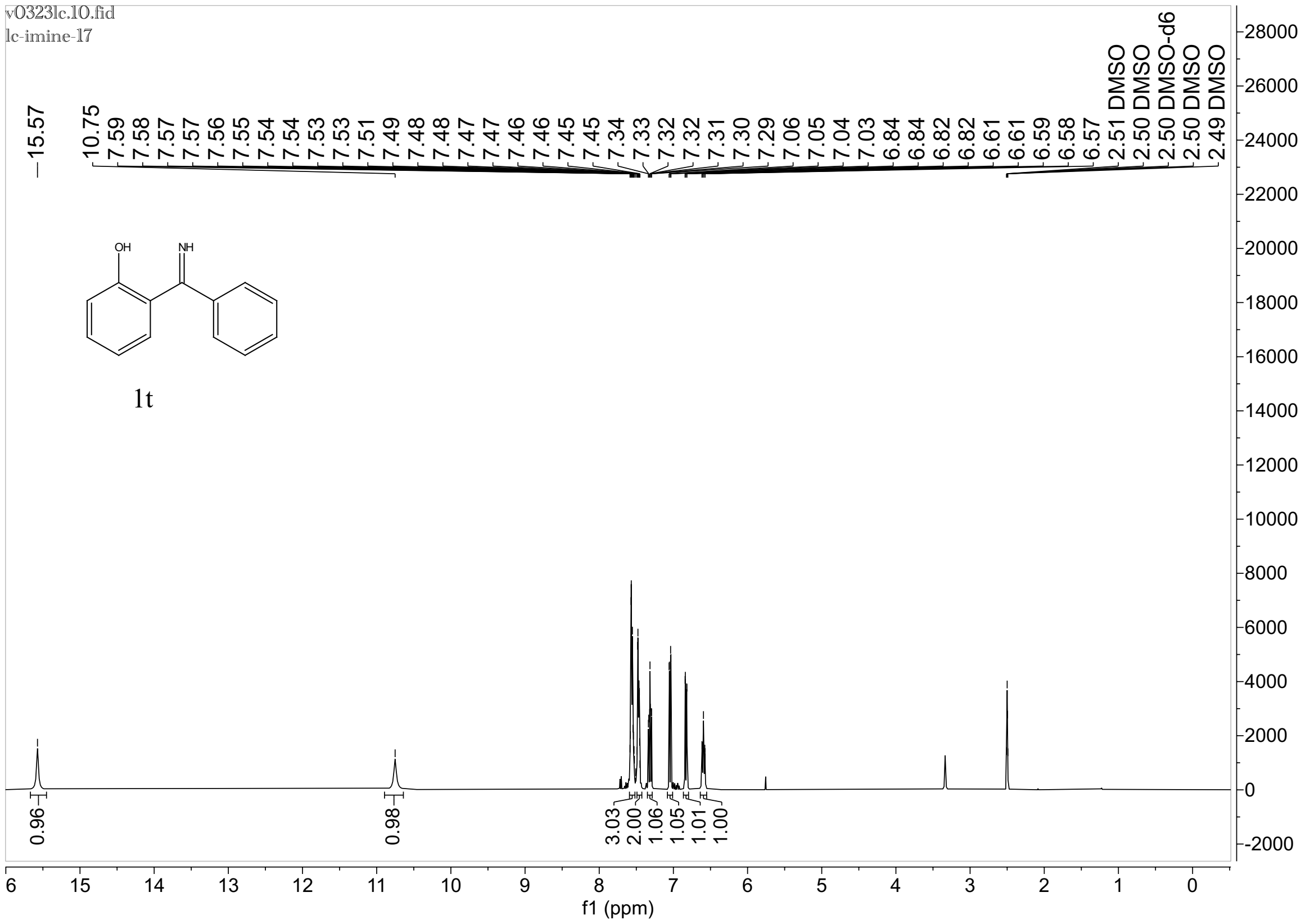
v0323lc.10.fid
lc-imine-17
15.57
10.75
2.51 DMSO
2.50 DMSO
2.50 DMSO-d6
2.50 DMSO
2.49 DMSO
OH
NH
1t
0.96
0.98
3.03
2.00
1.06
1.05
1.01
1.00
f1 (ppm)

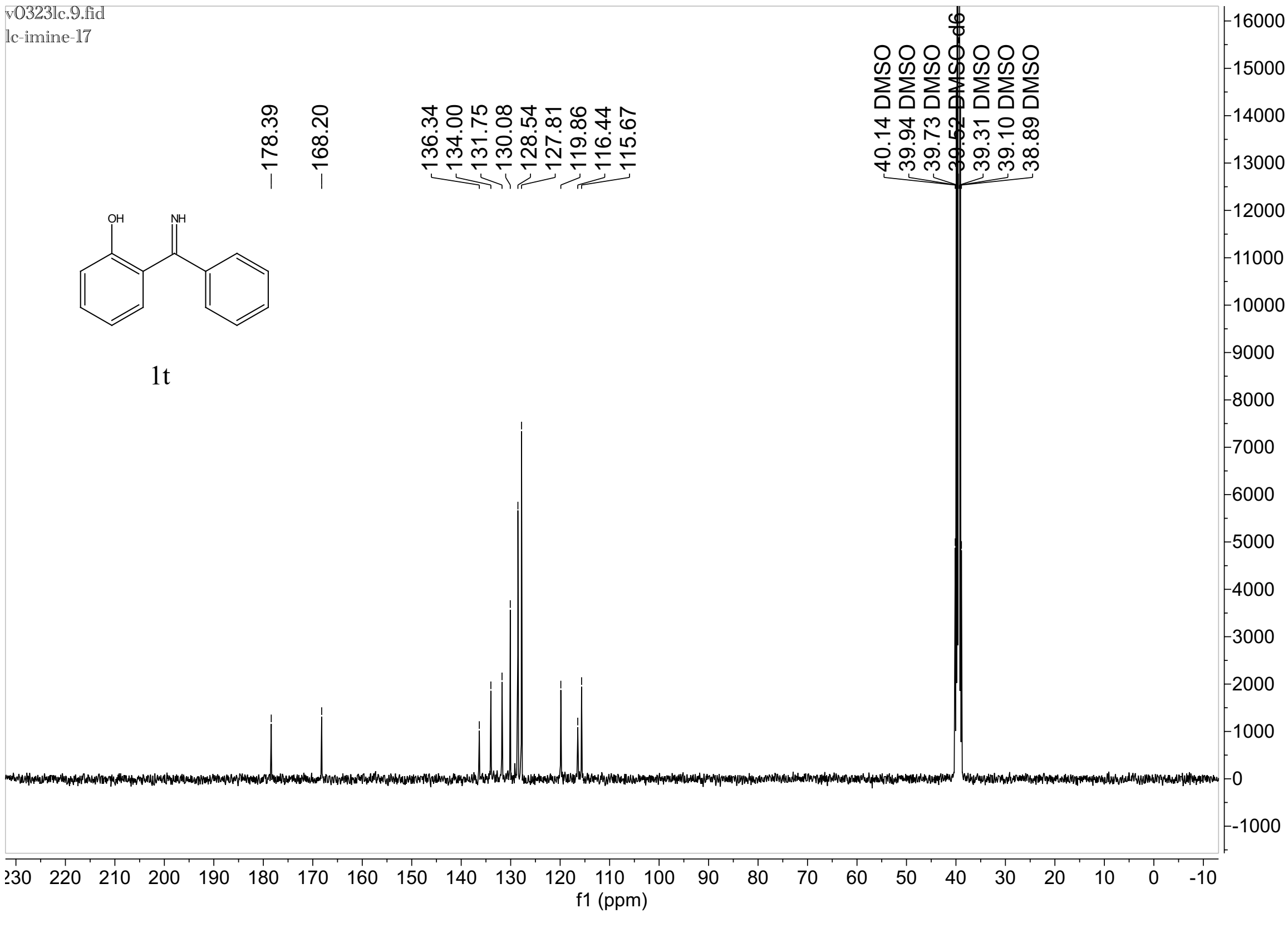
v0323lc.9.fid
lc-imine-17
178.39
168.20
136.34
134.00
131.75
130.08
128.54
127.81
119.86
116.44
115.67
40.14 DMSO
39.94 DMSO
39.73 DMSO
39.52 DMSO-d6
39.31 DMSO
39.10 DMSO
38.89 DMSO
OH
NH
1t
f1 (ppm)

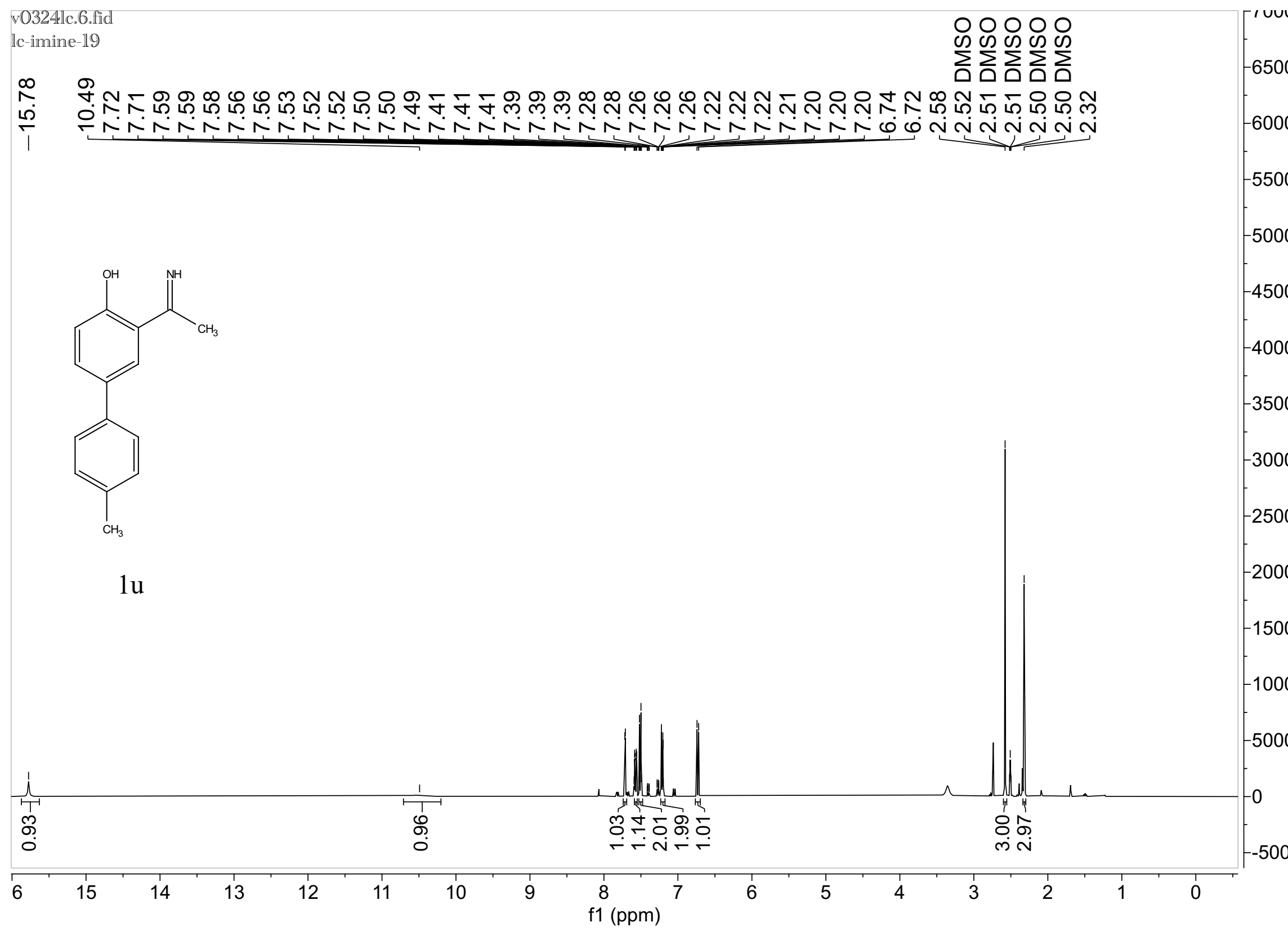
v0324lc.6.fid
lc-imine-19
1u
f1 (ppm)

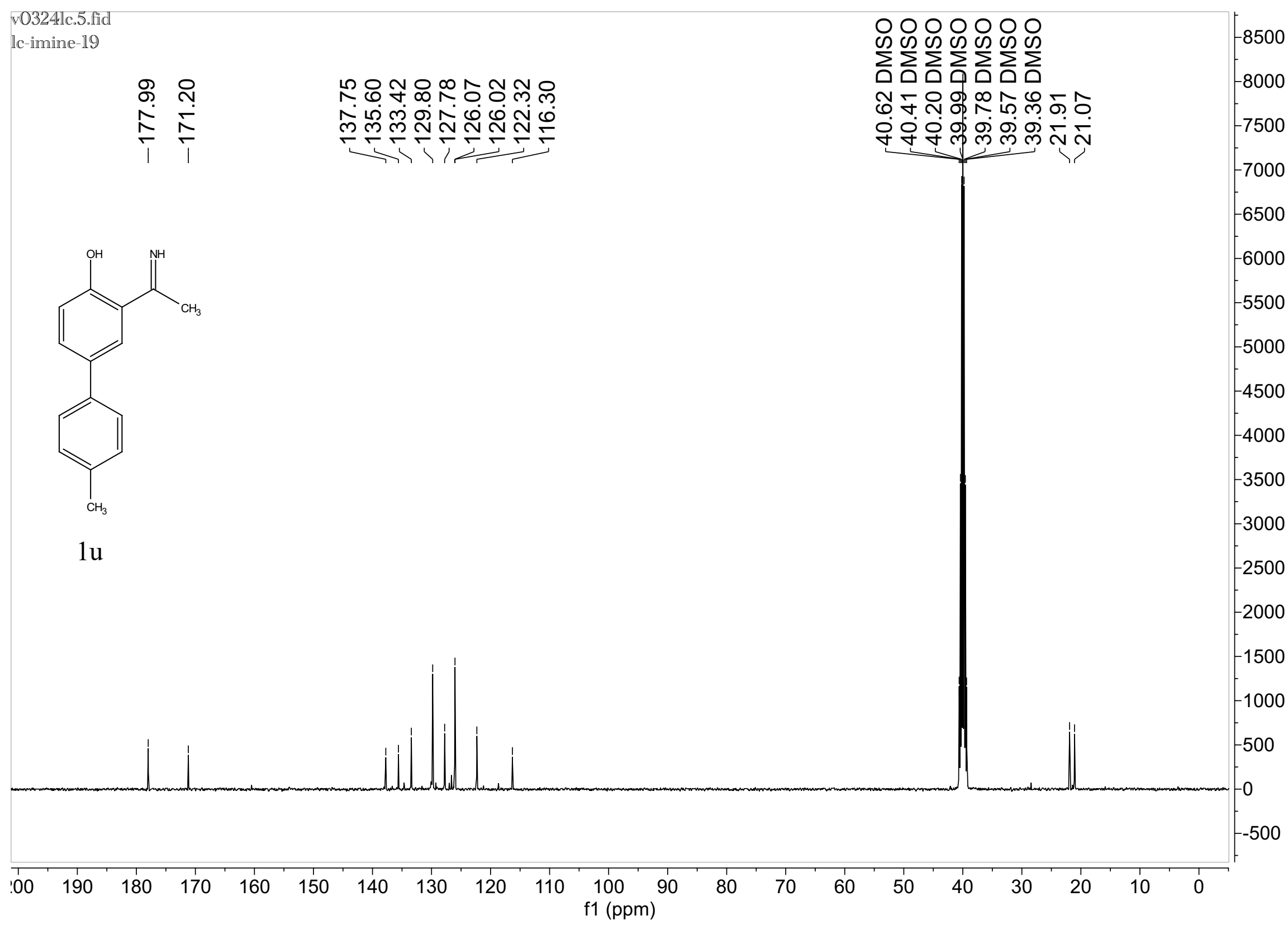
v0324lc.5.fid
lc-imine-19
1u
f1 (ppm)

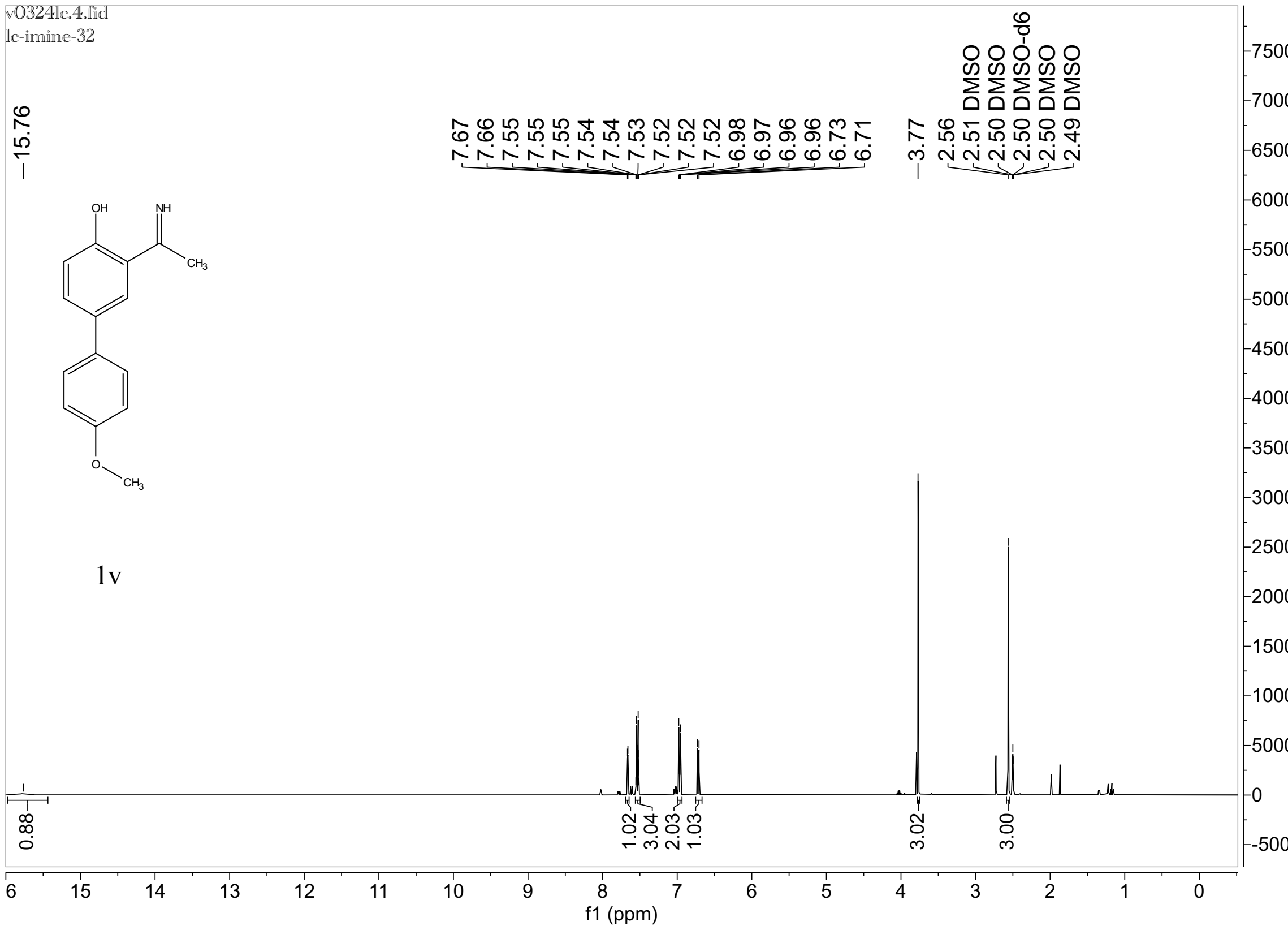

1v

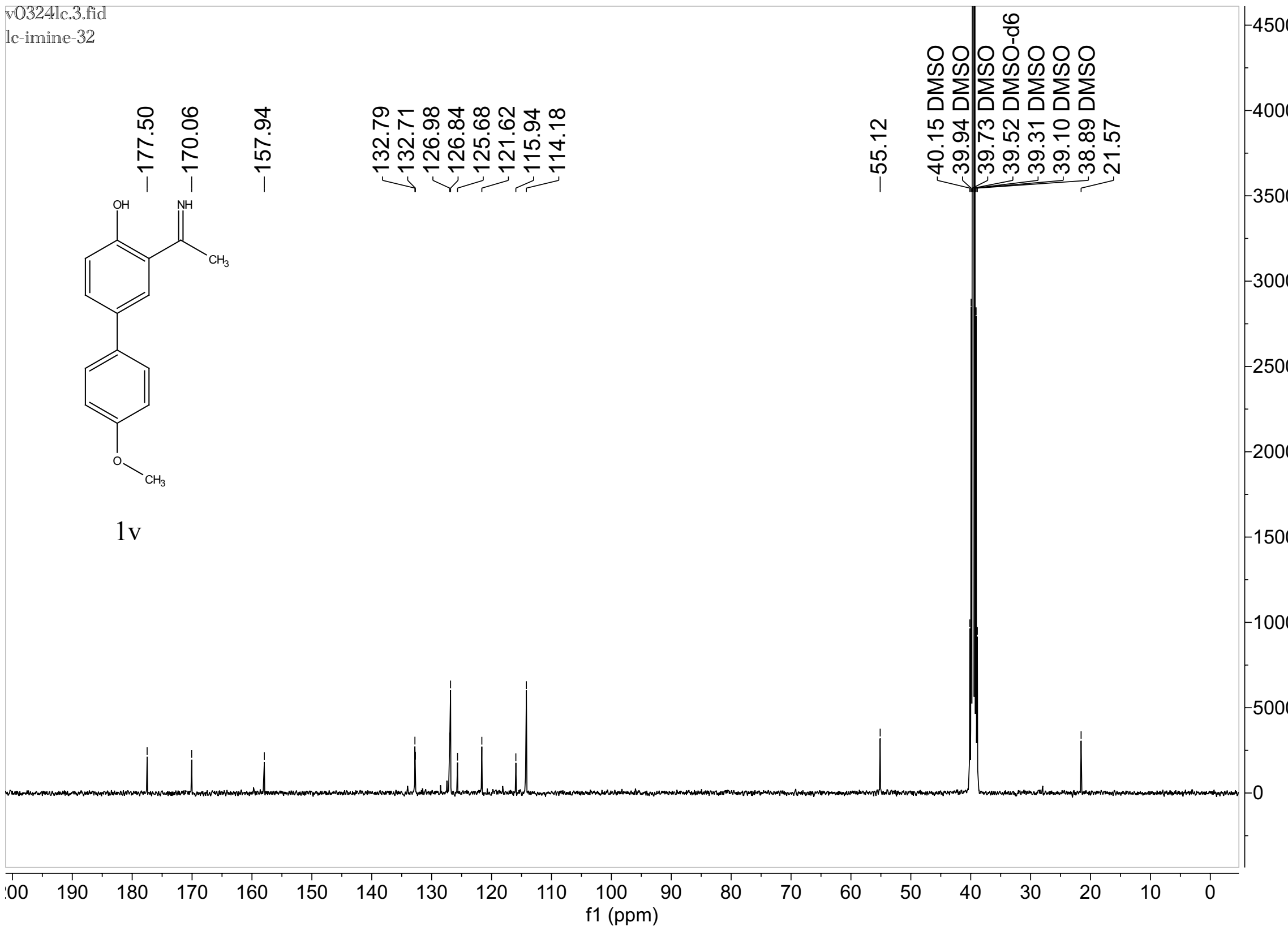

1v

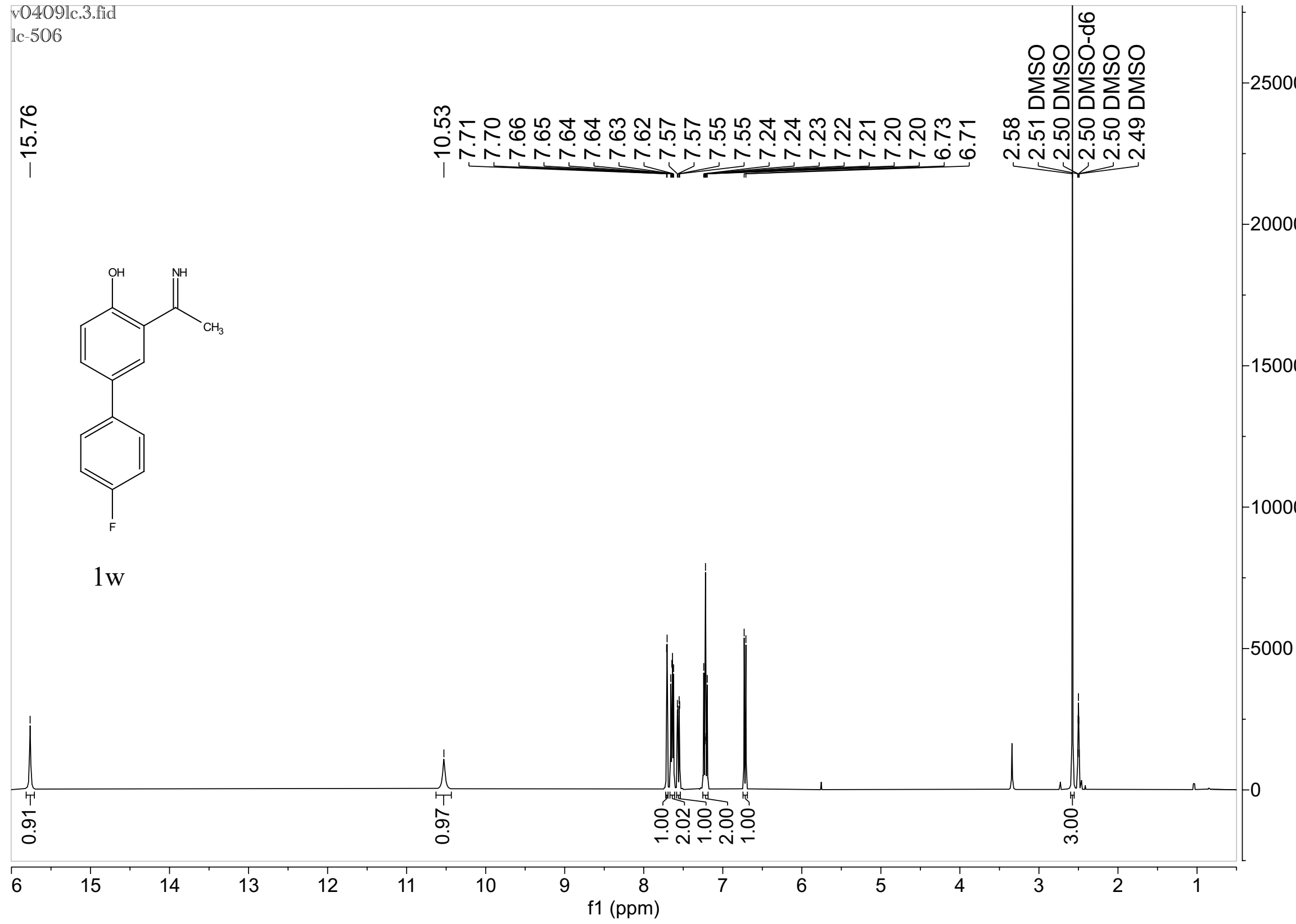
v0409lc.3.fid
lc-506
1w
f1 (ppm)

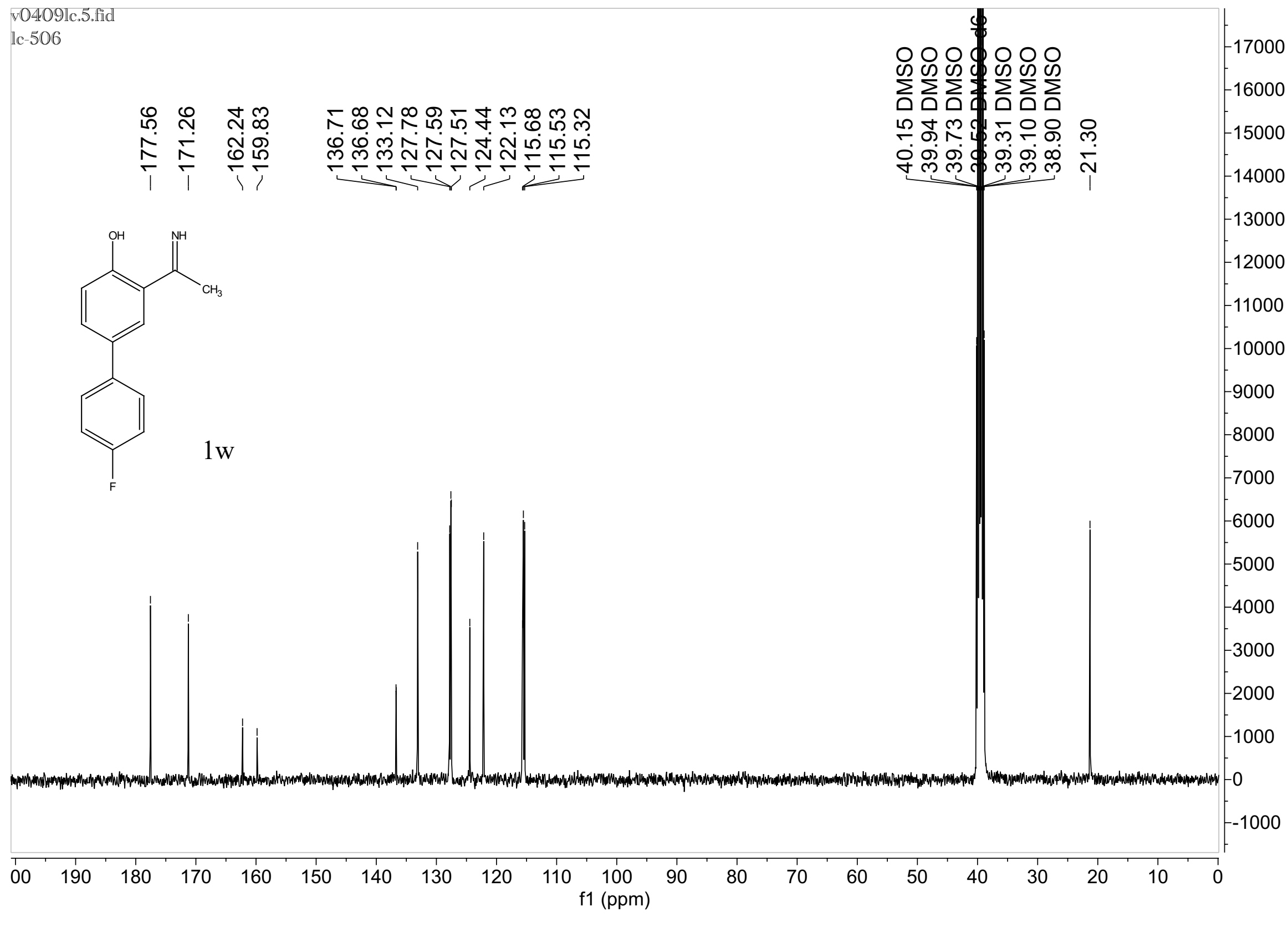
v0409lc.5.fid
lc-506
1w
f1 (ppm)

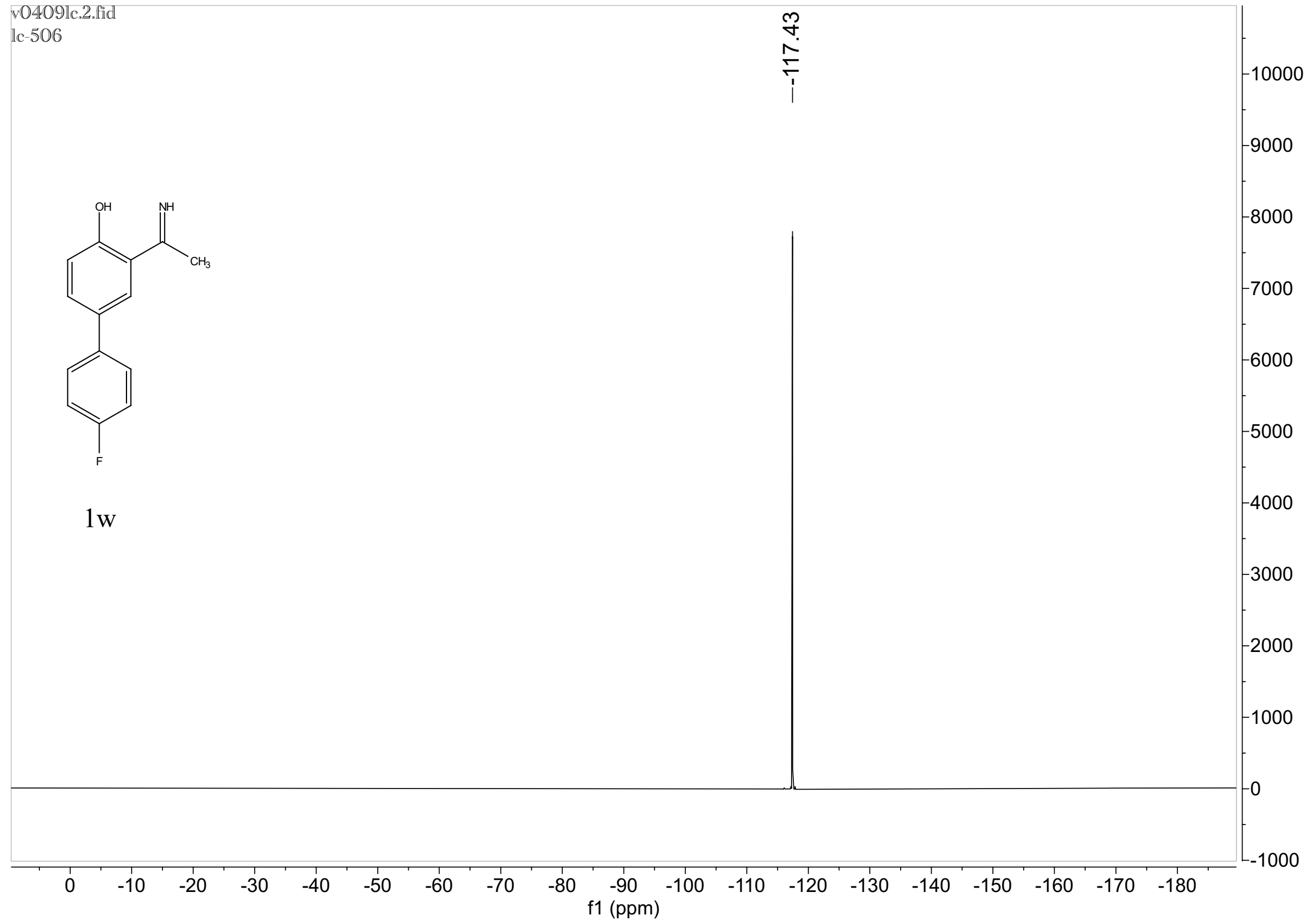
v0409lc.2.fid
lc-506
-117.43
OH
NH
CH3
F
10000
9000
8000
7000
6000
5000
4000
3000
2000
1000
0
-1000
0
-10
-20
-30
-40
-50
-60
-70
-80
-90
-100
-110
-120
-130
-140
-150
-160
-170
-180
f1 (ppm)

1w

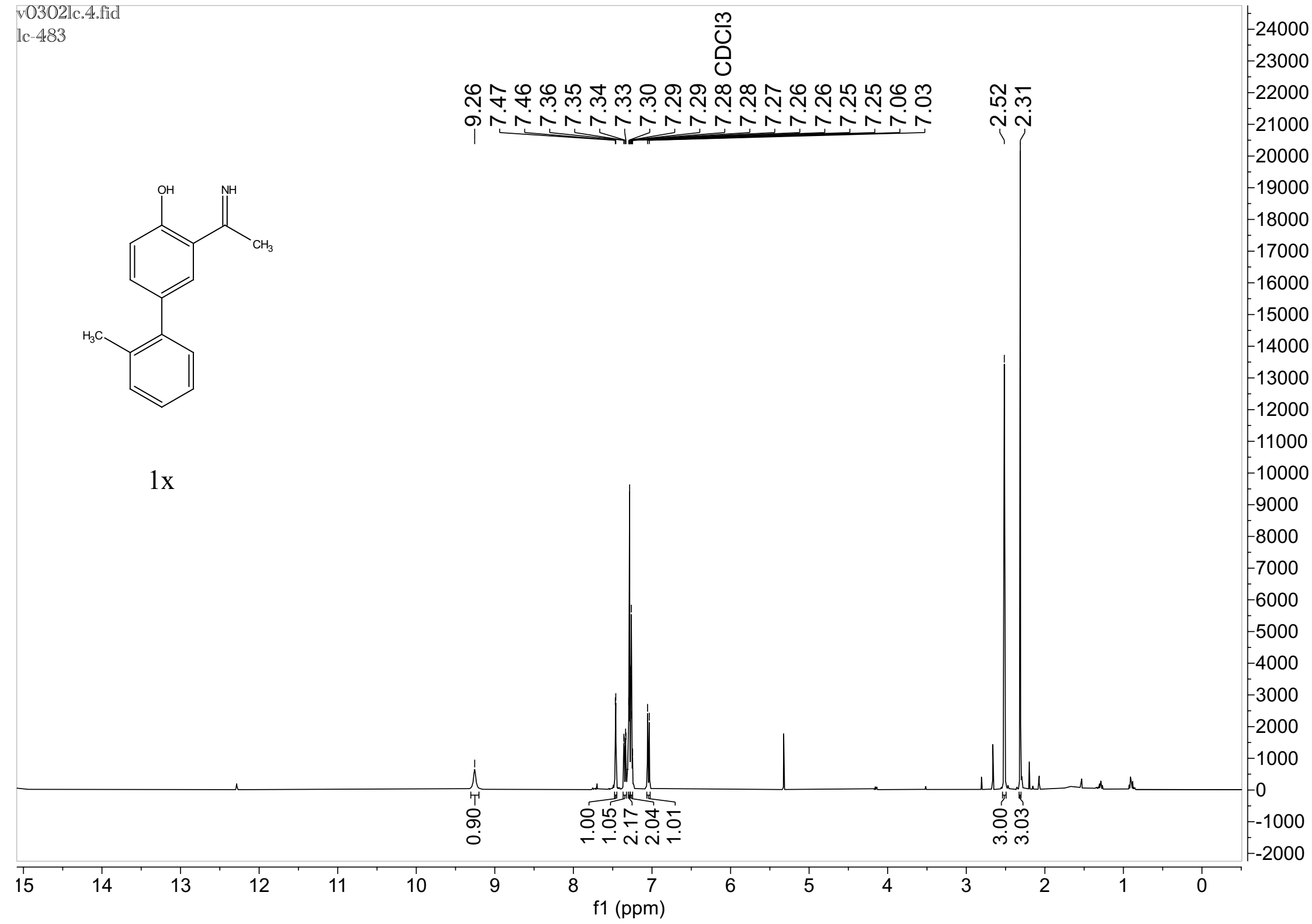

1x

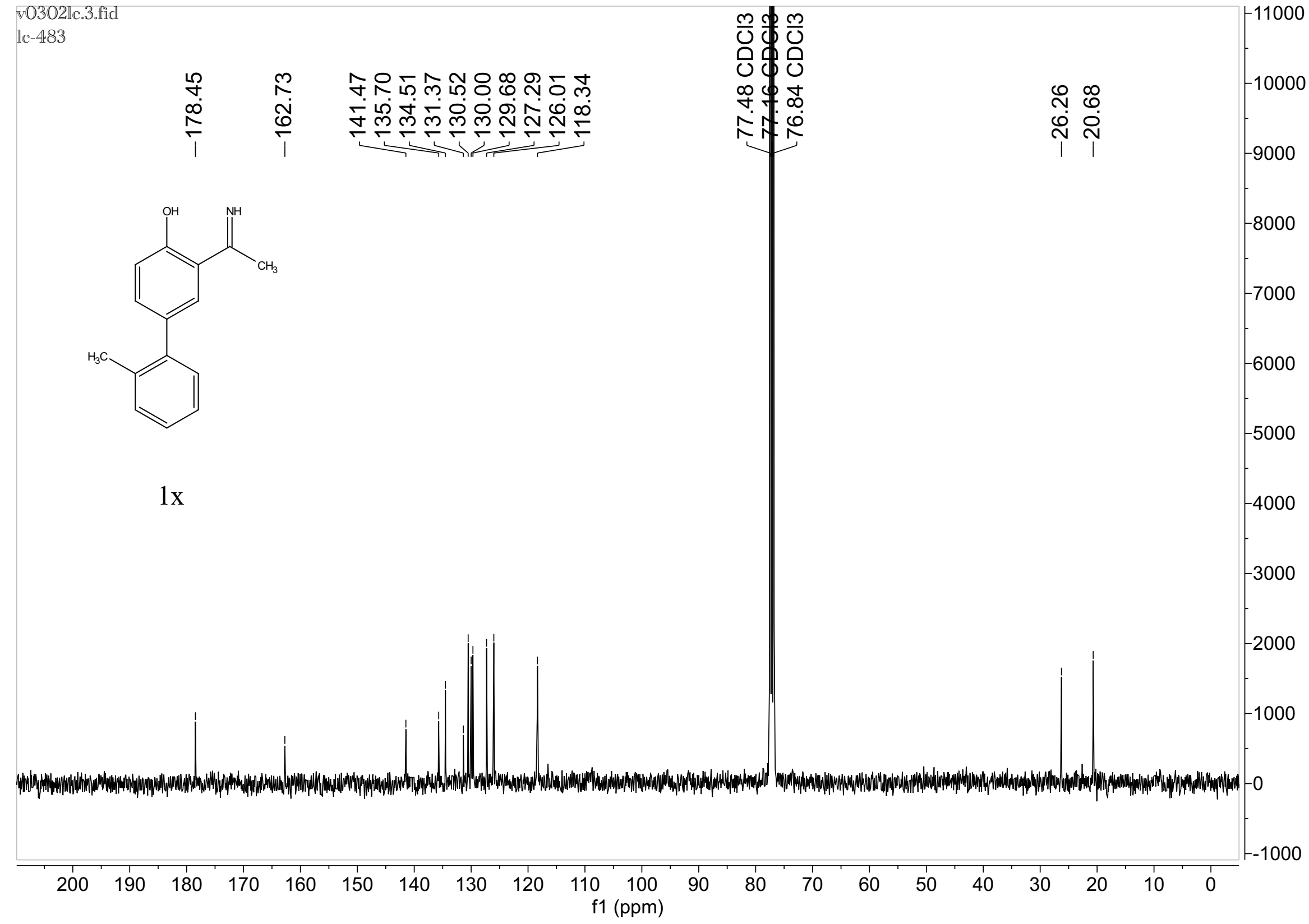

1x

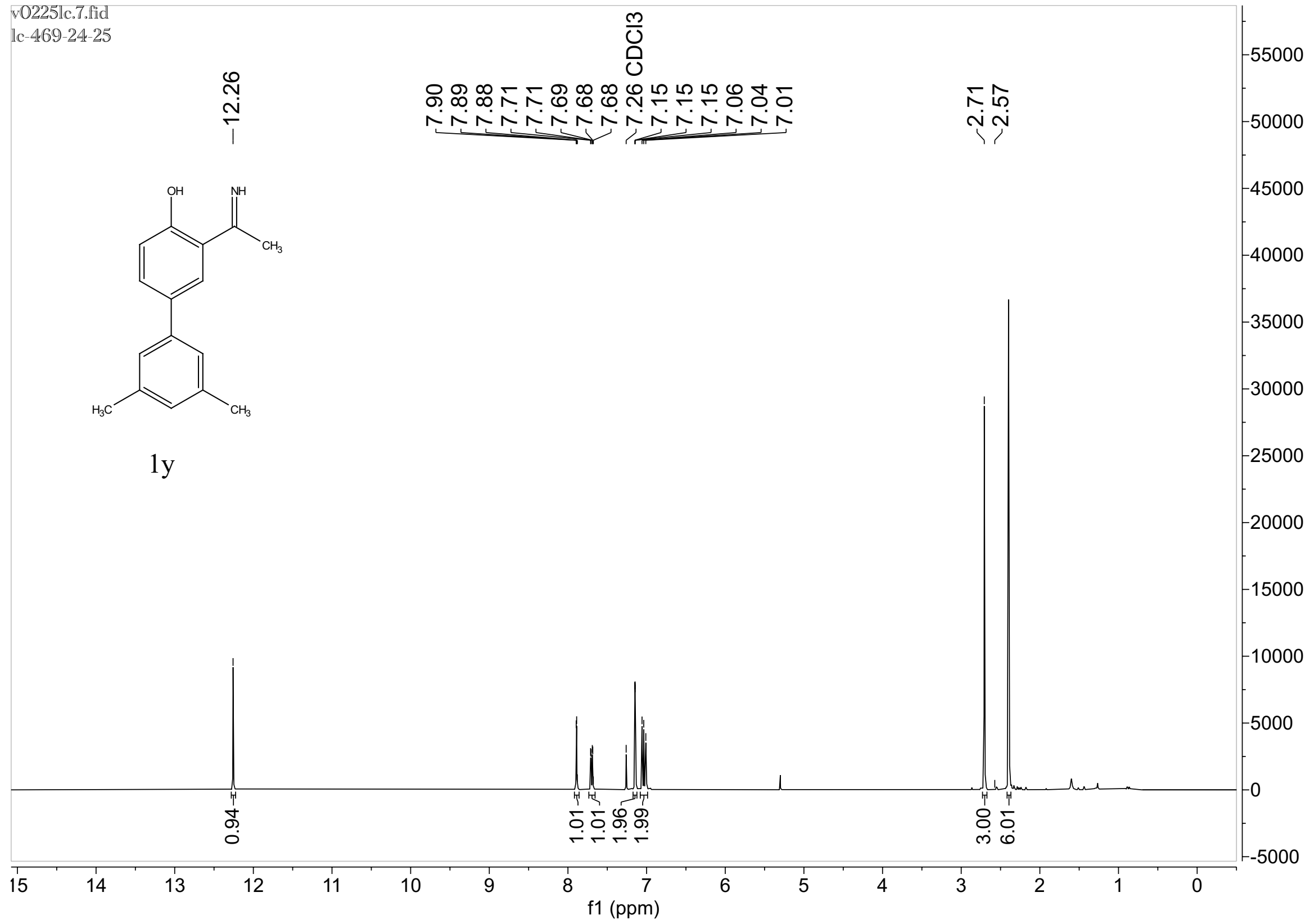
v0225lc.7.fid
lc-469-24-25
12.26
7.90
7.89
7.88
7.71
7.71
7.69
7.68
7.68
7.26 CDCl3
7.15
7.15
7.15
7.06
7.04
7.01
2.71
2.57
OH
NH
CH3
H3C
CH3
1y
0.94
1.01
1.01
1.96
1.99
3.00
6.01
f1 (ppm)

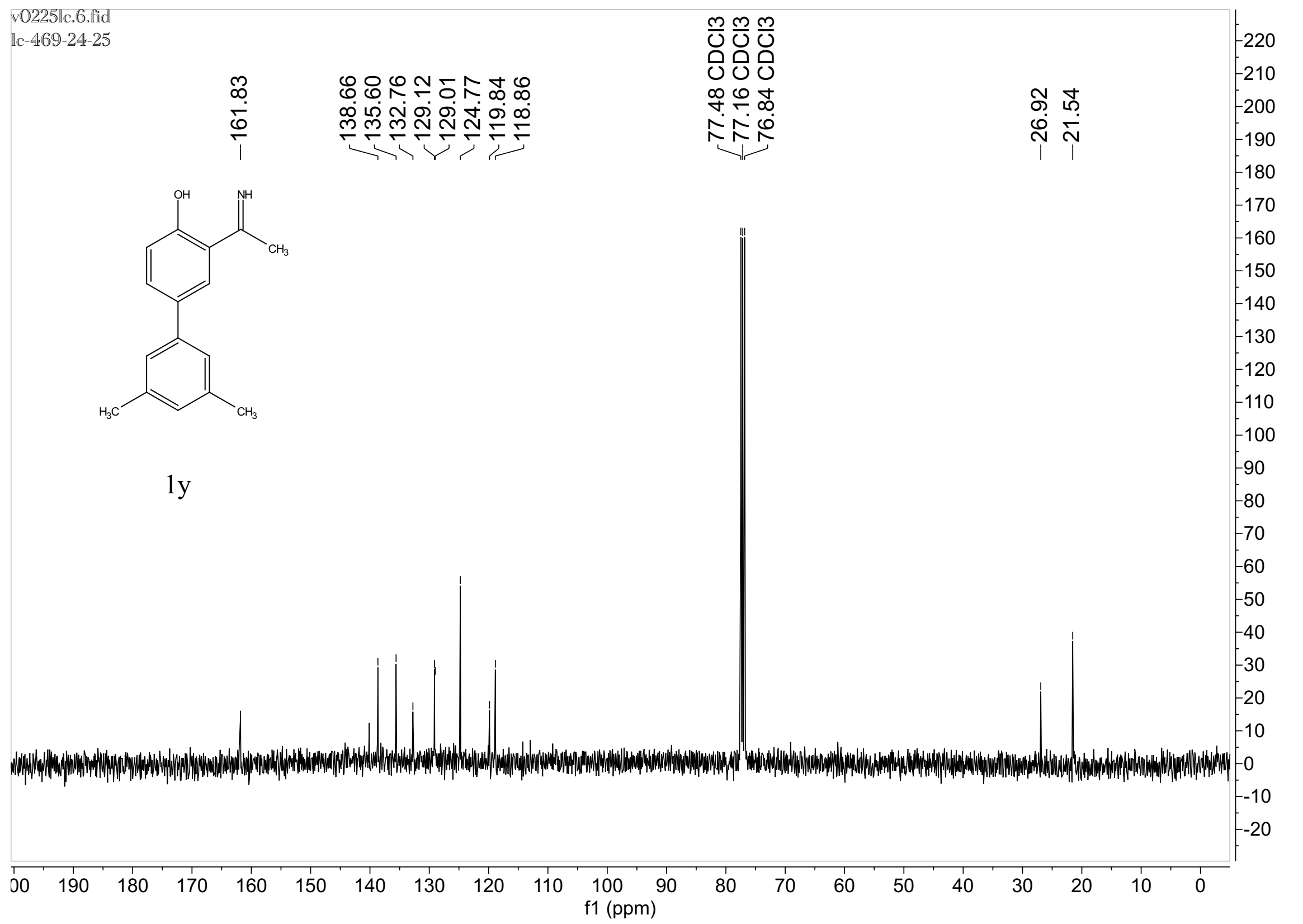
v0225lc.6.fid
lc-469-24-25
161.83
138.66
135.60
132.76
129.12
129.01
124.77
119.84
118.86
77.48 CDCl3
77.16 CDCl3
76.84 CDCl3
26.92
21.54
OH
NH
CH3
H3C
CH3
1y
f1 (ppm)

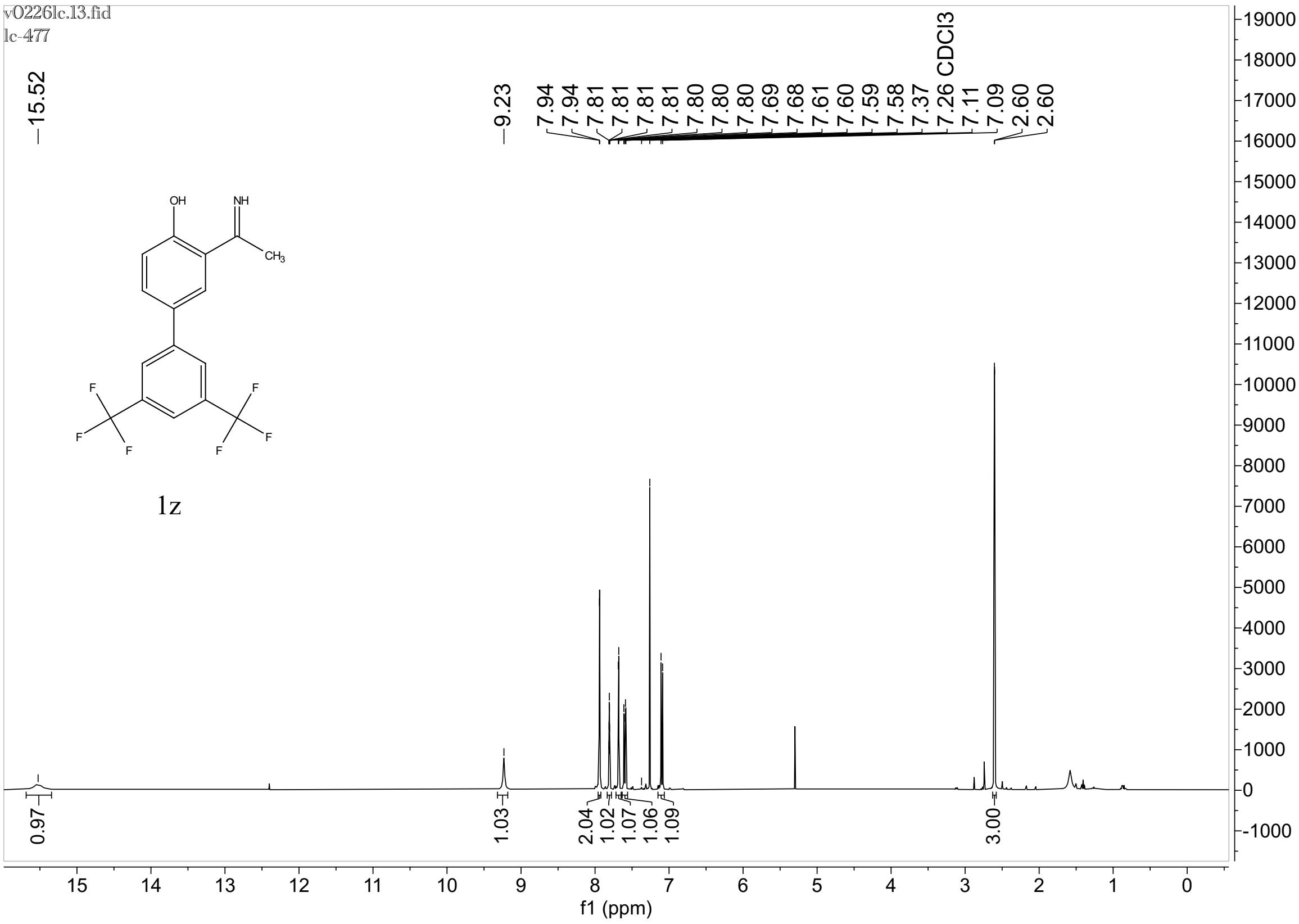
v0226lc.13.fid
lc-477
15.52
9.23
7.94
7.94
7.81
7.81
7.81
7.81
7.80
7.80
7.80
7.69
7.68
7.61
7.60
7.59
7.58
7.37
7.26 CDCl3
7.11
7.09
2.60
2.60
OH
NH
CH_3
F
1z
0.97
1.03
2.04
1.02
1.07
1.06
1.09
3.00
f1 (ppm)

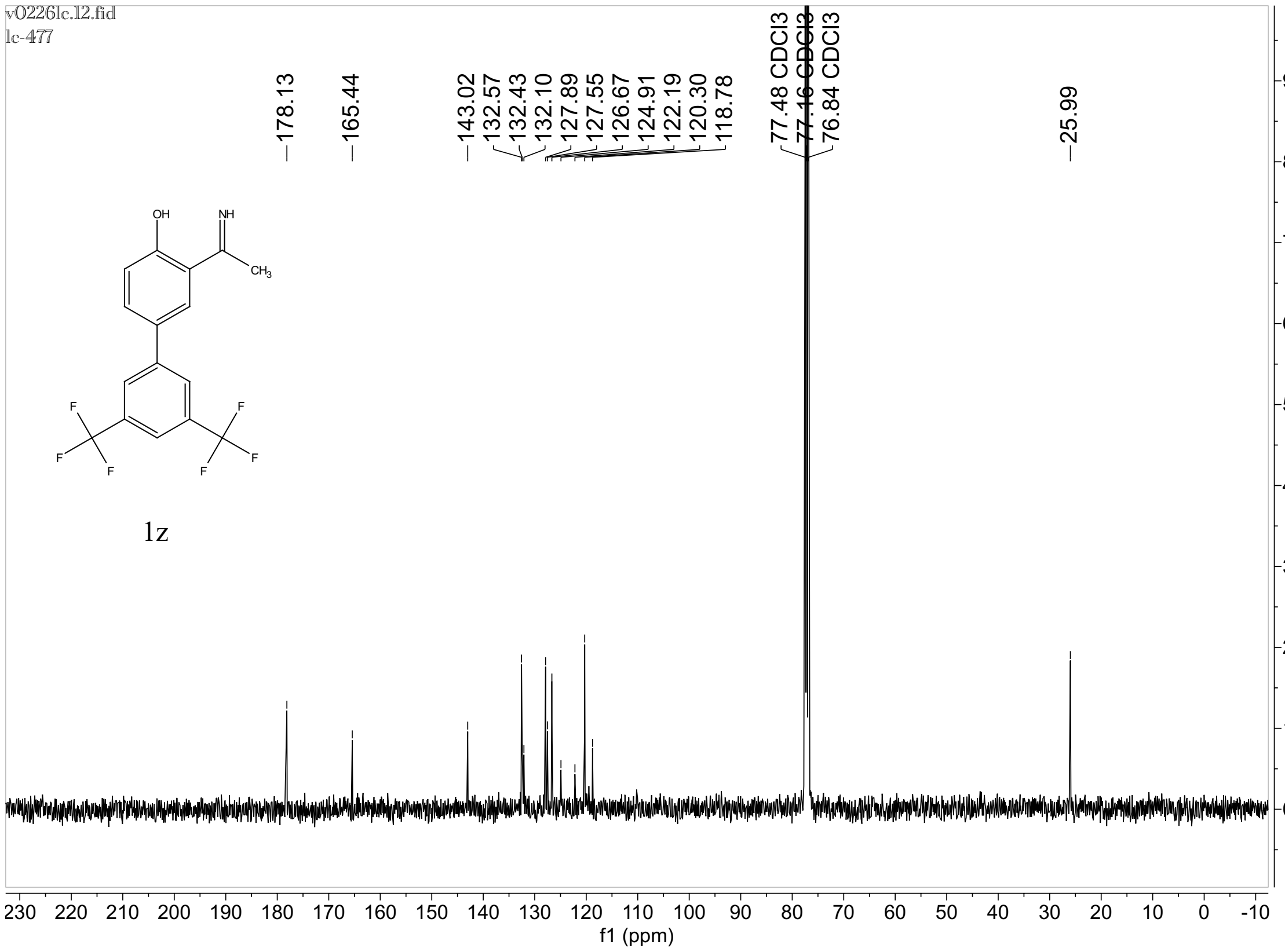
v0226lc.12.fid
lc-477
178.13
165.44
143.02
132.57
132.43
132.10
127.89
127.55
126.67
124.91
122.19
120.30
118.78
77.48 CDCl3
77.16 CDCl3
76.84 CDCl3
25.99
f1 (ppm)

1z

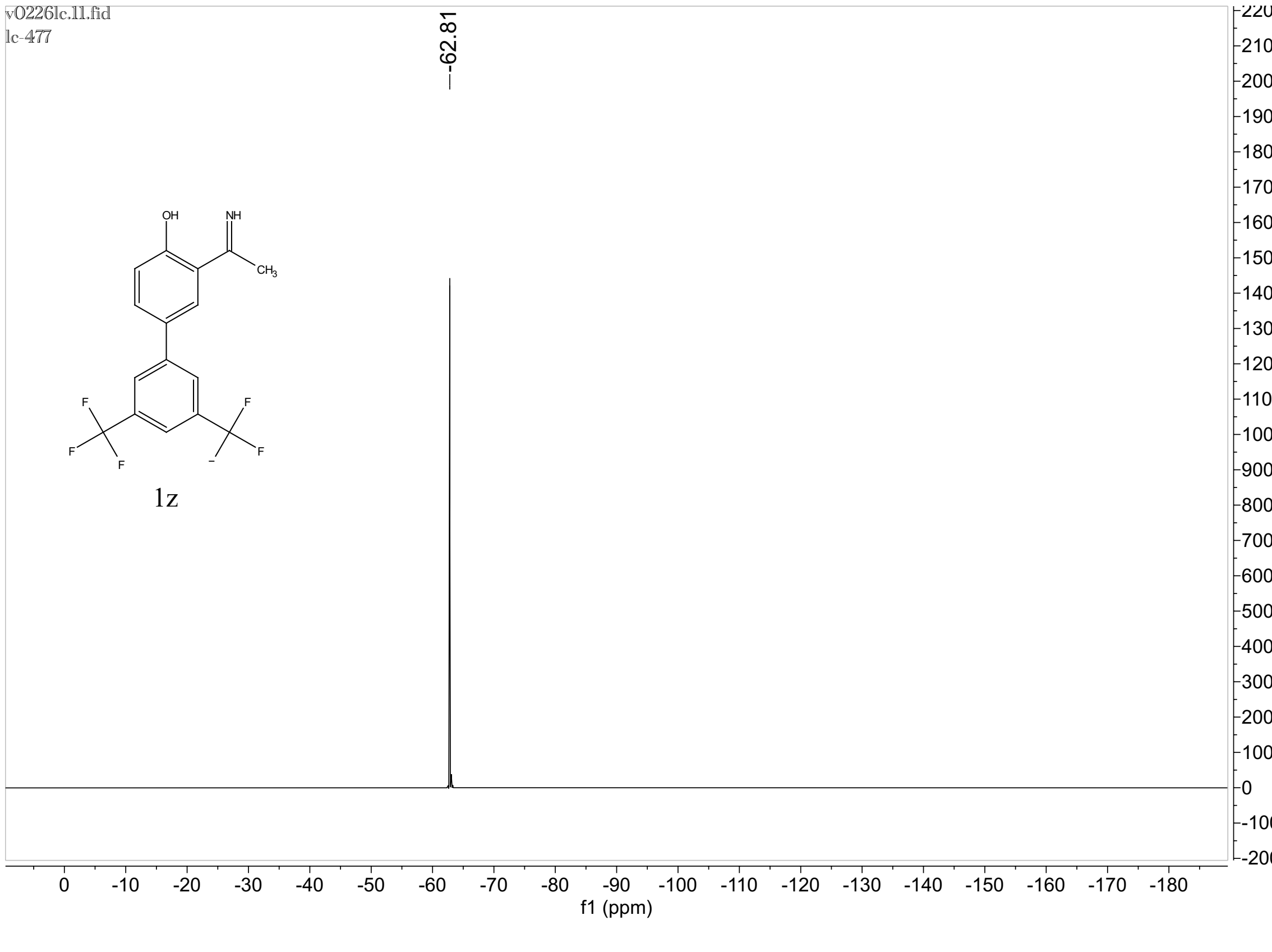
v0226lc.11.fid
lc-477
-62.81
f1 (ppm)

1z

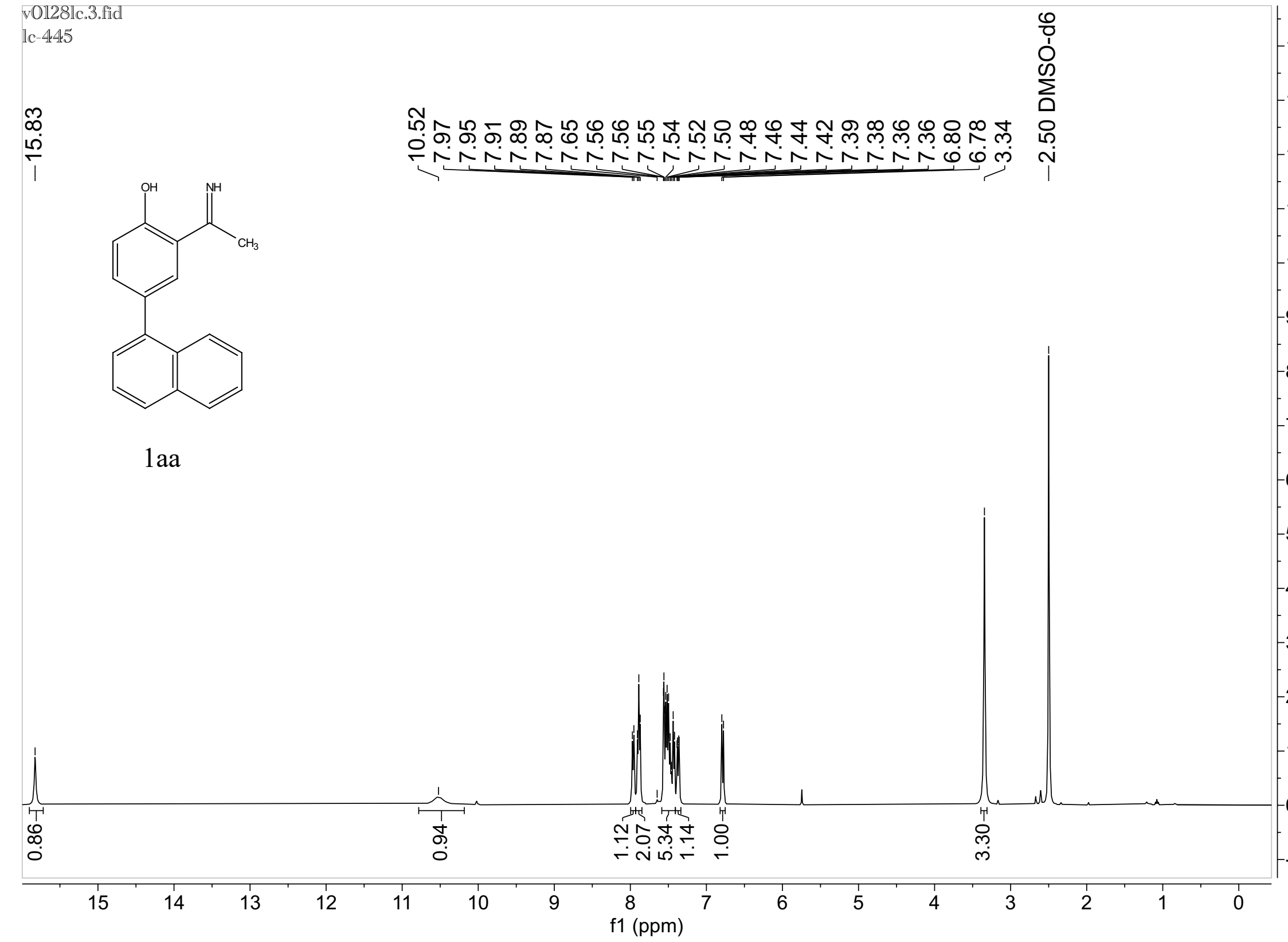

v0128lc.3.fid
lc-445
15.83
10.52
7.97
7.95
7.91
7.89
7.87
7.65
7.56
7.56
7.55
7.54
7.52
7.50
7.48
7.46
7.44
7.42
7.39
7.38
7.36
7.36
6.80
6.78
3.34
2.50 DMSO-d6
OH
NH
CH3
1aa
0.86
0.94
1.12
2.07
5.34
1.14
1.00
3.30
f1 (ppm)

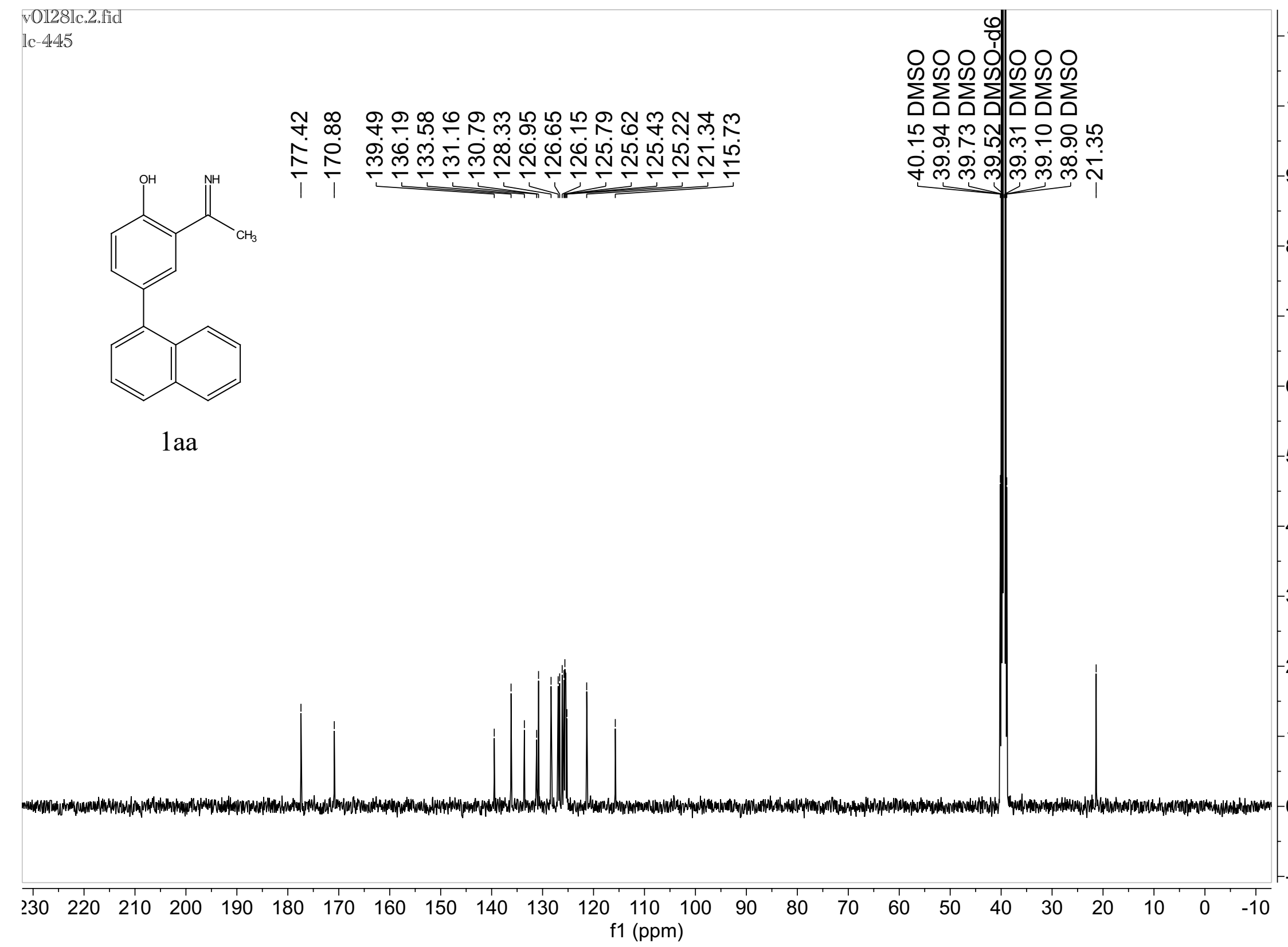

v0128lc.2.fid
lc-445
177.42
170.88
139.49
136.19
133.58
131.16
130.79
128.33
126.95
126.65
126.15
125.79
125.62
125.43
125.22
121.34
115.73
40.15 DMSO
39.94 DMSO
39.73 DMSO
39.52 DMSO-d6
39.31 DMSO
39.10 DMSO
38.90 DMSO
21.35
OH
NH
CH3
1aa
f1 (ppm)

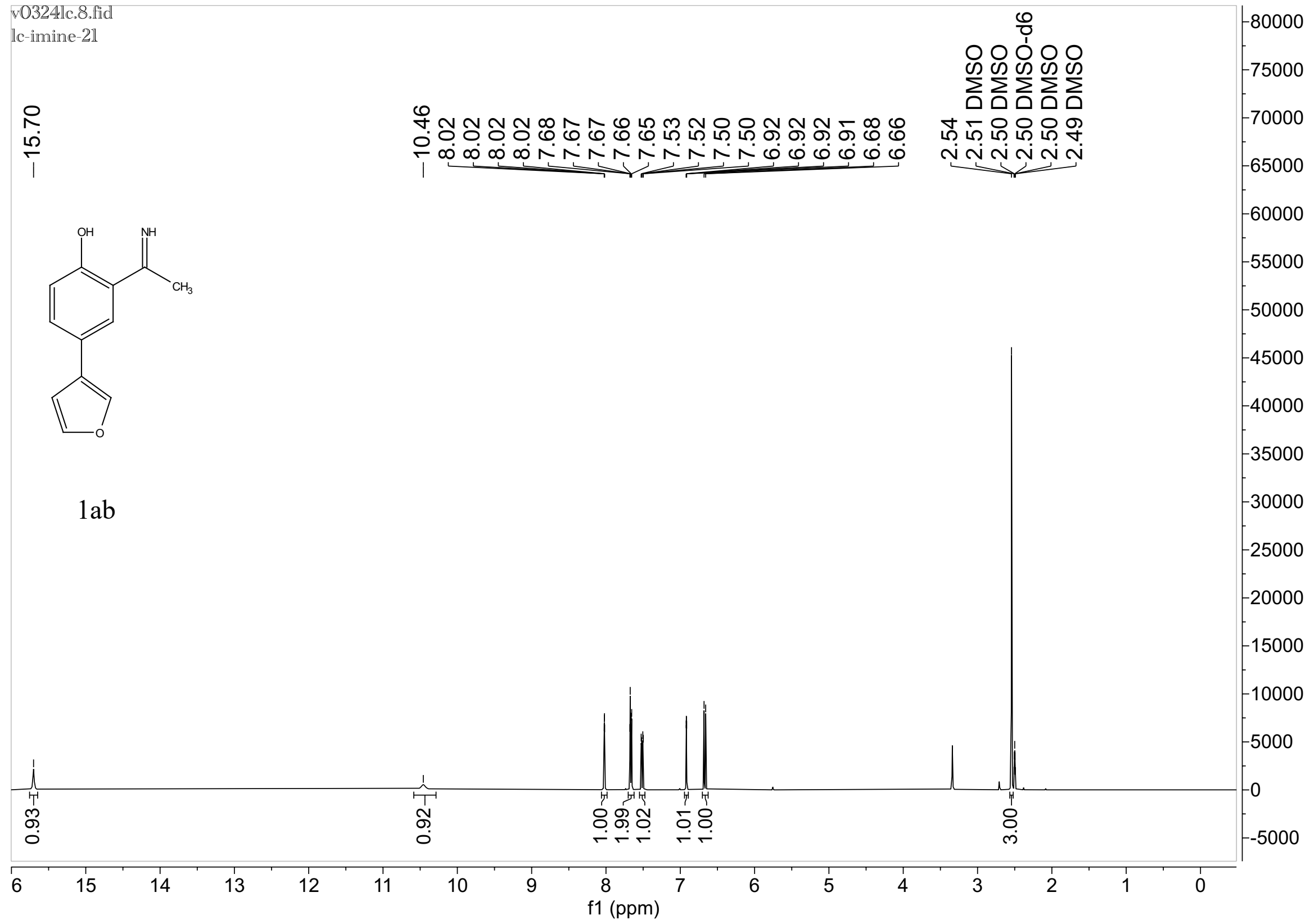
v0324lc.8.fid
lc-imine-21
1ab
f1 (ppm)

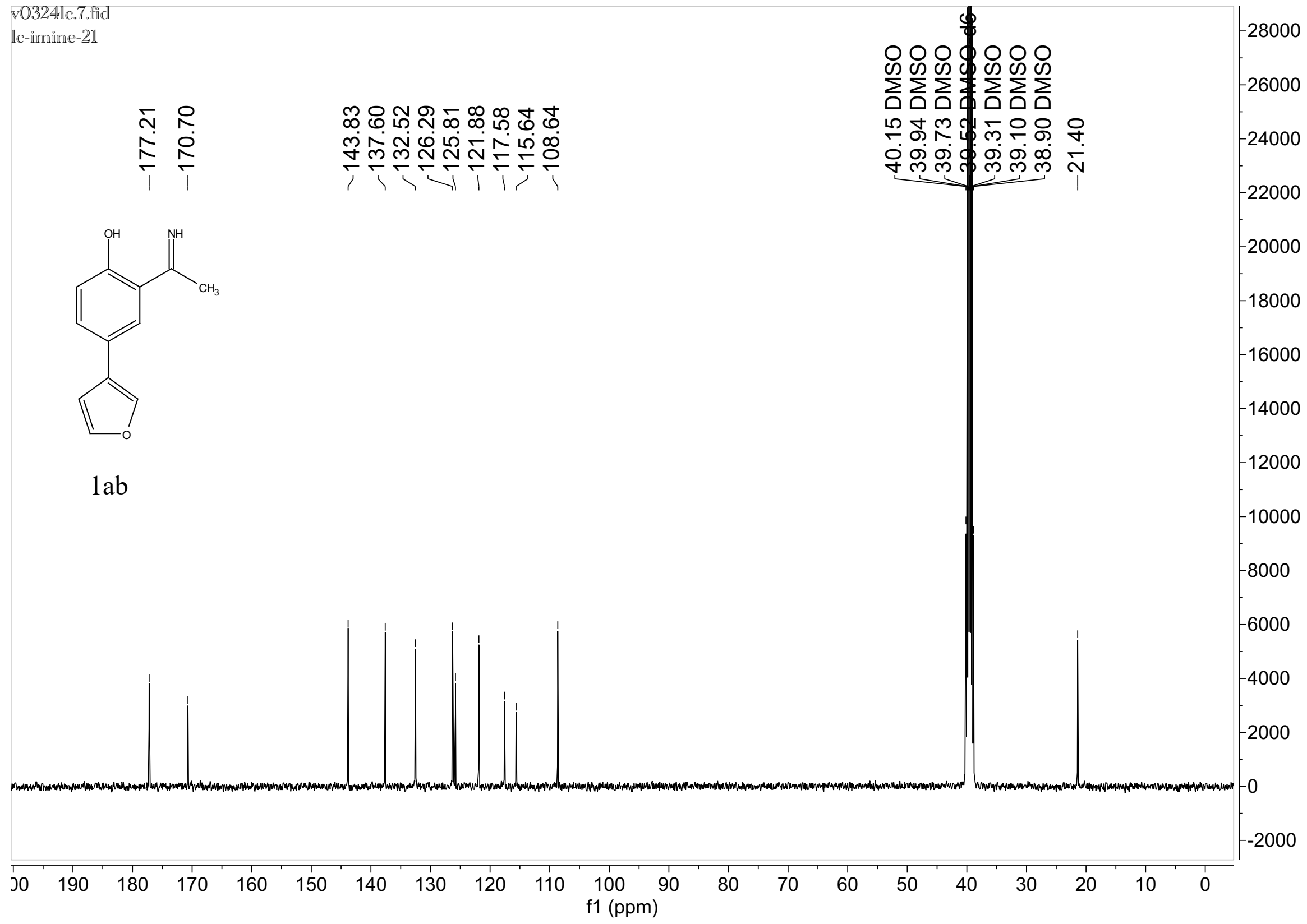
v0324lc.7.fid
lc-imine-21
1ab
f1 (ppm)

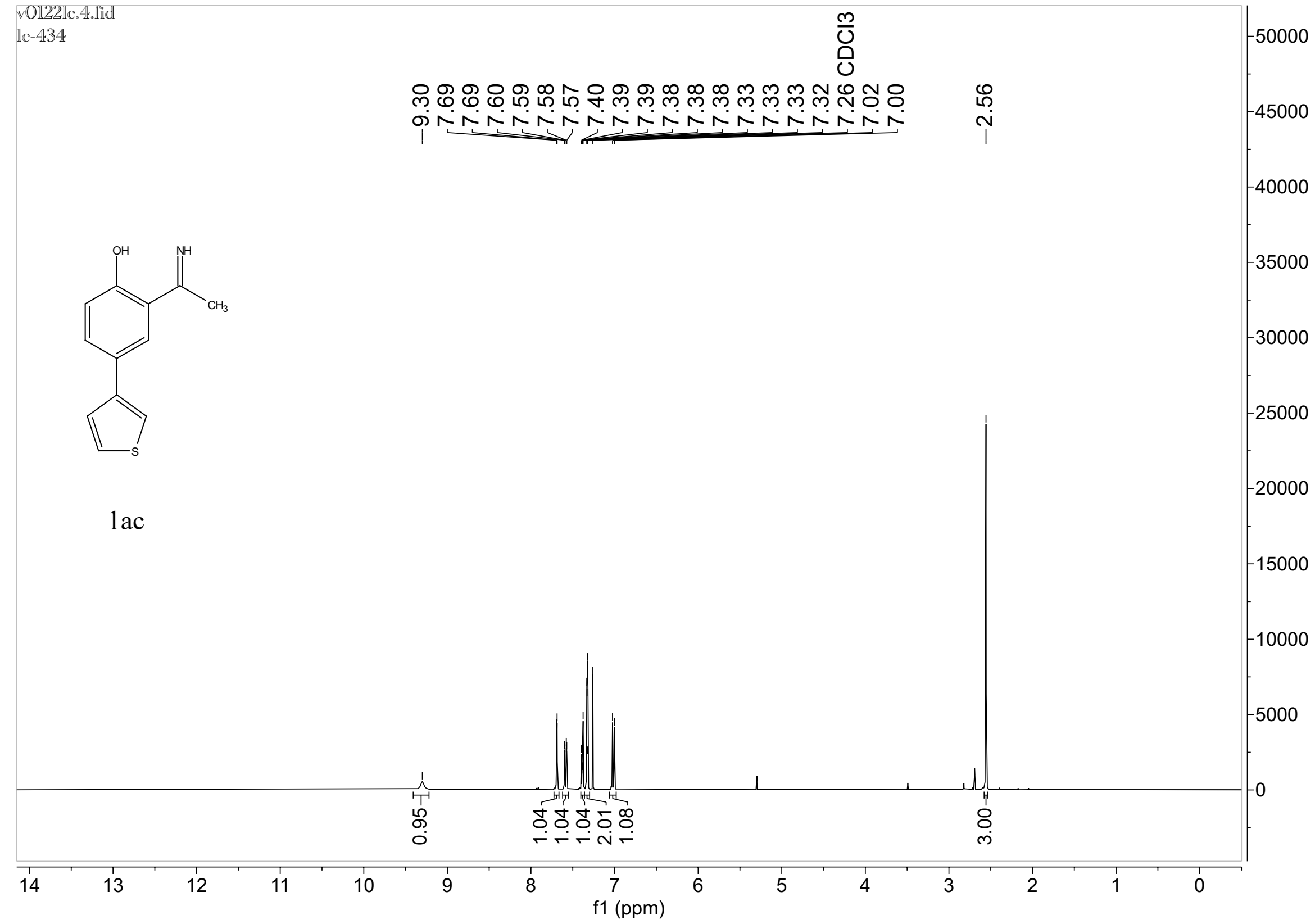
v0122lc.4.fid
lc-434
9.30
7.69
7.69
7.60
7.59
7.58
7.57
7.40
7.39
7.39
7.38
7.38
7.38
7.33
7.33
7.33
7.32
7.26 CDCl3
7.02
7.00
2.56
OH
NH
CH3
S
1ac
0.95
1.04
1.04
1.04
2.01
1.08
3.00
f1 (ppm)

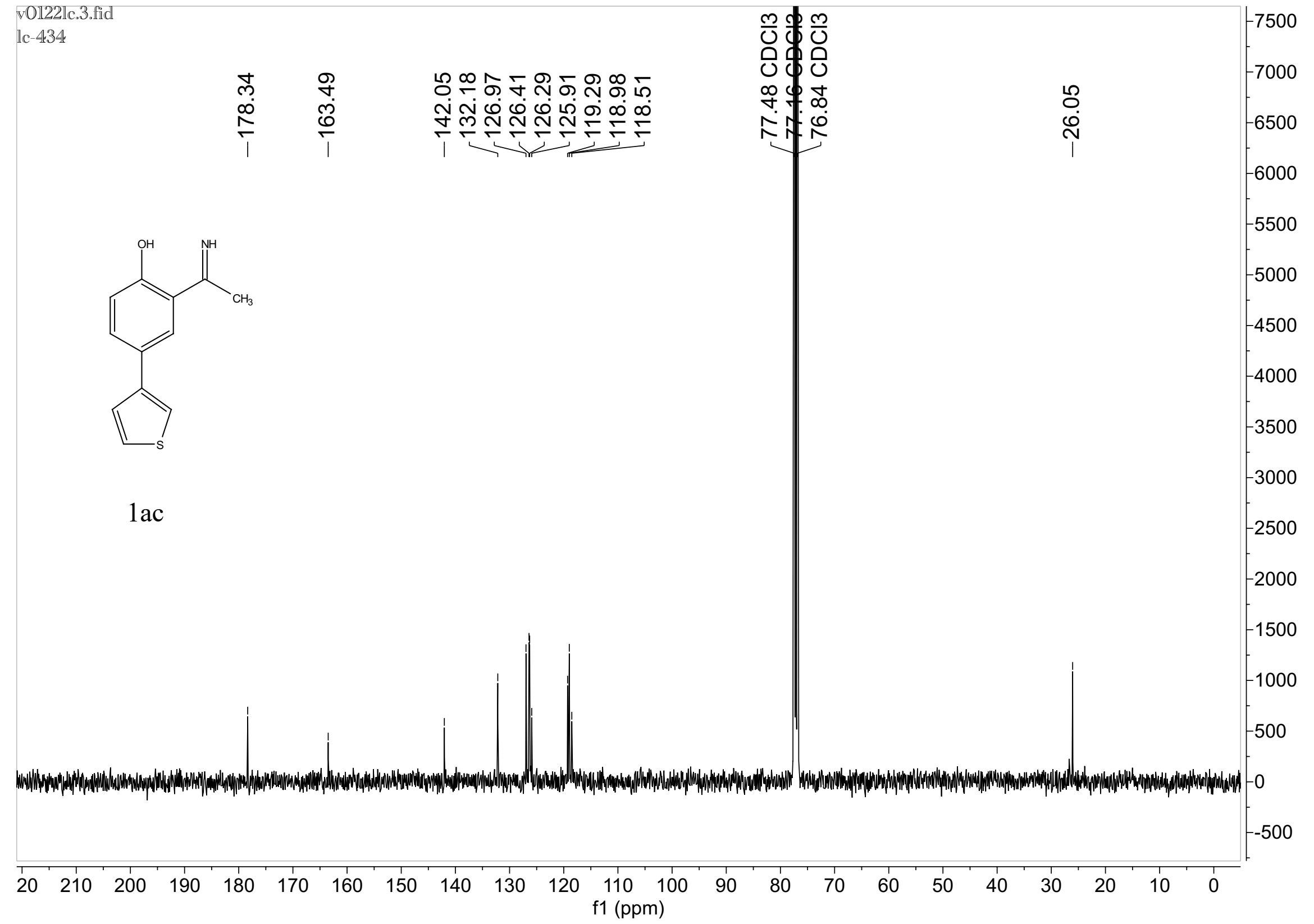
v0122lc.3.fid
lc-434
178.34
163.49
142.05
132.18
126.97
126.41
126.29
125.91
119.29
118.98
118.51
77.48 CDCl3
77.16 CDCl3
76.84 CDCl3
26.05
OH
NH
CH3
S
1ac
f1 (ppm)

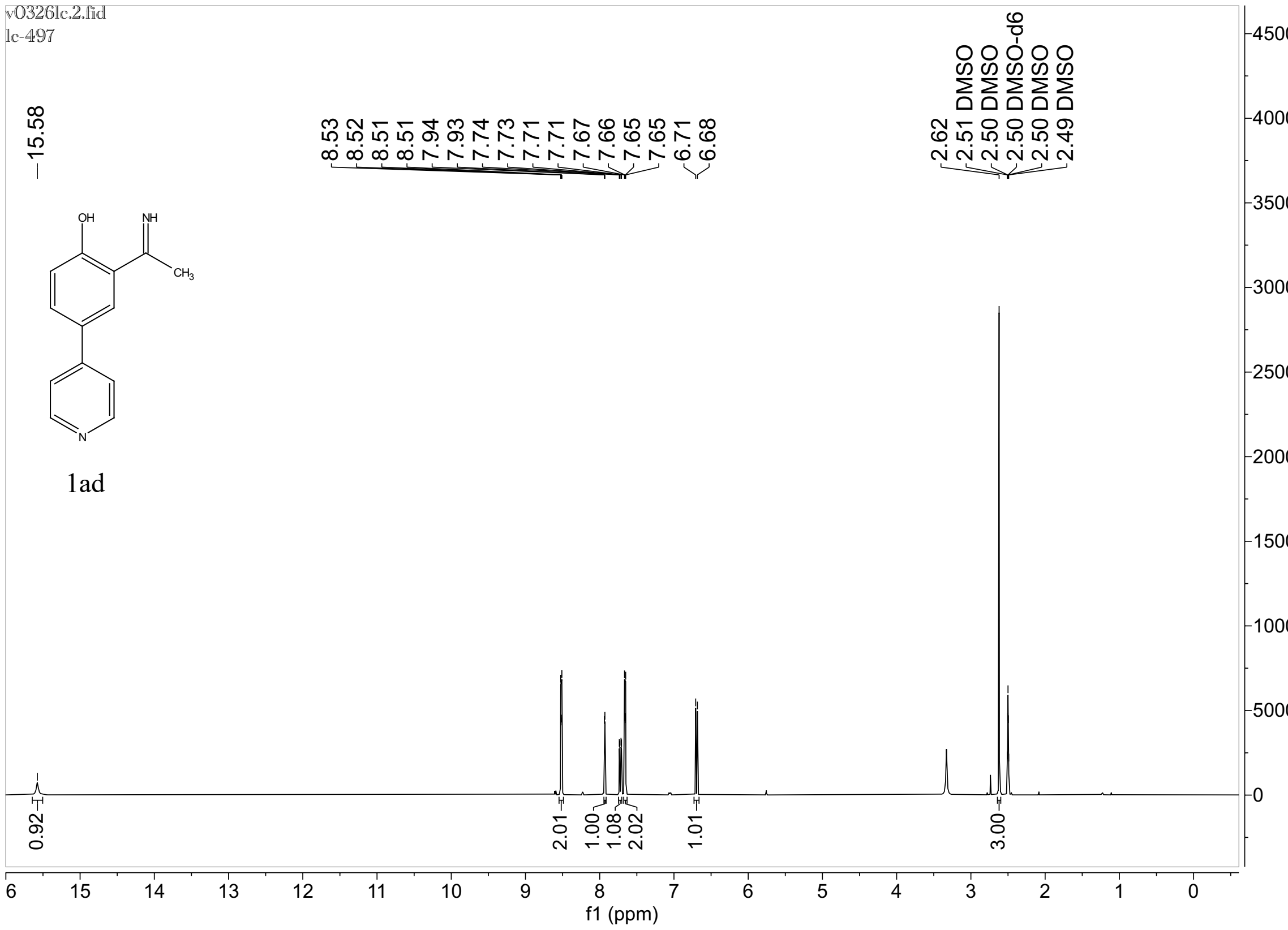
v0326lc.2.fid
lc-497
1ad
f1 (ppm)

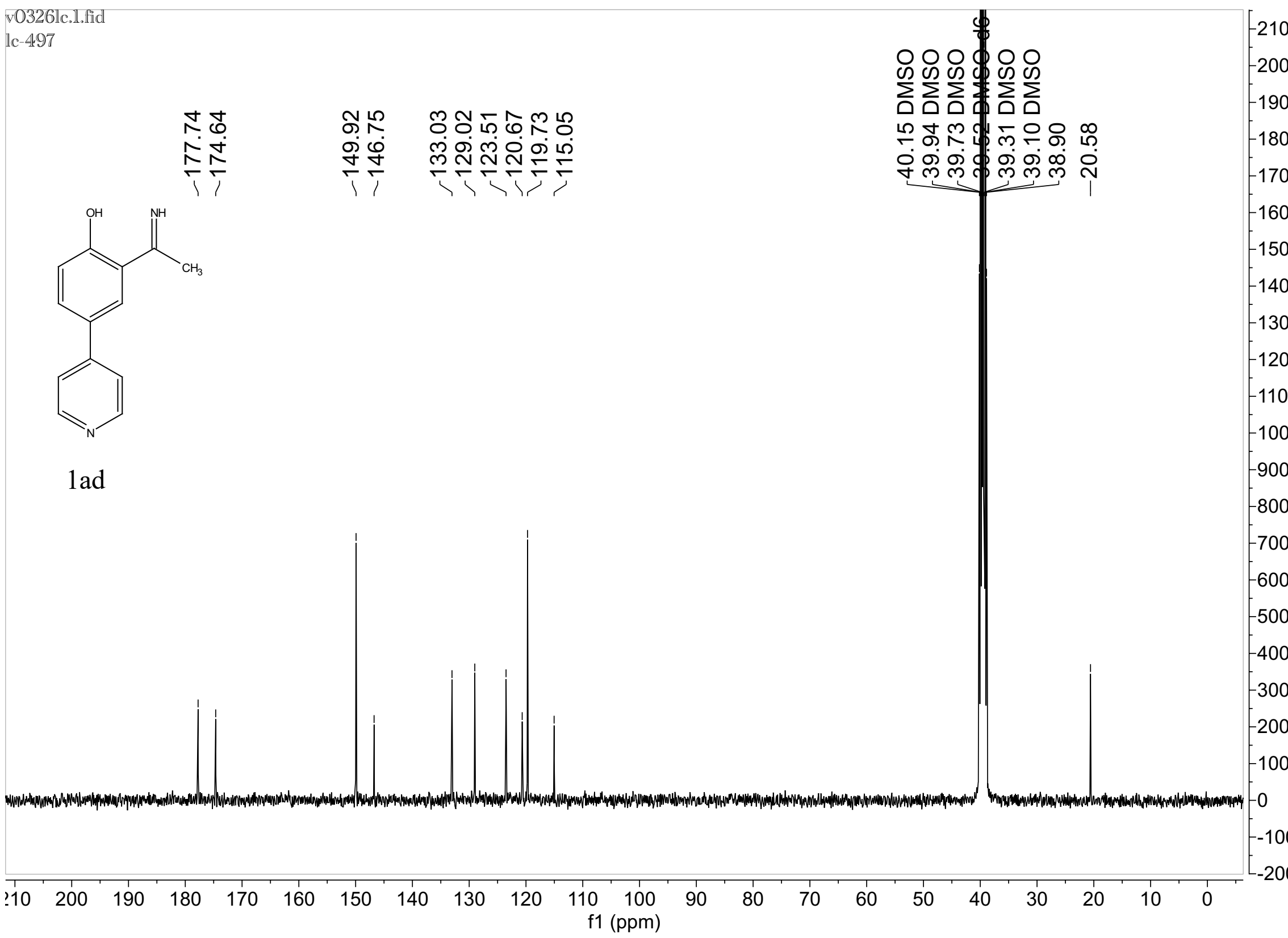
v0326lc.1.fid
lc-497
1ad
f1 (ppm)

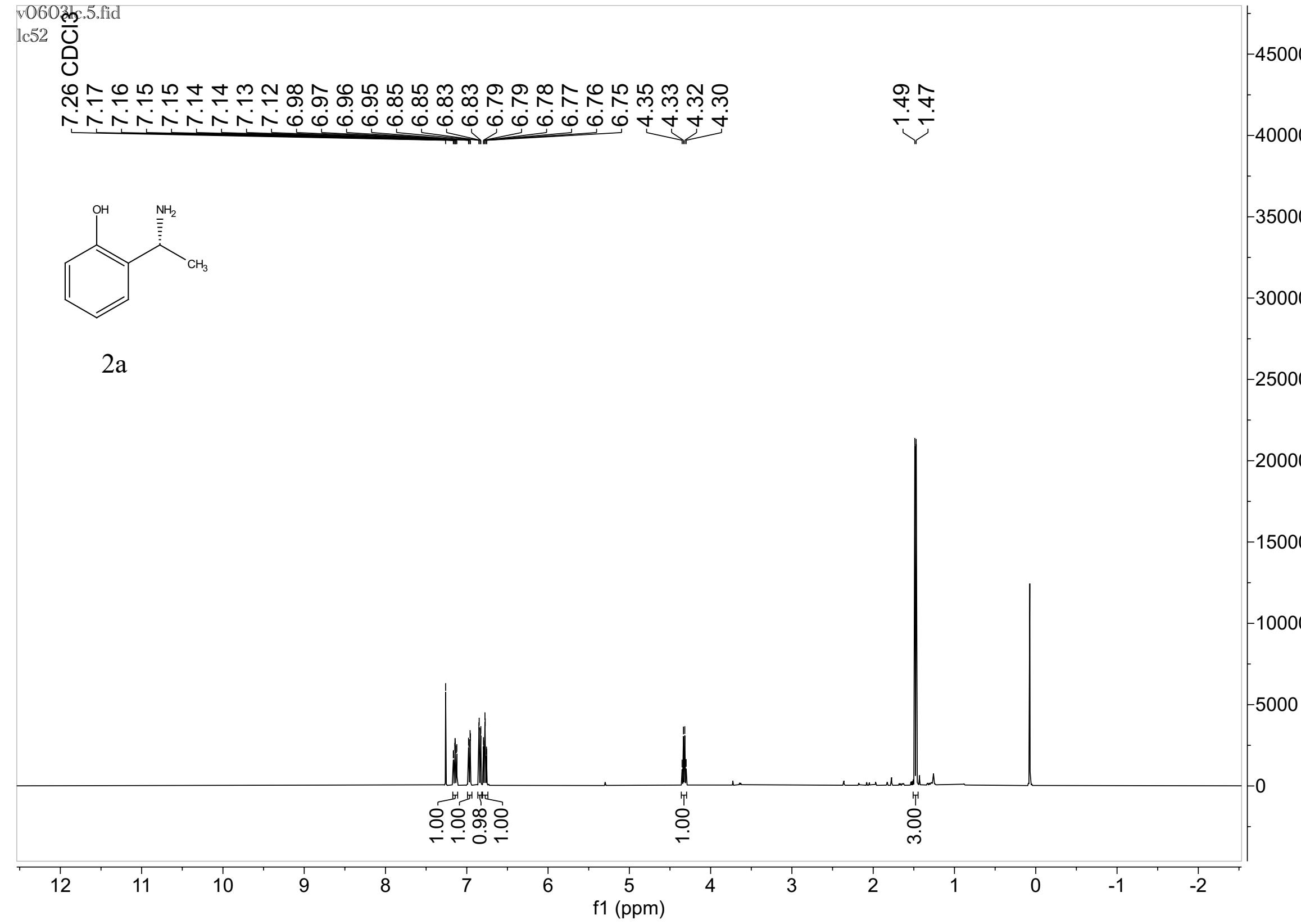

v0603lc.5.fid
lc52
7.26 CDCl3
7.17
7.16
7.15
7.15
7.14
7.14
7.13
7.12
6.98
6.97
6.96
6.95
6.85
6.85
6.83
6.83
6.79
6.79
6.78
6.77
6.76
6.75
4.35
4.33
4.32
4.30
1.49
1.47
OH
NH2
CH3
2a
1.00
1.00
0.98
1.00
1.00
3.00
f1 (ppm)

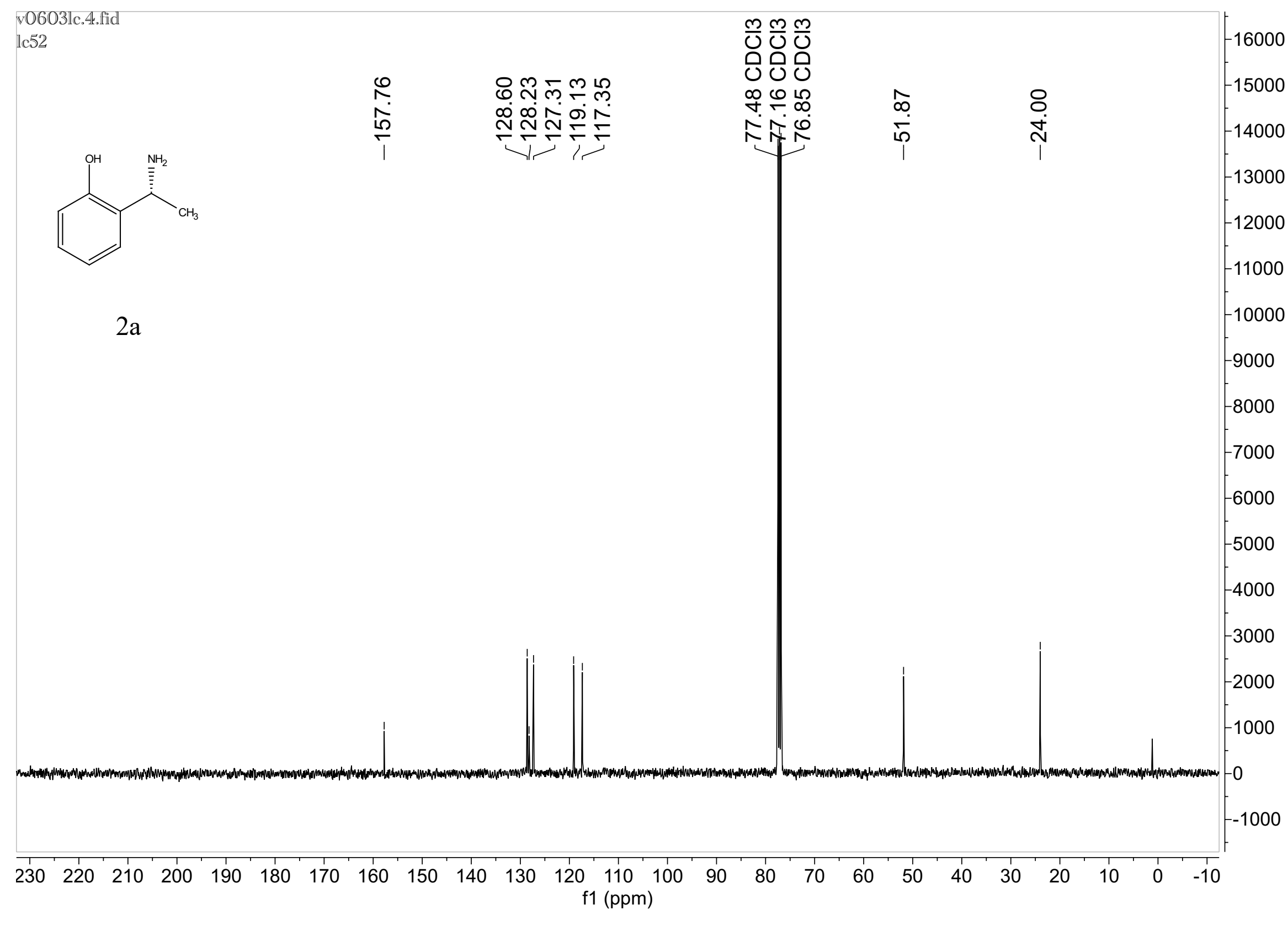

v0603lc.4.fid
lc52
157.76
128.60
128.23
127.31
119.13
117.35
77.48 CDCl3
77.16 CDCl3
76.85 CDCl3
51.87
24.00
OH
NH2
CH3
2a
f1 (ppm)

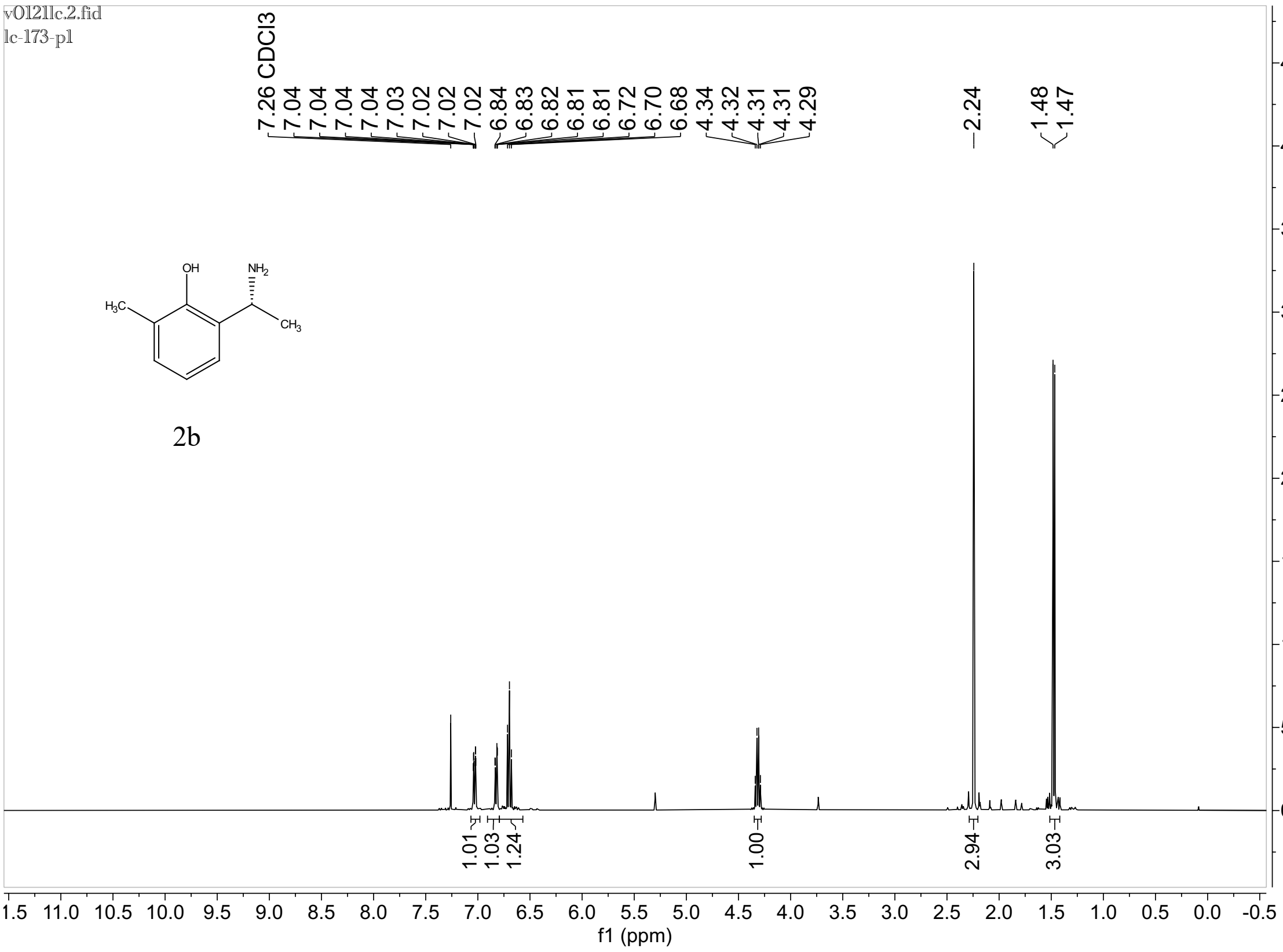
v0121lc.2.fid
lc-173-p1
7.26 CDCl3
7.04
7.04
7.04
7.04
7.03
7.02
7.02
7.02
6.84
6.83
6.82
6.81
6.81
6.72
6.70
6.68
4.34
4.32
4.31
4.31
4.29
2.24
1.48
1.47
OH
NH2
H3C
CH3
2b
1.01
1.03
1.24
1.00
2.94
3.03
f1 (ppm)

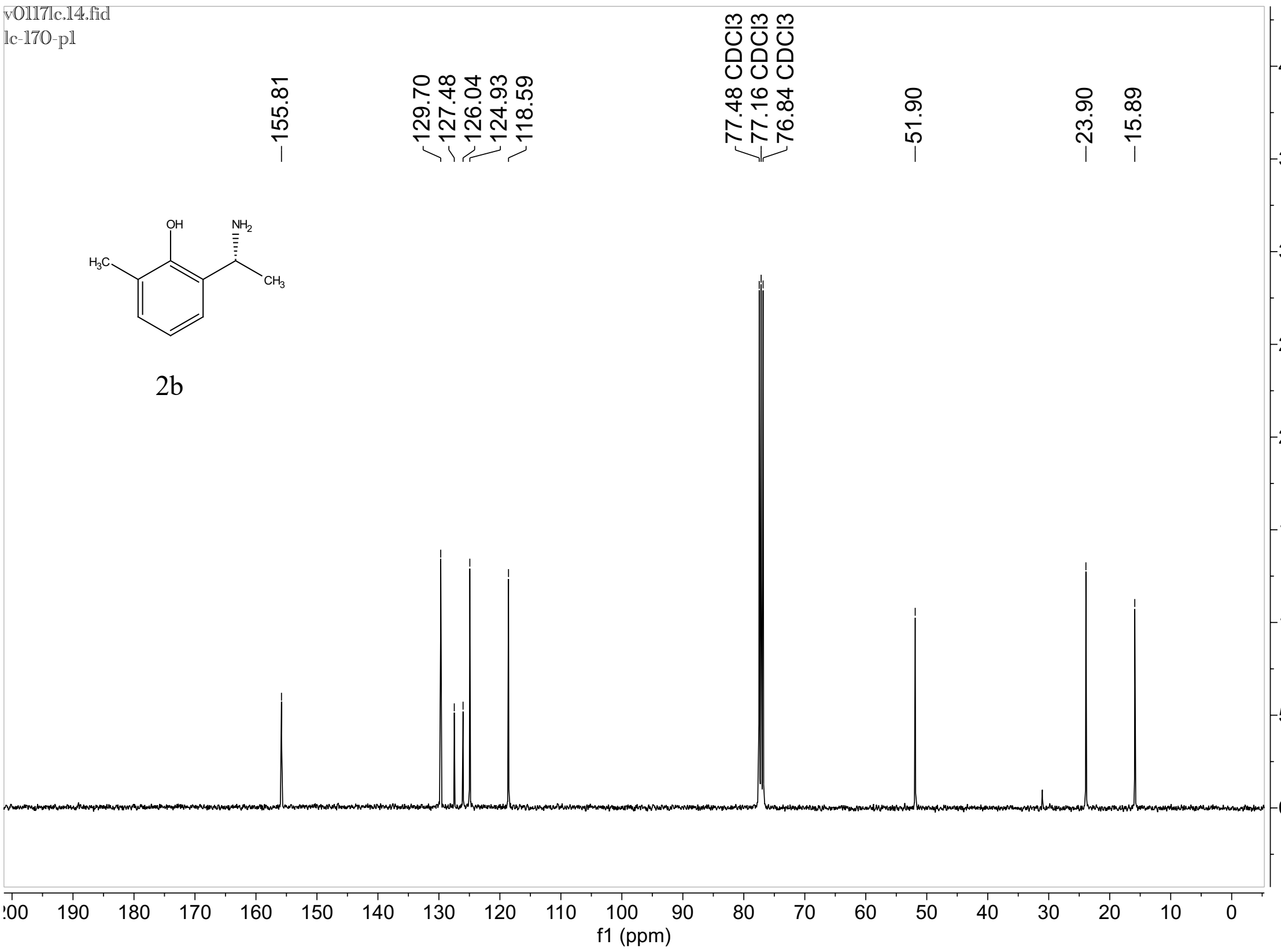
v0117lc.14.fid
lc-170-p1
155.81
129.70
127.48
126.04
124.93
118.59
77.48 CDCl3
77.16 CDCl3
76.84 CDCl3
51.90
23.90
15.89
OH
NH2
H3C
CH3
2b
f1 (ppm)

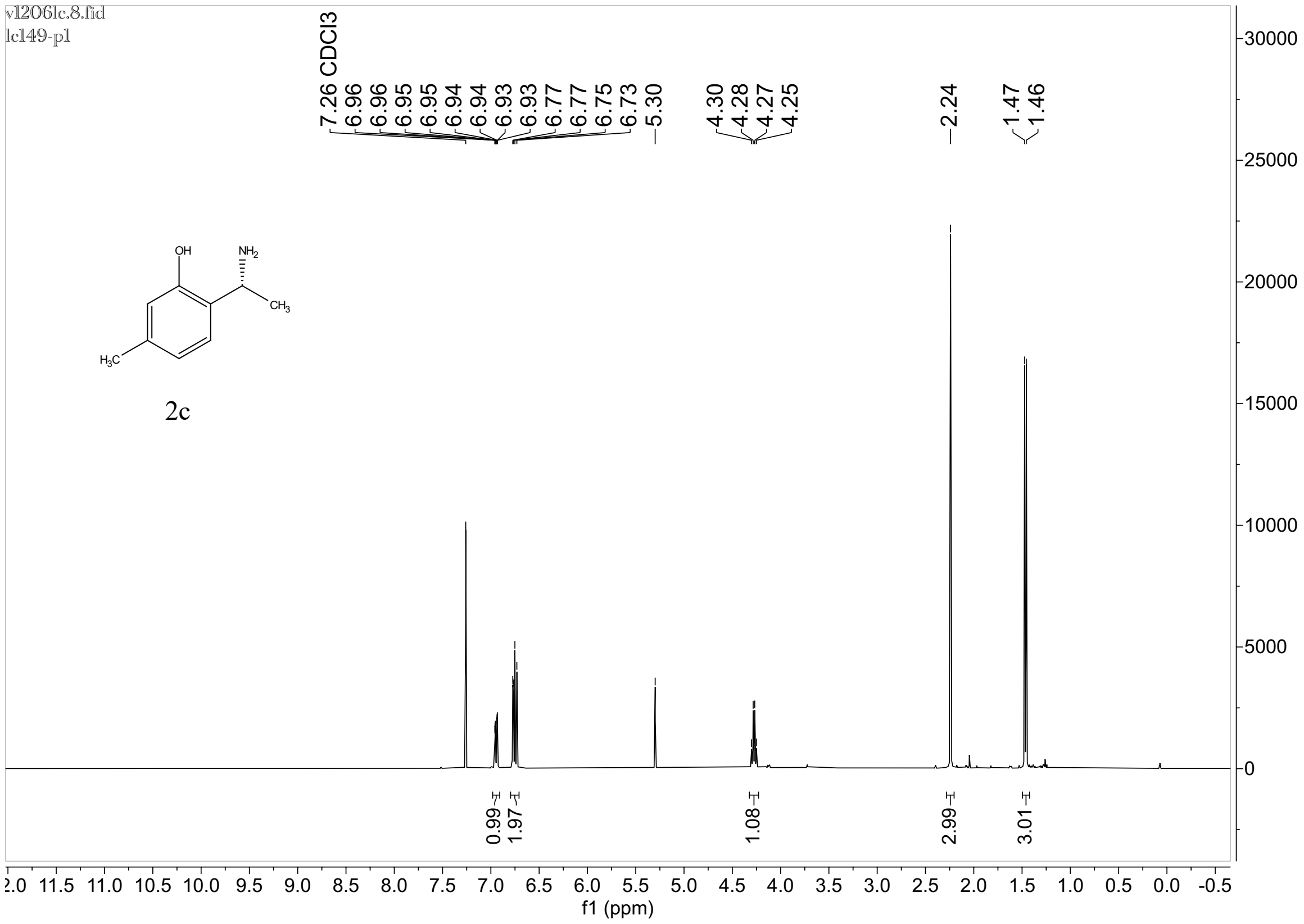

2c

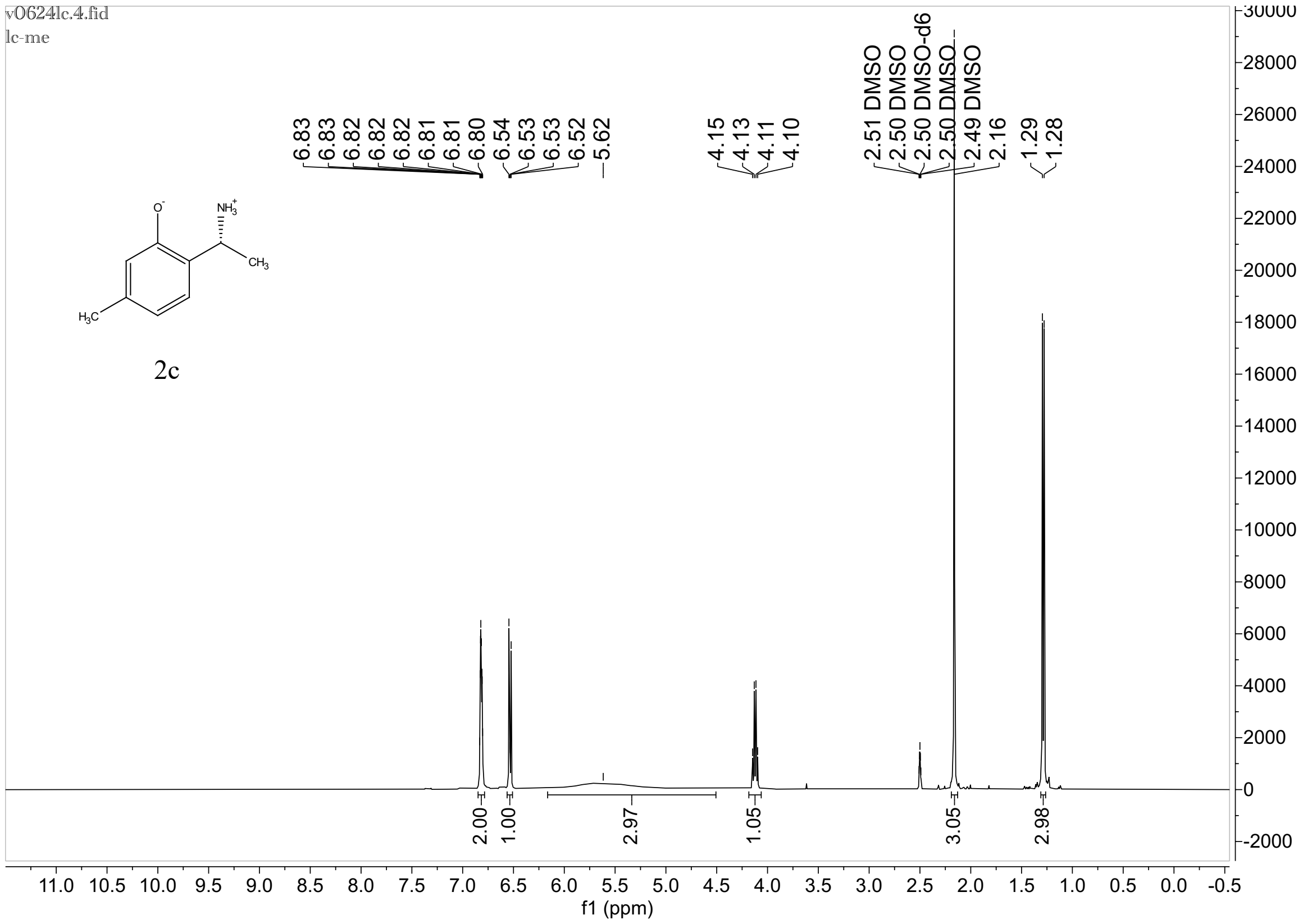

2c

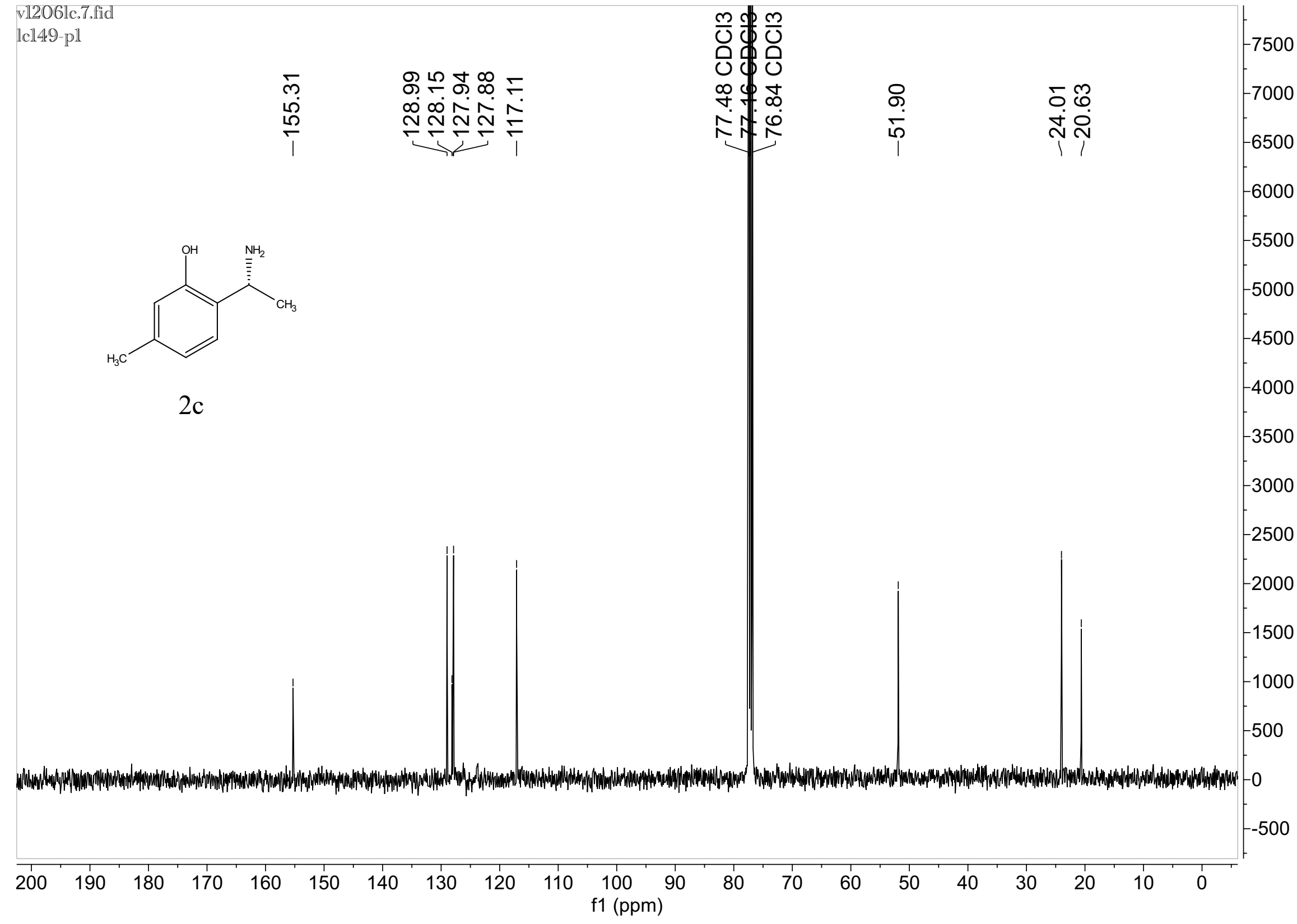

vl206lc.7.fid
lc149-p1
155.31
128.99
128.15
127.94
127.88
117.11
77.48 CDCl3
77.16 CDCl3
76.84 CDCl3
51.90
24.01
20.63
OH
NH2
CH3
H3C
2c
f1 (ppm)

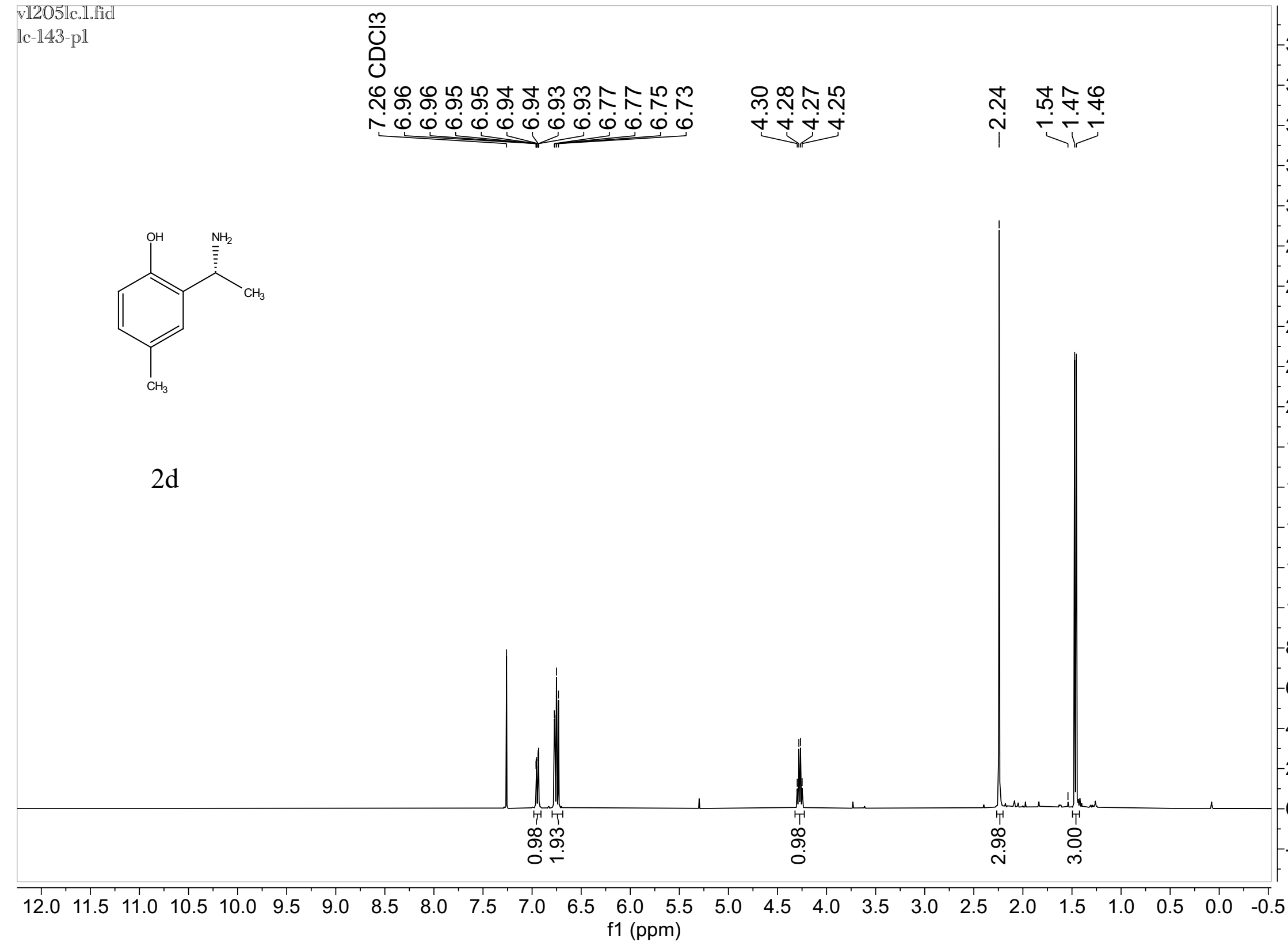
vl205lc.1.fid
lc-143-p1
7.26 CDCl3
6.96
6.96
6.95
6.95
6.94
6.94
6.93
6.93
6.77
6.77
6.75
6.73
4.30
4.28
4.27
4.25
2.24
1.54
1.47
1.46
OH
NH2
CH3
CH3
2d
0.98
1.93
0.98
2.98
3.00
f1 (ppm)

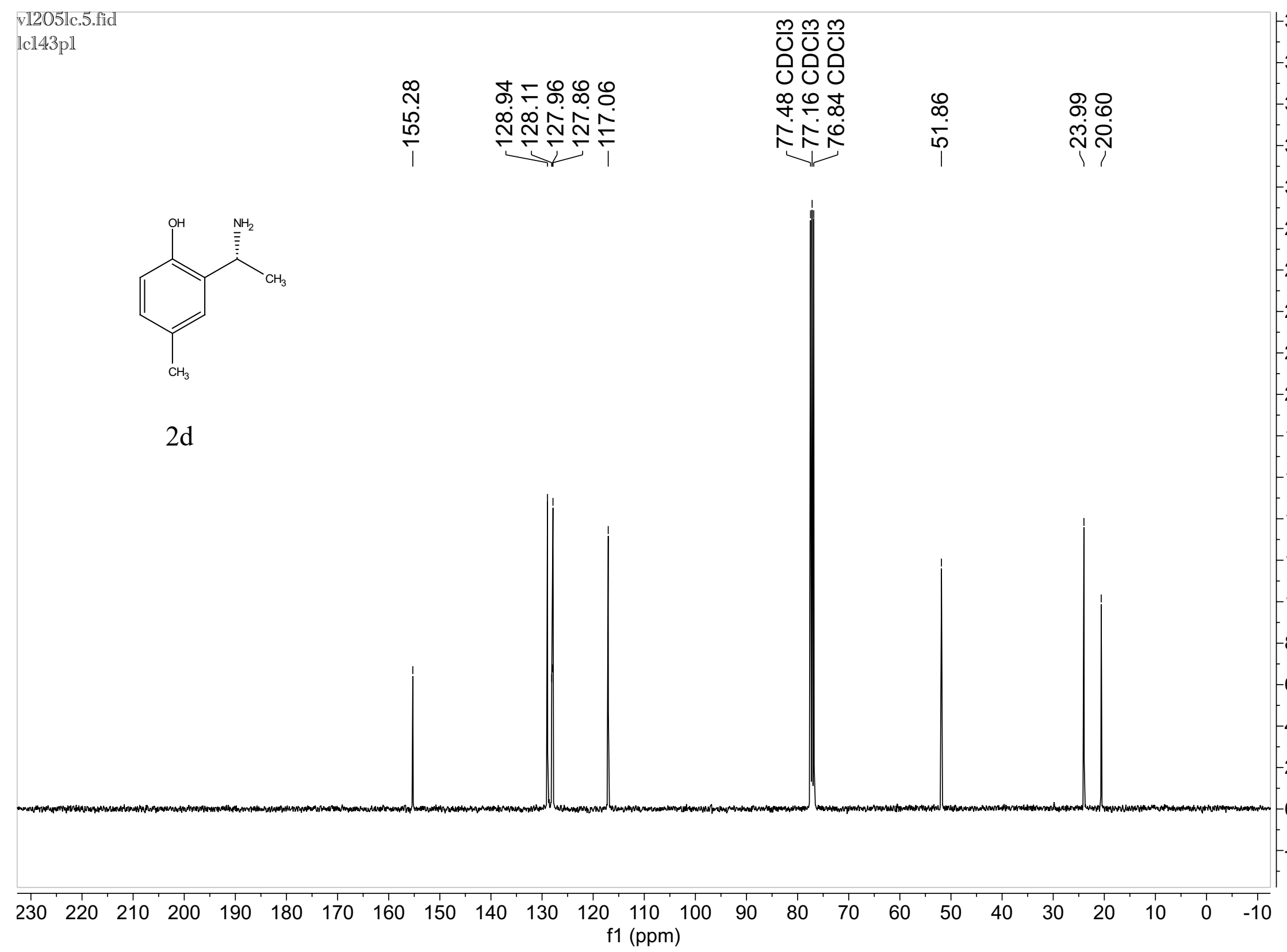
vl205lc.5.fid
lc143p1
155.28
128.94
128.11
127.96
127.86
117.06
77.48 CDCl3
77.16 CDCl3
76.84 CDCl3
51.86
23.99
20.60
OH
NH2
CH3
CH3
2d
f1 (ppm)

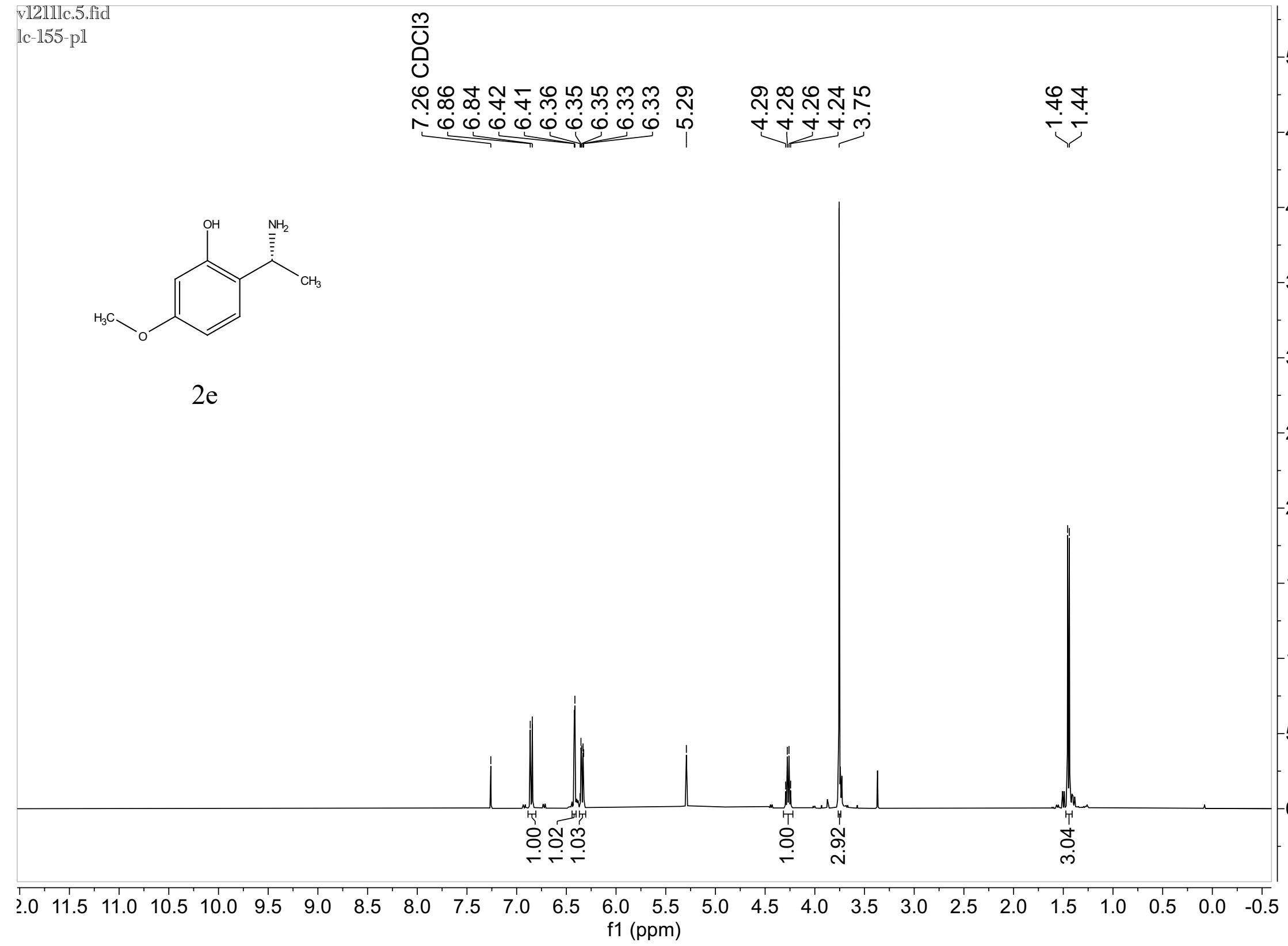
vl211lc.5.fid
lc-155-p1
7.26 CDCl3
6.86
6.84
6.42
6.41
6.36
6.35
6.35
6.33
6.33
5.29
4.29
4.28
4.26
4.24
3.75
1.46
1.44
OH
NH2
CH3
H3C
O
2e
1.00
1.02
1.03
1.00
2.92
3.04
f1 (ppm)

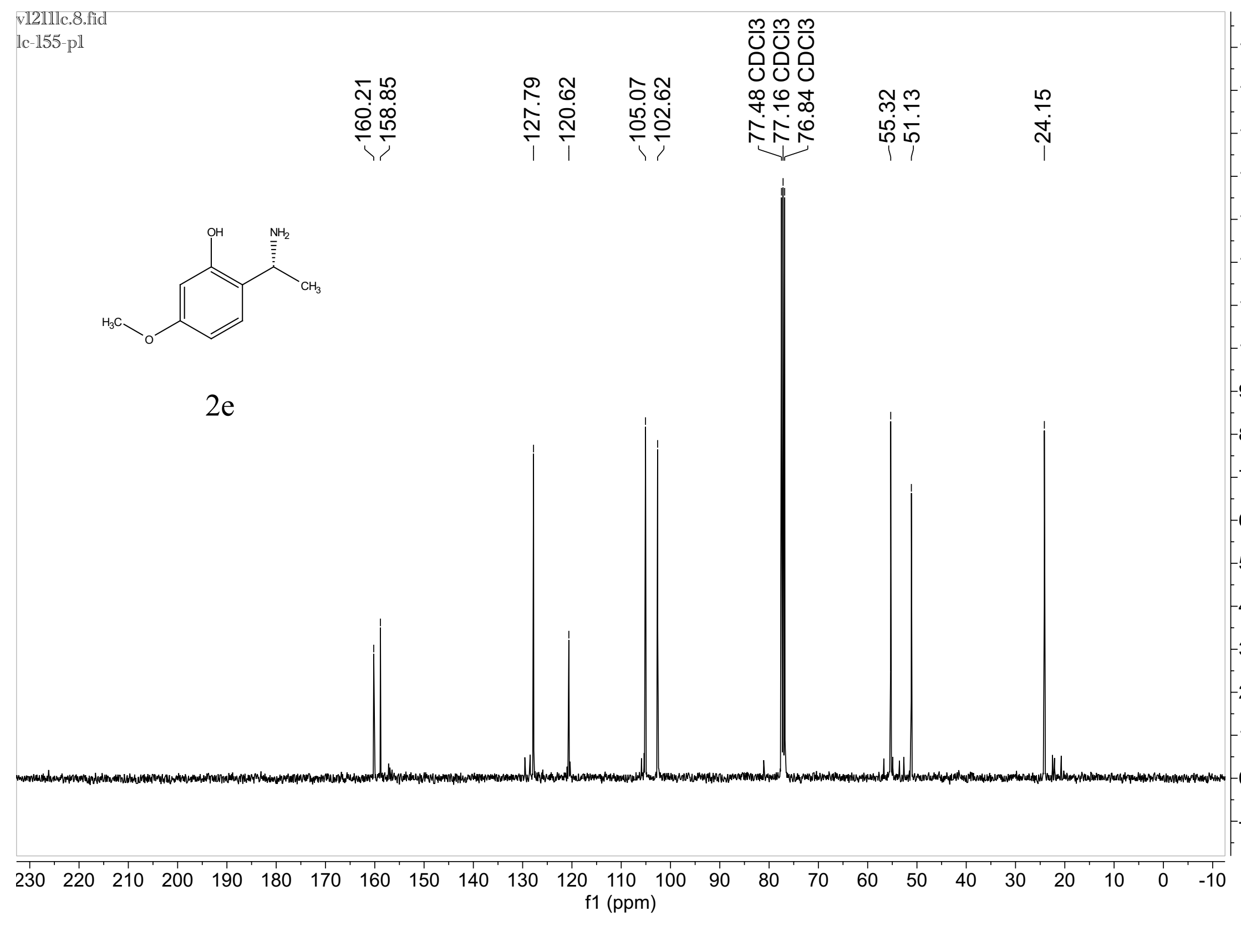
vl211lc.8.fid
lc-155-p1
160.21
158.85
127.79
120.62
105.07
102.62
77.48 CDCl3
77.16 CDCl3
76.84 CDCl3
55.32
51.13
24.15
OH
NH2
CH3
H3C
O
2e
f1 (ppm)

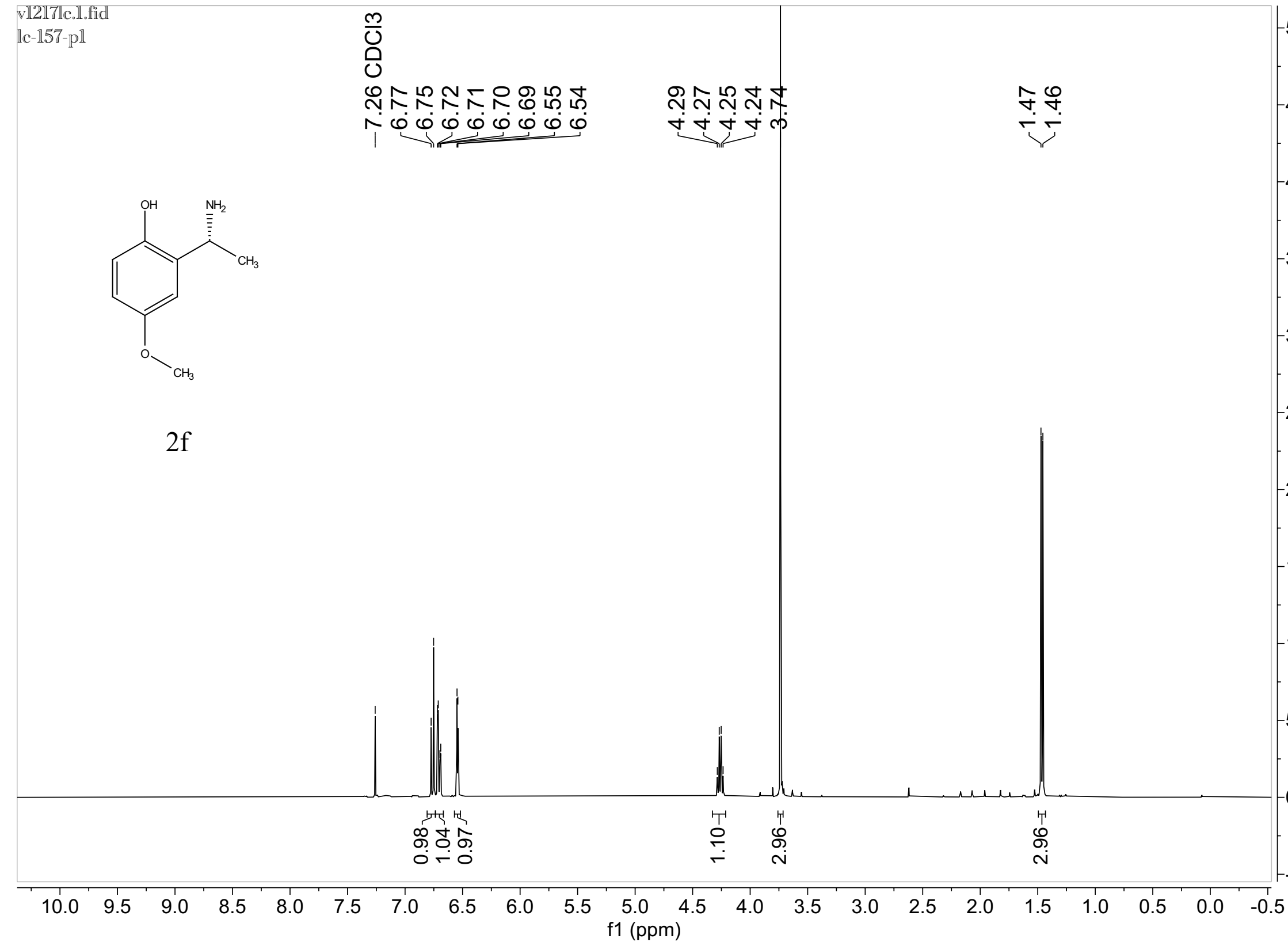
v1217lc.1.fid
lc-157-p1
7.26 CDCl3
6.77
6.75
6.72
6.71
6.70
6.69
6.55
6.54
4.29
4.27
4.25
4.24
3.74
1.47
1.46
OH
NH2
CH3
O
CH3
2f
0.98
1.04
0.97
1.10
2.96
2.96
f1 (ppm)

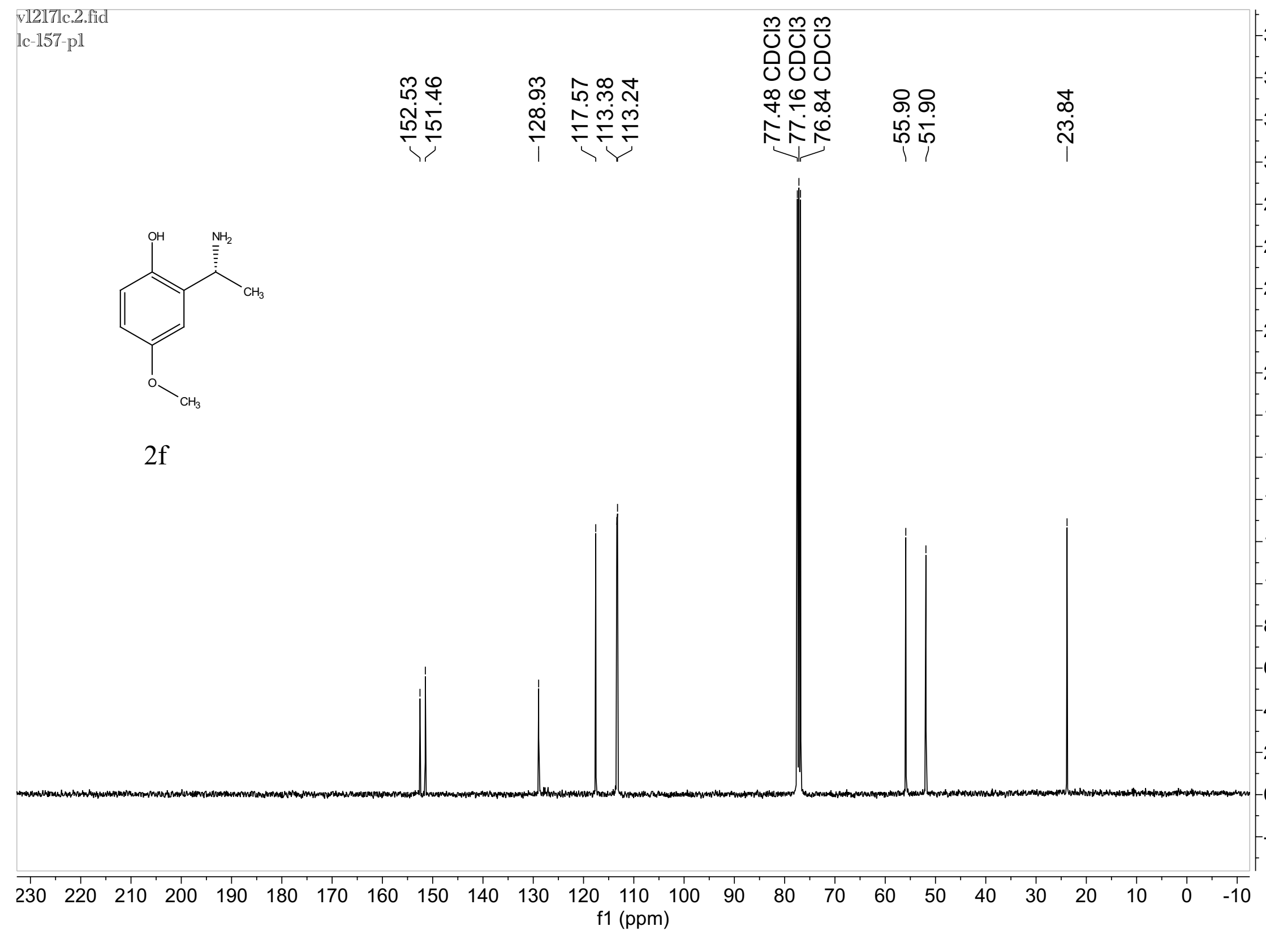
v1217lc.2.fid
lc-157-p1
152.53
151.46
128.93
117.57
113.38
113.24
77.48 CDCl3
77.16 CDCl3
76.84 CDCl3
55.90
51.90
23.84
OH
NH2
CH3
O
CH3
2f
f1 (ppm)

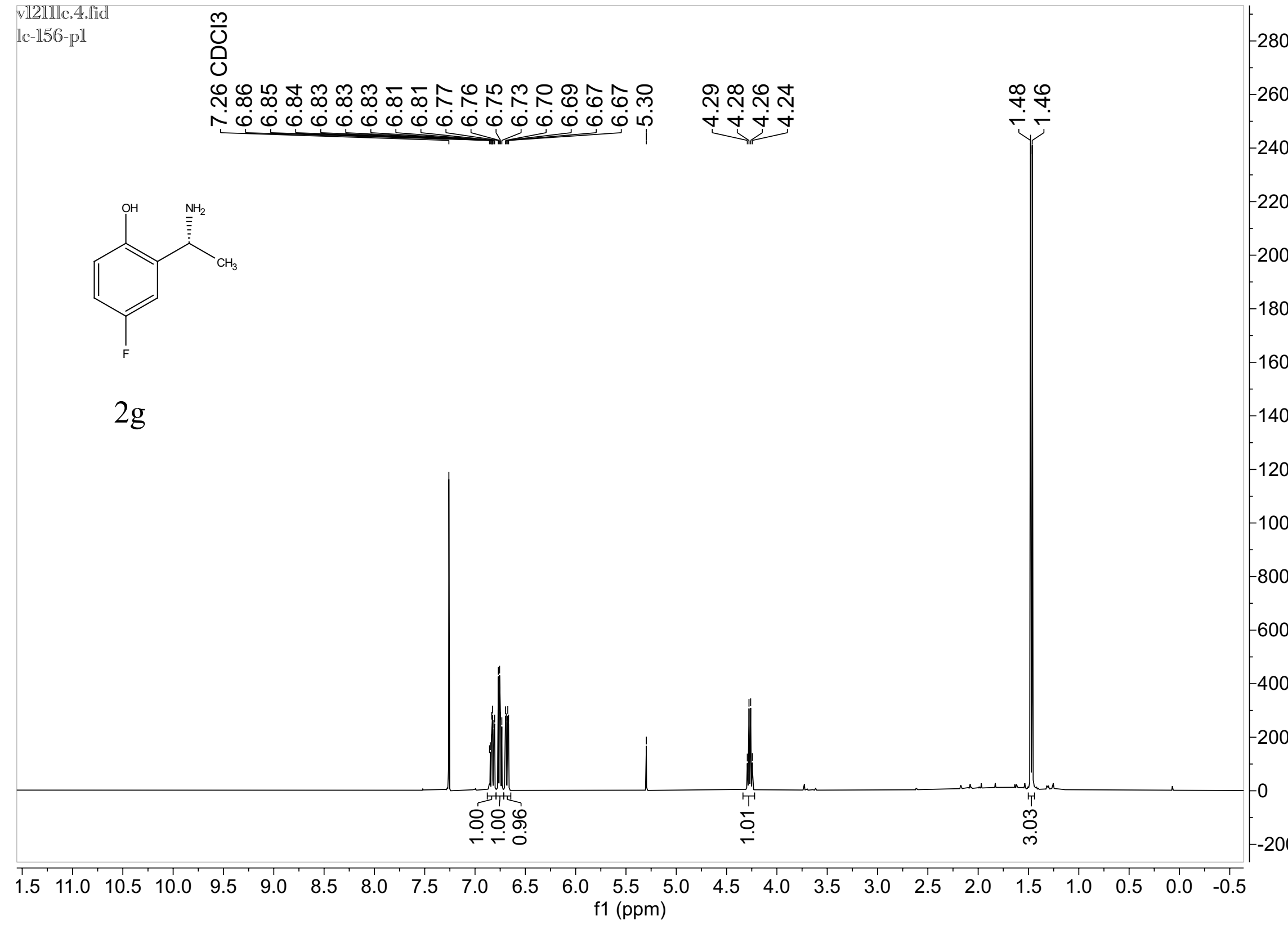
v1211lc.4.fid
lc-156-p1
7.26 CDCl3
6.86
6.85
6.84
6.83
6.83
6.83
6.81
6.81
6.77
6.76
6.75
6.73
6.70
6.69
6.67
6.67
5.30
4.29
4.28
4.26
4.24
1.48
1.46
1.00
1.00
0.96
1.01
3.03
f1 (ppm)

2g

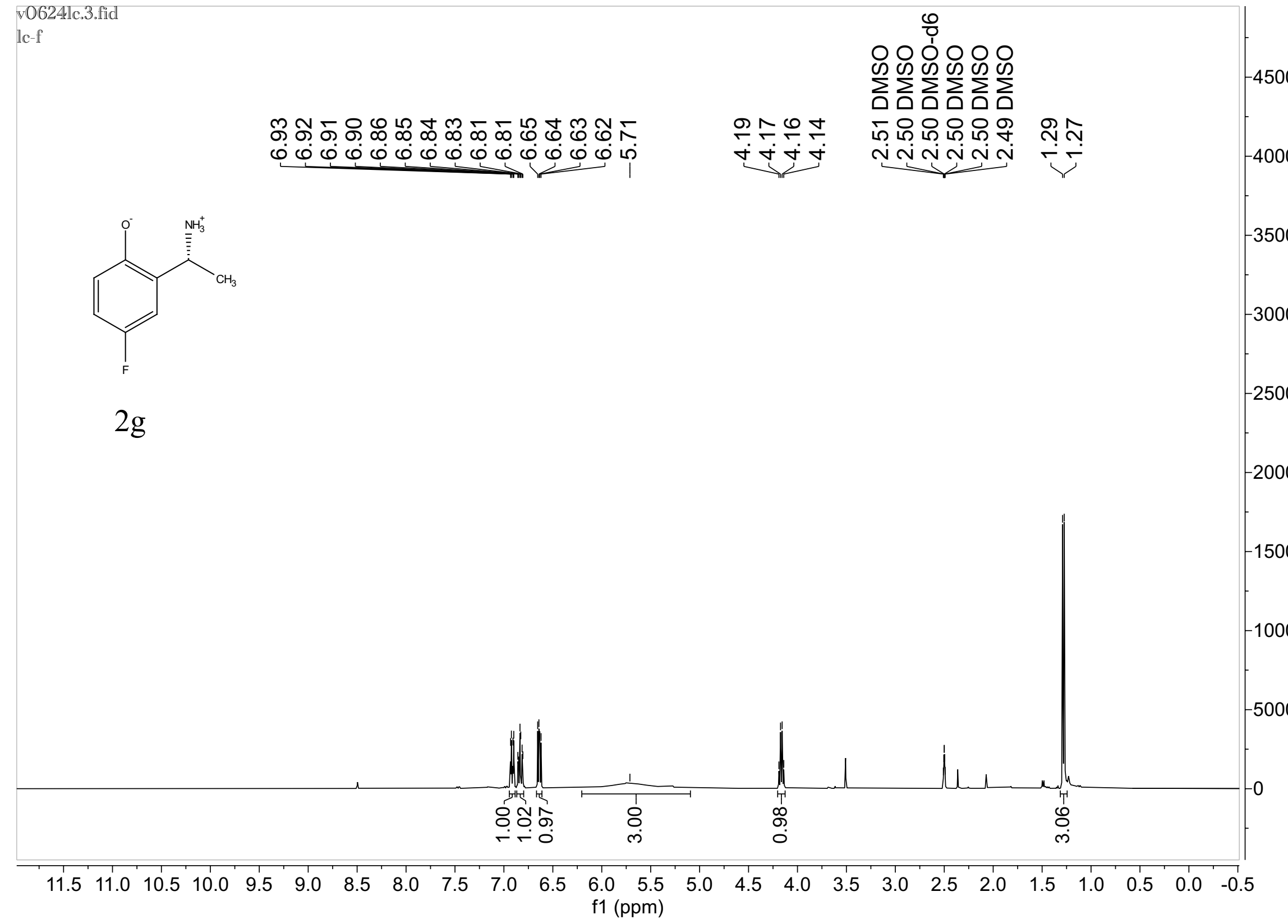
v0624lc.3.fid
lc-f
6.93
6.92
6.91
6.90
6.86
6.85
6.84
6.83
6.81
6.81
6.65
6.64
6.63
6.62
5.71
4.19
4.17
4.16
4.14
2.51 DMSO
2.50 DMSO
2.50 DMSO-d6
2.50 DMSO
2.50 DMSO
2.49 DMSO
1.29
1.27
1.00
1.02
0.97
3.00
0.98
3.06
f1 (ppm)

2g

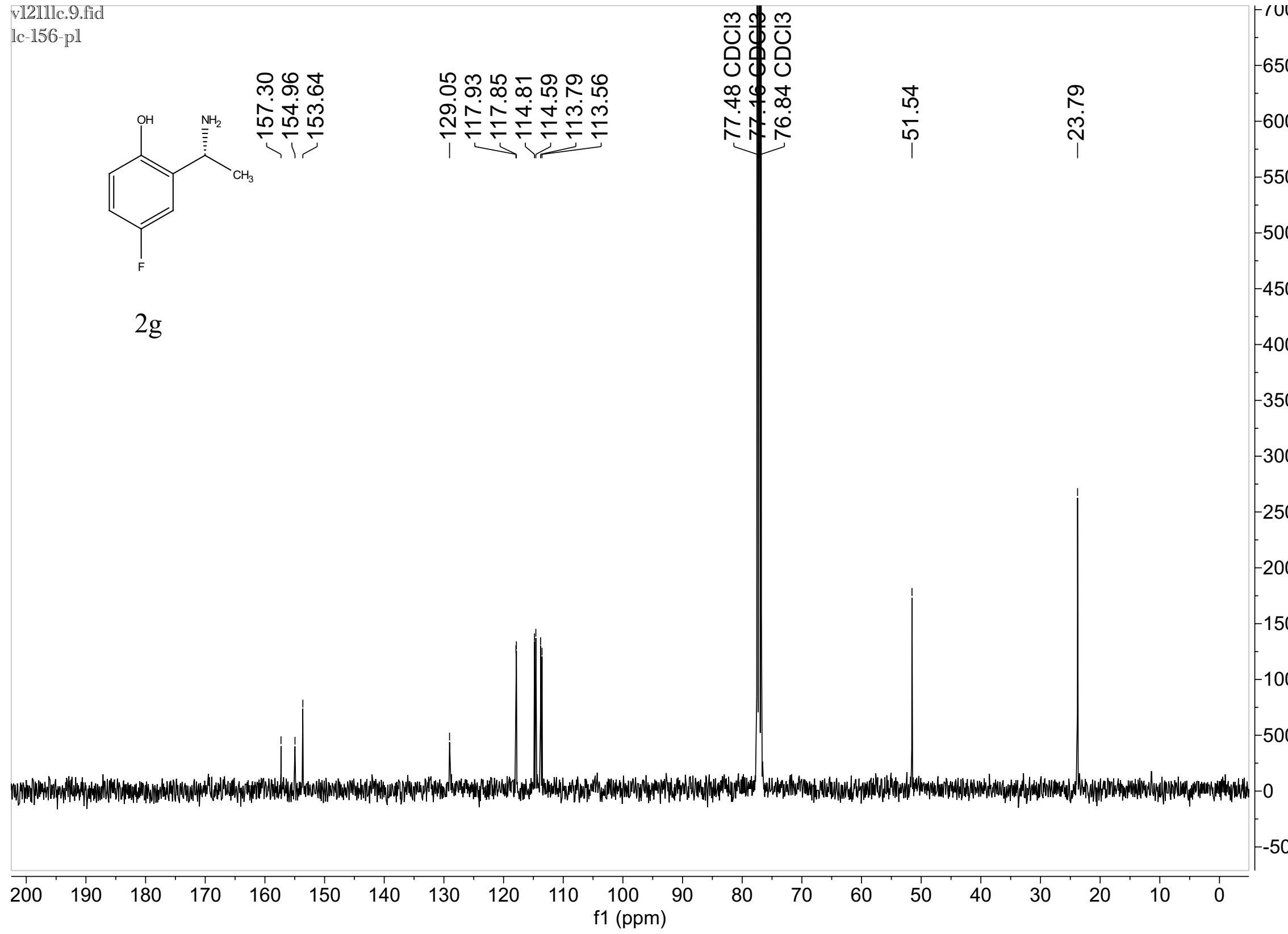

v1211lc.9.fid
lc-156-p1
2g
157.30
154.96
153.64
129.05
117.93
117.85
114.81
114.59
113.79
113.56
77.48 CDCl3
77.16 CDCl3
76.84 CDCl3
51.54
23.79
f1 (ppm)

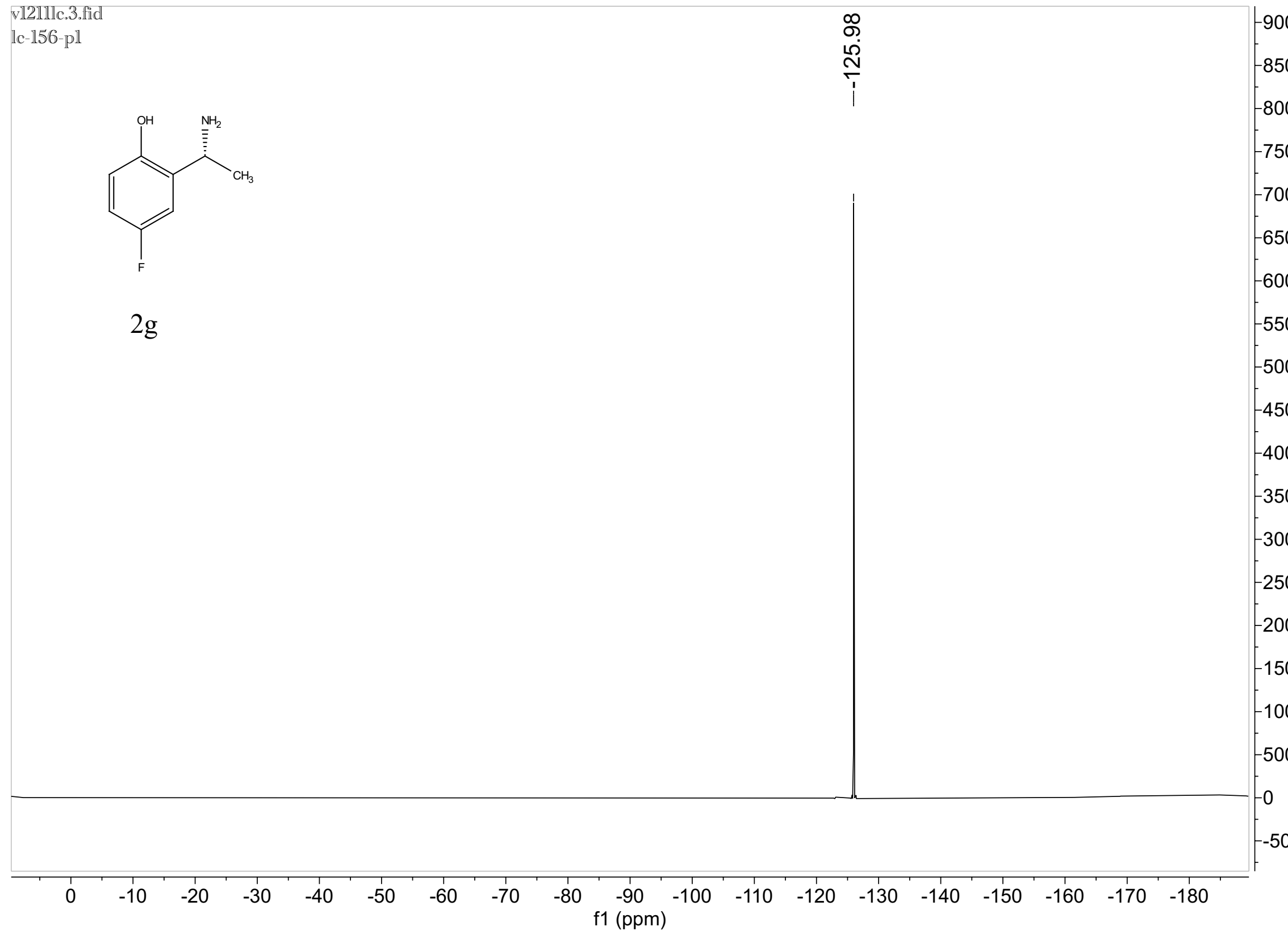

v1211lc.3.fid
lc-156-p1
2g
-125.98
f1 (ppm)

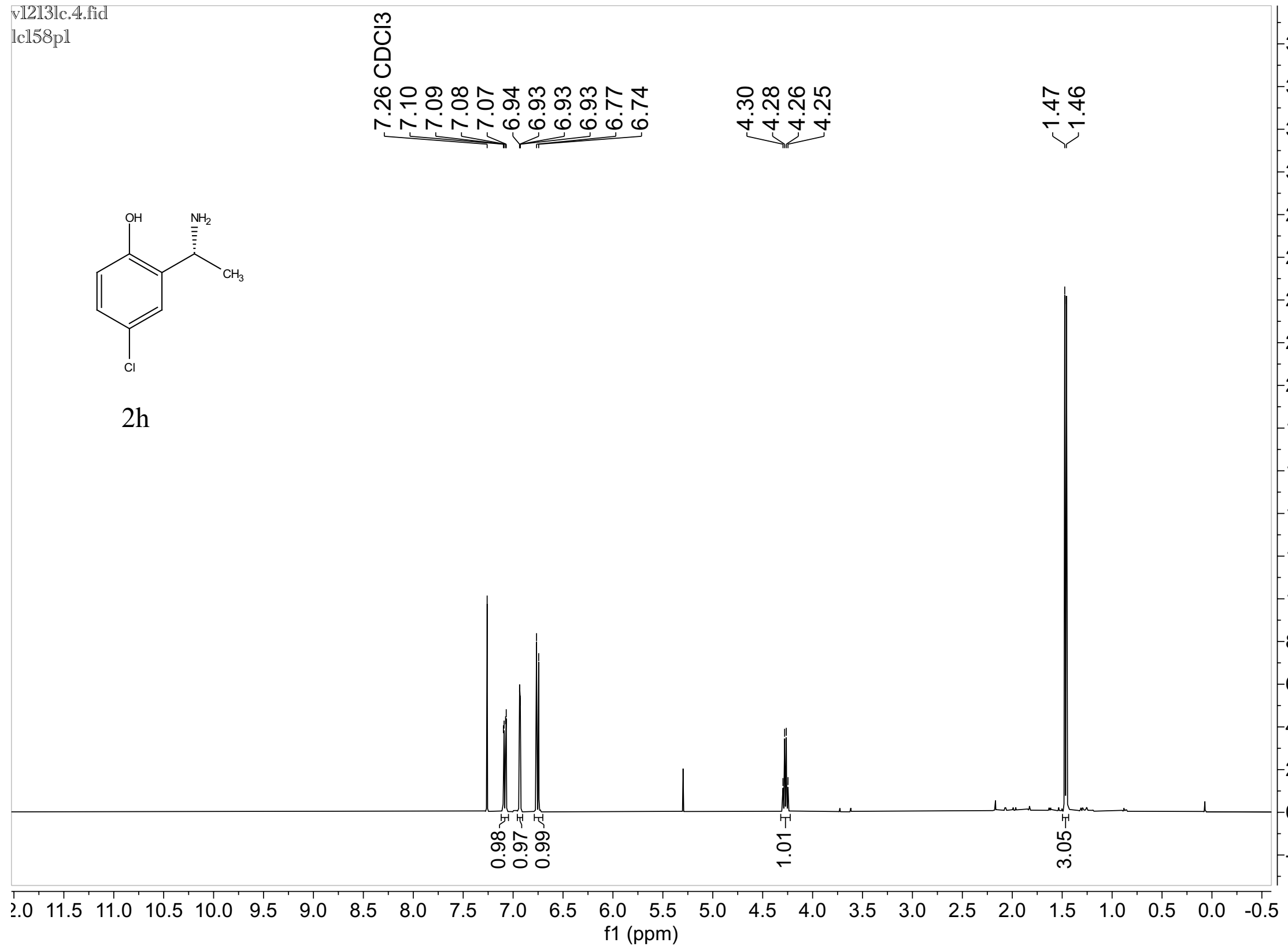
vl213lc.4.fid
lc158p1
7.26 CDCl3
7.10
7.09
7.08
7.07
6.94
6.93
6.93
6.93
6.77
6.74
4.30
4.28
4.26
4.25
1.47
1.46
OH
NH2
CH3
Cl
2h
0.98
0.97
0.99
1.01
3.05
f1 (ppm)

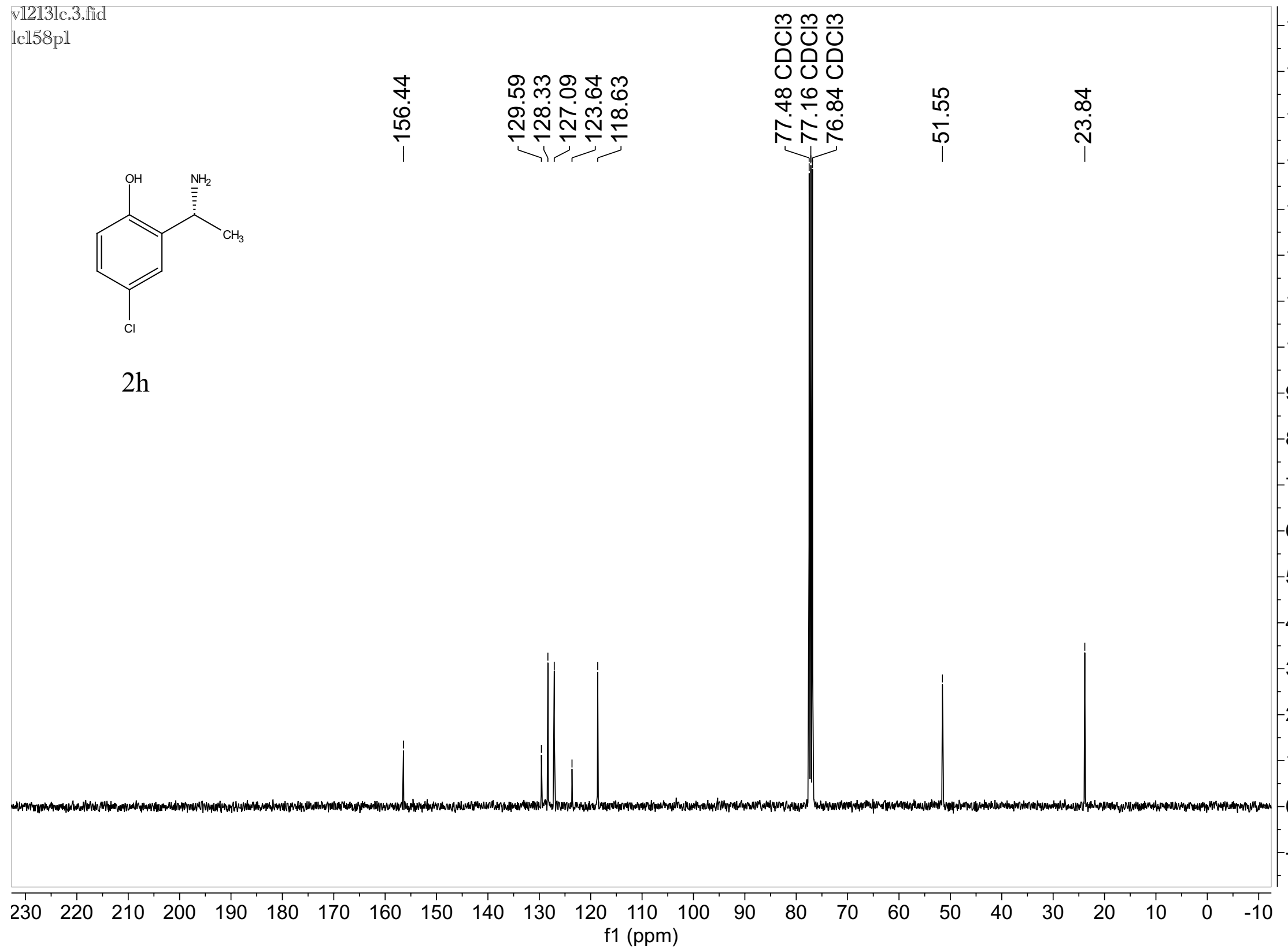
vl213lc.3.fid
lc158p1
156.44
129.59
128.33
127.09
123.64
118.63
77.48 CDCl3
77.16 CDCl3
76.84 CDCl3
51.55
23.84
OH
NH2
CH3
Cl
2h
f1 (ppm)

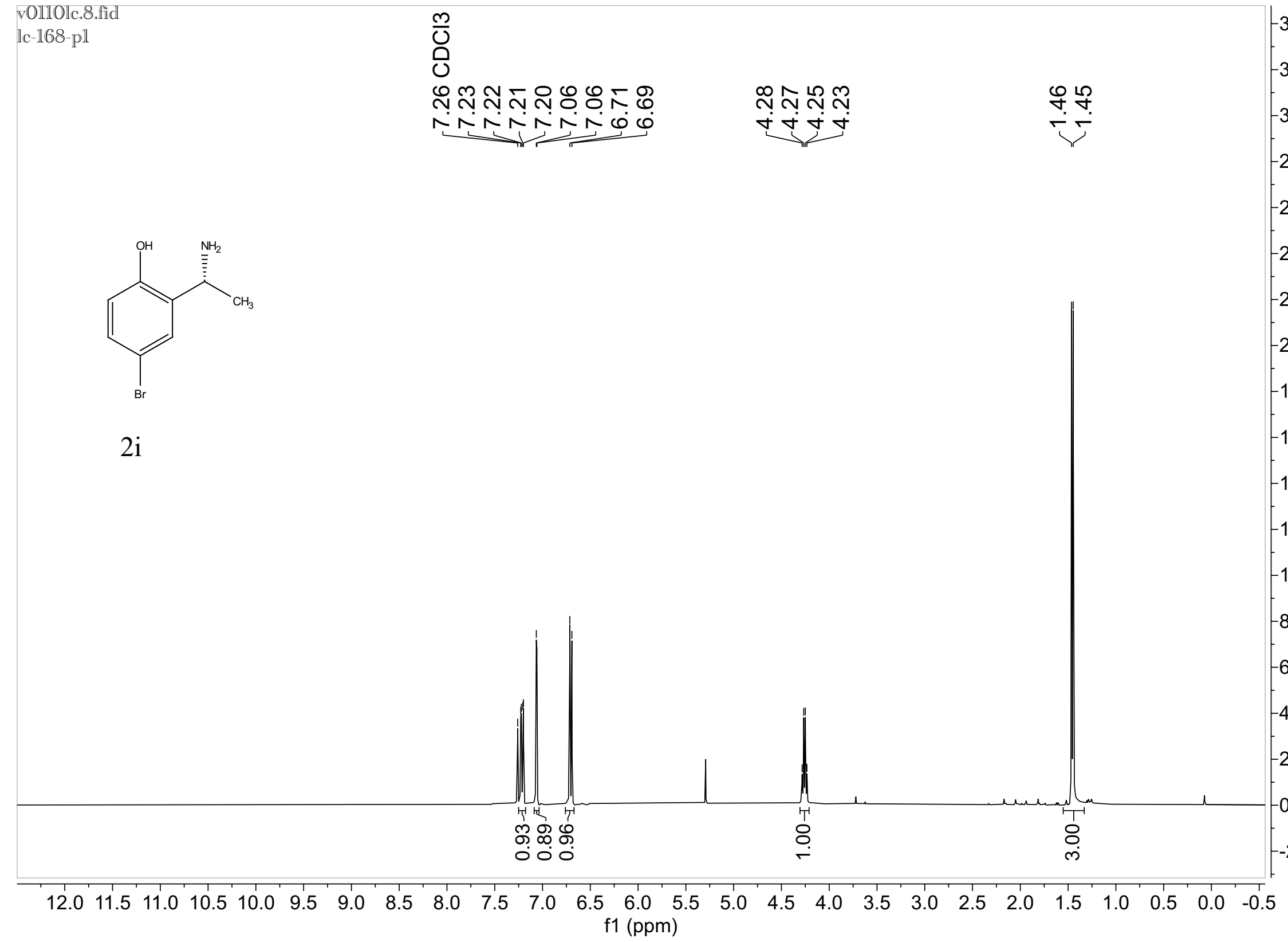
v0110lc.8.fid
lc-168-p1
7.26 CDCl3
7.23
7.22
7.21
7.20
7.06
7.06
6.71
6.69
4.28
4.27
4.25
4.23
1.46
1.45
OH
NH2
CH3
Br
0.93
0.89
0.96
1.00
3.00
f1 (ppm)

2i

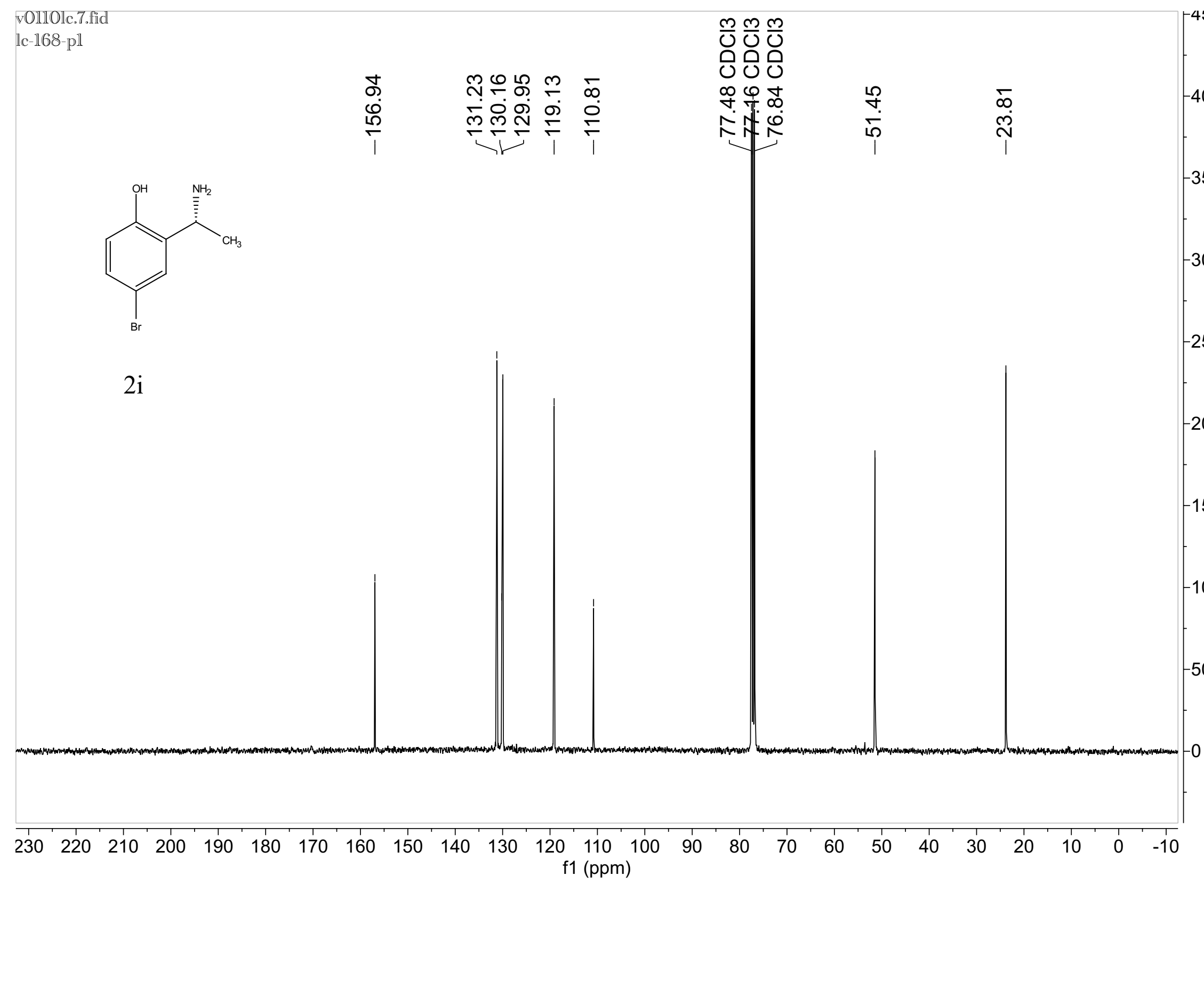
v0110lc.7.fid
lc-168-p1
156.94
131.23
130.16
129.95
119.13
110.81
77.48 CDCl3
77.16 CDCl3
76.84 CDCl3
51.45
23.81
OH
NH2
CH3
Br
f1 (ppm)

2i

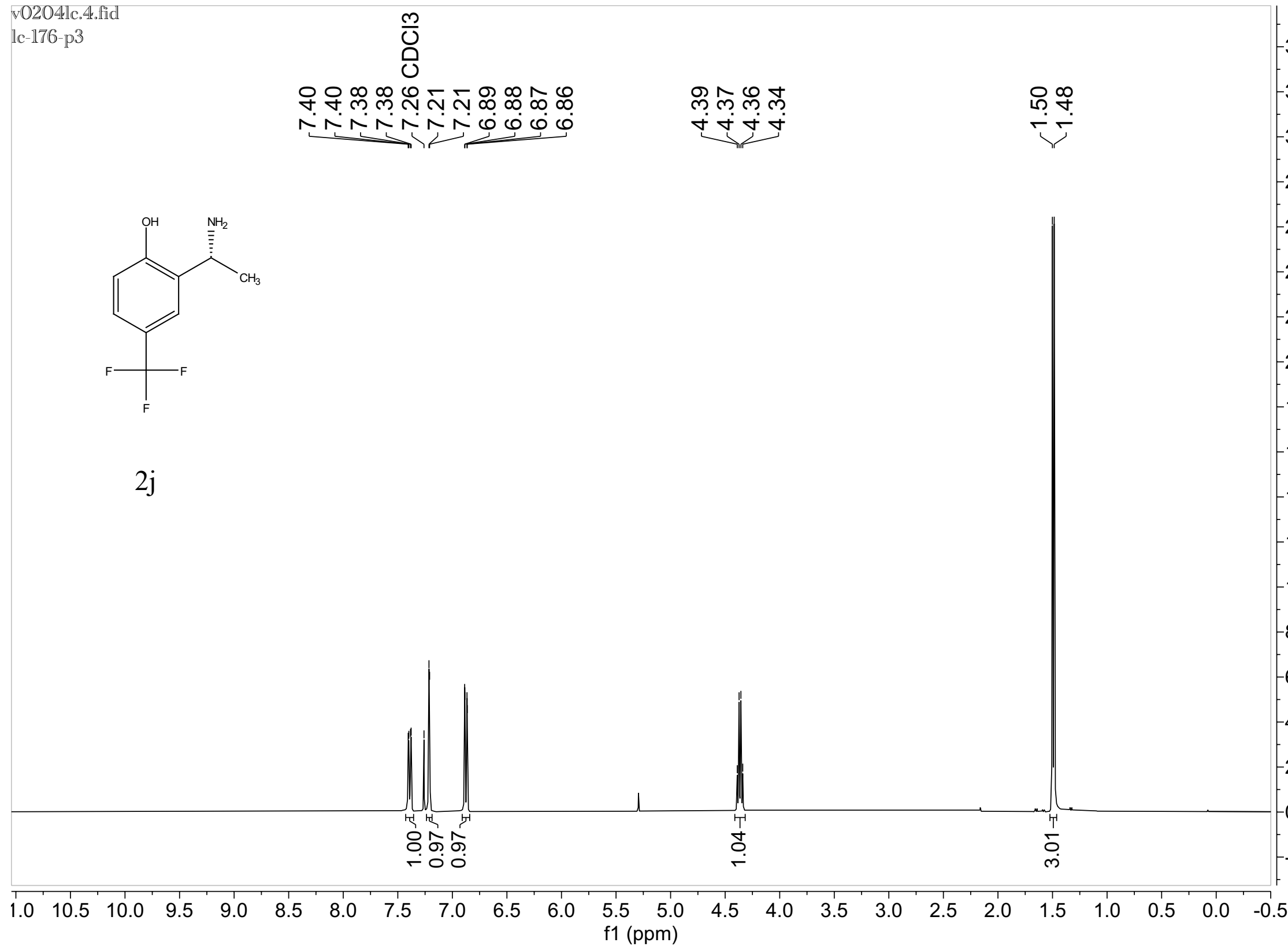

v0204lc.4.fid
lc-176-p3
7.40
7.40
7.38
7.38
7.26 CDCl3
7.21
7.21
6.89
6.88
6.87
6.86
4.39
4.37
4.36
4.34
1.50
1.48
OH
NH2
CH3
F
F
F
2j
1.00
0.97
0.97
1.04
3.01
f1 (ppm)

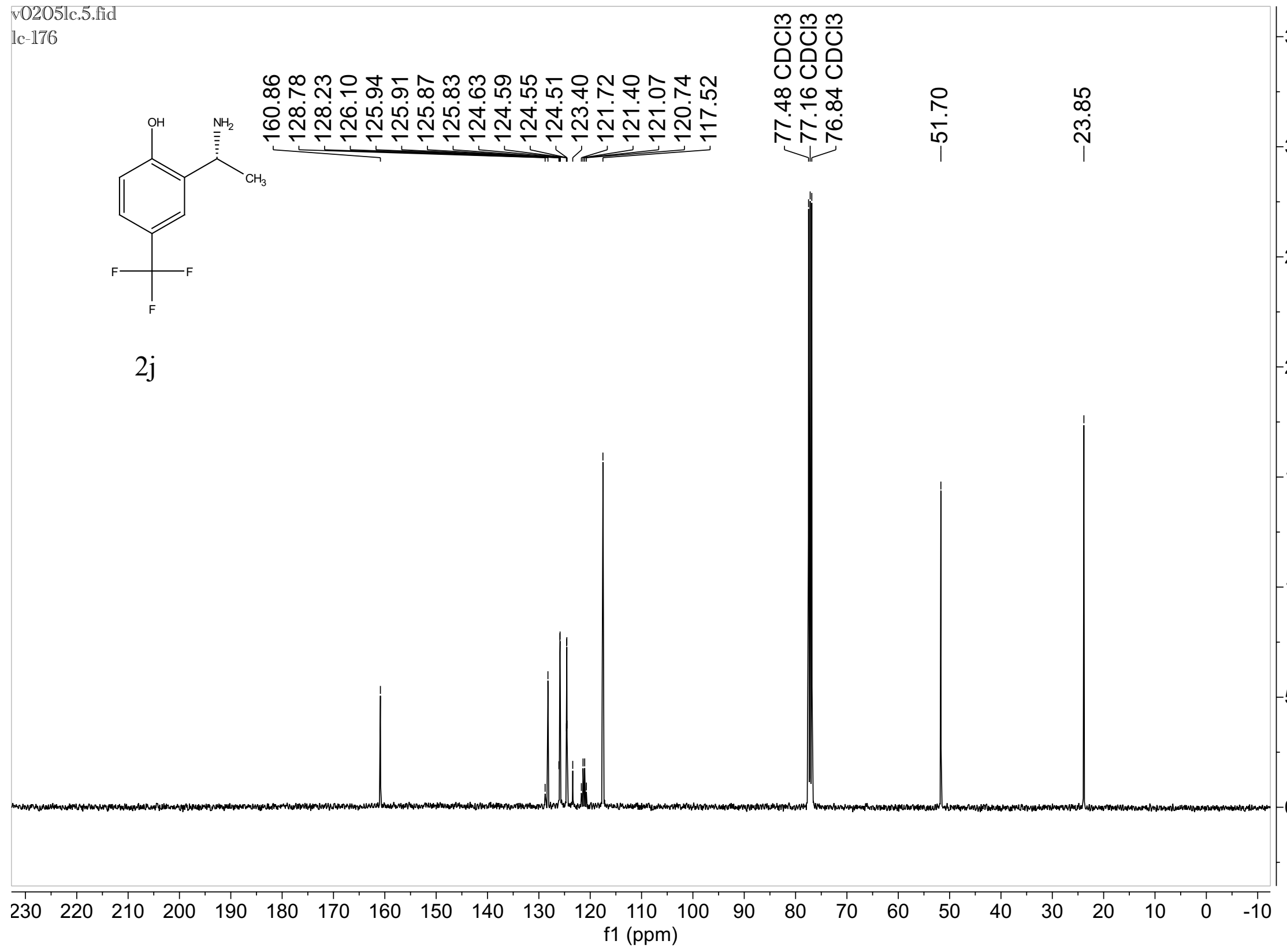

v0205lc.5.fid
lc-176
160.86
128.78
128.23
126.10
125.94
125.91
125.87
125.83
124.63
124.59
124.55
124.51
123.40
121.72
121.40
121.07
120.74
117.52
77.48 CDCl3
77.16 CDCl3
76.84 CDCl3
51.70
23.85
OH
NH2
CH3
F
F
F
2j
f1 (ppm)

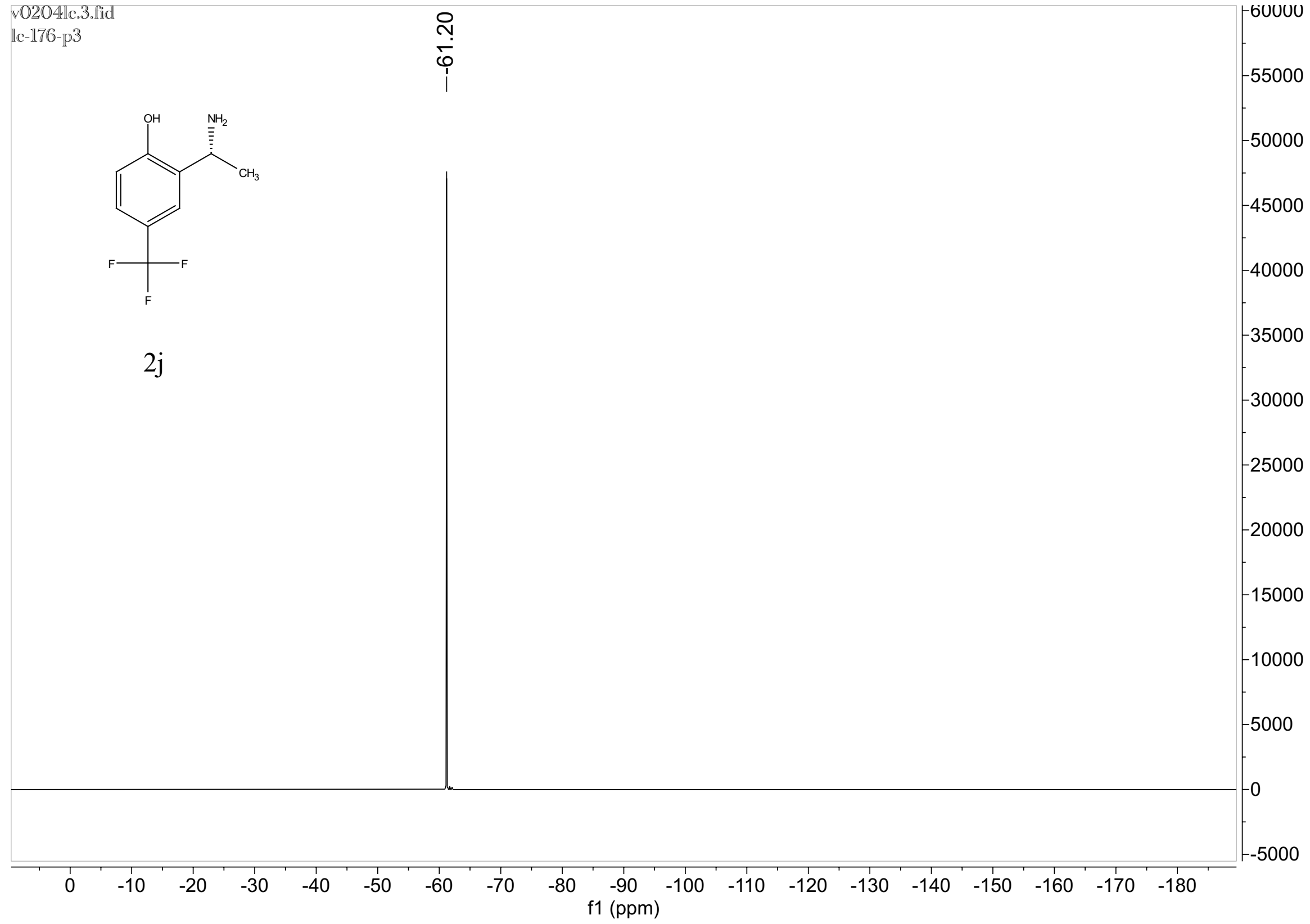
v0204lc.3.fid
lc-176-p3
--61.20
OH
NH2
CH3
F
F
F
2j
60000
55000
50000
45000
40000
35000
30000
25000
20000
15000
10000
5000
0
-5000
0 -10 -20 -30 -40 -50 -60 -70 -80 -90 -100 -110 -120 -130 -140 -150 -160 -170 -180
f1 (ppm)

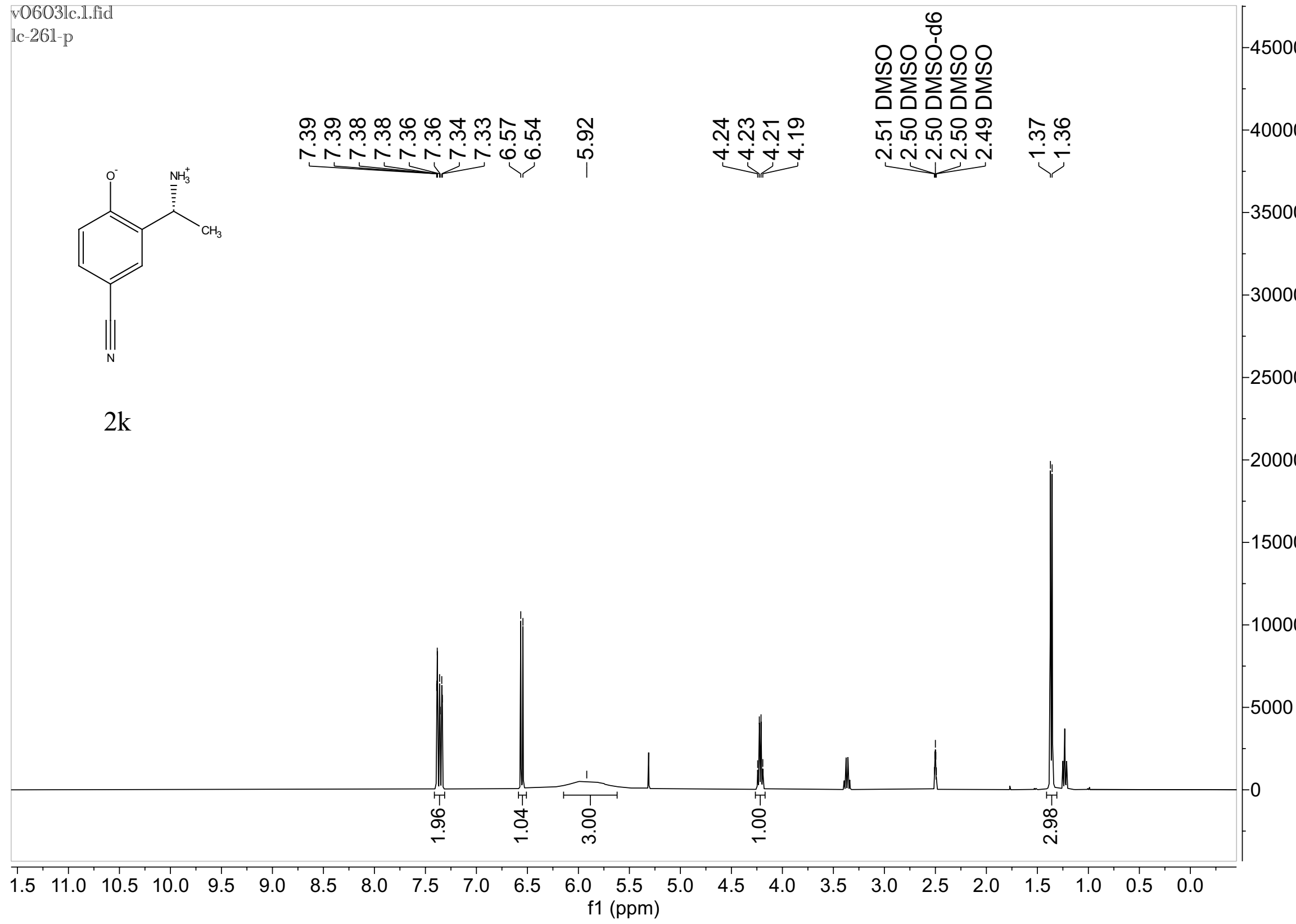
v0603lc.1.fid
lc-261-p
7.39 7.39 7.38 7.38 7.36 7.36 7.34 7.33 6.57 6.54
5.92
4.24 4.23 4.21 4.19
2.51 DMSO 2.50 DMSO 2.50 DMSO-d6 2.50 DMSO 2.49 DMSO
1.37 1.36
2k
1.96 1.04 3.00 1.00 2.98
f1 (ppm)

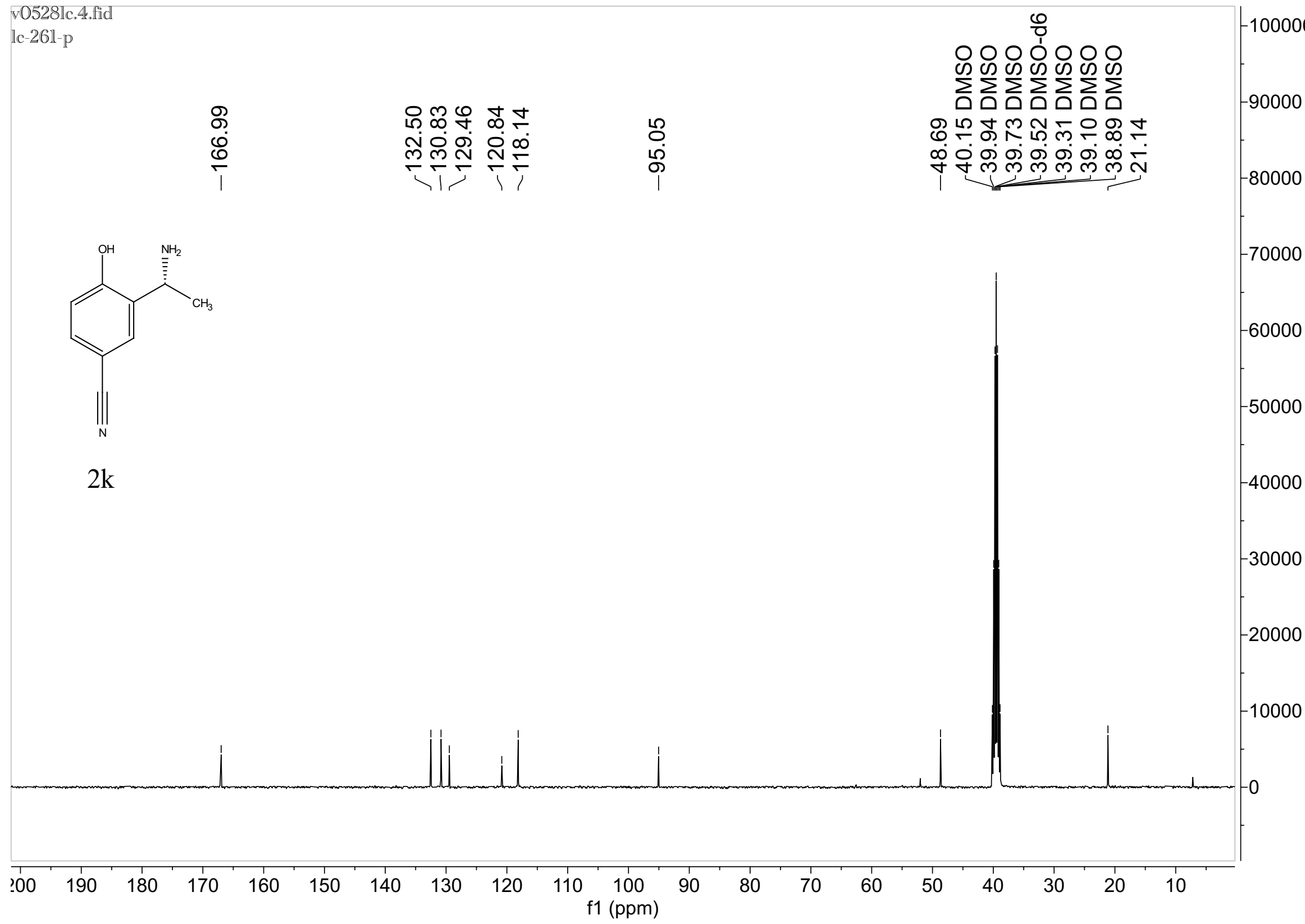
v0528lc.4.fid
lc-261-p
166.99
132.50 130.83 129.46 120.84 118.14
95.05
48.69 40.15 DMSO 39.94 DMSO 39.73 DMSO 39.52 DMSO-d6 39.31 DMSO 39.10 DMSO 38.89 DMSO 21.14
2k
f1 (ppm)

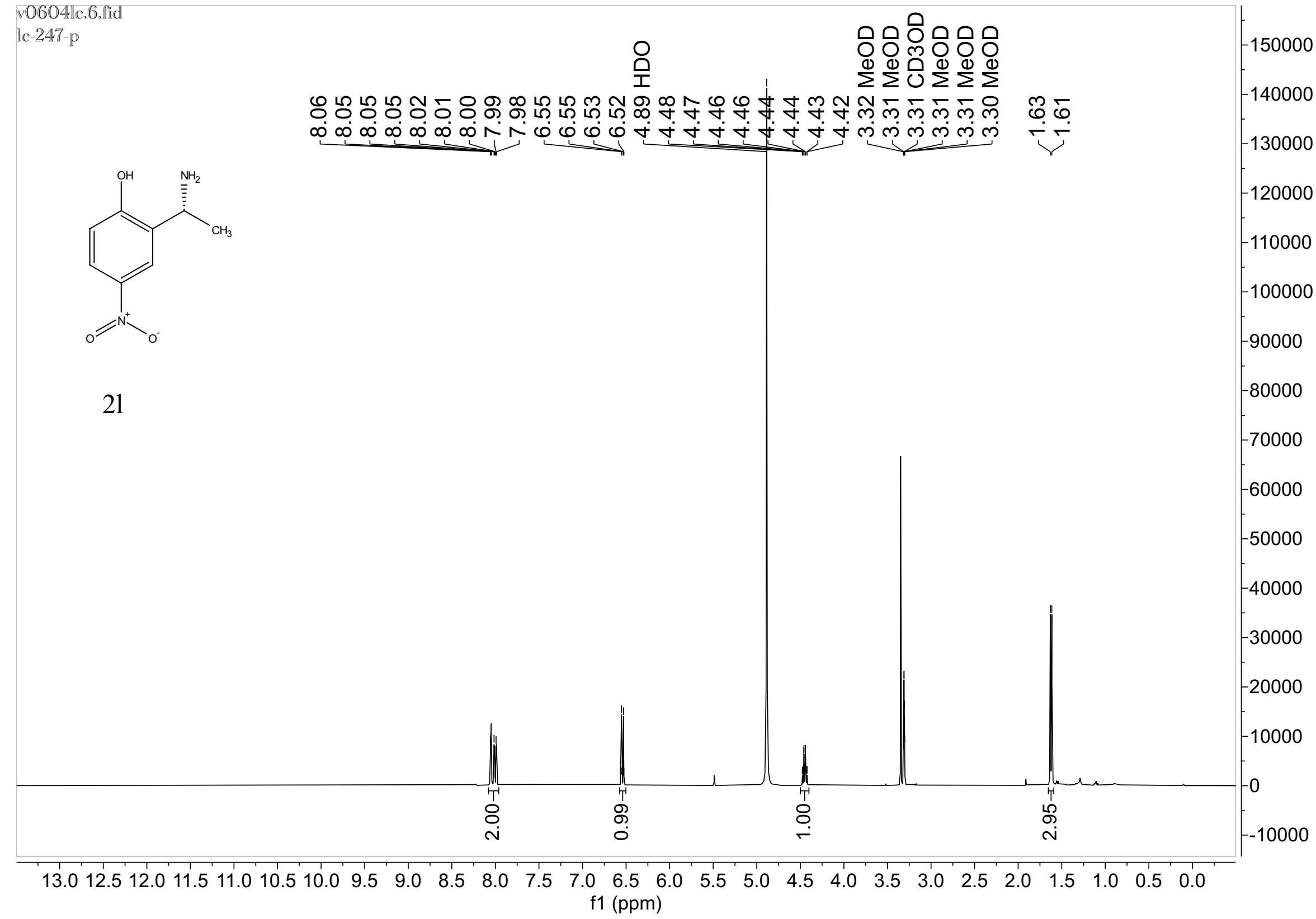
v0604lc.6.fid
lc-247-p
8.06
8.05
8.05
8.05
8.02
8.01
8.00
7.99
7.98
6.55
6.55
6.53
6.52
4.89 HDO
4.48
4.47
4.46
4.46
4.44
4.44
4.43
4.42
3.32 MeOD
3.31 MeOD
3.31 CD3OD
3.31 MeOD
3.31 MeOD
3.30 MeOD
1.63
1.61
OH
NH2
CH3
21
2.00
0.99
1.00
2.95
f1 (ppm)

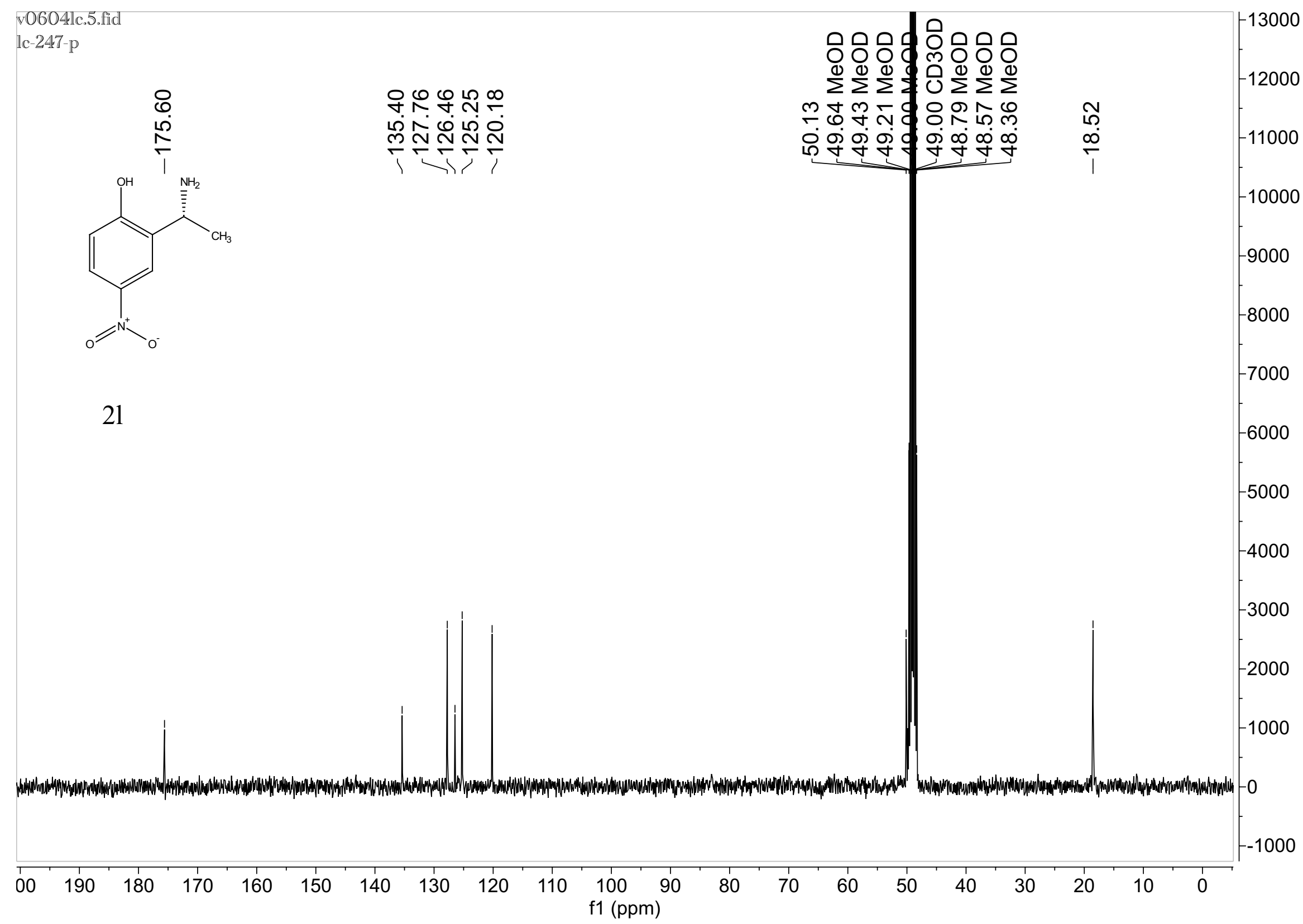
v0604lc.5.fid
lc-247-p
175.60
135.40
127.76
126.46
125.25
120.18
50.13
49.64 MeOD
49.43 MeOD
49.21 MeOD
49.00 MeOD
49.00 CD3OD
48.79 MeOD
48.57 MeOD
48.36 MeOD
18.52
OH
NH2
CH3
21
f1 (ppm)

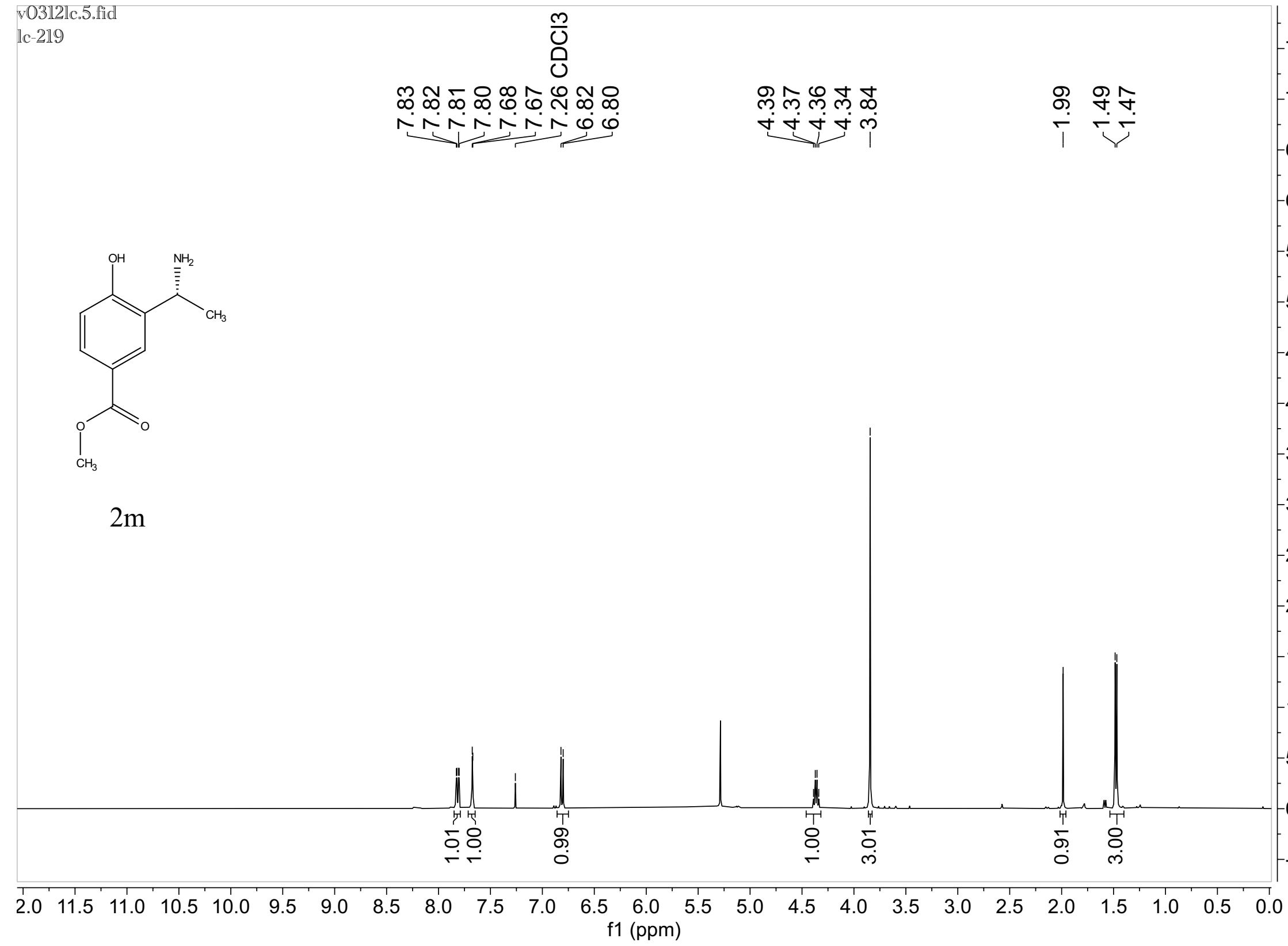
v0312lc.5.fid
lc-219
7.83
7.82
7.81
7.80
7.68
7.67
7.26 CDCl3
6.82
6.80
4.39
4.37
4.36
4.34
3.84
1.99
1.49
1.47
OH
NH2
CH3
O
O
CH3
2m
1.01
1.00
0.99
1.00
3.01
0.91
3.00
f1 (ppm)

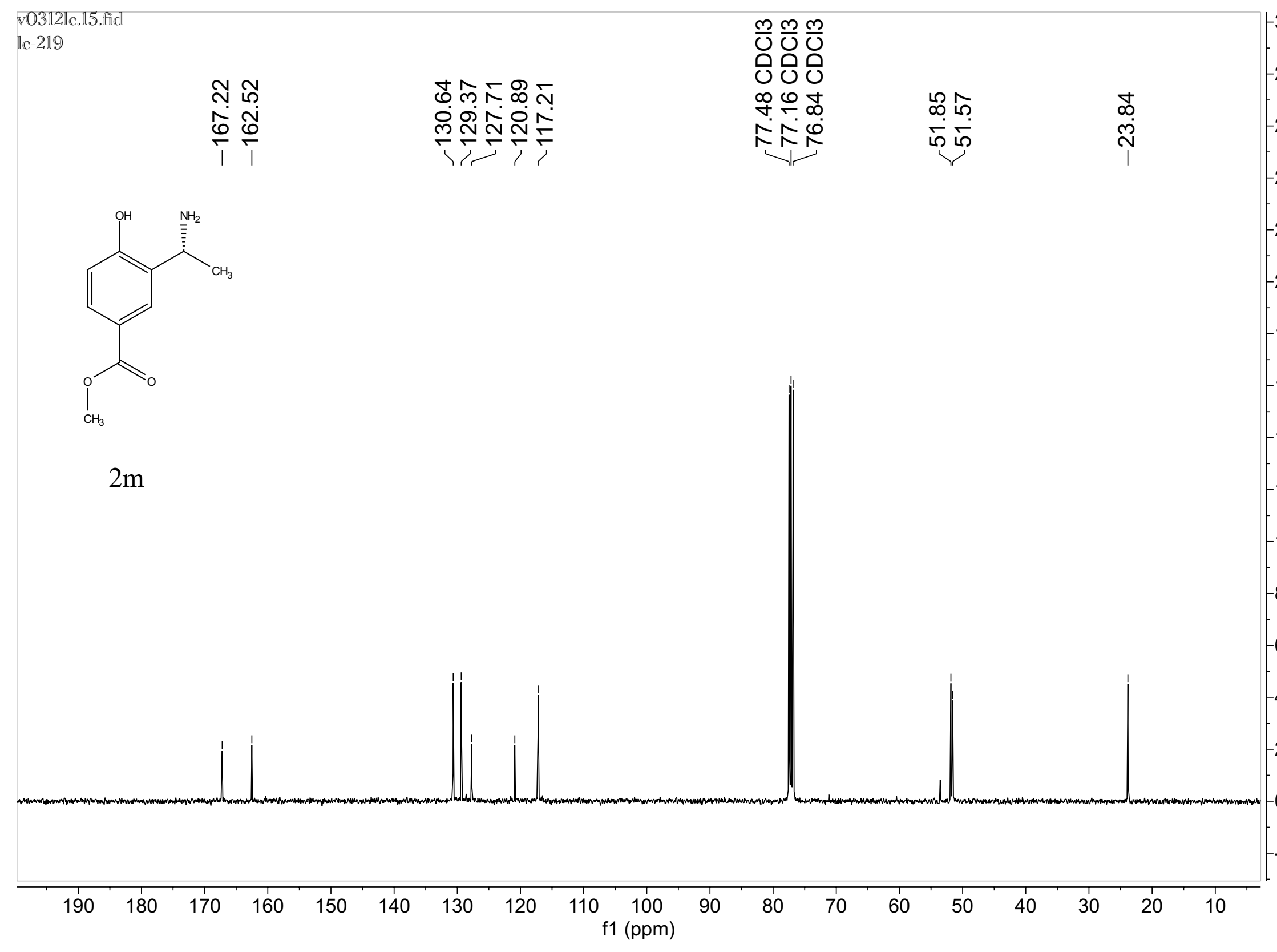
v0312lc.15.fid
lc-219
167.22
162.52
130.64
129.37
127.71
120.89
117.21
77.48 CDCl3
77.16 CDCl3
76.84 CDCl3
51.85
51.57
23.84
OH
NH2
CH3
O
O
CH3
2m
f1 (ppm)

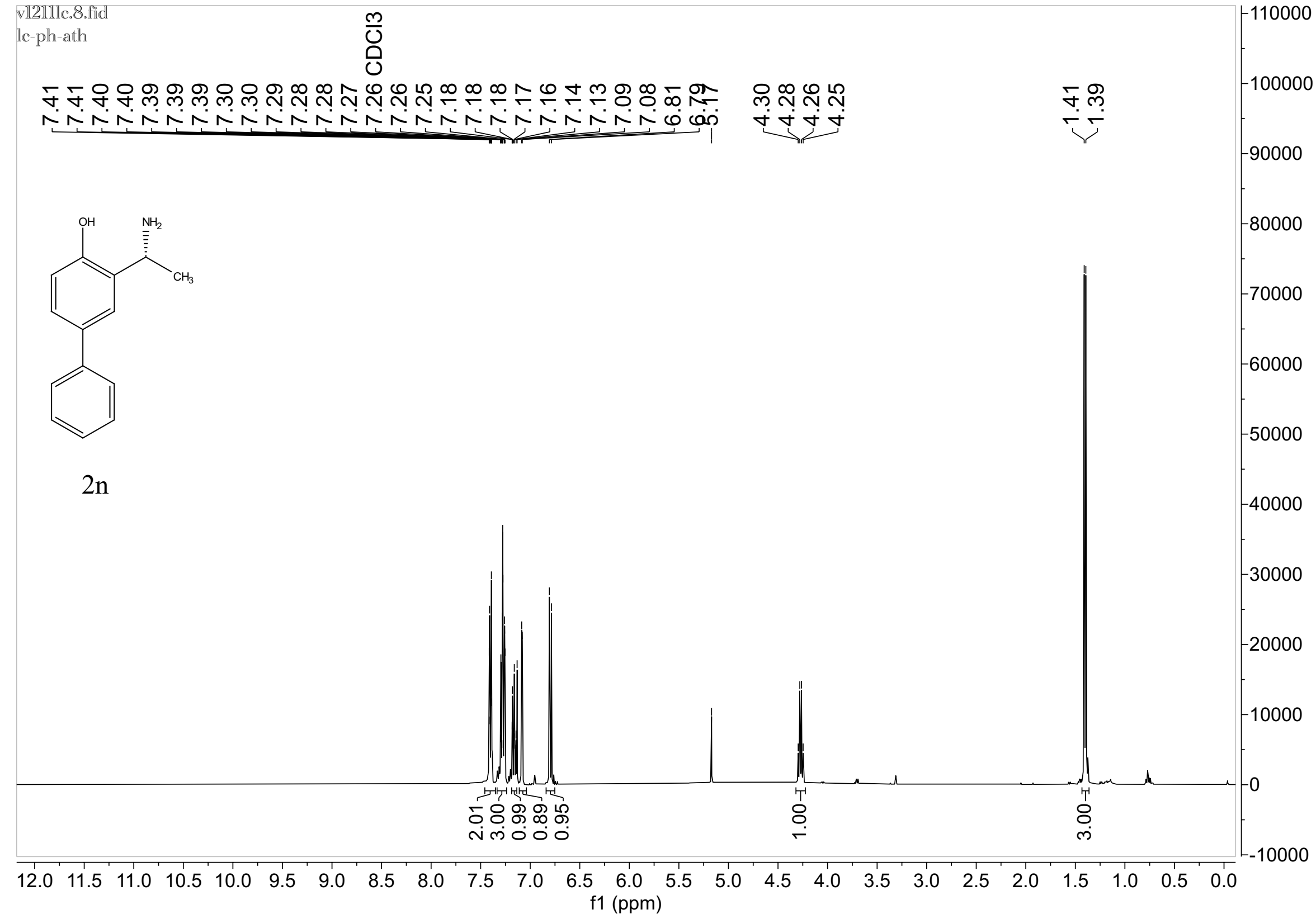

vl211lc.8.fid
lc-ph-ath
CDCl3
7.41
7.41
7.40
7.40
7.39
7.39
7.39
7.30
7.30
7.29
7.28
7.28
7.27
7.26
7.26
7.25
7.18
7.18
7.17
7.16
7.14
7.13
7.09
7.08
6.81
6.79
5.17
4.30
4.28
4.26
4.25
1.41
1.39
OH
NH2
CH3
2n
2.01
3.00
0.99
0.89
0.95
1.00
3.00
f1 (ppm)

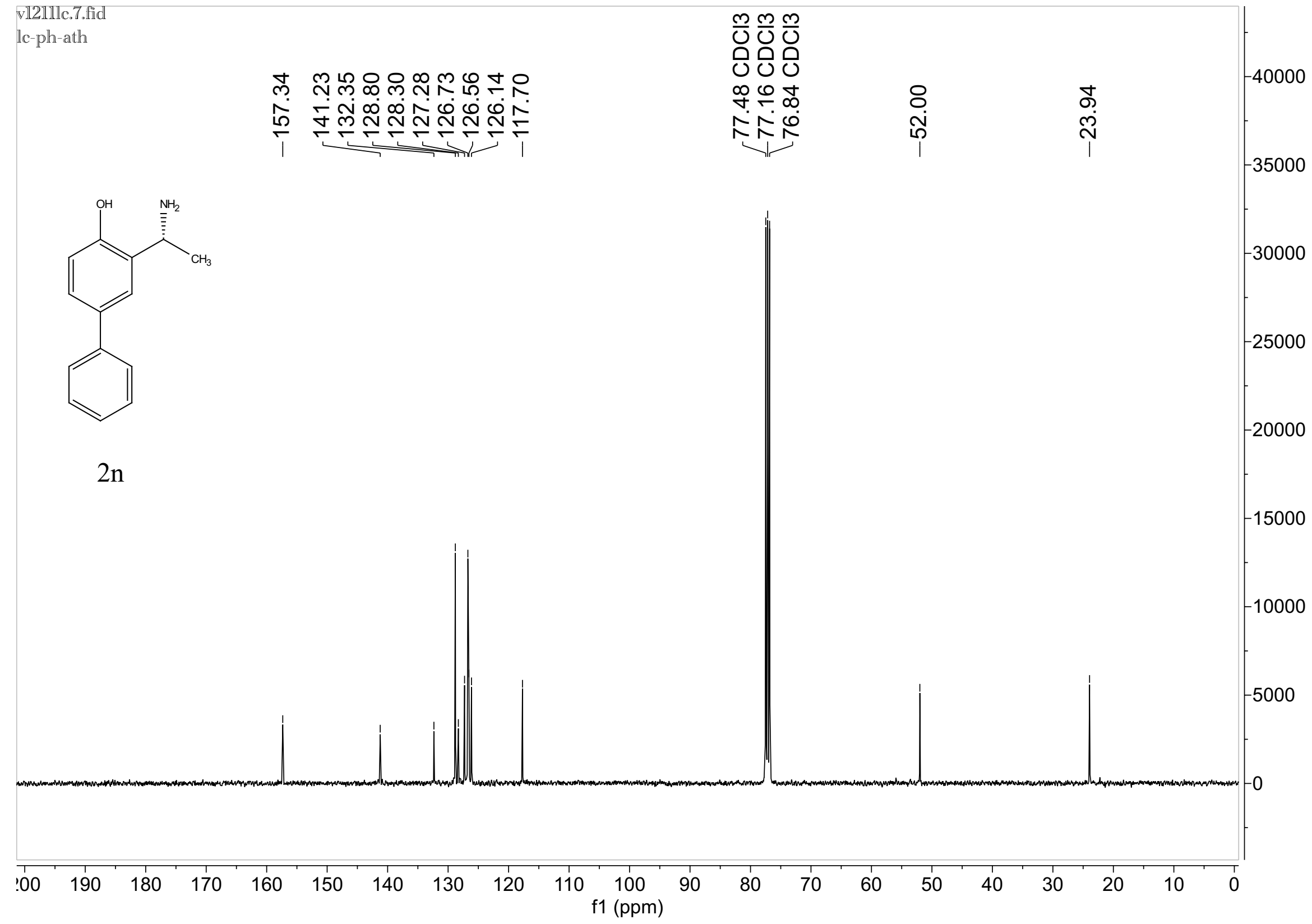

vl211lc.7.fid
lc-ph-ath
157.34
141.23
132.35
128.80
128.30
127.28
126.73
126.56
126.14
117.70
77.48 CDCl3
77.16 CDCl3
76.84 CDCl3
52.00
23.94
OH
NH2
CH3
2n
f1 (ppm)

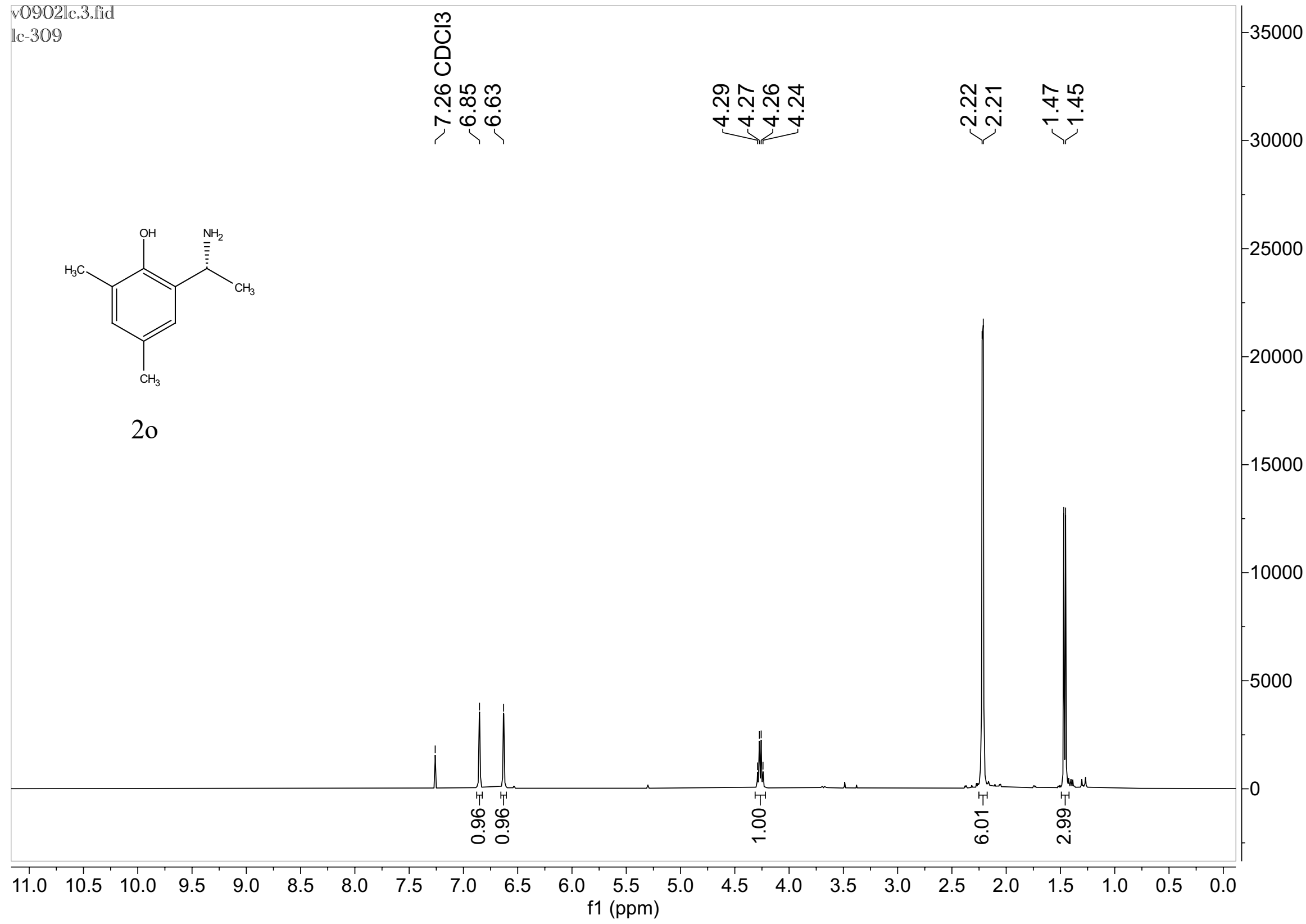
v0902lc.3.fid
lc-309
7.26 CDCl3
6.85
6.63
4.29
4.27
4.26
4.24
2.22
2.21
1.47
1.45
OH
NH2
H3C
CH3
CH3
2o
0.96
0.96
1.00
6.01
2.99
f1 (ppm)

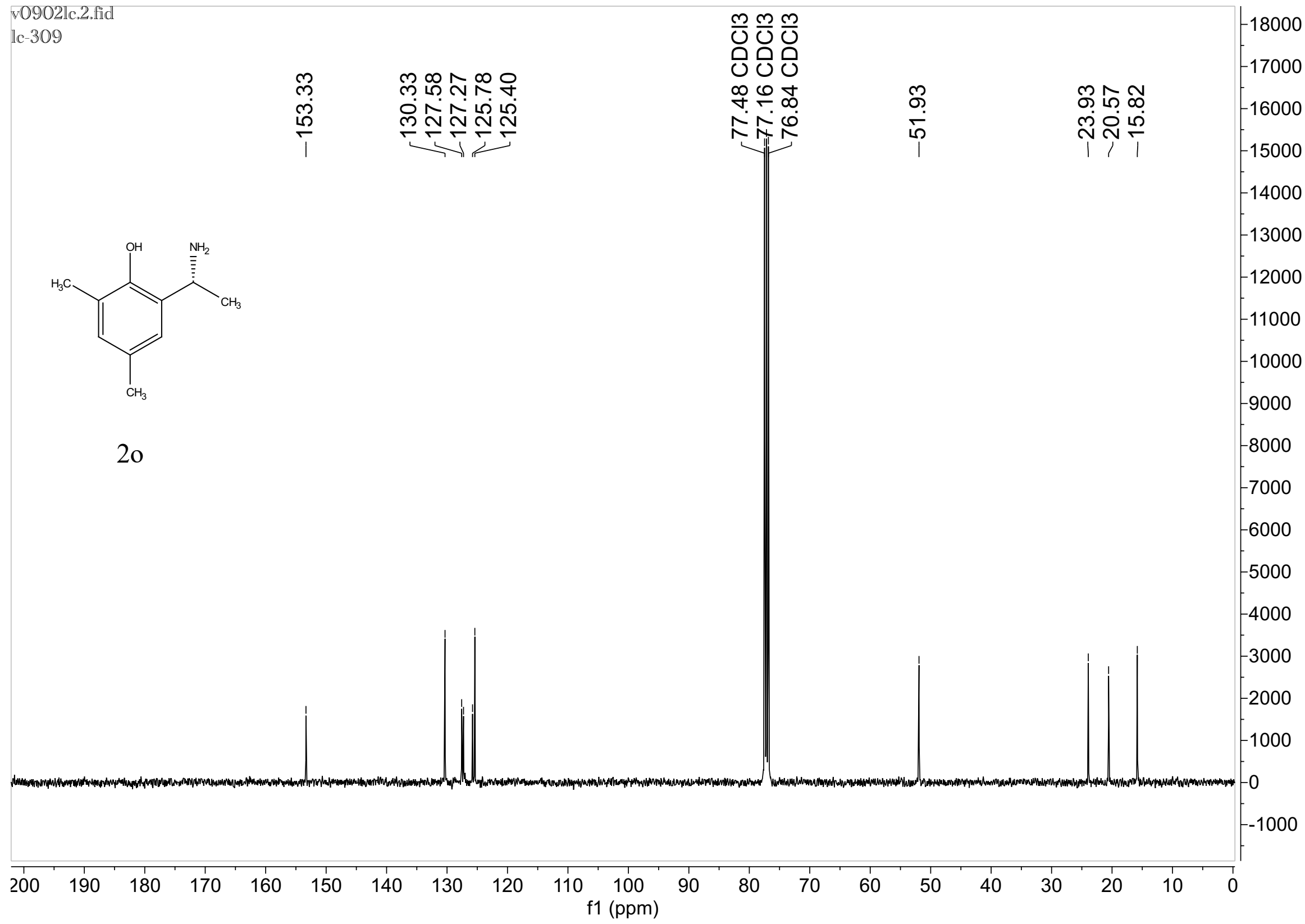
v0902lc.2.fid
lc-309
153.33
130.33
127.58
127.27
125.78
125.40
77.48 CDCl3
77.16 CDCl3
76.84 CDCl3
51.93
23.93
20.57
15.82
OH
NH2
H3C
CH3
CH3
2o
f1 (ppm)

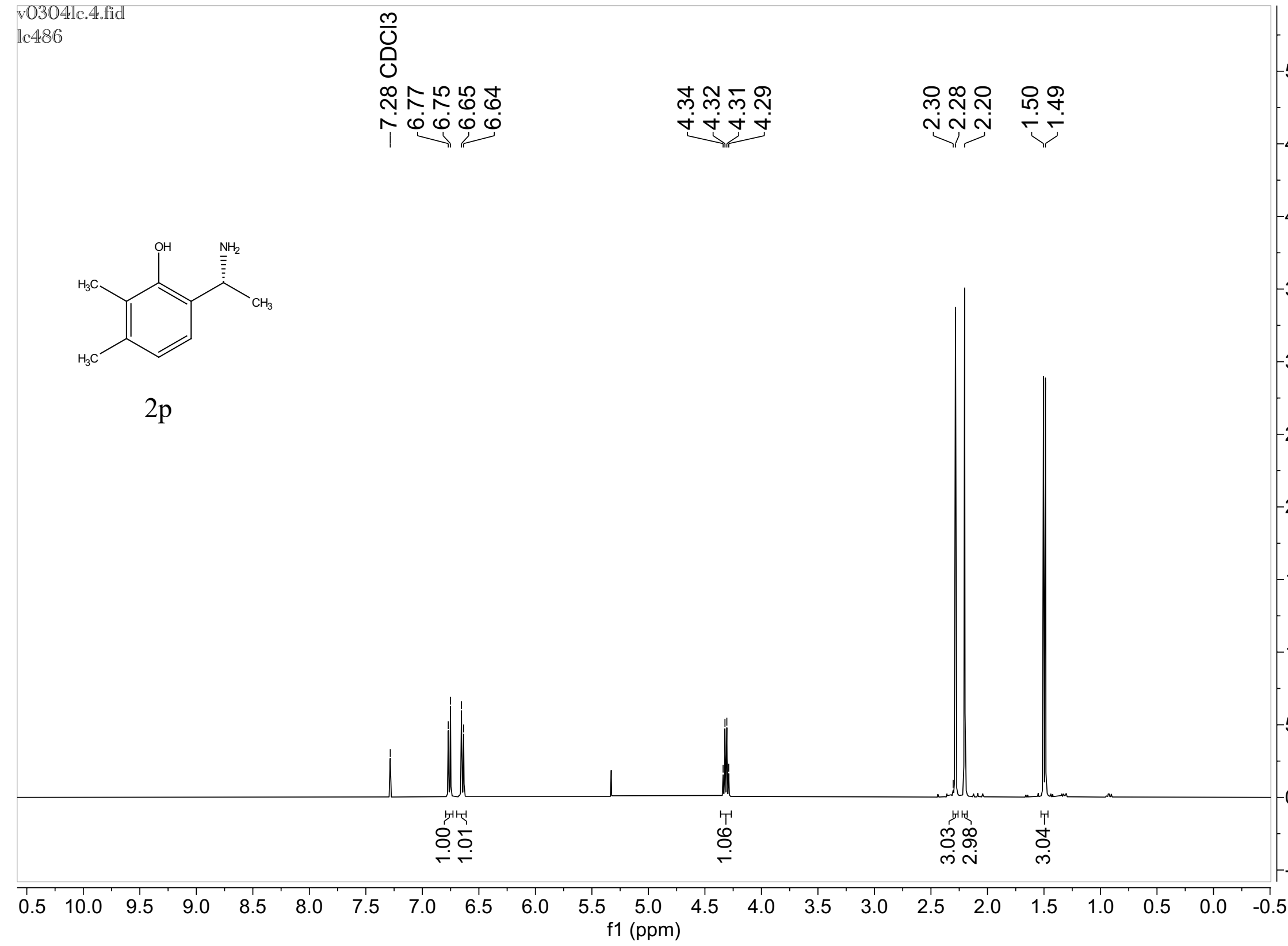

v0304lc.4.fid
lc486
7.28 CDCl3
6.77
6.75
6.65
6.64
4.34
4.32
4.31
4.29
2.30
2.28
2.20
1.50
1.49
OH
NH2
H3C
CH3
H3C
2p
1.00
1.01
1.06
3.03
2.98
3.04
f1 (ppm)

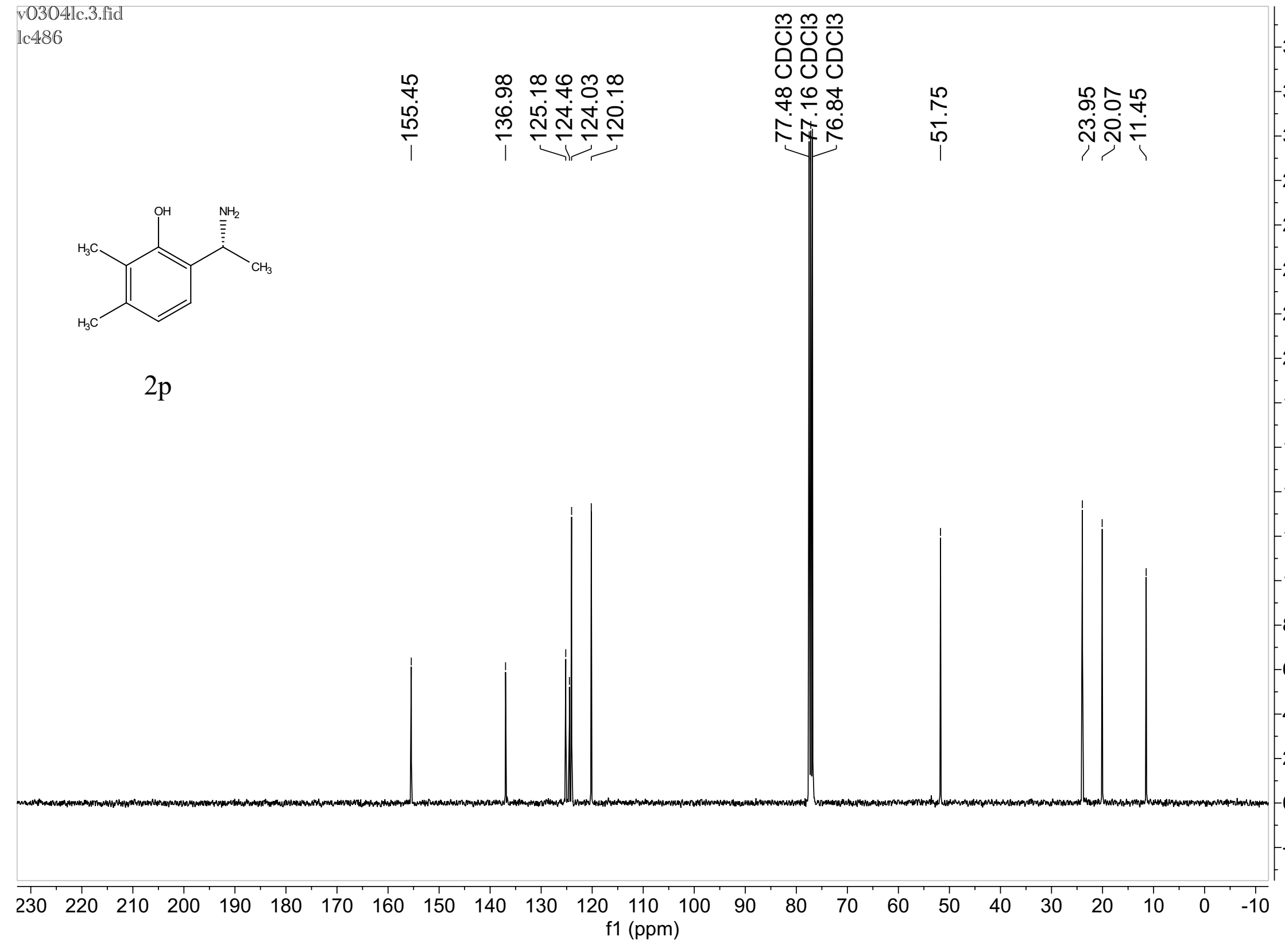

v0304lc.3.fid
lc486
155.45
136.98
125.18
124.46
124.03
120.18
77.48 CDCl3
77.16 CDCl3
76.84 CDCl3
51.75
23.95
20.07
11.45
OH
NH2
H3C
CH3
H3C
2p
f1 (ppm)

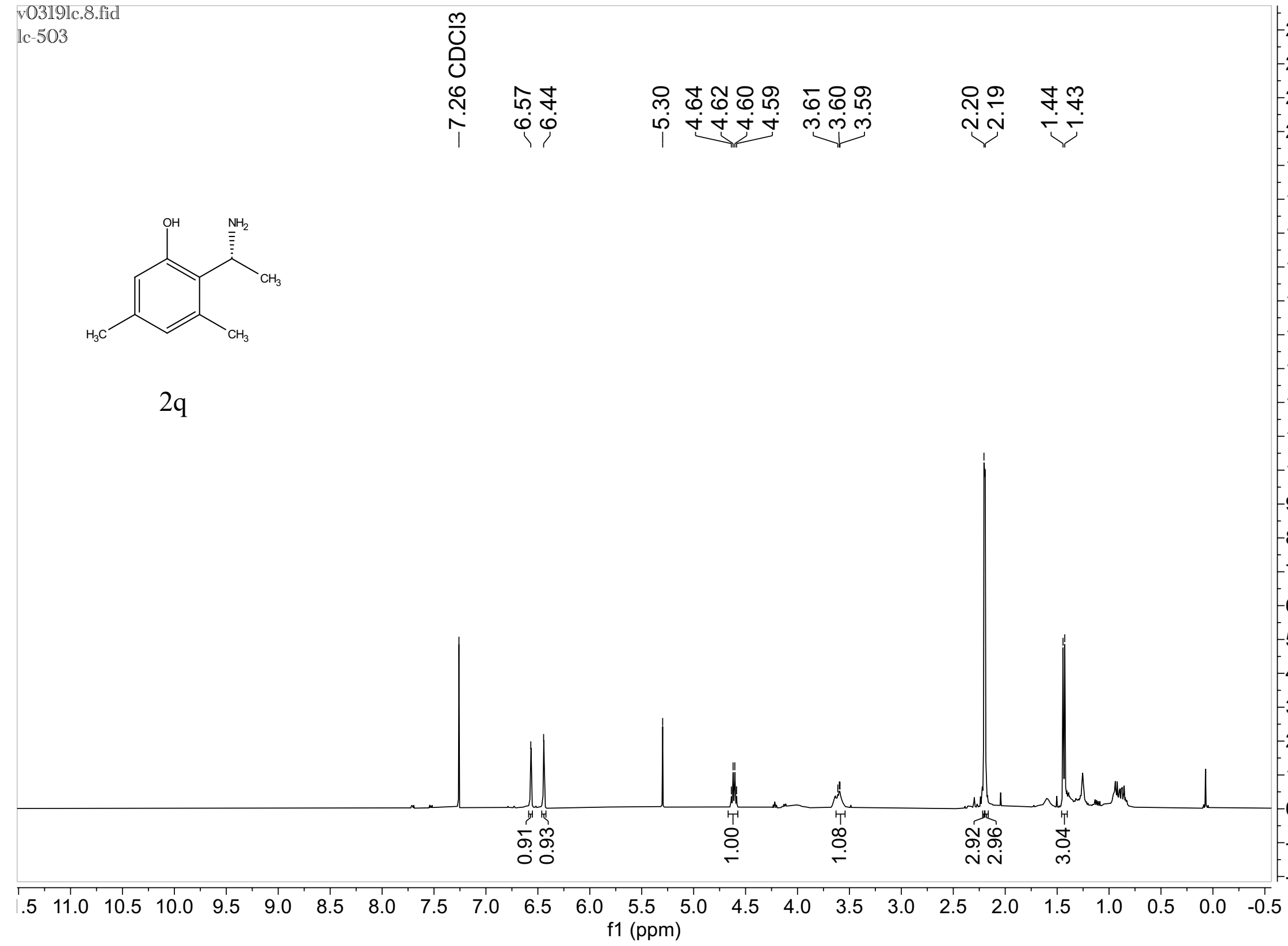
v0319lc.8.fid
lc-503
7.26 CDCl3
6.57
6.44
5.30
4.64
4.62
4.60
4.59
3.61
3.60
3.59
2.20
2.19
1.44
1.43
OH
NH2
CH3
H3C
CH3
2q
0.91
0.93
1.00
1.08
2.92
2.96
3.04
f1 (ppm)

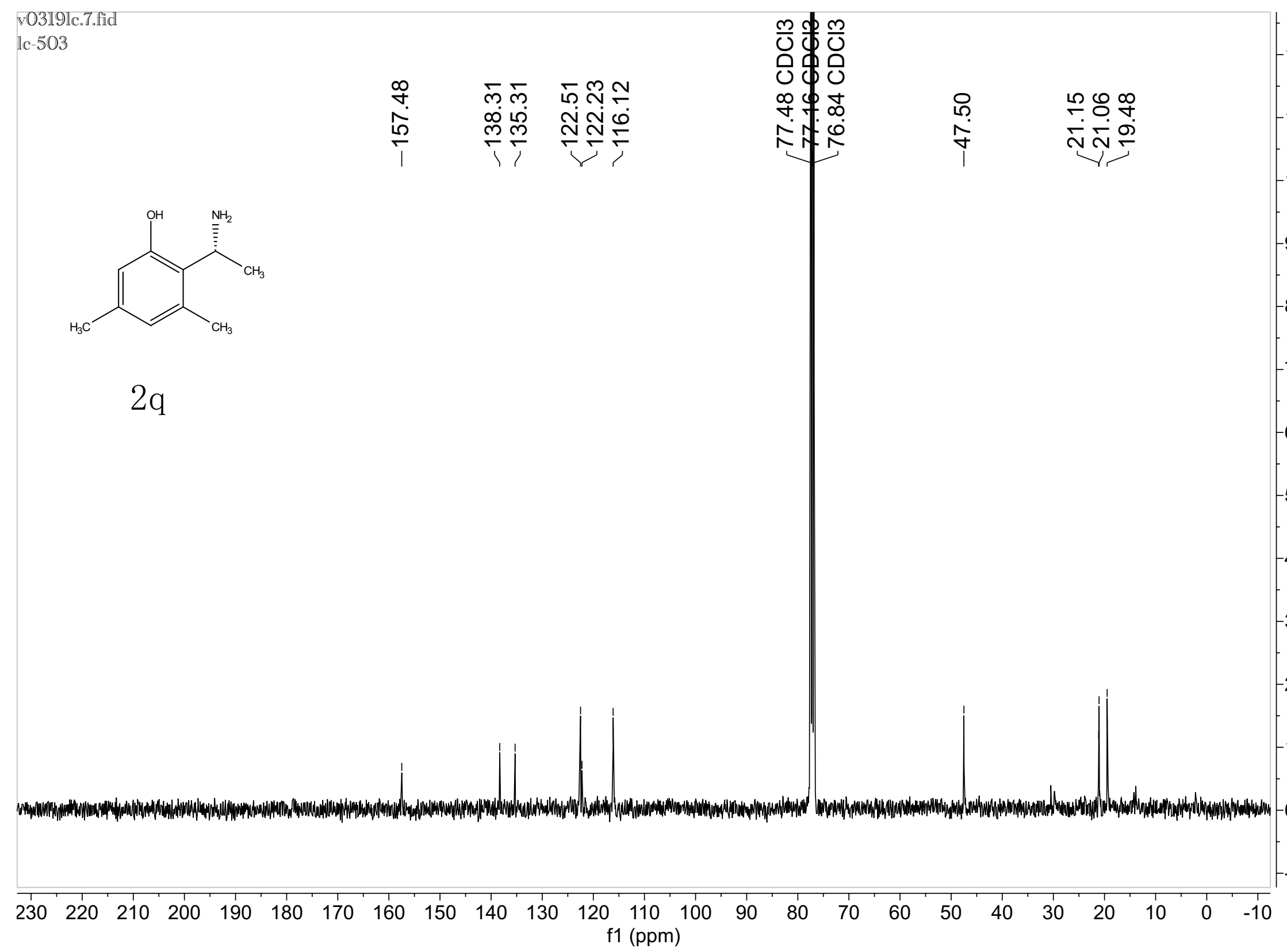
v0319lc.7.fid
lc-503
157.48
138.31
135.31
122.51
122.23
116.12
77.48 CDCl3
77.16 CDCl3
76.84 CDCl3
47.50
21.15
21.06
19.48
OH
NH2
CH3
H3C
CH3
2q
f1 (ppm)

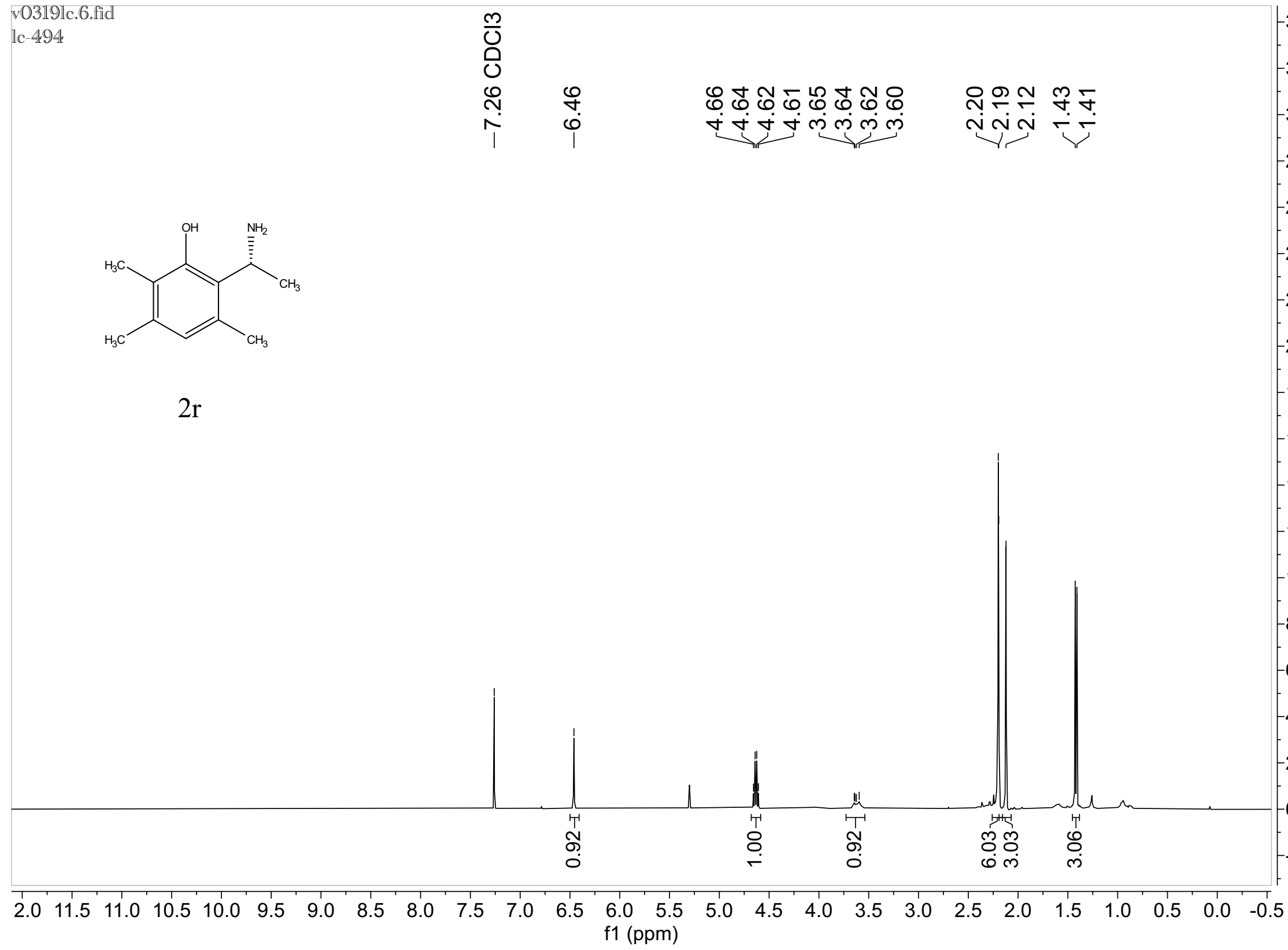
v0319lc.6.fid
lc-494
7.26 CDCl3
6.46
4.66
4.64
4.62
4.61
3.65
3.64
3.62
3.60
2.20
2.19
2.12
1.43
1.41
OH
NH2
H3C
CH3
H3C
CH3
2r
0.92
1.00
0.92
6.03
3.03
3.06
f1 (ppm)

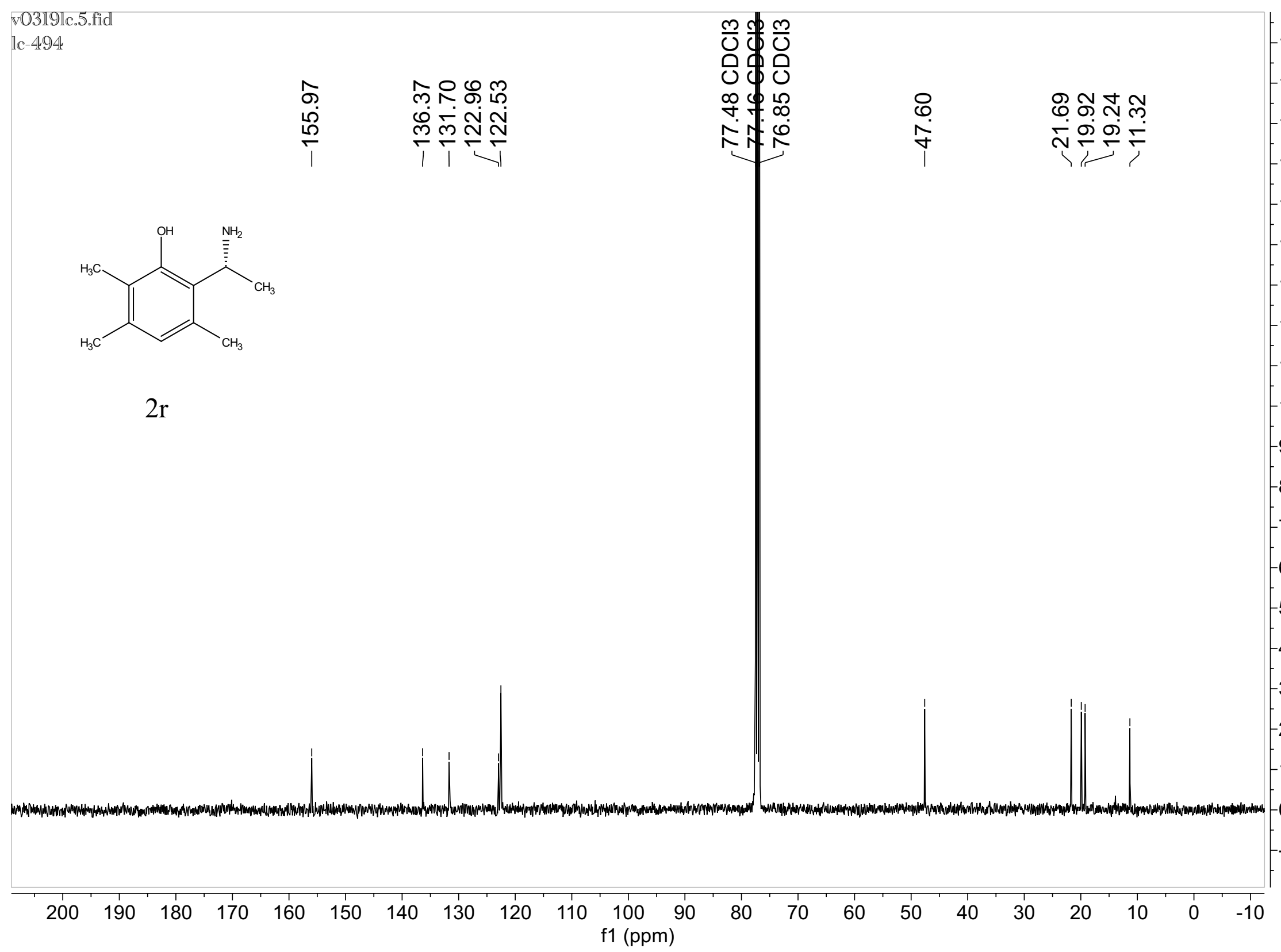
v0319lc.5.fid
lc-494
155.97
136.37
131.70
122.96
122.53
77.48 CDCl3
77.16 CDCl3
76.85 CDCl3
47.60
21.69
19.92
19.24
11.32
OH
NH2
H3C
CH3
H3C
CH3
2r
f1 (ppm)

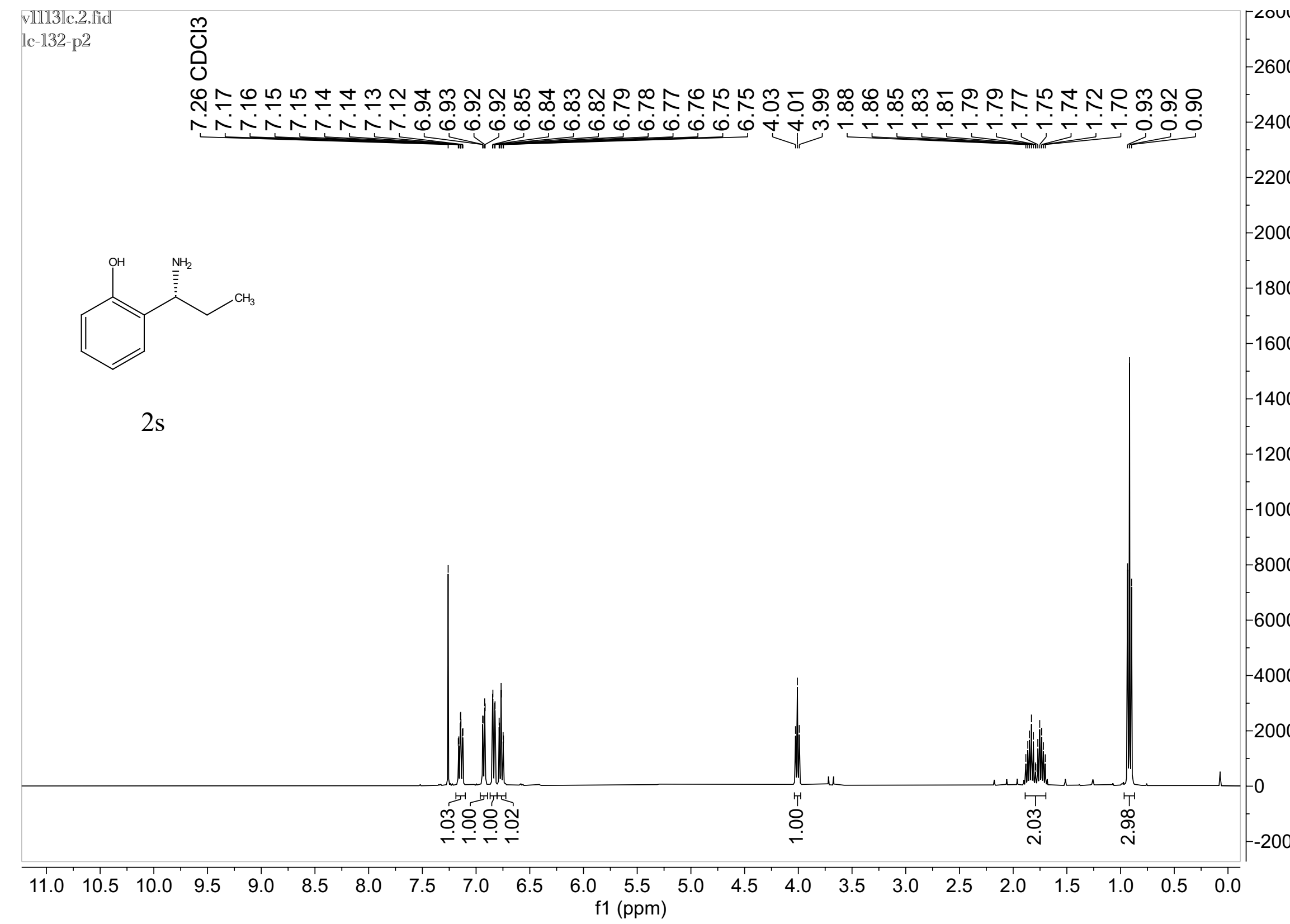
v1113lc.2.fid
lc-132-p2
7.26 CDCl3
7.17
7.16
7.15
7.15
7.14
7.14
7.13
7.12
6.94
6.93
6.92
6.92
6.85
6.84
6.83
6.82
6.79
6.78
6.77
6.76
6.75
6.75
4.03
4.01
3.99
1.88
1.86
1.85
1.83
1.81
1.79
1.79
1.77
1.75
1.74
1.72
1.70
0.93
0.92
0.90
OH
NH2
CH3
2s
1.03
1.00
1.00
1.02
1.00
2.03
2.98
f1 (ppm)

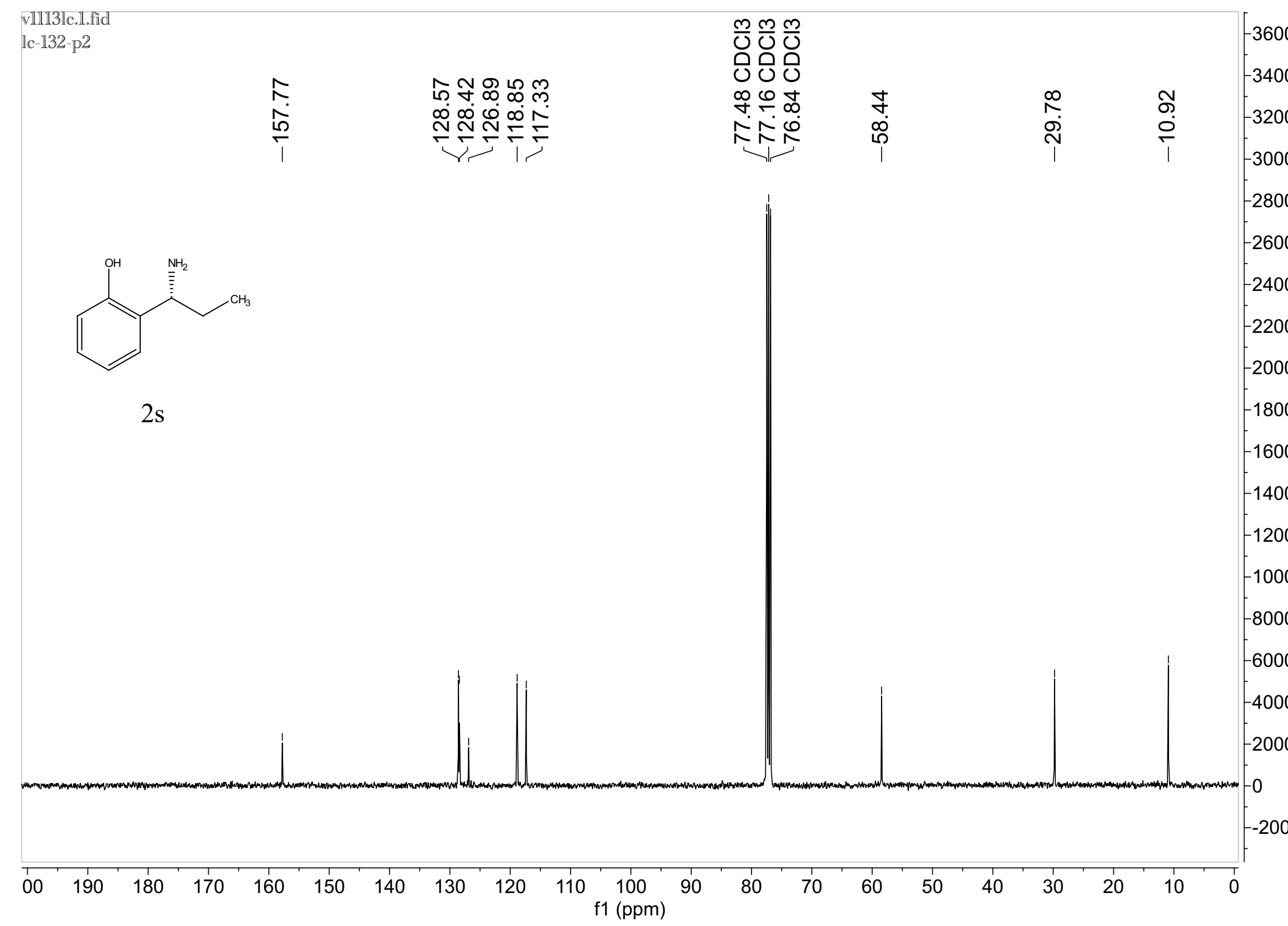
v1113lc.1.fid
lc-132-p2
157.77
128.57
128.42
126.89
118.85
117.33
77.48 CDCl3
77.16 CDCl3
76.84 CDCl3
58.44
29.78
10.92
OH
NH2
CH3
2s
f1 (ppm)

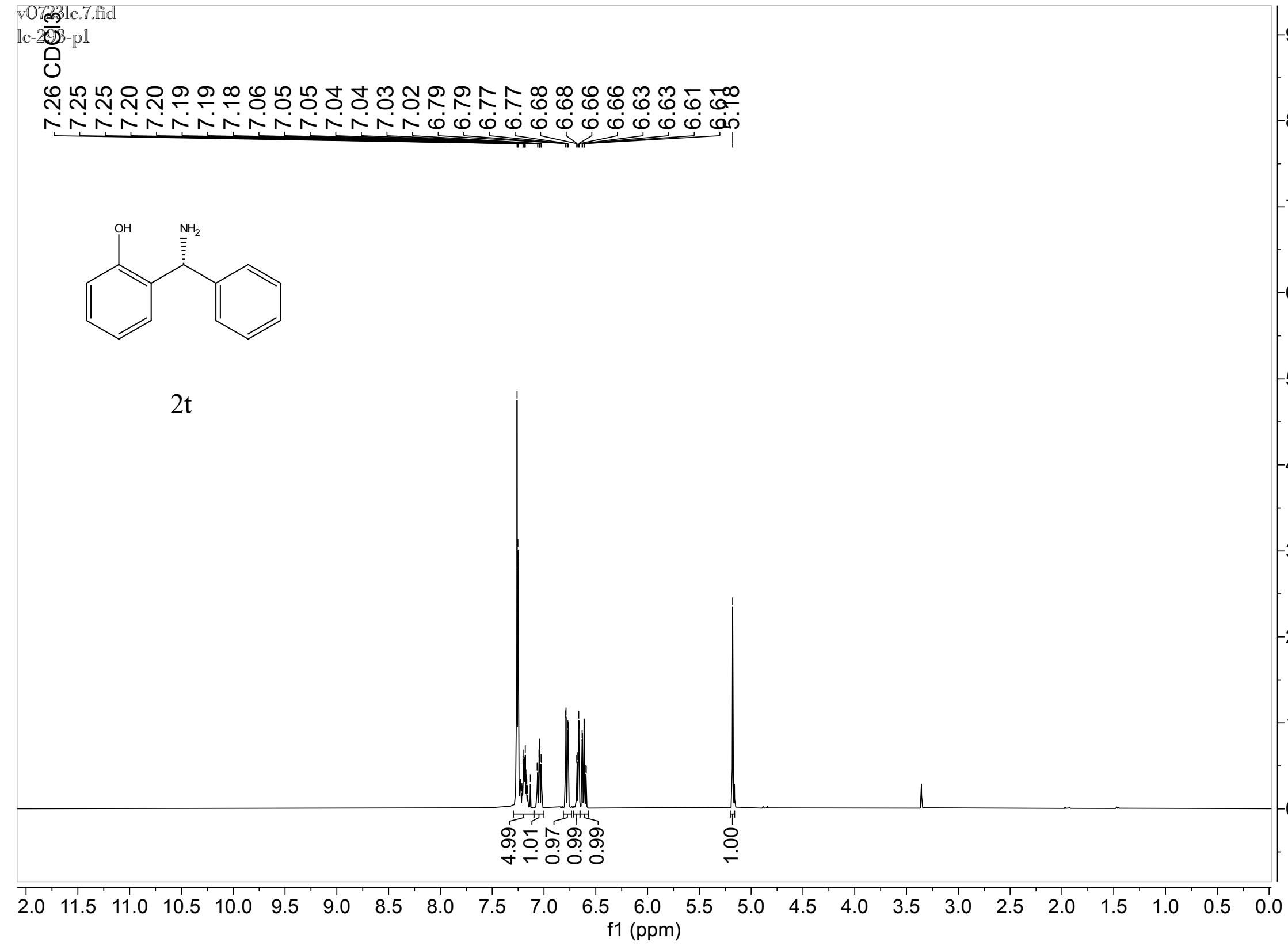
v0723lc.7.fid
lc-293-p1
7.26 CDCl3
7.25
7.25
7.20
7.20
7.19
7.19
7.18
7.06
7.05
7.05
7.04
7.04
7.03
7.02
6.79
6.77
6.77
6.68
6.68
6.66
6.66
6.63
6.63
6.61
6.61
5.18
OH
NH2
2t
4.99
1.01
0.97
0.99
0.99
1.00
f1 (ppm)

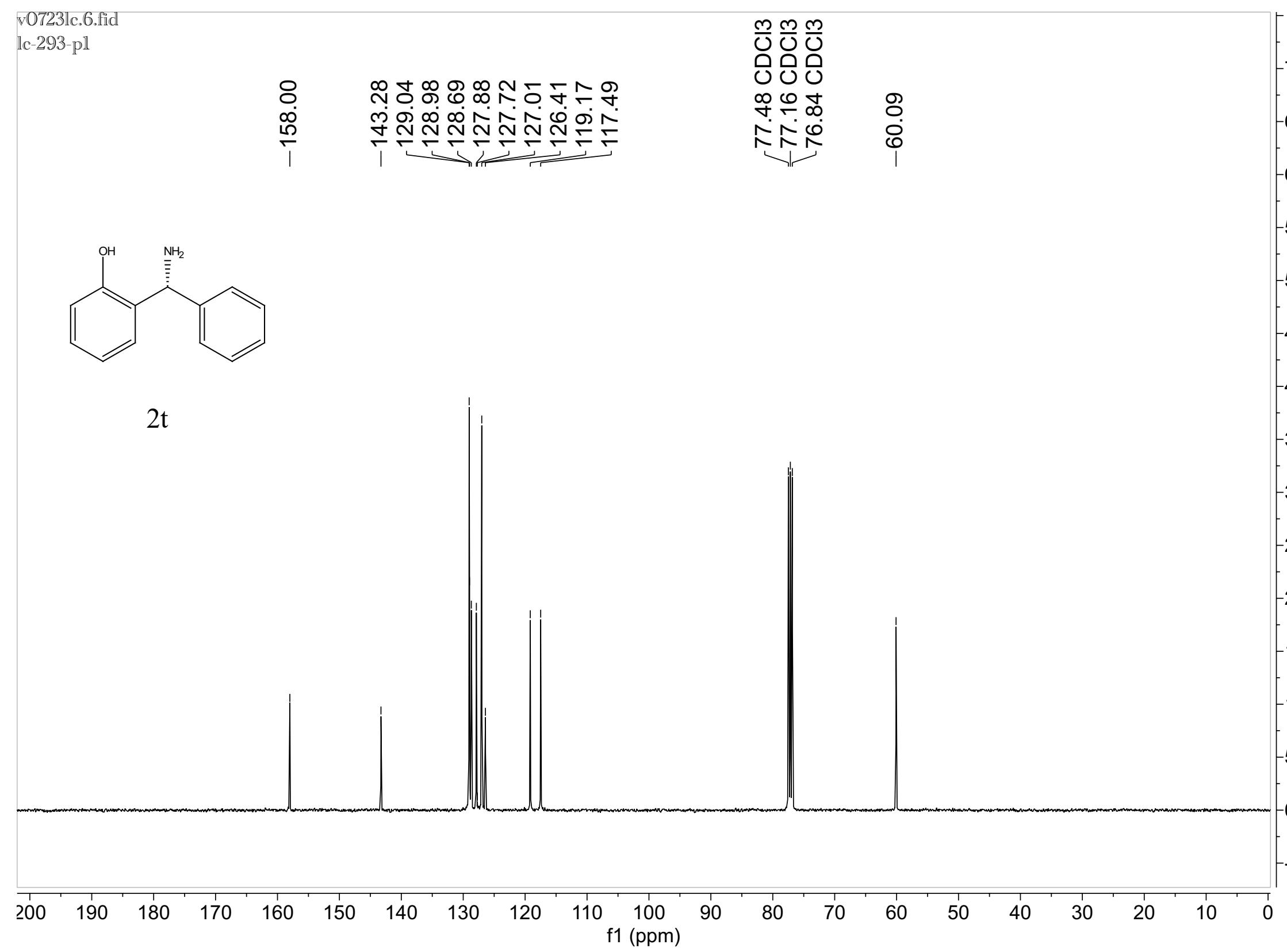
v0723lc.6.fid
lc-293-p1
158.00
143.28
129.04
128.98
128.69
127.88
127.72
127.01
126.41
119.17
117.49
77.48 CDCl3
77.16 CDCl3
76.84 CDCl3
60.09
OH
NH2
2t
f1 (ppm)

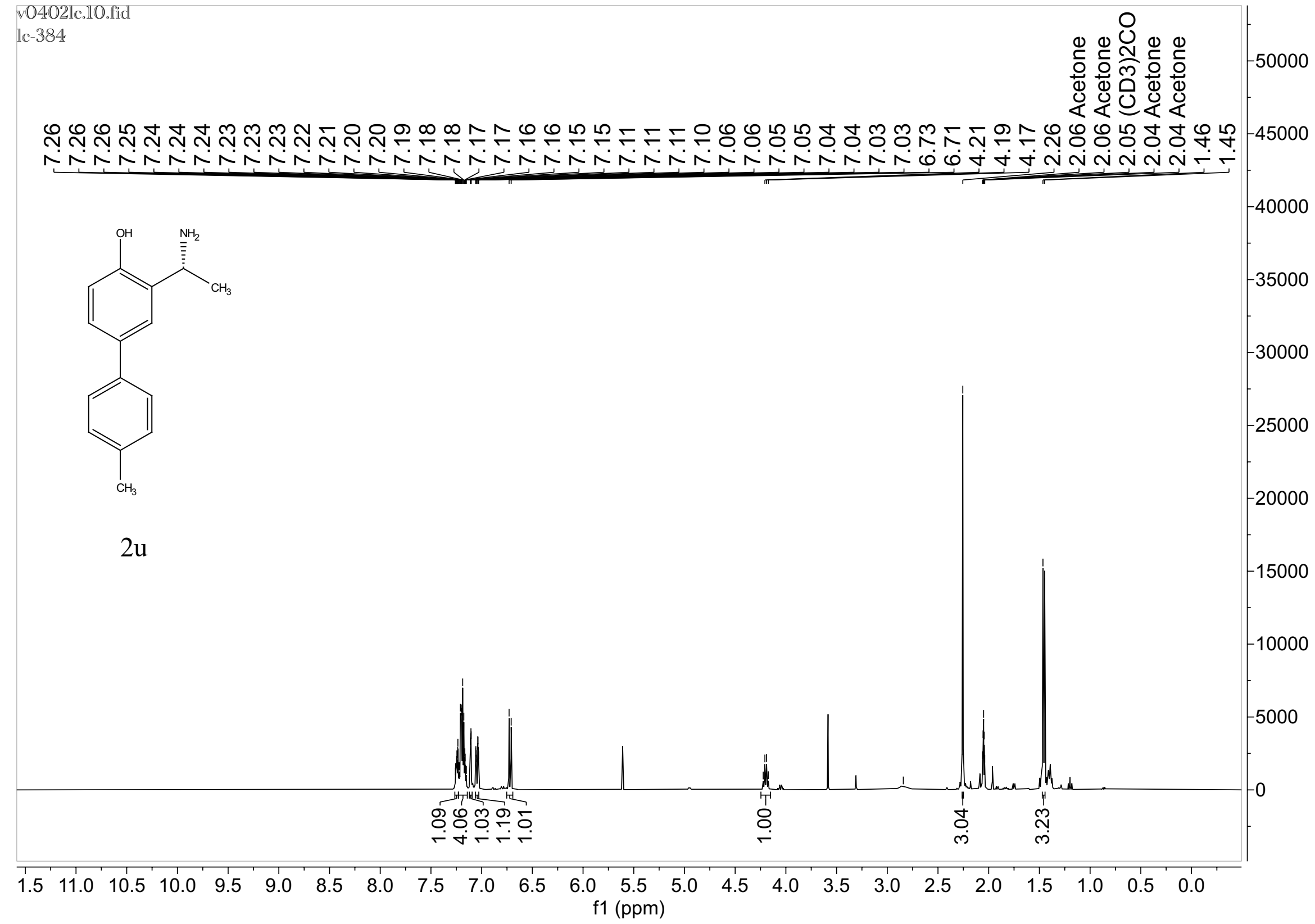
v0402lc.10.fid
lc-384
7.26 7.26 7.26 7.25 7.24 7.24 7.24 7.23 7.23 7.23 7.22 7.21 7.20 7.20 7.19 7.18 7.17 7.17 7.16 7.16 7.15 7.15 7.11 7.11 7.11 7.10 7.06 7.06 7.05 7.05 7.04 7.04 7.03 7.03 6.73 6.71 4.21 4.19 4.17 2.26 2.06 Acetone 2.06 Acetone 2.05 (CD3)2CO 2.04 Acetone 2.04 Acetone 1.46 1.45
OH
NH2
CH3
CH3
2u
1.09 4.06 1.03 1.19 1.01 1.00 3.04 3.23
f1 (ppm)

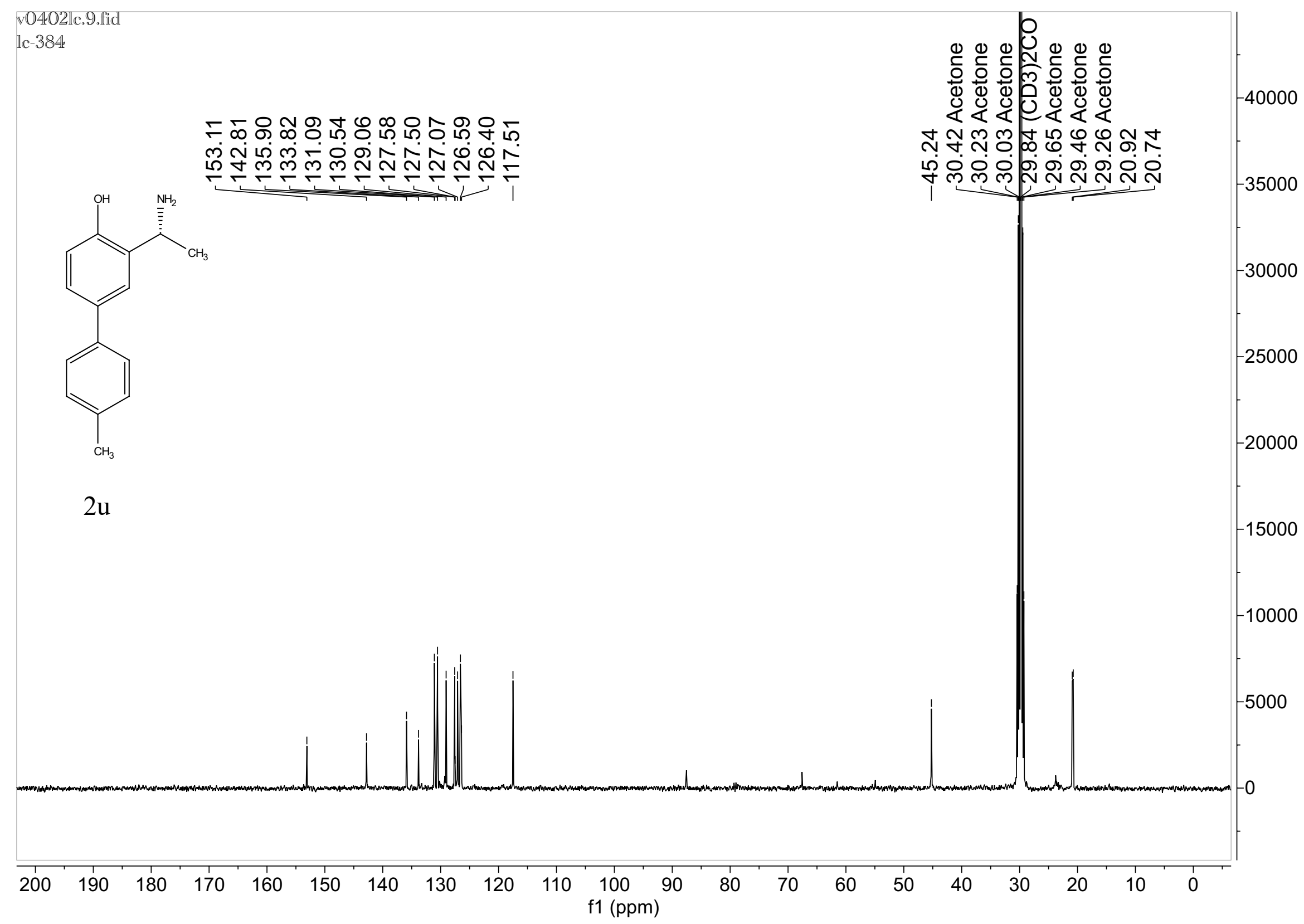
v0402lc.9.fid
lc-384
153.11 142.81 135.90 133.82 131.09 130.54 129.06 127.58 127.50 127.07 126.59 126.40 117.51
45.24 30.42 Acetone 30.23 Acetone 30.03 Acetone 29.84 (CD3)2CO 29.65 Acetone 29.46 Acetone 29.26 Acetone 20.92 20.74
OH
NH2
CH3
CH3
2u
f1 (ppm)

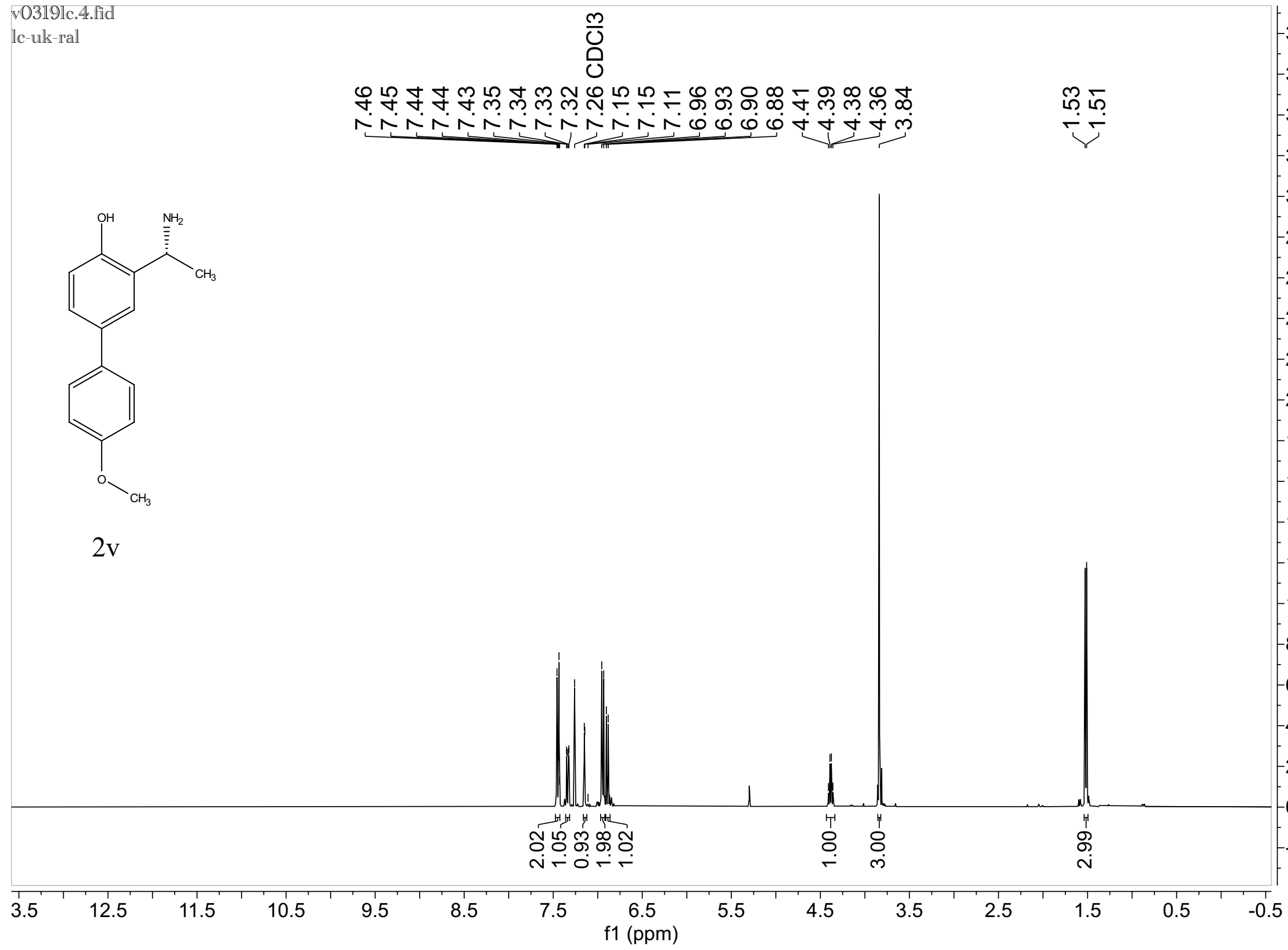
v0319lc.4.fid
lc-uk-ral
7.46 7.45 7.44 7.44 7.43 7.35 7.34 7.33 7.32 7.26 CDCl3 7.15 7.15 7.11 6.96 6.93 6.90 6.88 4.41 4.39 4.38 4.36 3.84
1.53 1.51
OH
NH2
CH3
O
CH3
2v
2.02 1.05 0.93 1.98 1.02 1.00 3.00 2.99
f1 (ppm)

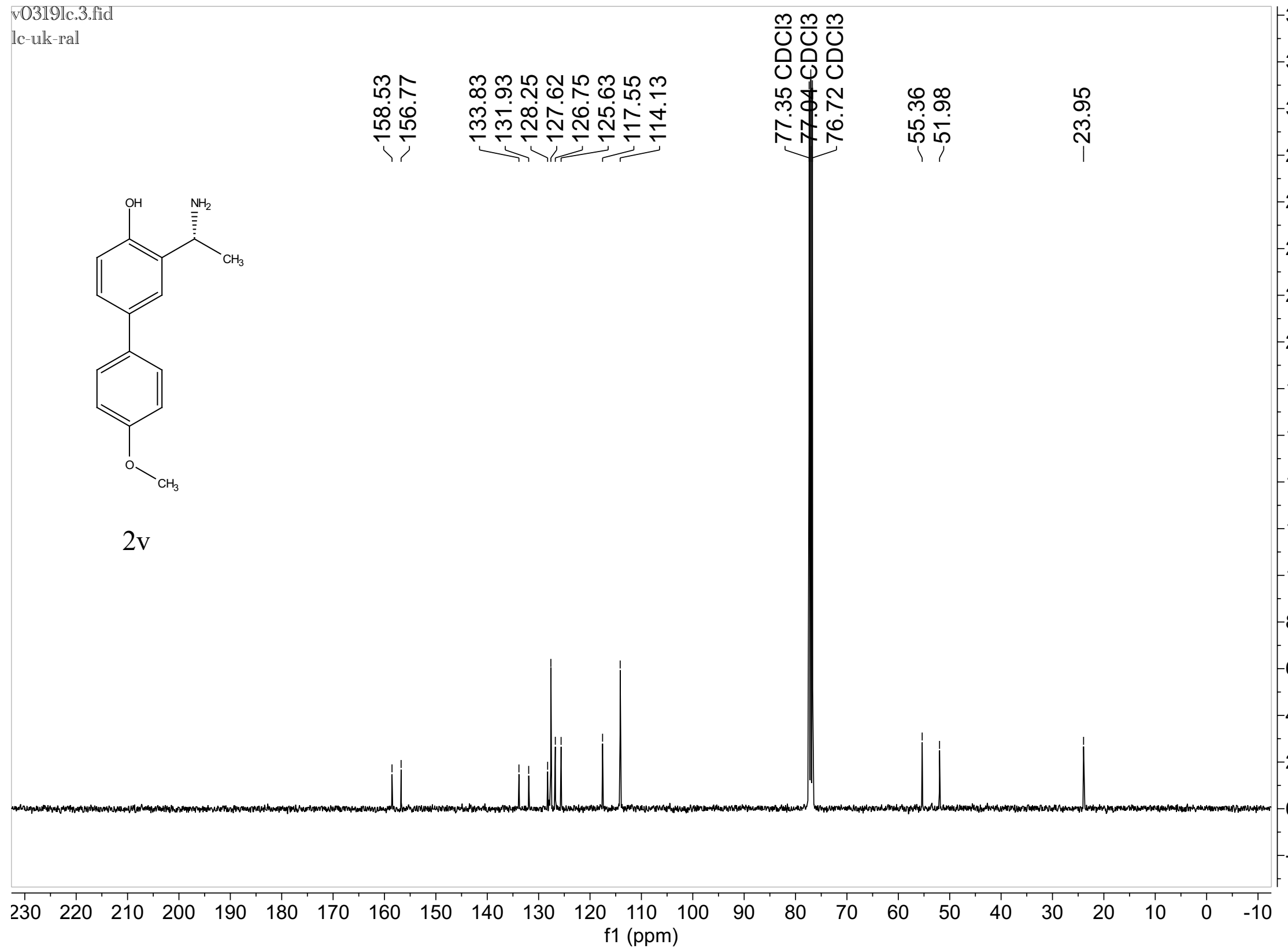
v0319lc.3.fid
lc-uk-ral
158.53 156.77 133.83 131.93 128.25 127.62 126.75 125.63 117.55 114.13
77.35 CDCl3 77.04 CDCl3 76.72 CDCl3
55.36 51.98 23.95
OH
NH2
CH3
O
CH3
2v
f1 (ppm)

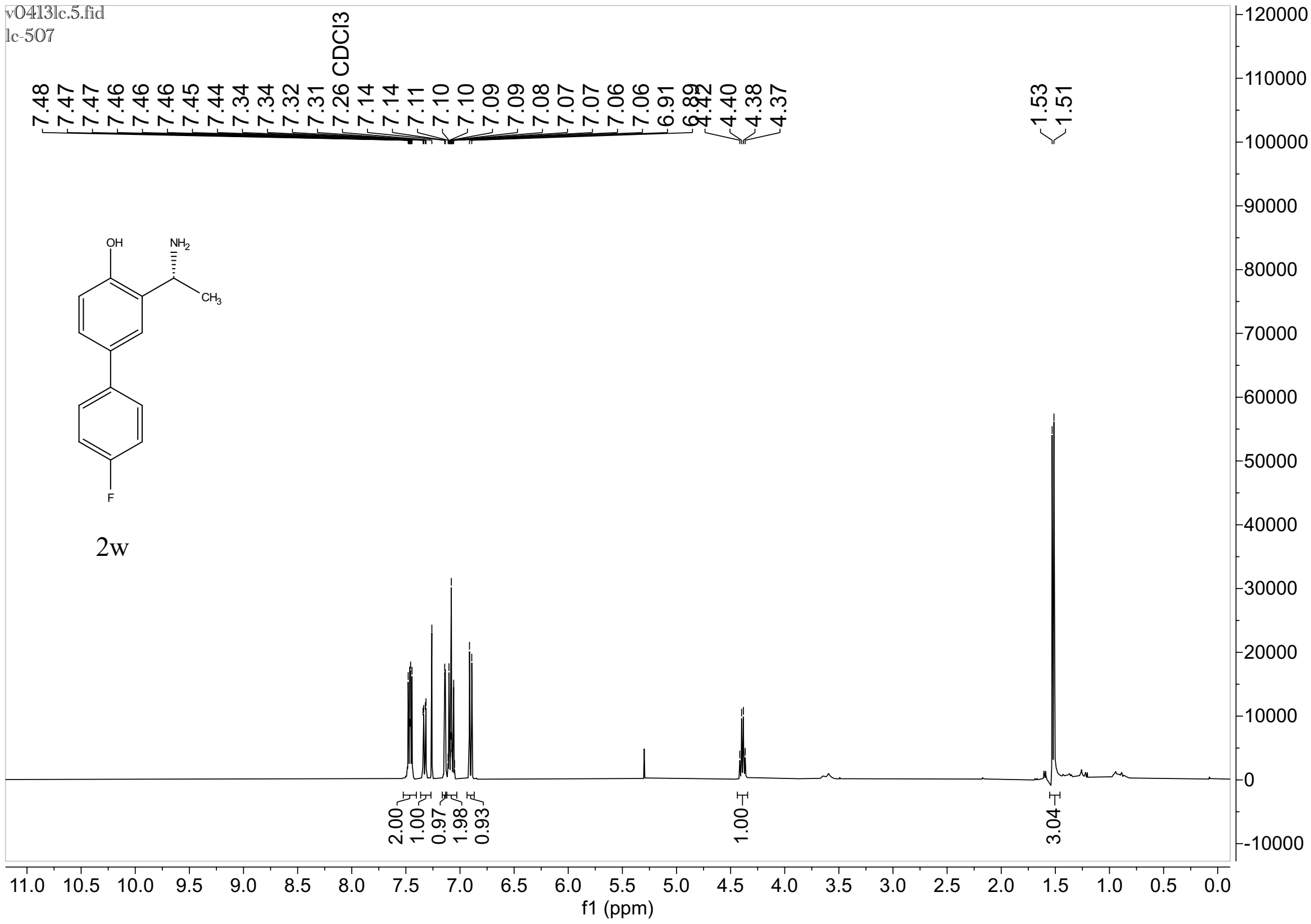
v0413lc.5.fid
lc-507
7.48 7.47 7.47 7.46 7.46 7.46 7.45 7.44 7.34 7.34 7.32 7.31 7.26 CDCl3 7.14 7.14 7.11 7.10 7.10 7.09 7.09 7.08 7.07 7.07 7.06 7.06 6.91 6.89 4.42 4.40 4.38 4.37
1.53 1.51
OH
NH2
CH3
F
2w
2.00 1.00 0.97 1.98 0.93
1.00
3.04
f1 (ppm)

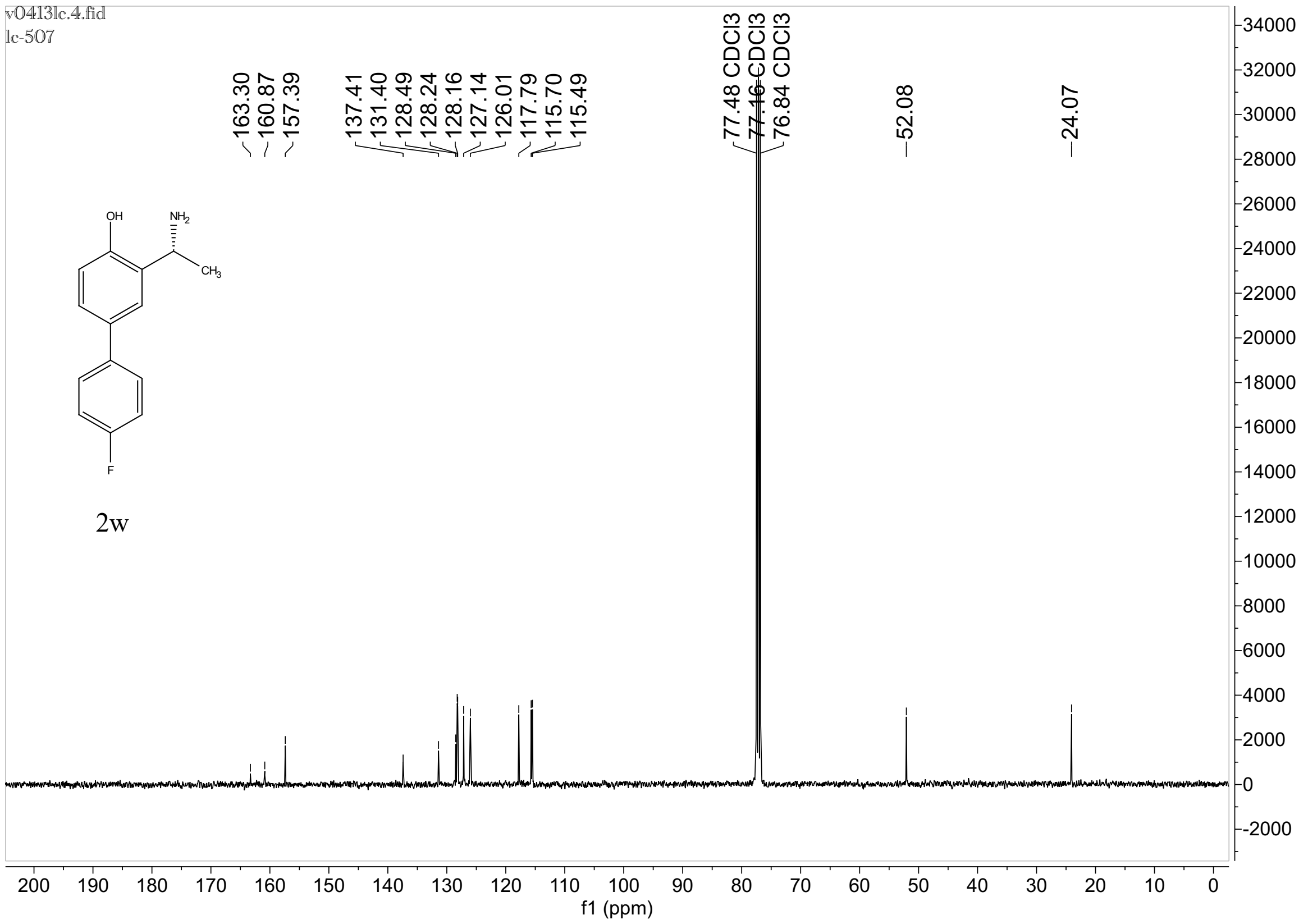
v0413lc.4.fid
lc-507
163.30 160.87 157.39
137.41 131.40 128.49 128.24 128.16 127.14 126.01 117.79 115.70 115.49
77.48 CDCl3 77.16 CDCl3 76.84 CDCl3
52.08
24.07
OH
NH2
CH3
F
2w
f1 (ppm)

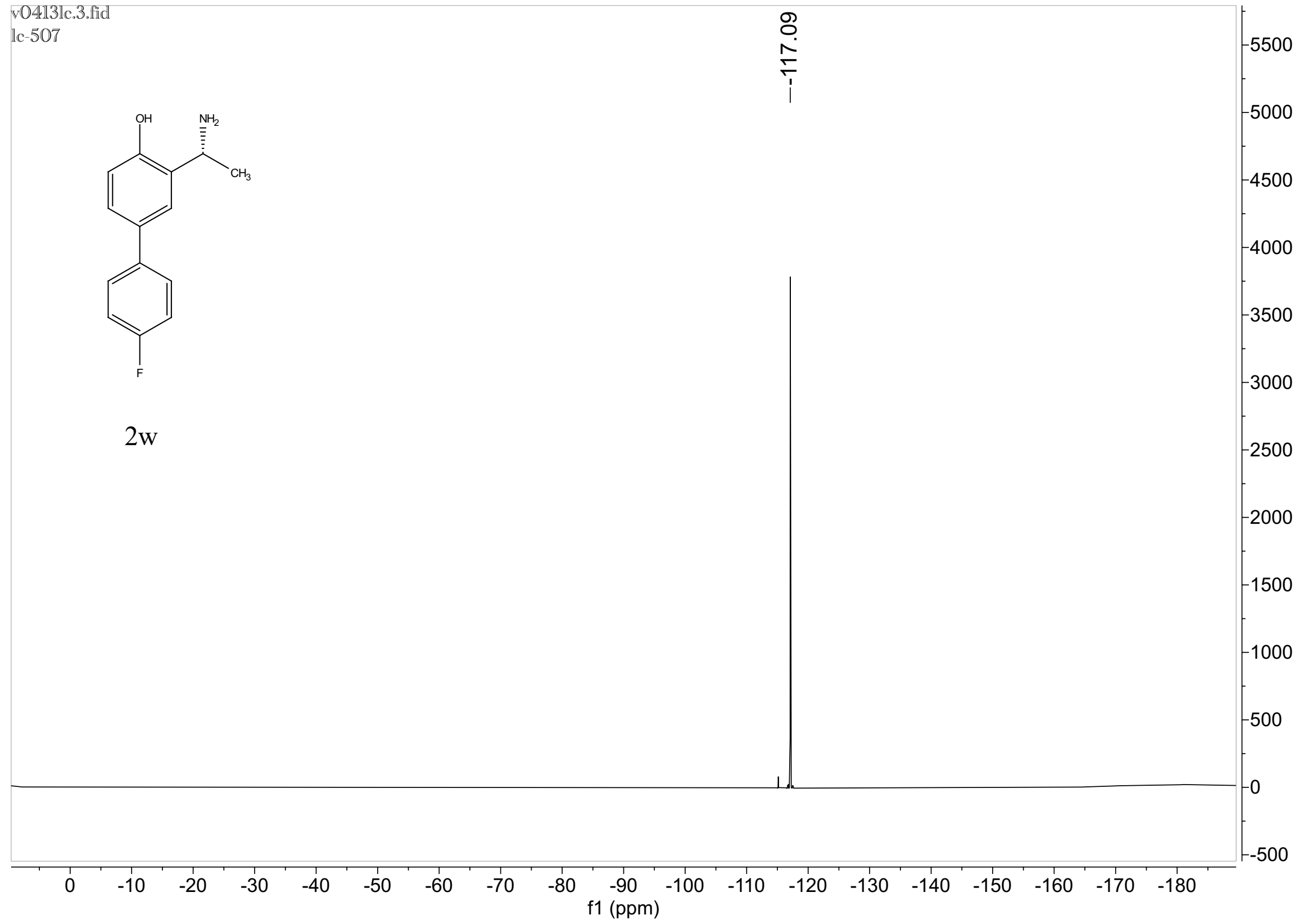
v0413lc.3.fid
lc-507
OH
NH2
CH3
F
2w
-117.09
5500
5000
4500
4000
3500
3000
2500
2000
1500
1000
500
0
-500
0
-10
-20
-30
-40
-50
-60
-70
-80
-90
-100
-110
-120
-130
-140
-150
-160
-170
-180
f1 (ppm)

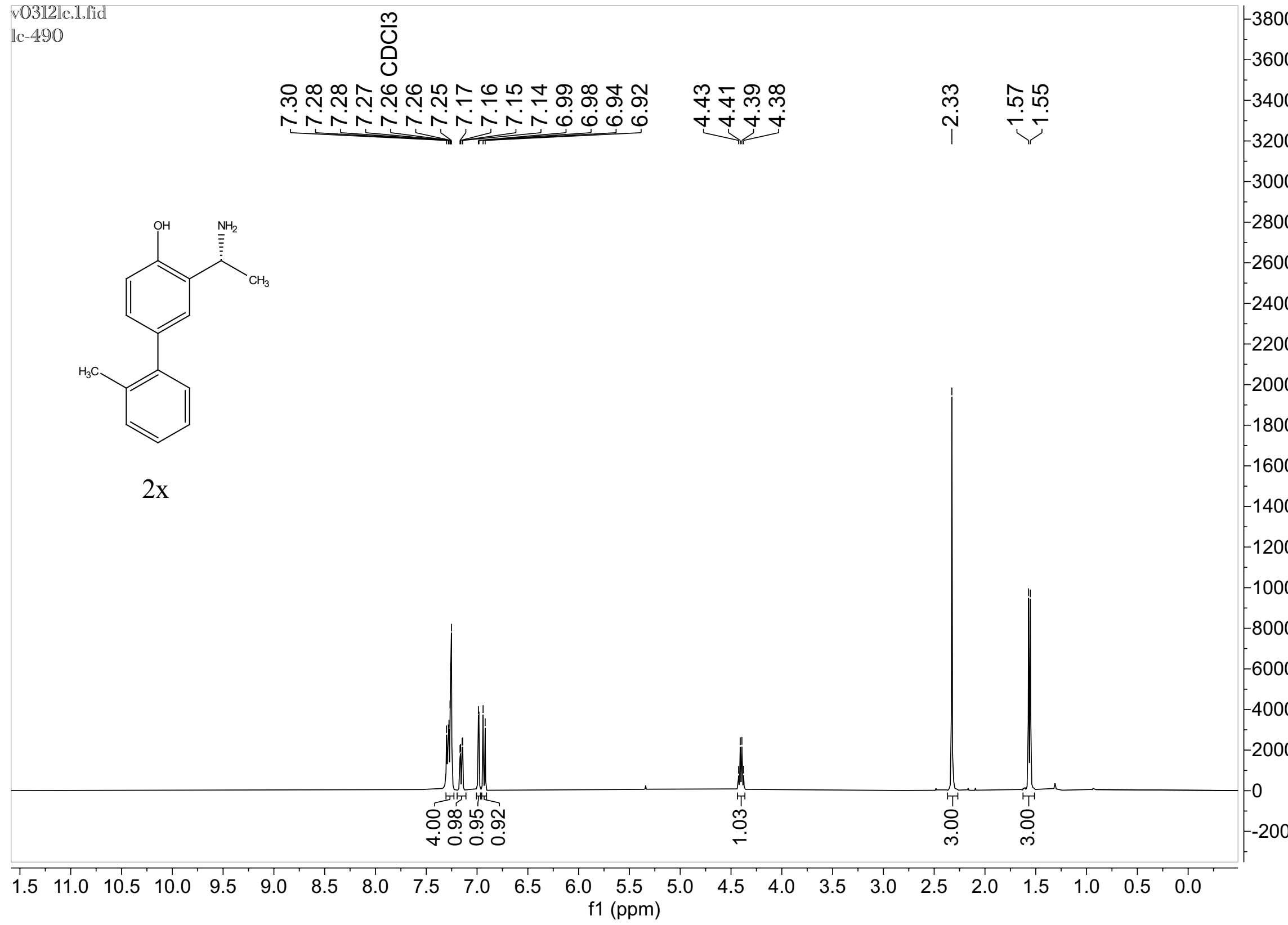

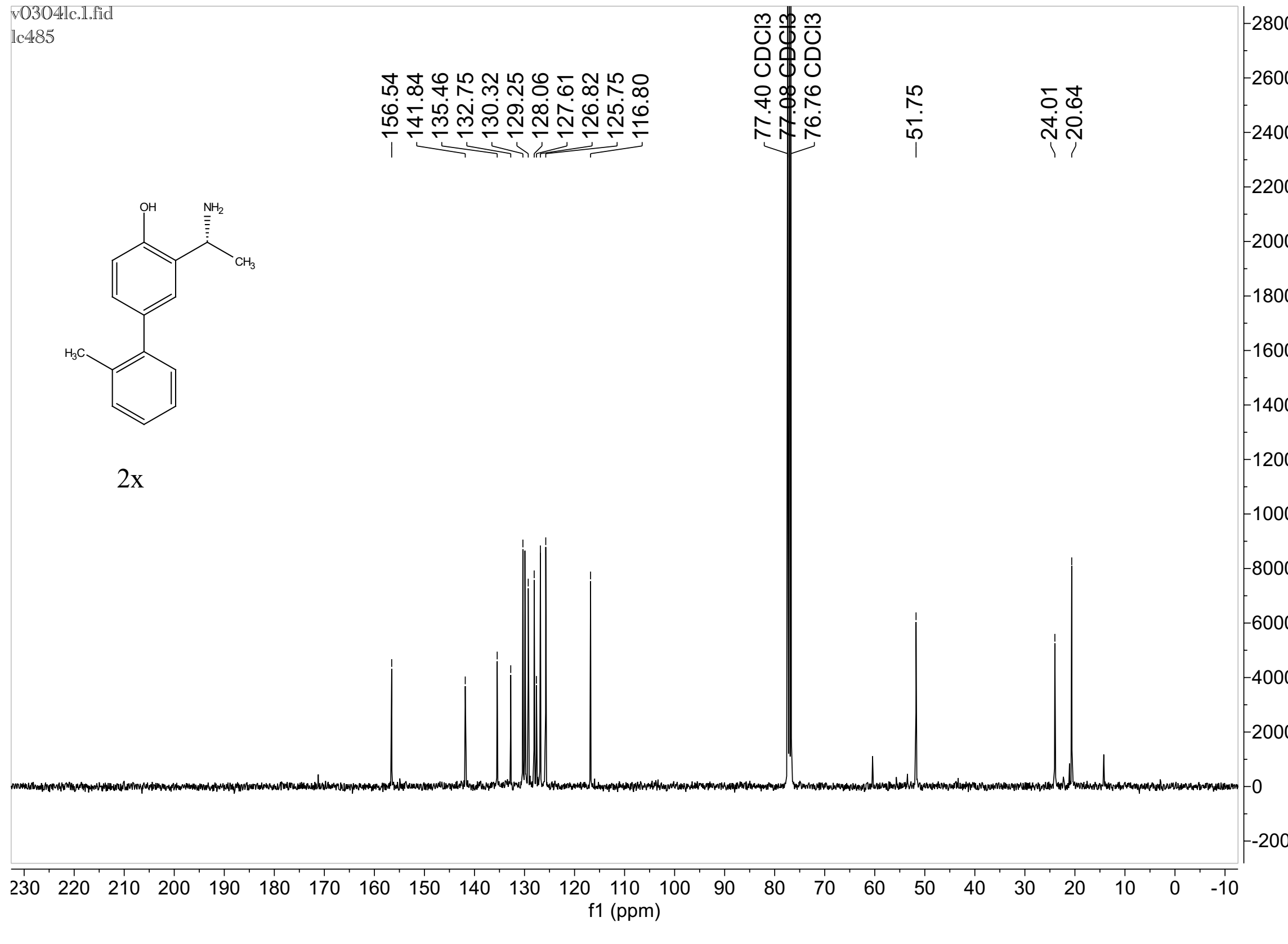

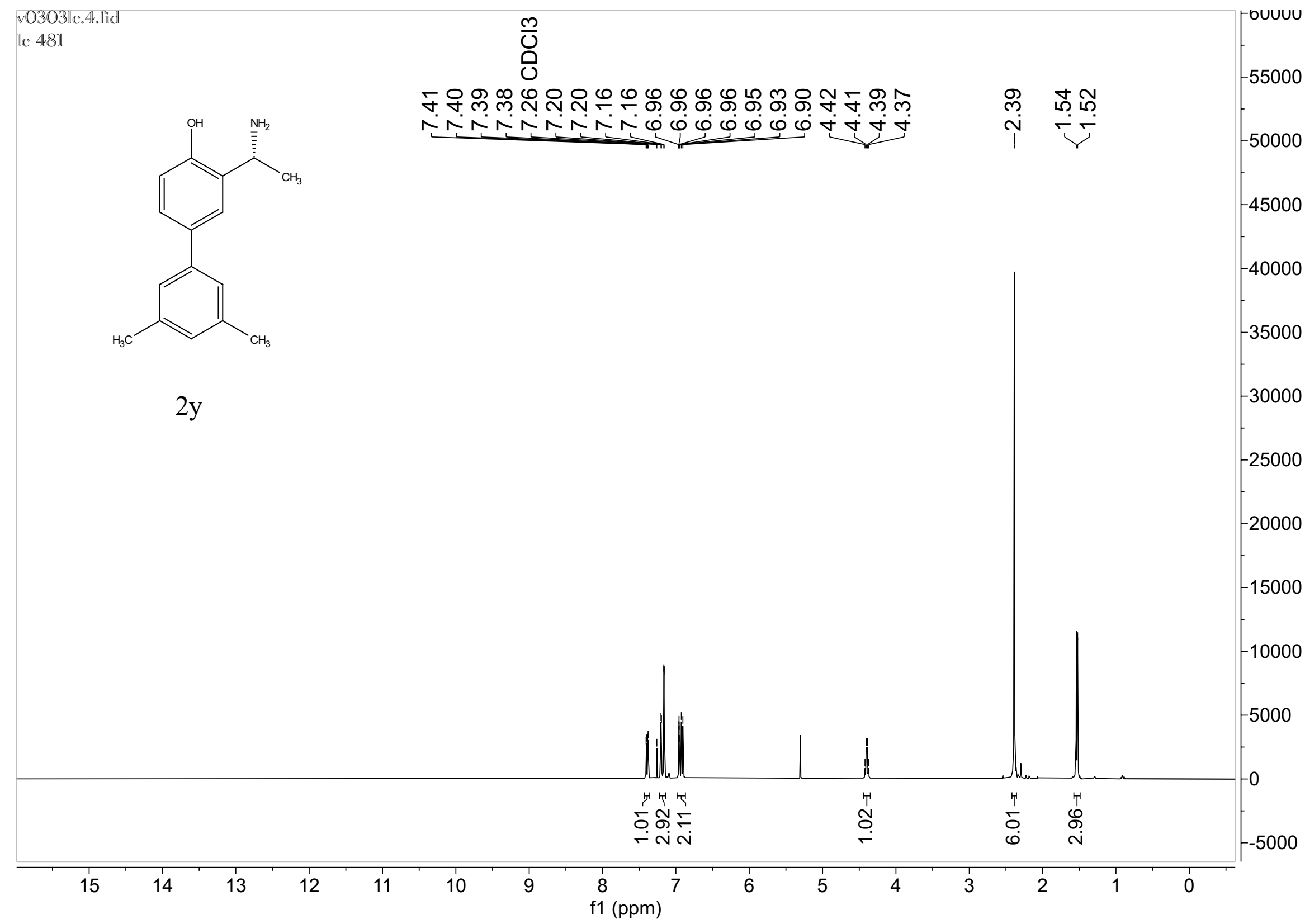
v0303lc.4.fid
lc-481
2y
7.41 7.40 7.39 7.38 7.26 CDCl3 7.20 7.20 7.16 7.16 6.96 6.96 6.96 6.95 6.93 6.90 4.42 4.41 4.39 4.37 2.39 1.54 1.52
1.01 2.92 2.11 1.02 6.01 2.96
f1 (ppm)

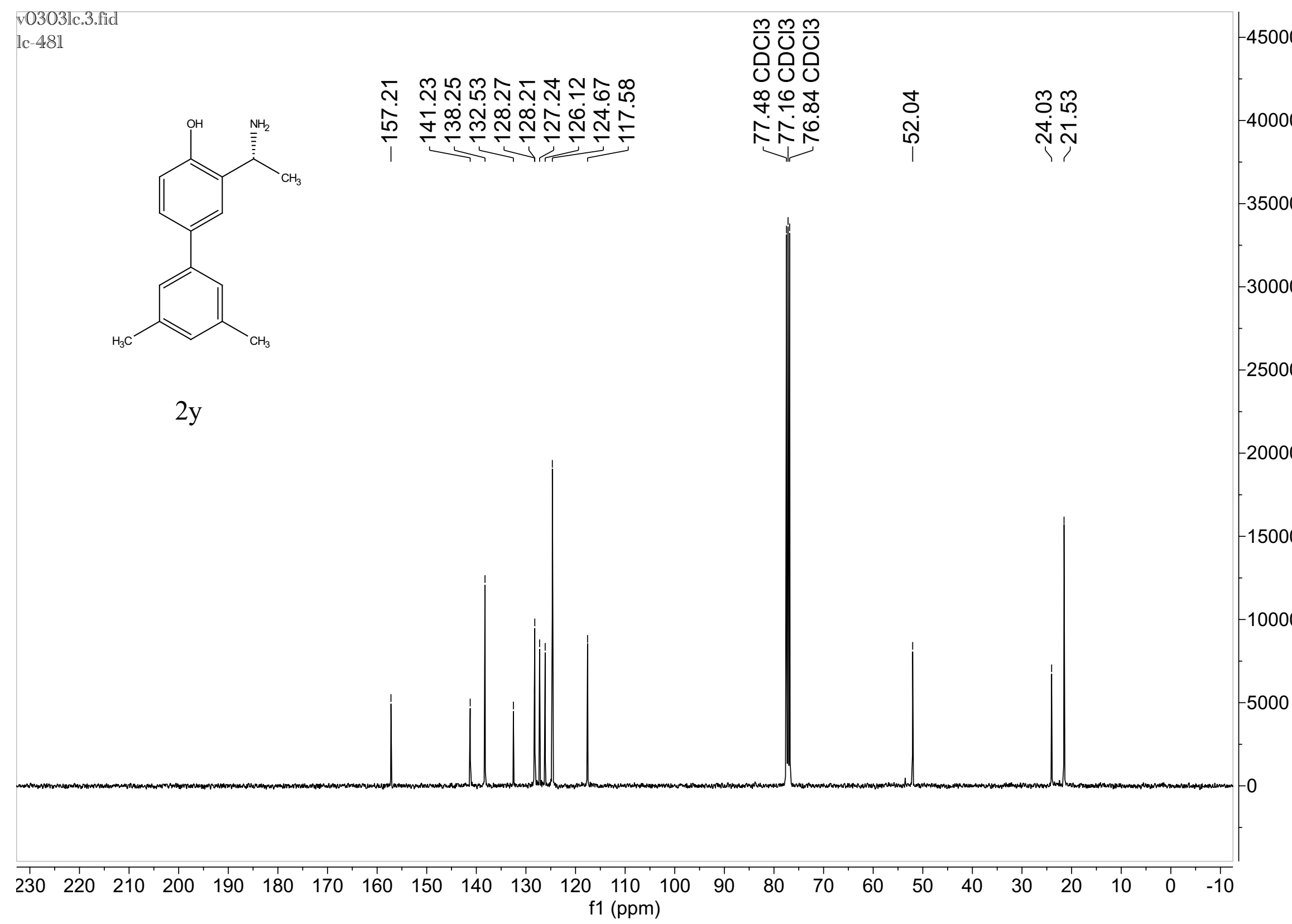
v0303lc.3.fid
lc-481
2y
157.21 141.23 138.25 132.53 128.27 128.21 127.24 126.12 124.67 117.58 77.48 CDCl3 77.16 CDCl3 76.84 CDCl3 52.04 24.03 21.53
f1 (ppm)

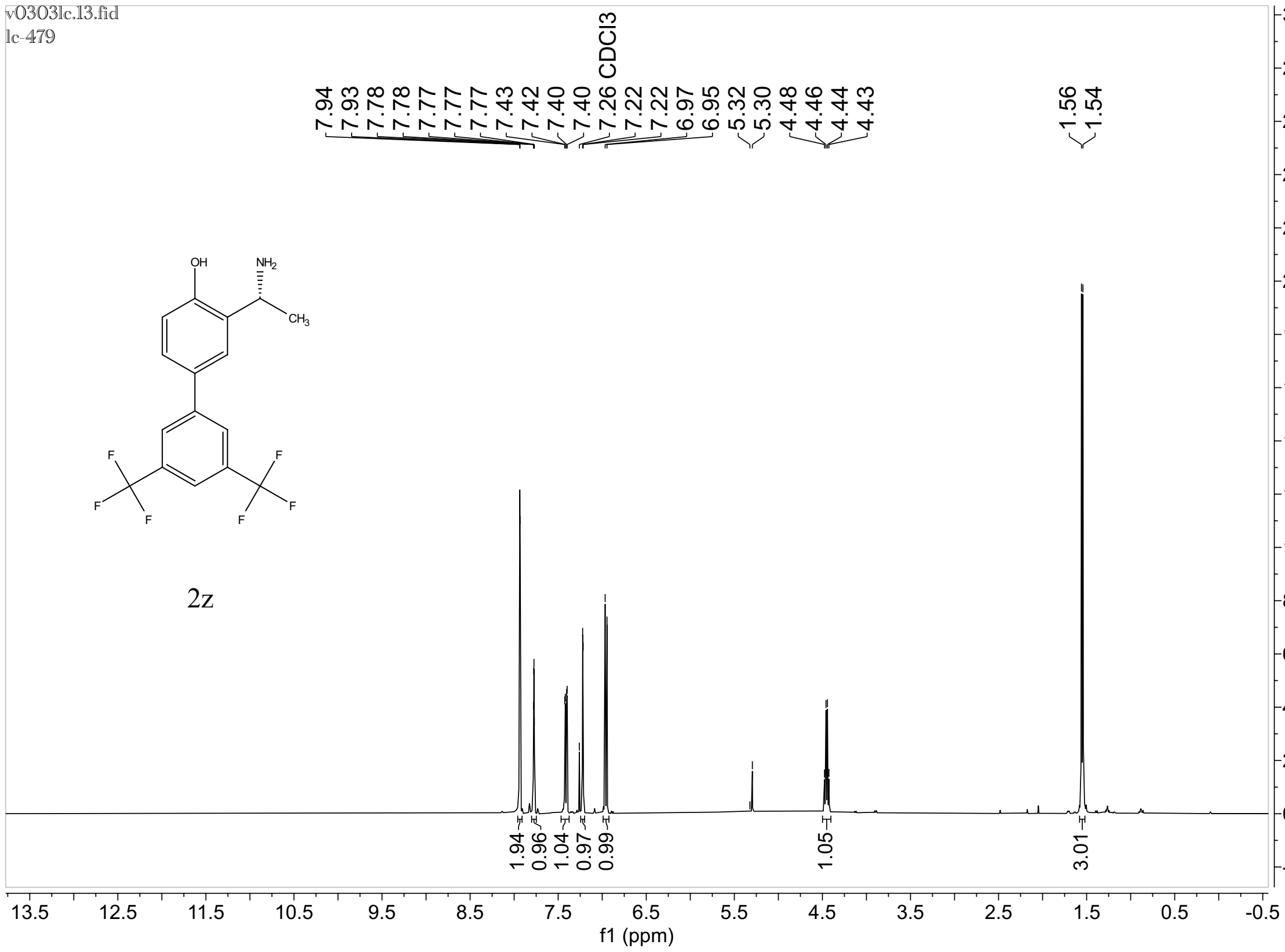

v0303lc.13.fid
lc-479
7.94 7.93 7.78 7.78 7.77 7.77 7.77 7.43 7.42 7.40 7.40 7.26 CDCl3 7.22 7.22 7.22 6.97 6.95 5.32 5.30 4.48 4.46 4.44 4.43 1.56 1.54
OH
NH2
CH3
F
2z
1.94 0.96 1.04 0.97 0.99 1.05 3.01
f1 (ppm)

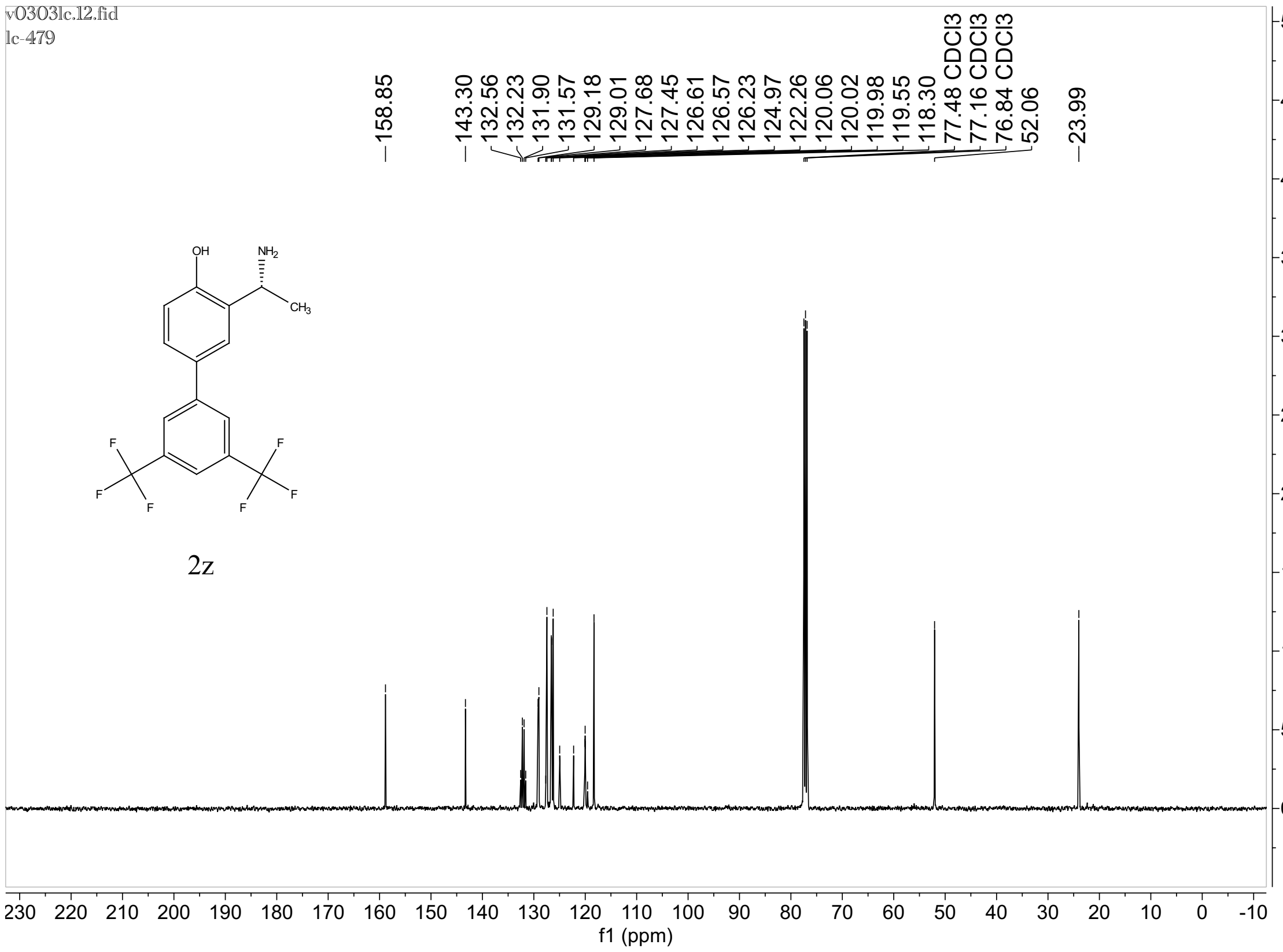

v0303lc.12.fid
lc-479
158.85 143.30 132.56 132.23 131.90 131.57 129.18 129.01 127.68 127.45 126.61 126.57 126.23 124.97 122.26 120.06 120.02 119.98 119.55 118.30 77.48 CDCl3 77.16 CDCl3 76.84 CDCl3 52.06 23.99
OH
NH2
CH3
F
2z
f1 (ppm)

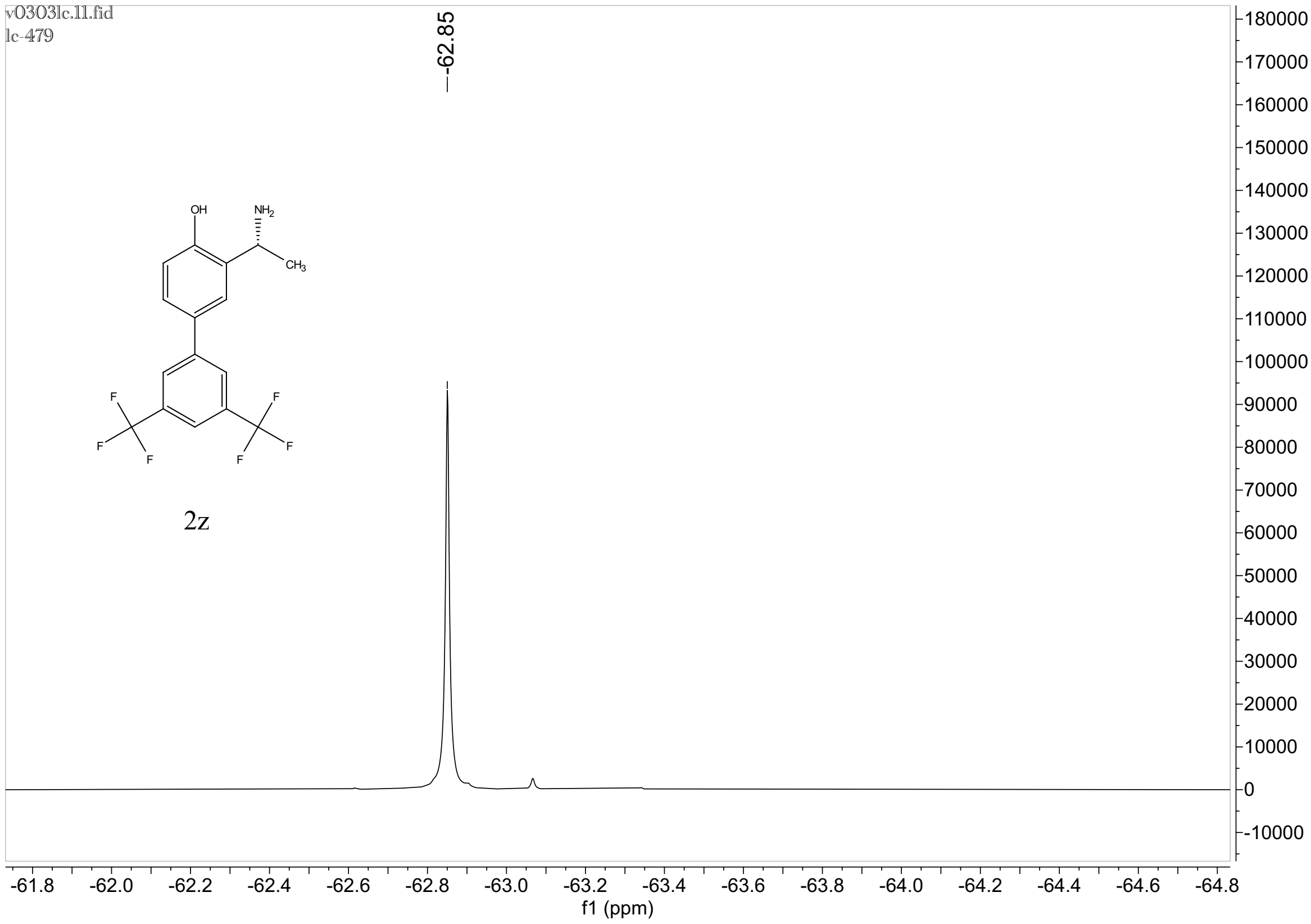

v0303lc.11.fid
lc-479
-62.85
OH
NH2
CH3
F
2z
f1 (ppm)

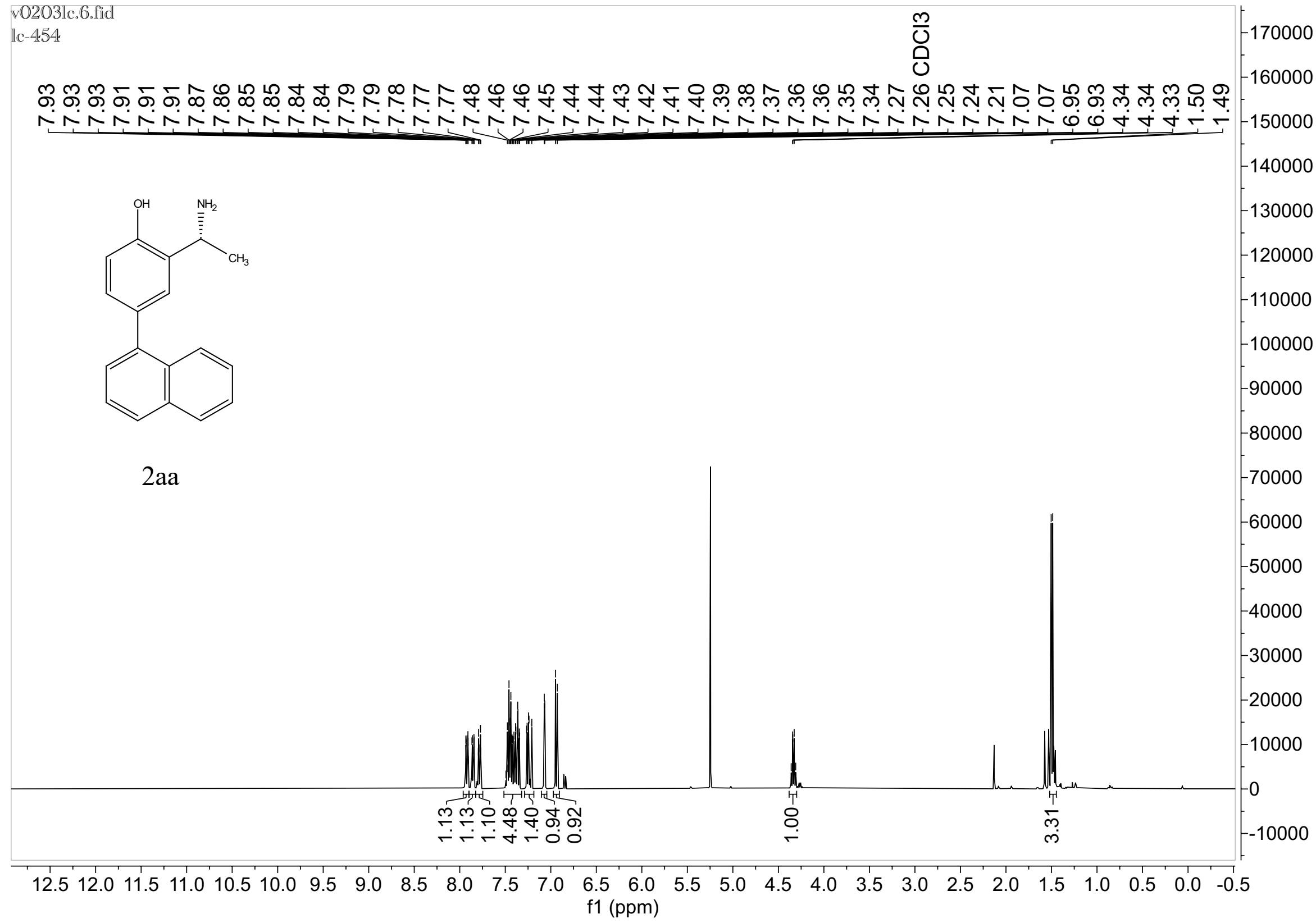

2aa

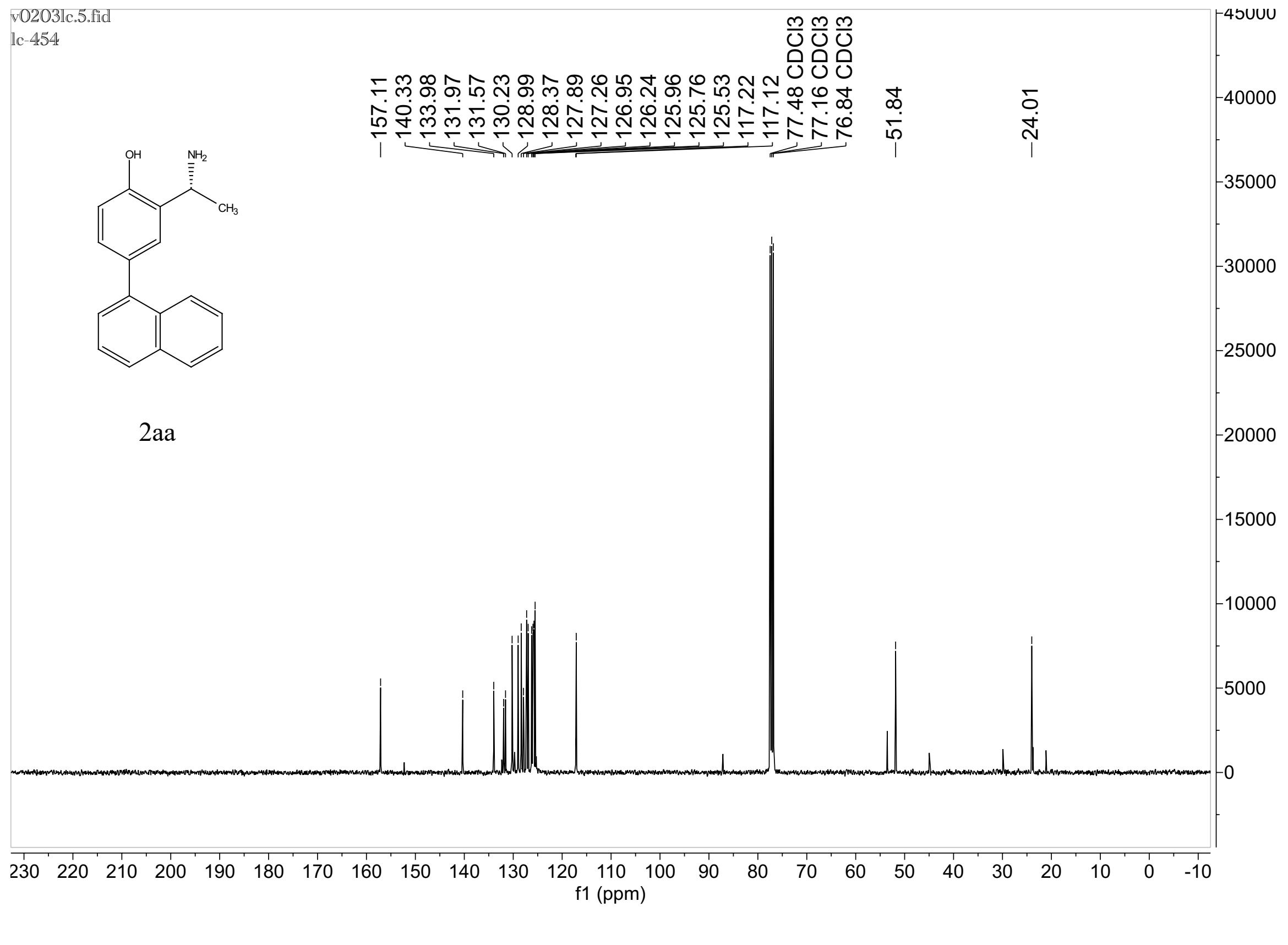

2aa

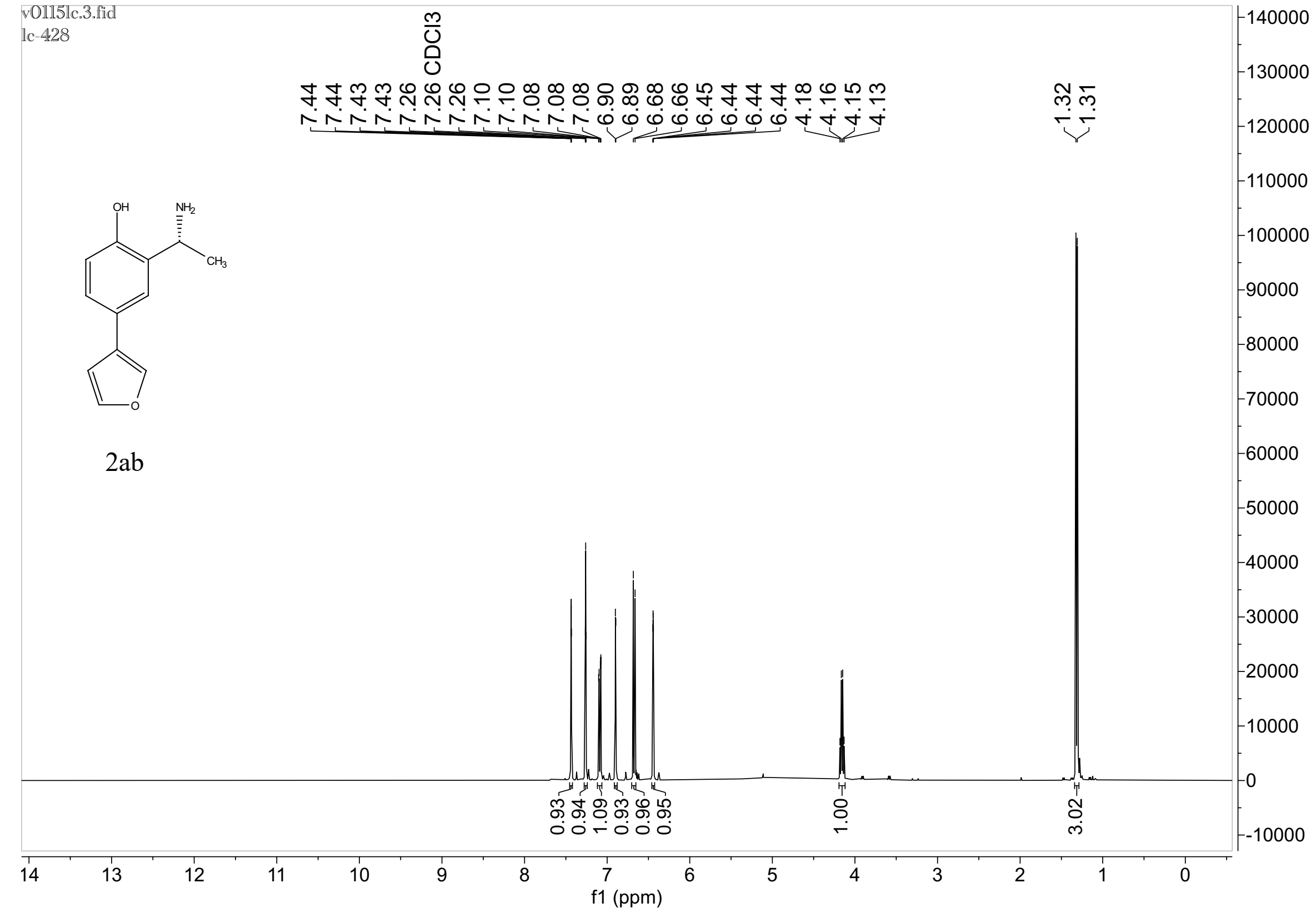
v0115lc.3.fid
lc-428
7.44 7.44 7.43 7.43 7.26 7.26 CDCl3 7.26 7.10 7.10 7.08 7.08 7.08 6.90 6.89 6.68 6.66 6.45 6.44 6.44 4.18 4.16 4.15 4.13
1.32 1.31
OH
NH_2
CH_3
O
2ab
0.93 0.94 1.09 0.93 0.96 0.95
1.00
3.02
f1 (ppm)

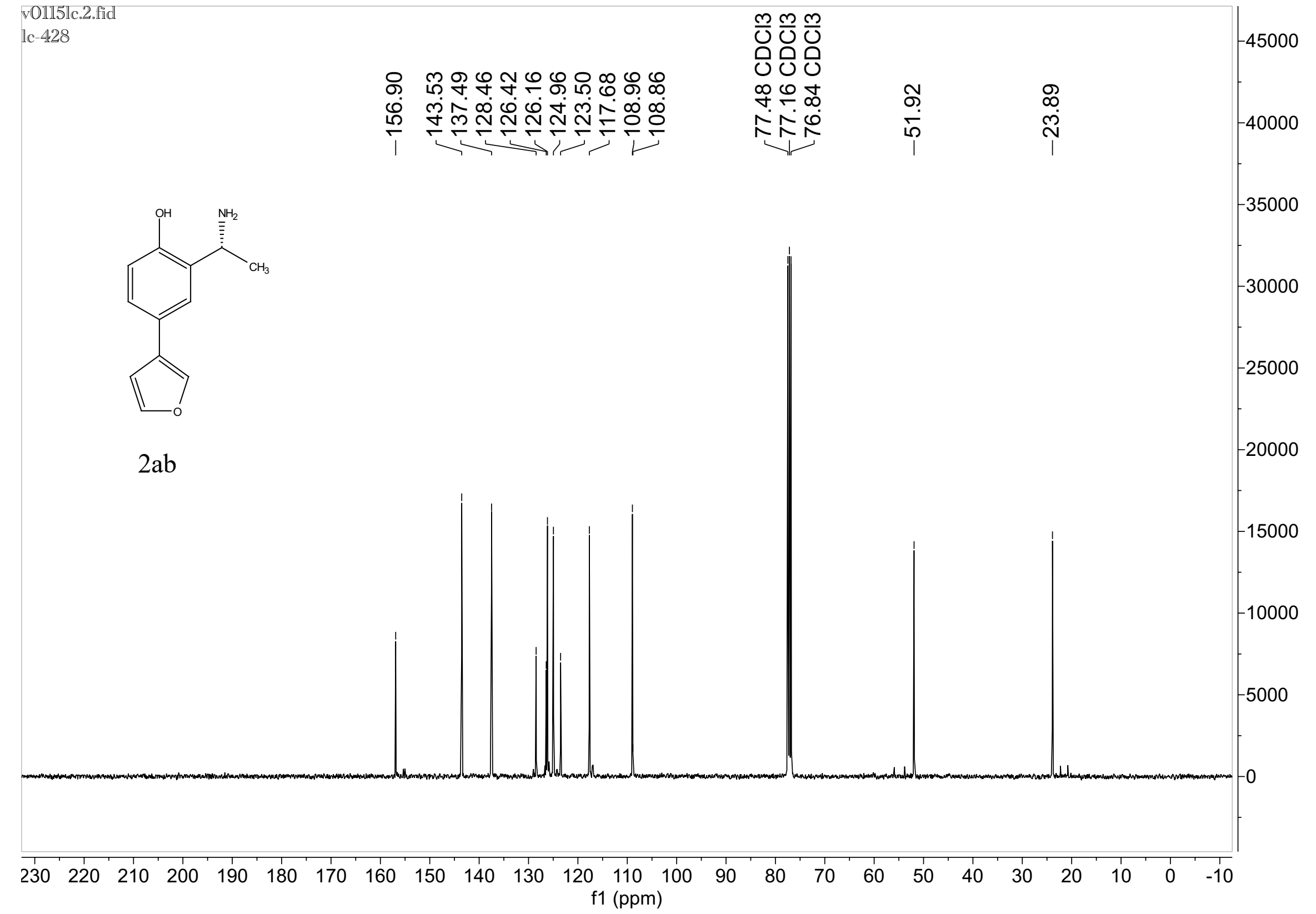
v0115lc.2.fid
lc-428
156.90
143.53 137.49 128.46 126.42 126.16 124.96 123.50 117.68 108.96 108.86
77.48 CDCl3 77.16 CDCl3 76.84 CDCl3
51.92
23.89
OH
NH_2
CH_3
O
2ab
f1 (ppm)

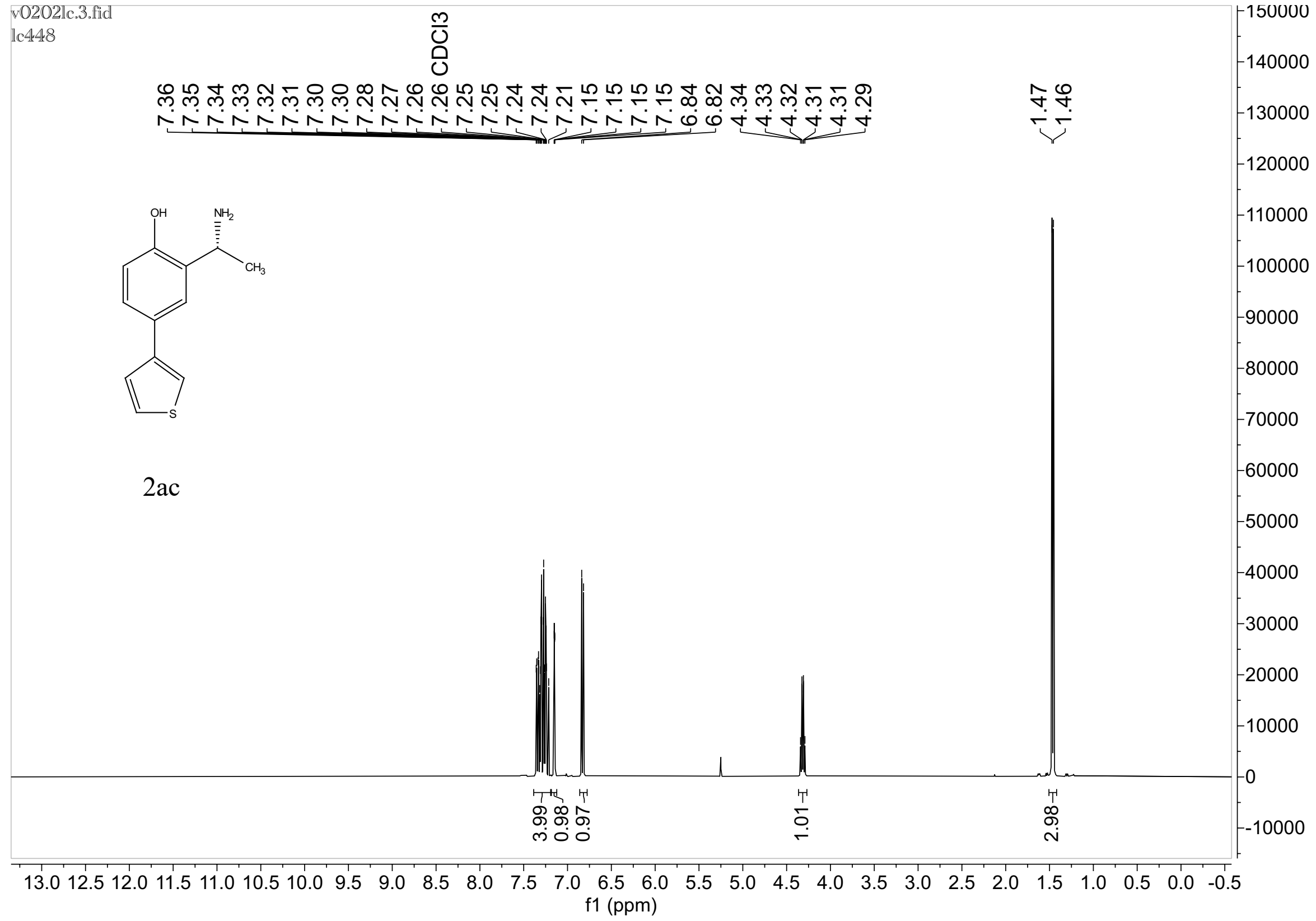
v0202lc.3.fid
lc448
7.36
7.35
7.34
7.33
7.32
7.31
7.30
7.30
7.28
7.27
7.26
7.26 CDCl3
7.25
7.25
7.24
7.24
7.21
7.15
7.15
7.15
7.15
6.84
6.82
4.34
4.33
4.32
4.31
4.31
4.29
1.47
1.46
OH
NH2
CH3
S
2ac
3.99
0.98
0.97
1.01
2.98
f1 (ppm)

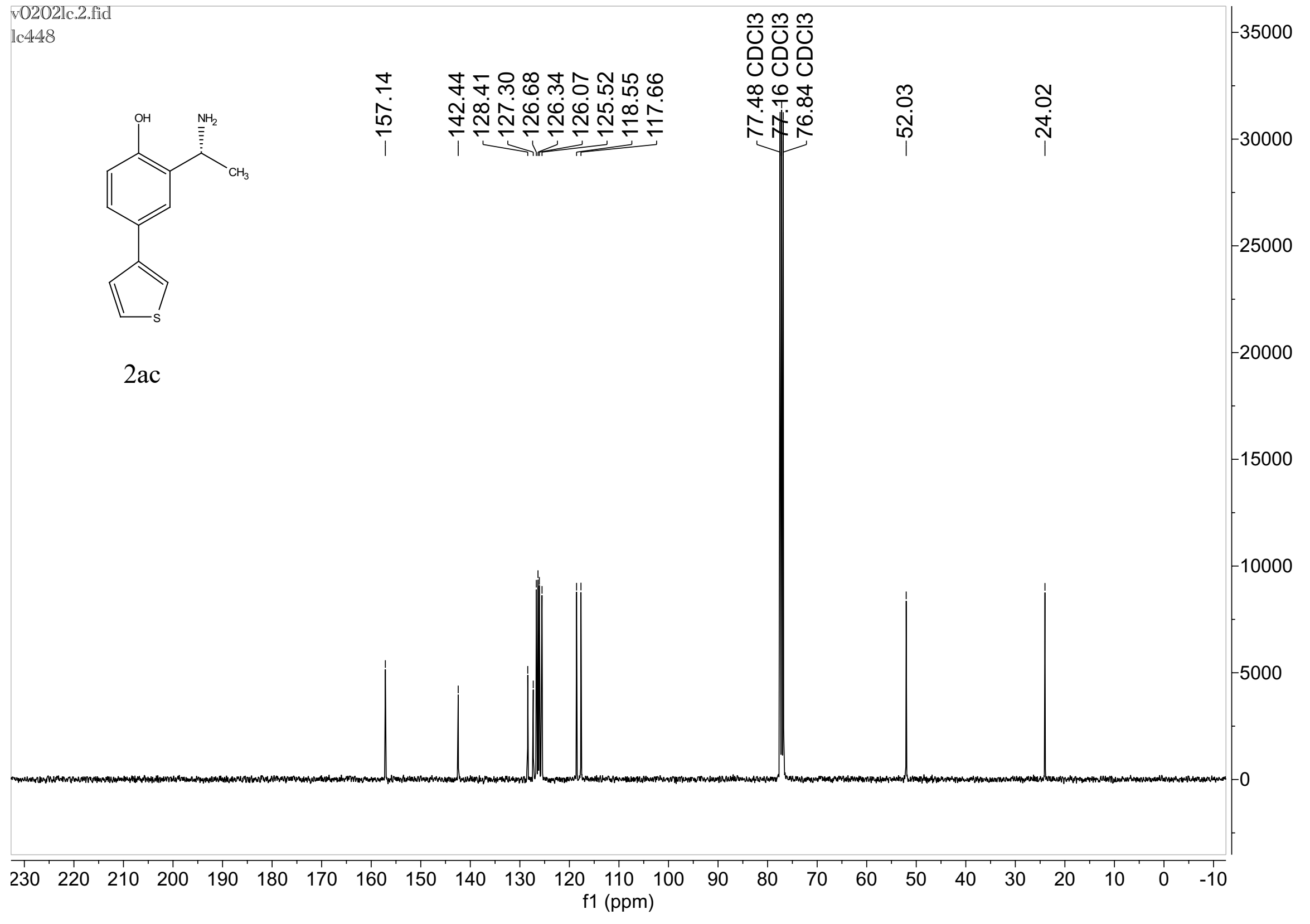
v0202lc.2.fid
lc448
157.14
142.44
128.41
127.30
126.68
126.34
126.07
125.52
118.55
117.66
77.48 CDCl3
77.16 CDCl3
76.84 CDCl3
52.03
24.02
OH
NH2
CH3
S
2ac
f1 (ppm)

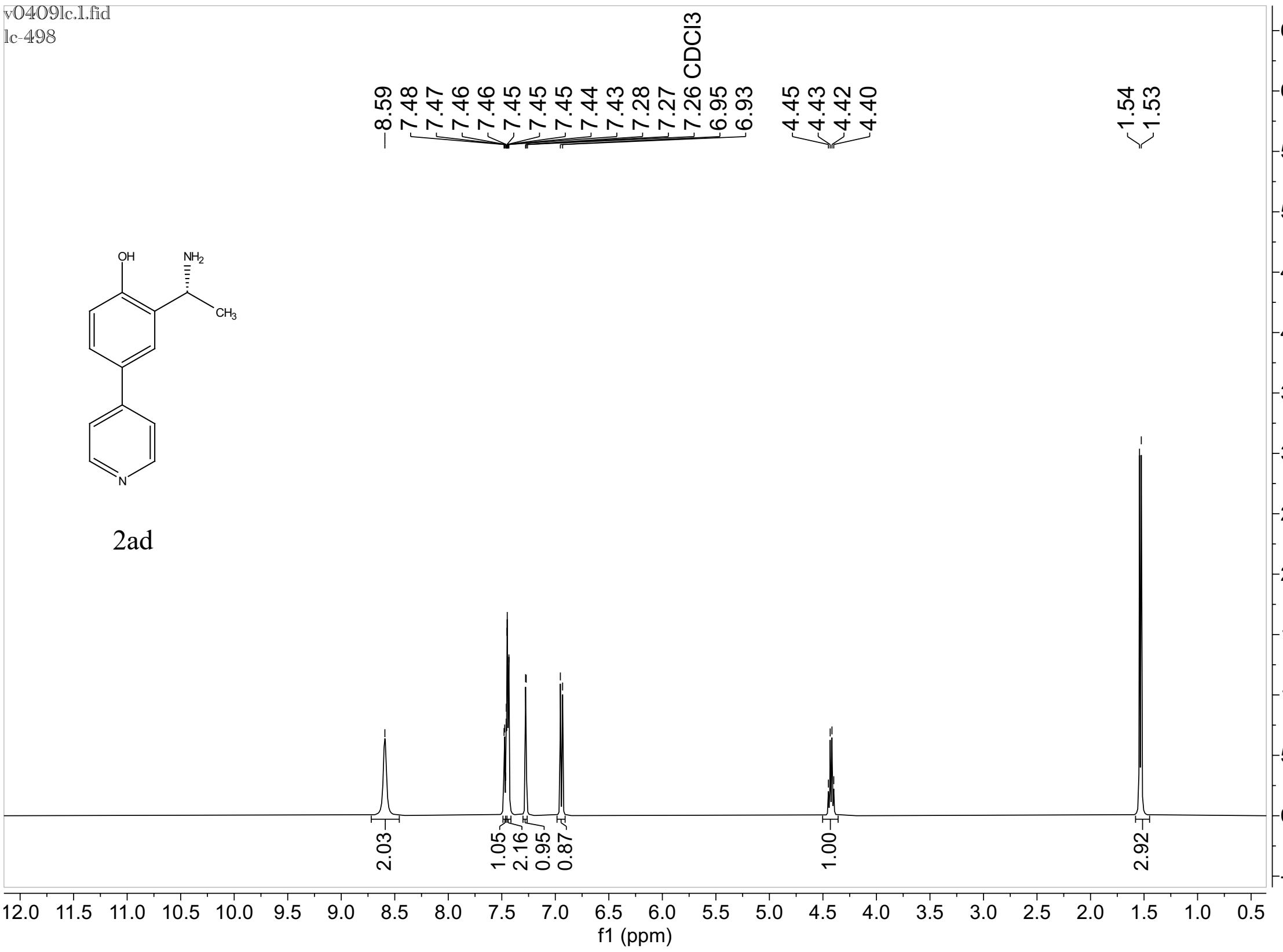
v0409lc.1.fid
lc-498
2ad
8.59
7.48
7.47
7.46
7.46
7.45
7.45
7.45
7.44
7.43
7.28
7.27
7.26 CDCl3
6.95
6.93
4.45
4.43
4.42
4.40
1.54
1.53
2.03
1.05
2.16
0.95
0.87
1.00
2.92
f1 (ppm)

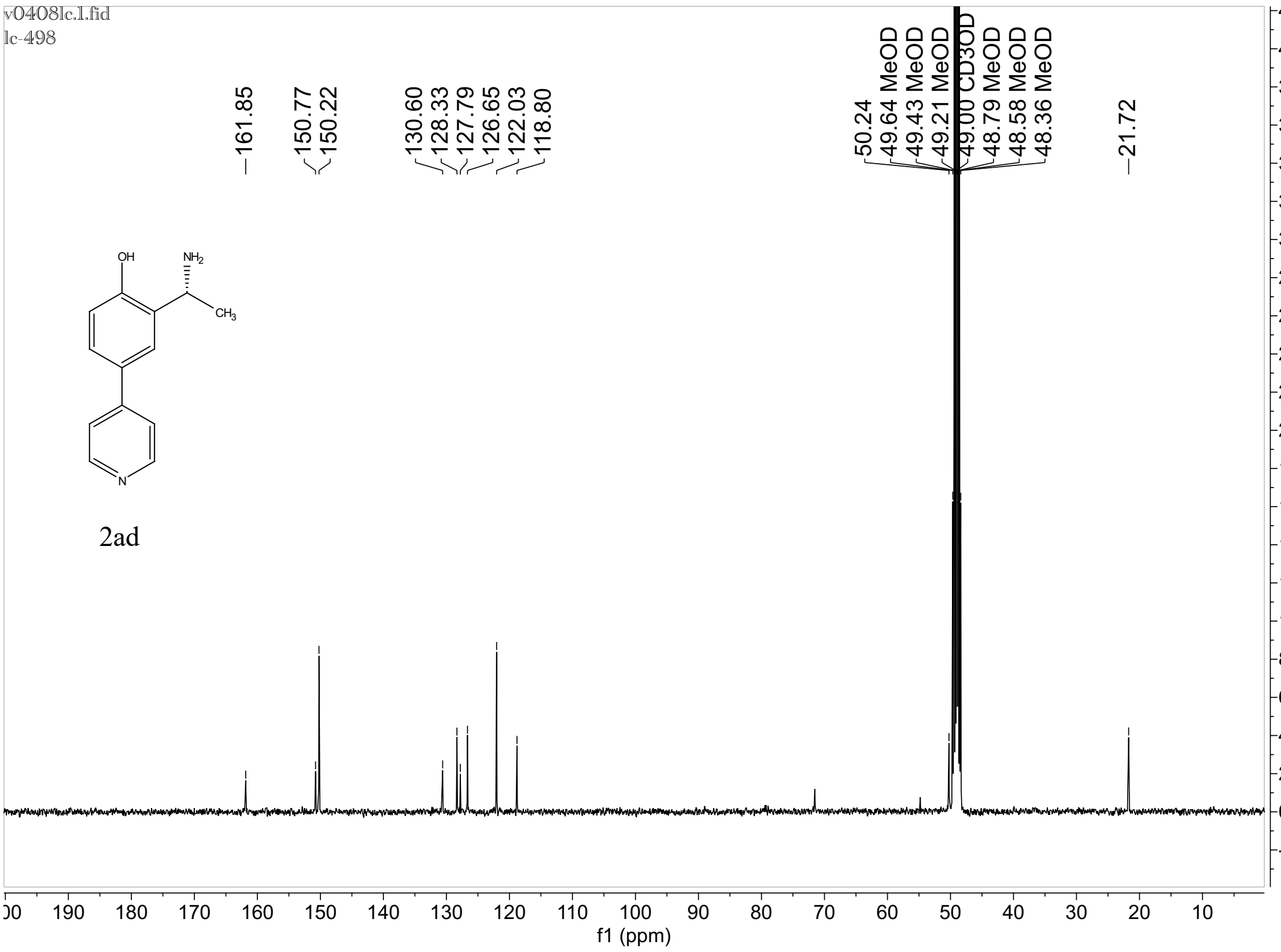
v0408lc.1.fid
lc-498
2ad
161.85
150.77
150.22
130.60
128.33
127.79
126.65
122.03
118.80
50.24
49.64 MeOD
49.43 MeOD
49.21 MeOD
49.00 CD3OD
48.79 MeOD
48.58 MeOD
48.36 MeOD
21.72
f1 (ppm)

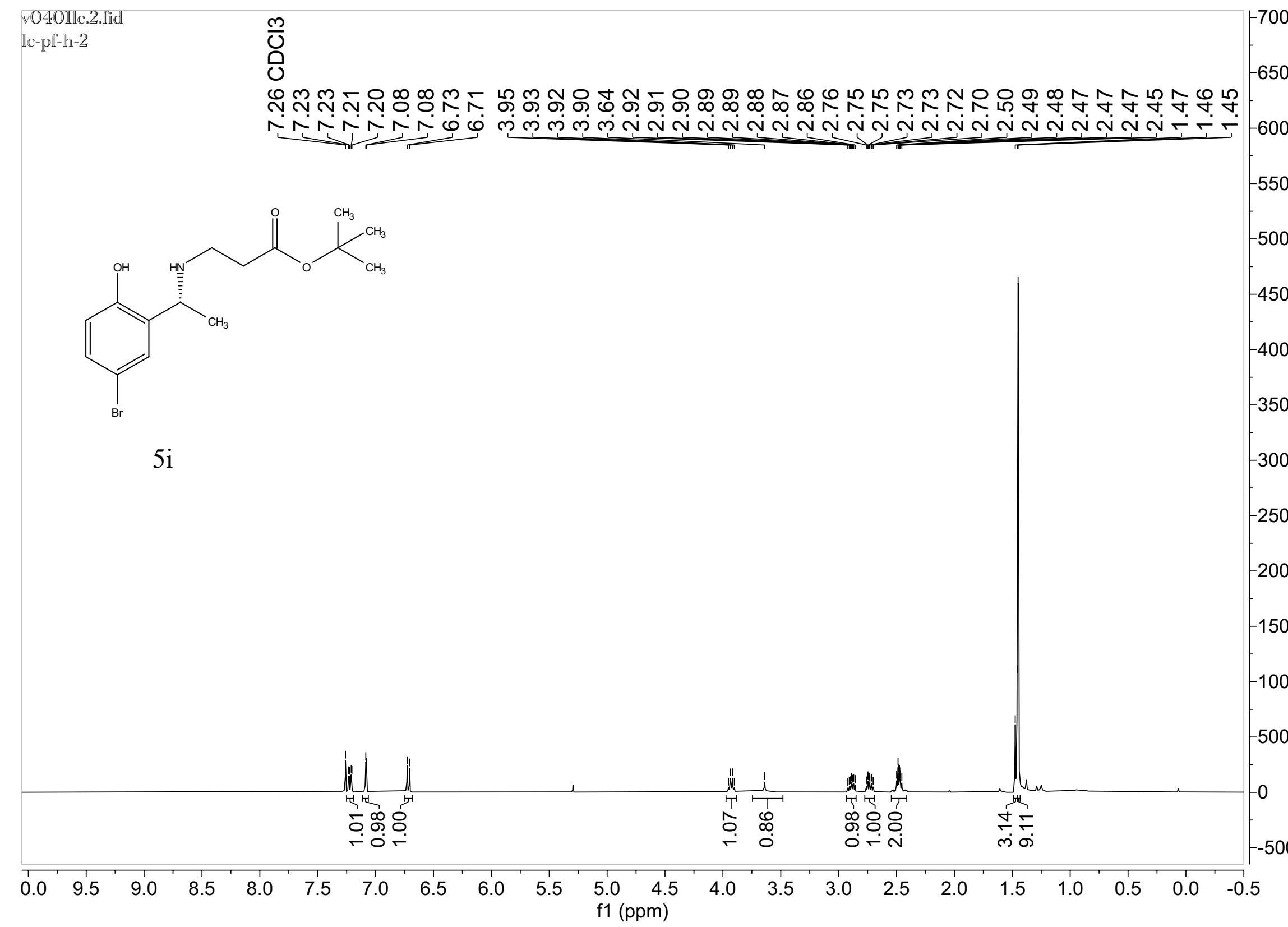

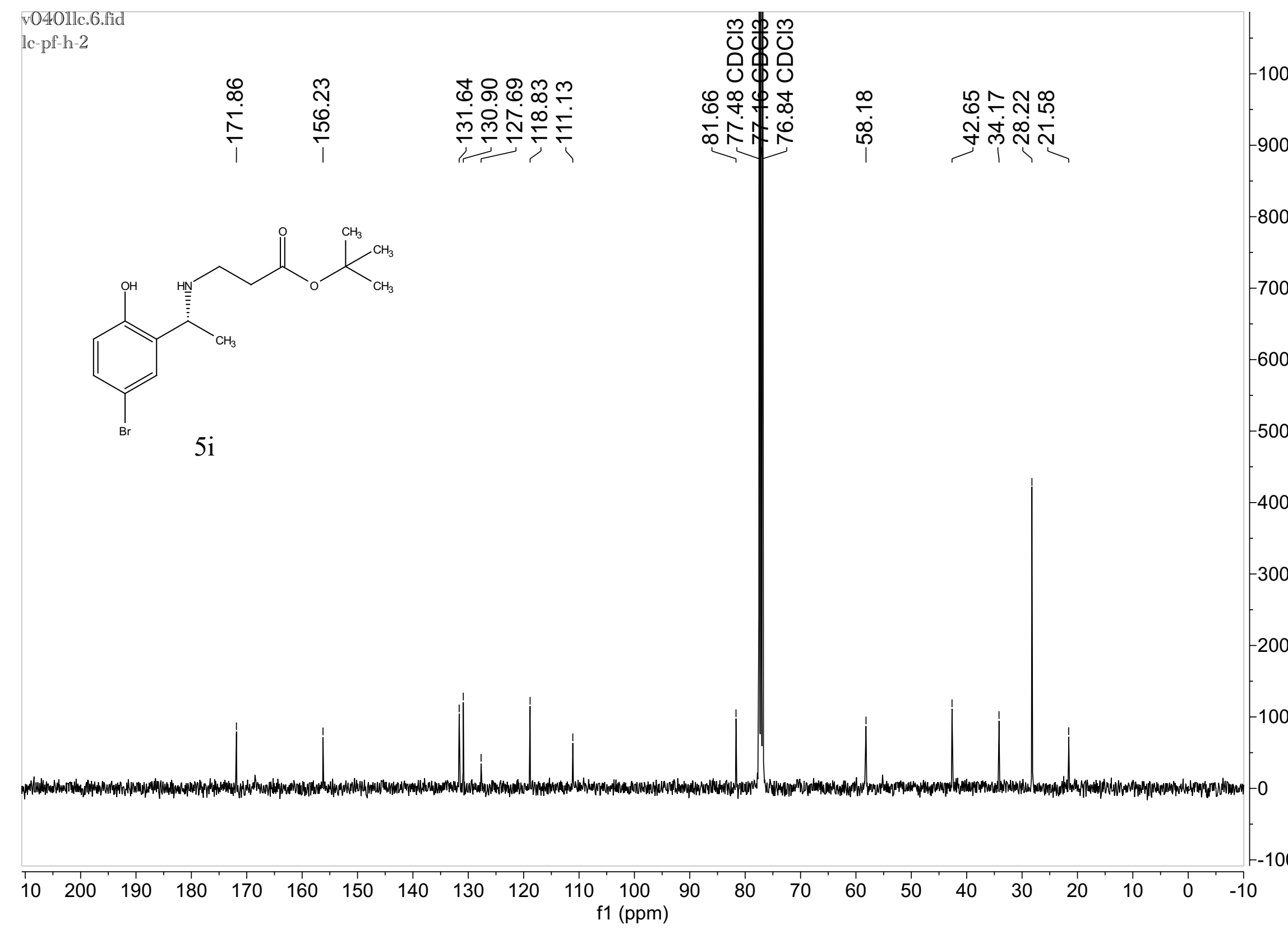

VI.2 HPLC and SFC chromatograms for compounds 2a-2ad after diacetylation

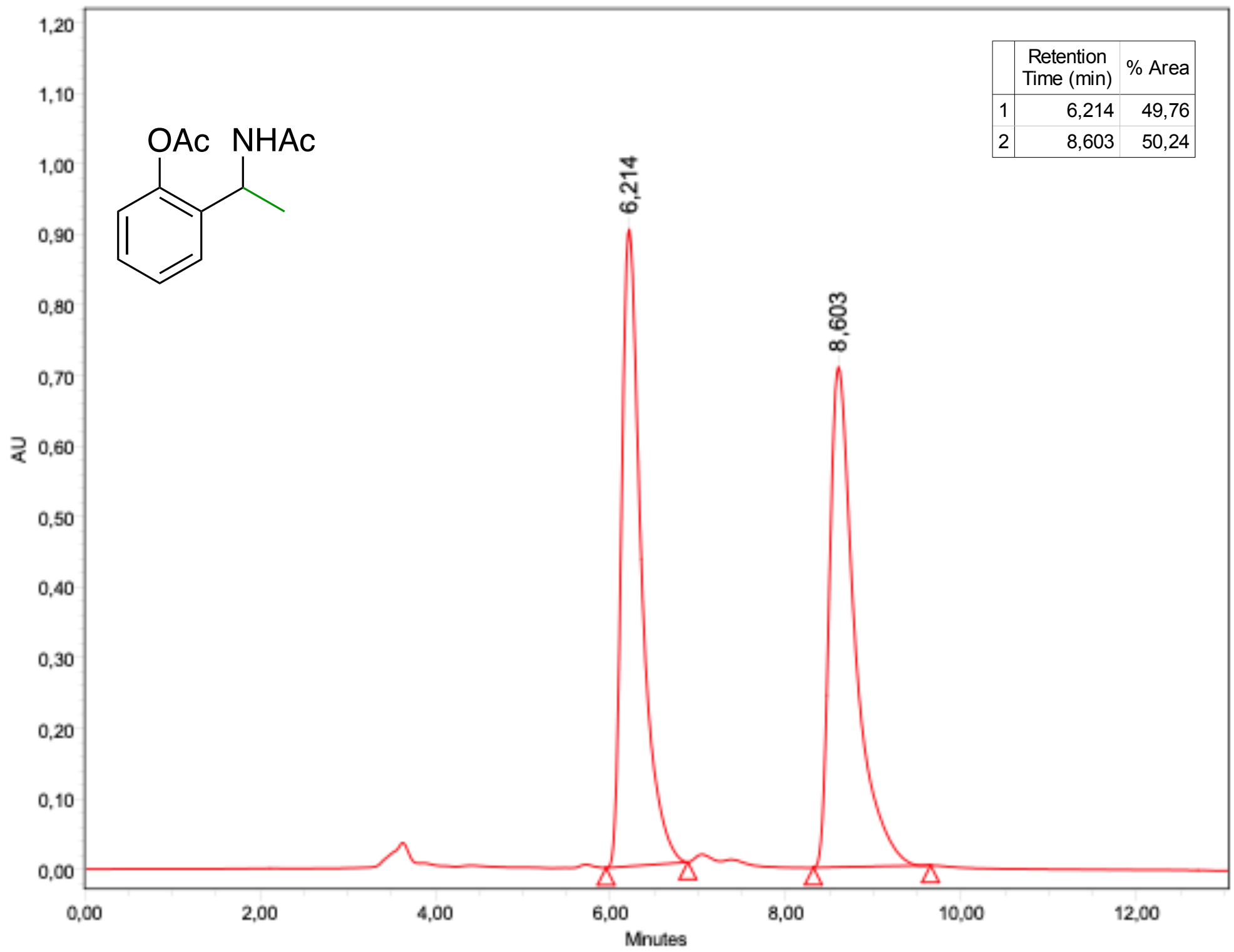

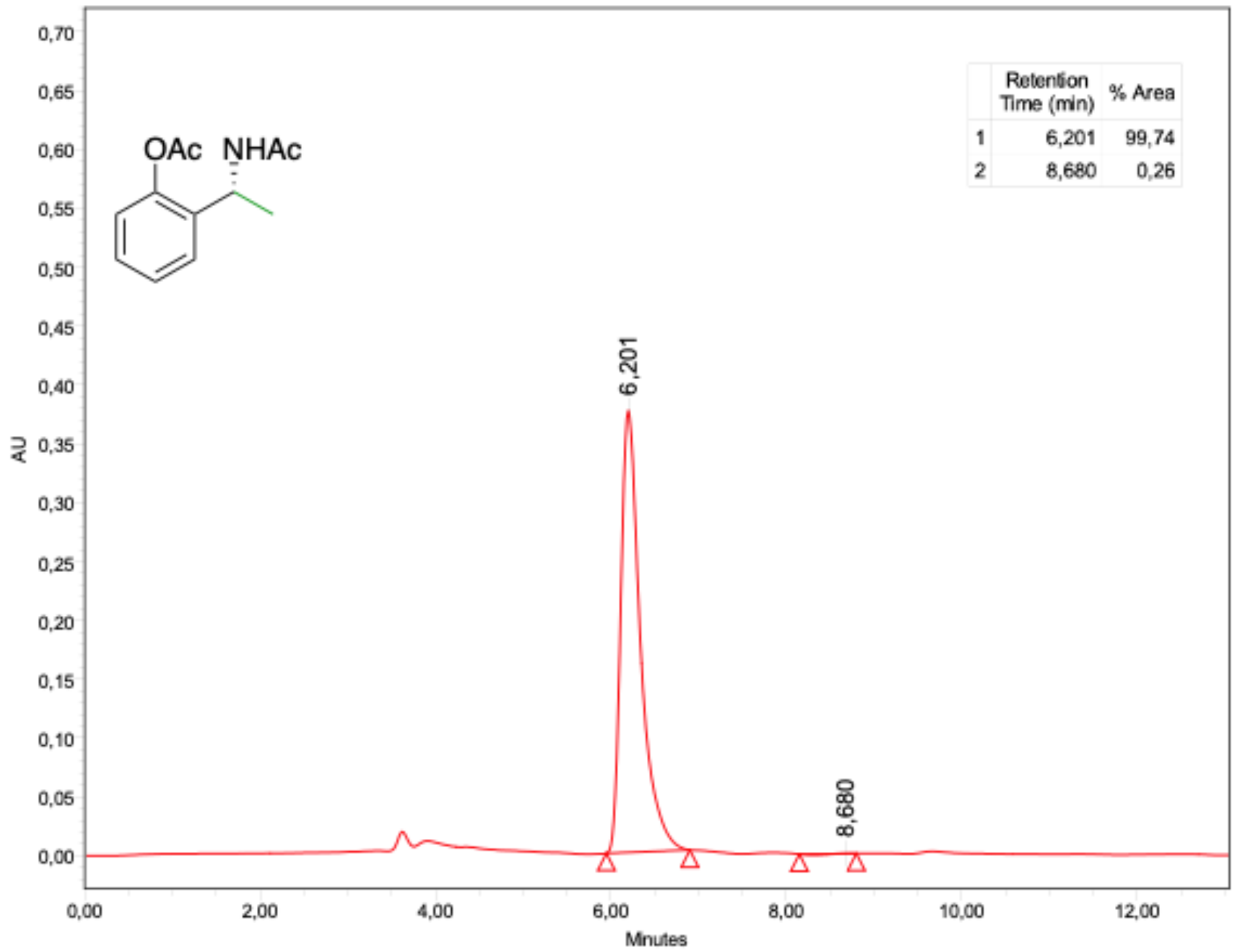

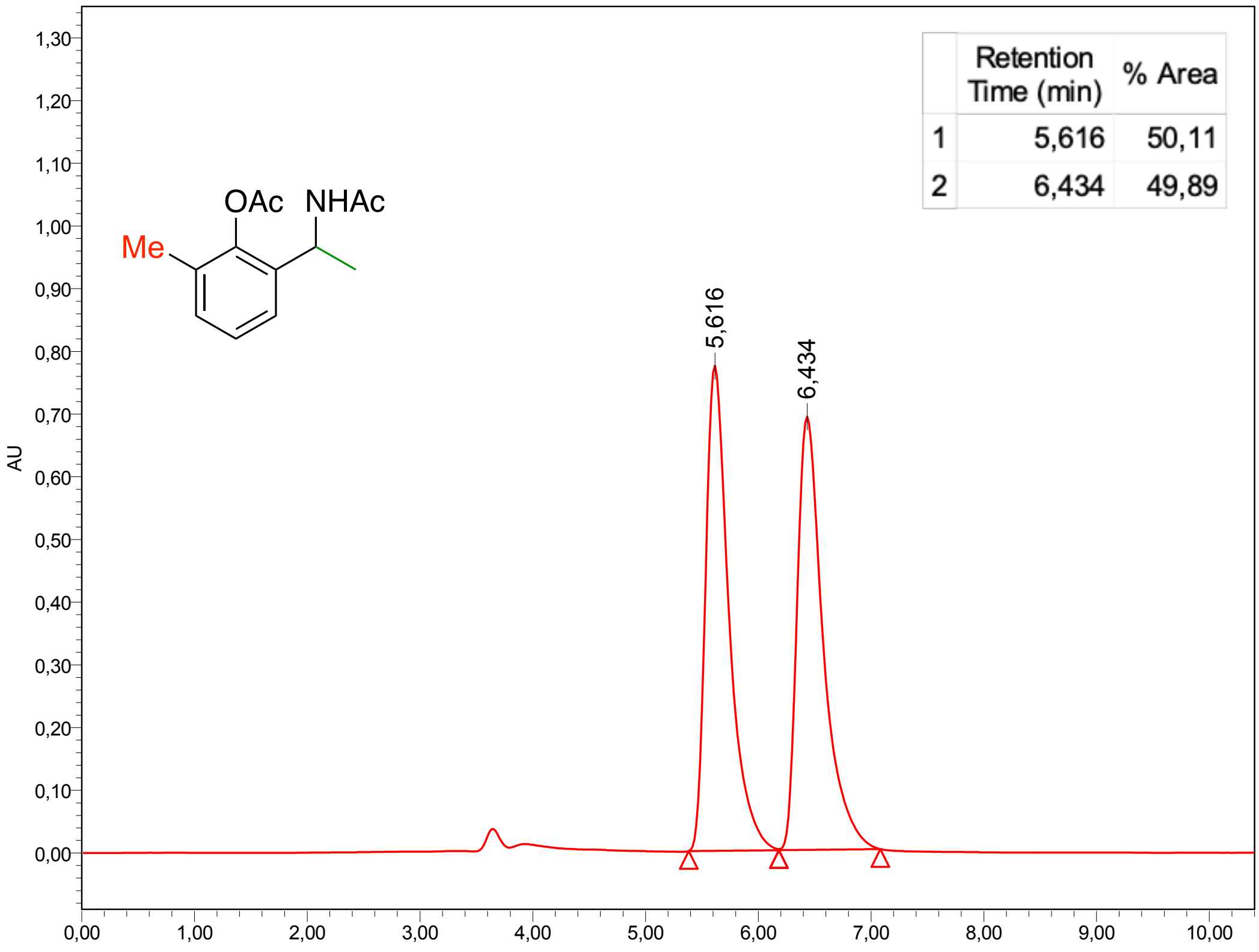

	Retention Time (min)	% Area
1	5,616	50,11
2	6,434	49,89

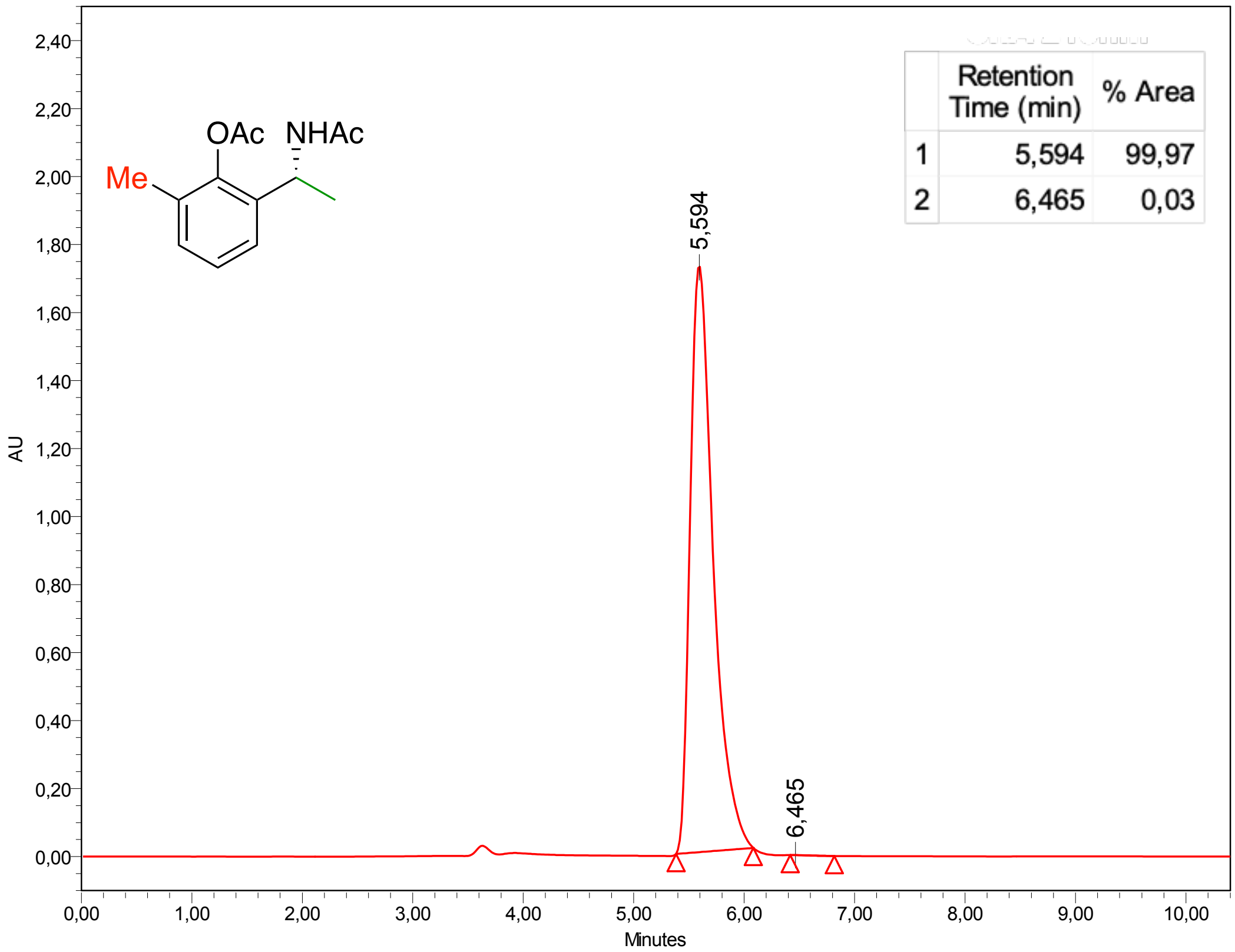

	Retention Time (min)	% Area
1	5,594	99,97
2	6,465	0,03

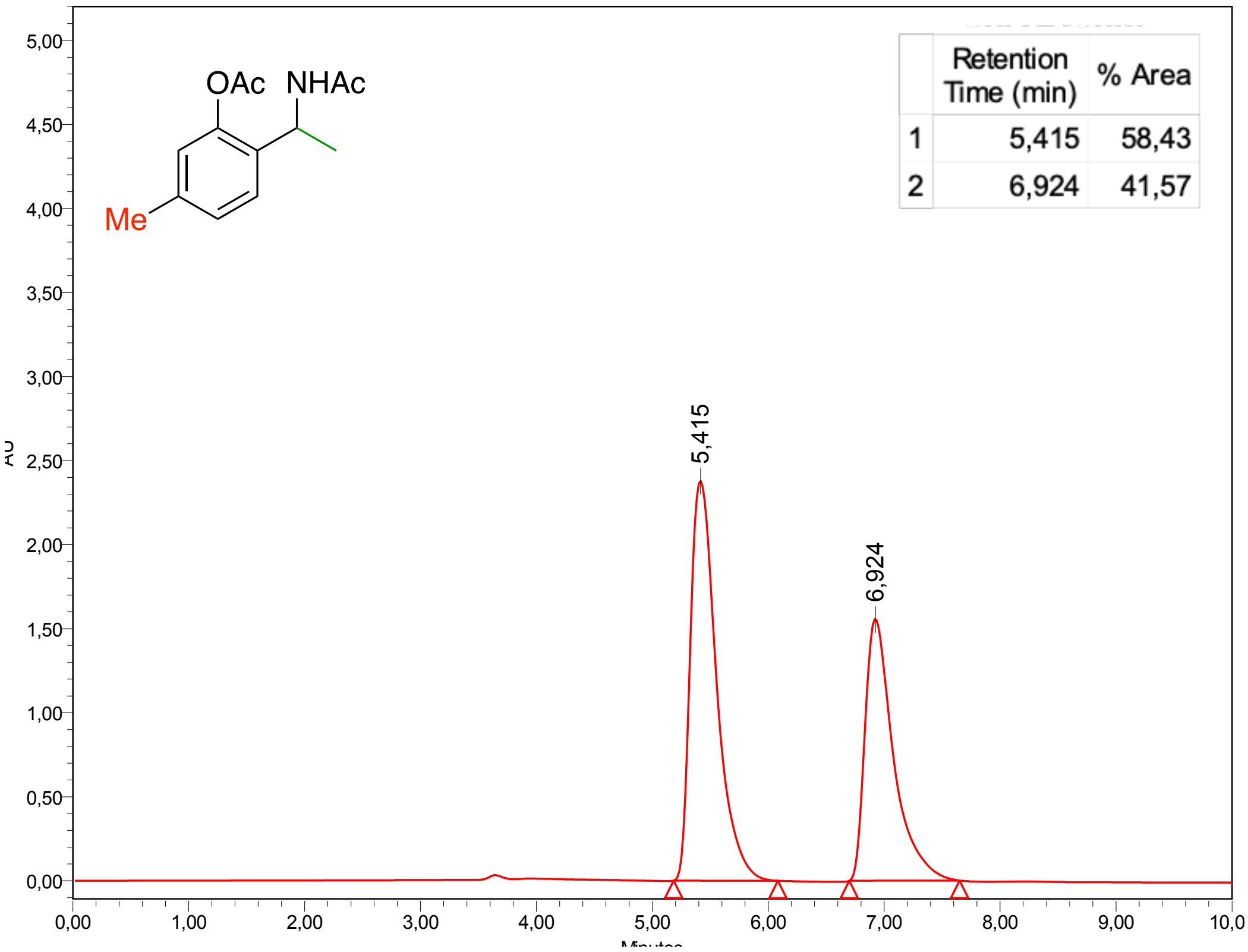
OAc NHAc
Me
Retention Time (min) | % Area
1 5,415 58,43
2 6,924 41,57
5,415
6,924
AU
0,00
0,50
1,00
1,50
2,00
2,50
3,00
3,50
4,00
4,50
5,00
0,00
1,00
2,00
3,00
4,00
5,00
6,00
7,00
8,00
9,00

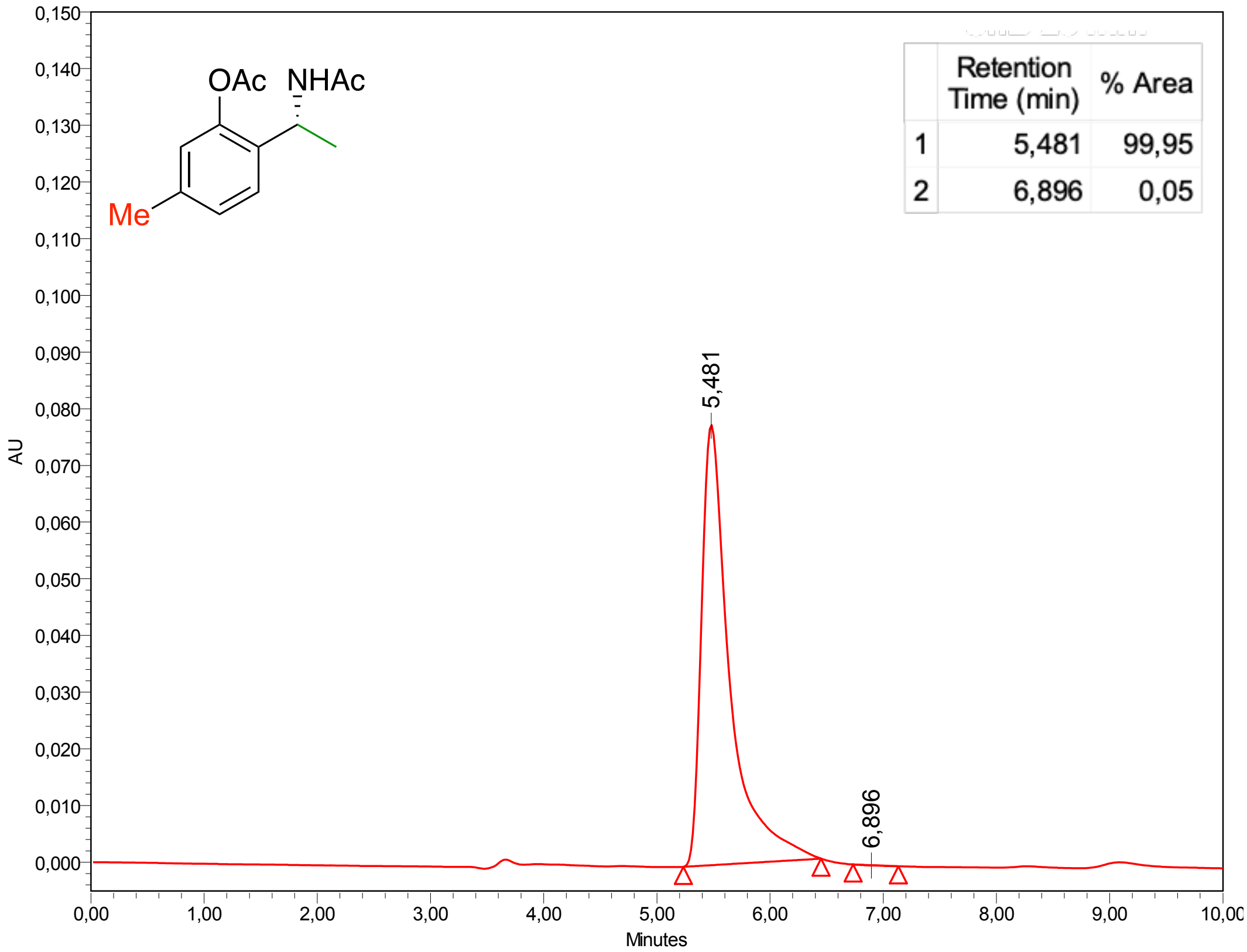
OAc NHAc
Me
Retention Time (min) | % Area
1 5,481 99,95
2 6,896 0,05
5,481
6,896
AU
Minutes
0,000
0,010
0,020
0,030
0,040
0,050
0,060
0,070
0,080
0,090
0,100
0,110
0,120
0,130
0,140
0,150
0,00
1,00
2,00
3,00
4,00
5,00
6,00
7,00
8,00
9,00

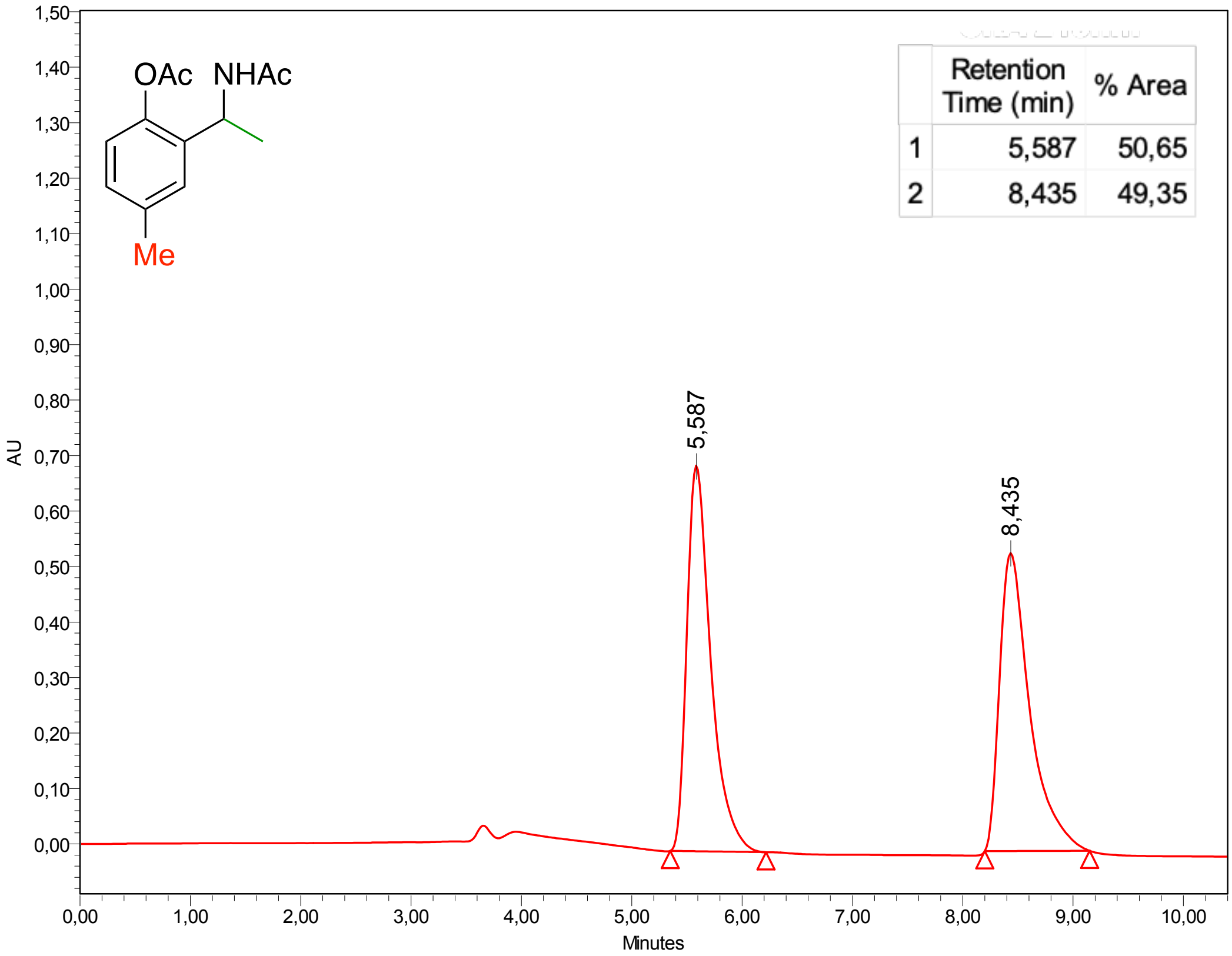
OAc NHAc
Me
Retention Time (min)
% Area
1 5,587 50,65
2 8,435 49,35
5,587
8,435
AU
Minutes
1,50
1,40
1,30
1,20
1,10
1,00
0,90
0,80
0,70
0,60
0,50
0,40
0,30
0,20
0,10
0,00
0,00 1,00 2,00 3,00 4,00 5,00 6,00 7,00 8,00 9,00 10,00

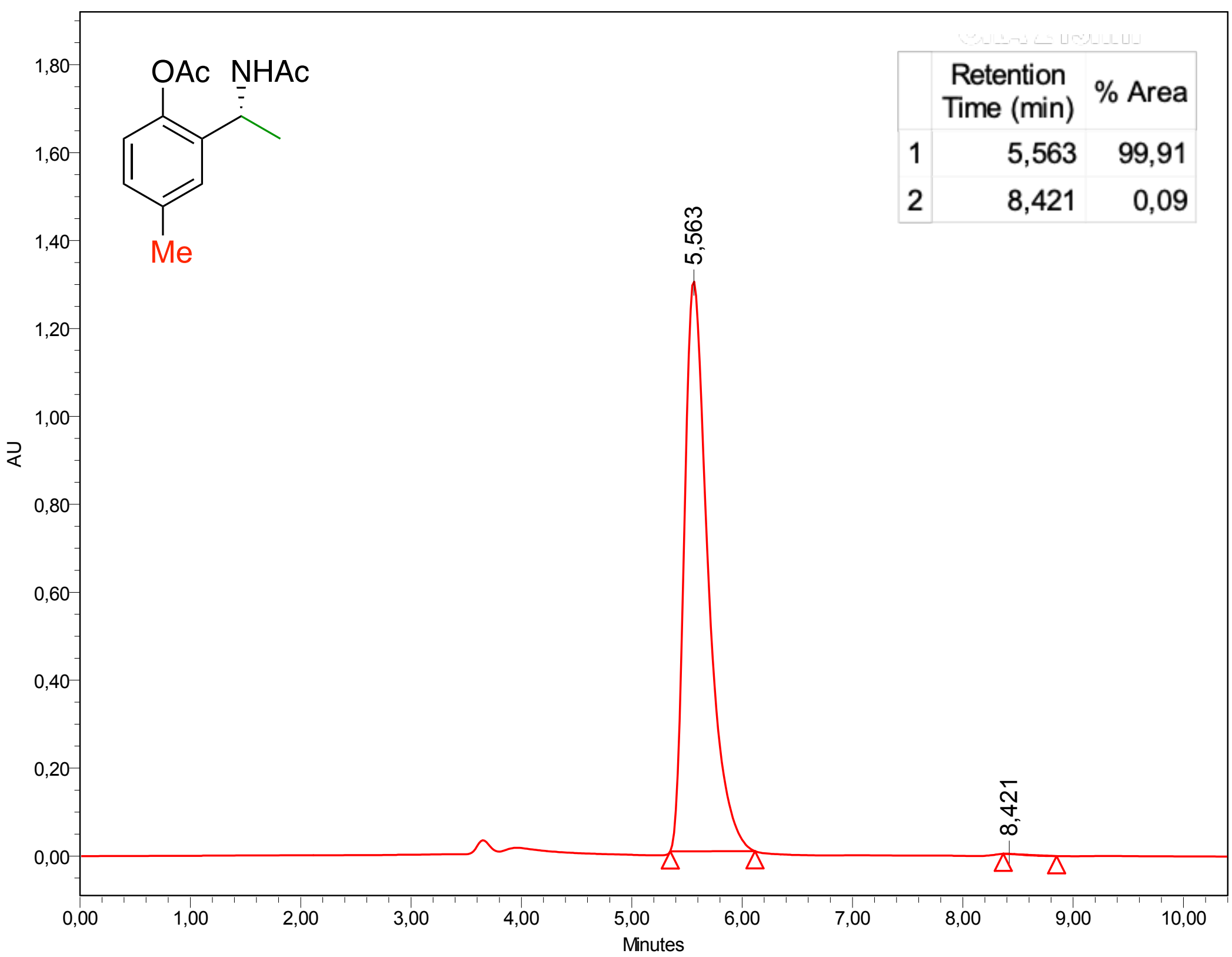
OAc NHAc
Me
Retention Time (min)
% Area
1 5,563 99,91
2 8,421 0,09
5,563
8,421
AU
Minutes
1,80
1,60
1,40
1,20
1,00
0,80
0,60
0,40
0,20
0,00
0,00 1,00 2,00 3,00 4,00 5,00 6,00 7,00 8,00 9,00 10,00

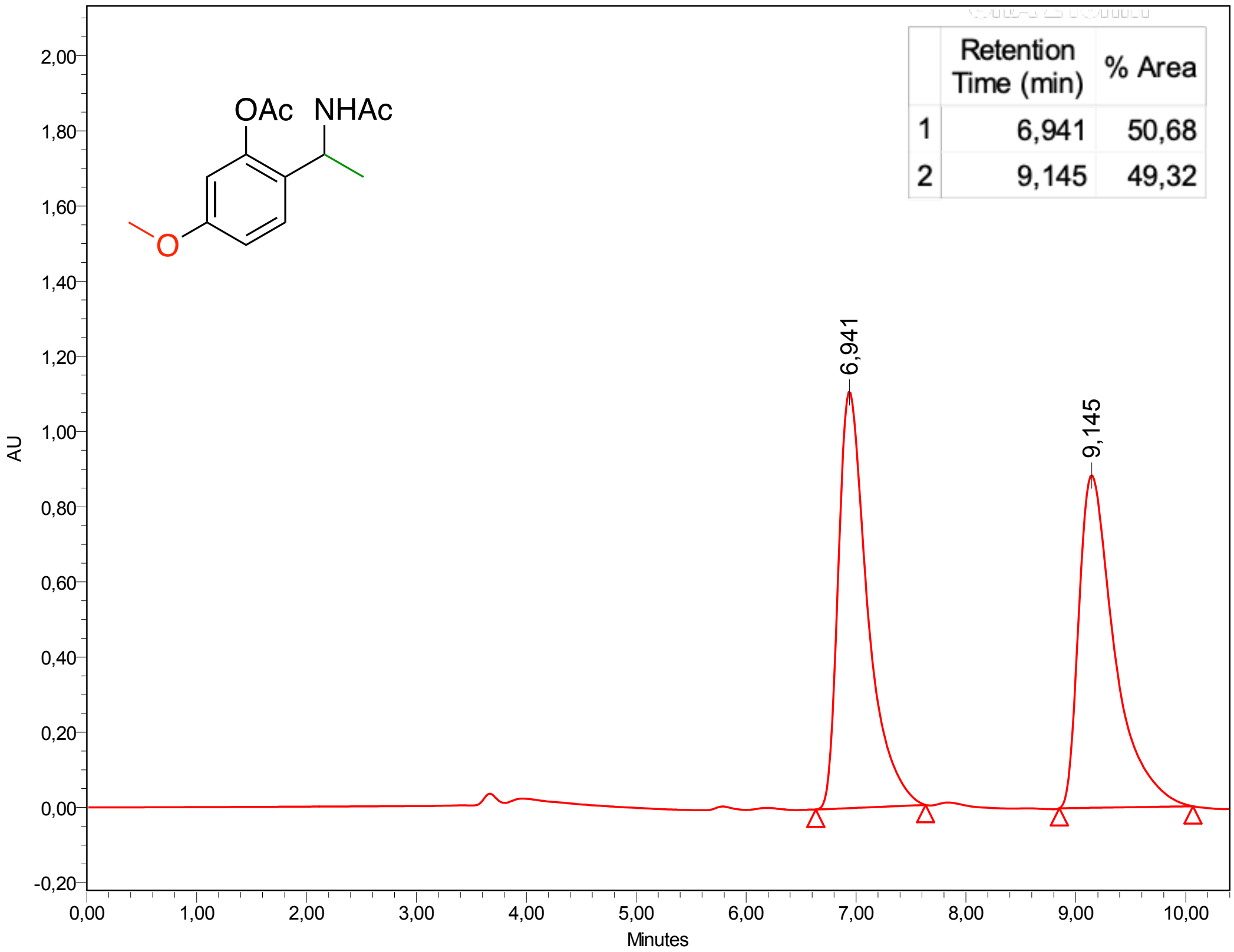
OAc NHAc
O
Retention Time (min) | % Area
1 | 6,941 | 50,68
2 | 9,145 | 49,32
6,941
9,145
AU
Minutes

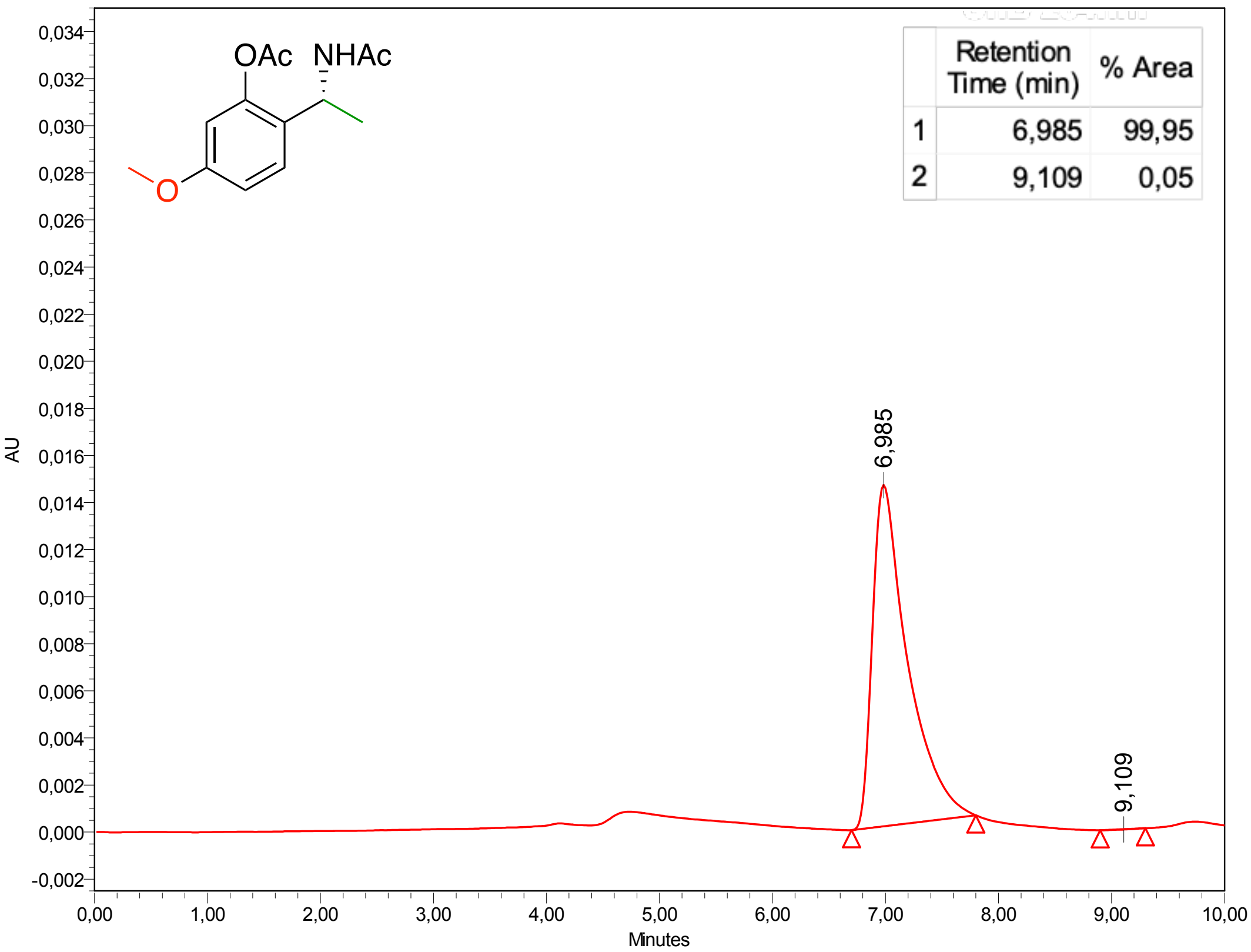
OAc NHAc
O
Retention Time (min) | % Area
1 | 6,985 | 99,95
2 | 9,109 | 0,05
6,985
9,109
AU
Minutes

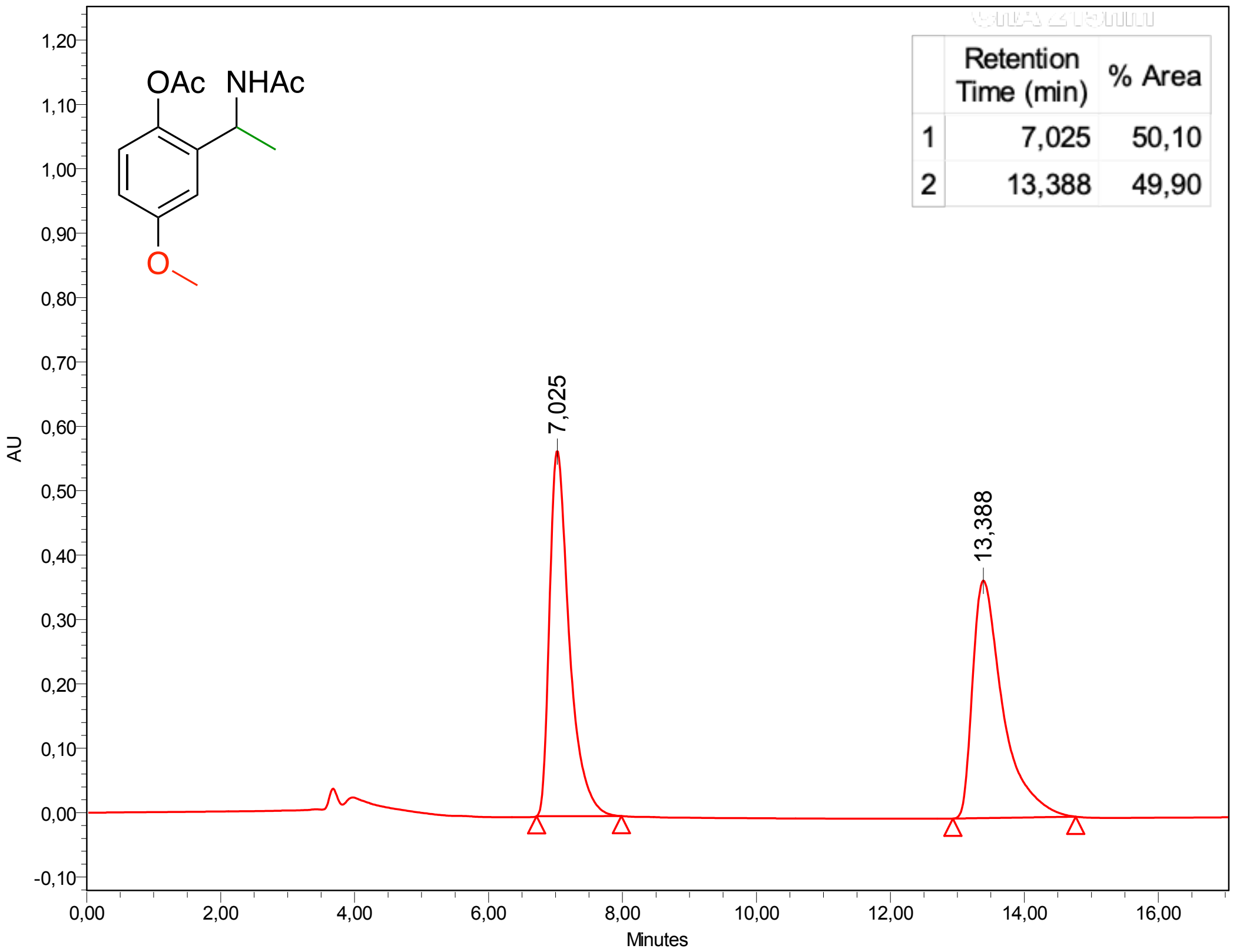
OAc NHAc
O
Retention Time (min) | % Area
1 7,025 50,10
2 13,388 49,90
7,025
13,388
AU
Minutes

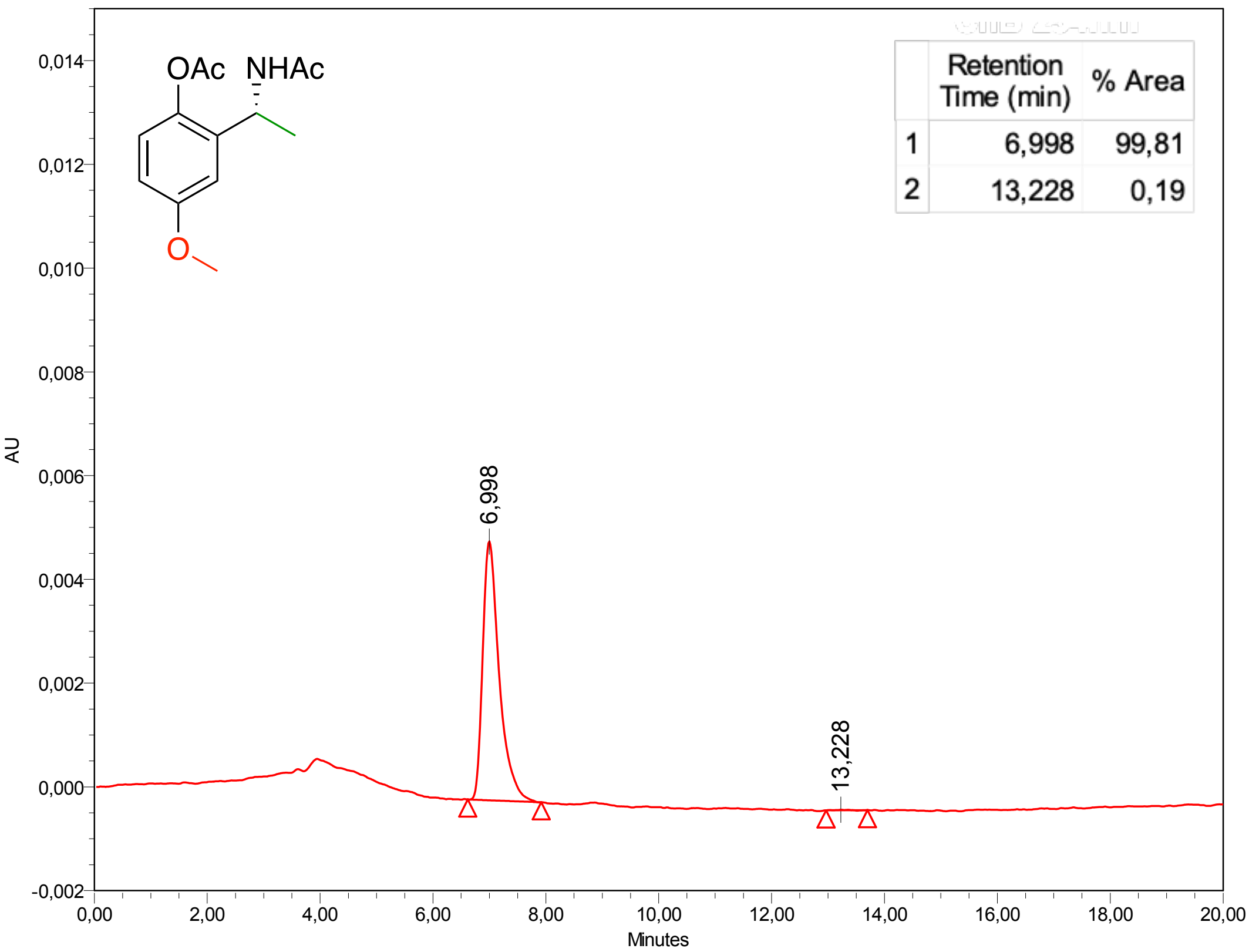
OAc NHAc
O
Retention Time (min) | % Area
1 6,998 99,81
2 13,228 0,19
6,998
13,228
AU
Minutes

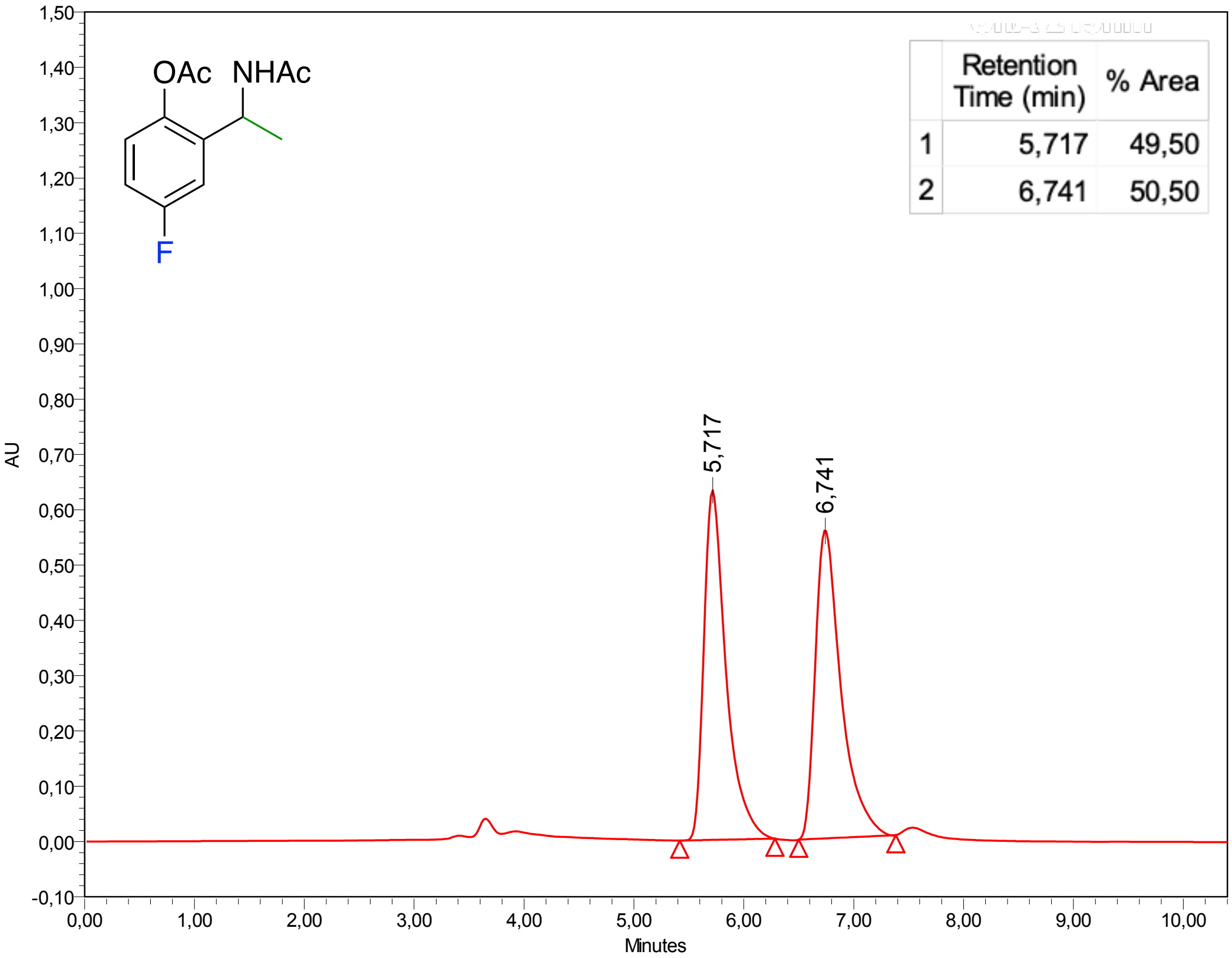

	Retention Time (min)	% Area
1	5,717	49,50
2	6,741	50,50

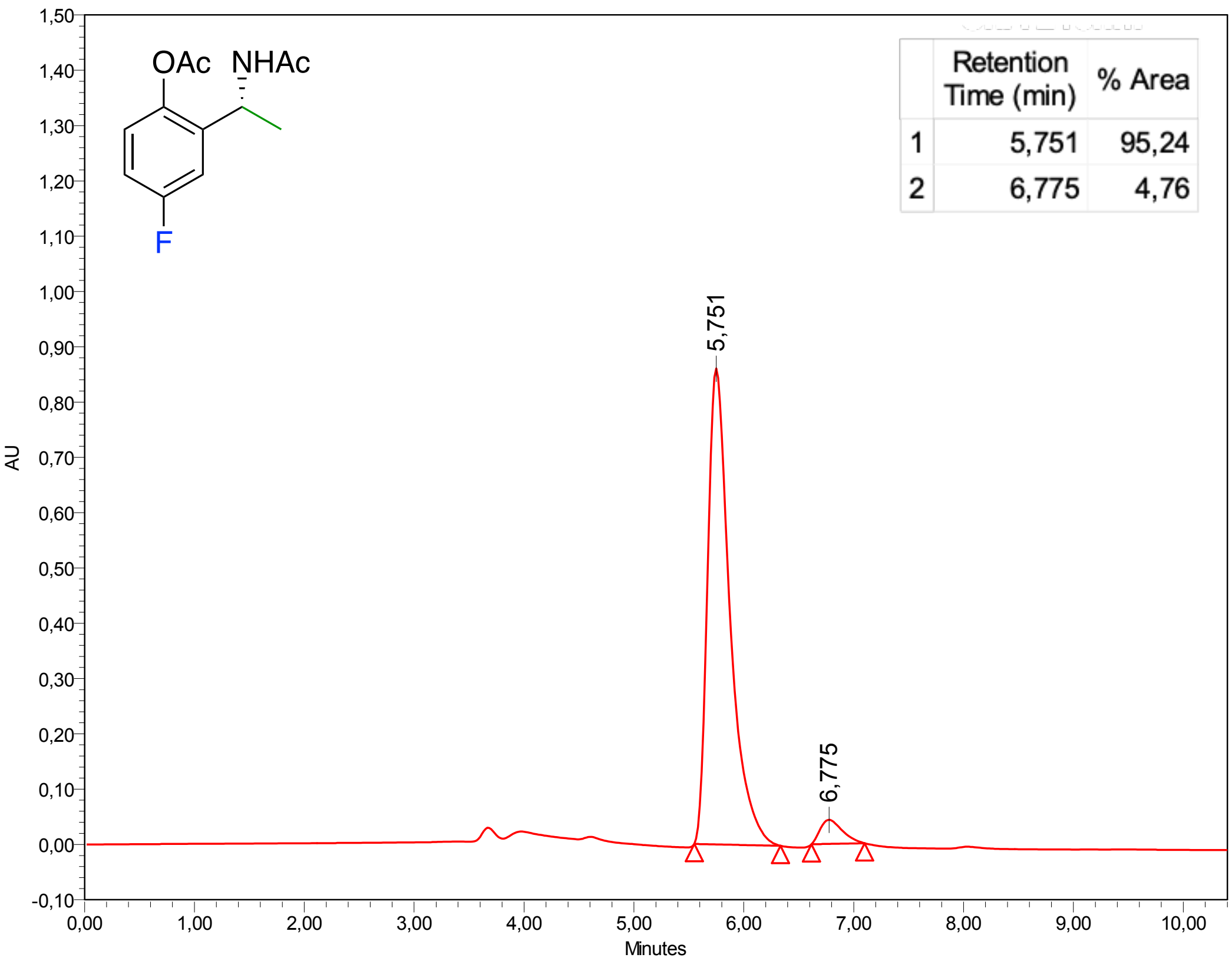

	Retention Time (min)	% Area
1	5,751	95,24
2	6,775	4,76

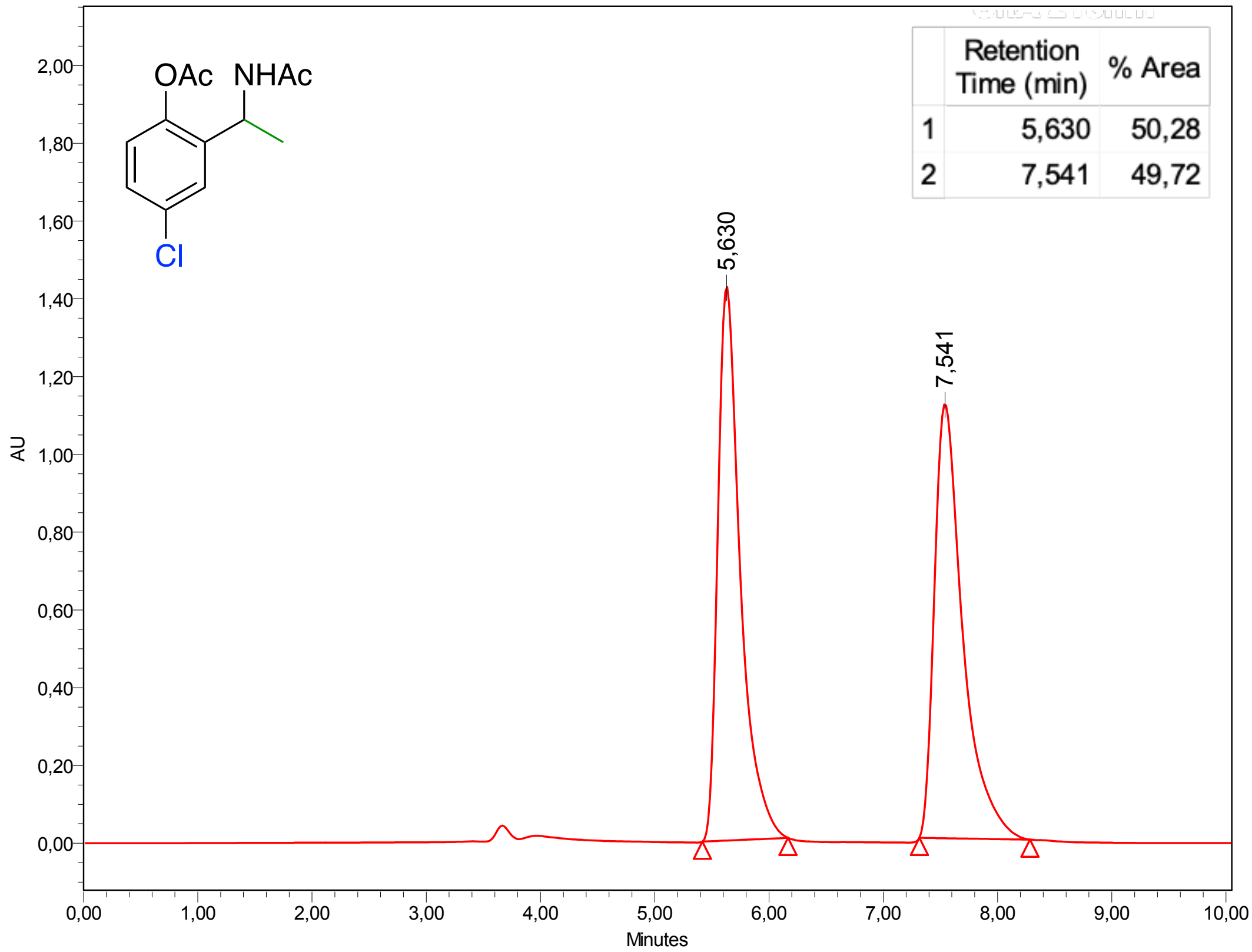
OAc NHAc
Cl
Retention Time (min) | % Area
1 5,630 50,28
2 7,541 49,72
5,630
7,541
AU
Minutes

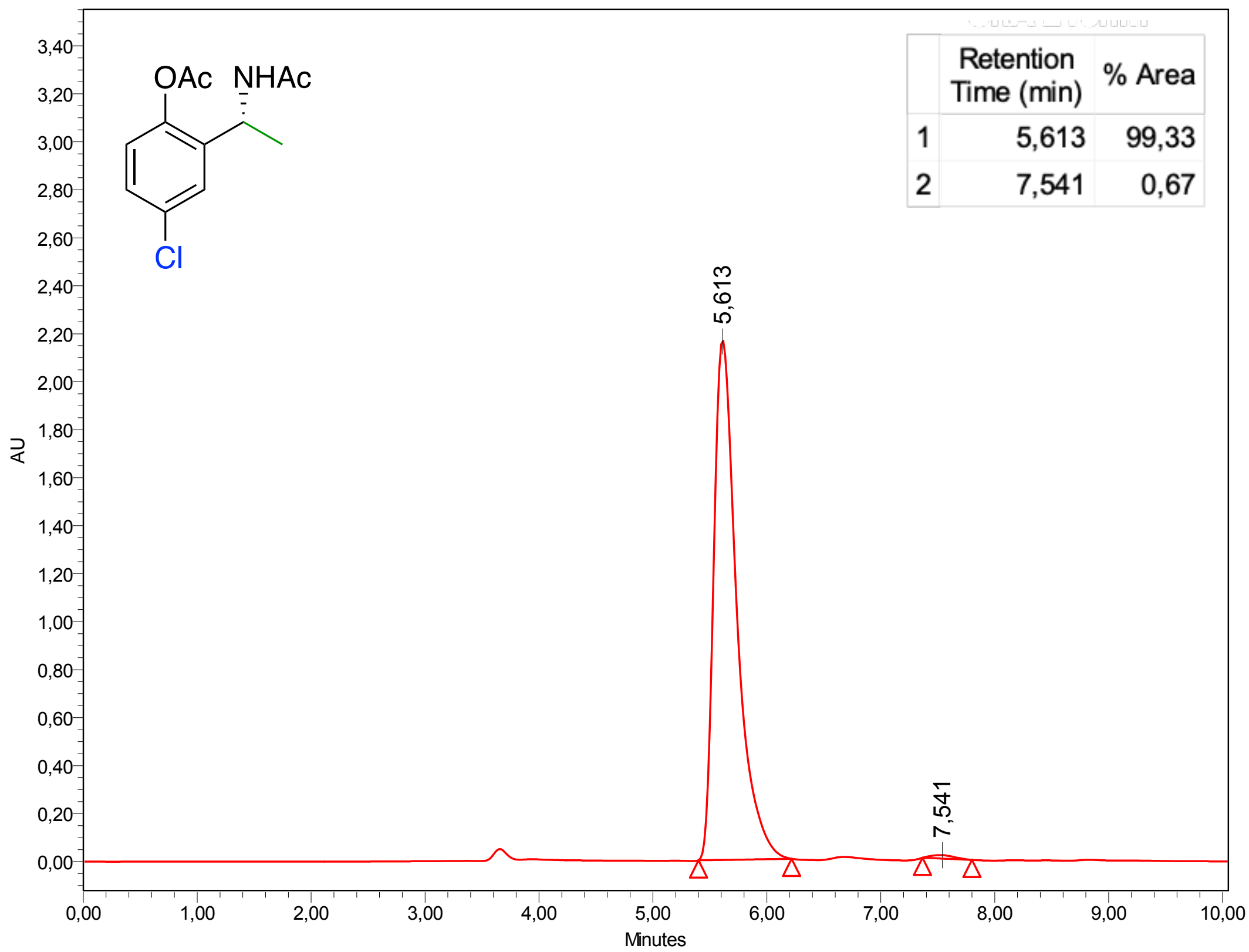
OAc NHAc
Cl
Retention Time (min) | % Area
1 5,613 99,33
2 7,541 0,67
5,613
7,541
AU
Minutes

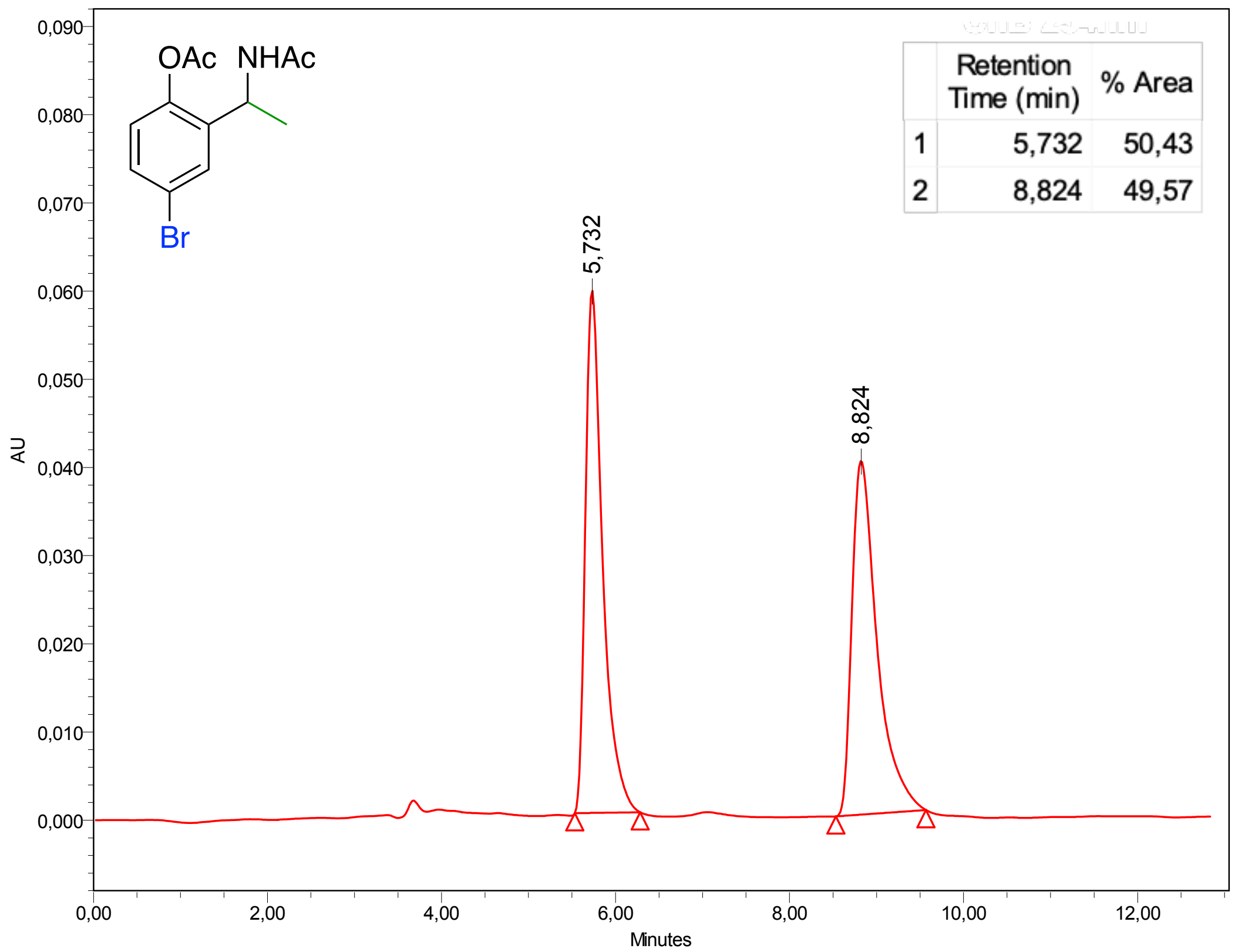
OAc NHAc
Br
Retention Time (min) | % Area
1 5,732 50,43
2 8,824 49,57
5,732
8,824
AU
Minutes

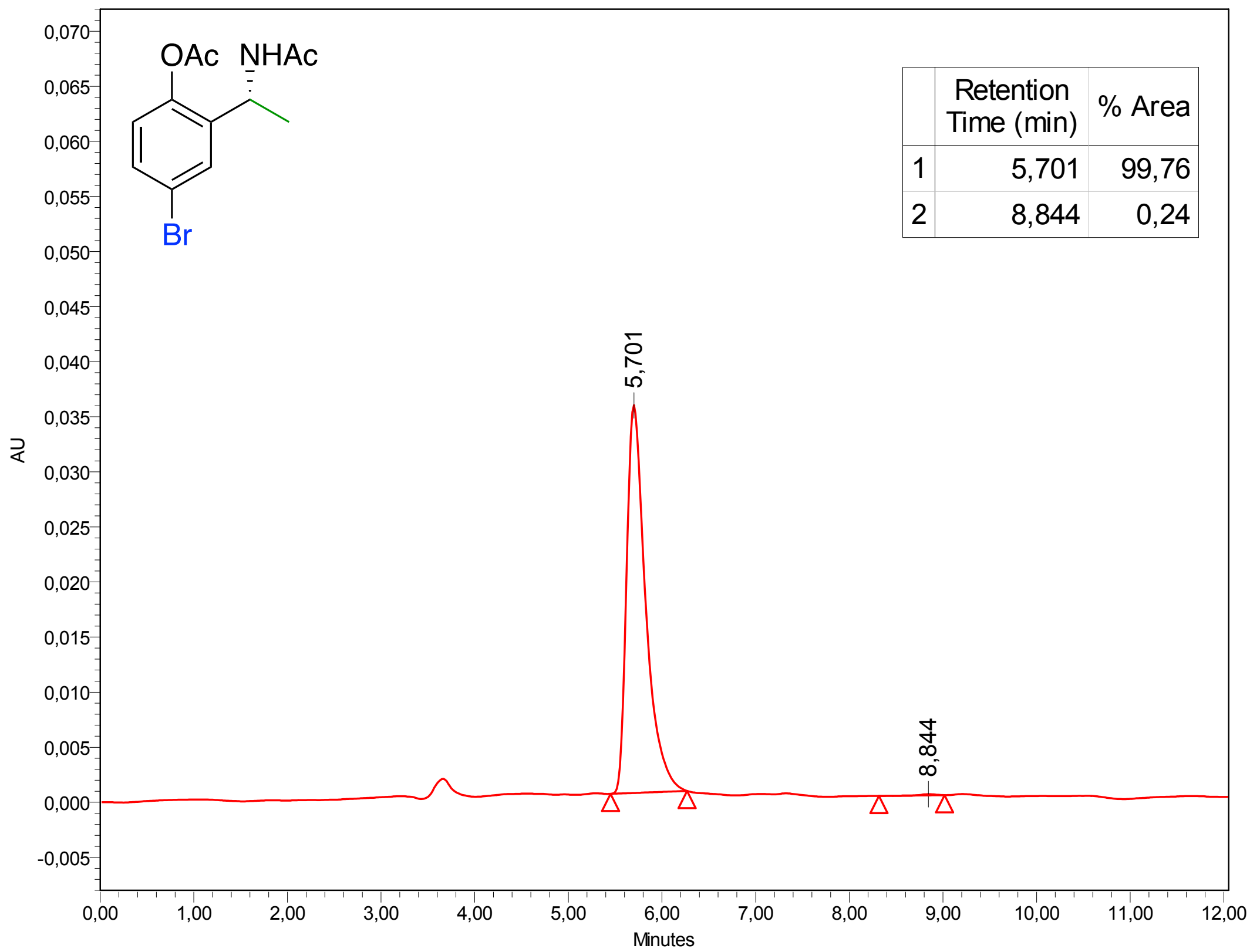
OAc NHAc
Br
Retention Time (min) | % Area
1 5,701 99,76
2 8,844 0,24
5,701
8,844
AU
Minutes

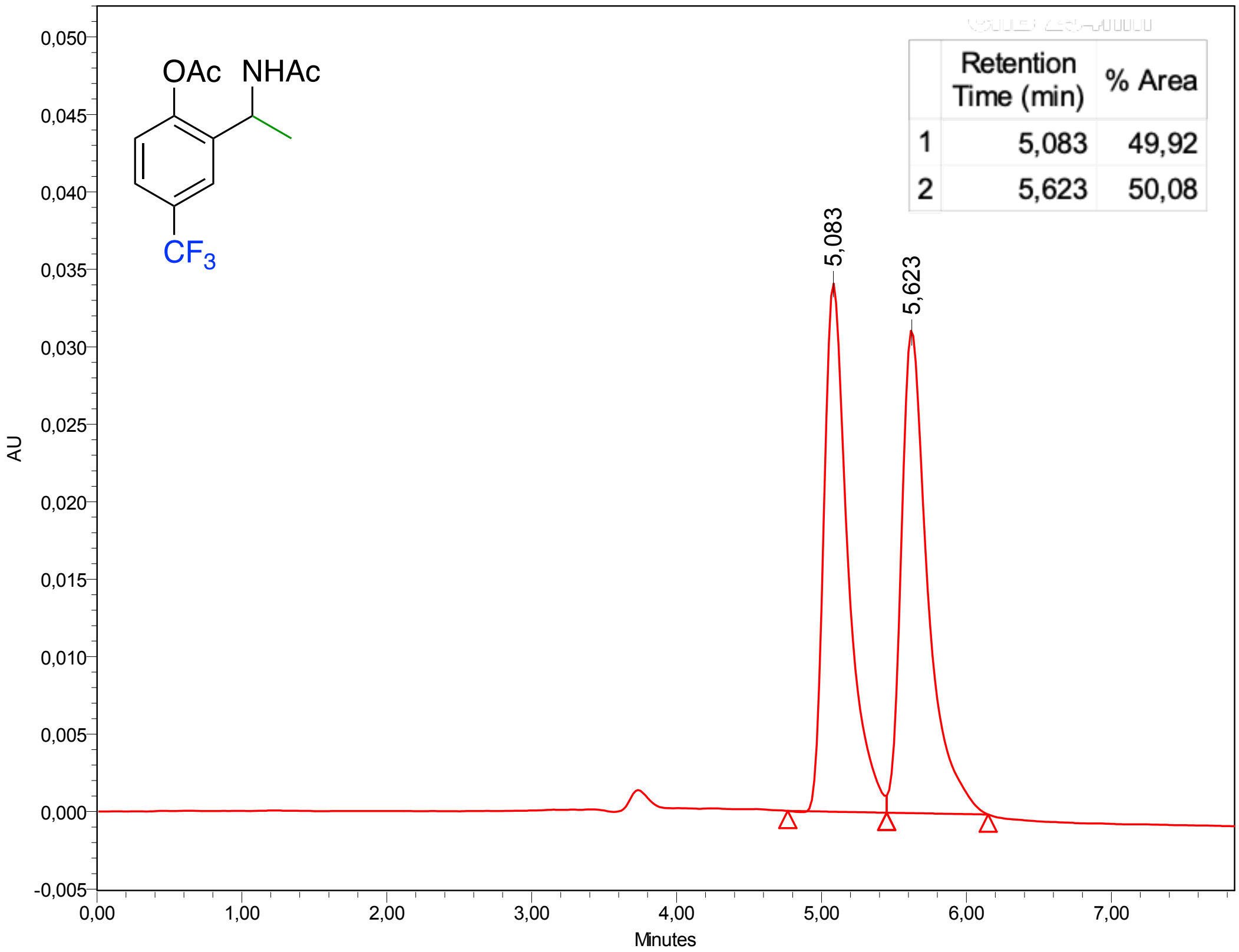
OAc NHAc
CF3
Retention Time (min) | % Area
1 5,083 49,92
2 5,623 50,08
5,083
5,623
AU
Minutes
0,050
0,045
0,040
0,035
0,030
0,025
0,020
0,015
0,010
0,005
0,000
-0,005
0,00
1,00
2,00
3,00
4,00
5,00
6,00
7,00

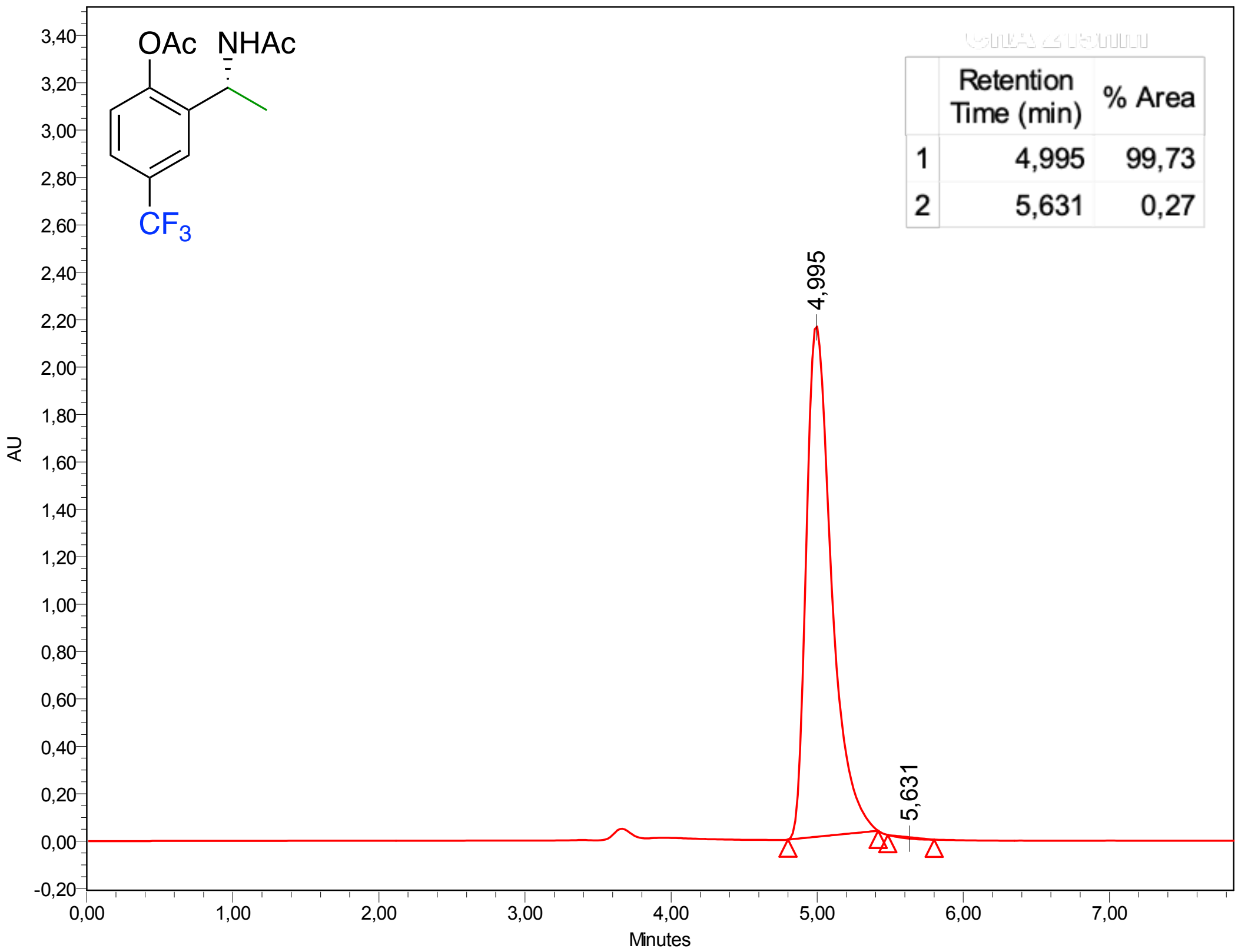
OAc NHAc
CF3
Retention Time (min) | % Area
1 4,995 99,73
2 5,631 0,27
4,995
5,631
AU
Minutes
3,40
3,20
3,00
2,80
2,60
2,40
2,20
2,00
1,80
1,60
1,40
1,20
1,00
0,80
0,60
0,40
0,20
0,00
-0,20
0,00
1,00
2,00
3,00
4,00
5,00
6,00
7,00

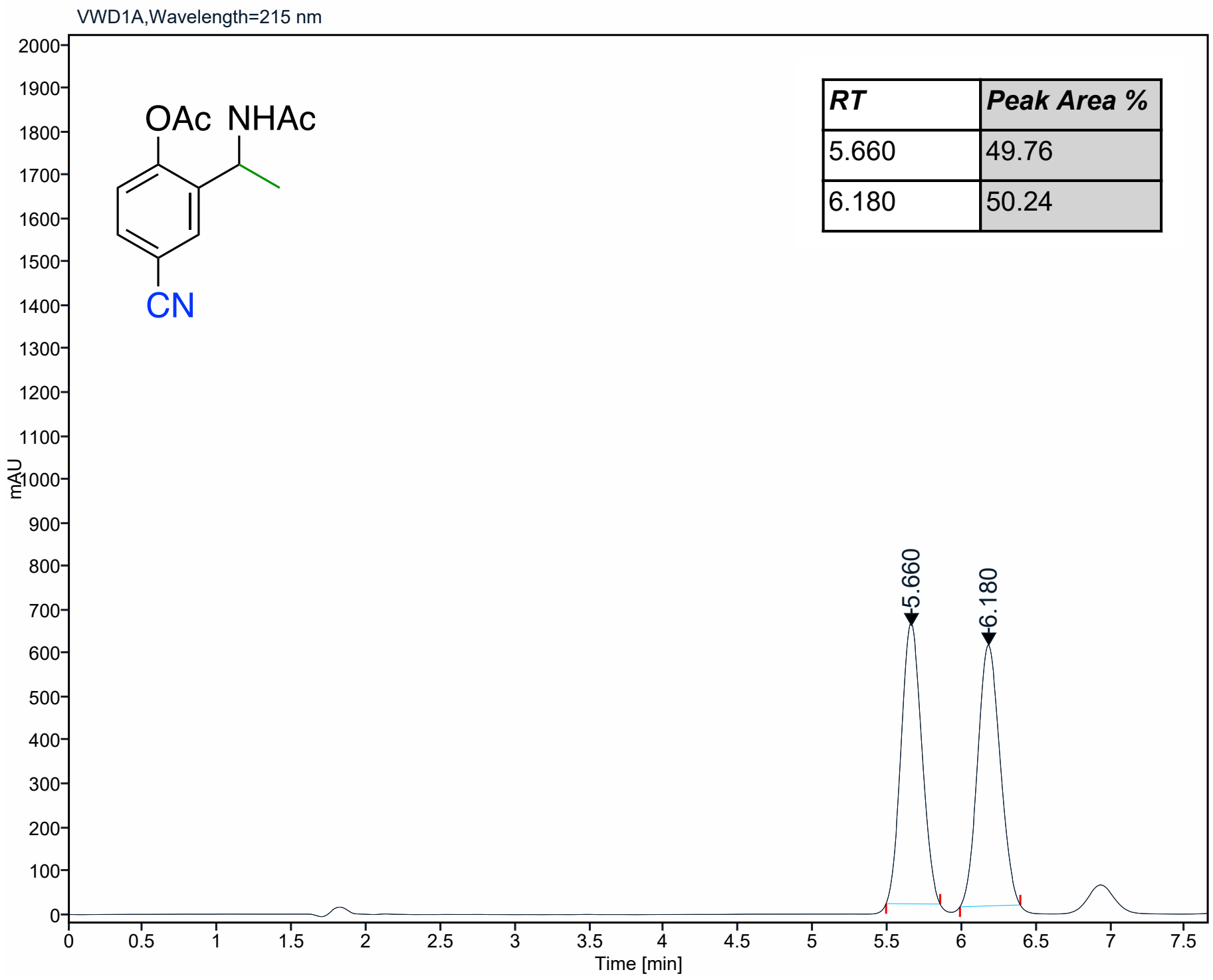
VWD1A,Wavelength=215 nm
OAc NHAc
CN
RT | Peak Area %
5.660 | 49.76
6.180 | 50.24
mAU
Time [min]
5.660
6.180

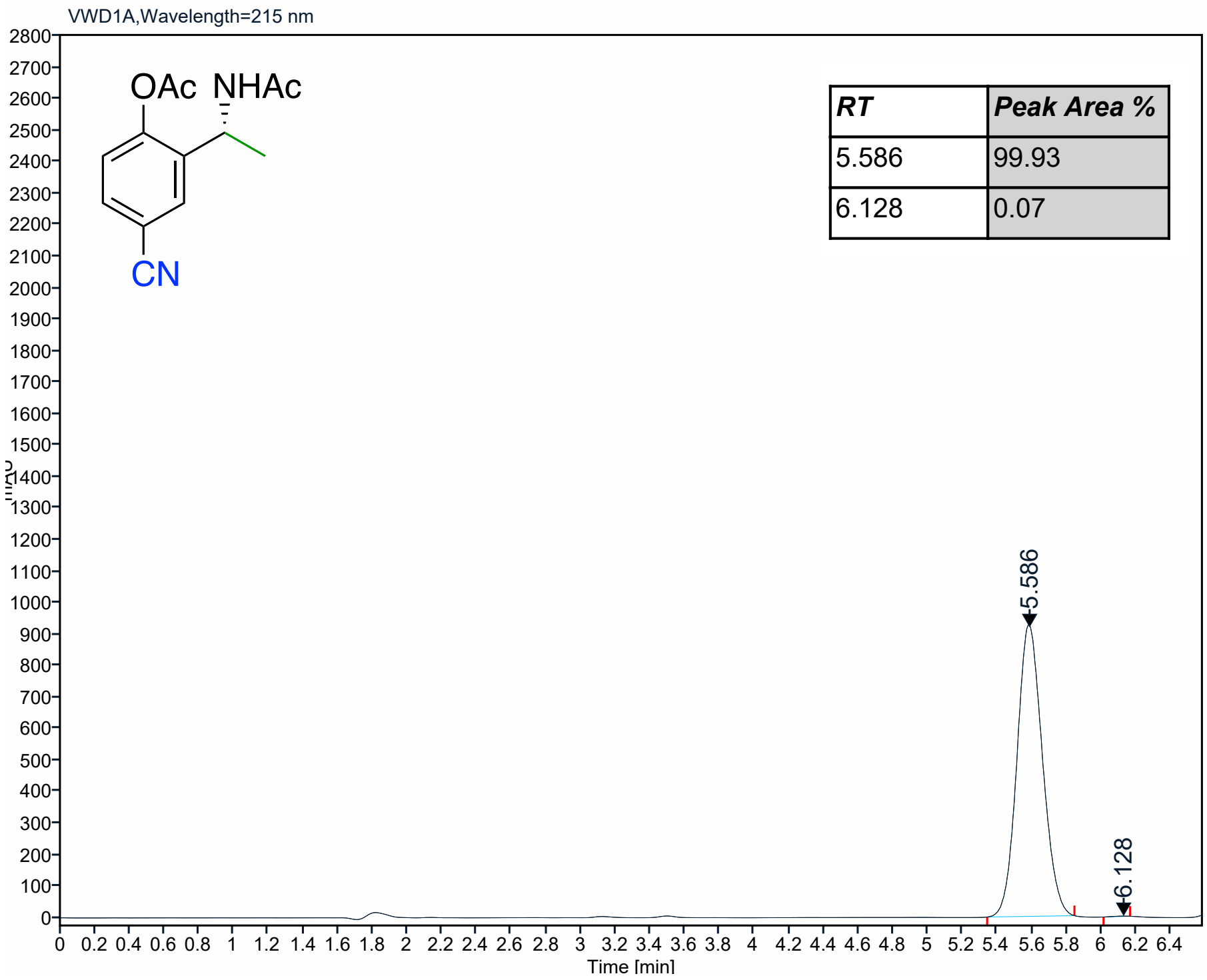
VWD1A,Wavelength=215 nm
OAc NHAc
CN
RT | Peak Area %
5.586 | 99.93
6.128 | 0.07
Time [min]
5.586
6.128

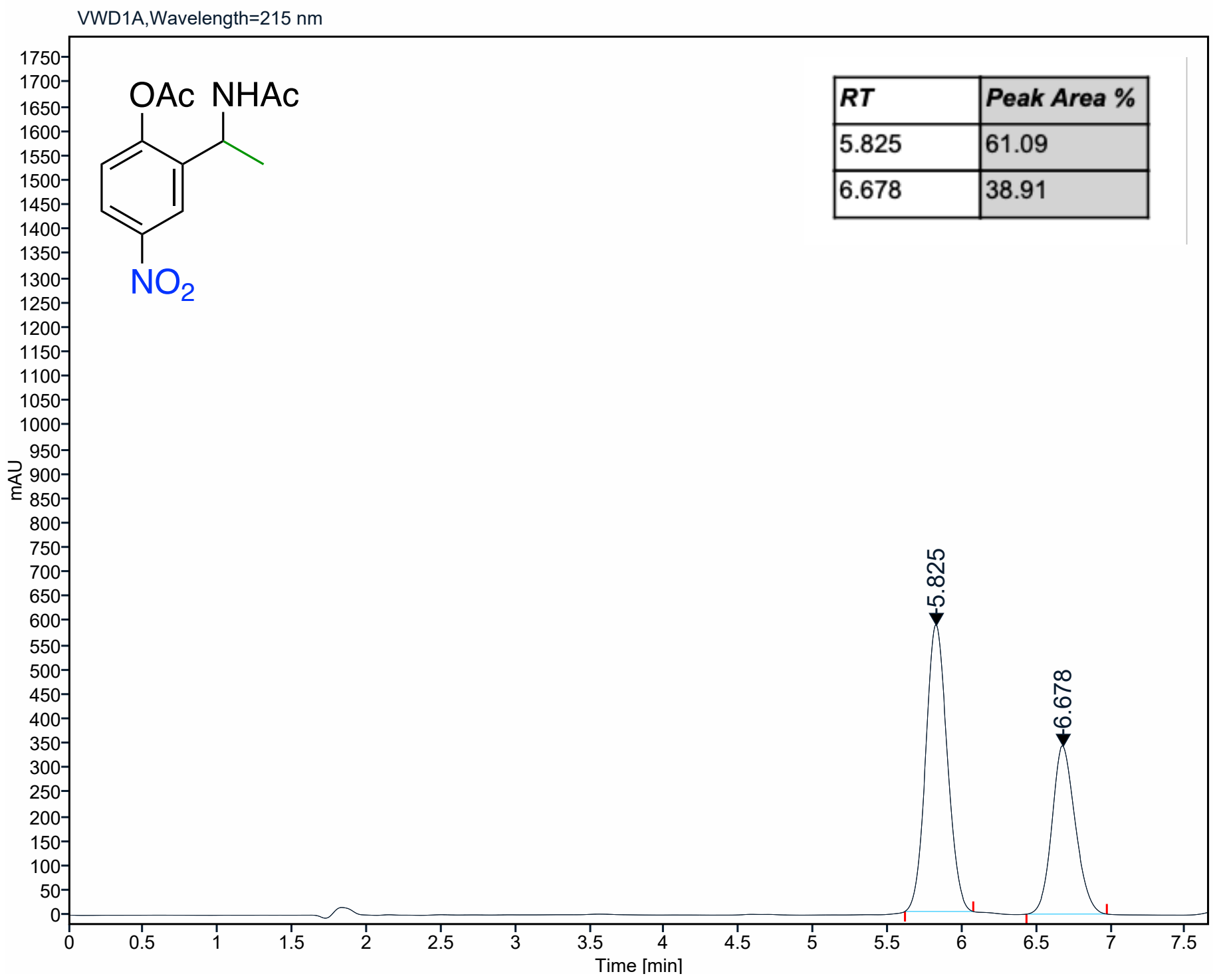

VWD1A,Wavelength=215 nm
OAc NHAc
NO2
RT | Peak Area %
5.825 | 61.09
6.678 | 38.91
5.825
6.678
mAU
Time [min]

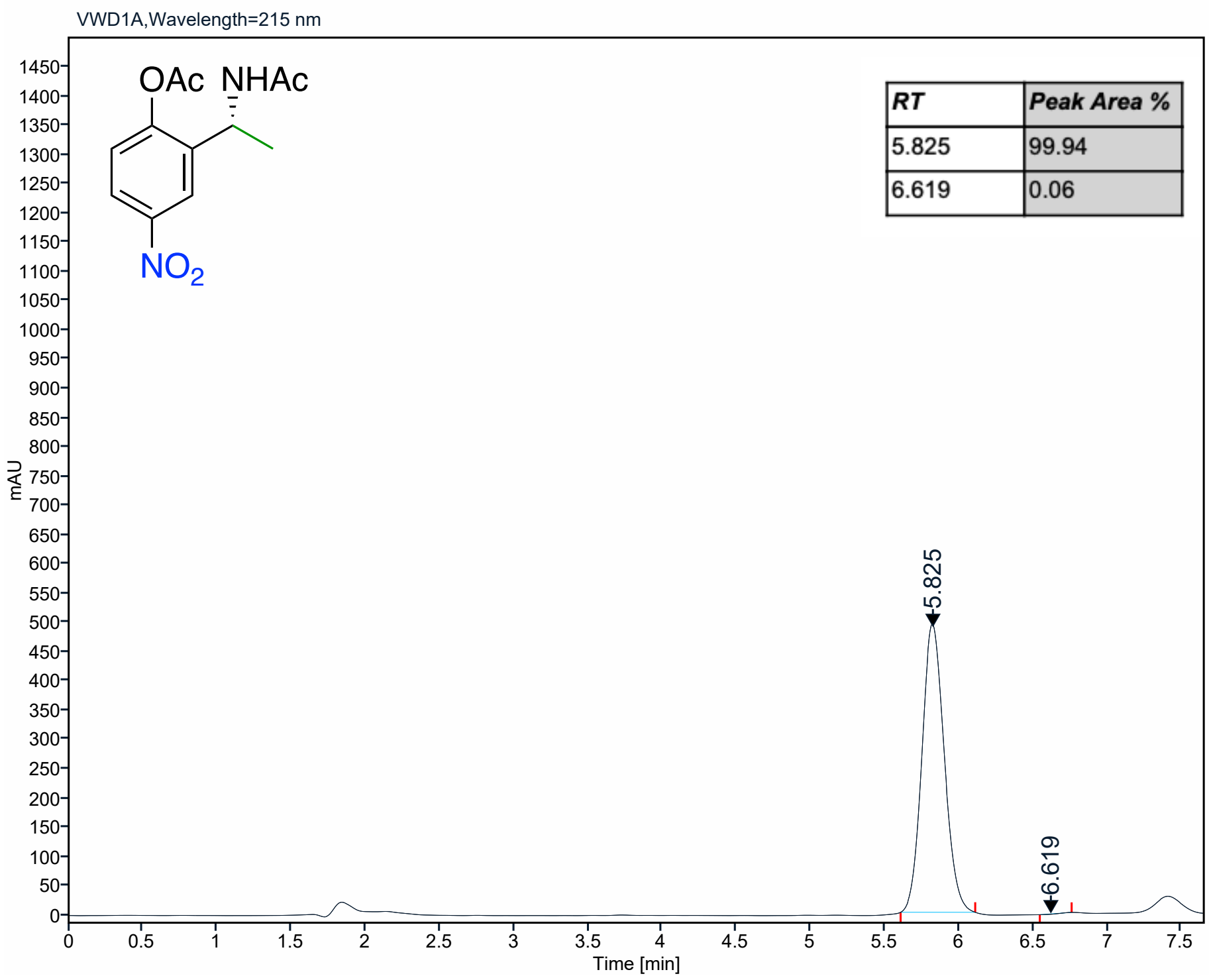

VWD1A,Wavelength=215 nm
OAc NHAc
NO2
RT | Peak Area %
5.825 | 99.94
6.619 | 0.06
5.825
6.619
mAU
Time [min]

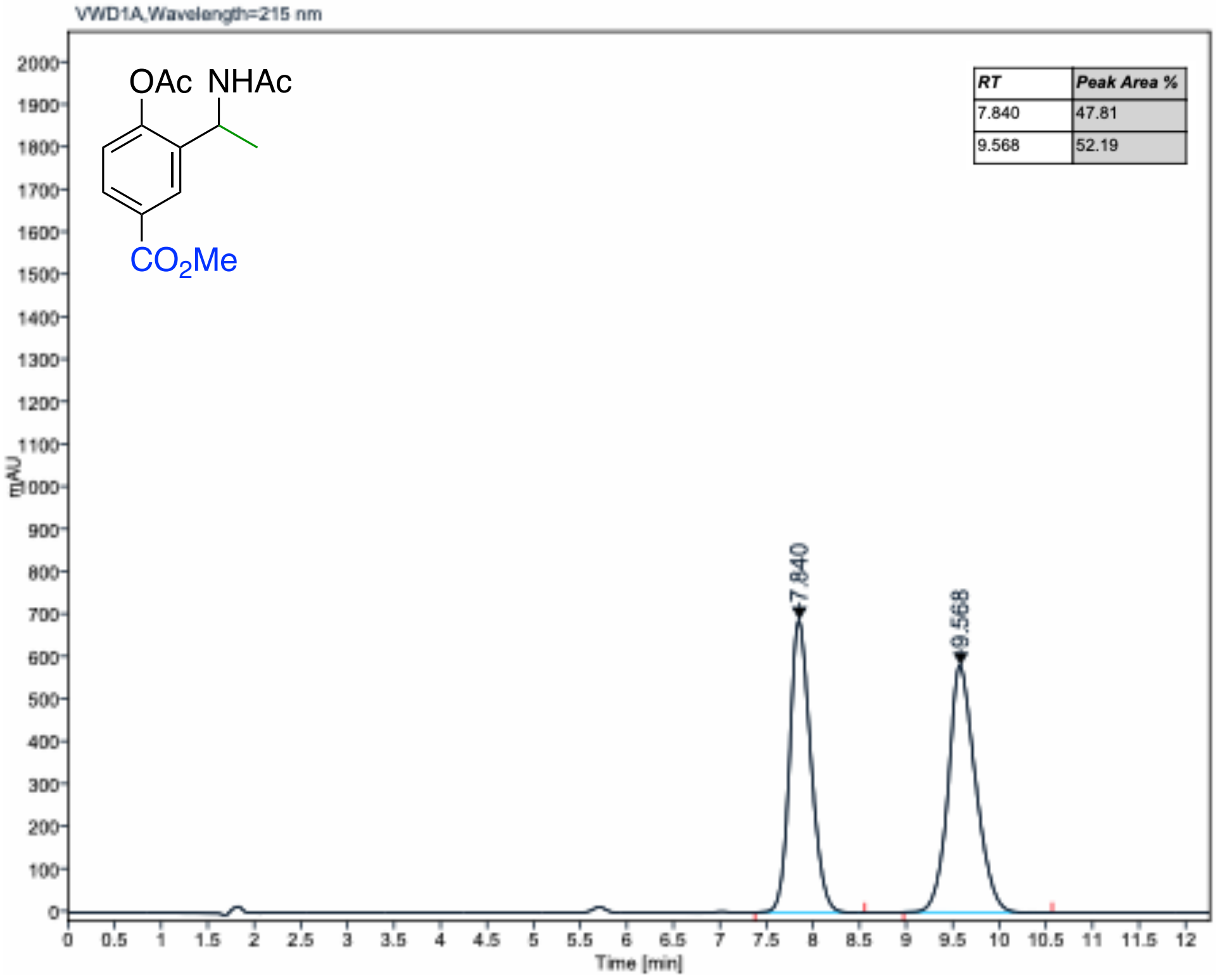

VWD1A,Wavelength=215 nm
OAc NHAc
CO_2Me
RT | Peak Area %
7.840 | 47.81
9.568 | 52.19
7.840
9.568
mAU
Time [min]

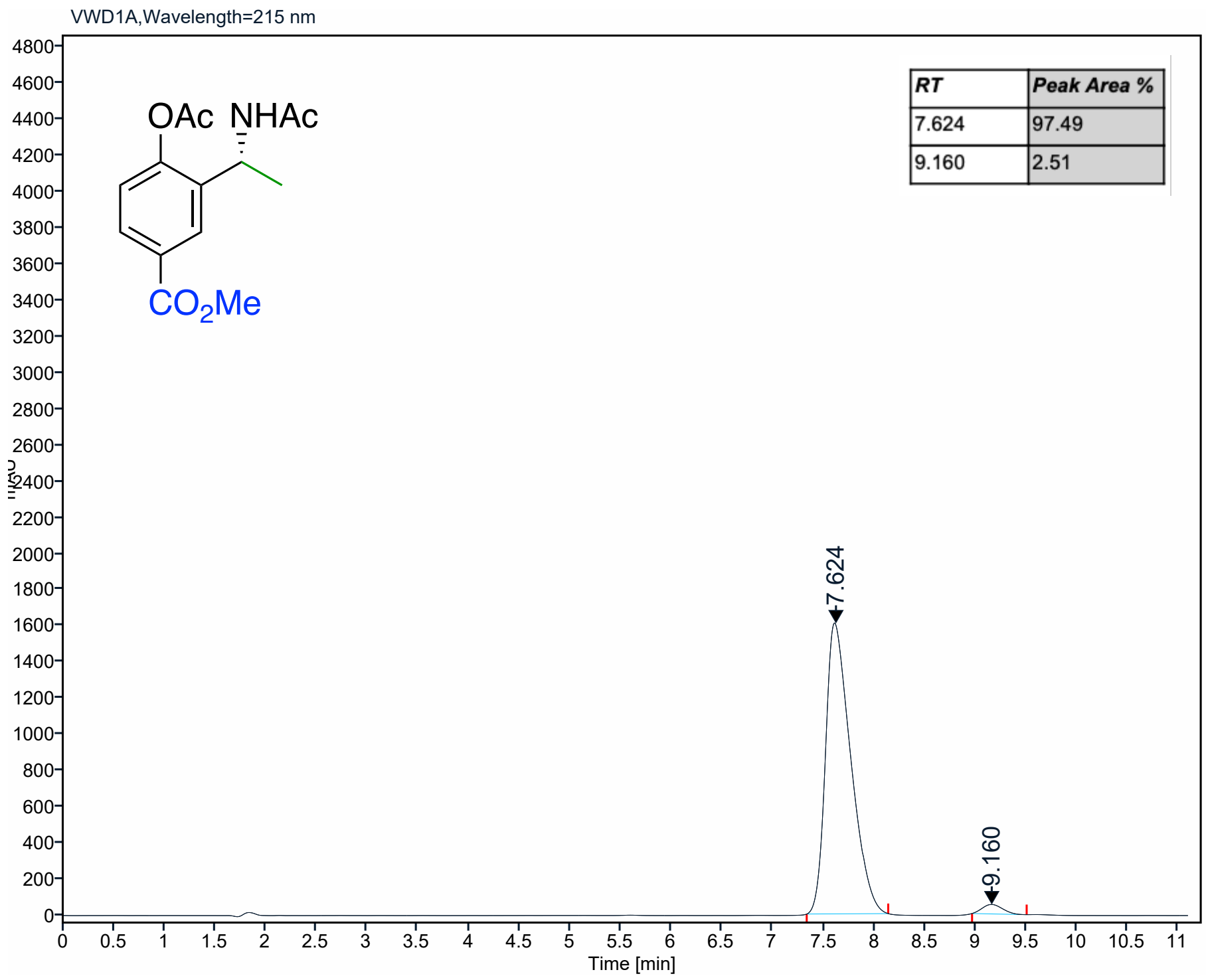

VWD1A,Wavelength=215 nm
OAc NHAc
CO_2Me
RT | Peak Area %
7.624 | 97.49
9.160 | 2.51
7.624
9.160
Time [min]

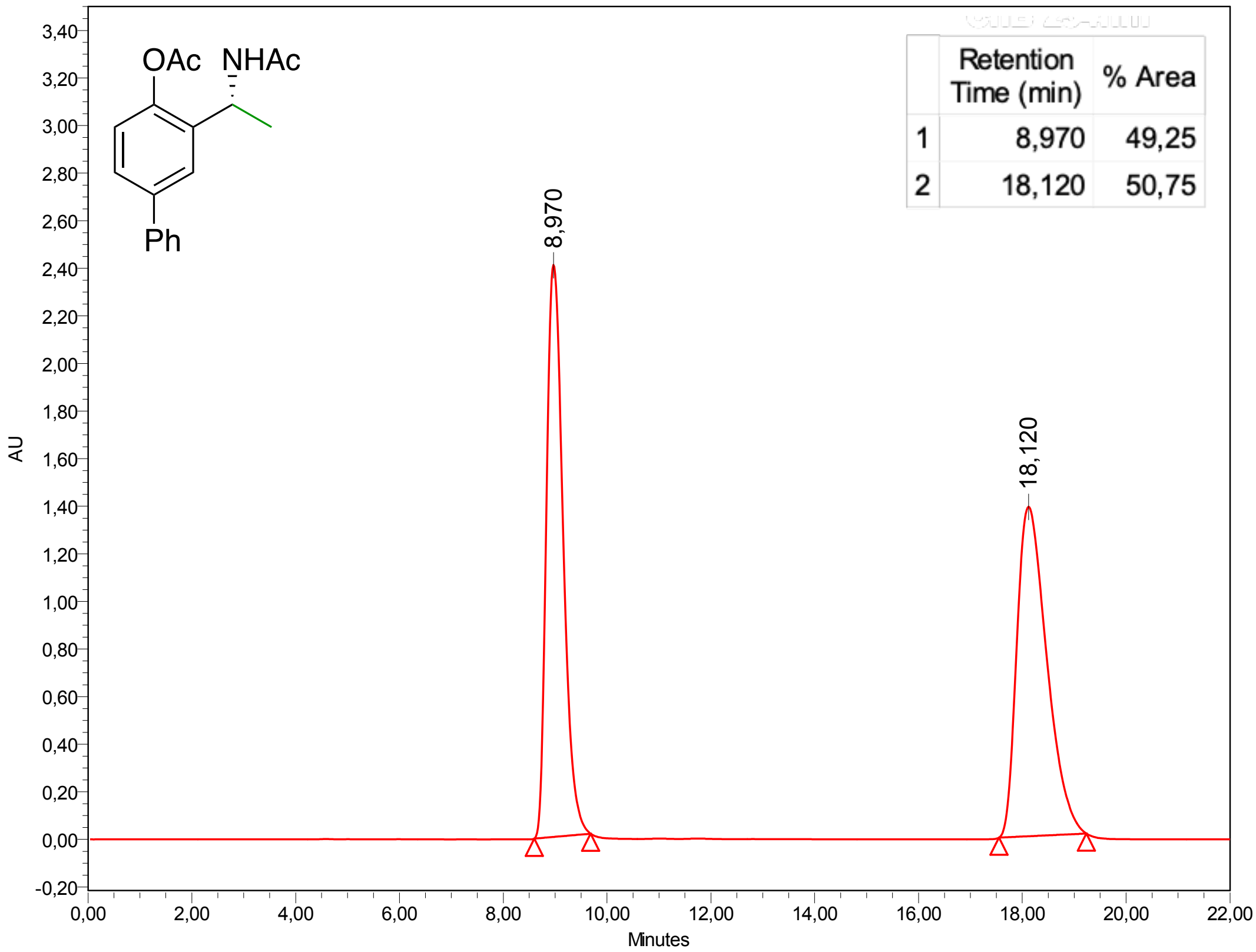

OAc NHAc
Ph
Retention Time (min)
% Area
1 8,970 49,25
2 18,120 50,75
8,970
18,120
AU
Minutes

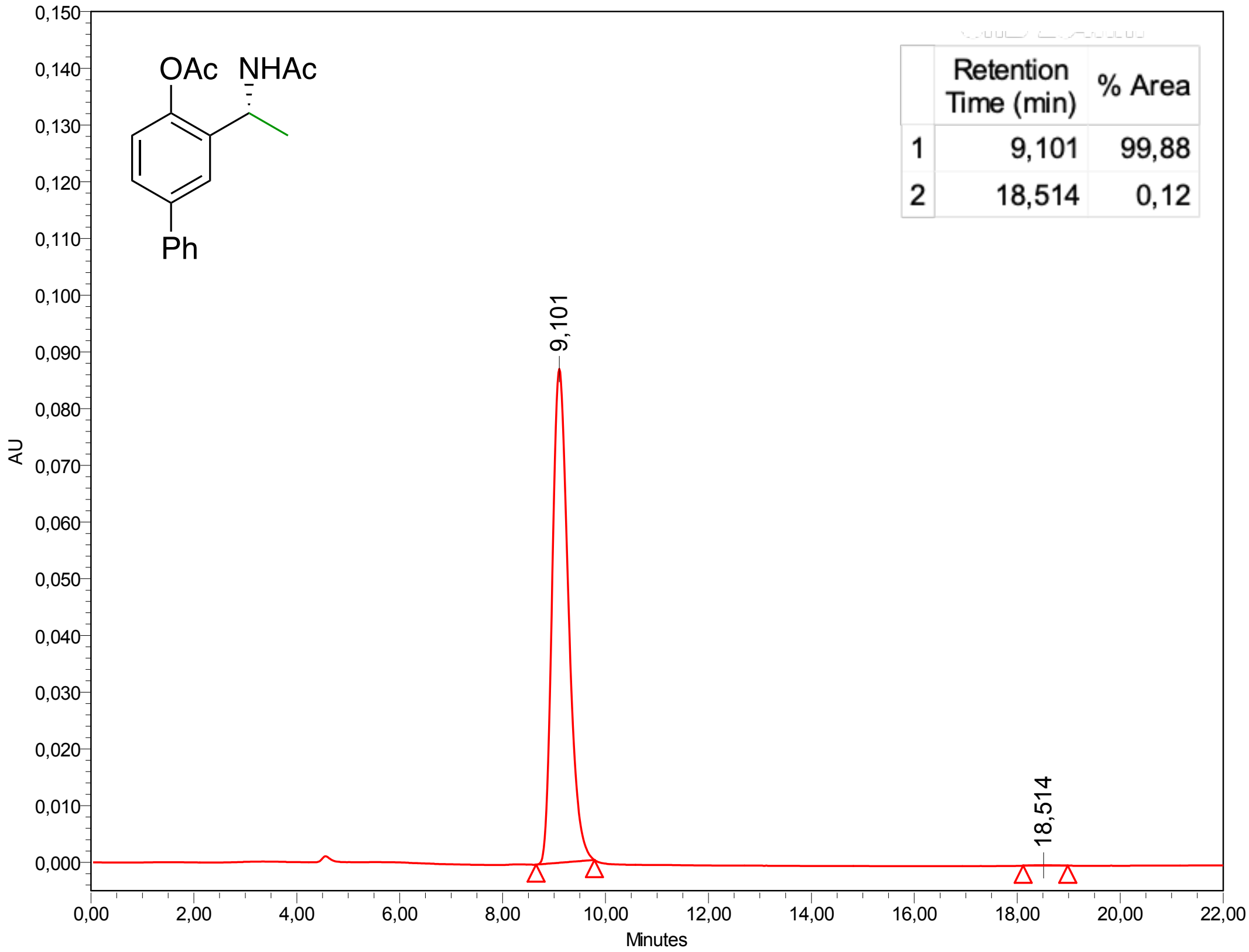

OAc NHAc
Ph
Retention Time (min)
% Area
1 9,101 99,88
2 18,514 0,12
9,101
18,514
AU
Minutes

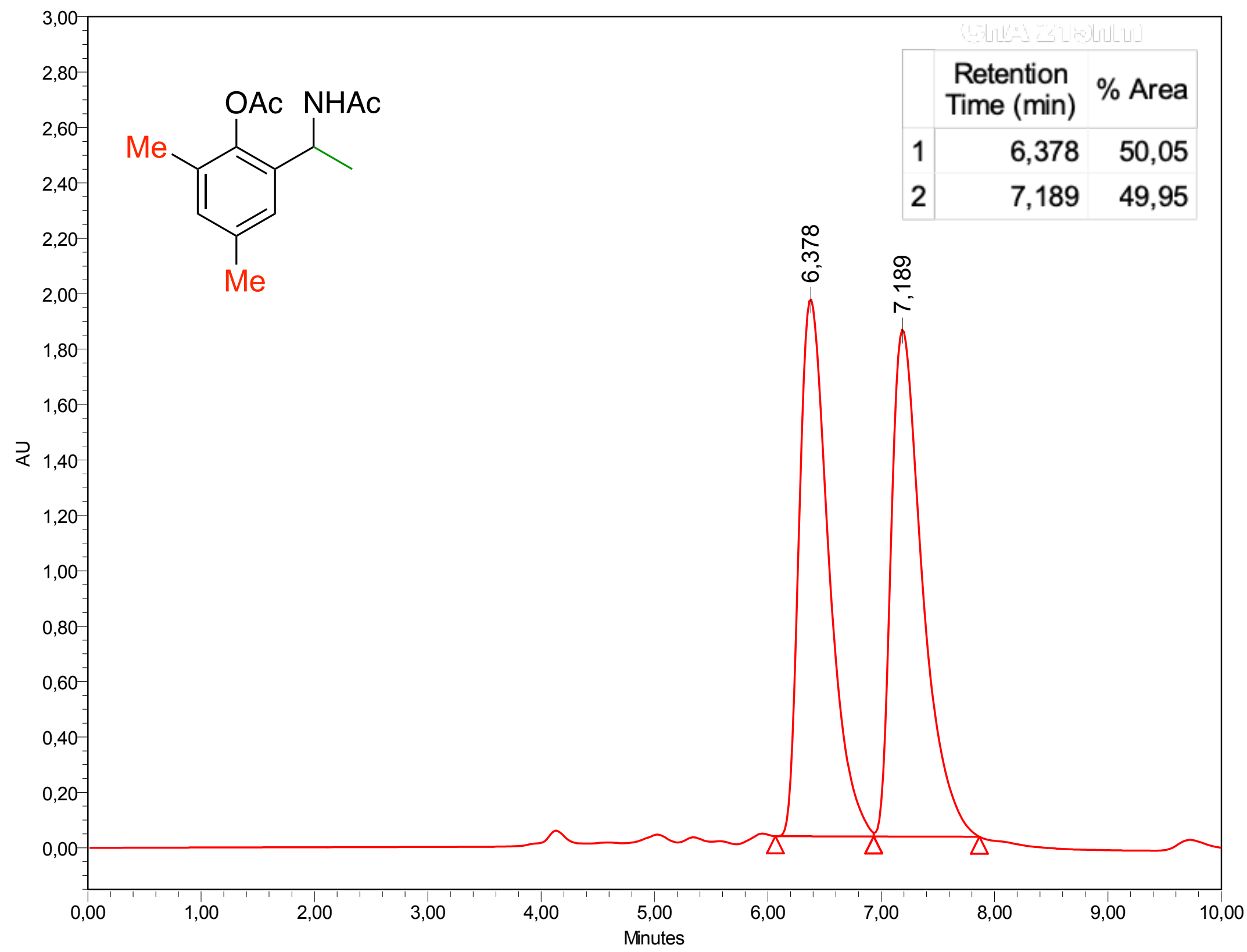
OAc NHAc
Me
Me
Retention Time (min)
% Area
1 6,378 50,05
2 7,189 49,95
6,378
7,189
AU
Minutes

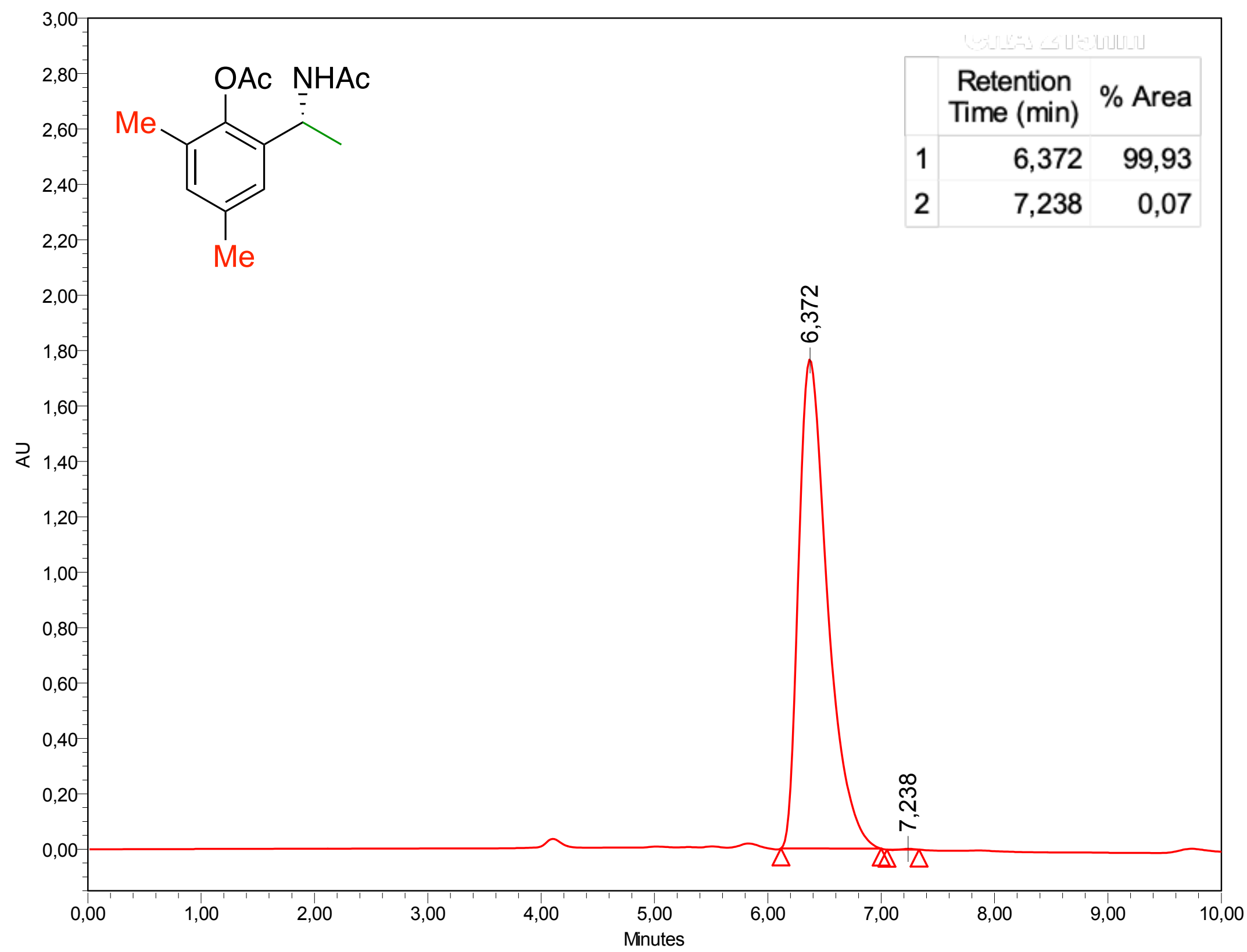
OAc NHAc
Me
Me
Retention Time (min)
% Area
1 6,372 99,93
2 7,238 0,07
6,372
7,238
AU
Minutes

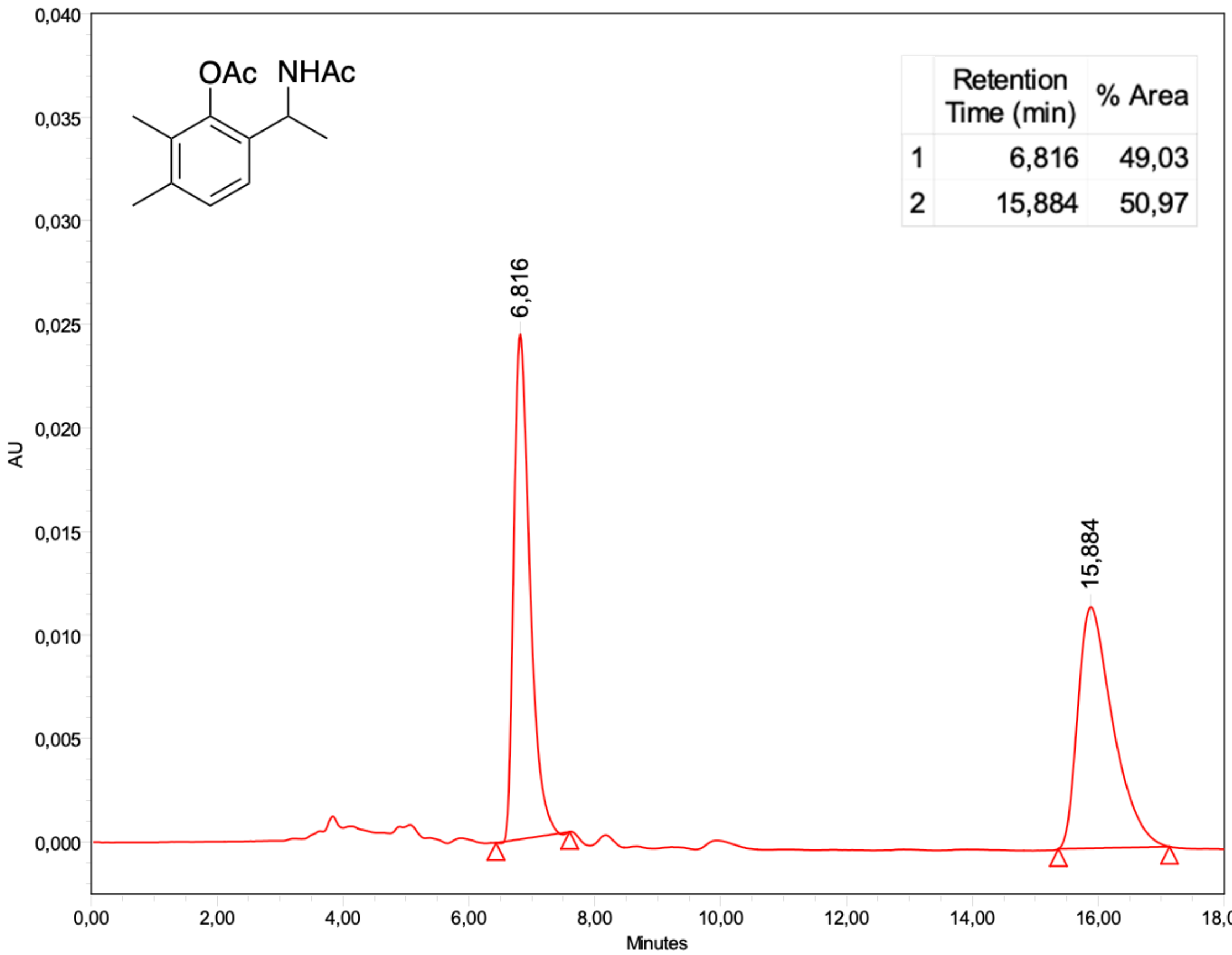
OAc NHAc
Retention Time (min) | % Area
1 6,816 49,03
2 15,884 50,97
6,816
15,884
AU
Minutes
0,040
0,035
0,030
0,025
0,020
0,015
0,010
0,005
0,000
0,00 2,00 4,00 6,00 8,00 10,00 12,00 14,00 16,00 18,00

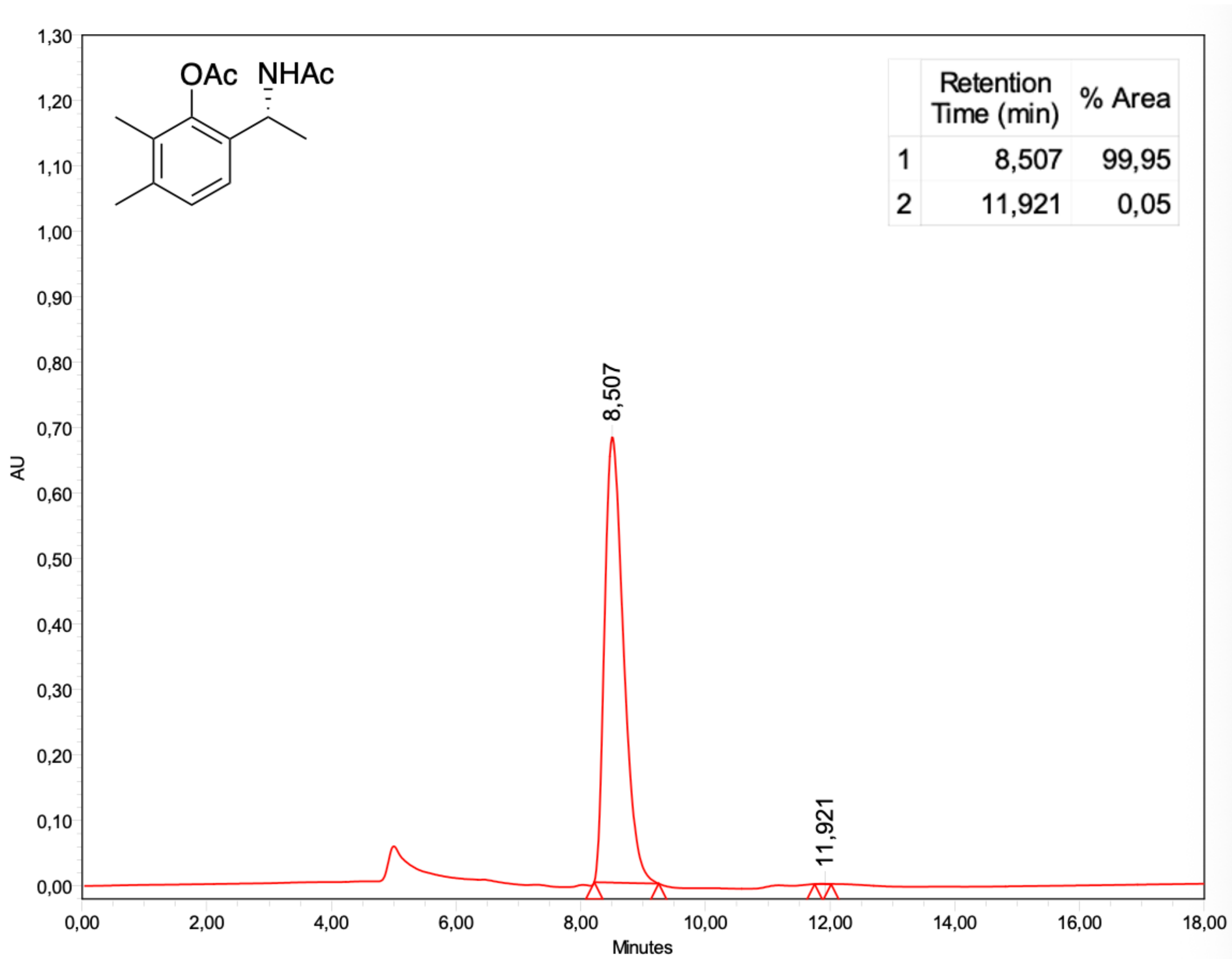
OAc NHAc
Retention Time (min) | % Area
1 8,507 99,95
2 11,921 0,05
8,507
11,921
AU
Minutes
1,30
1,20
1,10
1,00
0,90
0,80
0,70
0,60
0,50
0,40
0,30
0,20
0,10
0,00
0,00 2,00 4,00 6,00 8,00 10,00 12,00 14,00 16,00 18,00

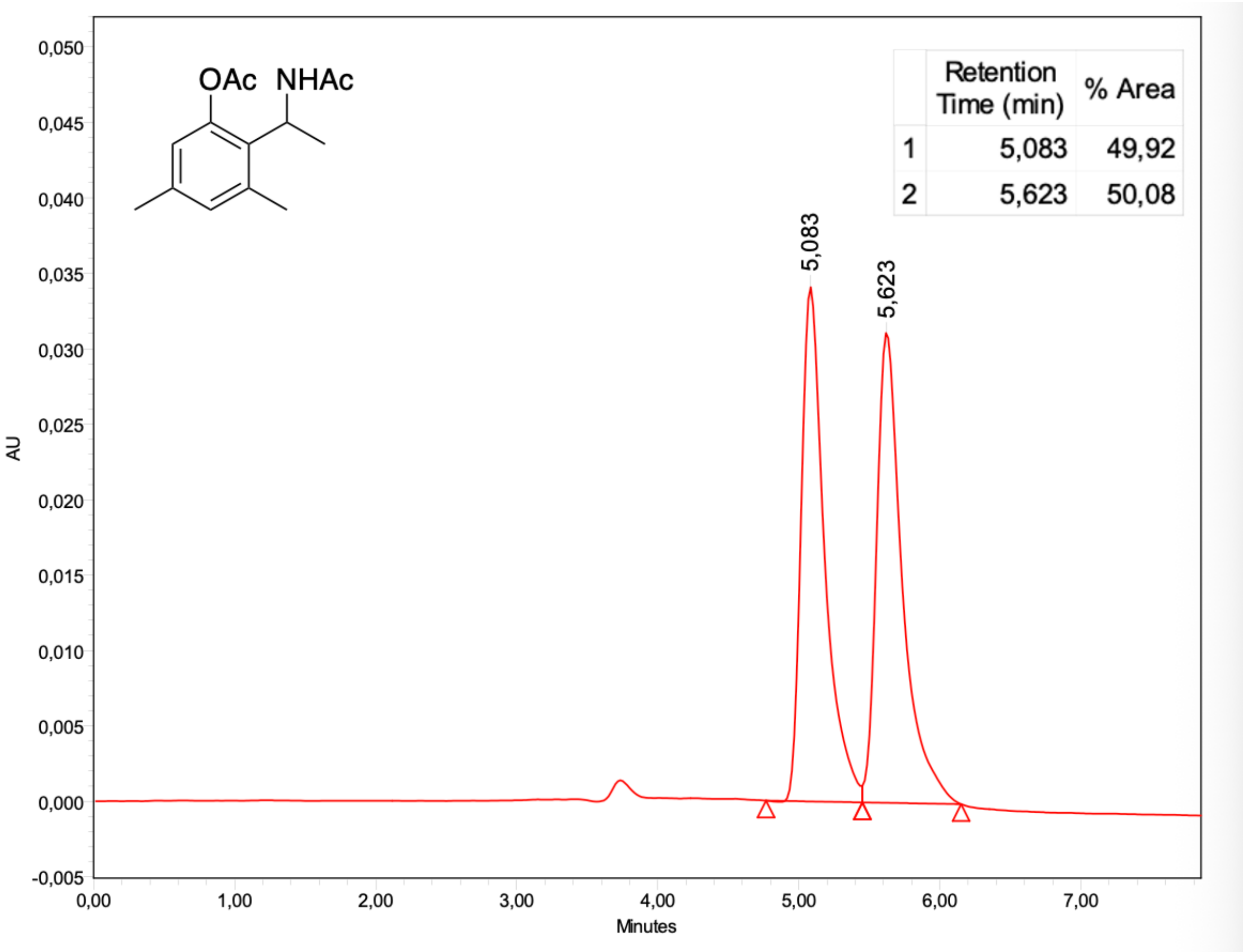
OAc NHAc
Retention Time (min) % Area
1 5,083 49,92
2 5,623 50,08
5,083
5,623
AU
Minutes

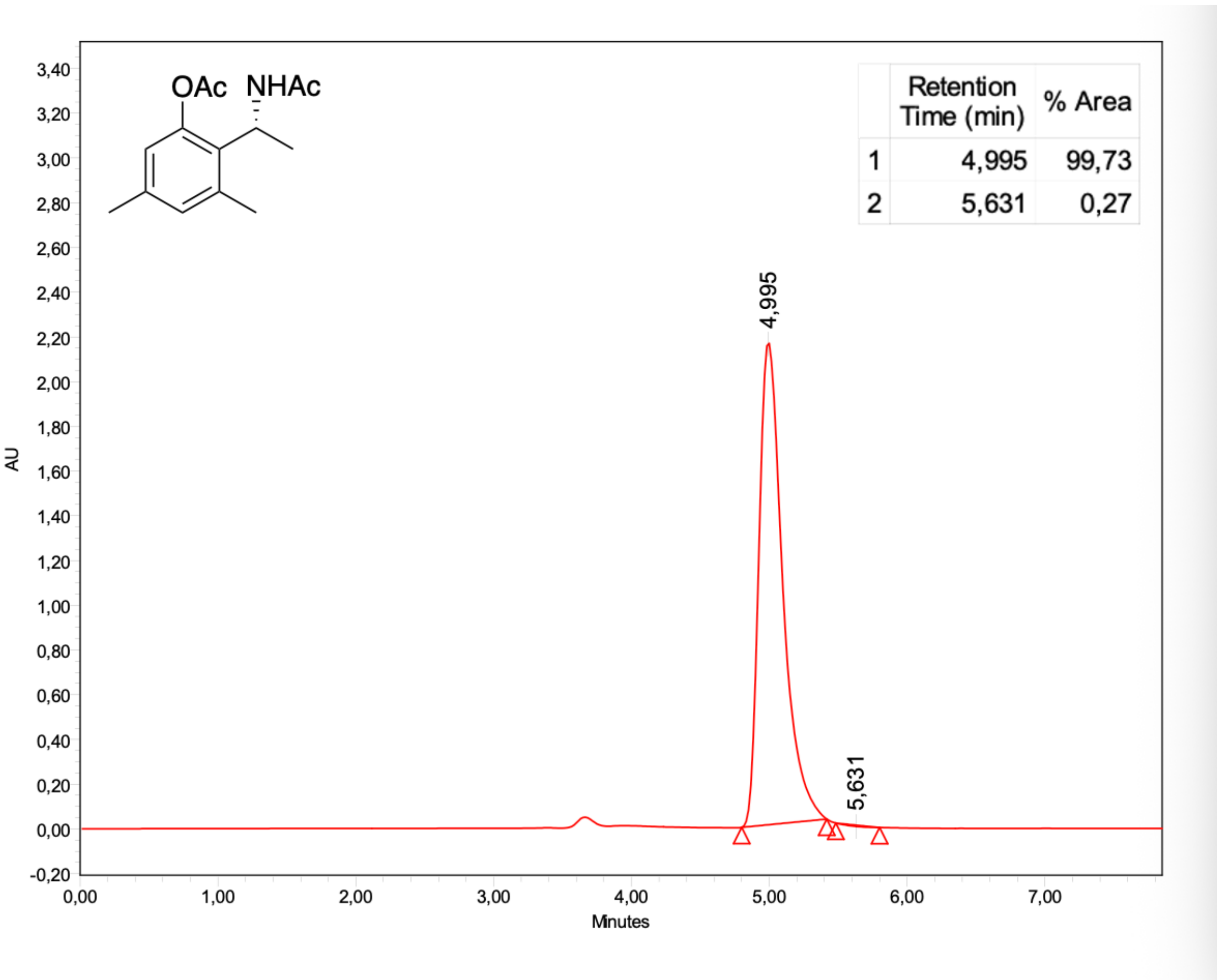
OAc NHAc
Retention Time (min) % Area
1 4,995 99,73
2 5,631 0,27
4,995
5,631
AU
Minutes

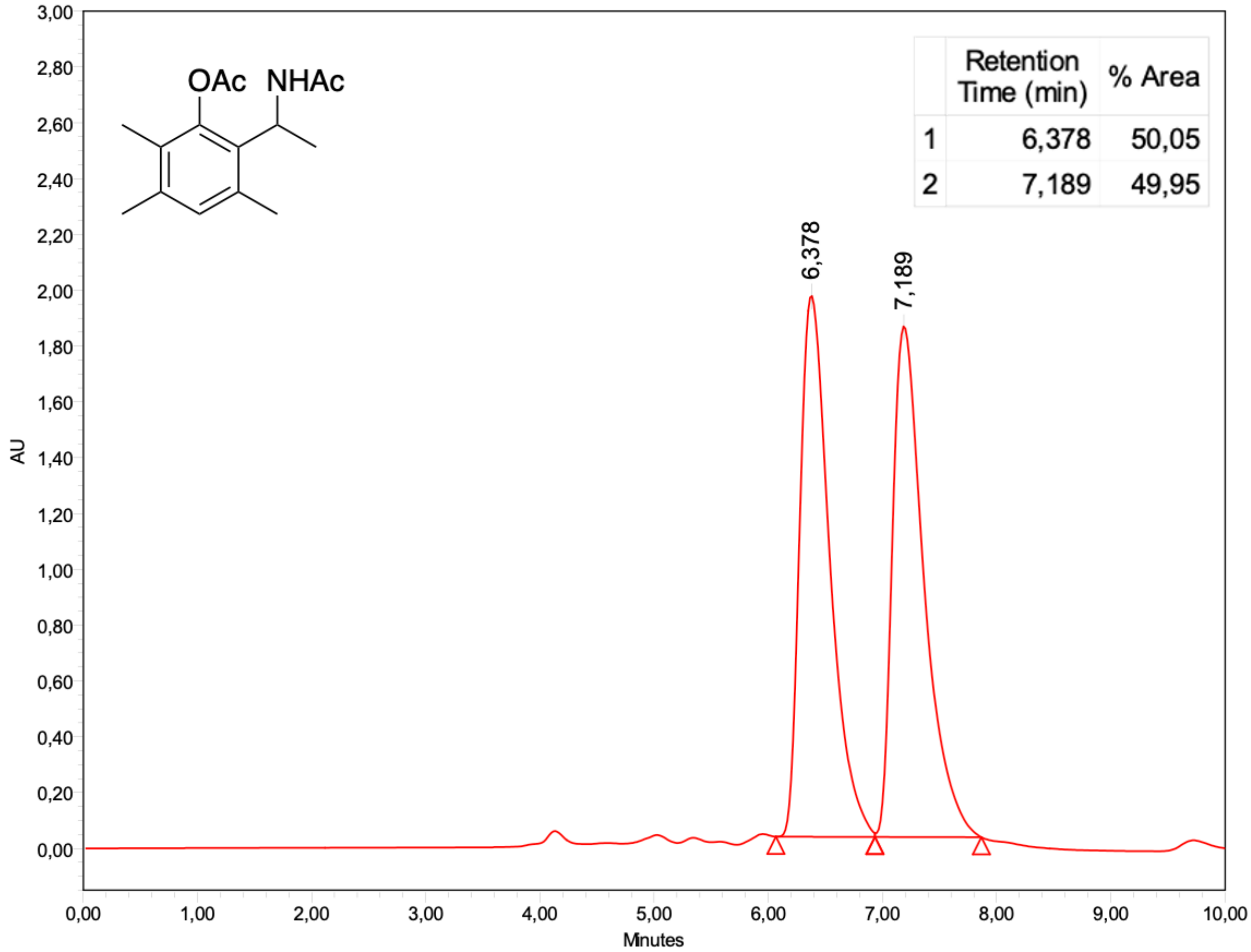

	Retention Time (min)	% Area
1	6,378	50,05
2	7,189	49,95

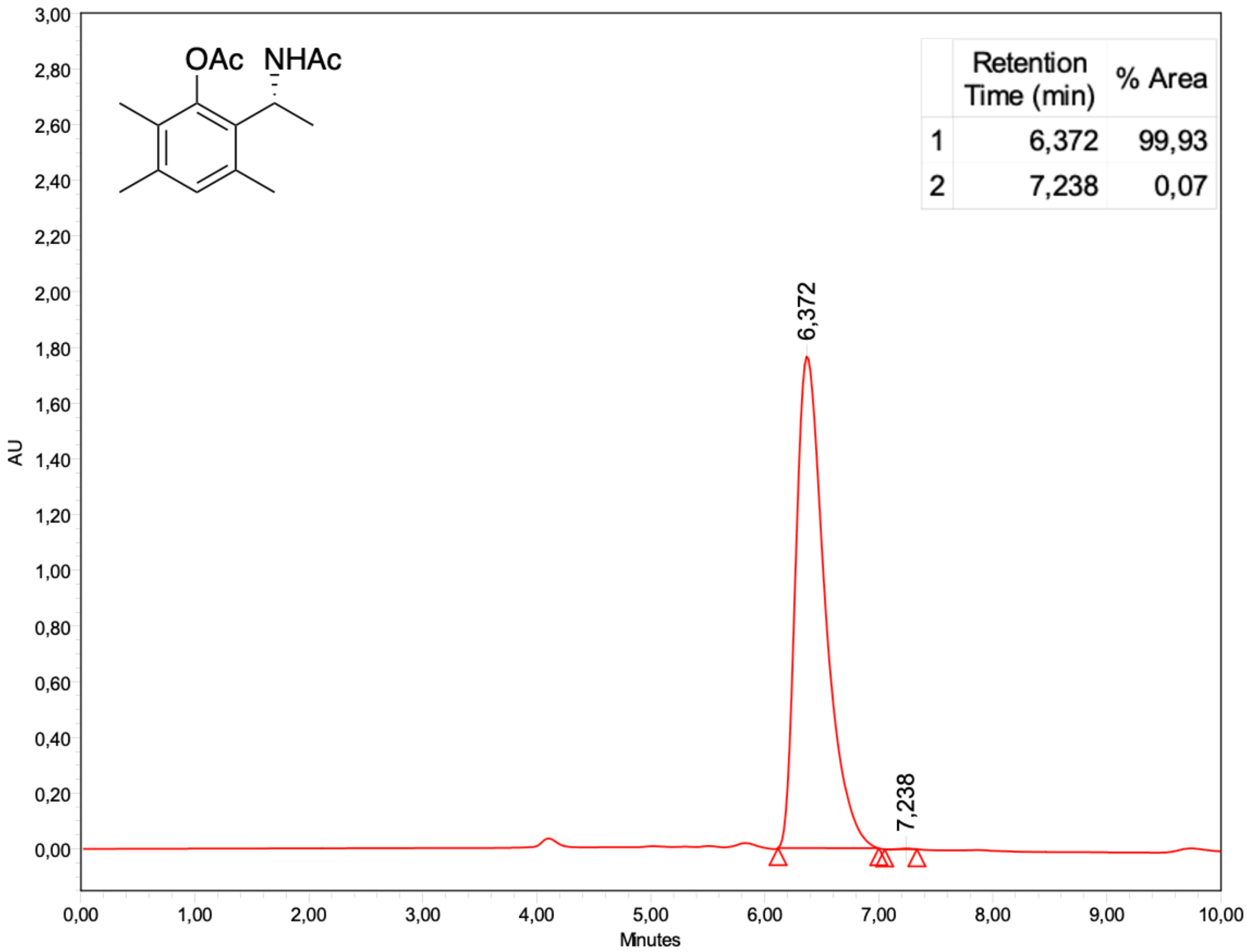

	Retention Time (min)	% Area
1	6,372	99,93
2	7,238	0,07

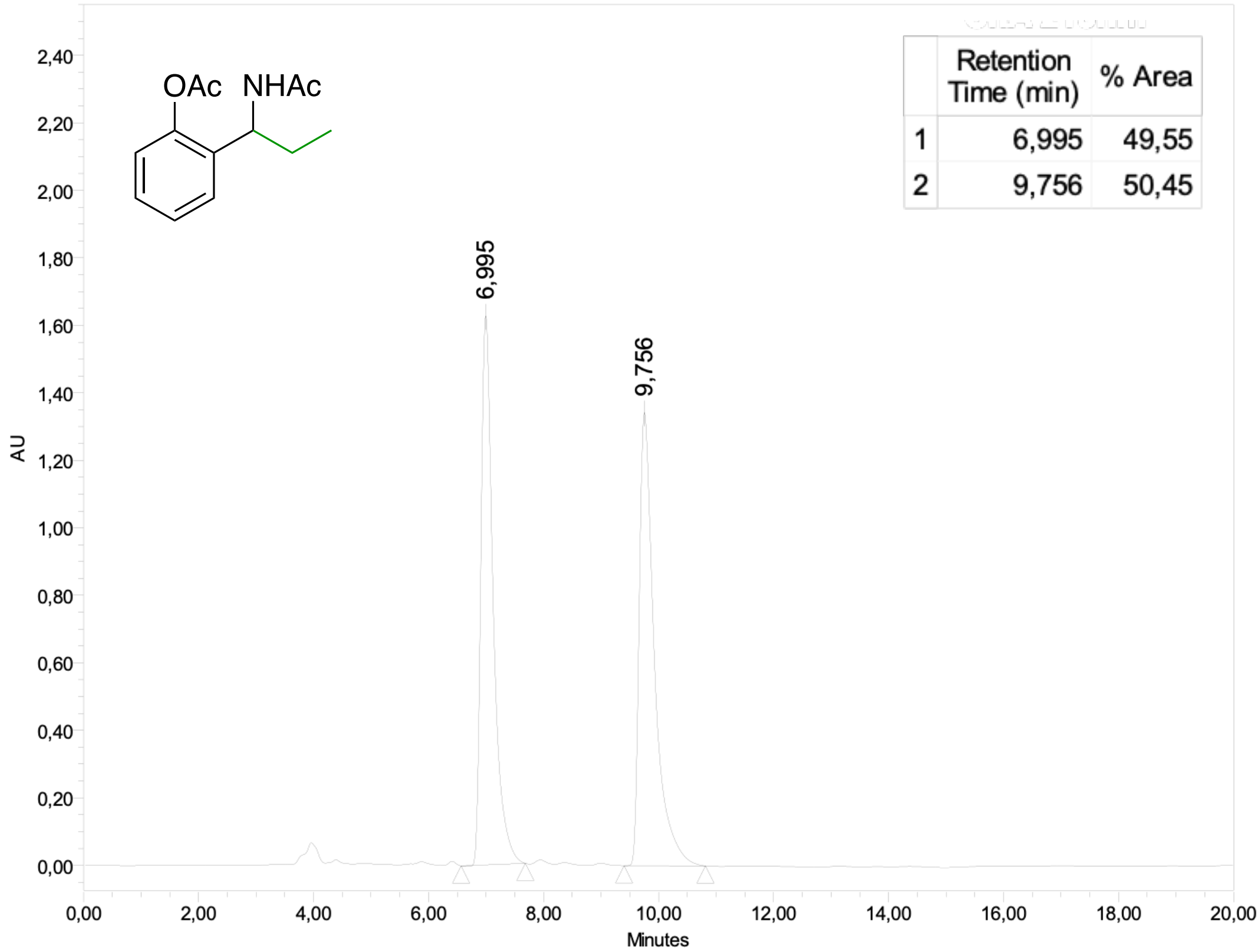
OAc NHAc
Retention Time (min) % Area
1 6,995 49,55
2 9,756 50,45
6,995
9,756
AU
Minutes

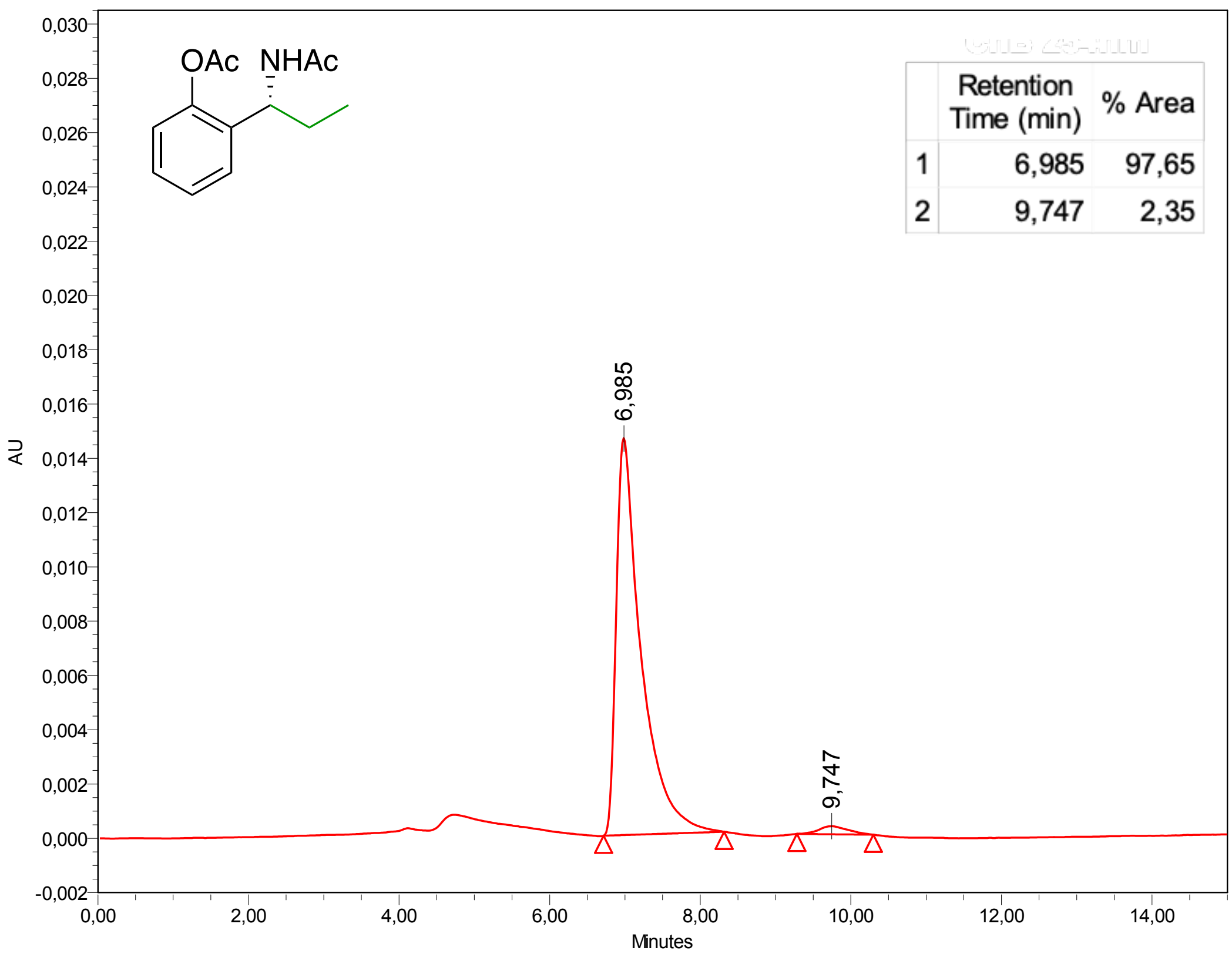
OAc NHAc
Retention Time (min) % Area
1 6,985 97,65
2 9,747 2,35
6,985
9,747
AU
Minutes

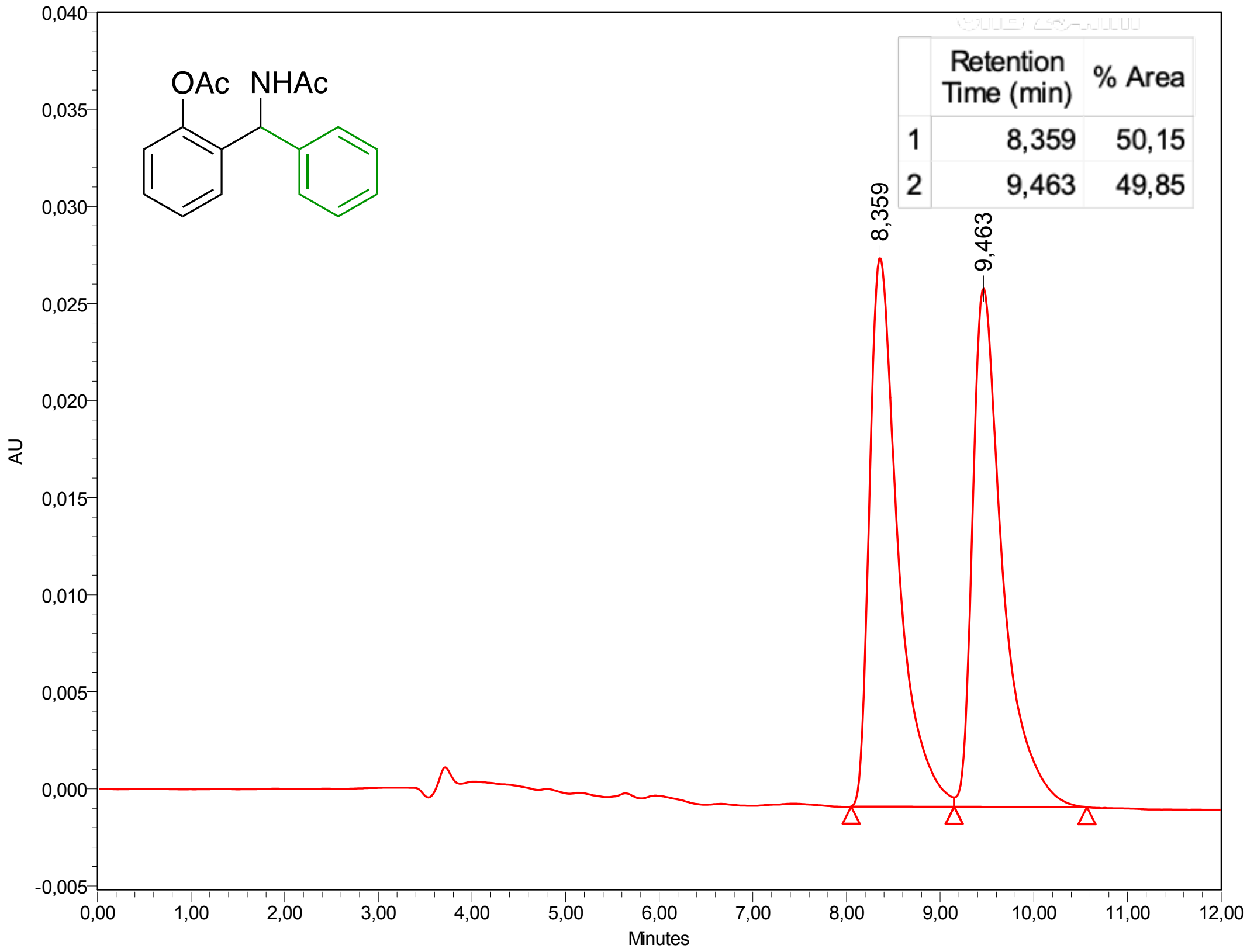
OAc NHAc
Retention Time (min) | % Area
1 8,359 50,15
2 9,463 49,85
8,359
9,463
AU
Minutes

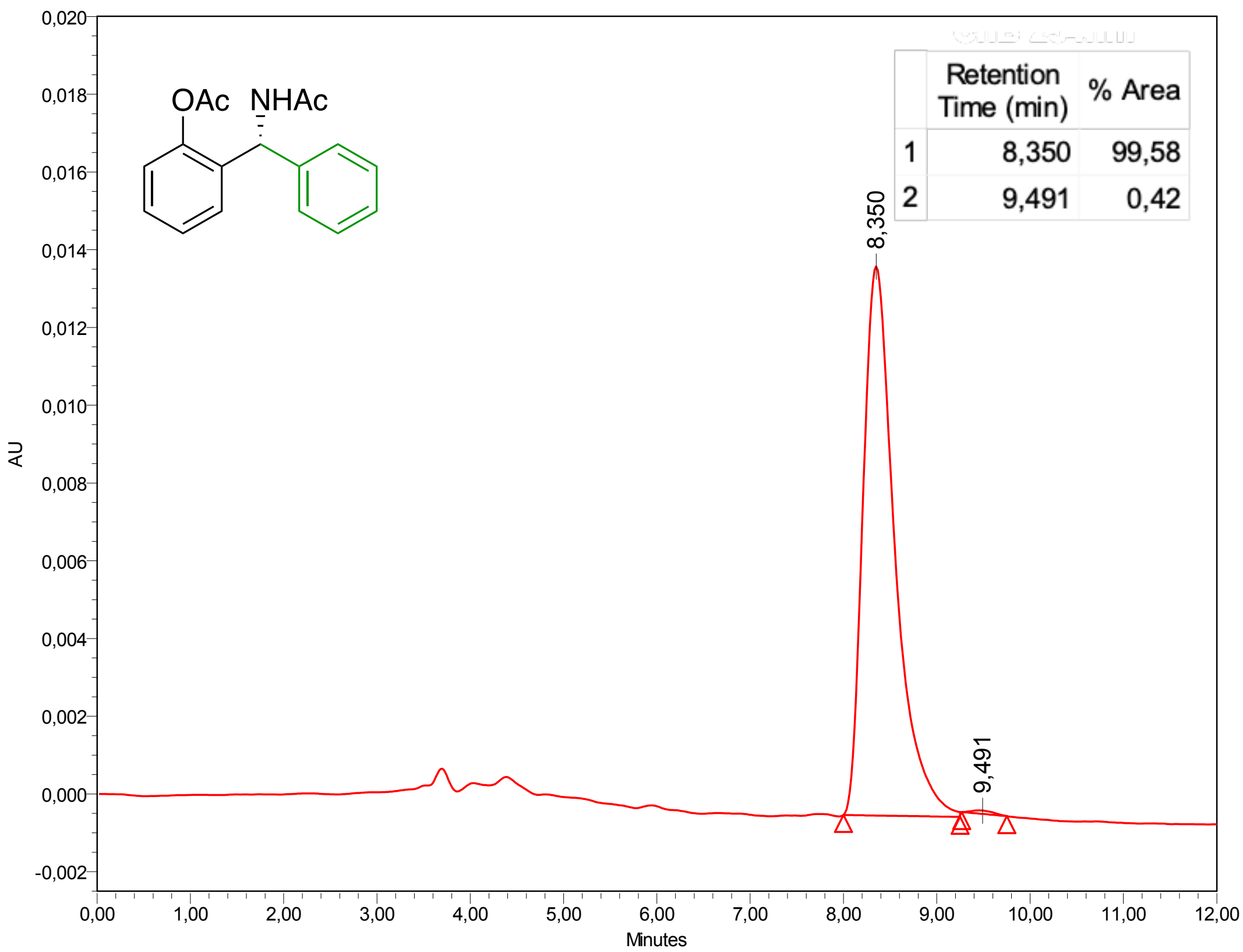
OAc NHAc
Retention Time (min) | % Area
1 8,350 99,58
2 9,491 0,42
8,350
9,491
AU
Minutes

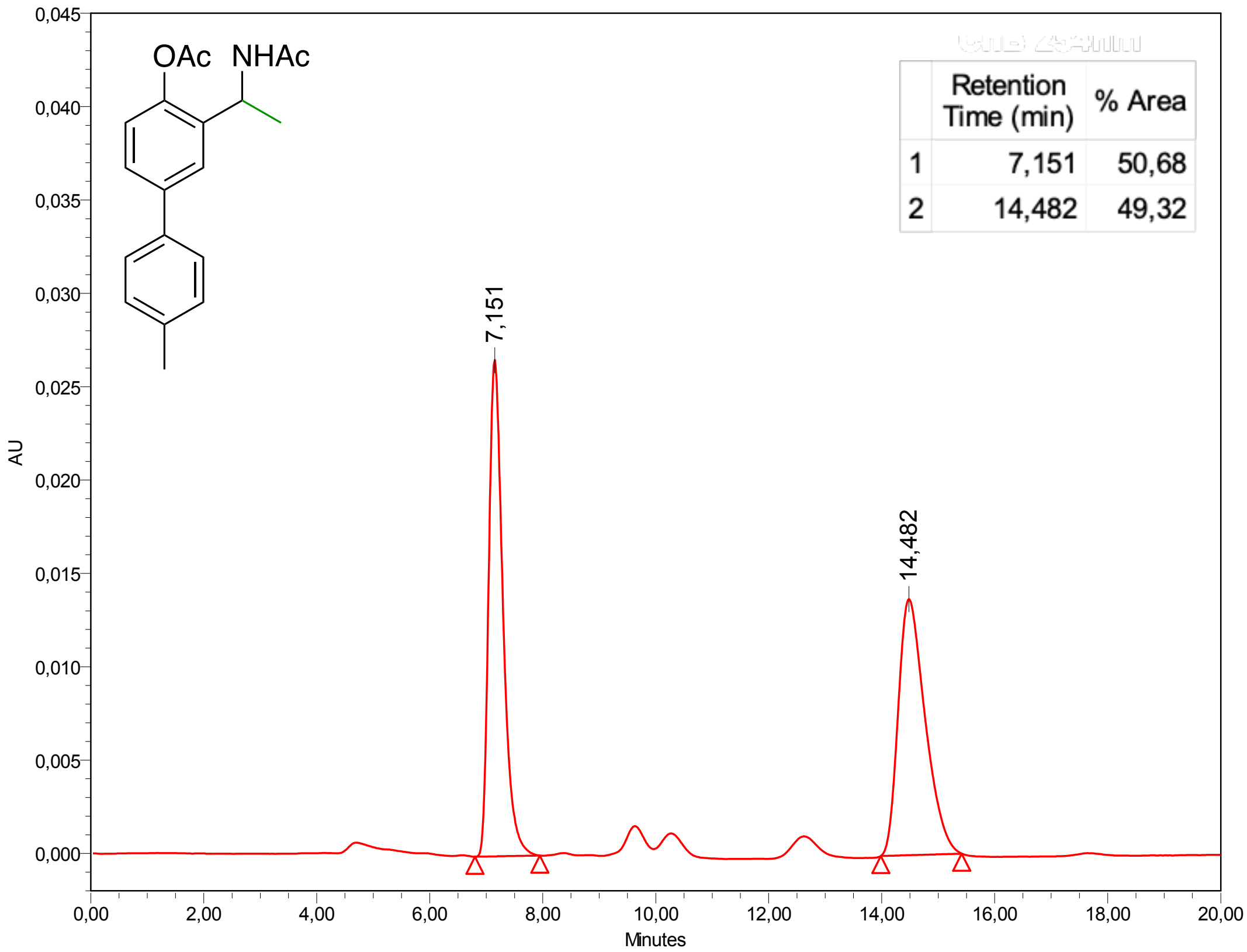
OAc NHAc
Retention Time (min) | % Area
1 | 7,151 | 50,68
2 | 14,482 | 49,32
7,151
14,482
AU
Minutes

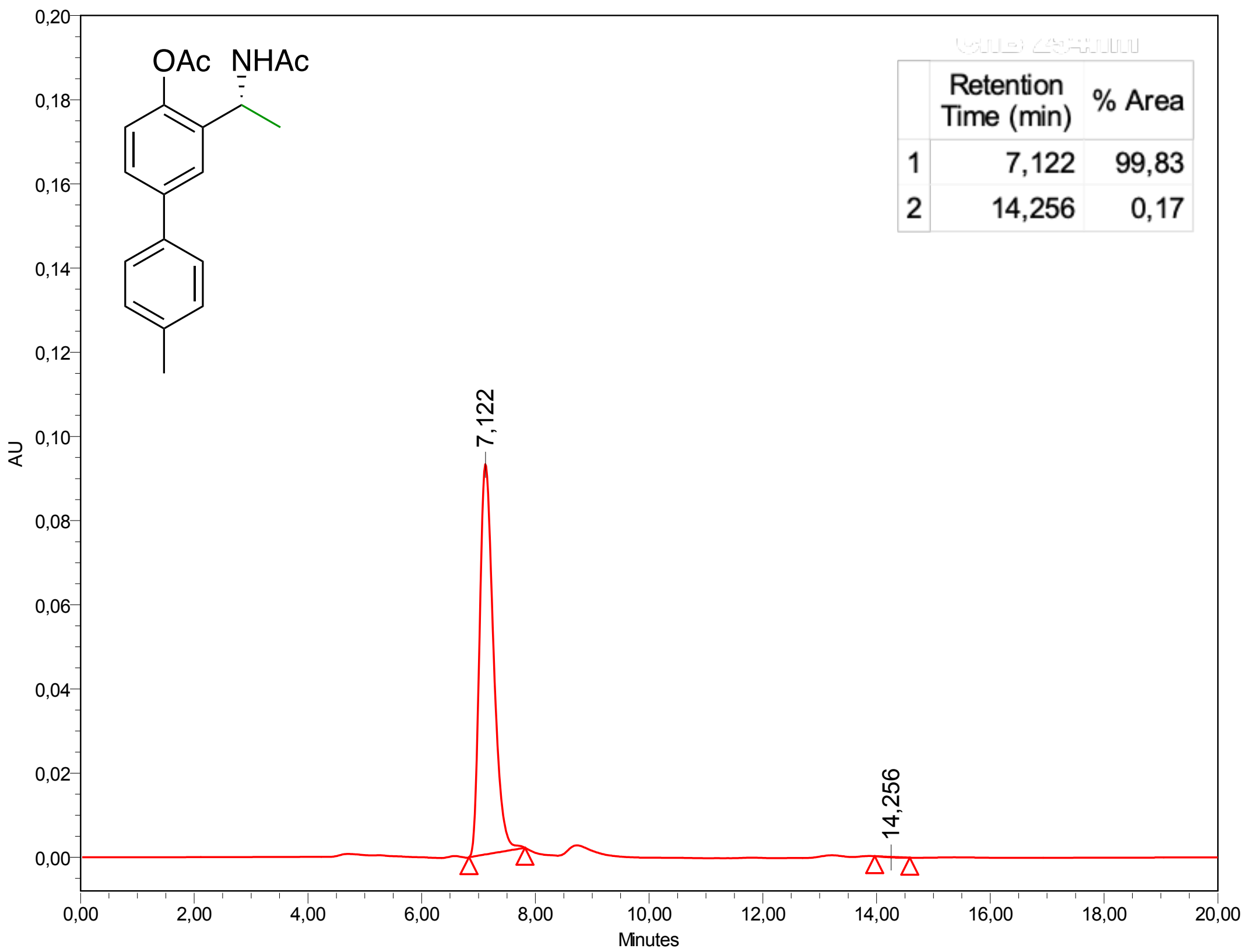
OAc NHAc
Retention Time (min) | % Area
1 | 7,122 | 99,83
2 | 14,256 | 0,17
7,122
14,256
AU
Minutes

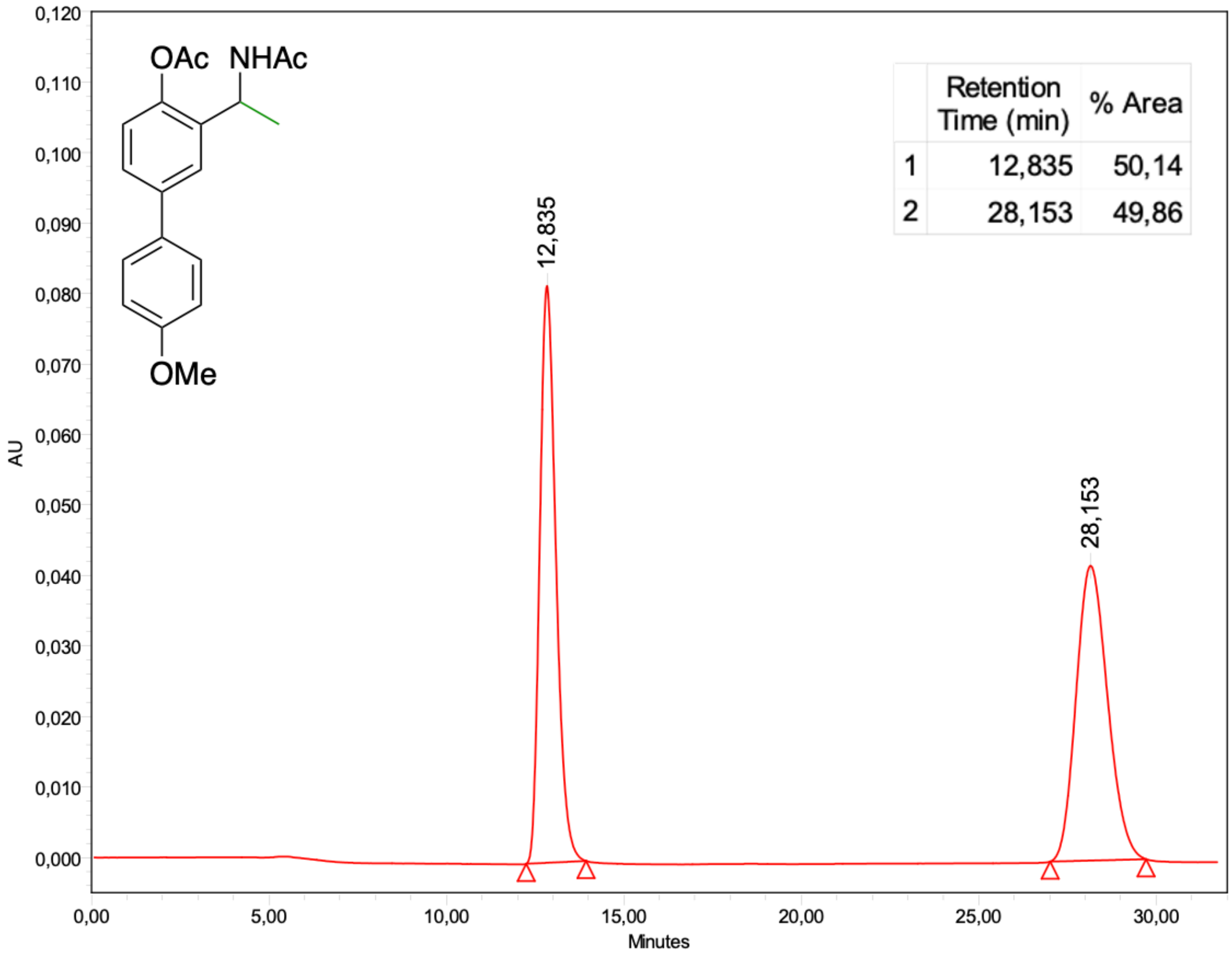

	Retention Time (min)	% Area
1	12,835	50,14
2	28,153	49,86

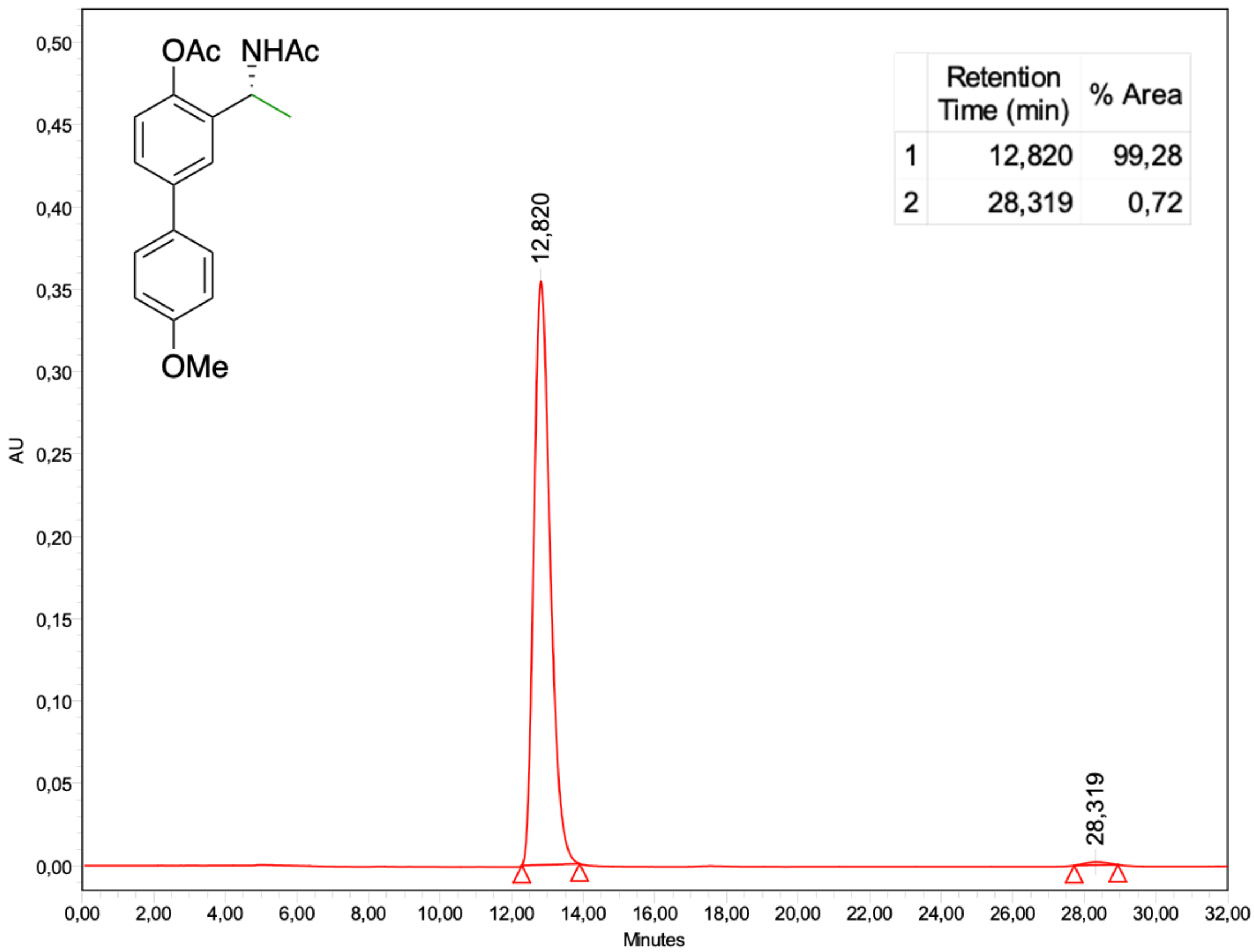

	Retention Time (min)	% Area
1	12,820	99,28
2	28,319	0,72

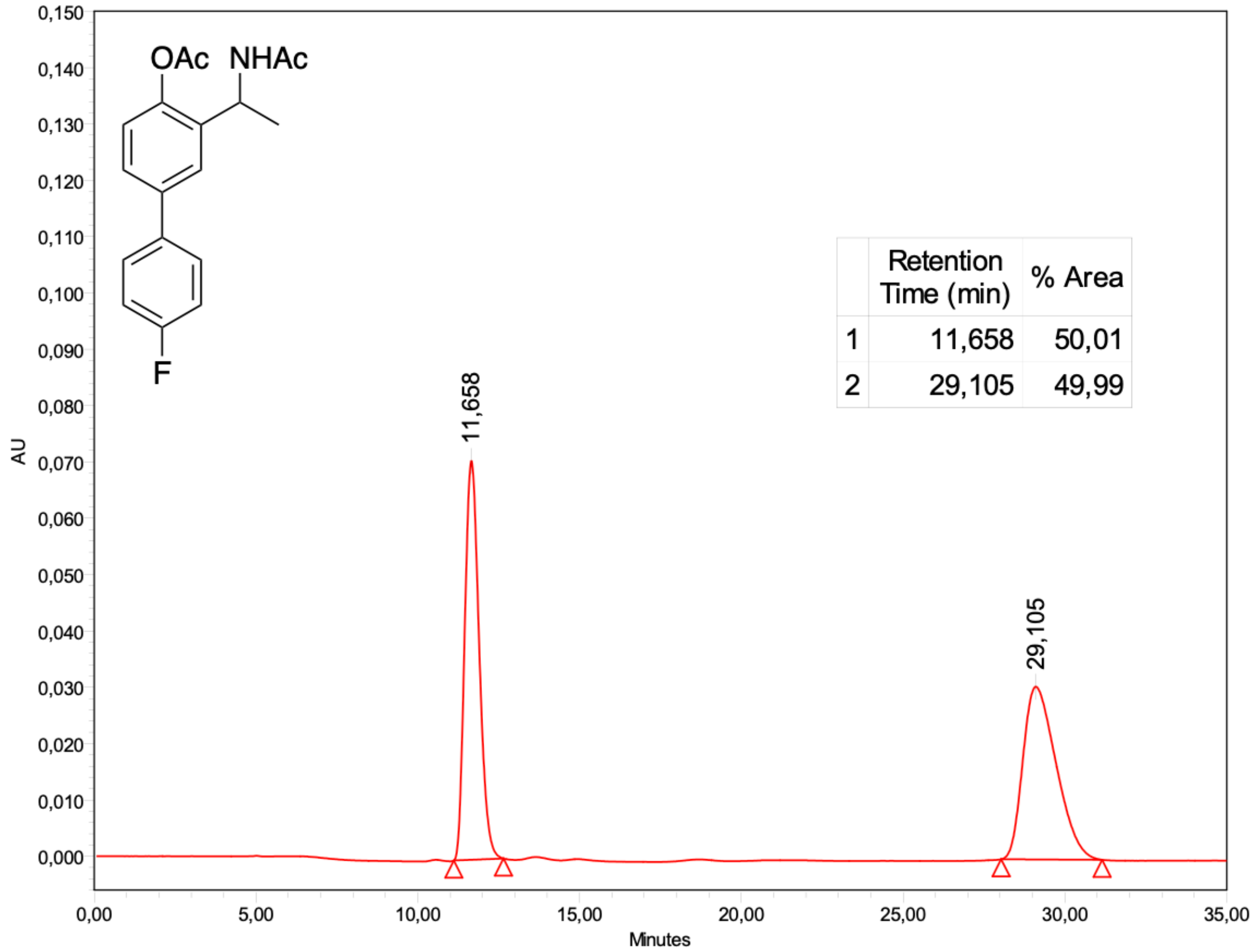
OAc
NHAc
F
Retention Time (min)
% Area
1 11,658 50,01
2 29,105 49,99
11,658
29,105
AU
Minutes
0,000
0,010
0,020
0,030
0,040
0,050
0,060
0,070
0,080
0,090
0,100
0,110
0,120
0,130
0,140
0,150
0,00
5,00
10,00
15,00
20,00
25,00
30,00
35,00

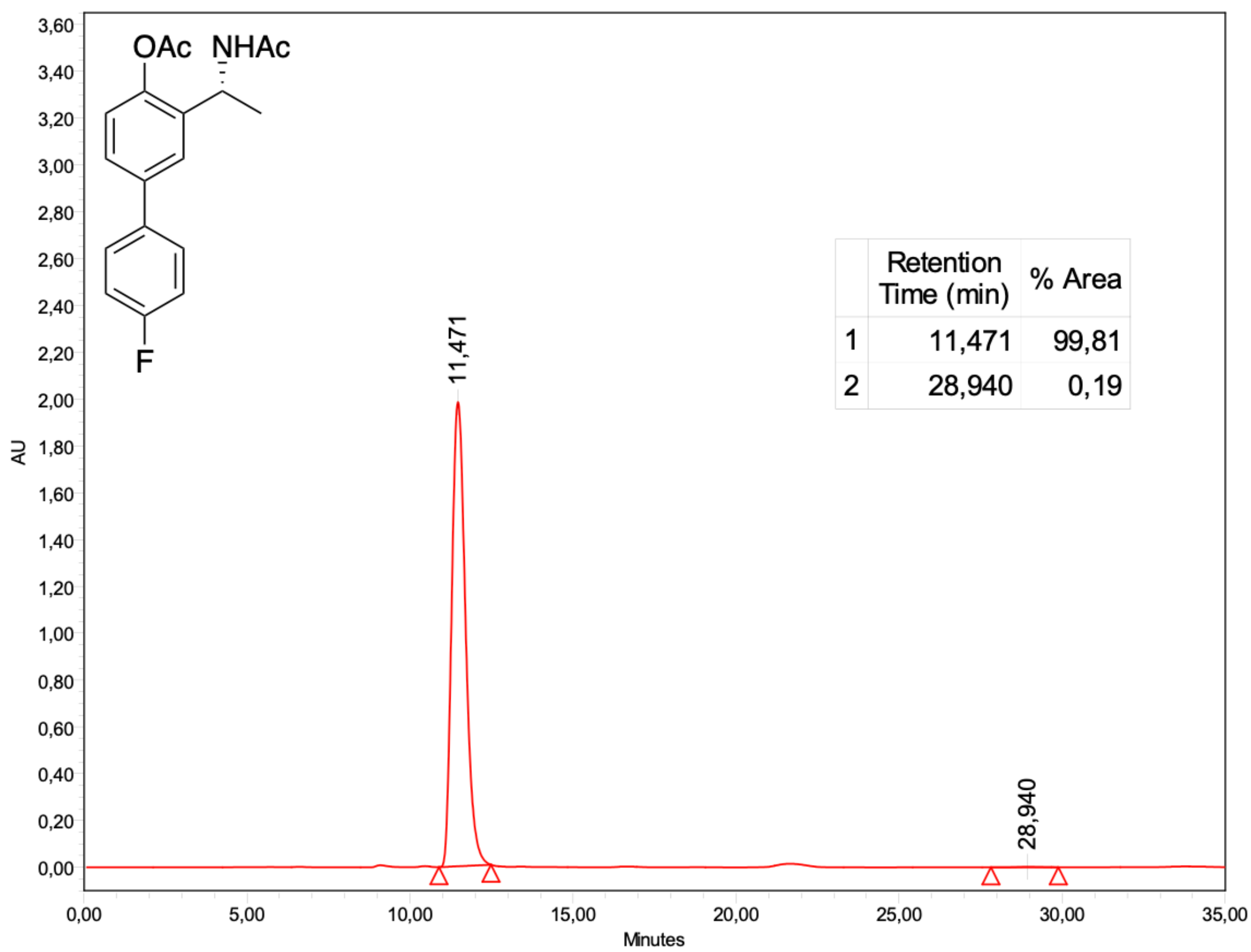
OAc
NHAc
F
Retention Time (min)
% Area
1 11,471 99,81
2 28,940 0,19
11,471
28,940
AU
Minutes
0,00
0,20
0,40
0,60
0,80
1,00
1,20
1,40
1,60
1,80
2,00
2,20
2,40
2,60
2,80
3,00
3,20
3,40
3,60
0,00
5,00
10,00
15,00
20,00
25,00
30,00
35,00

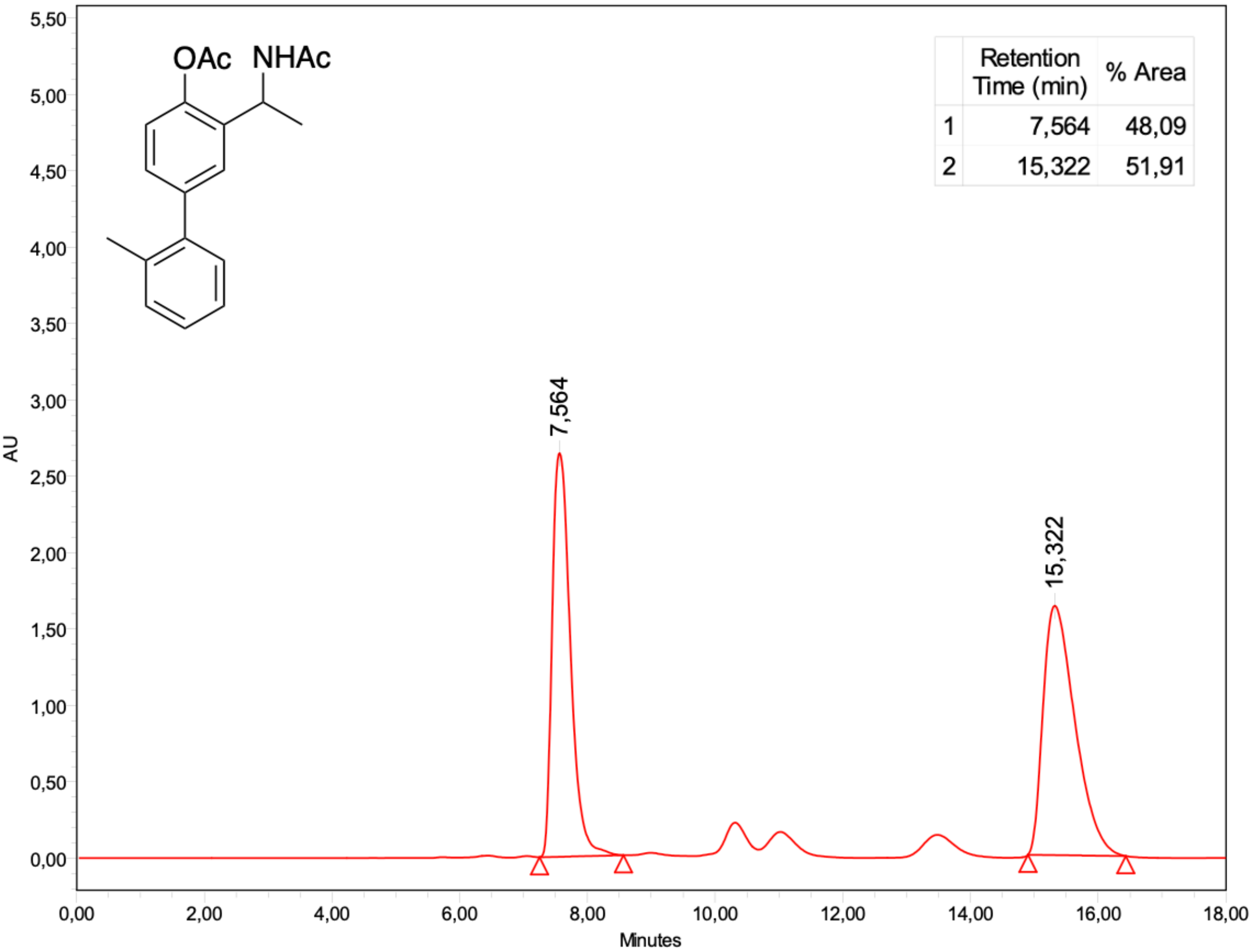
OAc
NHAc
Retention Time (min)
% Area
1 7,564 48,09
2 15,322 51,91
7,564
15,322
AU
Minutes

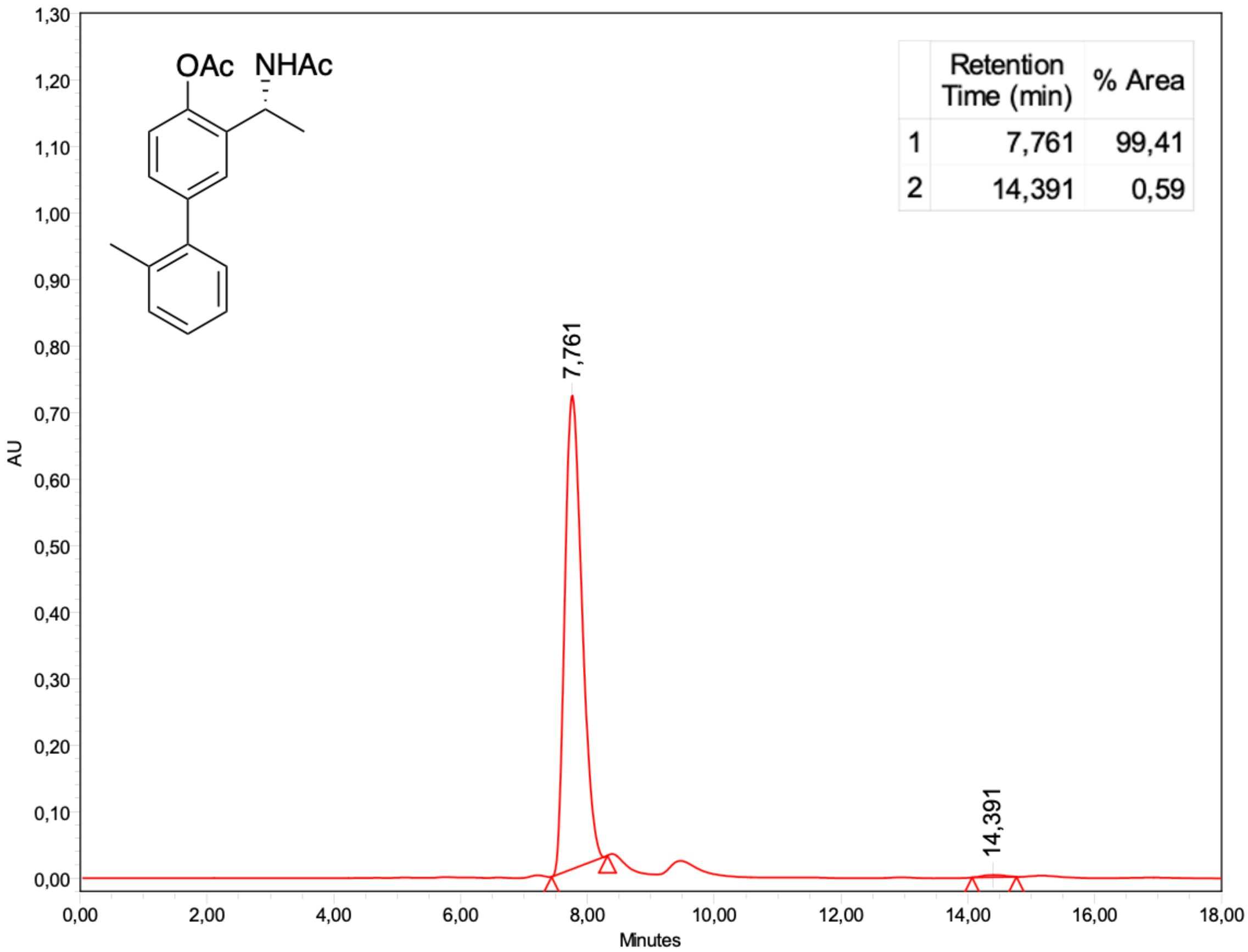
OAc
NHAc
Retention Time (min)
% Area
1 7,761 99,41
2 14,391 0,59
7,761
14,391
AU
Minutes

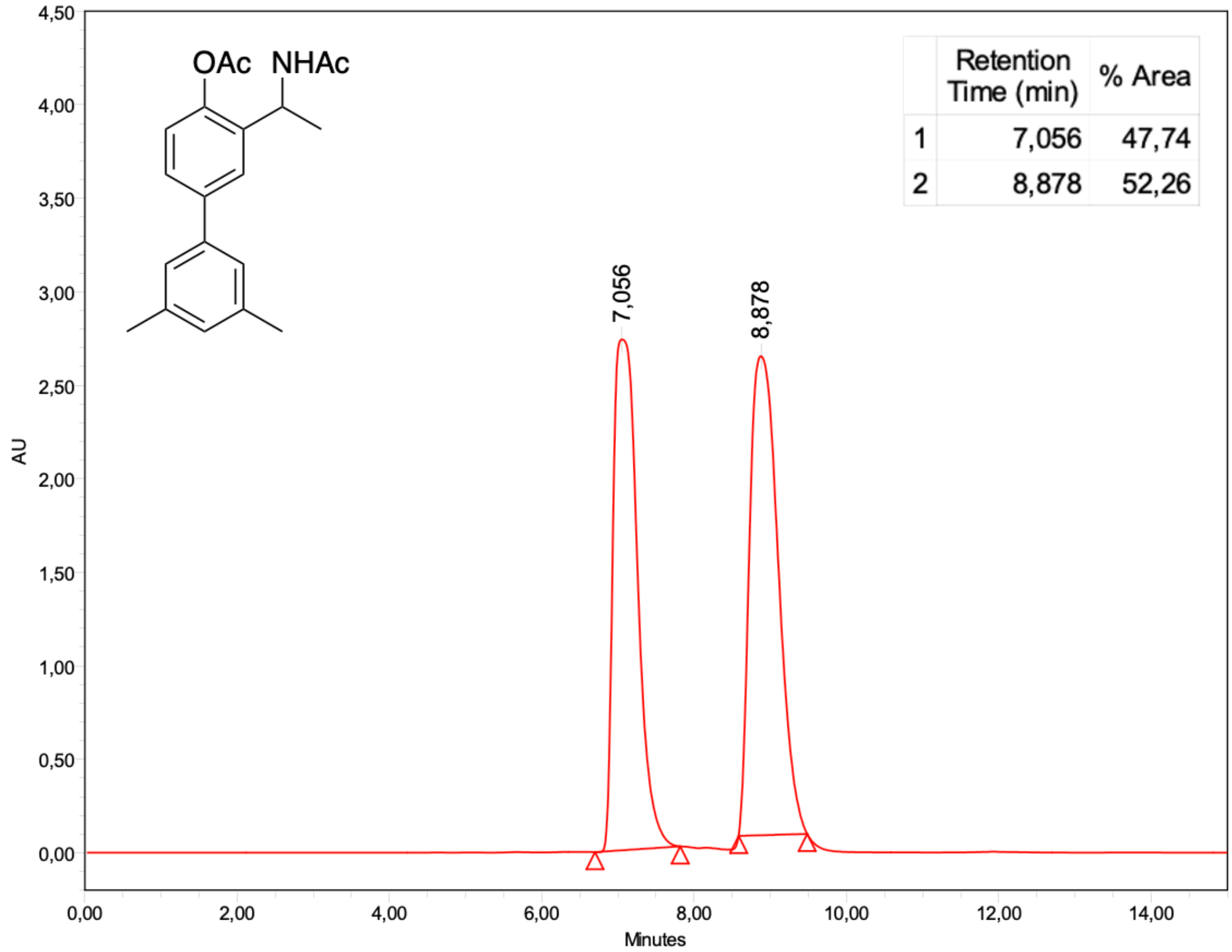

	Retention Time (min)	% Area
1	7,056	47,74
2	8,878	52,26

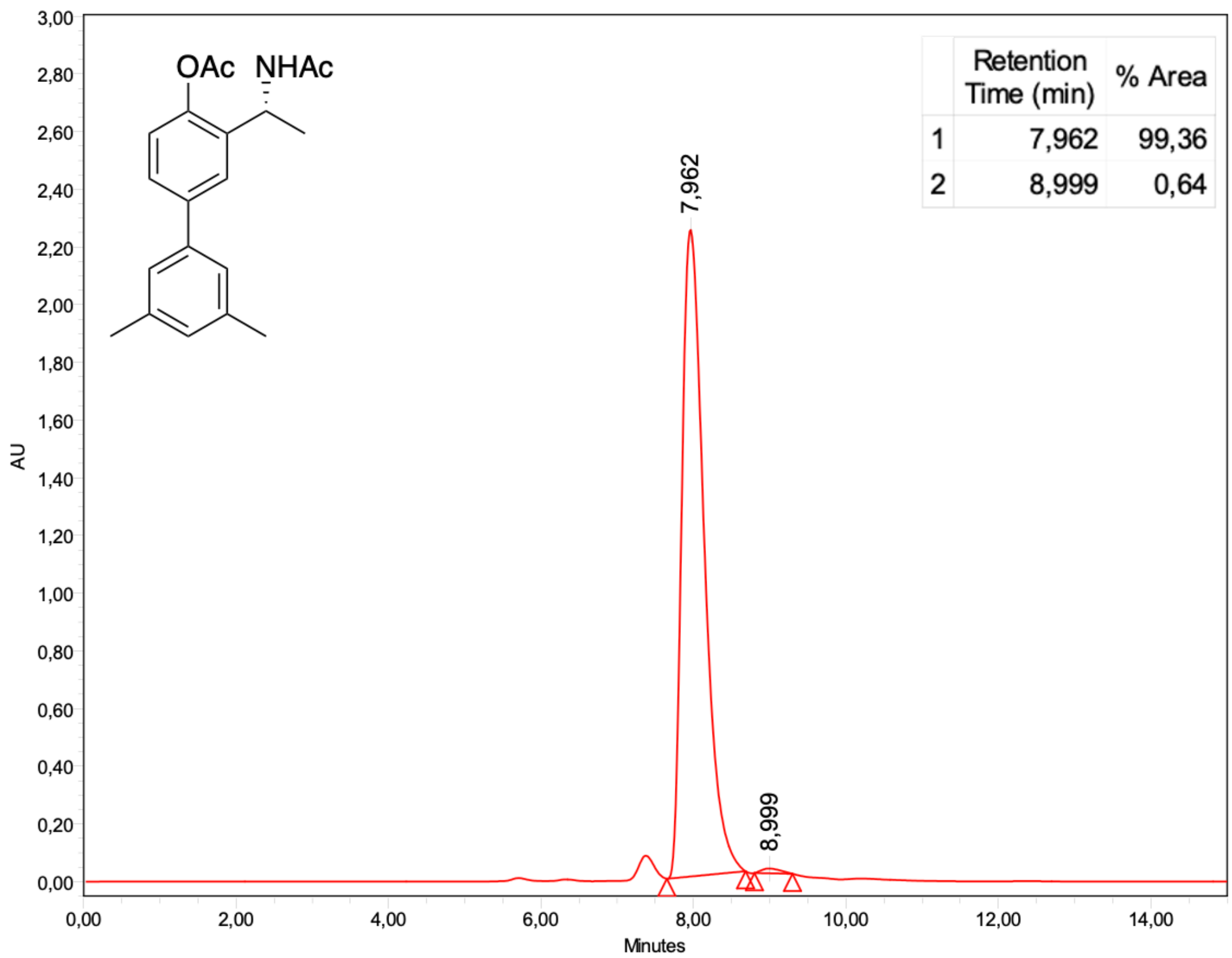

	Retention Time (min)	% Area
1	7,962	99,36
2	8,999	0,64

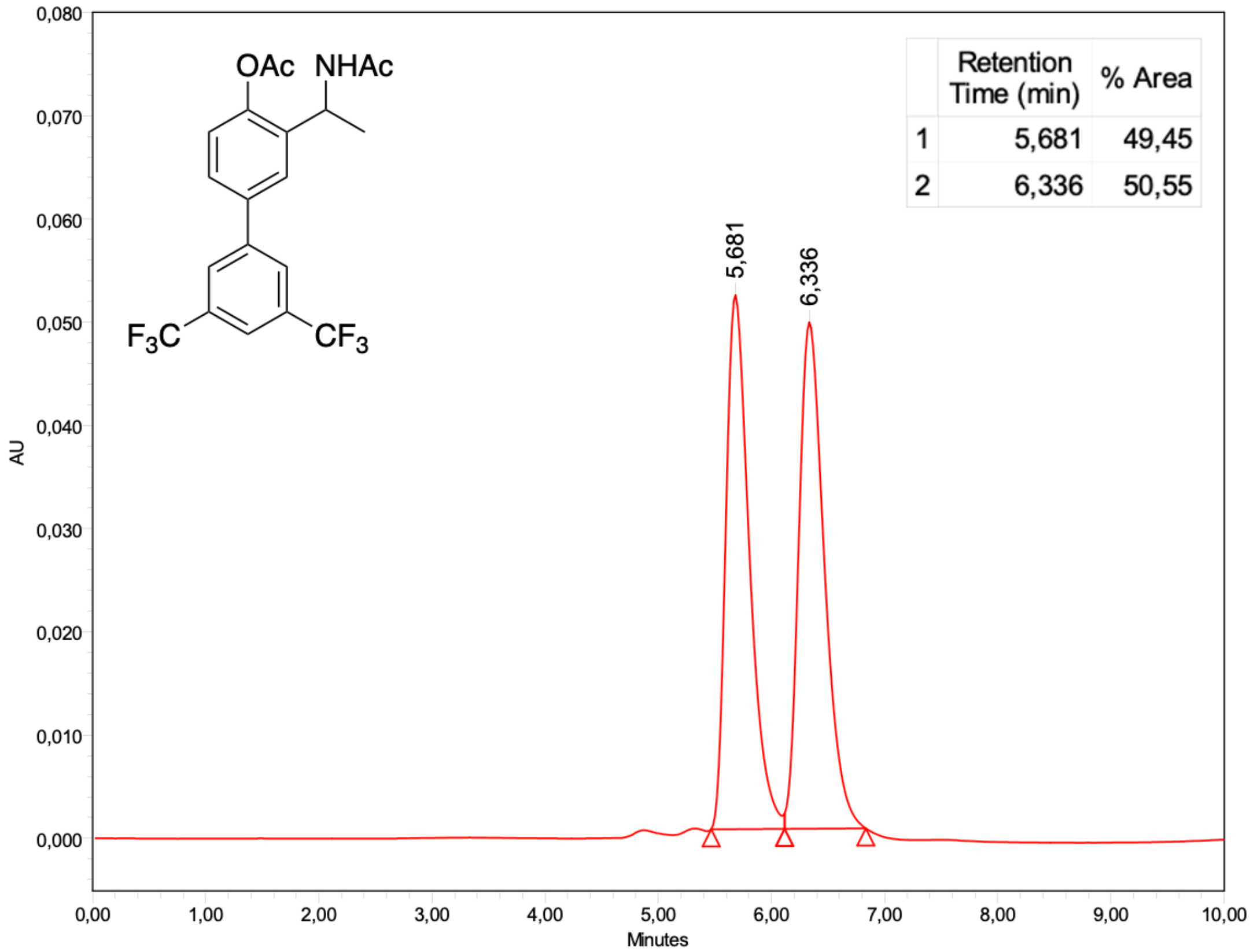
OAc NHAc
F3C CF3
Retention Time (min) | % Area
1 5,681 49,45
2 6,336 50,55
5,681
6,336
AU
Minutes

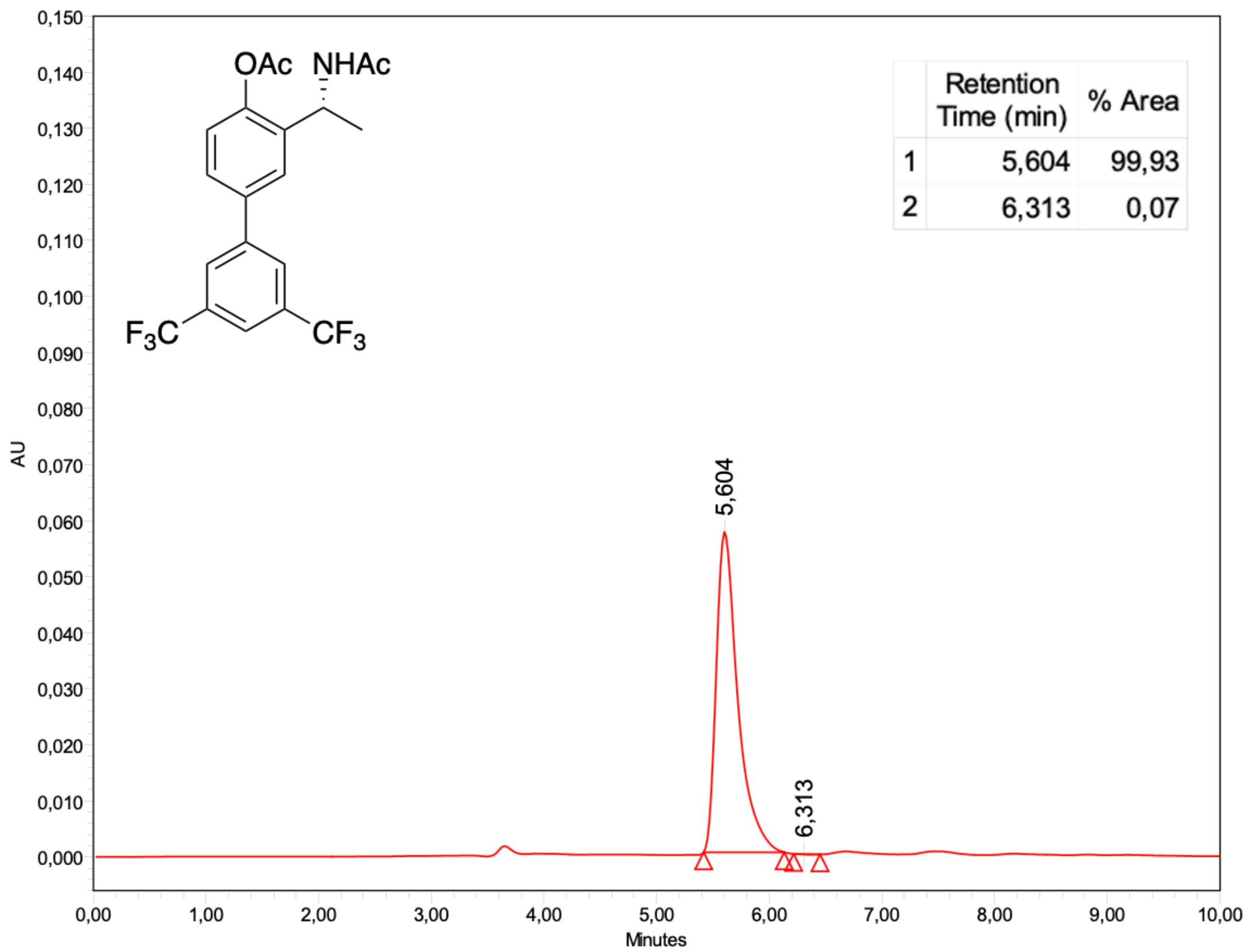
OAc NHAc
F3C CF3
Retention Time (min) | % Area
1 5,604 99,93
2 6,313 0,07
5,604
6,313
AU
Minutes

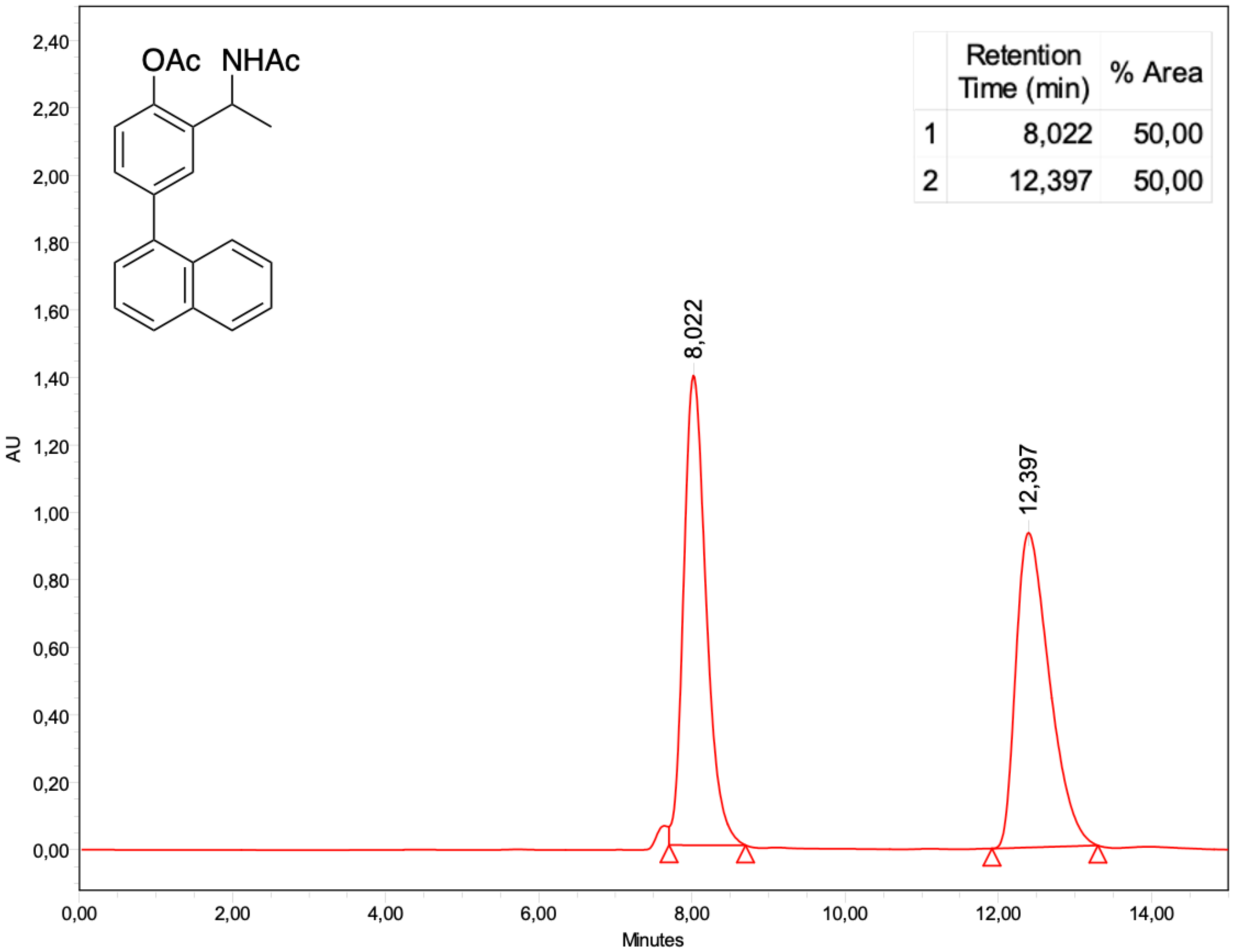

	Retention Time (min)	% Area
1	8,022	50,00
2	12,397	50,00

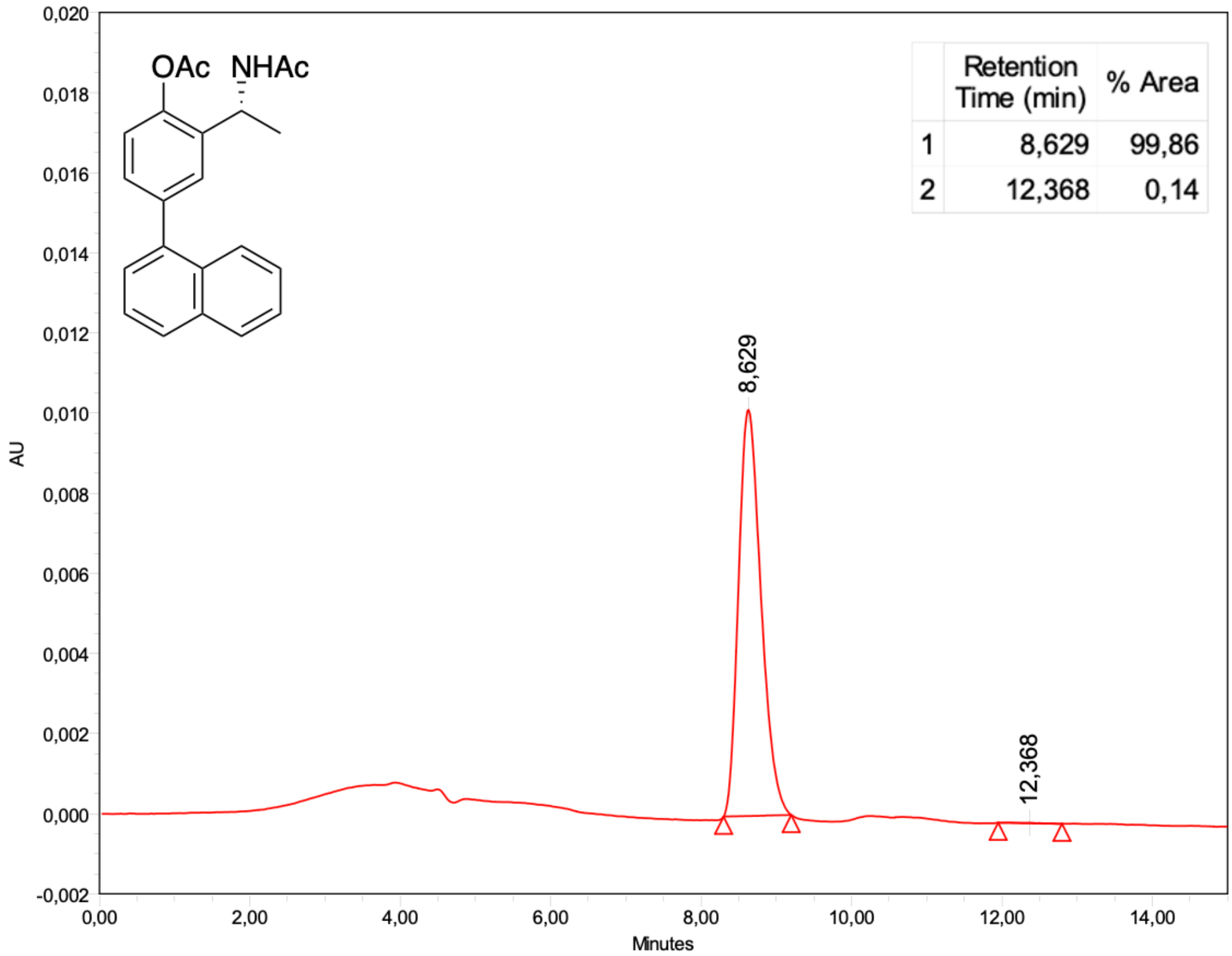

	Retention Time (min)	% Area
1	8,629	99,86
2	12,368	0,14

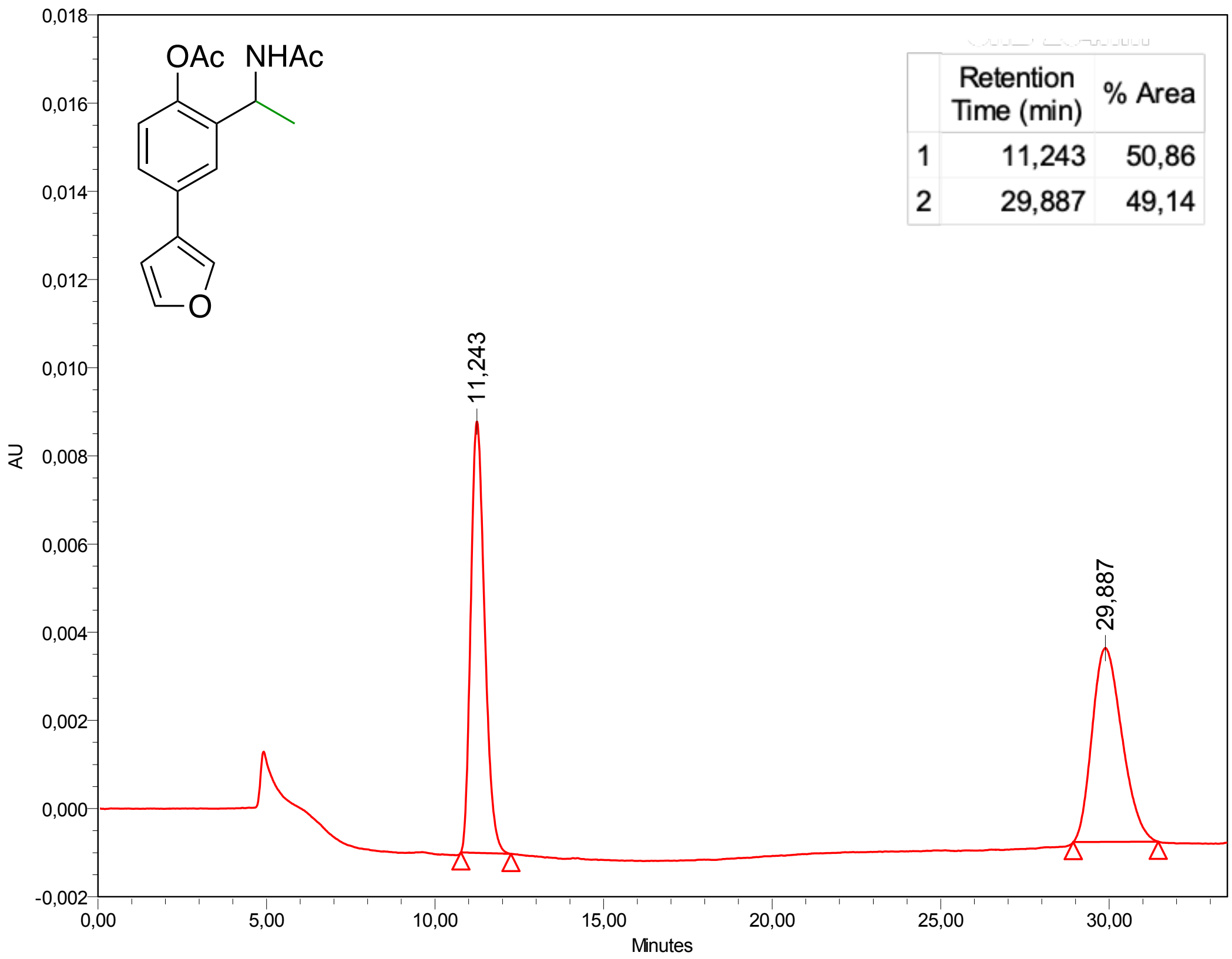
OAc
NHAc
O
Retention Time (min)
% Area
1
11,243
50,86
2
29,887
49,14
11,243
29,887
AU
Minutes

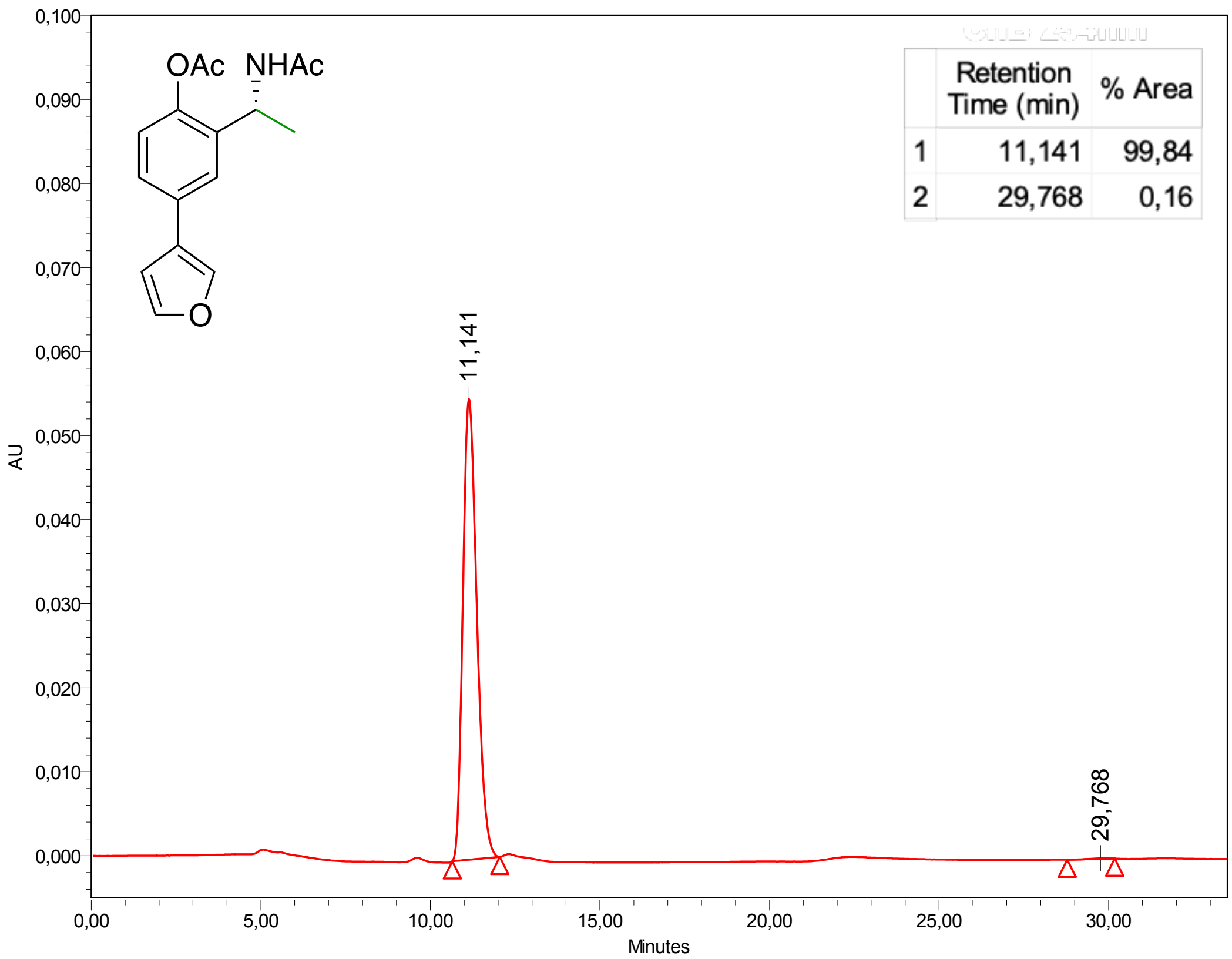
OAc
NHAc
O
Retention Time (min)
% Area
1
11,141
99,84
2
29,768
0,16
11,141
29,768
AU
Minutes

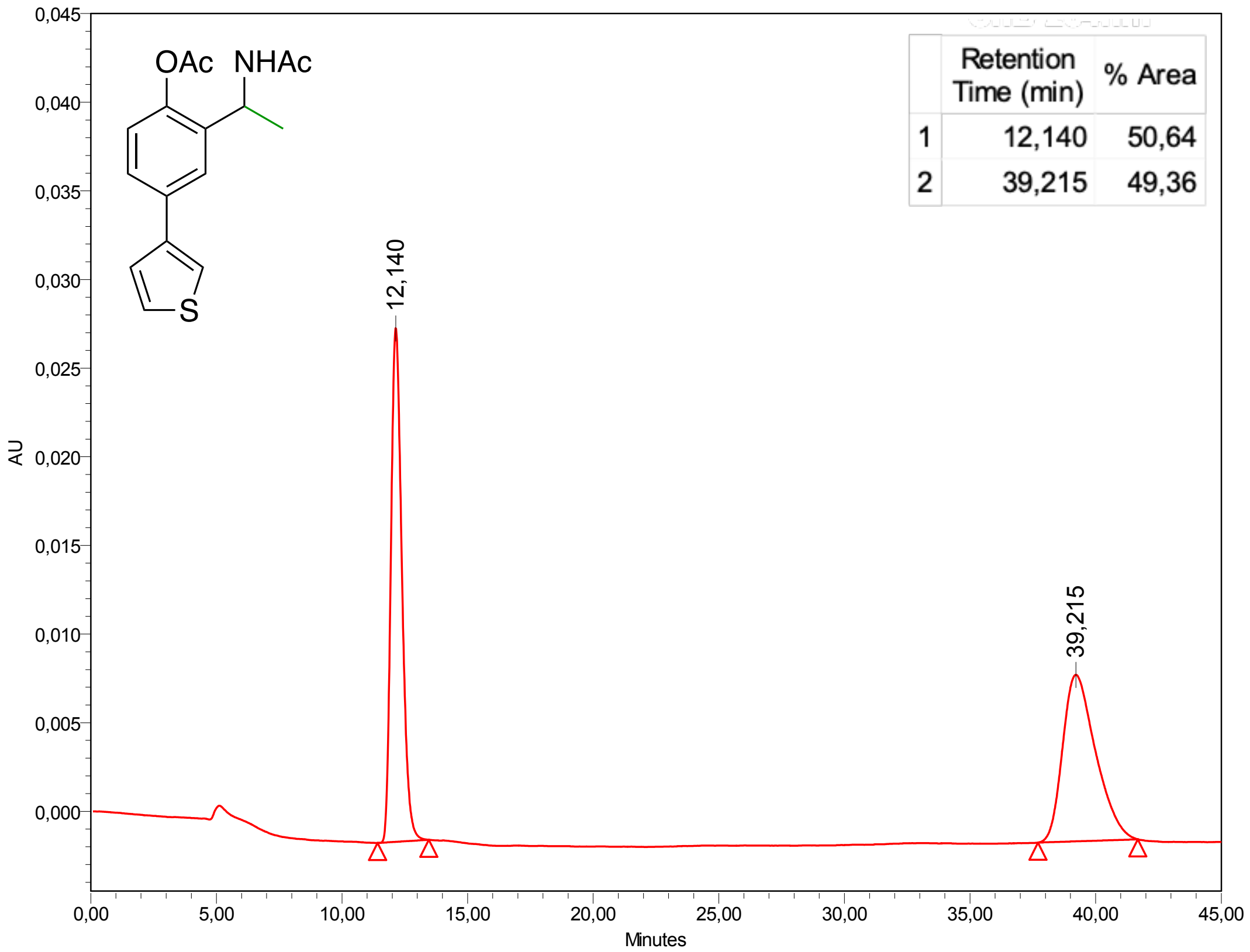
OAc NHAc
S
Retention Time (min) | % Area
1 | 12,140 | 50,64
2 | 39,215 | 49,36
12,140
39,215
AU
Minutes

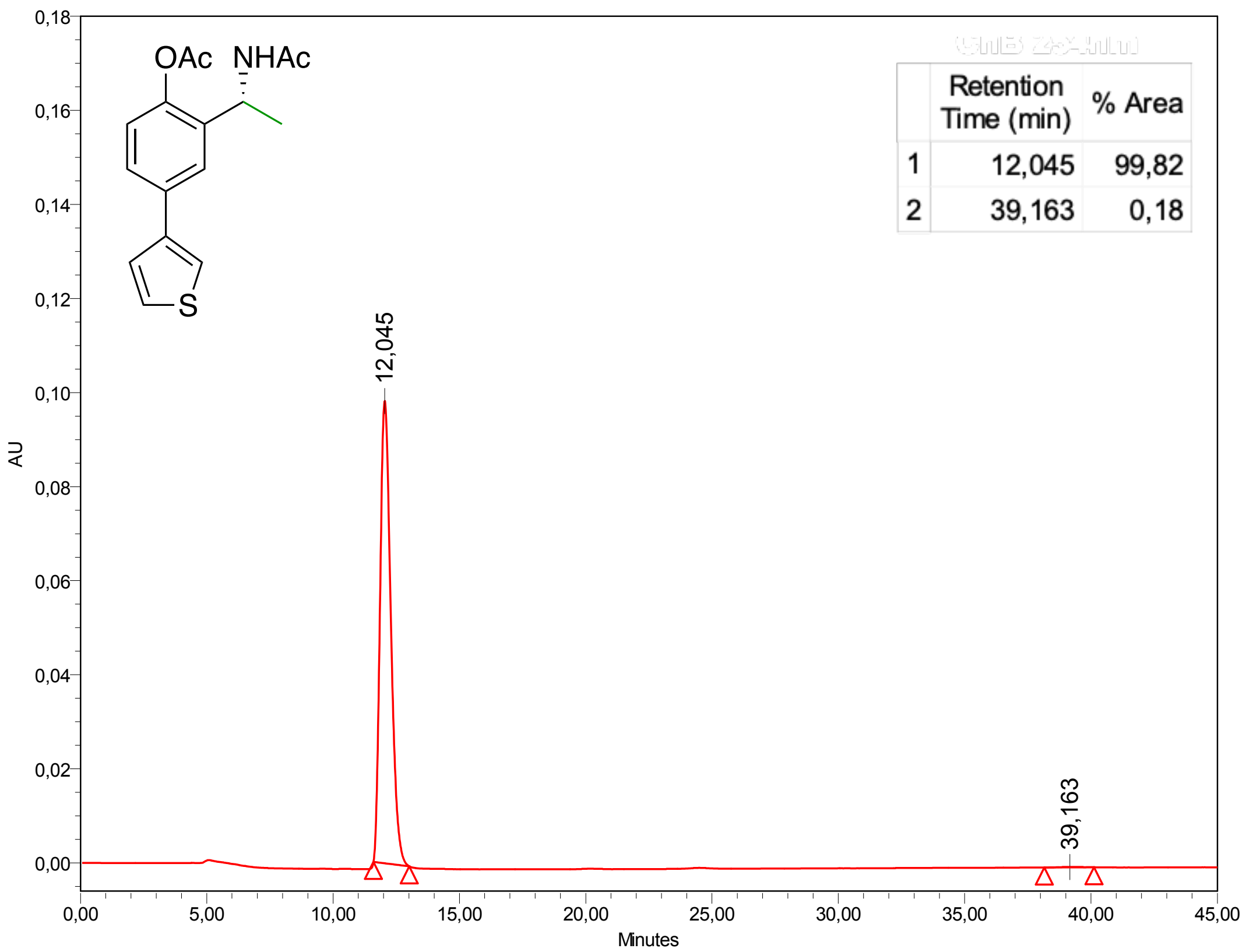
OAc NHAc
S
Retention Time (min) | % Area
1 | 12,045 | 99,82
2 | 39,163 | 0,18
12,045
39,163
AU
Minutes

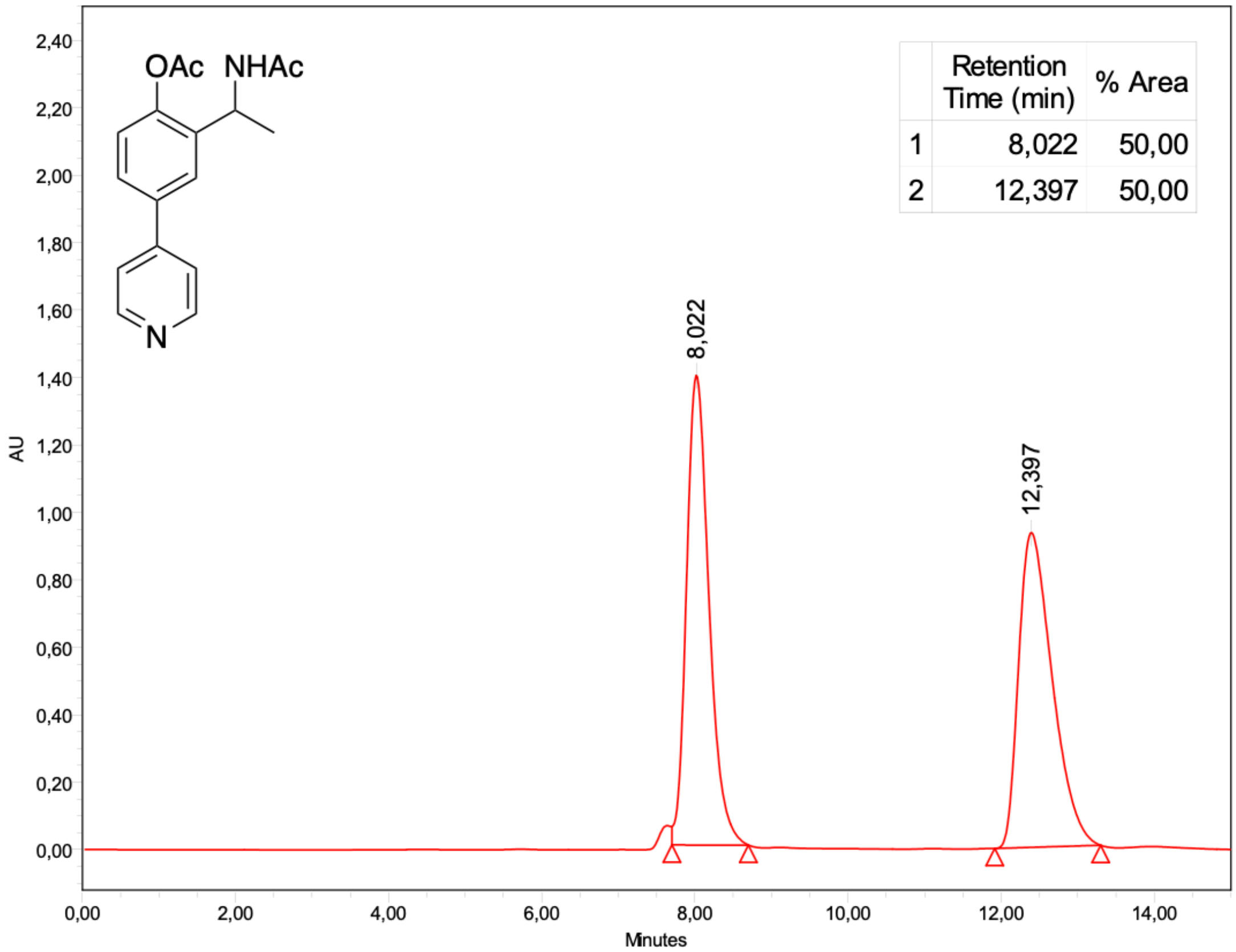
OAc NHAc
N
Retention Time (min) | % Area
1 8,022 50,00
2 12,397 50,00
8,022
12,397
AU
2,40 2,20 2,00 1,80 1,60 1,40 1,20 1,00 0,80 0,60 0,40 0,20 0,00
0,00 2,00 4,00 6,00 8,00 10,00 12,00 14,00
Minutes

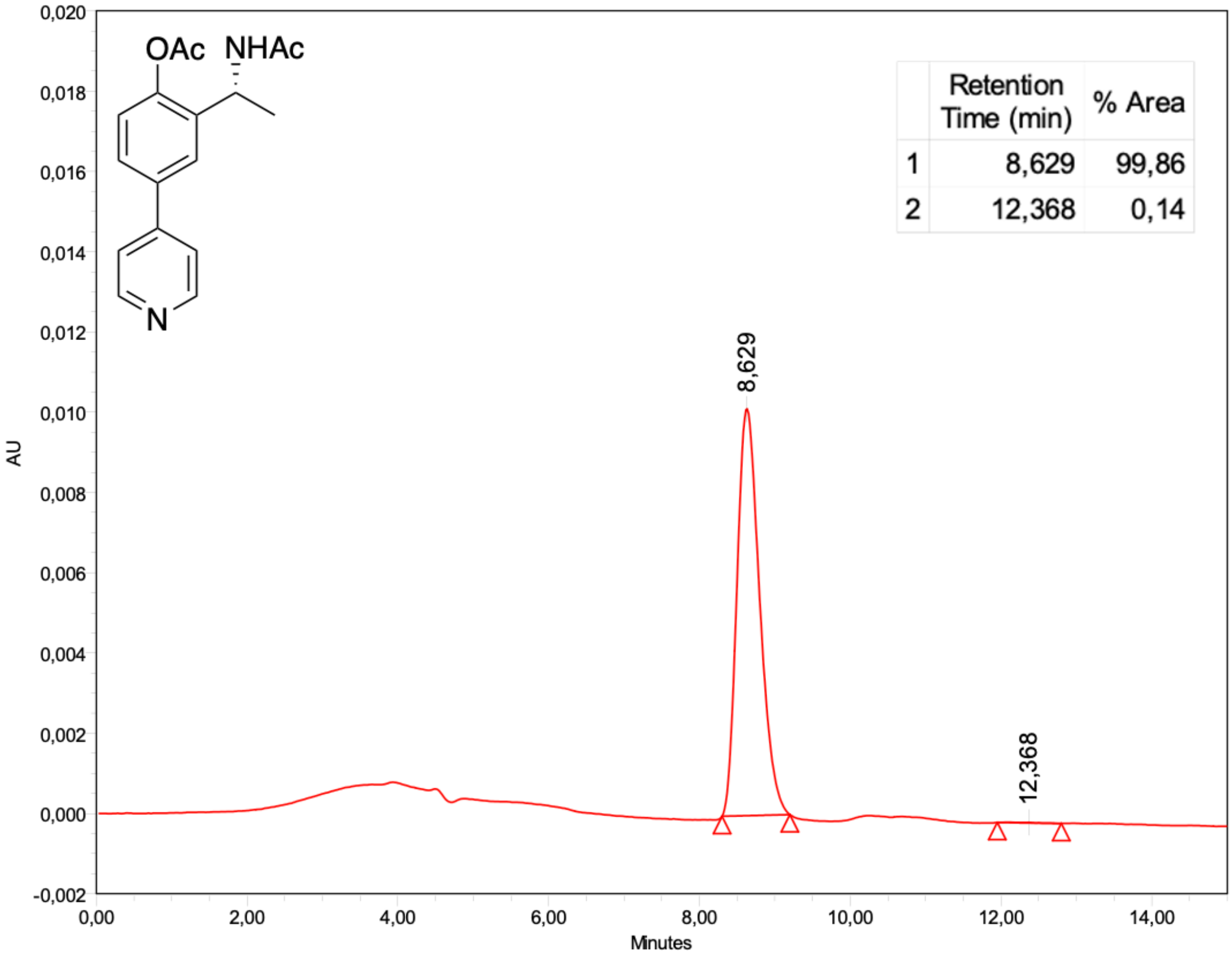
OAc NHAc
N
Retention Time (min) | % Area
1 8,629 99,86
2 12,368 0,14
8,629
12,368
AU
0,020 0,018 0,016 0,014 0,012 0,010 0,008 0,006 0,004 0,002 0,000 -0,002
0,00 2,00 4,00 6,00 8,00 10,00 12,00 14,00
Minutes

VII. Crystallographic data

Crystal data for compound 2ad (CCDC 2549812)have been deposited at the Cambridge Crystallographic Data Centre. Single crystals of 2ad were selected, mounted and transferred into a cold nitrogen gas stream. Intensity data were collected with a Bruker VENTURE diffractometer equipped with a PHOTON III 28 detector using an Incoatec IμS DIAMOND II micro-focus CuKα source (λ = 1.54178 Å). Unit-cell parameter determination, data collection strategy, integration and absorption correction were carried out with the Bruker APEX3 suite of programs. The structure was solved with SHELXT and refined anisotropically by full-matrix least-squares methods with SHELXL using WinGX. The absolute structure was determined by anomalous scattering effects analysis, and the absolute configuration was then deduced.

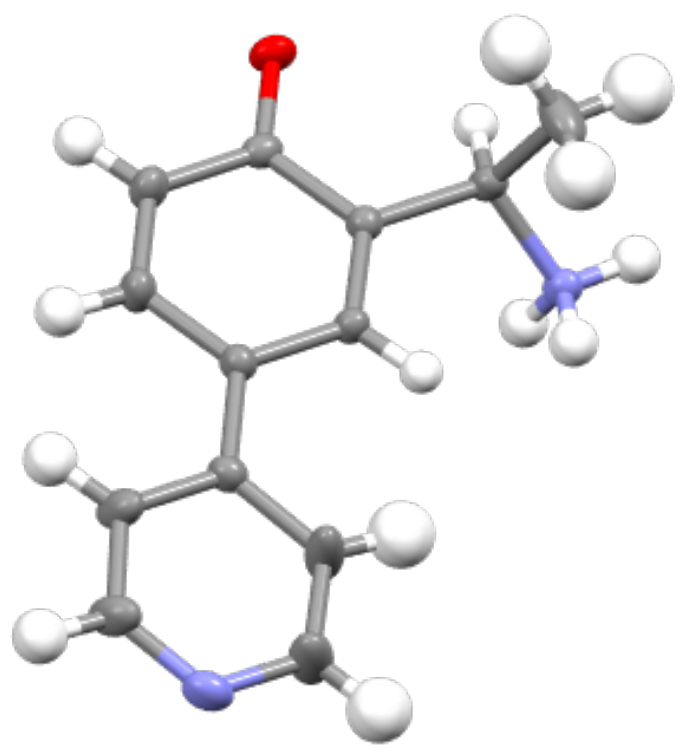

Crystal data structure and refinement for 2ad (CCDC 2549812)

Empirical formula	$C_{14}H_{18}N_2O_2$
Formula moiety	$C_{13}H_{14}N_2O$
Formula weight	214.11
Temperature (K)	295(2)
Crystal color/shape	colourless, prism
Crystal size (mm³)	0.30 × 0.30 × 0.10
Crystal system	Monoclinic
Space group	$P2_1$
a (Å)	7.7416(2)
b (Å)	7.48000(10)
c (Å)	11.4935(2)
α (°)	90
β (°)	97.8770(10)
γ (°)	90

Volume (Å^3)	659.28(2)
Z	2
Density (calcd, g·cm^{-3})	1.241
μ (mm^{-1})	0.675
F(000)	264
Radiation	Cu Kα (λ = 1.54178 Å)
Diffractometer	Bruker VENTURE
Detector	Bruker PHOTON III 28
Scan method	φ- and ω-scans
Absorption correction	multi-scan, SADABS; T_min/T_max = 0.63/0.75
Reflections collected	10618
Independent reflections	2289 (R_int = 0.0258)
Data/restraints/parameters	2289 / 1 / 166
Goodness-of-fit on F^2	1.037
Final R indexes [I > 2σ(I)]	R1 = 0.0364, wR2 = 0.1001
Final R indexes (all data)	R1 = 0.0368, wR2 = 0.1005
Flack parameter	–0.02(6)
Largest diff. peak/hole (e Å^{-3})	0.147 / –0.162

VIII. References

(1) Y. Lin, J-P. Wan, Y. Liu, "Cascade *in Situ* Iodination, Chromone Annulation, and Cyanation for Site-Selective Synthesis of 2-Cyanochromones" *J. Org. Chem.* **88**, (2023): 4017.

(2) R. Lin, S. Mitchell, T. Netscher, J. Medlock, R T. Stemmler, W. Bonrath, U. Létinois, J. Pérez-Ramírez, "Substrate substitution effects in the Fries rearrangement of aryl esters over zeolite catalysts" *Catal. Sci. Technol*. **10**, (2020): 4282.

(3) T. Javorskis, I. Karpavičienė, A. Jurys, G. Snarskis, R. Bukšnaitienė, E. Orentas, "An Enolate-Structure-Enabled Anionic Cascade Cyclization Reaction: Easy Access to Complex Scaffolds with Contiguous Six-, Five-, and Four-Membered Rings" *Angew. Chem. Int. Ed.* **59**, (2020): 20120.

(4) J. Ivanova, M. Abdoli, A. Nocentini, R. Žalubovskis, C. T. Supuran, "1,2,3-Benzoxathiazine-2,2-dioxides – effective inhibitors of human carbonic anhydrases" *Journal of Enzyme Inhibition and Medicinal Chemistry.* **38**, (2023): 2170370/1.

(5) M. Zhang, H. Li, H. Yang, Q. Yin, "Asymmetric Transfer Hydrogenation of Stable NH Imines for the Synthesis of Enantiopure α-Chiral Primary Amines" *Chin. J. Chem.* **42**, (2024): 2211.

(6) T. B. Nguyen, H. Bousserouel, Q. Wang, F. Guéritte, "Chiral Phosphoric Acid-Catalyzed Enantioselective Transfer Hydrogenation of ortho-Hydroxyaryl Alkyl N–H Ketimines" *Org. Lett.* **12**, (2010): 4705.

(7) L. Hu, Y. Zhang, W. Zhang, Q. Yin, X. Zhang, "Ruthenium-Catalyzed Direct Asymmetric Reductive Amination of Diaryl and Sterically Hindered Ketones with Ammonium Salts and H_2" *Angew. Chem. Int. Ed.* **59**, (2020): 5321.

(8) Gaussian 16, Revision C.01, M. J. Frisch, G. W. Trucks, H. B. Schlegel, G. E. Scuseria, M. A. Robb, J. R. Cheeseman, G. Scalmani, V. Barone, G. A. Petersson, H. Nakatsuji, X. Li, M. Caricato, A. V. Marenich, J. Bloino, B. G. Janesko, R. Gomperts, B. Mennucci, H. P. Hratchian, J. V. Ortiz, A. F. Izmaylov, J. L. Sonnenberg, D. Williams-Young, F. Ding, F. Lipparini, F. Egidi, J. Goings, B. Peng, A. Petrone, T. Henderson, D. Ranasinghe, V. G. Zakrzewski, J. Gao, N. Rega, G. Zheng, W. Liang, M. Hada, M. Ehara, K. Toyota, R. Fukuda, J. Hasegawa, M. Ishida, T. Nakajima, Y. Honda, O. Kitao, H. Nakai, T. Vreven, K. Throssell, J. A. Montgomery, Jr., J. E. Peralta, F. Ogliaro, M. J. Bearpark, J. J. Heyd, E. N. Brothers, K. N. Kudin, V. N. Staroverov, T. A. Keith, R. Kobayashi, J. Normand, K. Raghavachari, A. P. Rendell, J. C. Burant, S. S. Iyengar, J. Tomasi, M. Cossi, J. M. Millam, M. Klene, C. Adamo, R. Cammi, J. W. Ochterski, R. L. Martin, K. Morokuma, O. Farkas, J. B. Foresman, and D. J. Fox, Gaussian, Inc., Wallingford CT, 2019.

(9) Y. Zhao, D. G. Truhlar, "The M06 suite of density functionals for main group thermochemistry, thermochemical kinetics, noncovalent interactions, excited states, and transition elements: two new functionals and systematic testing of four M06-class functionals and 12 other functionals" *Theor. Chem. Acc*. **120**, (2008): 215.

(10) S. Grimme, J. Antony, S. Ehrlich, H. Krieg, "A consistent and accurate ab initio parameterization of density functional dispersion correction (DFT-D) for the 94 elements H-Pu" *J. Chem. Phys*. **132**, (2010): 154104.

(11) S. Miertus, E. Scrocco, J. Tomasi, "Electrostatic interaction of a solute with a continuum. A direct utilization of AB initio molecular potentials for the prevision of solvent effects" *Chem. Phys*. **55**, (1981):117.

(12) G. Scalmani, M. J. Frisch, B. Mennucci, J. Tomasi, R. Cammi, V. Barone, "Geometries and properties of excited states in the gas phase and in solution: Theory and application of a time-dependent density functional theory polarizable continuum model" *J. Chem. Phys*. **124**, (2006):094107.
(13) P. J. Hay, W. R. Wadt, "Ab initio effective core potentials for molecular calculations – potentials for the transition-metal atoms Sc to Hg". *J. Chem. Phys.* **82**, (1985):270.
(14) F. Weigend, R. Ahlrichs, "Balanced basis sets of split valence, triple zeta valence and quadruple zeta valence quality for H to Rn: Design and assessment of accuracy" *Phys. Chem. Chem. Phys.* **7** (2005):3297.
